# Identification of Diagnostic Markers in Infantile Hemangiomas

**DOI:** 10.1155/2022/9395876

**Published:** 2022-12-01

**Authors:** Sicong Huang, Ruiqi Chen, Shihua Gao, Yongjie Shi, Qiwen Xiao, Qiang Zhou, Jie Wei, Jiale Kang, Weimin Sun, Yingyu Hu, Gang Shen, Hongyun Jia

**Affiliations:** ^1^Department of Clinical Laboratory, The Second Affiliated Hospital of Guangzhou Medical University, Guangzhou, Guangdong 510260, China; ^2^Department of Transfusion Medicine, The First Affiliated Hospital of Guangzhou Medical University, Guangzhou, Guangdong 510120, China; ^3^Guangzhou University of Chinese Medicine, Guangzhou, Guangdong 510405, China; ^4^Department of Hospital Management, Southern Medical University, Guangzhou, Guangdong 510515, China; ^5^Department of Interventional Hemangioma, Children's Hospital, Capital Institute of Pediatrics, Beijing 100020, China

## Abstract

**Background:**

Infantile Hemangiomas (IHs) are common benign vascular tumors of infancy that may have serious consequences. The research on diagnostic markers for IHs is scarce.

**Methods:**

The “limma” *R* package was applied to identify differentially expressed genes (DEGs) in developing IHs. Plugin ClueGO in Cytoscape software performed functional enrichment of DEGs. The Search Tool for Retrieving Interacting Genes (STRING) database was utilized to construct the PPI network. The least absolute shrinkage and selection operator (LASSO) regression model and support vector machine recursive feature elimination (SVM-RFE) analysis were used to identify diagnostic genes for IHs. The receiver operating characteristic (ROC) curve evaluated diagnostic genes' discriminatory ability. Single-gene based on Gene Ontology (GO) and Kyoto Encyclopedia of Genes and Genomes (KEGG) was conducted by Gene Set Enrichment Analysis (GSEA). The chemicals related to the diagnostic genes were excavated by the Comparative Toxicogenomics Database (CTD). Finally, the online website Network Analyst was used to predict the transcription factors targeting the diagnostic genes.

**Results:**

A total of 205 DEGs were singled out from IHs samples of 6-, 12-, and 24-month-old infants. These genes principally participated in vasculogenesis and development-related, endothelial cell-related biological processes. Then we mined 127 interacting proteins and created a network with 127 nodes and 251 edges. Furthermore, LASSO and SVM-RRF algorithms identified five diagnostic genes, namely, TMEM2, GUCY1A2, ISL1, WARS, and STEAP4. ROC curve analysis results indicated that the diagnostic genes had a powerful ability to distinguish IHs samples from normal samples. Next, the results of GSEA for a single gene illustrated that all five diagnostic genes inhibited the “valine, leucine, and isoleucine degradation” pathway in the development of IHs. WARS, TMEM2, and STEAP4 activated the “blood vessel development” and “vasculature development” in IHs. Subsequently, inhibitors targeting TMEM2, GUCY1A2, ISL1, and STEAP4 were mined. Finally, 14 transcription factors regulating GUCY1A2, 14 transcription factors regulating STEAP4, and 26 transcription factors regulating ISL1 were predicted.

**Conclusion:**

This study identified five diagnostic markers for IHs and further explored the mechanisms and targeting drugs, providing a basis for diagnosing and treating IHs.

## 1. Introduction

Infants with infantile hemangiomas (IHs), with a morbidity rate of 4–10%, have developed one of the most prevalent benign vascular neoplasms in pediatrics [1]. IHs are clinically divided into the proliferation and involution stages and show a rapid proliferation during the first year of life. Where most of them multiply within 6 to 10 months, reaching the maximum size by 3 to 5 months old, and slowly regress before 4 years old in 90% of cases [2], while the remaining 10% of IHs may require intervention resulting from location, size, and complications, involving obstruction, dysfunction, ulcers, hypothyroidism, disfigurement, and other syndromes with life-threatening [3]. Head and neck ulcers are the most common severe anatomical deformations which heavily burden the child and their families [4, 5].

The formation of hemangiomas might be evoked by secondary physiological events in patients carrying germline risk mutations, such as perinatal hypoxia or mechanical stress during delivery [6]. Although the potential pathogenesis has not been fully elucidated, the aberrant responses of pluripotent stem cells to stimuli such as hypoxia [7], abnormal glucose metabolism, and the renin-angiotensin system [8] are considered critical pathogenic factors. The injury elicited by IHs depends on the depth and distribution of hemangioma-like lesions. IHs can occur in any organ system, and diagnosis may be delayed up to 3 months after birth in cases where deep local IHs present as a blue tumor with ill-defined boundaries [9].

The high expression of glucose transporter-1 (GLUT-1) protein draws a distinction between IHs and other vascular tumors or malformations in pathology [10]. As a classical pathological diagnostic marker of IHs, GLUT-1 is present at all stages of IHs and has limited significance for the early diagnosis of IHs. Clinical events summary proved if propranolol is used early in the IHs proliferation period, which could minimize the demand for surgical reduction and reconstructive surgery in the future [11]. Therefore, the diagnostic markers contributing to an early diagnosis of IHs are eager to be explored and will be a crucial component in disease treatment.

Recently, various new biomarkers were studied and examined for IHs diagnosis, prediction of therapeutic effect, and nosogenesis investigation [12–16]. Differentially expressed genes (DEGs) between normal and 6-, 12-, and 24-month-old IHs samples were screened from the Gene Expression Omnibus (GEO) database in the present study, and the front-rank genes were identified by DEGs coordinated with PPI network construction. Eventually, we confirmed TMEM2, GUCY1A2, ISL1, WARS, and STEAP4 for diagnosis and development of IHs based on receiver operator characteristic (ROC) curves analysis Gene Set Enrichment Analysis (GSEA), Gene-chemical interaction, and transcription factor prediction.

## 2. Materials and Methods

### 2.1. Data Source

The transcriptome data of the GSE127487 dataset [12] were obtained by downloading from the GEO database (https://www.ncbi.nlm.nih.gov/gds). Six normal skin samples, five hemangioma samples from 6-month-old infants, six hemangioma samples from 12-month-old infants, and six hemangioma samples from 24-month-old infants were selected for subsequent analysis. The hemangioma samples were all from infants who were not treated with propranolol.

### 2.2. Identification of DEGs in the Development of IHs

To acquire genes that were involved in the development of IHs, we first filtered genes that were differentially expressed between normal and 6-month-old IHs samples, normal and 12-month-old IHs samples, normal and 12-month-old IHs samples, respectively by “limma” *R* package (version 3.44.3), employing *P*-value <0.05 and |Log2FC| >2 as screening thresholds [17]. Then, the up- and down-regulated genes from the three groups were intersected separately. Finally, the common up- and down-regulated genes were obtained and considered candidate genes for the following analysis.

### 2.3. Functional Enrichment of DEGs

The above conjoint DEGs were submitted to Gene Ontology (GO) and Kyoto Encyclopedia of Genes and Genomes (KEGG) functional enrichment analysis in the plugin ClueGO of Cytoscape software (version 2.5.8) [18].

### 2.4. PPI Network Construction

The Search Tool for Retrieving Interacting Genes (STRING) database (https://string-db.org/) was utilized to construct the PPI network [19] after concealing the independent nodes. Nodes with an interaction confidence level greater than 0.4 were incorporated into the network. The data were introduced into Cytoscape software (version 2.5.8) for visual display.

### 2.5. Diagnostic Genes Screening and Verification

The least absolute shrinkage and selection operator (LASSO) is a high-dimensional data analysis method that synchronously conducts regularization and variable selection, which would enhance the prediction accuracy and validity [20]. The “glmnet” *R* package was utilized to execute the LASSO algorithm. Support Vector Machine-Recursive Feature Elimination (SVM-RFE) is a feature selection algorithm based on a support vector machine, which ranks the features based on deleting the recursive feature sequence [21]. The e1071 *R* package applied the SVM algorithm. Intersection genes identified from the two algorithms were deemed as characteristic genes. In addition, the area under the curve (AUC) of receiver operating characteristic (ROC) was computed by using the pROC package [22] to evaluate the diagnostic ability of potential diagnostic genes in discriminating hemangioma samples from normal samples.

### 2.6. Gene Set Enrichment Analysis

To explore the mechanisms of diagnostic genes in the development of IHs, we performed GSEA for a single gene in “clusterProfiler” and “org.Hs.eg.db” in *R*, took the expression value of each gene as the phenotype file and ranked the correlation coefficients between each gene and all genes in the gene sets. The threshold for enrichment significance was |NES| >1, and adjust *P*-value was <0.05.

### 2.7. Gene-Chemical Interaction and Transcription Factor Prediction

The chemicals related to the diagnostic genes were excavated by the Comparative Toxicogenomics Database (CTD) (https://ctdbase.org/) [23]. The online website Network Analyst (https://www.networkanalyst.ca/faces/home.xhtml) was used to predict the transcription factors targeting the diagnostic genes.

### 2.8. Statistical Analysis

All analyses were conducted using the *R* programming language, and the Wilcoxon test compared the data from different groups. In all analyses, *P*-values less than 0.05 were regarded as statistically significant.

## 3. Results

### 3.1. The Differentially Expressed Genes (DEGs) in the Development of IHs

**Figure 1:**
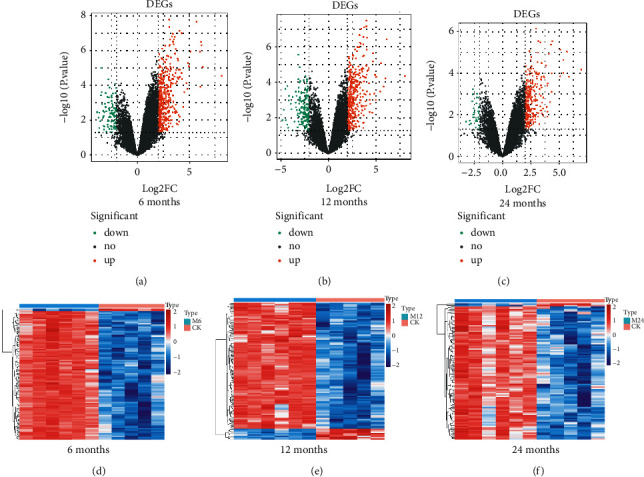
DEGs between IHs and normal. (a)–(c) The volcano plot of DEGs of IHs at 6, 12, and 24 months was compared with normal, where the green dots represent the down-regulated genes, and the red dots represent the genes that are up-regulated. The black dots represent the genes that are not significantly different. (d)–(f) Heatmaps of DEGs of IHs at 6, 12, and 24 months were compared with normal, where the aquamarine blue bar represents IHs and the red bar represents normal control samples.

**Figure 2:**
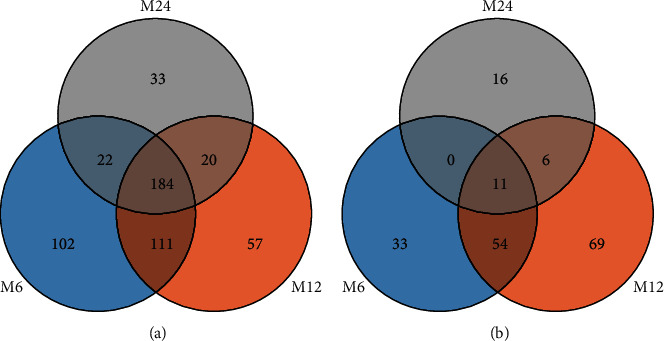
Venny maps of DEGs in GSE127487 gene chips. (a) Up-regulated genes of hemangioma at 6, 12, and 24 months. (b) Down-regulated genes of hemangioma at 6, 12, and 24 months.

**Figure 3:**
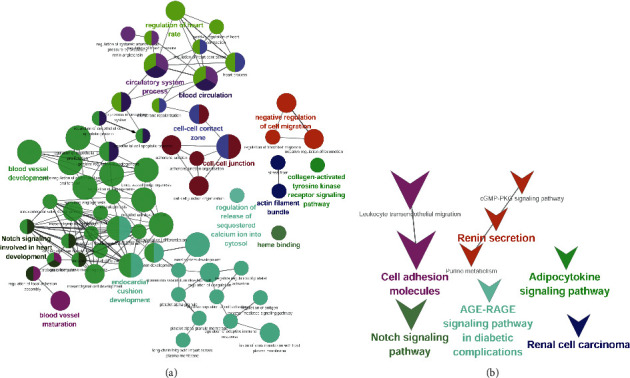
Analyses of candidate genes enriched in biological functions and signaling pathways. (a) GO enrichment network of candidate genes. (b) KEGG enrichment network of candidate genes.

Compared to normal samples, 527 DEGs were screened in the 6-month-old infant hemangioma samples, of which 429 were up-regulated and 98 were down-regulated (Figure 1(a), Supplementary Table S1). As to the 12-month-old infant hemangioma samples, 542 DEGs were identified, including 382 up-regulated and 160 down-regulated genes (Figure 1(b), Supplementary Table S2). For 24-month-old infants, 299 DEGs were mined, containing 266 up-regulated and 33 down-regulated genes (Figure 1(c), Supplementary Table S3). The TOP 100 DEGs in each group were also presented in a heat map separately (Figures 1(d)–1(f)). Then, we obtained 194 common up-regulated and 11 down-regulated genes by taking the intersection of up-regulated and down-regulated genes of the above three groups, respectively (Figures 2(a) and 2(b), Supplementary Table S4). These 205 common DEGs among the 6-, 12-, and 24-month-old infants were considered candidate genes for subsequent analysis. To investigate the function of these genes in the genesis of IHs, we performed functional enrichment analysis on these genes. As a result, 62 GO items and 9 KEGG pathways were enriched (Supplementary Table S5). Interestingly, we found that these genes were mainly involved in biological processes such as “blood vessel development,” “vasculogenesis,” “stress fiber,” “regulation of endothelial cell proliferation,” “blood vessel maturation,” “regulation of systemic arterial blood pressure by circulatory renin-angiotensin,” “sprouting angiogenesis,” “cell-cell junction,” “regulation of angiogenesis,” “regulation of adaptive immune response,” “vascular process in the circulatory system,” “endothelium development” and et al. (Figure 3(a)). At the same time, these genes were also associated with KEGG pathways such as “purine metabolism,” “cGMP-PKG signaling pathway,” “Notch signaling pathway,” “cell adhesion molecules,” “leukocyte transendothelial migration,” “adipocytokine signaling pathway,” “renin secretion” and et al. (Figure 3(b)). Hence, we speculated that these genes might impact the development of IHs via these processes.

### 3.2. Diagnostic Genes Identification

**Figure 4:**
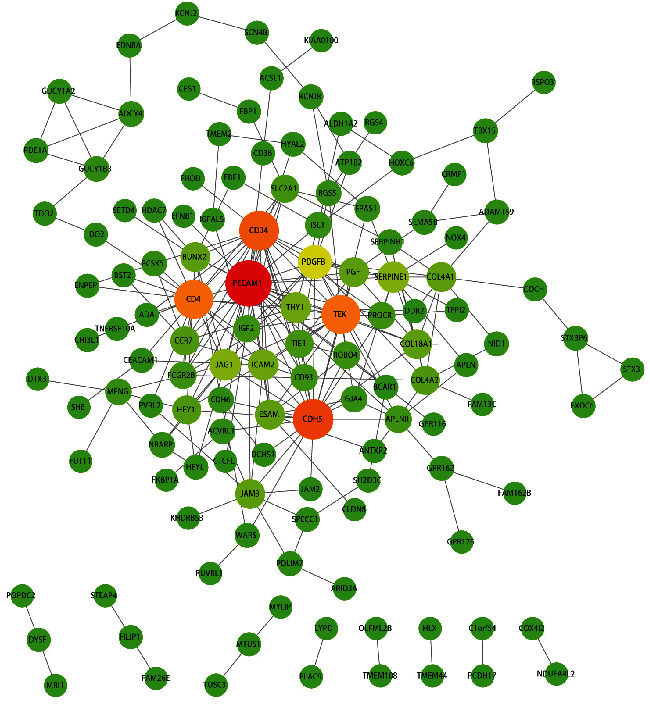
PPI network of DEGs. The increasing color from green to red, the higher connectivity of genes, and the greater the connection, the larger the point.

**Figure 5:**
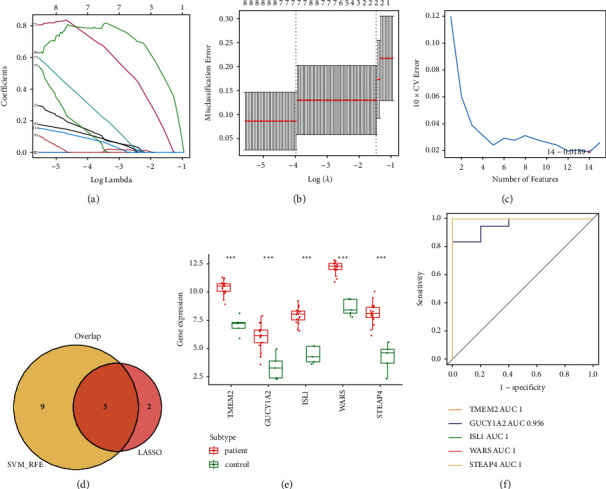
Identification of diagnostic markers. (a)-(b) LASSO logistic regression analysis result. (c) SVM-RFE algorithm of DEGs. (d) Venn diagram of lasso + SVM screening. (e) Differential expression of diagnostic genes in the GSE127487 dataset, the red bar charts represent IHs samples, and the green bar charts represent normal samples, ^*∗∗∗*^ means *P* < 0.001. (f) ROC curves of diagnostic genes.

A PPI network was constructed to explore the connections between the proteins encoded by the 205 DEGs. First, we dug up 127 interacting proteins and created a network with 127 nodes and 251 edges (Figure 4). In the following, we ran the LASSO logistic regression analysis based on these 127 genes and yielded the gene coefficients graph and the cross-validation graph, filtering out seven characteristic genes according to lambda close to 0 and the lowest error rate, namely PCDH17, TMEM2, GUCY1A2, ISL1, RUNX2, WARS, and STEAP4 (Figures 5(a) and 5(b)). Meanwhile, we conducted the SVM-RFE algorithm to rank the 127 genes for characteristics, and the top 20 genes were listed in Supplementary Table S6. As shown in Figure 5(c), 14 characteristic genes were identified using a 10-foldcross-validation approach at the optimal point “0.0189,” the top 14 genes in Supplementary Table S6. Subsequently, we obtained the characteristic genes for IHs by intersecting the distinct genes predicted by LASSO and SVM-RFE, namely TMEM2, GUCY1A2, ISL1, WARS, and STEAP4 (Figure 5(d)). After that, we proceeded with expression analysis and ROC curve analysis to further assess the diagnostic ability of the characteristic genes. As shown in Figure 5(e), the expression of all five distinct genes was increased in the infant hemangioma samples compared to the normal samples (all the *P*-value lower than 0.001). Furthermore, we found that except for GUCY1A2, which had an AUC value of 0.956, the other four diagnostic genes had an AUC value of 1, indicating the adequacy of five hub genes as diagnostic markers to accurately distinguish IHs samples from normal samples (Figure 5(f).

### 3.3. The Function of Diagnostic Genes in IHs

**Figure 6:**
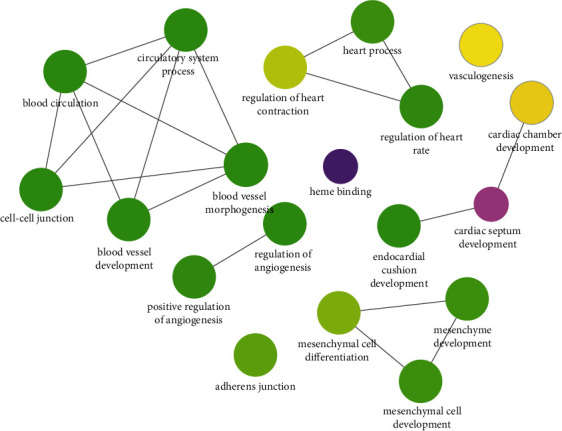
ClueGO function network of biomarkers. The point color from green-yellow-purple indicates that the *P* value decreases gradually, the line thickness indicates the number of overlapping genes between items, and the thicker the lines, the more overlapping genes.

**Figure 7:**
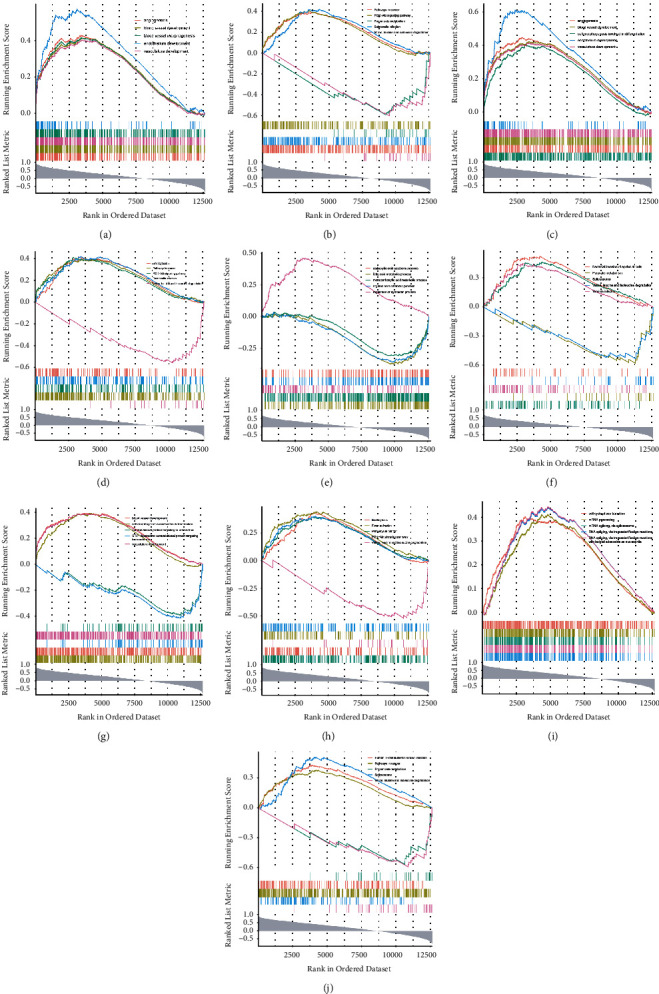
Functional annotation enrichment analysis of the identified diagnostic markers. ((a), (c), (e), (g), (i)) Top five gene set enrichment analysis (GSEA) plots of diagnostic markers by GO gene sets. ((b), (d), (f), (h), (j)) Top five GSEA plots of diagnostic genes by KEGG gene sets.

Based on the functional enrichment results of the DEGs, GO items relevant to diagnostic genes were picked and exhibited in Supplementary Table S7 and Figure 6. 19 biological processes were authenticated to be associated with diagnostic genes (Figure 6). WARS, a tryptophanyl-tRNA synthetase, was involved in “blood vessel development,” “vasculogenesis,” et al. TEME2, also known as CEMIP2, cell migration inducing hyaluronidase 2, was engaged in “cell-cell junction” and “adherens junction.” GUCY1A2, guanylate cyclase 1 soluble subunit alpha 2, and STEAP4, metalloreductase, were associated with “heme binding.” ISL1, ISL LIM homeobox 1, was correlated with multiple processes such as “blood vessel development,” “circulatory system process,” “heart process,” “blood circulation,” “regulation of angiogenesis,” “mesenchyme development,” and et al. To further gain insight into the biological role of each diagnostic gene in IHs, we performed the GSEA analysis for single-gene. TOP5 GO term and KEGG pathway for each gene are listed in Figure 7. All enrichment results and detailed information were shown in Supplementary Table S8. As to WARS, the top five biological processes, namely “angiogenesis,” “blood vessel development,” “vasculature development,” “blood vessel morphogenesis,” and “endothelium development,” were most positively related to WARS expression (Figure 7(a)). Among the top five KEGG pathways, “valine, leucine and isoleucine degradation” and “propanoate metabolism” were negatively correlated with WARS, suggesting that WARS may inhibit these pathways. Meanwhile, “pathways in cancer,” “salmonella infection,” and “PI3K-Akt signaling pathway” were positively correlated with WARS, indicating that WARS may activate these pathways (Figure 7(b)). The top five biological processes which were activated by TMEM2 were “angiogenesis,” “blood vessel development,” “vasculature development,” “blood vessel morphogenesis,” and “endothelium development” (Figure 7(c)). The top five KEGG pathways, “valine, leucine, and isoleucine degradation” was negatively correlated with TMEM2. “Pathways in cancer,” “PI3K-Akt signaling pathway,” “salmonella infection,” and “endocytosis” were positively correlated with TMEM2 (Figure 7(d)). For GUCY1A2, “regulation of symbiotic process” was activated most significantly. “Fatty acid metabolic process,” “monocarboxylic acid metabolic process,” “organic acid catabolic process,” and “carboxylic acid catabolic process” was inhibited by GUCY1A2 most significantly (Figure 7(e)). Among the top five KEGG pathways related to GUCY1A2, “pyruvate metabolism” and “valine, leucine, and isoleucine degradation” were inhibited. “Spliceosome,” “yersinia infection,” and “bacterial invasion of epithelial cells” were activated (Figure 7(f)). As to STEAP4, “cell morphogenesis involved in differentiation,” “blood vessel development” and “vasculature development” were positively relevant to STEAP4 expression most significantly. Moreover, STEAP4 expression was negatively related to “SRP-dependent cotranslational protein targeting to membrane” and “cotranslational protein targeting to membrane” (Figure 7(g)). Among the top five KEGG pathways related to STEAP4, “valine, leucine, and isoleucine degradation” was negatively related, and “pathways in cancer,” “endocytosis,” “focal adhesion,” and “PI3K-Akt signaling pathway” were positively related (Figure 7(h)). Concerning ISL1, “RNA splicing, via transesterification reactions,” “RNA splicing, via transesterification reactions with bulged adenosine as a nucleophile,” “mRNA splicing, via spliceosome,” “mRNA processing,” and “cell cycle phase transition” were activated most significantly by ISL1 (Figure 7(i)). Among the top five KEGG pathways related to ISL1, “propanoate metabolism” and “valine, leucine, and isoleucine degradation” were inhibited. “Spliceosome,” “pathways in cancer,” and “bacterial invasion of epithelial cells” were activated (Figure 7(j)). We noted that all five diagnostic genes inhibited the “valine, leucine, and isoleucine degradation” pathway in the development of IHs. WARS, TMEM2, STEAP4, and ISL1 activated the “pathways in cancer” pathway in IHs. WARS, TMEM2, and STEAP4 activated the “blood vessel development” and “vasculature development” in IHs.

### 3.4. Potential Inhibitors Related to Diagnostic Genes

**Figure 8:**
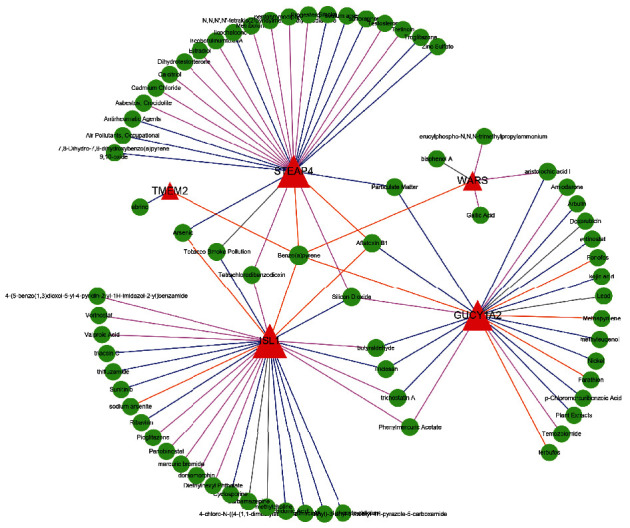
Targeted drug network of diagnostic markers. Red triangles are diagnostic markers, and green dots are drug molecules; the shape size of the node indicates connection degree, blue lines represent drugs inhibit biomarkers expression, pink lines represent drugs increasing biomarkers expression, yellow lines represent drugs' effect on gene methylation, gray lines show the effects of drugs on genes are unknown.

To further explore targeted drugs associated with diagnostic genes and provide more information for clinical treatment, we sought two compounds associated with TMEM2, five compounds associated with WARS, 24 compounds interacting with GUCY1A2, 30 compounds associated with ISL1, and 28 compounds correlated with STEAP4 by CTD, which are shown and listed in Figure 8 and Supplementary Table S9. In the interaction network, we detected that abrine inhibited the expression of TMEM2. The arbutin, aristolochic acid I, entinostat, trichostatin A, and triclosan inhibited GUCY1A2. STEAP4 was inhibited by antirheumatic agents, licochalcone B, sulforaphane, troglitazone, zinc zulfate, 7, 8-Dihydro-7, 8-dihydroxybenzo(a)pyrene 9, 10-oxide. The 4-chloro-N-((4-(1,1-dimethylethyl)phenyl)methyl)-3-ethyl-1-methyl-1H-pyrazole-5-carboxamide, 1-Methyl-4-phenylpyridinium, antimycin A, arachidonic acid, cyclosporine, ribavirin, sunitinib, thifluzamide, triacsin C, and triclosan were potential inhibitors for ISL1.

### 3.5. Transcription Factors Regulating the Diagnostic Genes

**Figure 9:**
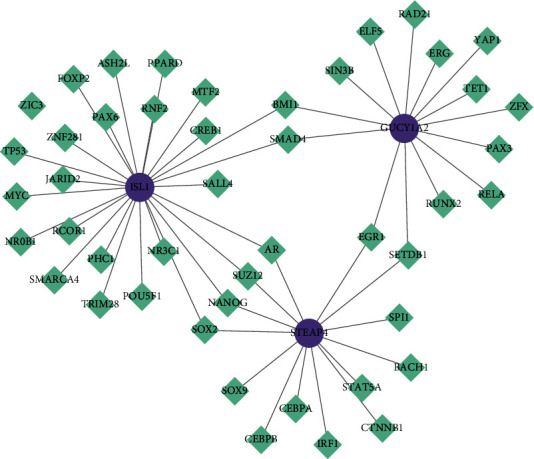
Transcription factor regulatory network of diagnostic markers. The purple dots are biomarkers, and the crinoline squares are transcription factors.

**Figure 10:**
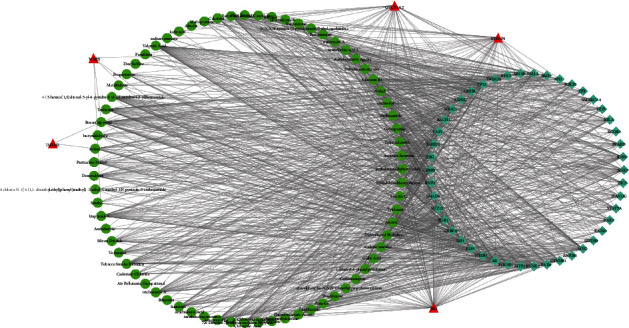
Potential compounds associated with the transcription factors of diagnostic markers. Red triangles are diagnostic markers, green dots are drugs, and green squares are transcription factors.

Via Network Analyst, we predicted 14 transcription factors regulating GUCY1A2, 14 transcription factors regulating STEAP4, and 26 transcription factors regulating ISL1, as revealed in the connection network (Figure 9). ISL1 and STEAP4 were both regulated by SOX2, NANOG, SUZ12, and AR in the network. ISL1 and GUCY1A2 were both regulated by BMI1 and SMAD4. ERG1 and SETDB1 regulated STEAP4 and GUCY1A2 simultaneously. Based on the potential drugs and the transcription factors of the target diagnostic marker identified, the linking of these drugs to the transcriptional factors was shown in the network diagram (Figure 10). Ulteriorly focusing on the relationship between diagnostic biomarkers-related drugs and corresponding major transcription factors (Supplementary Table S10), we found that GUCY1A2 inhibitors, aristolochic acid I, and entinostat could respectively inhibit the corresponding transcription factors SETDB1 and ERG1. The inhibitors of STEAP4, 7, 8-dihydro-7, 8-dihydroxybenzo(a)pyrene 9, 10-oxide simultaneously inhibited the expression of ERG1, SETDB1, and SUZ12, antirheumatic agents inhibited ERG1, sulforaphane and troglitazone inhibited AR. ISL1 inhibitors, cyclosporine inhibited AR, and sunitinib inhibited both AR and NANOG.

## 4. Discussion

IHs are characterized by abnormalities in the proliferation of vascular endothelial cells and vascular structure. Distinguished from congenital vascular malformations, IHs generally do not appear until a few weeks after birth [5], reporting a predominant prevalence (male-female ratio of 2.4 : 1) in females [24] and a higher incidence in premature infants and low birth weight (<2500 g). Current academic findings revealed a 40% increased risk for every 500 g birth weight loss [25], a history of chorionic villus sampling was accompanied by a two-fold raised risk of IHs [26], and siblings with IHs may elevate the incidence of subsequent siblings [27]. In addition, multiple pregnancies, Caucasians, preeclampsia, advanced maternal age, and placental abnormalities (such as placenta previa, placental abruption, and abnormal insertion of the umbilical cord) are also existing as risk factors for IHs occasion. According to guidelines published in multiple countries and territories, pediatricians and primary care clinicians are supposed to regularly monitor IHs in infants during the first months after birth and refer infants requiring intervention to specialists as early as possible [3]. Hence, early diagnosis is of great benefit for the subsequent treatment of IHs.

In this study, bioinformatics analyses were adopted for potential biomarkers identification in the development of IHs, providing a particular reference for the diagnosis and treatment of IHs. We firstly obtained 205 candidate genes by differential gene expression analysis and intersection. The results of GO enrichment analysis revealed that vascular development was the primary function of the candidate genes, which was primarily associated with cardiac development, vascular maturation, and the blood circulation system. Ulteriorly, we used the STRING tool to construct a PPI network, Lasso analysis, and SVM regression analysis, subtracting above 205 candidate genes, and finally acquiring five hub genes, i.e., TMEM2, GUCY1A2, ISL1, WARS, and STEAP4.

Acting as a transmembrane protein, TMEM2 is associated with tumor invasion and angiogenesis [28, 29] and manipulates cell adhesion and metastasis by degrading non-protein components of the extracellular matrix [30]. Gastric cancer prognosis is positively related to GUCY1A2 [31], which plays a vital role in rheumatoid arthritis development [32], and affects SARS-CoV 2 infections and drug repurposing [33]. ISL1 is identified as a biomarker of oral squamous cell carcinoma [34], up-regulator of vascular development [35], and activator of EMT to induce drug resistance in prostate cancer [36], serving to regulate reproductive system development [37]. WARS, an essential enzyme catalyzing the ligation of tryptophan to its cognate tRNA tryptophan during translation via aminoacylation, promoting cancer metastasis by controlling angiogenesis and immune response in the tumor microenvironment, which plays a pathological role in autoimmune disease and Alzheimer's disease concurrently. In addition, high expression of WARS could be referred to as a prognostic biomarker for sepsis [38]. STEAP4 is a metal reductase that reduces Fe^3+^ to Fe^2+^ and Cu^2+^ to Cu^+^, promoting iron and copper transmembrane transport and maintaining their homeostasis, as well as playing a beneficial role against damage from inflammatory diseases and metabolic disorders. Nevertheless, its abnormal expression may accelerate cancer proliferation or progression [39]. Furthermore, the expressions were significantly higher for five hub genes in IHs samples than in normal samples. And we finally defined these five genes as diagnostic markers for IHs by the ROC analysis.

Similar functional effects of five diagnostic markers were obtained through further biofunctional analyses. It is well known that the dramatic and disorganized growth of blood vessels is a defining feature of IHs, which represents a vascular perturbation comprising pathologic angiogenesis and vasculogenesis during post-natal growth [40]. Among the top five with highly significant enrichment, TMEM2, WARS, and STEAP4 were related to angiogenesis and vascular development. Valine, leucine, and isoleucine are collectively referred to as Branched-chain amino acids (BCAAs), which are essential for the growth of human tumors by donating nitrogen to generate macromolecules such as nucleotides. Cellular autonomous and involuntary effects of BCAA metabolic transformations play a role in cancer progression, indicating that the crucial proteins in BCAA metabolic pathway can be prognostic or diagnostic biomarkers for human cancer [41]. Consistently, all five biomarkers were involved in the degradation of valine, leucine, and isoleucine. In addition, several drugs were recognized to predict the diagnostic makers' inhibitor using the CTD database. Thereinto, most of these drugs are involved in angiogenesis inhibition, e.g., arbutin [42], entinostat [43], sulforaphane [44], and sunitinib [45], which have been demonstrated to suppress angiogenesis in tumor treatments effectively. Triclosan [46] and ribavirin [47] had been identified as effectual angiogenesis inhibitors. Cyclosporine promoted osteogenic differentiation of periodontal membrane stem cells and curbed angiogenesis [48]. Troglitazone can subdue angiogenesis by inhibiting endothelial cells (EC) proliferation and vascular endothelial growth factor (VEGF) expression [49]. Triacsin C inhibits angiogenesis manipulated by fatty acid metabolism [50]. The results were expected to make new progress in the treatment of IHs.

The function of hub genes is influenced by their expression level and regulated by transcription factors [51]. Therefore, we predicted the transcription factors regulating these screened biomarkers through the online website “Network Analyst,” reporting that only three genes can obtain new work analysis results, indicating that different transcription factors regulated each gene. The co-transcription factors involved in the regulation of 2 hub genes simultaneously involved SOX2, NANOG, SUZ12, AR, BMI1, SMAD4, ERG1, and SETDB1. of IHs are cellularly composed of EC, pericytes, and stem cells [52]. EC works as the main driving force of angiogenesis, which responds to VEGF stimulation, critical signal transduction for angiogenesis [53], and rapidly transforms from a static state to an active form in the proliferation of IHs [54]. Notably, most co-transcription factors regulate the VEGF pathway, affecting the angiogenesis of various tumors or organs. For example, high expression of SOX2, AR, and BMI1 stimulates angiogenesis in multiple tumors [55–58]. Over-expressed NANOG can promote EC proliferation and angiogenesis by inducing transcription of liver kinase-1 (FLK1) [59]. On the contrary, a low expression level of NANOG is required for the maintenance of EC homeostasis and angiogenesis [60]. EC development is also intervened by SMAD4 [61]; loss of SMAD4 leads to decreased expression of VEGFR2 and regulates angiogenesis [62].

It is interesting to note that there are some connections in potential drugs and transcription factors of target diagnostic markers, which were both involved in the regulation of angiogenesis. In addition to this study's results, recent literature also shows that aristolochic acid [63], and entinostat [64] induce the down-regulation of SMAD4 in human cells. Sulforaphane weakens the NANOG and SOX2 in respiratory tumors [65]. Sunitinib therapy can regulate SMAD4 expression by altering the expression of Mir-452-5p in renal cancer [66]. Overall, regulation of transcription factors may play a role in drug inhibition of diagnostic marker genes.

This study also has limitations, mainly reflected in a small sample size as source databases are derived. And the molecules as the diagnostic markers of IHs are only based on bioinformatic analysis data. On this account, we plan to collect as sufficient samples as possible to verify the clinical diagnosis value of Hub genes for IHs. More specifically, the discriminant capacity and potential action mechanism of hub genes on IHs development were further clarified by Single-cell sequencing. The feasibility of drug inhibitors and related transcription factors obtained in this study will be the primary objects discussed in the following survey on IHs therapy.

## 5. Conclusions

Bioinformatics methods were utilized to identify five hub genes (including TMEM2, GUCY1A2, ISL1, WARS, and STEAP4) with power diagnostic capacity for IHs in this study. These diagnostic markers may regulate IHs development by participating in tumorigenesis-related biological processes, such as angiogenesis and BCAAs metabolism. We also determined inhibitors and transcription factors associated with these diagnostic markers. Despite as a preliminary study that requires further research to validate these results, the analysis outcomes provide new insights into the diagnosis and treatment of IHs.

## Data Availability

The data used to support the findings of this study are available from the corresponding author upon request.

## Supplementary Materials

Supplementary Materials Table S1: DEGs of IHs in the 6-month-old compared to normal samples. Table S2: DEGs of IHs in the 12-month-old compared to normal samples. Table S3: DEGs of IHs in the 24-month-old compared to normal samples. Table S4: common up- and down-regulated genes among the 6-, 12-, and 24-month-old IHs samples. Table S5: GO and KEGG analysis of candidate genes. Table S6: the top 20 significant genes listed by the SVM-RFE algorithm ranked in 127 candidate genes for characteristics. Table S7: GO items relevant to diagnostic genes. Table S8: all functional annotation enrichment analysis results of the identified diagnostic genes. Table S9: all potential compounds are associated with the identified diagnostic genes. Table S10: potential compounds are associated with the major transcription factors.
