# Supplementary material for: Identification of Diagnostic Markers in Infantile Hemangiomas
Source: J Oncol. 2022 Dec 1;2022:9395876. doi: 10.1155/2022/9395876 (PMC9731762; doi:10.1155/2022/9395876)
Supplement: Supplementary Materials — Table S1: DEGs of IHs in the 6-month-old compared to normal samples. Table S2: DEGs of IHs in the 12-month-old compared to normal samples. Table S3: DEGs of IHs in the 24-month-old compared to normal samples. Table S4: common up- and down-regulated genes among the 6-, 12-, and 24-month-old IHs samples. Table S5: GO and KEGG analysis of candidate genes. Table S6: the top 20 significant genes listed by the SVM-RFE algorithm ranked in 127 candidate genes for characteristics. Table S7: GO items relevant to diagnostic genes. Table S8: all functional annotation enrichment analysis results of the identified diagnostic genes. Table S9: all potential compounds are associated with the identified diagnostic genes. Table S10: potential compounds are associated with the major transcription factors. [file 9395876.f1.zip › Supplementary Table S1.pdf]

## Table S1. DEGs of IHs in the 6-month-old compared to normal samples

| Symbol | logFC | AveExpr | t | P.Value | adj.P.Val | B |
|---|---|---|---|---|---|---|
| LINGO1 | 3.036174633 | 7.705386433 | 12.92742706 | 1.76E-08 | 0.000148155 | 9.565090766 |
| PCDH17 | 5.656653028 | 9.358767514 | 12.60598888 | 2.35E-08 | 0.000148155 | 9.327149901 |
| ARID3A | 3.056196385 | 8.642642248 | 11.88008938 | 4.60E-08 | 0.000193473 | 8.760343107 |
| CETP | 4.024194896 | 7.124350164 | 11.36128907 | 7.61E-08 | 0.000239381 | 8.328541787 |
| HS3ST3A1 | 4.234738577 | 7.784636798 | 11.03688567 | 1.05E-07 | 0.000239381 | 8.046467772 |
| PCSK5 | 3.385876863 | 9.261269608 | 10.95832629 | 1.14E-07 | 0.000239381 | 7.976707668 |
| MDK | 3.304700302 | 9.985070739 | 10.62187697 | 1.61E-07 | 0.000263565 | 7.671340321 |
| FAM184A | 3.573016693 | 7.605727907 | 10.58616867 | 1.67E-07 | 0.000263565 | 7.638290444 |
| WARS | 3.492174262 | 10.52259362 | 10.27882779 | 2.32E-07 | 0.000297515 | 7.348603468 |
| LXN | 3.128521393 | 9.707490993 | 10.26128026 | 2.36E-07 | 0.000297515 | 7.331776945 |
| HECW2 | 3.409115711 | 7.7778908 | 9.980481575 | 3.20E-07 | 0.000347897 | 7.058191643 |
| IGF2 | 6.100215312 | 7.899354656 | 9.949535348 | 3.31E-07 | 0.000347897 | 7.027535527 |
| TIE1 | 3.47023971 | 7.967951282 | 9.680874635 | 4.46E-07 | 0.000410789 | 6.757078292 |
| HEY1 | 3.11134961 | 7.578725327 | 9.601491834 | 4.88E-07 | 0.000410789 | 6.675660072 |
| APLN | 6.13018158 | 7.810218928 | 9.563756218 | 5.10E-07 | 0.000410789 | 6.636712361 |
| PVRL2 | 2.658956842 | 8.12300554 | 9.501788737 | 5.47E-07 | 0.000410789 | 6.572410062 |
| FAM69B | 3.06520463 | 9.798084539 | 9.49054093 | 5.54E-07 | 0.000410789 | 6.560692347 |
| MAP4K2 | 2.369966085 | 10.1692434 | 9.16747166 | 8.05E-07 | 0.000551767 | 6.217979014 |
| ICAM2 | 3.681269312 | 10.07455913 | 9.13991458 | 8.32E-07 | 0.000551767 | 6.18818827 |
| CRMP1 | 5.072559057 | 6.59186608 | 8.85195095 | 1.17E-06 | 0.000706727 | 5.871498528 |
| MGC16121 | 5.97995742 | 8.049128594 | 8.847872299 | 1.18E-06 | 0.000706727 | 5.866941502 |
| PDGFB | 3.59802964 | 7.503337183 | 8.632674348 | 1.53E-06 | 0.000876407 | 5.623613265 |
| MARCKSL1 | 2.098688668 | 10.10898253 | 8.563215165 | 1.67E-06 | 0.000913135 | 5.543849668 |

| | | | | | | |
|---|---|---|---|---|---|---|
| FKBP1A | 2.824408408 | 9.178062868 | 8.403614167 | 2.03E-06 | 0.001067148 | 5.35826746 |
| RASGRP3 | 2.96735366 | 8.467342248 | 8.33597521 | 2.21E-06 | 0.001115262 | 5.27863788 |
| WSCD1 | 3.20240737 | 6.593148671 | 8.225489014 | 2.54E-06 | 0.001151549 | 5.147294707 |
| TMEM2 | 3.199381299 | 8.809949258 | 8.221124124 | 2.56E-06 | 0.001151549 | 5.142073249 |
| CLEC11A | 2.304846534 | 7.877424952 | 8.166278023 | 2.74E-06 | 0.001192369 | 5.076251671 |
| ANKRD20A1 | 3.845687514 | 8.345704934 | 8.095254094 | 3.00E-06 | 0.001212345 | 4.99042722 |
| MAGED2 | 2.321284429 | 7.478434531 | 8.087309988 | 3.04E-06 | 0.001212345 | 4.980786222 |
| ANKRD47 | 2.428422985 | 7.527966873 | 8.076605167 | 3.08E-06 | 0.001212345 | 4.967781578 |
| C5orf13 | 2.775698932 | 10.39086247 | 8.014454176 | 3.33E-06 | 0.001273871 | 4.891977116 |
| TRIB3 | 2.479907792 | 7.597733769 | 7.967078258 | 3.55E-06 | 0.001282655 | 4.83384717 |
| ABHD7 | 2.095766295 | 6.080551703 | 7.963830538 | 3.56E-06 | 0.001282655 | 4.829851219 |
| MEG3 | 3.897806969 | 10.74214288 | 7.919455585 | 3.77E-06 | 0.001321285 | 4.775110763 |
| GPX7 | 2.591134734 | 8.864376166 | 7.77528863 | 4.56E-06 | 0.001495774 | 4.595427682 |
| AFAP1L1 | 2.670158701 | 7.875249532 | 7.737337228 | 4.80E-06 | 0.001511397 | 4.547655158 |
| GAP43 | 4.838347008 | 6.169089996 | 7.659316874 | 5.32E-06 | 0.001636014 | 4.448821652 |
| HLX | 3.127612772 | 6.929157995 | 7.58927989 | 5.84E-06 | 0.001754302 | 4.359382858 |
| BCORL1 | 2.134775185 | 6.525853448 | 7.406194961 | 7.49E-06 | 0.002146484 | 4.122334045 |
| NDUFA4L2 | 4.927326548 | 9.536158772 | 7.339812351 | 8.21E-06 | 0.002298812 | 4.035213849 |
| EXOC6 | 2.700636257 | 8.424015743 | 7.298429981 | 8.69E-06 | 0.002315074 | 3.980585548 |
| ACVRL1 | 3.141382868 | 9.441399598 | 7.283726116 | 8.87E-06 | 0.002315074 | 3.961116102 |
| MRI1 | 2.217165226 | 8.556775942 | 7.279858954 | 8.91E-06 | 0.002315074 | 3.955990424 |
| COX4I2 | 6.242434137 | 7.304278115 | 7.272976475 | 9.00E-06 | 0.002315074 | 3.946862822 |
| ISL1 | 3.454753374 | 6.308916345 | 7.236882735 | 9.46E-06 | 0.002380517 | 3.898883444 |
| LOC442041 | 2.078829933 | 5.78102304 | 7.224029015 | 9.63E-06 | 0.002380517 | 3.881751688 |
| C21orf63 | -3.445769608 | 6.121776996 | -7.184141901 | 1.02E-05 | 0.002448237 | 3.828437246 |
| GRAP | 2.418916518 | 8.472748981 | 7.176274281 | 1.03E-05 | 0.002448237 | 3.817893947 |

| | | | | | | |
|---|---|---|---|---|---|---|
| KCNJ2 | 2.804849248 | 7.725465741 | 7.145301345 | 1.07E-05 | 0.002509219 | 3.776300287 |
| COL18A1 | 3.220815889 | 11.23771589 | 7.067105425 | 1.20E-05 | 0.002749639 | 3.670669871 |
| TNFRSF10A | 2.959779045 | 6.016063262 | 7.03026159 | 1.26E-05 | 0.002797989 | 3.620590154 |
| ENPEP | 3.935439592 | 8.44862107 | 7.026068142 | 1.27E-05 | 0.002797989 | 3.614877637 |
| C20orf46 | 4.072497162 | 6.892455982 | 7.017180194 | 1.29E-05 | 0.002797989 | 3.602761514 |
| FAM101B | 3.705243613 | 6.352902887 | 6.990466523 | 1.34E-05 | 0.002856744 | 3.566275403 |
| RAMP2 | 2.338949632 | 8.011870111 | 6.926640952 | 1.46E-05 | 0.003076345 | 3.478675737 |
| PXDN | 3.135712279 | 9.877541394 | 6.901420955 | 1.52E-05 | 0.003136989 | 3.443895897 |
| FAM38B | 2.687534953 | 7.011164399 | 6.857622466 | 1.62E-05 | 0.003236192 | 3.383271397 |
| FHL3 | 2.402889097 | 7.935888426 | 6.849744509 | 1.63E-05 | 0.003236192 | 3.372336774 |
| DNAJC12 | 2.866850376 | 5.195808579 | 6.846255741 | 1.64E-05 | 0.003236192 | 3.367491411 |
| HOXB5 | 2.661017895 | 7.322842605 | 6.790851703 | 1.78E-05 | 0.003348369 | 3.290300975 |
| ZNF697 | 2.493033516 | 5.956406797 | 6.719128984 | 1.97E-05 | 0.003570195 | 3.189694659 |
| DYSF | 3.657150764 | 9.050265282 | 6.717897574 | 1.98E-05 | 0.003570195 | 3.187960625 |
| SEMA5B | 4.227880331 | 6.401802728 | 6.716423974 | 1.98E-05 | 0.003570195 | 3.185885247 |
| PGF | 3.052988349 | 6.820779331 | 6.623116843 | 2.27E-05 | 0.003809726 | 3.053810143 |
| EGFLAM | 2.324723068 | 7.95393601 | 6.620346024 | 2.28E-05 | 0.003809726 | 3.04986806 |
| PTP4A3 | 2.056937255 | 6.062515406 | 6.610126723 | 2.32E-05 | 0.003809726 | 3.035318909 |
| FXYD6 | 2.249086106 | 8.568199737 | 6.606857818 | 2.33E-05 | 0.003809726 | 3.030661668 |
| TUSC3 | 3.377108445 | 8.329432359 | 6.538667484 | 2.57E-05 | 0.004004641 | 2.93314231 |
| TYMS | 2.821946819 | 8.184688751 | 6.512179492 | 2.68E-05 | 0.004042337 | 2.895072031 |
| BCL6B | 2.619134126 | 8.371425413 | 6.482634222 | 2.80E-05 | 0.004042337 | 2.852482169 |
| RAPGEF5 | 2.250526549 | 9.377722116 | 6.472492005 | 2.84E-05 | 0.004042337 | 2.837831506 |
| TFPI2 | 8.071249683 | 6.567108458 | 6.461365459 | 2.89E-05 | 0.004042337 | 2.821740989 |
| EPS15L1 | 2.59756767 | 5.091318005 | 6.407247443 | 3.13E-05 | 0.004272174 | 2.743210519 |
| SCN4B | 3.530803467 | 8.678804193 | 6.402378396 | 3.15E-05 | 0.004272174 | 2.73612321 |

| | | | | | | |
|---|---|---|---|---|---|---|
| STEAP4 | 4.048272784 | 6.423565091 | 6.3840291 | 3.24E-05 | 0.004344567 | 2.709381811 |
| PAPSS2 | 2.983410337 | 8.598262355 | 6.359902953 | 3.36E-05 | 0.004400544 | 2.674143406 |
| PHACTR2 | 3.041865447 | 9.212431049 | 6.352645671 | 3.40E-05 | 0.004400544 | 2.663526125 |
| COL4A2 | 4.03099642 | 10.70847176 | 6.337762316 | 3.47E-05 | 0.004400544 | 2.641726885 |
| TPM4 | 2.525280999 | 8.935435555 | 6.325405974 | 3.54E-05 | 0.004400544 | 2.6236032 |
| RGS5 | 3.836739564 | 10.40482873 | 6.323439728 | 3.55E-05 | 0.004400544 | 2.620717056 |
| TDO2 | 2.082047327 | 6.105926025 | 6.321215402 | 3.56E-05 | 0.004400544 | 2.617451377 |
| TXNDC5 | 2.306198201 | 9.429673003 | 6.292986888 | 3.72E-05 | 0.004535904 | 2.575941577 |
| RBM9 | 2.152572807 | 9.489228702 | 6.28830639 | 3.74E-05 | 0.004535904 | 2.569047171 |
| NR2F1 | 2.224546613 | 7.198667245 | 6.251286171 | 3.96E-05 | 0.004733588 | 2.514398061 |
| Septin 4 | 2.34261233 | 7.687284731 | 6.226578943 | 4.11E-05 | 0.004774164 | 2.477808625 |
| C1orf54 | 2.978246269 | 9.991145345 | 6.208366618 | 4.22E-05 | 0.004774164 | 2.450777758 |
| NOX4 | 3.92214815 | 7.570696668 | 6.208061463 | 4.23E-05 | 0.004774164 | 2.450324412 |
| FAM43A | 2.546852167 | 9.337779926 | 6.20507122 | 4.25E-05 | 0.004774164 | 2.445881275 |
| EVI1 | 2.33678242 | 8.711624944 | 6.202627955 | 4.26E-05 | 0.004774164 | 2.442249864 |
| GUCY1A2 | 3.310782202 | 5.156328957 | 6.199962323 | 4.28E-05 | 0.004774164 | 2.438286904 |
| APLNR | 4.276600057 | 10.77167217 | 6.193061791 | 4.32E-05 | 0.004777301 | 2.428022919 |
| ZNF641 | 3.422602637 | 6.173141139 | 6.18803373 | 4.36E-05 | 0.004777301 | 2.420539487 |
| PDPN | -2.764625207 | 5.42191101 | -6.156837554 | 4.57E-05 | 0.004948529 | 2.374022521 |
| BCAR1 | 2.317422463 | 7.305775753 | 6.149198244 | 4.62E-05 | 0.004948529 | 2.362608707 |
| MTUS1 | 3.005645497 | 7.670606708 | 6.117182533 | 4.86E-05 | 0.005145883 | 2.314676957 |
| LOC100133999 | 2.465391556 | 7.157132922 | 6.107345102 | 4.93E-05 | 0.005180752 | 2.299917424 |
| FAM162B | 3.068975938 | 9.435988039 | 6.070975093 | 5.22E-05 | 0.00535009 | 2.245220866 |
| STARD8 | 3.157348811 | 7.791748955 | 6.060708513 | 5.30E-05 | 0.00535009 | 2.229744272 |
| LOC644162 | 2.068681953 | 9.452342924 | 6.052189181 | 5.37E-05 | 0.005371944 | 2.216889316 |
| LOC732360 | 2.150833801 | 7.088992436 | 6.041939879 | 5.45E-05 | 0.005379402 | 2.201409205 |

| | | | | | | |
|---|---|---|---|---|---|---|
| MYLIP | 2.487513314 | 10.78804722 | 6.041106881 | 5.46E-05 | 0.005379402 | 2.200150372 |
| SH2D3C | 3.776639012 | 8.530573429 | 5.970243929 | 6.10E-05 | 0.00584122 | 2.092671334 |
| B3GAT3 | 2.138395039 | 4.86941305 | 5.968213607 | 6.12E-05 | 0.00584122 | 2.089580541 |
| IKBIP | 2.272725834 | 5.715640197 | 5.944436974 | 6.35E-05 | 0.005883485 | 2.053337819 |
| LOC100132707 | 2.447124894 | 5.751592933 | 5.909471511 | 6.70E-05 | 0.006168588 | 1.999882177 |
| EBF2 | 2.462900623 | 6.234104418 | 5.904116466 | 6.76E-05 | 0.006175449 | 1.991678731 |
| MYO1B | 2.54823656 | 8.923673448 | 5.888806299 | 6.92E-05 | 0.006279903 | 1.968200639 |
| HYAL2 | 2.419522405 | 9.006200673 | 5.864700347 | 7.19E-05 | 0.006375677 | 1.931161279 |
| COL15A1 | 2.549880581 | 10.88198471 | 5.861534808 | 7.23E-05 | 0.006375677 | 1.926290726 |
| NID1 | 3.225296204 | 7.220243818 | 5.860802914 | 7.24E-05 | 0.006375677 | 1.925164403 |
| DOCK6 | 2.142423316 | 8.098495984 | 5.856665731 | 7.28E-05 | 0.006375677 | 1.918796079 |
| LOC100130623 | 2.545474458 | 6.486788625 | 5.844532905 | 7.42E-05 | 0.006425669 | 1.900104988 |
| COLEC11 | 2.32495367 | 6.886124268 | 5.842937321 | 7.44E-05 | 0.006425669 | 1.897645249 |
| H19 | 4.693535494 | 11.95025791 | 5.834446551 | 7.54E-05 | 0.006467888 | 1.884549371 |
| CDH5 | 3.673731307 | 10.97251493 | 5.81179607 | 7.82E-05 | 0.006468276 | 1.849559914 |
| IL32 | 2.435774979 | 7.445191769 | 5.807072196 | 7.87E-05 | 0.006468276 | 1.842252766 |
| PECAM1 | 3.380972999 | 11.42325971 | 5.806706554 | 7.88E-05 | 0.006468276 | 1.841687028 |
| STX3 | 2.140996513 | 8.070047044 | 5.804953631 | 7.90E-05 | 0.006468276 | 1.83897454 |
| CD34 | 2.393904313 | 9.664974377 | 5.804128017 | 7.91E-05 | 0.006468276 | 1.837696815 |
| CDKN1C | 2.467202215 | 8.831814782 | 5.800829893 | 7.95E-05 | 0.006468276 | 1.832591578 |
| C4orf32 | 2.187297856 | 8.795595894 | 5.774914456 | 8.29E-05 | 0.006644064 | 1.792418472 |
| PNPLA7 | -2.130624059 | 7.802079699 | -5.773646167 | 8.30E-05 | 0.006644064 | 1.790449777 |
| ADA | 2.330087978 | 7.368950841 | 5.767534307 | 8.38E-05 | 0.006644064 | 1.780959219 |
| FAM176B | 2.051382733 | 7.365232964 | 5.763837547 | 8.43E-05 | 0.006644064 | 1.775216075 |
| RUVBL1 | 2.352961793 | 6.96034466 | 5.752786156 | 8.58E-05 | 0.006719582 | 1.758034576 |
| ITM2C | 2.008761834 | 10.52453817 | 5.719370709 | 9.05E-05 | 0.006872718 | 1.705970126 |

| | | | | | | |
|---|---|---|---|---|---|---|
| GCOM1 | 2.797638161 | 7.427810505 | 5.704308569 | 9.27E-05 | 0.006997463 | 1.682446014 |
| BST2 | 3.182765332 | 9.840726709 | 5.676862826 | 9.68E-05 | 0.007181771 | 1.639491965 |
| SYN2 | 2.907098188 | 5.887563587 | 5.627224446 | 0.000104855 | 0.007510871 | 1.561513156 |
| KHDRBS3 | 2.250197566 | 8.050859115 | 5.544746216 | 0.000119753 | 0.008316927 | 1.431115244 |
| TMEM108 | 6.030687709 | 5.033421569 | 5.543127051 | 0.000120067 | 0.008316927 | 1.428545004 |
| LOC644242 | 2.976159934 | 5.006134115 | 5.521512331 | 0.000124341 | 0.008539208 | 1.394196038 |
| ROBO4 | 2.524642736 | 7.39777926 | 5.516978407 | 0.000125257 | 0.008539208 | 1.386981986 |
| KCNJ8 | 3.163960739 | 9.251255867 | 5.516730534 | 0.000125308 | 0.008539208 | 1.386587499 |
| PPP1R12B | 2.089795215 | 5.514312589 | 5.495023253 | 0.000129798 | 0.008599755 | 1.352004528 |
| FGD1 | 2.587483306 | 6.826967121 | 5.493673874 | 0.000130083 | 0.008599755 | 1.349852412 |
| FUT11 | 2.382080936 | 5.326716685 | 5.475903458 | 0.000133894 | 0.008746103 | 1.321484815 |
| CX3CL1 | -2.661457049 | 7.828901094 | -5.47100833 | 0.000134964 | 0.008766636 | 1.313662157 |
| GPR176 | 2.859313883 | 6.191745799 | 5.467946709 | 0.000135638 | 0.008766636 | 1.308767698 |
| TTYH2 | 2.267838372 | 5.265778607 | 5.448814741 | 0.000139931 | 0.008886057 | 1.278150372 |
| PDLIM7 | 2.259023032 | 5.369866007 | 5.447349981 | 0.000140265 | 0.008886057 | 1.27580401 |
| CHPT1 | -2.009133162 | 7.565827197 | -5.440209803 | 0.000141908 | 0.008945174 | 1.264361714 |
| GBP4 | 2.777741984 | 8.421841064 | 5.436071689 | 0.000142869 | 0.008960964 | 1.25772678 |
| GNG11 | 2.739323827 | 11.9359377 | 5.396795479 | 0.000152342 | 0.009323183 | 1.194624295 |
| CBX5 | 2.169847866 | 7.963304737 | 5.391813696 | 0.00015359 | 0.009341631 | 1.186603849 |
| KLHL23 | 4.775030092 | 4.817134348 | 5.381859662 | 0.000156116 | 0.009408117 | 1.170567166 |
| PMEPA1 | 2.608534893 | 9.310972717 | 5.333790751 | 0.000168943 | 0.009874178 | 1.092916092 |
| GVIN1 | 2.266939444 | 6.769196111 | 5.271176091 | 0.000187337 | 0.010768979 | 0.99125171 |
| SIPA1L2 | 2.12554713 | 9.531581157 | 5.257305082 | 0.000191689 | 0.010885695 | 0.968651416 |
| GJA4 | 2.892814335 | 9.032792245 | 5.25156701 | 0.000193521 | 0.010898126 | 0.959293958 |
| LOC652330 | 2.624691912 | 5.541945668 | 5.251204527 | 0.000193637 | 0.010898126 | 0.958702669 |
| MFNG | 2.971029321 | 8.812805521 | 5.233135798 | 0.000199529 | 0.011036606 | 0.929204102 |

| | | | | | | |
|---|---|---|---|---|---|---|
| MPDZ | 2.074932437 | 8.870920552 | 5.218158325 | 0.000204556 | 0.011163777 | 0.904715812 |
| KCTD7 | 3.188453247 | 3.831243892 | 5.209874963 | 0.000207393 | 0.011269823 | 0.891158291 |
| TINAGL1 | 3.2368906 | 7.006000737 | 5.199345846 | 0.000211059 | 0.011274642 | 0.873910574 |
| RTN2 | 2.091460634 | 6.334254814 | 5.192274306 | 0.000213559 | 0.01136007 | 0.862317586 |
| PRSS35 | 2.96304968 | 7.173542027 | 5.175940027 | 0.000219454 | 0.011575958 | 0.835511418 |
| AGRN | 2.045699549 | 9.258589497 | 5.159076891 | 0.000225719 | 0.011807639 | 0.807796473 |
| TCF4 | 2.230439145 | 10.01231227 | 5.144830721 | 0.000231158 | 0.011986857 | 0.784350291 |
| SEL1L3 | 2.29374202 | 8.42544617 | 5.128037254 | 0.000237748 | 0.012184076 | 0.756673925 |
| C6orf188 | 2.805053992 | 5.452876107 | 5.120400213 | 0.000240809 | 0.012241442 | 0.744074219 |
| GUCY1B3 | 2.512623764 | 6.841312312 | 5.114391121 | 0.000243247 | 0.012294972 | 0.734154393 |
| FJX1 | 2.729414642 | 8.035231483 | 5.113005858 | 0.000243812 | 0.012294972 | 0.731866855 |
| NEBL | -3.399111069 | 5.57338569 | -5.109576476 | 0.000245219 | 0.0123002 | 0.726202594 |
| PLAC8 | 4.487297432 | 5.214841965 | 5.108001537 | 0.000245867 | 0.0123002 | 0.723600722 |
| ATP1B2 | 2.156242817 | 6.222740449 | 5.099819122 | 0.000249267 | 0.012323551 | 0.710077219 |
| LBR | 2.564349715 | 6.330217788 | 5.088219506 | 0.00025417 | 0.012468195 | 0.690889382 |
| CNTNAP1 | 2.142901804 | 8.009650442 | 5.057505404 | 0.00026765 | 0.012921015 | 0.639989318 |
| CLYBL | -2.069673435 | 6.090752969 | -5.046317638 | 0.000272744 | 0.012975404 | 0.62141511 |
| NTN1 | -3.025084875 | 4.692620178 | -5.04009392 | 0.000275622 | 0.012984634 | 0.611074594 |
| C9orf61 | -2.334286221 | 5.969522057 | -5.005780112 | 0.00029207 | 0.013487662 | 0.553964162 |
| UGCGL1 | 2.335444357 | 7.022187339 | 4.99289308 | 0.000298511 | 0.013668873 | 0.532472358 |
| IGFALS | 2.575689962 | 4.563211368 | 4.991438415 | 0.000299247 | 0.013668873 | 0.530044925 |
| SPTBN1 | 2.013932057 | 9.401321695 | 4.98080873 | 0.000304685 | 0.013867038 | 0.512297854 |
| JAM3 | 3.042059148 | 10.8976057 | 4.973995483 | 0.000308226 | 0.013961717 | 0.500914235 |
| SERPINH1 | 2.858006715 | 10.4066733 | 4.961065636 | 0.000315063 | 0.014073611 | 0.479292987 |
| JAM2 | 2.699300691 | 9.589829901 | 4.958714613 | 0.000316323 | 0.014073611 | 0.475359089 |
| RASIP1 | 2.087566938 | 10.03199957 | 4.940091827 | 0.000326494 | 0.014118542 | 0.444170742 |

| | | | | | | |
|---|---|---|---|---|---|---|
| TSPAN18 | 2.999511698 | 9.525844175 | 4.939162611 | 0.00032701 | 0.014118542 | 0.442613273 |
| TFPI | 2.118779212 | 9.585050283 | 4.898998134 | 0.000350167 | 0.014644564 | 0.375178182 |
| STXBP6 | 3.011147751 | 7.788990929 | 4.893558063 | 0.000353433 | 0.014644564 | 0.366027223 |
| PABPC4L | 2.12383277 | 7.131864832 | 4.892131035 | 0.000354295 | 0.014644564 | 0.363626084 |
| VAMP5 | 2.206173318 | 9.855142772 | 4.886725422 | 0.00035758 | 0.014684069 | 0.354527966 |
| SCARF2 | 2.059008733 | 7.24588869 | 4.873487983 | 0.00036576 | 0.014894253 | 0.332231177 |
| MLPH | -3.479967638 | 6.938842413 | -4.867318104 | 0.00036964 | 0.01493185 | 0.321830553 |
| PMP2 | -2.477857998 | 5.196690597 | -4.86082385 | 0.00037377 | 0.015006734 | 0.310877478 |
| RSPO3 | 3.40291655 | 7.901368024 | 4.847201673 | 0.00038259 | 0.015215503 | 0.287883808 |
| SERPINE2 | 2.323180861 | 10.66481223 | 4.839330946 | 0.000387786 | 0.015325431 | 0.274586781 |
| RPL23AP13 | 2.126101363 | 7.693828906 | 4.834233593 | 0.00039119 | 0.015411646 | 0.265970659 |
| FCGR2B | 2.733892323 | 6.804459544 | 4.818199517 | 0.000402102 | 0.015645999 | 0.238844999 |
| LEPRE1 | 2.275479206 | 8.098382114 | 4.804628691 | 0.000411587 | 0.015781506 | 0.215859291 |
| GMFG | 2.297820518 | 9.00821835 | 4.804265968 | 0.000411844 | 0.015781506 | 0.215244585 |
| ALDH1A1 | -2.490448893 | 8.518124416 | -4.8001408 | 0.000414775 | 0.015834574 | 0.208252406 |
| MSL3L1 | 2.159310838 | 7.560746512 | 4.794422636 | 0.000418874 | 0.01587085 | 0.198556295 |
| CDC42BPA | 2.012578212 | 6.239826604 | 4.784881653 | 0.000425808 | 0.015976687 | 0.182368147 |
| LOC100132091 | 2.738811073 | 6.153122213 | 4.778865821 | 0.000430242 | 0.016095143 | 0.172154819 |
| LPAR4 | 2.371933295 | 5.058605702 | 4.745361942 | 0.000455833 | 0.016707808 | 0.115185335 |
| AFAP1L2 | 2.191846791 | 8.473755552 | 4.744887983 | 0.000456206 | 0.016707808 | 0.114378349 |
| IRX5 | -2.860673383 | 7.115638549 | -4.741924869 | 0.000458547 | 0.016707808 | 0.109332534 |
| POPDC2 | 2.374377461 | 6.713711795 | 4.735000365 | 0.000464066 | 0.01681171 | 0.097536422 |
| ESAM | 3.0310242 | 10.25416675 | 4.719950573 | 0.0004763 | 0.017085845 | 0.07187676 |
| HLF | -2.452680297 | 5.523988327 | -4.706534511 | 0.000487491 | 0.017361017 | 0.048977428 |
| CD9 | -2.719157107 | 8.330413769 | -4.69872816 | 0.000494129 | 0.017541975 | 0.035642215 |
| LRRC33 | 2.123603637 | 7.48977885 | 4.697297408 | 0.000495355 | 0.017541975 | 0.033197264 |

| | | | | | | |
|---|---|---|---|---|---|---|
| SLC6A9 | 2.213148354 | 7.967474733 | 4.675632617 | 0.000514324 | 0.017961438 | -0.003857385 |
| SYTL2 | 2.140249753 | 6.94813001 | 4.66873225 | 0.000520523 | 0.018127724 | -0.015672326 |
| VASH1 | 2.725625181 | 8.200383549 | 4.65818838 | 0.000530147 | 0.018412024 | -0.033737645 |
| IFIT3 | 3.131852021 | 7.485823445 | 4.649705508 | 0.000538025 | 0.01858322 | -0.048282166 |
| TCEAL7 | 2.213769418 | 6.470345738 | 4.642914535 | 0.000544419 | 0.018600236 | -0.059932473 |
| LOC100133627 | 2.372843712 | 5.643042122 | 4.636610431 | 0.000550425 | 0.018704081 | -0.070752819 |
| ADAMTS9 | 2.588709439 | 8.280154035 | 4.616662413 | 0.000569892 | 0.019040414 | -0.105024997 |
| G3BP1 | 2.232018289 | 5.763154778 | 4.610333893 | 0.000576217 | 0.019142415 | -0.115908445 |
| DIO3 | 2.579669149 | 6.199471579 | 4.609564644 | 0.00057699 | 0.019142415 | -0.117231704 |
| SDCCAG8 | 2.098098453 | 4.810009173 | 4.597795801 | 0.000588964 | 0.01933612 | -0.137485751 |
| RASL11A | 2.00972665 | 6.217748612 | 4.587804505 | 0.000599332 | 0.019625414 | -0.154694371 |
| TGFB1I1 | 2.411228637 | 9.728530026 | 4.566570143 | 0.000622005 | 0.020103329 | -0.191309185 |
| SERPINE1 | 2.502192366 | 7.171163168 | 4.541762841 | 0.000649628 | 0.020525967 | -0.234155956 |
| LYL1 | 2.376999989 | 9.255731194 | 4.534841503 | 0.000657561 | 0.020610704 | -0.246123944 |
| AURKA | 2.288140747 | 6.183729273 | 4.527184883 | 0.000666454 | 0.020694541 | -0.259370193 |
| ISG15 | 3.146107373 | 9.774391931 | 4.525267415 | 0.000668701 | 0.020713286 | -0.262688612 |
| DDR2 | 3.000983245 | 8.481629799 | 4.513913977 | 0.000682166 | 0.020859674 | -0.282346362 |
| TRO | 2.067174654 | 6.828780358 | 4.499410929 | 0.000699778 | 0.021028938 | -0.307480269 |
| PLXND1 | 2.174059451 | 7.281354777 | 4.487920569 | 0.000714067 | 0.021231706 | -0.327411225 |
| EDNRA | 2.7387625 | 9.96142195 | 4.478802682 | 0.000725622 | 0.021374186 | -0.343238237 |
| IDO2 | 2.920145205 | 6.540799071 | 4.462300913 | 0.000747031 | 0.021750162 | -0.371907622 |
| LOC100129681 | 2.534128314 | 8.971998356 | 4.456572083 | 0.000754616 | 0.021869994 | -0.381868194 |
| LOC652377 | 3.140342861 | 6.402070311 | 4.449422643 | 0.000764195 | 0.022046239 | -0.394304197 |
| TRIP13 | 2.255392152 | 6.221606689 | 4.446476615 | 0.000768179 | 0.022065348 | -0.399430385 |
| LOC285929 | -2.454279231 | 5.438332986 | -4.438297474 | 0.000779352 | 0.022204962 | -0.413667721 |
| HRC | 3.115750377 | 7.275236327 | 4.430213272 | 0.000790563 | 0.022217615 | -0.427747513 |

| | | | | | | |
|---|---|---|---|---|---|---|
| TM4SF18 | 2.281900872 | 8.404443026 | 4.413068631 | 0.000814895 | 0.022350356 | -0.45763259 |
| CD93 | 3.453789347 | 11.19258302 | 4.388541123 | 0.000851066 | 0.022968291 | -0.500445966 |
| IGF1 | 2.981982598 | 6.002435117 | 4.377507799 | 0.000867877 | 0.023229985 | -0.519727382 |
| TPX2 | 2.873514561 | 6.451205148 | 4.359373797 | 0.00089626 | 0.023687943 | -0.55144767 |
| ZNF792 | 2.03299972 | 6.891561805 | 4.345701523 | 0.000918296 | 0.02402963 | -0.575387893 |
| EFNB1 | 2.463580238 | 7.460748562 | 4.340495156 | 0.000926834 | 0.024141728 | -0.584509757 |
| ARHGAP4 | 2.729760058 | 7.713105931 | 4.332179976 | 0.000940642 | 0.024450874 | -0.599084691 |
| ALCAM | -2.726683104 | 6.757759846 | -4.320993526 | 0.000959555 | 0.024840074 | -0.618704471 |
| SLC2A1 | 3.450879705 | 10.19156392 | 4.319106727 | 0.000962784 | 0.024872585 | -0.622015063 |
| COL4A1 | 2.703220267 | 13.0087334 | 4.314137485 | 0.000971342 | 0.02492913 | -0.630735999 |
| FHOD1 | 2.333790747 | 8.084544401 | 4.294437371 | 0.001006052 | 0.025417429 | -0.665335854 |
| MEF2A | 2.263266618 | 6.619961963 | 4.289297732 | 0.001015317 | 0.025549116 | -0.67436966 |
| LOC728188 | 2.396598298 | 7.239177607 | 4.285862883 | 0.001021559 | 0.025603963 | -0.680408586 |
| GPR162 | 2.942889798 | 7.47648959 | 4.262873787 | 0.001064369 | 0.026362487 | -0.720858981 |
| MRVI1 | 2.163724316 | 5.867154995 | 4.261385946 | 0.001067203 | 0.02638085 | -0.72347884 |
| CYTSB | 2.065844825 | 4.89264976 | 4.256779825 | 0.001076027 | 0.026414648 | -0.731590997 |
| FBP1 | -2.418009337 | 5.837836467 | -4.255212074 | 0.001079047 | 0.026414648 | -0.734352579 |
| LOC642342 | -2.022119927 | 5.011520565 | -4.252763076 | 0.001083782 | 0.026427941 | -0.738666991 |
| FSCN1 | 2.04242084 | 11.70641098 | 4.243674392 | 0.001101546 | 0.02655295 | -0.754684065 |
| NRP1 | 2.031713319 | 8.723969597 | 4.235814477 | 0.001117151 | 0.02677019 | -0.768542617 |
| SQLE | 2.040566057 | 8.005757119 | 4.231694617 | 0.001125422 | 0.02677019 | -0.775809289 |
| LOC100134134 | 2.54438205 | 12.01525068 | 4.228212916 | 0.001132462 | 0.026786013 | -0.781951736 |
| CYGB | 2.469465115 | 9.938343364 | 4.225735985 | 0.001137497 | 0.026848609 | -0.78632232 |
| SFRP1 | -3.103353305 | 8.565631204 | -4.222052023 | 0.00114503 | 0.026931696 | -0.792823895 |
| KCNMB3 | 2.58388571 | 6.599853314 | 4.205927531 | 0.001178611 | 0.027423078 | -0.82129727 |
| ARAP3 | 2.271609763 | 8.549772501 | 4.202570247 | 0.001185731 | 0.027428449 | -0.827229044 |

| | | | | | | |
|---|---|---|---|---|---|---|
| C16orf30 | 2.017106791 | 8.238208164 | 4.200646772 | 0.001189829 | 0.027472856 | -0.830628026 |
| CCL15 | -3.366503806 | 8.304876732 | -4.194890478 | 0.001202184 | 0.027640621 | -0.84080223 |
| CD4 | 2.223276904 | 5.818349164 | 4.191970915 | 0.001208501 | 0.027640621 | -0.845963809 |
| OAS2 | 2.481489386 | 9.286643847 | 4.177806559 | 0.001239638 | 0.027640621 | -0.871017483 |
| Gcom1 | 2.730706828 | 6.445328549 | 4.172964496 | 0.001250471 | 0.027705963 | -0.879586617 |
| TK1 | 3.684419285 | 6.282212727 | 4.157435323 | 0.001285882 | 0.028234834 | -0.907084615 |
| FOXC1 | -2.353112552 | 10.40432711 | -4.145961018 | 0.001312711 | 0.028289487 | -0.927417661 |
| GPSM3 | 2.975786908 | 6.907267524 | 4.129971351 | 0.001351067 | 0.028370212 | -0.95577334 |
| DENND2A | 2.210472961 | 8.31233988 | 4.119003338 | 0.001378046 | 0.028491008 | -0.975237847 |
| TMEM136 | 2.367744583 | 6.053643362 | 4.106584146 | 0.001409267 | 0.028795176 | -0.997291402 |
| THOC4 | 2.354586767 | 8.413469521 | 4.082732794 | 0.001471293 | 0.029353617 | -1.039685979 |
| COL6A2 | 2.036397459 | 10.16489875 | 4.070191773 | 0.001505031 | 0.029757986 | -1.0619979 |
| CHN1 | 3.347950285 | 8.624453846 | 4.043029741 | 0.001580868 | 0.030293506 | -1.110370622 |
| NR2F2 | 2.458146646 | 8.816052381 | 4.029752298 | 0.001619363 | 0.030516163 | -1.134039987 |
| PLLP | -2.355741168 | 6.378081663 | -4.015364609 | 0.001662171 | 0.031150996 | -1.159705746 |
| CCR10 | 2.042204478 | 5.18094741 | 4.014566183 | 0.00166458 | 0.031150996 | -1.161130551 |
| LIX1L | 2.448991658 | 6.796174604 | 4.0114014 | 0.001674166 | 0.031222203 | -1.166778697 |
| C10orf11 | 4.383742497 | 5.613453854 | 4.004608867 | 0.001694933 | 0.031331403 | -1.178904099 |
| IFIT2 | 2.181400502 | 8.805480781 | 4.00024001 | 0.00170843 | 0.031498027 | -1.18670503 |
| LOC399942 | 2.518738698 | 7.531492592 | 3.998991032 | 0.001712309 | 0.031509286 | -1.188935469 |
| FLJ41603 | -3.033954471 | 6.239163069 | -3.997221046 | 0.001717821 | 0.031523395 | -1.192096555 |
| P2RY14 | 2.920153725 | 5.709338849 | 3.988172809 | 0.001746289 | 0.031822018 | -1.20826021 |
| LOC728059 | 3.198458618 | 6.774365363 | 3.968055288 | 0.001811337 | 0.032528163 | -1.24422197 |
| MPZL2 | -3.037723456 | 6.311006877 | -3.964626877 | 0.001822669 | 0.032528877 | -1.250353813 |
| CXCL10 | 3.120194661 | 6.301987438 | 3.957244846 | 0.001847318 | 0.032689878 | -1.263560046 |
| RCAN1 | 2.709969301 | 9.734650235 | 3.940177391 | 0.00190563 | 0.033116798 | -1.294109705 |

| | | | | | | |
|---|---|---|---|---|---|---|
| GPER | 2.84141989 | 8.688062892 | 3.93905491 | 0.00190953 | 0.033116798 | -1.296119672 |
| TMEM44 | 2.446488376 | 8.039620482 | 3.933340574 | 0.001929516 | 0.033215287 | -1.306353547 |
| SEC14L1 | 2.065267169 | 6.911578534 | 3.929915686 | 0.001941598 | 0.033238411 | -1.312488426 |
| FAM13C1 | 3.4929635 | 5.326765249 | 3.926644062 | 0.001953212 | 0.03332089 | -1.318349606 |
| STARD3NL | 2.666729977 | 6.603582292 | 3.922233561 | 0.001968981 | 0.033499254 | -1.326252401 |
| CKB | -2.65836797 | 7.65879643 | -3.920696508 | 0.001974508 | 0.033502852 | -1.32900686 |
| AVPI1 | -2.237733152 | 9.132427438 | -3.918146257 | 0.001983712 | 0.033554034 | -1.333577403 |
| RASL12 | 2.10478387 | 9.997888948 | 3.918116539 | 0.00198382 | 0.033554034 | -1.333630667 |
| MT1X | -2.12737555 | 10.07966103 | -3.914006379 | 0.001998749 | 0.033642488 | -1.34099791 |
| C6orf225 | 2.006661831 | 5.512237057 | 3.887355821 | 0.002098393 | 0.034617738 | -1.388798011 |
| MKL1 | 2.002701344 | 6.09745816 | 3.88677401 | 0.002100624 | 0.034617738 | -1.389842118 |
| USHBP1 | 2.446825729 | 5.489764872 | 3.885587321 | 0.002105183 | 0.034647577 | -1.391971804 |
| P704P | 2.512764442 | 5.764032858 | 3.881587516 | 0.002120624 | 0.034810821 | -1.399150781 |
| SDC4 | -2.738214628 | 6.724726151 | -3.871156878 | 0.002161442 | 0.0350248 | -1.417877388 |
| ZNF485 | -2.087579885 | 3.573288332 | -3.867681656 | 0.00217522 | 0.035199209 | -1.424118321 |
| SH2B3 | 2.566968962 | 9.301378283 | 3.86501532 | 0.002185852 | 0.035239172 | -1.4289072 |
| LOC158376 | 2.354650924 | 5.384072546 | 3.860998581 | 0.00220197 | 0.035318359 | -1.436122407 |
| C8orf4 | 2.742515653 | 9.386028846 | 3.849717611 | 0.002247893 | 0.035781796 | -1.456392197 |
| HEYL | 3.560628187 | 9.582521309 | 3.825019115 | 0.002351903 | 0.036722579 | -1.500800761 |
| SHE | 2.221037333 | 7.668599847 | 3.813159203 | 0.002403591 | 0.037089433 | -1.522139447 |
| FILIP1 | 2.415173258 | 6.318956942 | 3.803602967 | 0.002446086 | 0.037243726 | -1.539339843 |
| CDK6 | 2.116346872 | 8.919462327 | 3.79440026 | 0.002487738 | 0.037488649 | -1.555909333 |
| EPAS1 | 2.192782434 | 11.98498067 | 3.792559994 | 0.002496154 | 0.037507763 | -1.559223365 |
| PDE1A | 2.26383244 | 6.012956322 | 3.787746412 | 0.002518306 | 0.03764454 | -1.567892859 |
| MXRA5 | 2.193386575 | 11.46335085 | 3.787375433 | 0.002520022 | 0.03764454 | -1.568561068 |
| ECHDC2 | -2.006679682 | 9.341457854 | -3.771184772 | 0.002596076 | 0.038324034 | -1.597731866 |

| | | | | | | |
|---|---|---|---|---|---|---|
| C9orf24 | -2.688630003 | 4.1905277 | -3.762954835 | 0.002635633 | 0.038636539 | -1.612565742 |
| C5orf39 | 2.08495415 | 7.211493279 | 3.760085149 | 0.002649571 | 0.038688706 | -1.617739073 |
| TSPAN15 | 2.031833051 | 6.92722509 | 3.759953193 | 0.002650214 | 0.038688706 | -1.617976968 |
| C1orf133 | -2.113724892 | 5.965359447 | -3.747026304 | 0.002713958 | 0.039418049 | -1.641286871 |
| C10orf72 | 2.129969294 | 5.953012447 | 3.741270962 | 0.002742842 | 0.039481851 | -1.651667992 |
| RASSF7 | -2.389200998 | 7.247572318 | -3.736851718 | 0.002765233 | 0.039481851 | -1.659640389 |
| TSPAN8 | -2.473985767 | 6.956988509 | -3.733040139 | 0.002784697 | 0.03954091 | -1.666517405 |
| CD163L1 | 3.02933982 | 5.495995806 | 3.732292289 | 0.002788532 | 0.039544458 | -1.667866802 |
| LOC643977 | 3.682506709 | 5.370493617 | 3.710979586 | 0.002900135 | 0.040942888 | -1.706335287 |
| USF1 | 2.046350402 | 7.115278262 | 3.708007462 | 0.002916057 | 0.041105575 | -1.711701727 |
| CA12 | -3.256375713 | 7.350819097 | -3.70713454 | 0.002920751 | 0.041105575 | -1.713277953 |
| CDH6 | 2.968083537 | 8.052293627 | 3.692349889 | 0.00300143 | 0.041672935 | -1.739980271 |
| LOC646383 | -2.963670411 | 2.827282646 | -3.685109711 | 0.003041767 | 0.042093915 | -1.753060582 |
| NES | 2.296550597 | 8.799964064 | 3.679139041 | 0.003075448 | 0.042494532 | -1.763849268 |
| GTSF1 | 2.907212834 | 5.426282157 | 3.675114587 | 0.003098366 | 0.04264182 | -1.771122198 |
| FAM20A | -2.029791313 | 4.498393069 | -3.674539716 | 0.003101654 | 0.04264182 | -1.772161158 |
| LOC647597 | 2.121955967 | 7.060864407 | 3.662560598 | 0.003170984 | 0.043192989 | -1.793814411 |
| TEK | 2.217475224 | 9.178299543 | 3.644081973 | 0.00328105 | 0.043870104 | -1.82722871 |
| GEFT | 2.097469689 | 5.210506529 | 3.642935116 | 0.003288009 | 0.043870104 | -1.829303024 |
| EBF1 | 3.014515187 | 8.940799659 | 3.64229314 | 0.003291911 | 0.043870104 | -1.830464188 |
| TMEM8 | 2.168891693 | 6.792971609 | 3.638265451 | 0.003316498 | 0.044004333 | -1.8377496 |
| NDC80 | 3.599065209 | 5.53910524 | 3.637131837 | 0.003323452 | 0.044004333 | -1.83980024 |
| COCH | -3.153509425 | 5.740561027 | -3.628138661 | 0.00337915 | 0.044329804 | -1.856070249 |
| CCL14 | -3.847529791 | 8.39441433 | -3.624274465 | 0.003403374 | 0.0444846 | -1.863062176 |
| THY1 | 3.767555817 | 10.50571015 | 3.622608138 | 0.003413875 | 0.044553539 | -1.866077434 |
| ATP9A | -2.189334195 | 9.441469642 | -3.606836188 | 0.003514918 | 0.045325882 | -1.894622548 |

| | | | | | | |
|---|---|---|---|---|---|---|
| LOC645993 | 3.374540024 | 4.275792972 | 3.603826557 | 0.003534543 | 0.045423021 | -1.900070657 |
| IGF2AS | 2.201513414 | 4.226195102 | 3.598384474 | 0.003570314 | 0.045667133 | -1.909922905 |
| ERAP2 | 2.548618377 | 7.542826654 | 3.595941383 | 0.003586493 | 0.045689901 | -1.914346185 |
| RPESP | -2.471882651 | 6.600406391 | -3.595809693 | 0.003587367 | 0.045689901 | -1.914584619 |
| C1QA | 2.085632549 | 6.984560282 | 3.59499594 | 0.003592774 | 0.045705451 | -1.916057991 |
| ECSCR | 2.125293977 | 10.0945334 | 3.593596834 | 0.003602089 | 0.045777764 | -1.918591254 |
| KCNMA1 | -3.626226759 | 5.556211343 | -3.584966203 | 0.003660101 | 0.046004875 | -1.934219665 |
| LRRC8E | -2.224457032 | 4.472786007 | -3.577625688 | 0.00371019 | 0.046328532 | -1.947513956 |
| UHRF1 | 2.24146941 | 7.168965579 | 3.576955951 | 0.003714795 | 0.046328532 | -1.948726997 |
| C1QTNF5 | 2.94357866 | 9.594689002 | 3.575838663 | 0.003722489 | 0.046328532 | -1.950750683 |
| C1QC | 2.638894672 | 9.28109759 | 3.575290046 | 0.003726274 | 0.046328532 | -1.951744382 |
| MPRIP | 2.193497425 | 9.413453645 | 3.56772223 | 0.003778877 | 0.046757846 | -1.965452804 |
| C12orf35 | 2.33583362 | 7.849200815 | 3.566435193 | 0.003787899 | 0.046757846 | -1.96778434 |
| MORC2 | 2.190171256 | 6.077893624 | 3.558315099 | 0.003845323 | 0.047103709 | -1.982495537 |
| FAM107A | -2.247298339 | 8.402422925 | -3.554664746 | 0.003871426 | 0.047202196 | -1.989109556 |
| LOC646332 | 3.629435239 | 4.506231908 | 3.550180234 | 0.003903742 | 0.047458507 | -1.997235526 |
| CDC20 | 2.289504305 | 8.119468178 | 3.547132965 | 0.003925857 | 0.047552052 | -2.002757541 |
| FAM127C | 2.01143036 | 6.426052124 | 3.546983939 | 0.003926942 | 0.047552052 | -2.003027602 |
| LYPLA2P1 | 2.171646156 | 4.521272188 | 3.536679439 | 0.004002698 | 0.048127479 | -2.021702605 |
| ALDH1A2 | -2.597282725 | 6.110630091 | -3.529779197 | 0.004054257 | 0.048601918 | -2.034209667 |
| KCNAB1 | 2.767174 | 9.308379517 | 3.52953956 | 0.00405606 | 0.048601918 | -2.034644045 |
| SHROOM2 | 2.25249362 | 6.329190254 | 3.529085637 | 0.004059476 | 0.048601918 | -2.035466854 |
| SLC9A2 | -2.32017272 | 4.269286141 | -3.522800579 | 0.00410709 | 0.048847244 | -2.046860075 |
| LOC100134361 | -2.444248585 | 5.216666571 | -3.506358043 | 0.004234359 | 0.049797166 | -2.076670906 |
| ZCCHC3 | 2.09265897 | 6.117123748 | 3.50291599 | 0.004261505 | 0.049863519 | -2.082912265 |
| SMC4 | 2.003770095 | 8.357406879 | 3.497105188 | 0.004307736 | 0.050191887 | -2.093449399 |

| | | | | | | |
|---|---|---|---|---|---|---|
| CD248 | 2.387458427 | 10.9681858 | 3.492649308 | 0.004343532 | 0.050469034 | -2.101530054 |
| RNASE1 | 2.217050499 | 9.708934377 | 3.466226057 | 0.004562105 | 0.052285869 | -2.149456191 |
| FLJ90757 | 2.005841136 | 5.201445142 | 3.464916487 | 0.004573224 | 0.052365698 | -2.1518318 |
| ANTXR2 | 2.695675839 | 9.54527458 | 3.462896455 | 0.004590429 | 0.052409419 | -2.155496269 |
| PLAGL1 | 2.025643108 | 5.175457633 | 3.443029556 | 0.004763188 | 0.053520061 | -2.191539451 |
| GPR116 | 2.141548346 | 10.83760352 | 3.441575247 | 0.004776091 | 0.053521388 | -2.194178125 |
| FAM46C | -2.328430564 | 6.872858745 | -3.439973145 | 0.004790346 | 0.053538909 | -2.197084982 |
| PPAP2C | -2.275094445 | 4.945184282 | -3.419772519 | 0.004973837 | 0.054659865 | -2.233739609 |
| SOX18 | 2.162311309 | 11.28763908 | 3.412467554 | 0.005041934 | 0.055033469 | -2.246995687 |
| LOC641700 | 3.945815145 | 5.960538393 | 3.41080344 | 0.005057578 | 0.055055951 | -2.250015564 |
| CHI3L1 | -2.158013475 | 6.441722103 | -3.393074121 | 0.005227343 | 0.055848397 | -2.282190241 |
| PROCR | 2.13313956 | 8.322999646 | 3.381764043 | 0.005338648 | 0.056321616 | -2.302716322 |
| PCDH18 | 2.065307631 | 9.176913777 | 3.377169597 | 0.005384546 | 0.056616322 | -2.31105467 |
| IVD | 2.233670225 | 5.536163333 | 3.354032272 | 0.005621835 | 0.058179918 | -2.353046403 |
| C1QB | 3.132354192 | 8.810735884 | 3.34571156 | 0.005709734 | 0.058713395 | -2.368147472 |
| PPL | -3.222230451 | 7.091342955 | -3.341815582 | 0.005751368 | 0.058990584 | -2.375218124 |
| LOC647251 | 2.532098385 | 4.795083278 | 3.327839394 | 0.005903267 | 0.059882146 | -2.400582379 |
| NR1D1 | -2.050230912 | 4.007975444 | -3.315923944 | 0.00603597 | 0.060588553 | -2.422205804 |
| LRRC1 | -2.626343333 | 7.143546362 | -3.312548908 | 0.006074104 | 0.060694608 | -2.428330406 |
| LOC728505 | 2.193807478 | 6.278139353 | 3.31185806 | 0.006081939 | 0.060708636 | -2.429584061 |
| HIC1 | 2.128453819 | 5.340274972 | 3.30269437 | 0.006186846 | 0.061367084 | -2.446212599 |
| MGC2752 | 2.238328874 | 4.582940646 | 3.299477594 | 0.006224104 | 0.061599854 | -2.452049591 |
| TBX15 | 2.991709569 | 7.419460807 | 3.297610494 | 0.006245833 | 0.061658054 | -2.455437477 |
| OSR2 | -2.216028181 | 6.289704541 | -3.294582386 | 0.006281237 | 0.061711461 | -2.460931945 |
| SCG2 | 4.058854255 | 5.24414054 | 3.294104632 | 0.006286842 | 0.061711461 | -2.461798815 |
| ARHGEF17 | 2.265727787 | 7.904103743 | 3.289461781 | 0.006341569 | 0.061975315 | -2.470222966 |

| | | | | | | |
|---|---|---|---|---|---|---|
| INPP4B | 2.473940139 | 7.104261209 | 3.284418073 | 0.006401566 | 0.062032703 | -2.479374124 |
| MGC4677 | 2.110478314 | 10.2216043 | 3.269369363 | 0.006584007 | 0.063169389 | -2.506675831 |
| MAOA | -2.532576096 | 8.837959768 | -3.266753969 | 0.006616246 | 0.063368571 | -2.511420364 |
| CDCA5 | 2.753307495 | 6.85997045 | 3.260046983 | 0.006699649 | 0.063763537 | -2.523586816 |
| NFKBIE | 2.653590638 | 4.684176998 | 3.258937949 | 0.006713542 | 0.063785843 | -2.525598521 |
| ASS1 | -2.704797436 | 8.43115895 | -3.250355759 | 0.00682204 | 0.064327193 | -2.541165168 |
| RUNX2 | 2.264229942 | 4.670845195 | 3.239968177 | 0.00695573 | 0.065149251 | -2.560004458 |
| HN1 | 2.727858193 | 5.619696271 | 3.232620704 | 0.007051886 | 0.065619477 | -2.573328648 |
| CES1 | -3.707257331 | 5.953177406 | -3.232204594 | 0.007057371 | 0.065619477 | -2.574083199 |
| MUC1 | -3.286786484 | 6.692840175 | -3.224122569 | 0.007164772 | 0.066351945 | -2.588737878 |
| NLF2 | 2.107010079 | 7.295556712 | 3.211597332 | 0.007334482 | 0.067345819 | -2.61144585 |
| EFNB3 | 2.182402829 | 6.853767028 | 3.176881462 | 0.007826318 | 0.070421027 | -2.674360849 |
| ADCY4 | 2.660740346 | 9.164669364 | 3.158089658 | 0.008106254 | 0.072297562 | -2.708399967 |
| HRIHFB2122 | 2.937409501 | 4.294209727 | 3.157915976 | 0.008108888 | 0.072297562 | -2.708714508 |
| LYPD1 | 2.807282158 | 6.323694325 | 3.154281482 | 0.008164199 | 0.072445562 | -2.715296408 |
| MGC61598 | 2.096532682 | 9.332549193 | 3.140464557 | 0.00837795 | 0.07324606 | -2.740313388 |
| PKMYT1 | 2.675416927 | 4.109703319 | 3.133005557 | 0.00849567 | 0.073826745 | -2.753815372 |
| FAM108C1 | -2.011141413 | 7.331463131 | -3.115228904 | 0.008782962 | 0.075091259 | -2.785983996 |
| KIAA0100 | 2.39795025 | 4.309708196 | 3.102162886 | 0.009000321 | 0.076301483 | -2.809618772 |
| HOXC6 | 2.863896706 | 8.255367726 | 3.098288102 | 0.00906581 | 0.076537501 | -2.816626141 |
| ACOT2 | 2.438858858 | 7.264190703 | 3.093480319 | 0.009147734 | 0.076983972 | -2.82531973 |
| GOLPH4 | 2.00722232 | 7.68926944 | 3.080659484 | 0.009369842 | 0.077765371 | -2.848496841 |
| HDAC7 | 2.233818593 | 6.563549171 | 3.077806016 | 0.009420006 | 0.077976373 | -2.853654044 |
| ADAM9 | 2.015366964 | 4.697921389 | 3.068337348 | 0.0095884 | 0.07885988 | -2.870763943 |
| DZIP1 | 2.944308539 | 4.796384327 | 3.059690028 | 0.009744818 | 0.07972286 | -2.886385171 |
| CD36 | 2.75243133 | 10.27399863 | 3.057298683 | 0.009788524 | 0.079924818 | -2.890704317 |

| | | | | | | |
|---|---|---|---|---|---|---|
| ADAP2 | 3.283776408 | 8.549615005 | 3.055816882 | 0.009815704 | 0.07996325 | -2.893380511 |
| LOC652815 | 2.068283252 | 7.24417261 | 3.054353281 | 0.009842625 | 0.080092109 | -2.896023704 |
| GUCY1A3 | 2.048062112 | 9.001656323 | 3.049697769 | 0.009928748 | 0.08059995 | -2.904430475 |
| COL5A2 | 2.597898767 | 10.64028125 | 3.043065295 | 0.010052746 | 0.080806553 | -2.916404872 |
| SLC7A1 | -2.388982927 | 7.742642945 | -3.042852363 | 0.010056753 | 0.080806553 | -2.916789258 |
| ATP8B2 | 2.21076372 | 5.794123041 | 3.041881642 | 0.010075038 | 0.080850415 | -2.918541572 |
| NUP50 | 2.085610021 | 5.586834886 | 3.031201431 | 0.010278424 | 0.081909033 | -2.937817172 |
| CAMK1 | 2.075562722 | 6.981917854 | 3.027928078 | 0.010341577 | 0.082204453 | -2.943723419 |
| CEACAM1 | 3.098665825 | 7.301421882 | 3.019748079 | 0.010501095 | 0.082742065 | -2.958479808 |
| KIF21A | -2.370905218 | 6.00277716 | -3.017175045 | 0.010551778 | 0.083037619 | -2.963120522 |
| GINS2 | 2.215921831 | 6.167949323 | 3.01479172 | 0.010598942 | 0.083164919 | -2.967418669 |
| SLC31A2 | -2.253533252 | 7.688019084 | -3.014691106 | 0.010600938 | 0.083164919 | -2.96760011 |
| SOX15 | -2.739671776 | 6.367072453 | -3.011018904 | 0.010674034 | 0.083478628 | -2.974221855 |
| CDCA7 | 2.163842343 | 7.974392911 | 3.00575694 | 0.010779653 | 0.084065005 | -2.983708619 |
| SLC35F2 | -2.733273204 | 5.789600545 | -3.002706547 | 0.010841358 | 0.084232269 | -2.989207253 |
| TMTC1 | -2.332535246 | 6.070361129 | -2.998707568 | 0.010922786 | 0.084480712 | -2.996414789 |
| PDGFRB | 2.075716051 | 11.96500828 | 2.993838356 | 0.011022758 | 0.084971446 | -3.005189209 |
| IL6 | -3.544855483 | 4.04107445 | -2.985321086 | 0.01119983 | 0.085574506 | -3.020533265 |
| TRIM27 | 2.728062644 | 5.231579981 | 2.984360655 | 0.011219974 | 0.085675478 | -3.022263161 |
| IFI44 | 2.007042458 | 8.200124708 | 2.983439335 | 0.011239332 | 0.085719455 | -3.023922543 |
| RERGL | -2.81408088 | 6.562973507 | -2.975755677 | 0.011402076 | 0.086385804 | -3.037758986 |
| RGS4 | 2.239202512 | 6.013669066 | 2.973971273 | 0.011440206 | 0.086426326 | -3.040971616 |
| RAD51AP1 | 2.007374979 | 6.205107302 | 2.965281783 | 0.011627709 | 0.087256269 | -3.056612518 |
| C20orf160 | 3.992988112 | 7.522266039 | 2.955884851 | 0.011833925 | 0.088382877 | -3.073519938 |
| SH3RF2 | -2.488677169 | 5.47476633 | -2.954971099 | 0.011854171 | 0.088429309 | -3.075163618 |
| CLIP2 | 2.047690155 | 7.002529563 | 2.942721582 | 0.012128932 | 0.089290367 | -3.097191582 |

| | | | | | | |
|---|---|---|---|---|---|---|
| DLEU2 | 2.210321698 | 4.626425769 | 2.919742518 | 0.012661567 | 0.091632824 | -3.138479021 |
| S1PR3 | 2.136205948 | 7.257788023 | 2.918524664 | 0.012690436 | 0.09178906 | -3.140665865 |
| COL6A3 | 2.088836971 | 11.60801026 | 2.91185144 | 0.01284979 | 0.092303264 | -3.152646243 |
| SETD4 | 2.208708097 | 4.293823549 | 2.9023385 | 0.013080397 | 0.093008781 | -3.169717524 |
| ENC1 | 2.533073375 | 7.409622285 | 2.898204661 | 0.013181885 | 0.093152478 | -3.177133141 |
| CFB | -2.095656785 | 7.20145455 | -2.896747777 | 0.013217838 | 0.093354219 | -3.179746225 |
| ITGA5 | 2.232335203 | 9.625648444 | 2.87466504 | 0.01377487 | 0.095356682 | -3.219328384 |
| LAMC3 | 2.064370687 | 8.475152673 | 2.869304723 | 0.013913558 | 0.095851491 | -3.228929024 |
| CCL8 | 2.555862358 | 7.728644657 | 2.856996864 | 0.014237263 | 0.096811851 | -3.250961746 |
| RIPK4 | -2.039235565 | 5.770501606 | -2.845046712 | 0.014558697 | 0.098120919 | -3.272338604 |
| GBP1 | 2.097037129 | 8.008720568 | 2.842101724 | 0.014639009 | 0.098413803 | -3.277604305 |
| NBPF14 | 2.960962586 | 5.552067576 | 2.833828862 | 0.01486697 | 0.099477963 | -3.292391187 |
| GPT2 | -2.180515824 | 8.007826706 | -2.832776265 | 0.014896225 | 0.099497934 | -3.294272045 |
| CRHBP | 2.118648377 | 6.422874291 | 2.828940028 | 0.015003331 | 0.099675305 | -3.301125844 |
| OSBPL10 | 3.168196696 | 7.681573676 | 2.827423667 | 0.015045877 | 0.099833352 | -3.303834505 |
| THAP10 | 2.020561347 | 5.971781023 | 2.82033664 | 0.015246311 | 0.100409708 | -3.316490475 |
| LOC642956 | 2.172753616 | 10.20478778 | 2.815128688 | 0.015395283 | 0.100700662 | -3.325787079 |
| FAM89A | 2.353265184 | 9.046061157 | 2.814709357 | 0.015407341 | 0.100700662 | -3.32653548 |
| ProSAPiP1 | -2.021269453 | 4.582567754 | -2.814678385 | 0.015408232 | 0.100700662 | -3.326590757 |
| TNMD | 2.297670302 | 7.725221904 | 2.811890511 | 0.015488635 | 0.101068957 | -3.331565871 |
| TNN | 3.743653068 | 5.101776421 | 2.805815166 | 0.015665291 | 0.101486291 | -3.342404455 |
| HEPH | 2.093016375 | 6.619234134 | 2.804188007 | 0.015712942 | 0.10169048 | -3.345306599 |
| RSAD2 | 2.262401263 | 5.19078761 | 2.8000689 | 0.015834209 | 0.101927494 | -3.352651867 |
| GINS3 | 2.202667616 | 6.829274329 | 2.771990363 | 0.016685833 | 0.104838739 | -3.402666006 |
| DCHS1 | 2.425138947 | 7.060008379 | 2.759577942 | 0.01707656 | 0.1062021 | -3.424743157 |
| PPAN | 2.043212064 | 5.496722723 | 2.754561058 | 0.017237032 | 0.106680589 | -3.433660587 |

| | | | | | | |
|---|---|---|---|---|---|---|
| AKAP2 | 3.872712869 | 5.431117315 | 2.751406957 | 0.01733868 | 0.106941656 | -3.439265226 |
| CLDN8 | -2.858341565 | 5.665536547 | -2.745829478 | 0.017519875 | 0.107637948 | -3.449172766 |
| LOC441711 | 2.715700623 | 5.815534507 | 2.728983257 | 0.018078554 | 0.109417344 | -3.479071552 |
| CLDN15 | 2.034561766 | 7.724518019 | 2.723361416 | 0.018268867 | 0.1100935 | -3.489040392 |
| PLAC9 | 2.380572206 | 10.62606058 | 2.71509257 | 0.018552373 | 0.111164338 | -3.503694792 |
| LOC100133888 | 2.375203746 | 6.661071549 | 2.705561607 | 0.018884521 | 0.112353544 | -3.520573671 |
| JAG1 | 2.083642936 | 9.28189429 | 2.690617719 | 0.019417103 | 0.113818515 | -3.547011511 |
| CDK5R1 | 2.011992663 | 5.302850645 | 2.688987356 | 0.019476092 | 0.114043238 | -3.549893819 |
| PTGDS | -2.231027681 | 7.557028903 | -2.682841473 | 0.019700045 | 0.114927562 | -3.560755438 |
| APOE | 2.114040759 | 11.64681241 | 2.675776228 | 0.019960621 | 0.116071749 | -3.573234659 |
| LTBP2 | 2.244838244 | 8.916467287 | 2.6740922 | 0.020023228 | 0.116382128 | -3.576207982 |
| CIAPIN1 | 2.01541616 | 6.345597574 | 2.672948136 | 0.02006587 | 0.116576234 | -3.578227691 |
| LOC100132439 | 2.081655163 | 4.863466759 | 2.647604683 | 0.021033648 | 0.119532998 | -3.622915156 |
| ACSL1 | -2.564122978 | 8.370112255 | -2.647214943 | 0.021048882 | 0.119532998 | -3.623601563 |
| SPINT2 | -3.2265274 | 8.492782957 | -2.646341936 | 0.021083045 | 0.119586263 | -3.625139008 |
| SLC38A11 | 3.001719116 | 5.308165408 | 2.626585819 | 0.021870832 | 0.122104101 | -3.659897432 |
| MGC52282 | 3.35377584 | 3.350146541 | 2.611718611 | 0.022482581 | 0.123988584 | -3.686010734 |
| CKMT2 | -2.073186633 | 6.362055169 | -2.611002432 | 0.022512469 | 0.123990692 | -3.687267689 |
| SLC38A1 | -3.13139448 | 5.957130231 | -2.609055677 | 0.022593906 | 0.12411389 | -3.690683959 |
| TFAP2A | -2.445893153 | 5.233461753 | -2.605447745 | 0.022745597 | 0.124466256 | -3.69701359 |
| ARSK | 2.994767551 | 4.205762802 | 2.600201466 | 0.022967945 | 0.125179354 | -3.706213377 |
| RAPGEFL1 | -2.809598883 | 5.713559874 | -2.597691275 | 0.023075081 | 0.125607746 | -3.710613475 |
| CHSY3 | 2.054607259 | 7.318912528 | 2.596217893 | 0.023138192 | 0.125734133 | -3.713195631 |
| SP140 | -2.169431585 | 3.271880296 | -2.594990445 | 0.023190898 | 0.125811254 | -3.715346484 |
| ATP1B1 | -2.130898303 | 8.167137294 | -2.59374861 | 0.023244341 | 0.125930984 | -3.71752227 |
| NFAT5 | 2.427851284 | 7.735828004 | 2.590455054 | 0.023386662 | 0.126417178 | -3.723291476 |

| | | | | | | |
|---|---|---|---|---|---|---|
| ALDH1L1 | -2.802616743 | 5.708480974 | -2.569194597 | 0.024326033 | 0.129838397 | -3.760484949 |
| PTGDR | 2.092985823 | 4.875262797 | 2.565531261 | 0.024491565 | 0.130211712 | -3.766885185 |
| TCF7L1 | 2.147479689 | 6.038636556 | 2.563865832 | 0.024567181 | 0.130396858 | -3.76979403 |
| CFD | -2.1735101 | 11.77372019 | -2.548069116 | 0.025295804 | 0.132435299 | -3.797358355 |
| LOC388564 | -2.074561132 | 6.915919601 | -2.54464162 | 0.025456651 | 0.132634321 | -3.803332781 |
| ABCC3 | -2.244665728 | 5.269601999 | -2.538822921 | 0.025731993 | 0.133351299 | -3.813470032 |
| GPR124 | 2.270196831 | 9.034395481 | 2.527141479 | 0.026293527 | 0.135409514 | -3.833801082 |
| LOC113230 | -2.030318202 | 3.787907817 | -2.526683649 | 0.026315776 | 0.13546834 | -3.834597362 |
| PRC1 | 2.532059559 | 7.690317015 | 2.516341321 | 0.026823274 | 0.13643742 | -3.852573964 |
| RGS16 | 2.776594889 | 6.139556678 | 2.510742323 | 0.027101967 | 0.136872977 | -3.862296833 |
| NEK4 | 2.090108132 | 3.198598842 | 2.508381976 | 0.027220295 | 0.13710198 | -3.866393734 |
| LRRC26 | -3.622572859 | 6.749990027 | -2.500810441 | 0.027603258 | 0.138382194 | -3.879528016 |
| LOC731486 | -2.218027417 | 6.025170355 | -2.459333837 | 0.029795517 | 0.144585098 | -3.951261076 |
| MAPK13 | -2.679842424 | 7.048461748 | -2.451120084 | 0.030249203 | 0.145625527 | -3.965422012 |
| HSPB3 | -2.042877734 | 5.080422893 | -2.444363273 | 0.030627411 | 0.146619143 | -3.97705974 |
| PEAR1 | 2.502883191 | 6.836384488 | 2.440230132 | 0.030861006 | 0.147317192 | -3.984173433 |
| LOC285016 | 2.840128609 | 6.83079469 | 2.431808705 | 0.031342297 | 0.148657767 | -3.998655746 |
| CPB1 | 2.340473235 | 5.531592152 | 2.425001119 | 0.03173664 | 0.149795516 | -4.010350764 |
| LOC729646 | 2.84324126 | 5.94105303 | 2.423712787 | 0.031811806 | 0.149981837 | -4.012562824 |
| C14orf78 | -2.47105398 | 8.593411803 | -2.416551217 | 0.032232773 | 0.150614738 | -4.024852116 |
| OLFML2B | 2.577702863 | 9.298592638 | 2.39937105 | 0.033264633 | 0.153221493 | -4.054283736 |
| ATE1 | 2.048088473 | 4.310256324 | 2.392489287 | 0.033686813 | 0.154544997 | -4.066053051 |
| PERP | -3.191382415 | 6.805176288 | -2.389424281 | 0.033876498 | 0.15502033 | -4.07129116 |
| CSPG4 | 2.729302394 | 8.576103678 | 2.379805228 | 0.034478484 | 0.15612375 | -4.08771515 |
| PLXNA2 | 2.301190324 | 6.168447898 | 2.359890141 | 0.035757639 | 0.159461111 | -4.121645564 |
| HCLS1 | 2.050926413 | 8.410295914 | 2.346201273 | 0.036663144 | 0.162009204 | -4.144909434 |

| | | | | | | |
|---|---|---|---|---|---|---|
| TSPAN14 | 2.894080839 | 7.62011755 | 2.337652777 | 0.037239717 | 0.163582268 | -4.159412699 |
| CACNA1H | 2.220561469 | 7.403686787 | 2.32180938 | 0.038331331 | 0.16617713 | -4.18624138 |
| DTX3 | 2.387386146 | 5.755259926 | 2.290895444 | 0.040549963 | 0.172297524 | -4.238394521 |
| SPR | -2.968329201 | 4.559103693 | -2.23332068 | 0.045011991 | 0.183349327 | -4.334805083 |
| LOC643986 | 2.016198239 | 4.48629524 | 2.225504134 | 0.045652559 | 0.184832193 | -4.347818863 |
| FLJ90650 | -2.399983198 | 4.500131749 | -2.213748542 | 0.046632217 | 0.18723561 | -4.367355814 |
| LOC286444 | 2.20230795 | 11.2427687 | 2.213127288 | 0.046684539 | 0.187377263 | -4.368387117 |
| EVPL | -2.581949944 | 6.380336891 | -2.212260696 | 0.046757617 | 0.187551152 | -4.369825488 |
| LOC647474 | 2.03218301 | 5.529567727 | 2.209760614 | 0.046969049 | 0.187742171 | -4.373973835 |
| DSP | -3.678476296 | 7.172132566 | -2.197392465 | 0.048028399 | 0.189880177 | -4.394467575 |
