# Supplementary material for: Identification of Diagnostic Markers in Infantile Hemangiomas
Source: J Oncol. 2022 Dec 1;2022:9395876. doi: 10.1155/2022/9395876 (PMC9731762; doi:10.1155/2022/9395876)
Supplement: Supplementary Materials — Table S1: DEGs of IHs in the 6-month-old compared to normal samples. Table S2: DEGs of IHs in the 12-month-old compared to normal samples. Table S3: DEGs of IHs in the 24-month-old compared to normal samples. Table S4: common up- and down-regulated genes among the 6-, 12-, and 24-month-old IHs samples. Table S5: GO and KEGG analysis of candidate genes. Table S6: the top 20 significant genes listed by the SVM-RFE algorithm ranked in 127 candidate genes for characteristics. Table S7: GO items relevant to diagnostic genes. Table S8: all functional annotation enrichment analysis results of the identified diagnostic genes. Table S9: all potential compounds are associated with the identified diagnostic genes. Table S10: potential compounds are associated with the major transcription factors. [file 9395876.f1.zip › Supplementary Table S10.pdf]

Table S10. Potential compounds associated with the major transcription factors

Gene Symbol	Chemical Name	Interaction Actions
AR	1-Methyl-4-phenylpyridinium	increases^expression
AR	4-(5-benzo(1,3)dioxol-5-yl-4-pyridin-2-yl-1H-imidazol-2-yl)benzamide	decreases^expression
AR	abrine	decreases^expression
AR	Aflatoxin B1	affects^expression
AR	Antirheumatic Agents	increases^expression
AR	Benzo(a)pyrene	decreases^expression
AR	bisphenol A	decreases^expression
AR	butyraldehyde	decreases^expression
AR	Cadmium Chloride	increases^expression
AR	Calcitriol	decreases^expression
AR	Cyclosporine	decreases^expression
AR	Diethylhexyl Phthalate	decreases^expression
AR	Dihydrotestosterone	increases^expression
AR	dorsomorphin	decreases^expression
AR	Doxorubicin	decreases^expression
AR	Estradiol	increases^expression
AR	Methapyrilene	increases^methylation
AR	Metribolone	increases^expression
AR	Nickel	decreases^expression
AR	Particulate Matter	decreases^expression
AR	Progesterone	decreases^expression
AR	Silicon Dioxide	decreases^expression
AR	sodium arsenite	increases^expression

AR	sulforaphane	decreases^expression
AR	Sunitinib	decreases^expression
AR	Temozolomide	increases^expression
AR	Testosterone	increases^expression
AR	Tetrachlorodibenzodioxin	increases^expression
AR	Tobacco Smoke Pollution	decreases^expression
AR	Tretinoin	decreases^expression
AR	trichostatin A	decreases^expression
AR	Troglitazone	decreases^expression
AR	Valproic Acid	decreases^expression
AR	Vorinostat	decreases^expression
BMI1	abrine	increases^expression
BMI1	Arsenic	affects^methylation
BMI1	bisphenol A	decreases^expression
BMI1	Estradiol	increases^expression
BMI1	Particulate Matter	decreases^expression
BMI1	Plant Extracts	increases^expression
BMI1	Silicon Dioxide	affects^expression
BMI1	Smoke	increases^expression
BMI1	sodium arsenite	increases^expression
BMI1	Sunitinib	increases^expression
BMI1	Temozolomide	decreases^expression
BMI1	Tretinoin	increases^expression
BMI1	trichostatin A	increases^expression
BMI1	Valproic Acid	decreases^methylation
EGR1	1-Methyl-4-phenylpyridinium	decreases^expression

EGR1	4-(5-benzo(1,3)dioxol-5-yl-4-pyridin-2-yl-1H-imidazol-2-yl)benzamide	increases^expression
EGR1	4-chloro-N-((4-(1,1-dimethylethyl)phenyl)methyl)-3-ethyl-1-methyl-1H-pyrazole-5-carboxamide	increases^expression
EGR1	7,8-Dihydro-7,8-dihydroxybenzo(a)pyrene 9,10-oxide	decreases^expression
EGR1	abrine	increases^expression
EGR1	Aflatoxin B1	affects^expression
EGR1	Amiodarone	increases^expression
EGR1	Antimycin A	increases^expression
EGR1	Antirheumatic Agents	decreases^expression
EGR1	Arachidonic Acid	increases^expression
EGR1	Arsenic	affects^expression
EGR1	Asbestos, Crocidolite	affects^expression
EGR1	Benzo(a)pyrene	decreases^expression
EGR1	beta-methylcholine	affects^expression
EGR1	bisphenol A	affects^expression
EGR1	butyraldehyde	increases^expression
EGR1	Cadmium Chloride	decreases^expression
EGR1	Calcitriol	increases^expression
EGR1	Cyclosporine	increases^expression
EGR1	Diethylhexyl Phthalate	decreases^expression
EGR1	dorsomorphin	increases^expression
EGR1	Doxorubicin	affects^expression
EGR1	entinostat	decreases^expression
EGR1	Estradiol	affects^expression
EGR1	incobotulinumtoxinA	decreases^expression
EGR1	licochalcone B	increases^expression
EGR1	Particulate Matter	increases^expression

EGR1	p-Chloromercuribenzoic Acid	increases^expression
EGR1	pentabromodiphenyl ether	increases^expression
EGR1	Progesterone	affects^expression
EGR1	Silicon Dioxide	decreases^expression
EGR1	Smoke	increases^expression
EGR1	sodium arsenite	increases^expression
EGR1	Sunitinib	decreases^expression
EGR1	Testosterone	increases^expression
EGR1	Tetrachlorodibenzodioxin	affects^expression
EGR1	thifluzamide	increases^expression
EGR1	Tobacco Smoke Pollution	decreases^expression
EGR1	Tretinoin	increases^expression
EGR1	triacsin C	increases^expression
EGR1	trichostatin A	increases^expression
EGR1	Troglitazone	increases^expression
EGR1	Valproic Acid	affects^expression
EGR1	Vorinostat	increases^expression
NANOG	4-(5-benzo(1,3)dioxol-5-yl-4-pyridin-2-yl-1H-imidazol-2-yl)benzamide	decreases^expression
NANOG	Benzo(a)pyrene	affects^methylation
NANOG	bisphenol A	increases^expression
NANOG	butyraldehyde	decreases^expression
NANOG	Diethylhexyl Phthalate	decreases^expression
NANOG	dorsomorphin	increases^expression
NANOG	entinostat	increases^expression
NANOG	Estradiol	increases^expression
NANOG	Panobinostat	increases^expression

NANOG	Particulate Matter	decreases^expression
NANOG	sodium arsenite	decreases^expression
NANOG	Sunitinib	decreases^expression
NANOG	Testosterone	increases^expression
NANOG	Tetrachlorodibenzodioxin	increases^expression
NANOG	Tretinoin	decreases^expression
NANOG	trichostatin A	increases^expression
NANOG	Valproic Acid	increases^expression
NANOG	Vorinostat	increases^expression
SETDB1	4-(5-benzo(1,3)dioxol-5-yl-4-pyridin-2-yl-1H-imidazol-2-yl)benzamide	decreases^expression
SETDB1	7,8-Dihydro-7,8-dihydroxybenzo(a)pyrene 9,10-oxide	decreases^expression
SETDB1	abrine	increases^expression
SETDB1	aristolochic acid I	decreases^expression
SETDB1	dorsomorphin	decreases^expression
SETDB1	Doxorubicin	decreases^expression
SETDB1	Gallic Acid	decreases^expression
SETDB1	Sunitinib	increases^expression
SETDB1	Tobacco Smoke Pollution	increases^expression
SETDB1	Valproic Acid	decreases^expression
SMAD4	1-Methyl-4-phenylpyridinium	increases^expression
SMAD4	4-(5-benzo(1,3)dioxol-5-yl-4-pyridin-2-yl-1H-imidazol-2-yl)benzamide	decreases^expression
SMAD4	7,8-Dihydro-7,8-dihydroxybenzo(a)pyrene 9,10-oxide	decreases^expression
SMAD4	Aflatoxin B1	decreases^methylation
SMAD4	Arsenic	affects^methylation
SMAD4	bisphenol A	decreases^expression
SMAD4	Carbamazepine	affects^expression

SMAD4	Cyclosporine	increases^expression
SMAD4	Dihydrotestosterone	decreases^expression
SMAD4	dorsomorphin	decreases^expression
SMAD4	Doxorubicin	increases^expression
SMAD4	Gallic Acid	increases^expression
SMAD4	Particulate Matter	affects^expression
SMAD4	Phenylmercuric Acetate	decreases^expression
SMAD4	sodium arsenite	increases^expression
SMAD4	Tetrachlorodibenzodioxin	affects^expression
SMAD4	Tretinoin	increases^expression
SMAD4	trichostatin A	increases^expression
SMAD4	Valproic Acid	decreases^expression
SMAD4	Vorinostat	decreases^expression
SOX2	4-(5-benzo(1,3)dioxol-5-yl-4-pyridin-2-yl-1H-imidazol-2-yl)benzamide	decreases^expression
SOX2	abrine	decreases^expression
SOX2	Aflatoxin B1	decreases^methylation
SOX2	Antirheumatic Agents	increases^expression
SOX2	Arsenic	decreases^expression
SOX2	Benzo(a)pyrene	decreases^expression
SOX2	bisphenol A	decreases^expression
SOX2	butyraldehyde	decreases^expression
SOX2	Diethylhexyl Phthalate	decreases^expression
SOX2	dorsomorphin	decreases^expression
SOX2	erucylphospho-N,N,N-trimethylpropylammonium	decreases^expression
SOX2	Estradiol	decreases^expression
SOX2	Fonofos	increases^methylation

SOX2	mercuric bromide	decreases^expression
SOX2	Panobinostat	decreases^expression
SOX2	Parathion	increases^methylation
SOX2	Particulate Matter	increases^expression
SOX2	p-Chloromercuribenzoic Acid	decreases^expression
SOX2	Phenylmercuric Acetate	decreases^expression
SOX2	Silicon Dioxide	decreases^expression
SOX2	sodium arsenate	decreases^expression
SOX2	sodium arsenite	increases^expression
SOX2	Temozolomide	increases^expression
SOX2	terbufos	increases^methylation
SOX2	Tetrachlorodibenzodioxin	increases^expression
SOX2	Tobacco Smoke Pollution	affects^expression
SOX2	Tretinoin	decreases^expression
SOX2	trichostatin A	decreases^expression
SOX2	Valproic Acid	decreases^expression
SOX2	Vorinostat	decreases^expression
SUZ12	7,8-Dihydro-7,8-dihydroxybenzo(a)pyrene 9,10-oxide	decreases^expression
SUZ12	aristolochic acid I	decreases^expression
SUZ12	bisphenol A	affects^expression
SUZ12	kojic acid	decreases^expression
SUZ12	Sunitinib	increases^expression
SUZ12	Testosterone	decreases^expression
SUZ12	trichostatin A	decreases^expression
SUZ12	Valproic Acid	decreases^expression
