# Supplementary material for: Identification of Diagnostic Markers in Infantile Hemangiomas
Source: J Oncol. 2022 Dec 1;2022:9395876. doi: 10.1155/2022/9395876 (PMC9731762; doi:10.1155/2022/9395876)
Supplement: Supplementary Materials — Table S1: DEGs of IHs in the 6-month-old compared to normal samples. Table S2: DEGs of IHs in the 12-month-old compared to normal samples. Table S3: DEGs of IHs in the 24-month-old compared to normal samples. Table S4: common up- and down-regulated genes among the 6-, 12-, and 24-month-old IHs samples. Table S5: GO and KEGG analysis of candidate genes. Table S6: the top 20 significant genes listed by the SVM-RFE algorithm ranked in 127 candidate genes for characteristics. Table S7: GO items relevant to diagnostic genes. Table S8: all functional annotation enrichment analysis results of the identified diagnostic genes. Table S9: all potential compounds are associated with the identified diagnostic genes. Table S10: potential compounds are associated with the major transcription factors. [file 9395876.f1.zip › Supplementary Table S2.pdf]

Table S2. DEGs of IHs in the 12-month-old compared to normal samples

Symbol	logFC	AveExpr	t	P.Value	adj.P.Val	B
WARS	3.99168006	10.79505133	12.16891959	3.29E-08	0.000303959	8.888684844
CETP	3.983209497	7.101994492	11.39769589	6.92E-08	0.000303959	8.278104365
FAM184A	3.747283743	7.700782661	11.26273689	7.91E-08	0.000303959	8.165925695
WSCD1	3.443401635	6.724600089	11.06596379	9.64E-08	0.000303959	7.999385455
KCNJ2	3.267794577	7.977981374	10.29006902	2.17E-07	0.000547059	7.306555937
ISL1	3.685436449	6.434743476	10.08080464	2.72E-07	0.000547664	7.109311613
HS3ST3A1	4.057288087	7.687845622	9.980357362	3.04E-07	0.000547664	7.012993814
APLN	6.106514518	7.797309621	9.788701755	3.76E-07	0.000593029	6.82620265
HECW2	3.318839583	7.728649276	9.424673486	5.69E-07	0.000797488	6.460215608
TDO2	3.403367887	6.826646331	9.292205964	6.64E-07	0.000823126	6.323288353
TMEM2	3.661078399	9.06178404	9.225071177	7.18E-07	0.000823126	6.253111959
EXOC6	3.047751634	8.613351404	8.956194641	9.88E-07	0.000985904	5.966683042
FAM69B	3.212558669	9.87845947	8.932561076	1.02E-06	0.000985904	5.941089654
FKBP1A	2.798916818	9.164158364	8.671220111	1.40E-06	0.001176602	5.65348293
C20orf46	4.024586233	6.866322748	8.669500862	1.40E-06	0.001176602	5.651562672
HEY1	3.003253245	7.519763674	8.500528383	1.73E-06	0.001359905	5.461003963
IDO2	4.678281043	7.499782255	8.291046102	2.25E-06	0.001666235	5.219653812
ASS1	-3.122781943	8.203167401	-8.142026756	2.72E-06	0.001841646	5.044453432
STX3	2.548277796	8.292200471	8.125932448	2.78E-06	0.001841646	5.02535441
PDGFB	3.73718929	7.579242446	8.059435784	3.02E-06	0.001854424	4.946074001
RAPGEF5	2.616802556	9.577509028	8.043230252	3.09E-06	0.001854424	4.926662588
ICAM2	3.630711315	10.04698205	7.976940101	3.37E-06	0.001929733	4.846887536
CMTM8	2.154414331	9.133216961	7.921533563	3.62E-06	0.001984707	4.779750391

ZNF697	2.555088634	5.990255043	7.792269168	4.29E-06	0.002190747	4.621476325
KCNMB3	2.895235723	6.769680594	7.765546823	4.45E-06	0.002190747	4.588468075
MRI1	2.169899935	8.530994875	7.754108044	4.52E-06	0.002190747	4.574308125
CRMP1	4.666469825	6.370362863	7.476538452	6.57E-06	0.003067441	4.225056518
THSD1	2.220257478	6.244875711	7.40819045	7.21E-06	0.003248149	4.137372595
MAP4K2	2.271702088	10.11564485	7.331793997	8.02E-06	0.003471089	4.038564507
MGC16121	5.507933846	7.79166119	7.31008214	8.26E-06	0.003471089	4.010328613
ADA	2.355896053	7.383027973	7.140000262	1.05E-05	0.004200347	3.786752815
DYSF	3.956427163	9.213506953	7.127430479	1.07E-05	0.004200347	3.770060593
PRKAB1	2.218585296	8.254961498	7.078874504	1.14E-05	0.004235427	3.705360093
ENPEP	3.974840758	8.470112615	7.078606084	1.14E-05	0.004235427	3.705001451
COL18A1	3.162084359	11.20568051	7.016510958	1.25E-05	0.004493432	3.621746891
AFAP1L1	2.56335121	7.8169909	6.977059645	1.32E-05	0.004602814	3.568553229
SLC38A1	-2.989171261	6.034706532	-6.943919317	1.38E-05	0.004602814	3.523688739
CCND1	2.141626417	12.52437356	6.939680779	1.39E-05	0.004602814	3.517938833
LOC100130623	2.585905411	6.508841872	6.923957705	1.42E-05	0.004602814	3.496585677
PGF	3.600078727	7.119192265	6.899433144	1.47E-05	0.004619584	3.463205179
RGS5	4.245813637	10.62796004	6.886637378	1.50E-05	0.004619584	3.445752837
SCN4B	3.676076012	8.758043763	6.808778492	1.68E-05	0.004908232	3.339026899
STARD8	3.484744372	7.970328352	6.776528913	1.76E-05	0.004908232	3.294551522
TBX3	2.86574883	8.25115156	6.750207561	1.83E-05	0.004908232	3.258134658
GPX7	2.336452028	8.725458327	6.734911758	1.87E-05	0.004908232	3.236923743
LOC644242	3.50843118	5.296463885	6.7129422	1.93E-05	0.004908232	3.206395866
RAMP2	2.429592473	8.061311661	6.712803747	1.93E-05	0.004908232	3.206203244
NOX4	4.199023718	7.721719706	6.707421506	1.95E-05	0.004908232	3.198712993
TUSC3	3.259550184	8.265309672	6.694646415	1.99E-05	0.004908232	3.180916706

EGFLAM	2.253626056	7.915155821	6.671035336	2.06E-05	0.004983468	3.147959771
SH2D3C	4.091616826	8.70237951	6.607976885	2.26E-05	0.005365176	3.059522972
ARID3B	2.043155525	7.37252832	6.578918972	2.35E-05	0.005371919	3.018565194
NRIP2	2.292163686	5.78708847	6.569905318	2.39E-05	0.005371919	3.005833878
MEG3	3.558441373	10.55703438	6.547180585	2.47E-05	0.005458603	2.973680904
RUVBL1	2.842951407	7.227611721	6.524497598	2.55E-05	0.005543478	2.941507695
OAS2	2.183934442	9.12434115	6.513614335	2.59E-05	0.005543478	2.926042884
PPM1F	2.385975365	11.16965252	6.489054907	2.69E-05	0.005561697	2.891077485
SLC6A9	2.731095681	8.249991457	6.434050922	2.92E-05	0.005917398	2.812429934
TIE1	3.287091002	7.868051987	6.426088006	2.96E-05	0.005917398	2.801005362
MYLIP	2.644386171	10.87361424	6.392505367	3.11E-05	0.006126585	2.752715534
DNAJC12	2.294176047	4.883440763	6.342022404	3.36E-05	0.006509869	2.679794932
C1orf54	3.078858205	10.04602458	6.327307227	3.43E-05	0.006516848	2.658464957
RASGRP3	2.635488031	8.286324633	6.321297941	3.46E-05	0.006516848	2.649744685
COL4A2	4.022346133	10.70375342	6.298439305	3.59E-05	0.006647572	2.616522462
ARID3A	2.877334603	8.545081276	6.286757173	3.65E-05	0.006668547	2.599512529
H19	4.891760743	12.05838077	6.274365484	3.72E-05	0.006698347	2.581446217
C5orf13	2.325926121	10.14553185	6.243124972	3.90E-05	0.00685931	2.535793355
ACVRL1	3.201727991	9.47431512	6.240303042	3.92E-05	0.00685931	2.531662082
TFPI2	8.017624277	6.537858236	6.172550085	4.35E-05	0.007169898	2.432099674
PECAM1	3.61396944	11.55034868	6.168157	4.38E-05	0.007169898	2.425619344
AVPI1	-2.377424585	9.056232111	-6.159154072	4.44E-05	0.007169898	2.412329527
COLEC11	2.401399994	6.927822263	6.139320363	4.57E-05	0.007200858	2.383007021
LOC158376	2.824453605	5.640328554	6.131546157	4.63E-05	0.007200858	2.371496735
ANKRD20A1	3.704951536	8.268939855	6.125254519	4.67E-05	0.007200858	2.362174588
GUCY1B3	2.469952618	6.818037142	6.113409158	4.76E-05	0.007200858	2.344606858

PCDH17	5.016991022	9.009860965	6.108273533	4.80E-05	0.007200858	2.336983453
DOCK6	2.220345483	8.140998984	6.097813536	4.88E-05	0.007232111	2.321443717
FCGR2B	2.633920049	6.749929213	6.058028549	5.19E-05	0.007494144	2.262181508
STEAP4	3.369400266	6.05327099	6.053867259	5.22E-05	0.007494144	2.255968712
GPC5	3.944067222	5.768580148	6.045613178	5.29E-05	0.007494144	2.243637377
CDH5	3.855209187	11.07150287	6.045177331	5.29E-05	0.007494144	2.242985938
BST2	3.327517343	9.919682351	6.014169426	5.55E-05	0.007623674	2.196563794
ANKS1A	2.280231836	10.16454281	6.011983992	5.57E-05	0.007623674	2.193286293
TRIB2	2.14522148	8.599628753	5.984668083	5.81E-05	0.007623674	2.152257528
COX4I2	5.979960719	7.161110796	5.975905372	5.89E-05	0.007623674	2.139071122
FLJ11235	2.215508294	6.854637933	5.959382366	6.05E-05	0.007701607	2.114174103
PCDHB17	2.116676685	6.532981413	5.933323005	6.30E-05	0.007853593	2.074820827
ANKRD47	2.458681887	7.544471728	5.927872197	6.35E-05	0.007853593	2.066575913
DCXR	-2.22170629	8.246901264	-5.907962039	6.56E-05	0.00796149	2.036420242
CHI3L1	-3.124540414	5.91452559	-5.906829718	6.57E-05	0.00796149	2.034703381
GCOM1	2.662916839	7.354326148	5.895836937	6.68E-05	0.008023252	2.018025347
CD34	2.450145316	9.695651288	5.86342307	7.03E-05	0.008209675	1.96873767
POPDC2	2.884056014	6.991718279	5.857562094	7.10E-05	0.008209675	1.959808091
TUBB2B	2.170529081	8.315659587	5.835141587	7.35E-05	0.008428609	1.925599438
KCNK6	-2.554027178	6.652946292	-5.829304347	7.42E-05	0.008430223	1.916680237
PAPSS2	2.99193931	8.602914522	5.798435558	7.79E-05	0.008696453	1.869424779
ZNF165	-2.207781585	4.145717523	-5.762261382	8.26E-05	0.008931711	1.813858284
LINGO1	2.799058793	7.576050521	5.761766893	8.26E-05	0.008931711	1.813097293
FLJ41603	-2.922953114	6.299709263	-5.757717534	8.32E-05	0.008931711	1.806864129
LXN	2.388428576	9.303804002	5.754443948	8.36E-05	0.008931711	1.801823239
IGFALS	2.969979197	4.778278223	5.742643513	8.52E-05	0.008953194	1.783638264

ALDH2	-2.227740613	9.483017842	-5.732928793	8.65E-05	0.008953194	1.768651175
Septin 4	2.354113922	7.693558327	5.732762258	8.65E-05	0.008953194	1.76839413
JAM2	3.077720464	9.796240686	5.732046084	8.66E-05	0.008953194	1.767288673
RSPO3	3.740238099	8.085361596	5.721823738	8.81E-05	0.009026652	1.751501175
ATP1B2	2.951141881	6.656321756	5.712488344	8.94E-05	0.009063878	1.737069273
BCL6B	2.510802258	8.312335303	5.70916694	8.99E-05	0.009063878	1.731931328
ROBO4	2.635304217	7.458140068	5.696408033	9.17E-05	0.009177546	1.712178358
PCSK5	3.046494374	9.076151887	5.68147711	9.39E-05	0.009325874	1.689030565
RASSF7	-2.995579013	6.916820673	-5.655247313	9.80E-05	0.009559722	1.648281889
IL18R1	3.458485222	7.288529377	5.632913482	0.000101576	0.009559722	1.613501438
IL32	2.372602808	7.410734221	5.628468878	0.000102306	0.009559722	1.606570623
PXDN	2.565264878	9.566388266	5.620611501	0.00010361	0.009559722	1.594310506
PMEPA1	2.730064605	9.377261651	5.614175743	0.000104691	0.009559722	1.584261452
RASIP1	2.373924854	10.1881948	5.608615241	0.000105635	0.009559722	1.575573889
HLX	3.256180085	6.99928562	5.606996197	0.000105912	0.009559722	1.573043441
ISG15	2.688192042	9.524619932	5.590570416	0.000108761	0.009559722	1.547348177
TYMS	2.524988887	8.022711697	5.590422848	0.000108787	0.009559722	1.547117142
ANKRD35	-2.247772126	7.130274959	-5.582807298	0.000110135	0.009559722	1.535189593
APLNR	4.269805806	10.76796622	5.576391657	0.000111285	0.009559722	1.525134384
RTN2	2.431241003	6.51958956	5.566604205	0.000113063	0.009630982	1.509782279
STXBP6	3.24830477	7.918349303	5.486532262	0.000128785	0.010752302	1.383629901
FAM162B	2.903261391	9.345598286	5.465407654	0.000133306	0.011056509	1.350183682
RCAN1	3.109813702	9.952747181	5.439247456	0.000139137	0.01128163	1.308669727
FUT11	2.355769545	5.312365017	5.437220575	0.0001396	0.01128163	1.305448864
FHOD1	2.917688101	8.403033867	5.413027152	0.000145251	0.011589753	1.266955144
EPS15L1	2.460641502	5.016631004	5.36485547	0.000157232	0.012235979	1.19004372

CA12	-4.257868153	6.804550493	-5.354045269	0.000160061	0.012304219	1.172735449
KHDRBS3	2.302610397	8.079447932	5.345548707	0.000162322	0.012327684	1.159119084
SLC1A5	-2.151222644	6.472106972	-5.335311962	0.000165091	0.012462876	1.142699382
IGF2	5.787359715	7.728706148	5.324362828	0.000168107	0.012552357	1.12511941
GJA4	3.001627826	9.092145058	5.323786834	0.000168267	0.012552357	1.124194089
ADAMTS9	2.882567991	8.440440518	5.310113401	0.00017212	0.012689557	1.102213262
CHST10	2.376639942	5.679880566	5.301782433	0.000174512	0.012791141	1.088806878
LOC100132091	2.926913772	6.255723685	5.287810402	0.000178604	0.013015345	1.066299215
COL15A1	2.304407025	10.74809004	5.278311181	0.000181443	0.013142925	1.050980006
KCNJ8	3.006952915	9.165615236	5.271583746	0.000183482	0.013142925	1.04012257
MYO1B	2.559571416	8.929856097	5.25725157	0.000187907	0.013186055	1.016969081
SCARB1	2.296345153	9.042850957	5.254017233	0.000188921	0.013186055	1.011739767
TCEAL7	2.554249307	6.656062041	5.250121248	0.00019015	0.013186055	1.005438604
LOC100129681	2.603643392	9.009915671	5.249460137	0.000190359	0.013186055	1.004369132
SIPA1L2	2.060894827	9.496316265	5.243172407	0.000192363	0.01318639	0.99419425
PLXND1	2.409567162	7.409813528	5.242882191	0.000192456	0.01318639	0.993724476
IRX5	-3.086843208	6.99227319	-5.233879149	0.000195365	0.013313359	0.979144917
GAP43	3.338922837	5.351222266	5.220374686	0.000199816	0.013363639	0.957252957
TPM4	2.365652781	8.848365619	5.218025339	0.000200601	0.013363639	0.953441661
GNG11	2.754180258	11.94404121	5.197627028	0.000207555	0.013557759	0.92031524
C9orf24	-2.409160528	4.342965595	-5.184881302	0.000212028	0.013778567	0.89958492
DHRS3	-2.112247162	8.309486424	-5.16516439	0.000219149	0.014095957	0.867468642
FCGBP	-4.467921715	7.446013016	-5.136242555	0.00023005	0.0145741	0.820254322
LYL1	2.84026783	9.508422743	5.130172723	0.00023241	0.01464995	0.81032972
EPAS1	2.841510363	12.33883227	5.118496848	0.00023702	0.014683693	0.791223544
PDE9A	2.294396955	6.401229271	5.114727152	0.000238529	0.014683693	0.78505059

MBP	-2.19145702	8.027273725	-5.091635152	0.000247994	0.015176971	0.747191323
ITM2C	2.036827828	10.53984689	5.086212463	0.000250273	0.015188098	0.738289478
FAM43A	2.332470581	9.220844516	5.085474098	0.000250585	0.015188098	0.73707705
C20orf107	-2.368769569	3.856081173	-5.07634138	0.000254478	0.015204786	0.722074117
FJX1	2.680221378	8.008398793	5.062276681	0.000260599	0.015377346	0.698945208
COL4A1	2.954594869	13.14584682	5.059813065	0.000261687	0.015377346	0.694890902
TMEM204	2.270520381	8.457280447	5.058552346	0.000262246	0.015377346	0.692815827
PDE1A	2.642828977	6.219681706	5.036076679	0.000272416	0.015753875	0.655783236
SEMA5B	3.618814026	6.069584743	5.029762247	0.000275347	0.015850668	0.645365882
CAPG	-2.219335965	4.970904472	-5.002110296	0.000288577	0.016461928	0.599678461
NID1	2.752826823	6.962533247	4.978202161	0.000300552	0.017050786	0.560087673
MFNG	3.018239387	8.838556466	4.976149268	0.000301604	0.017050786	0.556684341
LRRC1	-3.081255458	6.895412476	-4.951676072	0.00031445	0.017588754	0.516065575
CHN1	4.047428663	9.005987506	4.947754914	0.000316561	0.017588754	0.509549566
SERPINH1	2.811913389	10.38153148	4.947495435	0.000316701	0.017588754	0.509118298
MGC52282	5.435506503	4.485635993	4.943112137	0.000319079	0.017636218	0.501831565
JAM3	3.056459022	10.90546018	4.940777657	0.000320353	0.017636218	0.497949639
SLC31A2	-3.3982741	7.063614985	-4.933379079	0.000324427	0.017705849	0.485641673
SDC4	-3.094966193	6.530134389	-4.920378106	0.000331717	0.018003799	0.463994931
GRAP	2.231101715	8.370304543	4.918573657	0.000332743	0.018003799	0.460988611
C16orf30	2.37540992	8.433646235	4.913231521	0.000335797	0.018091448	0.45208558
SLC6A10P	-2.213478693	6.966164852	-4.885983725	0.000351842	0.018701191	0.406612508
ATP6V0E2	-2.816340974	7.926831991	-4.884112748	0.000352973	0.018701191	0.403486248
FGD1	2.291617424	6.665585731	4.883987954	0.000353049	0.018701191	0.403277707
C6orf188	2.65962759	5.373552615	4.873164136	0.000359667	0.018972034	0.385182074
TM7SF2	-2.76263656	7.162199986	-4.856569578	0.000370066	0.019278598	0.357406815

SLC7A1	-2.6183545	7.617531177	-4.847831424	0.000375668	0.01948989	0.342765793
NTN1	-2.48925647	4.984890218	-4.835595642	0.000383661	0.019742111	0.322246516
PKIG	2.055187599	6.75097634	4.832023112	0.000386029	0.019778246	0.316251495
PDGFC	-2.830321414	5.943614794	-4.806878911	0.000403128	0.020290528	0.274007276
ARAP3	2.648995396	8.755619209	4.805660918	0.000403976	0.020290528	0.271958735
TEK	2.642381135	9.410066403	4.780955009	0.000421588	0.020943166	0.230361836
LOC285016	4.80634068	7.903274001	4.780453552	0.000421953	0.020943166	0.229516675
GPER	3.350612113	8.965804105	4.768764348	0.000430571	0.021203963	0.209805818
SLC2A1	3.768936857	10.36504964	4.75703335	0.000439405	0.021306096	0.19000575
CCDC85A	2.350823359	7.489113318	4.739863419	0.000452678	0.021741501	0.160991923
SEC14L1	2.337382235	7.060004933	4.73979768	0.00045273	0.021741501	0.160880759
LRRC33	2.309616276	7.59124029	4.738742658	0.000453559	0.021741501	0.159096666
MDK	2.449465435	9.518578993	4.734701445	0.000456749	0.021811518	0.152261392
KLHL23	4.352869331	4.586864842	4.724488994	0.000464918	0.022117798	0.134978315
C13orf23	2.13766644	8.363913502	4.706820426	0.000479411	0.022636474	0.105043739
FAM150B	3.896607747	8.286987509	4.698932889	0.000486033	0.022778506	0.091666956
SYN2	2.450765362	5.638654773	4.684027066	0.000498808	0.023204717	0.066364935
GMFG	2.243542276	8.978612036	4.672488064	0.000508939	0.023395165	0.046757684
CKB	-2.314898121	7.846143621	-4.670144945	0.000511022	0.023395165	0.042774067
ESAM	3.079325996	10.28051318	4.646210935	0.000532816	0.023697585	0.002041578
SHMT1	-2.263430318	5.678897907	-4.64109506	0.000537599	0.023697585	-0.006674718
NOXA1	-2.152166553	4.405385757	-4.636422538	0.000542006	0.023703848	-0.014638633
FZD3	-2.286411919	5.389128406	-4.618680242	0.000559089	0.023974249	-0.044904785
LOC731486	-3.309219943	5.429974432	-4.611756409	0.000565906	0.024050558	-0.056727078
SLC7A8	-2.050319584	6.059225377	-4.611065366	0.000566591	0.024050558	-0.057907359
ACSL1	-4.099089297	7.5328579	-4.607120561	0.000570519	0.024136021	-0.064646154

IRS2	-2.09854354	6.982993756	-4.578930309	0.000599427	0.025189919	-0.112861062
SRGN	2.306037245	10.44198987	4.554952851	0.000625214	0.025842881	-0.1539506
IFIT2	2.294126946	8.866967933	4.544015974	0.000637358	0.026065588	-0.172717037
GTSF1	3.62779903	5.819329173	4.543790387	0.000637611	0.026065588	-0.173104276
HDHD3	-2.382202643	5.775318244	-4.541179269	0.000640548	0.026065588	-0.177586962
GBP4	2.352229155	8.189743157	4.538521559	0.000643551	0.026065588	-0.182150516
PCDHB2	2.490888254	5.559130016	4.53417779	0.000648491	0.026065588	-0.189611111
HRC	3.378022732	7.418293975	4.533386233	0.000649396	0.026065588	-0.190970895
MUC1	-3.716848744	6.458260761	-4.531742918	0.000651278	0.026065588	-0.193794135
AACS	-2.949198678	6.228823782	-4.529904611	0.00065339	0.026067364	-0.196952773
LDB2	2.219217242	11.37159372	4.52101166	0.000663708	0.026128587	-0.212238897
ELMO1	2.060764602	6.392644381	4.519181905	0.000665852	0.026128587	-0.215385293
CYGB	2.647097223	10.0352336	4.518276847	0.000666915	0.026128587	-0.21694176
SH2B3	2.999791053	9.53746306	4.517898991	0.00066736	0.026128587	-0.217591605
RAPGEF3	2.547112245	6.592616556	4.508403947	0.000678628	0.026352192	-0.233927227
IKBIP	2.032101039	5.584390309	4.49593459	0.000693728	0.026583062	-0.255396935
MPDZ	2.148121603	8.910841915	4.486241139	0.000705707	0.026960155	-0.272100345
RBM47	-2.981330894	6.476551623	-4.473208754	0.000722153	0.027071562	-0.294575453
ATP9A	-2.364041793	9.346174588	-4.471730189	0.000724044	0.027071562	-0.297126634
SLC44A4	2.269192199	8.190900173	4.460622119	0.000738414	0.027379973	-0.316301506
CHST14	2.13573131	7.077590563	4.456499859	0.000743823	0.027499625	-0.323421208
Gcom1	2.895325623	6.535120619	4.448741534	0.000754113	0.027717506	-0.336826458
GUCY1A2	2.527210777	4.728926361	4.421573358	0.00079133	0.028607406	-0.383826101
LRRC8E	-2.228141595	4.470776246	-4.421139157	0.00079194	0.028607406	-0.384577963
NR2F2	2.63292287	8.911384867	4.403127107	0.000817682	0.029202595	-0.415787326
ALCAM	-2.923496194	6.650407251	-4.401454743	0.000820116	0.029206775	-0.418686965

SERPINE1	2.027771957	6.9123884	4.377518613	0.000855786	0.029766072	-0.460224675
VASH1	2.346376488	7.993520625	4.376456248	0.000857405	0.029766072	-0.462069805
LOC644423	2.137056967	6.064424974	4.371234625	0.000865414	0.029766072	-0.471140694
CYTSB	2.058943618	4.888885466	4.366726131	0.000872392	0.029766072	-0.478975289
SEL1L3	2.105404576	8.322716655	4.364435676	0.000875959	0.029766072	-0.482956406
ZYX	2.05699455	9.902297553	4.354615376	0.000891425	0.030048653	-0.500032242
HYAL2	2.54126121	9.072603658	4.352074227	0.000895473	0.030069086	-0.504452672
TNFRSF10A	2.909248873	5.98850135	4.351243018	0.000896801	0.030069086	-0.505898754
CHPT1	-2.409096368	7.347665448	-4.346632178	0.000904207	0.03018939	-0.513921817
LOC645166	2.006566877	7.930082474	4.346029855	0.000905179	0.03018939	-0.514970066
KCNMB4	2.151835479	7.083948246	4.326938301	0.000936558	0.030832638	-0.54821725
NEBL	-2.968158023	5.808450988	-4.32685677	0.000936694	0.030832638	-0.548359323
NDUFA4L2	4.331081466	9.210934182	4.316060993	0.000954938	0.031237496	-0.567178058
SULT1A2	2.185441693	5.627938894	4.315189315	0.000956427	0.031237496	-0.5686981
TMEM108	4.97807297	4.459268075	4.311771675	0.000962288	0.03126693	-0.574658643
VAMP5	2.250141437	9.879125383	4.303373959	0.00097685	0.031658463	-0.589310229
STARD3NL	2.804868732	6.678930704	4.297342838	0.000987448	0.031838253	-0.59983763
KCNJ13	-2.047493924	4.228142732	-4.287891953	0.001004296	0.032078702	-0.616342364
PTP4A3	2.123702887	6.098933023	4.287063479	0.001005787	0.032078702	-0.617789656
SLC38A11	3.407495314	5.529497879	4.286790979	0.001006278	0.032078702	-0.618265712
ZNF792	2.245158899	7.007284994	4.286042546	0.001007628	0.032078702	-0.619573264
MAPK13	-4.127017985	6.25909326	-4.275070066	0.001027632	0.032464566	-0.638749811
LOC100134361	-2.644805723	5.107271768	-4.260854194	0.001054162	0.032783611	-0.663614334
CD4	2.561874739	6.003038892	4.259867816	0.001056029	0.032783611	-0.665340387
GPT2	-2.972481728	7.575845304	-4.250457652	0.001074013	0.032944233	-0.681812425
PPL	-2.708801868	7.37139491	-4.247550177	0.001079633	0.033009278	-0.686903744

EDNRA	2.602896307	9.887313117	4.246397383	0.00108187	0.033009278	-0.688922668
TM4SF18	2.330096578	8.430731592	4.245308042	0.001083988	0.033009278	-0.690830596
EFNB1	2.582898164	7.525831068	4.240487782	0.001093413	0.03313619	-0.699274564
N6AMT1	2.060833415	4.984740982	4.236742078	0.001100795	0.033279905	-0.705837871
ARHGAP4	2.578613617	7.630662417	4.216254211	0.001142095	0.034038762	-0.74176345
CEACAM1	3.889142172	7.732590799	4.21298819	0.001148826	0.034158605	-0.747494513
RPESP	-2.538005741	6.564339251	-4.199780263	0.001176466	0.034664392	-0.770682503
SH3RF2	-2.792294561	5.309156843	-4.19466561	0.001187353	0.034730777	-0.779666702
RASL12	2.239380338	10.0713052	4.152219743	0.001281827	0.036537766	-0.854328411
GPR176	2.448882611	5.967874196	4.151321329	0.001283908	0.036537766	-0.85591067
LOC100133999	2.499441413	7.175705571	4.140984095	0.001308112	0.036641226	-0.874122047
OR51E1	2.288965258	5.234669082	4.138798254	0.00131329	0.036641226	-0.877974254
C5orf39	2.287085055	7.3217465	4.121426696	0.001355203	0.037438265	-0.908605587
LOC644632	2.073295293	5.306161573	4.119467355	0.001360016	0.037438265	-0.912062346
TMEM45A	-3.3966558	6.204603585	-4.118160165	0.001363237	0.037438265	-0.914368756
CD93	3.276980991	11.0961421	4.110496199	0.001382281	0.037534103	-0.927894403
TMEM44	2.659009124	8.155540891	4.109228961	0.001385456	0.037534103	-0.930131414
PVRL2	2.4678991	8.018792225	4.08010915	0.001460533	0.038749585	-0.981577713
RCBTB1	2.001055071	4.198526049	4.077704147	0.001466918	0.038749585	-0.985830229
MT1X	-3.036609324	9.583715332	-4.056887346	0.001523406	0.039517662	-1.022660787
TXNDC5	2.072938599	9.302440493	4.050013758	0.001542547	0.039888956	-1.034830751
GPR116	2.512296952	11.03983003	4.046064732	0.001553656	0.039888956	-1.041824601
SOX15	-2.590907952	6.448216357	-4.044178862	0.001558991	0.039888956	-1.045165032
FBP1	-3.91022336	5.023901545	-4.043869307	0.001559868	0.039888956	-1.045713377
C10orf11	4.382817462	5.61294929	4.037290131	0.001578636	0.040145916	-1.057369727
TGFB1I1	2.115366695	9.567150785	4.036999924	0.00157947	0.040145916	-1.057883976

USHBP1	2.721784551	5.639742411	4.025412055	0.001613113	0.040591849	-1.078423945
DBNDD1	-2.269307799	6.257205178	-4.016072608	0.001640766	0.040960662	-1.094987138
PLA2G4C	2.506320222	8.613854747	4.010160445	0.001658523	0.041159445	-1.105476106
KIAA1522	-2.115075904	6.381714301	-4.006585824	0.001669355	0.041265806	-1.111819438
APOLD1	2.253091382	9.290994664	4.005479345	0.001672723	0.041268138	-1.11378316
LAMC3	2.086029788	8.486966728	3.999704125	0.001690415	0.041398684	-1.124034443
MTUS1	3.007378903	7.671552202	3.994324309	0.001707069	0.041707396	-1.133586444
CDK6	2.26629384	9.001251583	3.988441345	0.001725474	0.042002947	-1.144034626
NRGN	2.144036431	7.084999125	3.98202264	0.001745788	0.042163133	-1.155437636
FAM108C1	-2.151216055	7.25505878	-3.974009923	0.001771494	0.042539478	-1.169677317
CDKN1C	2.459240355	8.827471949	3.964261894	0.001803293	0.042894556	-1.187008094
G3BP1	2.117750943	5.700827135	3.958331084	0.001822927	0.043194995	-1.197556172
PROCR	2.369260536	8.451792906	3.923203277	0.001943814	0.045295624	-1.260090057
RDH10	-2.458605973	5.926814904	-3.922210266	0.001947349	0.045295624	-1.26185922
FAM13C1	3.481125666	5.320308249	3.919840324	0.001955812	0.045408692	-1.266081859
LOC441019	2.180946573	5.836653023	3.909807163	0.00199206	0.045803229	-1.283963245
LOC646332	3.958118953	4.685513934	3.907395999	0.002000874	0.045803229	-1.288261647
FLJ35776	2.604557949	4.476486815	3.896659114	0.002040611	0.046269759	-1.307407765
LOC645993	3.734091378	4.471911892	3.886528021	0.00207885	0.046920221	-1.325481604
LYSMD1	2.036168294	4.600522083	3.873015949	0.002130999	0.047731155	-1.349598956
ECSCR	2.365223201	10.22540389	3.871198198	0.002138116	0.047792968	-1.352844434
GNAI2	2.047056778	10.8977921	3.845070068	0.002243161	0.049439738	-1.39952075
LOC652377	3.110341752	6.38570607	3.843553728	0.002249418	0.049491111	-1.40223108
LOC100132288	2.376795054	5.989635025	3.838613862	0.002269924	0.049585011	-1.411061784
LOC401720	2.102997422	6.263085239	3.831712462	0.002298896	0.049585011	-1.423401826
PDLIM7	2.411393566	5.452977207	3.831334063	0.002300495	0.049585011	-1.424078515

ZCCHC3	2.289465613	6.224472826	3.829454685	0.002308456	0.049585011	-1.427439549
HEYL	3.526408394	9.563855967	3.828650092	0.002311873	0.049585011	-1.428878537
LOC375295	2.225681918	8.244967156	3.828496637	0.002312525	0.049585011	-1.42915299
LOC728765	-2.752202441	3.369902013	-3.822687288	0.002337356	0.049607828	-1.439544178
TMEM154	-2.5038911	5.415544941	-3.810310172	0.002391177	0.050225217	-1.461690675
PRSS35	2.414455521	6.87430885	3.800427483	0.002435063	0.050359866	-1.479381118
NES	2.397342728	8.854941589	3.794824134	0.002460312	0.050580759	-1.489414168
ALDH1L1	-3.363607912	5.402485791	-3.791486992	0.002475476	0.050580759	-1.495390429
MAP7	-2.335625769	6.537990174	-3.782857434	0.002515136	0.051307966	-1.510847789
C12orf35	2.417012769	7.893480351	3.776441024	0.002545047	0.051667318	-1.522343957
ZNF185	-2.690383004	6.410960426	-3.772014571	0.002565893	0.051762651	-1.530276241
BCAR1	2.210877587	7.247660367	3.766728178	0.002591019	0.052014294	-1.539751126
PPAP2C	-2.130767884	5.023907861	-3.763057596	0.002608613	0.052284238	-1.546330963
CRB3	-2.70741754	4.920698055	-3.756203689	0.002641794	0.052781457	-1.558619357
FOXC1	-2.446759743	10.35324682	-3.745735491	0.002693309	0.053344876	-1.577393193
LOC388564	-2.832612338	6.502437124	-3.744979219	0.002697071	0.053344876	-1.578749751
SFRP1	-2.515182946	8.8864514	-3.744468476	0.002699614	0.053344876	-1.579665912
TSPAN33	-2.181927301	6.837479419	-3.733868313	0.002752954	0.054228902	-1.59868367
KCNAB1	2.862349112	9.360293215	3.717703827	0.002836381	0.055460315	-1.627696578
MGC61598	2.45958591	9.530578227	3.715178409	0.002849646	0.05547443	-1.632230638
ABHD12B	-2.046433465	4.205380889	-3.700401751	0.002928548	0.056534434	-1.658767079
C8orf4	2.819059522	9.427780047	3.679657668	0.003043101	0.057778151	-1.696039228
THY1	3.829960002	10.5397488	3.668737381	0.003105235	0.058516745	-1.71566903
ARHGEF17	2.548776478	8.058493938	3.653799157	0.003192337	0.059046955	-1.742530569
S100A9	-3.828007721	6.446674807	-3.65356004	0.003193751	0.059046955	-1.742960629
CLDN8	-3.026435438	5.57384898	-3.649142338	0.003219997	0.059348681	-1.750906476

ADCY4	3.039890133	9.371478339	3.646014339	0.003238713	0.059519619	-1.756533153
ALDOC	-2.531114193	7.97148481	-3.643823388	0.003251889	0.059588033	-1.760474522
RNF150	-2.095939086	7.83322178	-3.638240419	0.003285712	0.0599728	-1.770518867
RASL11A	2.027388999	6.22738262	3.624950606	0.003367676	0.060393023	-1.794434122
SPINT2	-4.646376743	7.718319679	-3.619818237	0.003399886	0.060560729	-1.80367193
LIX1L	2.218174369	6.670274265	3.618914534	0.00340559	0.060560729	-1.805298628
TPX2	2.583667212	6.293106594	3.618826656	0.003406145	0.060560729	-1.805456815
TCF4	2.10501117	9.943897008	3.616359438	0.00342177	0.060560729	-1.809898079
ALDH1A1	-2.23885978	8.655354841	-3.612815975	0.003444338	0.060719872	-1.816277143
NDC80	3.539041855	5.506365228	3.610932383	0.003456397	0.060719872	-1.819668257
MPRIP	2.235406739	9.43631327	3.609080085	0.003468297	0.060719872	-1.823003172
MYCN	-2.447288734	6.018594449	-3.60807234	0.003474789	0.060719872	-1.824817594
CES1	-3.284083023	6.183999756	-3.598686676	0.00353585	0.061231395	-1.841718202
CCR10	2.051066323	5.185781144	3.584883334	0.003627645	0.062053888	-1.866579803
LOC650803	-2.99373622	5.569050881	-3.582483905	0.003643847	0.062162361	-1.870902208
SMC4	2.019717449	8.366105436	3.572617227	0.003711254	0.0629346	-1.888678524
PLAC8	3.586927535	4.723731112	3.569959431	0.003729628	0.062945686	-1.893467534
IFIT3	2.329470371	7.048160727	3.569363388	0.003733761	0.062945686	-1.894541561
TMEM136	2.066361492	5.889252584	3.565959814	0.003757453	0.063084701	-1.900674794
NEURL1B	2.071592762	9.482713375	3.553951445	0.003842271	0.063652445	-1.922316953
EVI1	2.044911723	8.552422745	3.552689215	0.003851299	0.063718276	-1.924592089
DENND2D	-2.187900209	5.193039816	-3.548958663	0.003878108	0.064077728	-1.931316609
SETD4	2.499094959	4.452216382	3.518638585	0.004103167	0.06589634	-1.98598575
TBX15	3.197580134	7.531753842	3.505605362	0.004203952	0.066749656	-2.009493391
C10orf72	2.10818117	5.941128015	3.501622345	0.004235252	0.067077672	-2.016678305
CDH6	2.801762234	7.961572916	3.48853361	0.004339787	0.068004596	-2.040291578

LOC100134134	2.110303924	11.77848079	3.474023374	0.004458744	0.069201278	-2.066473831
ANTXR2	2.690982153	9.542714388	3.472912474	0.004467987	0.069201278	-2.068478518
SEMA4D	-2.513239716	6.290922785	-3.472008293	0.004475524	0.069201278	-2.070110186
SCG2	4.32782098	5.390849663	3.462399192	0.004556428	0.07003592	-2.087451567
TNFRSF19	-2.804908906	8.191385287	-3.462292728	0.004557332	0.07003592	-2.087643711
FILIP1	2.050059134	6.119803784	3.46187096	0.004560918	0.07003592	-2.088404908
JAG1	2.615980319	9.572260136	3.453916147	0.004629084	0.070652373	-2.102762178
FLJ90650	-2.878199226	4.239286642	-3.450376572	0.004659747	0.07081598	-2.10915094
SIRPA	-2.420814596	7.027712139	-3.431705606	0.004824936	0.072500561	-2.142854354
CEBPA	-2.839140009	7.878148811	-3.42812022	0.004857331	0.072641017	-2.149326985
PNPLA7	-2.018453519	7.86326363	-3.427298938	0.004864783	0.072666255	-2.150809654
FAM46C	-2.538553447	6.758246264	-3.42100078	0.004922314	0.07326518	-2.162180058
C20orf160	4.60859066	7.858049247	3.40651633	0.005057254	0.074308627	-2.188331288
BLVRB	-2.648189244	5.899058348	-3.395623927	0.005161196	0.075222191	-2.207998483
GPSM3	2.50771217	6.651954031	3.38091537	0.005304998	0.076697373	-2.234557424
LRRC32	2.22029405	9.731664369	3.37660895	0.005347864	0.077140185	-2.242333662
FAM101B	3.445260931	6.211094152	3.374512264	0.005368861	0.077242971	-2.246119742
SLC39A8	-2.398218647	6.240423524	-3.371509652	0.005399076	0.077350585	-2.251541724
PDPN	-3.169151172	5.201260483	-3.36485046	0.005466703	0.078139142	-2.263566688
ID3	2.083771653	10.75645934	3.363480942	0.005480717	0.078250733	-2.266039735
PHACTR2	2.647254563	8.997188748	3.358062304	0.005536522	0.078272737	-2.275824629
ASCL2	-2.965206122	5.692986413	-3.354023183	0.005578494	0.078666743	-2.283118432
SHE	2.218604858	7.667273042	3.341512327	0.005710551	0.079992134	-2.305710379
ZNF485	-2.06852395	3.583682478	-3.33487444	0.005781897	0.080544057	-2.317696892
ALDH1A3	-2.156189331	6.895051009	-3.331638596	0.005817002	0.080727748	-2.323540043
IGF1	2.324507103	5.64381212	3.33149884	0.005818523	0.080727748	-2.323792408

SLC35F2	-2.228233755	6.065076608	-3.32920312	0.005843567	0.080867012	-2.327937897
ABCC3	-2.426491234	5.170424451	-3.327913258	0.005857685	0.08097351	-2.330267054
C1QTNF5	2.800528187	9.516661471	3.306614929	0.006095862	0.082991934	-2.368724967
ALDH1A2	-2.541762471	6.140913866	-3.305615284	0.006107278	0.083057666	-2.370529926
LOC651751	-2.278512706	4.500533229	-3.297904192	0.006196071	0.083574781	-2.384452781
SHROOM2	2.160752095	6.279149422	3.287644217	0.006316238	0.084531642	-2.402976891
TNFRSF1B	2.116895878	9.080624368	3.276909168	0.006444488	0.085833526	-2.422357421
KIF21A	-2.187247888	6.102953886	-3.273690082	0.006483456	0.086154447	-2.428168707
TK1	3.1927962	6.01405468	3.265145084	0.006588053	0.086969201	-2.443593913
RBPMS2	2.09855111	9.696986198	3.263510304	0.006608257	0.087053603	-2.446544848
PON3	-2.04779476	6.006067477	-3.251651333	0.006756703	0.088546526	-2.467950088
PDE8B	-2.285129582	5.269637511	-3.240343714	0.006901378	0.089512008	-2.488357751
SOX18	2.310536204	11.36848902	3.236194965	0.006955238	0.089657146	-2.495844653
HDAC7	2.357915651	6.631238476	3.234667871	0.00697517	0.089756469	-2.49860038
RAPGEFL1	-3.202100174	5.499468261	-3.232331218	0.007005779	0.089849299	-2.502816904
LOC643977	3.701540672	5.380875779	3.223064151	0.007128514	0.090578746	-2.519538258
ANKRD22	-2.218820799	6.262872385	-3.218477406	0.00719006	0.090879427	-2.527813765
DCHS1	2.857400018	7.295787145	3.212319076	0.007273536	0.091530725	-2.53892394
HRIHFB2122	3.12972663	4.399109979	3.212224166	0.00727483	0.091530725	-2.539095159
ALPL	-2.123349795	6.121216311	-3.210507175	0.007298282	0.091702045	-2.542192587
HIC1	2.409574867	5.493613726	3.2101628	0.007302995	0.091702045	-2.542813824
MORC2	2.069942827	6.012314481	3.194537968	0.007520075	0.0929252	-2.570996974
S1PR3	2.374845315	7.38795495	3.182892925	0.007686074	0.09407605	-2.591996996
CAV1	2.089293408	11.40890297	3.179315031	0.007737813	0.094525778	-2.598448322
TFAP2A	-2.081865087	5.432022517	-3.167854812	0.007905904	0.095358732	-2.619109491
RUNX2	2.225587829	4.649767679	3.161197695	0.008005226	0.095896927	-2.631109266

KIAA1598	-2.211950492	6.96091246	-3.160893535	0.008009793	0.095896927	-2.631657491
RNASE1	2.05065048	9.61817073	3.140639483	0.008319928	0.09708704	-2.668156095
HOXC6	2.768740036	8.203464088	3.140481341	0.008322397	0.09708704	-2.668441011
COCH	-2.636838934	6.022381295	-3.136044225	0.008391954	0.097448516	-2.676434695
MFAP4	-2.406060915	9.711003968	-3.134142399	0.008421945	0.097587741	-2.679860683
MLPH	-3.060038239	7.167894813	-3.133260478	0.008435889	0.097659557	-2.681449341
CD36	2.824395525	10.31325183	3.128542375	0.008510883	0.098167156	-2.689947798
TSPAN8	-2.123351056	7.148243806	-3.127318193	0.00853045	0.098302911	-2.692152693
INF2	2.190995787	7.101142506	3.122071235	0.00861483	0.098823619	-2.70160235
PRRG2	-2.302162658	6.35050815	-3.11845056	0.008673543	0.099046524	-2.7081224
THAP10	2.150584449	6.042702715	3.115277747	0.008725324	0.099099248	-2.713835464
TNMD	2.582236526	7.880439845	3.108637616	0.008834698	0.099981183	-2.725790397
ECHDC2	-2.055510896	9.314822647	-3.107604333	0.008851841	0.100048485	-2.727650541
ZNF296	-2.499011435	5.826036279	-3.107322834	0.008856517	0.100048485	-2.728157294
IGFBP7	2.239607386	12.70376903	3.096946996	0.009030611	0.101180434	-2.746833195
HN1	2.57119972	5.534246195	3.082529437	0.009278227	0.10287652	-2.772775075
UAP1	-2.046821372	6.670398592	-3.074947674	0.009411156	0.103711927	-2.786412798
PLXNA2	2.928101692	6.510399554	3.072770901	0.009449672	0.103954637	-2.790327705
GINS3	2.373089628	6.92223179	3.058517372	0.009705802	0.10541401	-2.815956055
TMEM8	2.055415461	6.731075483	3.041864872	0.010013848	0.1070813	-2.845882945
TSPAN15	2.0627022	6.944062807	3.033474324	0.010172743	0.108043619	-2.860955556
LAMA2	2.574142823	7.530252628	3.008190136	0.010666951	0.111507668	-2.906348092
TMEM166	-2.133224363	5.160774352	-2.993986673	0.01095501	0.113112051	-2.931828369
C14orf78	-2.715471563	8.460093121	-2.992799967	0.010979426	0.113178761	-2.933956607
PDGFRB	2.035787104	11.94322885	2.981885595	0.011206543	0.11494687	-2.953525609
KIAA0100	2.344794245	4.280714012	2.97821424	0.011283988	0.115292193	-2.960106196

CFB	-2.117335769	7.18962965	-2.97643627	0.011321684	0.115385995	-2.963292688
PLA2G4B	-2.221250208	7.865828988	-2.967987417	0.011502539	0.116376034	-2.97843147
EVPL	-2.801942414	6.260340998	-2.967425682	0.011514665	0.116376034	-2.979437799
CDCA7	2.013747118	7.892522789	2.956210489	0.011759444	0.118034484	-2.999524194
FRMD3	2.068865075	7.270505735	2.94106153	0.012098323	0.119908453	-3.026639716
FASN	-2.04357515	10.60415271	-2.939258897	0.012139288	0.12003138	-3.02986502
AP1S2	2.088664937	9.595119112	2.938293363	0.012161287	0.120140662	-3.031592457
GPR162	2.071948903	7.00143092	2.925679293	0.012452359	0.121957383	-3.054152909
PLVAP	2.120552645	7.897428521	2.917492453	0.012644966	0.123007135	-3.068787704
INPP4B	2.144428518	6.924527598	2.914192223	0.012723443	0.1231004	-3.07468549
TMEM100	2.070039222	7.423234107	2.913389474	0.012742605	0.1231004	-3.07611992
DSP	-4.776458048	6.573233428	-2.90350727	0.012980858	0.124023456	-3.093773538
LYZ	-4.431886516	7.560810792	-2.899418989	0.013080712	0.124553274	-3.1010742
RARRES1	-2.422214458	5.886606735	-2.894645343	0.013198271	0.125199851	-3.10959675
COL7A1	-2.413730545	6.368101723	-2.864130158	0.013974917	0.129464154	-3.164023735
ADAP2	3.045605911	8.419703825	2.863480454	0.013991936	0.129464154	-3.165181526
OSR2	-2.002057431	6.406415859	-2.85774324	0.014143116	0.13014764	-3.175403499
LPAR5	-2.360182478	5.470200852	-2.850797159	0.014328318	0.131023725	-3.187774667
PLAC9	2.591047882	10.7408655	2.843658626	0.014521153	0.131558926	-3.200483223
LOC100133773	-2.490598349	2.782700827	-2.841945879	0.014567801	0.131558926	-3.203531564
ERAP2	2.745773596	7.650365864	2.838742087	0.014655456	0.131966531	-3.209232799
ZNF417	2.004192404	5.222294346	2.837622554	0.014686209	0.131966531	-3.211224774
NBEA	-2.178500933	5.037153395	-2.835999154	0.014730917	0.132085828	-3.214113028
EBI2	-2.009498121	5.233180583	-2.830726427	0.014877057	0.132921966	-3.223491923
PSORS1C2	-2.792888362	3.960248567	-2.825915898	0.015011637	0.13374679	-3.232045962
GUCY1A3	2.001718107	8.976377775	2.824063313	0.015063786	0.133906204	-3.235339513

GPD1	-3.105943643	5.233598957	-2.800783648	0.015734549	0.137617253	-3.276692601
SILV	-3.526009898	5.189192603	-2.800493268	0.0157431	0.137617253	-3.277208016
ITGA5	2.190653649	9.602913051	2.799460996	0.015773533	0.137617253	-3.279040188
RBP4	-2.696459208	5.587658792	-2.797437829	0.015833348	0.137852911	-3.28263072
LOC647251	2.01019746	4.510410047	2.795218544	0.015899218	0.138140211	-3.286568734
OSBPL10	3.150694441	7.672026992	2.78687743	0.016149219	0.139680048	-3.301364273
ABCC4	-2.107528125	4.706576219	-2.780559603	0.016341155	0.140470682	-3.312565173
DLEU2	2.05215242	4.540151618	2.770782713	0.016642622	0.141751147	-3.329888782
TCF7L1	2.345482099	6.14663787	2.770495398	0.016651564	0.141751147	-3.33039769
LOC100132439	2.081922064	4.863612341	2.763213924	0.016879762	0.142874685	-3.343291488
KRT10	-2.626731877	10.35832243	-2.761936381	0.016920116	0.142874685	-3.345553012
LOC652330	2.168616031	5.293177005	2.758568576	0.017026953	0.143392646	-3.351513726
LOC388588	-2.140064934	5.976225424	-2.749553895	0.017316205	0.144790508	-3.367461558
GSDMA	-2.755931591	3.840790648	-2.749086868	0.017331321	0.144790508	-3.368287479
LOC641700	3.731255928	5.843506092	2.736130255	0.017755913	0.146392355	-3.391189072
CAMK1	2.033953616	6.959221978	2.724754404	0.018137129	0.147710622	-3.411277587
TNN	3.647370986	5.049258922	2.714030917	0.018503847	0.149154731	-3.430197462
B3GALTL	2.017164137	5.722344004	2.71081603	0.018615201	0.149574151	-3.435866417
CLEC3B	2.183049752	10.53722015	2.698395349	0.019051635	0.151249346	-3.457754262
COL5A2	2.335446394	10.49712541	2.694696136	0.019183547	0.152009416	-3.464268668
DDR2	2.459088611	8.186050908	2.685833664	0.019503236	0.153433738	-3.479867388
CDCA5	2.255958356	6.588689102	2.675660546	0.019876648	0.154400191	-3.497758287
RGS4	2.025146036	5.896910988	2.659420873	0.020487306	0.156797763	-3.526284883
CALB2	-2.637647215	5.65317934	-2.658763928	0.020512392	0.156822154	-3.527437998
CIAPIN1	2.009298315	6.342260568	2.643658261	0.021097582	0.158634484	-3.55393339
DTX3	2.852878916	6.009165073	2.642054299	0.02116067	0.158887771	-3.556744571

LYPD1	2.4372602	6.121864166	2.63273186	0.021531017	0.159953759	-3.573075134
TRIM27	2.473486932	5.092720501	2.630630671	0.021615361	0.160296972	-3.576753894
HEPH	2.015753252	6.577090612	2.623445273	0.021906237	0.161664988	-3.589328484
BCL11A	-2.070771189	5.353965492	-2.614867434	0.022258493	0.162870473	-3.604328405
CD7	-2.760276588	4.428101098	-2.60948614	0.022482297	0.163325627	-3.613732133
LOC653879	-2.985332274	6.098554299	-2.603353447	0.022740031	0.16382112	-3.624442787
KCNMA1	-2.242759339	6.310829935	-2.594747821	0.023106559	0.165232209	-3.639461234
PERP	-4.270092691	6.216788865	-2.592467489	0.023204644	0.165651722	-3.643438644
FAM89A	2.17257756	8.947504271	2.550675493	0.025075793	0.171810064	-3.716164951
EBF1	2.494964457	8.657408352	2.534661307	0.025831025	0.17470464	-3.743945371
SOX7	2.091126541	8.787802037	2.521603563	0.02646314	0.176210516	-3.766559906
PEAR1	2.669916323	6.927493469	2.512770388	0.026899233	0.177057564	-3.781838616
ENC1	2.238927277	7.249178959	2.511810778	0.026947026	0.17712231	-3.783497498
TPD52L1	-2.30713816	7.168400047	-2.511248277	0.02697508	0.17712231	-3.784469808
DSC3	-2.546243952	5.651565178	-2.506718173	0.027202045	0.178056171	-3.792297962
ID1	2.085740088	8.486638717	2.498211609	0.027633249	0.179296122	-3.806986153
OLFML2B	2.710828699	9.371206731	2.48920816	0.028096851	0.180365981	-3.822515909
RGS16	2.6776247	6.085572938	2.478581604	0.028653722	0.182997707	-3.840823303
CDC25B	3.229716255	9.088891631	2.476103582	0.028785107	0.183557836	-3.845088965
CCL8	2.092512059	7.475908131	2.475333936	0.028826032	0.183725877	-3.846413565
AHNAK2	-2.43597005	7.224283059	-2.470169479	0.029102106	0.184787564	-3.855298542
TPPP3	2.882527995	7.945324531	2.465684134	0.029343949	0.185204774	-3.863010476
PCDH10	-2.237586396	3.542487192	-2.457711705	0.029778613	0.186312147	-3.876707106
TSPAN14	2.945274174	7.648041187	2.436462308	0.030967734	0.189795926	-3.913144487
KRT8	-2.235398639	5.556723599	-2.43036279	0.03131744	0.190809438	-3.92358475
LOC158160	-2.380054839	4.6074773	-2.429389878	0.031373572	0.190809438	-3.925249248

PPM1D	2.329327788	6.769676867	2.416005399	0.032155685	0.193133266	-3.948125719
LOC643986	2.239107729	4.607882235	2.415128372	0.032207584	0.193187822	-3.949623255
AKR1C2	-2.359331455	8.878493115	-2.40699966	0.03269246	0.194705992	-3.963494515
TINAGL1	2.922285963	6.834398208	2.398917976	0.033181488	0.196028322	-3.977269947
CACNA1H	2.196069135	7.390327332	2.395607741	0.033383816	0.196728211	-3.982907801
ME1	-3.195852781	5.160919391	-2.395358157	0.033399119	0.196728211	-3.983332774
LOC728910	-4.189088131	5.89783599	-2.369344211	0.035031641	0.201204508	-4.027543894
AKAP2	3.661615844	5.315973483	2.355404115	0.035937691	0.203870638	-4.051165915
CA2	2.37468026	8.464519382	2.355289736	0.035945217	0.203870638	-4.051359531
CSPG4	2.660849355	8.538765657	2.327831079	0.037796359	0.208899034	-4.097742422
MYOZ1	2.101953837	6.737234105	2.313480995	0.038799699	0.21129495	-4.121903424
FMO3	-2.431926625	4.244279345	-2.312600585	0.038862077	0.211451968	-4.123383956
SPRR1A	-2.098035713	7.211507336	-2.305671304	0.039356378	0.2126762	-4.135029179
PPARG	2.195271118	8.99642822	2.299158968	0.039826399	0.213905767	-4.145961757
GGT5	2.238064763	5.725398989	2.280028908	0.041238231	0.21711025	-4.178008504
ARSK	2.597933701	3.989307975	2.261918392	0.042618619	0.221472763	-4.208252302
CA4	2.155622323	8.23060359	2.239535228	0.04438537	0.22666917	-4.245500323
MYOM1	2.073562016	9.100509087	2.23156995	0.045030684	0.228819761	-4.258719786
PRC1	2.247430844	7.535064989	2.226318955	0.045460949	0.230078757	-4.267424151
LOC113230	-2.170295216	3.711556718	-2.220134568	0.04597269	0.231896296	-4.277665101
ZNF788	2.169722253	5.606376667	2.210106444	0.046814094	0.23438653	-4.294246316
LOC441711	2.304768745	5.591389846	2.187308494	0.048781427	0.239173691	-4.331826487
CALML5	-3.714581236	7.450521317	-2.180471442	0.049386474	0.239836392	-4.343064901
MFSD6L	-2.04972515	4.045441124	-2.180099728	0.049419571	0.239846075	-4.343675481
TGM3	-2.165196311	5.638139579	-2.179363591	0.049485177	0.23994601	-4.344884534
