# Supplementary material for: Identification of Diagnostic Markers in Infantile Hemangiomas
Source: J Oncol. 2022 Dec 1;2022:9395876. doi: 10.1155/2022/9395876 (PMC9731762; doi:10.1155/2022/9395876)
Supplement: Supplementary Materials — Table S1: DEGs of IHs in the 6-month-old compared to normal samples. Table S2: DEGs of IHs in the 12-month-old compared to normal samples. Table S3: DEGs of IHs in the 24-month-old compared to normal samples. Table S4: common up- and down-regulated genes among the 6-, 12-, and 24-month-old IHs samples. Table S5: GO and KEGG analysis of candidate genes. Table S6: the top 20 significant genes listed by the SVM-RFE algorithm ranked in 127 candidate genes for characteristics. Table S7: GO items relevant to diagnostic genes. Table S8: all functional annotation enrichment analysis results of the identified diagnostic genes. Table S9: all potential compounds are associated with the identified diagnostic genes. Table S10: potential compounds are associated with the major transcription factors. [file 9395876.f1.zip › Supplementary Table S3.pdf]

## Table S3. DEGs of IHs in the 24-month-old compared to normal samples

| Symbol | logFC | AveExpr | t | P.Value | adj.P.Val | B |
|---|---|---|---|---|---|---|
| CETP | 2.952349001 | 6.539706948 | 8.900642665 | 7.97E-07 | 0.010045211 | 5.744358431 |
| WARS | 3.156889091 | 10.3397108 | 7.864718498 | 3.07E-06 | 0.011117847 | 4.642102169 |
| PCDH17 | 4.38349224 | 8.664316175 | 7.854576658 | 3.11E-06 | 0.011117847 | 4.630562878 |
| ISL1 | 3.335869982 | 6.244070858 | 7.76334078 | 3.53E-06 | 0.011117847 | 4.526074117 |
| STEAP4 | 4.266641051 | 6.542675055 | 7.590626736 | 4.48E-06 | 0.011295487 | 4.324879344 |
| IDO2 | 5.082885755 | 7.720475735 | 7.196489581 | 7.84E-06 | 0.015272085 | 3.848737195 |
| ADA | 2.373107725 | 7.392416158 | 7.089142166 | 9.16E-06 | 0.015272085 | 3.714864673 |
| MGC16121 | 5.661785171 | 7.875580095 | 7.050264235 | 9.69E-06 | 0.015272085 | 3.665930347 |
| TMEM2 | 3.124389258 | 8.769044509 | 6.958235055 | 1.11E-05 | 0.015539301 | 3.549136696 |
| CRMP1 | 4.082875634 | 6.052038759 | 6.876395444 | 1.25E-05 | 0.01578576 | 3.444134559 |
| HECW2 | 2.528186951 | 7.297384204 | 6.644066311 | 1.77E-05 | 0.020336073 | 3.140143262 |
| PCSK5 | 2.478384794 | 8.766273935 | 6.433932078 | 2.45E-05 | 0.023745024 | 2.857580025 |
| APLN | 5.355691956 | 7.387770042 | 6.43360905 | 2.45E-05 | 0.023745024 | 2.857140042 |
| FAM184A | 3.08414724 | 7.339071842 | 6.280076486 | 3.11E-05 | 0.027994159 | 2.646057585 |
| C20orf46 | 3.445734133 | 6.550585238 | 6.211748741 | 3.46E-05 | 0.029086912 | 2.550855289 |
| RGS5 | 3.819474173 | 10.39541124 | 6.033841982 | 4.59E-05 | 0.03342754 | 2.299311102 |
| LOC653158 | 2.057235835 | 6.98494309 | 6.003903308 | 4.82E-05 | 0.03342754 | 2.256459185 |
| FAM69B | 2.556372609 | 9.520539801 | 5.997887108 | 4.86E-05 | 0.03342754 | 2.247829919 |
| FCGR2B | 2.701351094 | 6.786709783 | 5.837103145 | 6.30E-05 | 0.036915246 | 2.014962283 |
| HEY1 | 2.442356861 | 7.213820191 | 5.833132049 | 6.35E-05 | 0.036915246 | 2.009155987 |
| EXOC6 | 2.517885342 | 8.324333426 | 5.815875393 | 6.53E-05 | 0.036915246 | 1.983893651 |
| PVRL2 | 2.257598636 | 7.904082882 | 5.782477762 | 6.89E-05 | 0.036915246 | 1.934860541 |
| TFPI2 | 6.924015015 | 5.941344094 | 5.770556821 | 7.03E-05 | 0.036915246 | 1.917313441 |

| | | | | | | |
|---|---|---|---|---|---|---|
| PCDHB2 | 2.411031637 | 5.515571861 | 5.667539517 | 8.32E-05 | 0.04092579 | 1.764686324 |
| IL18R1 | 2.830114108 | 6.945781497 | 5.64110588 | 8.70E-05 | 0.04092579 | 1.725237368 |
| C5orf13 | 2.065707988 | 10.00359468 | 5.627944504 | 8.89E-05 | 0.04092579 | 1.705552194 |
| PDE1A | 2.977168324 | 6.402048622 | 5.573340307 | 9.73E-05 | 0.04092579 | 1.623573932 |
| COX4I2 | 5.342415208 | 6.8133587 | 5.541274274 | 0.000102645 | 0.04092579 | 1.575201685 |
| DYSF | 3.23306321 | 8.818944797 | 5.53702442 | 0.000103376 | 0.04092579 | 1.568777894 |
| ACVRL1 | 2.596432211 | 9.144153785 | 5.530432499 | 0.00010452 | 0.04092579 | 1.558808066 |
| H19 | 4.338960385 | 11.7568533 | 5.514431549 | 0.000107352 | 0.04092579 | 1.534577785 |
| ICAM2 | 3.016838957 | 9.712142578 | 5.510405928 | 0.000108077 | 0.04092579 | 1.528475103 |
| HS3ST3A1 | 2.98171821 | 7.101171144 | 5.485186532 | 0.000112739 | 0.04092579 | 1.490182484 |
| MTUS1 | 3.155020364 | 7.752083908 | 5.461232326 | 0.000117362 | 0.04092579 | 1.453713597 |
| KHDRBS3 | 2.389297632 | 8.126731878 | 5.458583457 | 0.000117886 | 0.04092579 | 1.449675033 |
| TDO2 | 3.10958546 | 6.666401371 | 5.44339085 | 0.000120935 | 0.04092579 | 1.426489482 |
| SCN4B | 3.175469269 | 8.48498554 | 5.429232486 | 0.000123852 | 0.04092579 | 1.404848073 |
| LOC152217 | 2.165735975 | 4.655821666 | 5.416183734 | 0.000126605 | 0.04092579 | 1.38487353 |
| KCNMB3 | 2.657972657 | 6.640264376 | 5.357461763 | 0.000139813 | 0.042990737 | 1.294638266 |
| TINAGL1 | 3.639243397 | 7.225465899 | 5.3121946 | 0.000150977 | 0.043538963 | 1.224693453 |
| IGF2 | 5.069961266 | 7.337397903 | 5.310557135 | 0.000151398 | 0.043538963 | 1.222157056 |
| PDGFB | 3.021172836 | 7.188688017 | 5.29707994 | 0.00015491 | 0.043538963 | 1.201264616 |
| MGC52282 | 5.847754255 | 4.710498404 | 5.29173393 | 0.000156326 | 0.043538963 | 1.192969017 |
| SH2D3C | 3.431133531 | 8.342115894 | 5.282284403 | 0.000158864 | 0.043538963 | 1.178294493 |
| ANKRD20A1 | 3.673894698 | 8.251999762 | 5.247125308 | 0.000168689 | 0.045248084 | 1.123567553 |
| LOC100132091 | 3.03708419 | 6.315816641 | 5.167313494 | 0.000193429 | 0.049766577 | 0.998596674 |
| COL4A2 | 3.389768242 | 10.35871093 | 5.136326494 | 0.000204033 | 0.050436143 | 0.949801639 |
| MEG3 | 3.418089521 | 10.48047882 | 5.099801016 | 0.000217317 | 0.051166989 | 0.892089078 |
| FAM162B | 2.663357144 | 9.214741424 | 5.098307782 | 0.000217879 | 0.051166989 | 0.889725174 |

| | | | | | | |
|---|---|---|---|---|---|---|
| TIE1 | 2.211211014 | 7.281208357 | 5.094905621 | 0.000219165 | 0.051166989 | 0.884337976 |
| CYTSB | 2.125139896 | 4.924992526 | 5.068386507 | 0.000229466 | 0.051658516 | 0.842283295 |
| WSCD1 | 2.423211975 | 6.168133001 | 5.021441962 | 0.00024896 | 0.054691705 | 0.767566389 |
| PGF | 2.656492542 | 6.604508891 | 4.995009856 | 0.00026069 | 0.055191235 | 0.725345527 |
| FKBP1A | 2.262886217 | 8.871778037 | 4.981197751 | 0.000267047 | 0.055191235 | 0.703239809 |
| C1orf54 | 2.483596934 | 9.721336617 | 4.892199717 | 0.000312091 | 0.059552233 | 0.560097164 |
| BST2 | 2.643075399 | 9.546350382 | 4.88965411 | 0.00031349 | 0.059552233 | 0.555985042 |
| GUCY1B3 | 2.10486102 | 6.61889627 | 4.88423586 | 0.000316491 | 0.059552233 | 0.547229238 |
| NDUFA4L2 | 3.984071937 | 9.021656257 | 4.875601604 | 0.000321334 | 0.059574382 | 0.533267222 |
| GJA4 | 2.634160943 | 8.891708577 | 4.850960416 | 0.000335586 | 0.060517274 | 0.493359241 |
| PECAM1 | 3.060210004 | 11.24829808 | 4.837136446 | 0.000343868 | 0.060517274 | 0.470930402 |
| SYTL2 | 2.096106788 | 6.924052029 | 4.828317636 | 0.000349262 | 0.060517274 | 0.45660726 |
| SEMA5B | 2.890041243 | 5.672072316 | 4.82644181 | 0.000350421 | 0.060517274 | 0.453559121 |
| GPC5 | 3.359148425 | 5.449533531 | 4.75810468 | 0.000395488 | 0.065209946 | 0.342157509 |
| APLNR | 3.414621056 | 10.30150181 | 4.750802776 | 0.000400648 | 0.065209946 | 0.330213352 |
| PLAC8 | 3.946454089 | 4.919836505 | 4.739699394 | 0.000408629 | 0.065209946 | 0.312035934 |
| TUSC3 | 2.818475548 | 8.024723507 | 4.714731928 | 0.000427186 | 0.066734498 | 0.271095897 |
| BCAR1 | 2.152763949 | 7.215962018 | 4.704807746 | 0.000434804 | 0.066848435 | 0.254797743 |
| B3GAT3 | 2.298670663 | 4.956836118 | 4.695672211 | 0.000441941 | 0.067127131 | 0.239782182 |
| FAM43B | 2.080934883 | 5.220128105 | 4.651626142 | 0.000478103 | 0.068125868 | 0.16721812 |
| TCEAL7 | 2.374909225 | 6.558240178 | 4.649215868 | 0.000480169 | 0.068125868 | 0.163239309 |
| CDH5 | 3.020600056 | 10.61626153 | 4.648979805 | 0.000480371 | 0.068125868 | 0.16284958 |
| STARD8 | 2.717032239 | 7.551576279 | 4.642074358 | 0.000486343 | 0.068125868 | 0.151445479 |
| LOC652330 | 2.558314921 | 5.505740037 | 4.620086592 | 0.000505877 | 0.06874777 | 0.115088609 |
| RSPO3 | 3.440885599 | 7.922078414 | 4.61869333 | 0.000507142 | 0.06874777 | 0.112782552 |
| STX3 | 2.008098438 | 7.997557185 | 4.591127418 | 0.000532856 | 0.069128357 | 0.067101016 |

| | | | | | | |
|---|---|---|---|---|---|---|
| RUVBL1 | 2.231061948 | 6.893853835 | 4.590657242 | 0.000533306 | 0.069128357 | 0.066320934 |
| MYLIP | 2.143910435 | 10.60062747 | 4.586435175 | 0.000537366 | 0.069128357 | 0.059314608 |
| MYO1B | 2.095263793 | 8.676597393 | 4.536252494 | 0.000588155 | 0.071822709 | -0.024149397 |
| CD4 | 2.427697032 | 5.929851052 | 4.52711076 | 0.000597931 | 0.071822709 | -0.039391022 |
| NOX4 | 3.269758562 | 7.214847802 | 4.525116389 | 0.000600087 | 0.071822709 | -0.042717657 |
| ARID3A | 2.396700137 | 8.282917022 | 4.519225279 | 0.0006065 | 0.071822709 | -0.052547244 |
| KCNJ8 | 2.564138966 | 8.924080355 | 4.516937161 | 0.000609009 | 0.071822709 | -0.056366336 |
| FLJ41603 | -2.600789842 | 6.475434684 | -4.511944912 | 0.000614523 | 0.071822709 | -0.064701338 |
| PMEPA1 | 2.100731865 | 9.033989247 | 4.511262084 | 0.000615281 | 0.071822709 | -0.065841642 |
| ATP1B2 | 2.47127628 | 6.394576883 | 4.460678362 | 0.000674233 | 0.074561884 | -0.150488125 |
| RCAN1 | 2.741062634 | 9.751610235 | 4.445571574 | 0.000692952 | 0.075965576 | -0.17583334 |
| MRI1 | 2.186824543 | 8.540226479 | 4.440165793 | 0.00069978 | 0.076052815 | -0.18491007 |
| TBX3 | 2.284088611 | 7.93388235 | 4.417685121 | 0.000728933 | 0.077016383 | -0.222697597 |
| ZNF641 | 2.836156353 | 5.853261348 | 4.384329997 | 0.000774525 | 0.077016383 | -0.278883624 |
| FHOD1 | 2.457911813 | 8.152246801 | 4.362954433 | 0.000805286 | 0.077016383 | -0.314964568 |
| LOC645993 | 4.32686656 | 4.79524381 | 4.358047523 | 0.000812524 | 0.077016383 | -0.323255301 |
| TNFRSF10A | 2.385775715 | 5.702970536 | 4.354482652 | 0.000817824 | 0.077016383 | -0.329280404 |
| SDCCAG8 | 2.040659963 | 4.778679088 | 4.354129826 | 0.000818351 | 0.077016383 | -0.329876812 |
| NPDC1 | 2.399365978 | 7.468341665 | 4.353957518 | 0.000818608 | 0.077016383 | -0.330168084 |
| LOC100133999 | 2.142383577 | 6.980946751 | 4.332799813 | 0.000850844 | 0.07799015 | -0.365961275 |
| PAPSS2 | 2.467999455 | 8.317129146 | 4.271927054 | 0.000951115 | 0.083517617 | -0.469246786 |
| POPDC2 | 2.277892331 | 6.661083543 | 4.262779399 | 0.000967206 | 0.083517617 | -0.484806442 |
| COL18A1 | 2.287182696 | 10.72846142 | 4.255895709 | 0.000979501 | 0.08400385 | -0.496521733 |
| LOC643911 | -2.225597955 | 4.751112595 | -4.239070173 | 0.001010235 | 0.084930258 | -0.525180346 |
| FUT11 | 2.108106877 | 5.17727629 | 4.236761172 | 0.00101453 | 0.084930258 | -0.529115797 |
| LOC652377 | 3.249479637 | 6.461599461 | 4.219404921 | 0.001047422 | 0.084930258 | -0.558717452 |

| | | | | | | |
|---|---|---|---|---|---|---|
| HYAL2 | 2.253971615 | 8.915900243 | 4.209231641 | 0.001067211 | 0.084930258 | -0.576084394 |
| EPAS1 | 2.438854989 | 12.11920207 | 4.202807428 | 0.001079906 | 0.084930258 | -0.587057327 |
| XAGE1 | -2.62798244 | 4.03840841 | -4.20113731 | 0.001083231 | 0.084930258 | -0.589910754 |
| ADAMTS9 | 2.403835209 | 8.179313546 | 4.196148378 | 0.001093229 | 0.085076146 | -0.59843631 |
| MKL1 | 2.287109134 | 6.252589681 | 4.190105874 | 0.001105465 | 0.085356039 | -0.608766069 |
| ENPEP | 2.83731962 | 7.84964654 | 4.149929188 | 0.001190499 | 0.088285999 | -0.677552122 |
| ADAM9 | 2.406468902 | 4.911249718 | 4.142251052 | 0.001207502 | 0.089023286 | -0.690717949 |
| ANKRD47 | 2.238362533 | 7.424297535 | 4.129124797 | 0.001237153 | 0.089384067 | -0.713240514 |
| COL4A1 | 2.431773536 | 12.86067154 | 4.127551625 | 0.001240756 | 0.089384067 | -0.715941067 |
| CHN1 | 3.366822198 | 8.634747616 | 4.119554784 | 0.001259242 | 0.090200337 | -0.729672774 |
| EVI1 | 2.010936352 | 8.533890725 | 4.099501803 | 0.001306854 | 0.091024907 | -0.764136365 |
| RPL23AP13 | 2.378765734 | 7.831645836 | 4.076471653 | 0.001363828 | 0.091661612 | -0.803768382 |
| CD34 | 2.030491117 | 9.466748998 | 4.070261077 | 0.001379626 | 0.091661612 | -0.814465352 |
| KCTD7 | 2.894264438 | 3.670777268 | 4.06650306 | 0.001389276 | 0.091661612 | -0.820939993 |
| LOC100132707 | 2.104440105 | 5.564673957 | 4.05721308 | 0.001413432 | 0.091661612 | -0.836951722 |
| MSL3L1 | 2.041095842 | 7.496265604 | 4.054352496 | 0.001420956 | 0.091661612 | -0.841883828 |
| JAM3 | 2.6017789 | 10.65745284 | 4.048516109 | 0.001436437 | 0.091661612 | -0.851949239 |
| NRGN | 2.149371911 | 7.087909387 | 4.042652071 | 0.001452165 | 0.091844837 | -0.862065761 |
| DBNDD1 | -2.247468439 | 6.269117556 | -4.018603177 | 0.001518544 | 0.094306791 | -0.903589906 |
| FAM13C1 | 3.587635651 | 5.378404604 | 4.013315821 | 0.001533551 | 0.094499281 | -0.912726906 |
| JAM2 | 2.522930327 | 9.493627885 | 4.012235965 | 0.001536635 | 0.094499281 | -0.914593322 |
| SERPINE1 | 2.230736801 | 7.023096496 | 4.009476022 | 0.001544545 | 0.094524659 | -0.919364097 |
| PHF21B | -2.100326769 | 3.937585029 | -3.995748409 | 0.001584517 | 0.095226727 | -0.943104156 |
| COLEC11 | 2.326611922 | 6.887028769 | 3.994087164 | 0.001589425 | 0.095226727 | -0.94597827 |
| LOC158376 | 2.223905873 | 5.312757064 | 3.992616396 | 0.001593784 | 0.095226727 | -0.948523057 |
| SLC38A11 | 2.905913979 | 5.25590806 | 3.926665214 | 0.001802407 | 0.101735047 | -1.062839555 |

| | | | | | | |
|---|---|---|---|---|---|---|
| SRGN | 2.083434992 | 10.32057046 | 3.921423169 | 0.001820147 | 0.101735047 | -1.071942578 |
| GPR176 | 2.489698371 | 5.990137338 | 3.920534676 | 0.001823171 | 0.101735047 | -1.07348572 |
| CRIM1 | 2.03275005 | 8.077598481 | 3.920362406 | 0.001823758 | 0.101735047 | -1.073784928 |
| SERPINH1 | 2.262472626 | 10.08183652 | 3.871090525 | 0.001999868 | 0.107154605 | -1.159466542 |
| ARAP3 | 2.255565044 | 8.541020836 | 3.865894754 | 0.002019427 | 0.107154605 | -1.168513507 |
| ESAM | 2.537042551 | 9.984722212 | 3.859576194 | 0.002043477 | 0.107154605 | -1.179518427 |
| CD163L1 | 3.058333679 | 5.511810638 | 3.857347241 | 0.002052031 | 0.107154605 | -1.183401319 |
| STXBP6 | 2.766933631 | 7.655783227 | 3.852159693 | 0.002072081 | 0.107154605 | -1.19243969 |
| ROBO4 | 2.087014793 | 7.15907311 | 3.852018446 | 0.00207263 | 0.107154605 | -1.192685817 |
| SULT1A2 | 2.046884897 | 5.55236246 | 3.849682169 | 0.002081727 | 0.107154605 | -1.196757083 |
| HRC | 2.976792576 | 7.199441162 | 3.841954004 | 0.002112114 | 0.107368618 | -1.210227478 |
| MFNG | 2.305348168 | 8.44970671 | 3.827433178 | 0.002170441 | 0.107727378 | -1.235550229 |
| LYL1 | 2.100434424 | 9.104877249 | 3.818268886 | 0.002208098 | 0.109166656 | -1.251540076 |
| MGC2752 | 2.895632254 | 4.941469763 | 3.809952191 | 0.002242851 | 0.109595436 | -1.266056511 |
| BCHE | -2.657129735 | 5.492007789 | -3.793533429 | 0.00231311 | 0.110507866 | -1.294729783 |
| KCNJ2 | 2.086721007 | 7.333759427 | 3.790403245 | 0.002326758 | 0.110507866 | -1.300198478 |
| PHACTR2 | 2.149391372 | 8.725627008 | 3.789286289 | 0.002331648 | 0.110507866 | -1.302150065 |
| HLX | 2.611954342 | 6.647889761 | 3.769491851 | 0.002420071 | 0.112733815 | -1.336750355 |
| NID1 | 2.249605227 | 6.68804874 | 3.766368859 | 0.002434332 | 0.112733815 | -1.342211791 |
| LOC285016 | 4.179167343 | 7.561179454 | 3.74755879 | 0.002522057 | 0.113962628 | -1.375120628 |
| CEACAM1 | 3.483929501 | 7.511565706 | 3.718454187 | 0.002664201 | 0.116188814 | -1.426086257 |
| C13orf23 | 2.018666204 | 8.299004283 | 3.703118329 | 0.002742362 | 0.116800547 | -1.452962813 |
| ATP6V0E2 | -2.290840261 | 8.213468744 | -3.673676533 | 0.002899033 | 0.119524471 | -1.504600202 |
| ERAP2 | 2.552544757 | 7.544968316 | 3.654018663 | 0.003008691 | 0.121572328 | -1.539105469 |
| SHANK3 | 2.111333173 | 9.919370028 | 3.637332451 | 0.003105082 | 0.123099896 | -1.568411128 |
| EFNB1 | 2.137819713 | 7.283061003 | 3.614686147 | 0.003240952 | 0.126497485 | -1.608207414 |

| | | | | | | |
|---|---|---|---|---|---|---|
| TEK | 2.260434621 | 9.201731941 | 3.609311493 | 0.003274077 | 0.127395968 | -1.617656017 |
| C6orf188 | 2.0070031 | 5.01757562 | 3.601964968 | 0.003319913 | 0.127994325 | -1.630573396 |
| ARHGAP4 | 2.314699364 | 7.486709189 | 3.595730541 | 0.003359322 | 0.128689997 | -1.641537367 |
| EBF1 | 3.05928808 | 8.965221237 | 3.570678257 | 0.003522536 | 0.129849733 | -1.685612458 |
| SLC2A1 | 2.939144302 | 9.912435515 | 3.564227966 | 0.003565848 | 0.131063123 | -1.696965017 |
| CYGB | 2.078917805 | 9.725317559 | 3.551527738 | 0.003652716 | 0.132326987 | -1.719322517 |
| LOC100130367 | 2.201685992 | 6.082279541 | 3.536022103 | 0.003761691 | 0.133448675 | -1.746627288 |
| PRSS35 | 2.155649798 | 6.733142092 | 3.532970708 | 0.003783523 | 0.133448675 | -1.752001738 |
| TTC14 | 2.28997159 | 6.769873949 | 3.528874974 | 0.003813028 | 0.133448675 | -1.759216131 |
| RPL14 | -2.0005528 | 7.23917221 | -3.512850982 | 0.003930723 | 0.134551081 | -1.78744717 |
| MORC2 | 2.170183869 | 6.066991413 | 3.511021169 | 0.003944395 | 0.134551081 | -1.790671492 |
| FAM150B | 3.328720193 | 7.977230661 | 3.507021184 | 0.003974451 | 0.134551081 | -1.797720275 |
| GCOM1 | 2.032352932 | 7.010382198 | 3.502021875 | 0.004012342 | 0.134551081 | -1.806530806 |
| C10orf11 | 3.891488197 | 5.344951509 | 3.471352371 | 0.004252944 | 0.13841543 | -1.86059796 |
| GPR116 | 2.181455979 | 10.85937132 | 3.464851211 | 0.004305795 | 0.138694098 | -1.872062265 |
| PROCR | 2.142374017 | 8.328036623 | 3.459186265 | 0.00435239 | 0.13900122 | -1.882052869 |
| FLJ35776 | 2.27958415 | 4.299228379 | 3.457521006 | 0.004366184 | 0.13900122 | -1.884989849 |
| LOC100132439 | 2.283089822 | 4.973340209 | 3.44999032 | 0.004429118 | 0.139562609 | -1.898272391 |
| EDNRA | 2.226158068 | 9.681819532 | 3.441589483 | 0.004500407 | 0.140090445 | -1.91309128 |
| LOC643432 | -2.051639804 | 3.778612051 | -3.436516496 | 0.004544017 | 0.140668488 | -1.922040682 |
| SH2B3 | 2.35898714 | 9.187933653 | 3.435549609 | 0.004552378 | 0.140668488 | -1.923746459 |
| ALDH1A2 | -2.09143493 | 6.38654707 | -3.435541187 | 0.00455245 | 0.140668488 | -1.923761316 |
| PPEF1 | 2.059379238 | 4.081353922 | 3.41734644 | 0.00471271 | 0.143164173 | -1.955863934 |
| TMEM44 | 2.157347833 | 7.881907459 | 3.405759276 | 0.004817731 | 0.144124635 | -1.976311415 |
| FILIP1 | 2.222441705 | 6.213830641 | 3.400335504 | 0.004867699 | 0.144733677 | -1.98588333 |
| SLC41A2 | -2.520240477 | 3.653352583 | -3.38745958 | 0.00498843 | 0.146615559 | -2.008608626 |

| | | | | | | |
|---|---|---|---|---|---|---|
| GGCX | 2.121718668 | 5.228295441 | 3.38304774 | 0.005030489 | 0.146615559 | -2.016395802 |
| KLHL23 | 3.565809317 | 4.15755938 | 3.380457645 | 0.005055348 | 0.146615559 | -2.020967594 |
| STARD3NL | 2.579551343 | 6.55603031 | 3.37439604 | 0.005114011 | 0.146615559 | -2.031667282 |
| RPA4 | 2.168137764 | 4.446695448 | 3.372427613 | 0.005133209 | 0.146615559 | -2.035141954 |
| GPR162 | 2.334724823 | 7.14476324 | 3.370924942 | 0.005147913 | 0.146615559 | -2.037794499 |
| C1QTNF5 | 2.754704826 | 9.49166691 | 3.369350945 | 0.005163361 | 0.146615559 | -2.040572976 |
| TMEM8 | 2.034961741 | 6.719918908 | 3.357774293 | 0.005278424 | 0.148140575 | -2.061009221 |
| GUCY1A2 | 2.176036087 | 4.53737653 | 3.352647144 | 0.005330207 | 0.148904845 | -2.070060527 |
| PLA2G4C | 2.330842287 | 8.518139509 | 3.351811069 | 0.0053387 | 0.148904845 | -2.071536527 |
| FCAR | 2.025943202 | 9.200194477 | 3.345922477 | 0.005398903 | 0.14939277 | -2.08193232 |
| ADCY4 | 2.855710808 | 9.271016889 | 3.341594871 | 0.005443583 | 0.149514707 | -2.089572461 |
| LOC728059 | 2.79639873 | 6.55505997 | 3.331189387 | 0.005552546 | 0.149731509 | -2.107943114 |
| P2RY14 | 2.35230675 | 5.399604135 | 3.331010103 | 0.005554442 | 0.149731509 | -2.10825964 |
| GPER | 2.504068881 | 8.504053251 | 3.319623065 | 0.005676246 | 0.150021883 | -2.128363604 |
| LOC644242 | 2.516392463 | 4.755351858 | 3.292766824 | 0.005974286 | 0.154022629 | -2.175778752 |
| DDR2 | 2.334196528 | 8.117927953 | 3.286799297 | 0.006042623 | 0.15439843 | -2.186314277 |
| NEBL | -2.442487632 | 6.095180292 | -3.270366396 | 0.006234895 | 0.156946282 | -2.215325145 |
| THY1 | 3.429544964 | 10.3213406 | 3.26987741 | 0.00624071 | 0.156946282 | -2.216188378 |
| CRHBP | 2.247306761 | 6.493051591 | 3.269190734 | 0.006248885 | 0.156946282 | -2.217400601 |
| GPSM3 | 2.426976384 | 6.60791633 | 3.263128009 | 0.006321529 | 0.157812892 | -2.228103255 |
| LOC643977 | 3.355553069 | 5.192155268 | 3.259525768 | 0.006365092 | 0.158273609 | -2.234462207 |
| TMEM100 | 2.297887317 | 7.547514887 | 3.247533047 | 0.006512313 | 0.160059482 | -2.255631683 |
| SETD4 | 2.29352112 | 4.340085197 | 3.242308432 | 0.006577515 | 0.16070298 | -2.264853606 |
| SLC31A2 | -2.126178515 | 7.757485304 | -3.221951673 | 0.00683788 | 0.161370295 | -2.300781408 |
| LOC196549 | 2.200861513 | 5.958353741 | 3.214834594 | 0.006931329 | 0.161521743 | -2.313340786 |
| SCG2 | 3.965862328 | 5.193417671 | 3.188239597 | 0.007292011 | 0.164124941 | -2.360263512 |

| | | | | | | |
|---|---|---|---|---|---|---|
| FLJ21865 | 2.42168903 | 5.613900382 | 3.18445631 | 0.007344827 | 0.164319865 | -2.366937239 |
| IGFALS | 2.131848874 | 4.321116229 | 3.150595186 | 0.00783494 | 0.167400054 | -2.426651227 |
| NDC80 | 3.010353941 | 5.217990003 | 3.146681611 | 0.007893657 | 0.167400054 | -2.433550609 |
| COPG2IT1 | -2.479906129 | 4.17791913 | -3.130634858 | 0.008139054 | 0.167400054 | -2.461834664 |
| RTN4R | -2.096755305 | 4.451095606 | -3.112223188 | 0.00843003 | 0.169553515 | -2.494275683 |
| ALDOC | -2.08456703 | 8.21505599 | -3.111877974 | 0.008435584 | 0.169553515 | -2.494883819 |
| HRIHFB2122 | 3.145157159 | 4.407526631 | 3.106239917 | 0.008526809 | 0.169883037 | -2.504815254 |
| PTGDR | 2.708318094 | 5.210898581 | 3.102731553 | 0.008584073 | 0.169883037 | -2.510994578 |
| CD93 | 2.6305549 | 10.74354605 | 3.102570329 | 0.008586713 | 0.169883037 | -2.511278532 |
| C12orf35 | 2.007144654 | 7.669915924 | 3.093844987 | 0.008730843 | 0.170123251 | -2.52664427 |
| TMEM108 | 3.468711184 | 3.635979829 | 3.089994029 | 0.008795222 | 0.170782761 | -2.533424936 |
| SHE | 2.169779861 | 7.640641226 | 3.065285664 | 0.009219707 | 0.171942079 | -2.576914513 |
| MGC61598 | 2.023877254 | 9.29291896 | 3.053619904 | 0.009427165 | 0.173794688 | -2.59743719 |
| IGFBP7 | 2.190164343 | 12.6768001 | 3.051104471 | 0.009472504 | 0.173866043 | -2.601861471 |
| GGT5 | 2.672627328 | 5.962433115 | 3.049871663 | 0.009494805 | 0.173866043 | -2.60402968 |
| CAMK1 | 2.154519217 | 7.024985033 | 3.046071796 | 0.00956387 | 0.174049147 | -2.610712205 |
| JAG1 | 2.320539737 | 9.411110727 | 3.040298675 | 0.009669759 | 0.174094618 | -2.62086343 |
| MYO3B | 2.085668835 | 7.13576402 | 3.027506586 | 0.009908568 | 0.175471262 | -2.643349866 |
| HEYL | 2.76334966 | 9.147642112 | 3.027020193 | 0.009917763 | 0.175471262 | -2.644204682 |
| PDLIM7 | 2.073558578 | 5.268703578 | 3.026694132 | 0.009923932 | 0.175471262 | -2.644777712 |
| LOC654203 | -2.57469534 | 3.320169128 | -3.022068174 | 0.010011865 | 0.175910508 | -2.65290685 |
| ZNF417 | 2.322742417 | 5.396048899 | 3.005199857 | 0.01033913 | 0.177889359 | -2.682538333 |
| C1QB | 2.879807297 | 8.672983032 | 2.986173378 | 0.010721049 | 0.178547241 | -2.71593915 |
| RUNX2 | 2.086760665 | 4.574043772 | 2.983746657 | 0.010770758 | 0.178667033 | -2.720197501 |
| MGC16384 | 2.118114179 | 6.501862003 | 2.982293739 | 0.010800629 | 0.178836778 | -2.722746853 |
| LOXL3 | 2.226806426 | 5.041199491 | 2.97336866 | 0.010985938 | 0.179150852 | -2.738403958 |

| | | | | | | |
|---|---|---|---|---|---|---|
| LOC646332 | 3.59696507 | 4.488520907 | 2.958422709 | 0.011303354 | 0.180782804 | -2.764610618 |
| TRIM27 | 2.748493594 | 5.242724135 | 2.957921202 | 0.011314161 | 0.180782804 | -2.765489691 |
| NSUN5B | 2.188892787 | 5.643844863 | 2.947566872 | 0.011539597 | 0.182578908 | -2.783635157 |
| CDH6 | 2.481031699 | 7.786628988 | 2.928897328 | 0.011957386 | 0.184869124 | -2.816331549 |
| INF2 | 2.014292957 | 7.004759144 | 2.913544236 | 0.012312165 | 0.186184578 | -2.8431985 |
| DTX3 | 3.132889273 | 6.161897996 | 2.912853852 | 0.012328361 | 0.186184578 | -2.844406164 |
| TBX15 | 2.682763735 | 7.250944897 | 2.912242386 | 0.012342723 | 0.186184578 | -2.845475743 |
| C20orf160 | 3.961291095 | 7.504976757 | 2.908057549 | 0.012441464 | 0.186301777 | -2.852795021 |
| HDAC7 | 2.365836988 | 6.635559205 | 2.906755987 | 0.012472333 | 0.186301777 | -2.855071144 |
| FAM101B | 2.902419986 | 5.914999091 | 2.900451573 | 0.012622931 | 0.187309905 | -2.866093966 |
| HOXC6 | 2.699289398 | 8.165581922 | 2.88767913 | 0.012933573 | 0.187309905 | -2.888414811 |
| AACS | -2.132963517 | 6.674042961 | -2.881957799 | 0.013075163 | 0.187309905 | -2.898408456 |
| CLEC3B | 2.166327648 | 10.528099 | 2.880422424 | 0.013113421 | 0.187309905 | -2.901089831 |
| SEC24A | -2.57146807 | 4.616954114 | -2.870397511 | 0.013365948 | 0.188483783 | -2.91859184 |
| KIAA0100 | 2.265592611 | 4.237513121 | 2.844410059 | 0.014043155 | 0.193066576 | -2.963916415 |
| C14orf121 | -2.337089845 | 3.052210705 | -2.829742355 | 0.014440215 | 0.195177032 | -2.989467896 |
| ANTXR2 | 2.243421181 | 9.298590221 | 2.825798217 | 0.01454886 | 0.195901195 | -2.996334797 |
| C8orf4 | 2.175769324 | 9.076894485 | 2.820689295 | 0.014690789 | 0.195901195 | -3.005227138 |
| RAPGEF3 | 2.118648253 | 6.358908924 | 2.800906767 | 0.01525334 | 0.199051643 | -3.039632485 |
| Gcom1 | 2.022834144 | 6.059216176 | 2.799442066 | 0.015295825 | 0.199051643 | -3.042178109 |
| FUT6 | 2.135801398 | 5.655450909 | 2.788083744 | 0.015629259 | 0.2003156 | -3.061910257 |
| CAPN3 | -2.112483943 | 2.858800495 | -2.774442184 | 0.016039179 | 0.201519547 | -3.085588841 |
| C15orf28 | 2.875538805 | 4.958223494 | 2.76439632 | 0.01634779 | 0.203451721 | -3.103011733 |
| FASTKD1 | 2.175684808 | 4.953434484 | 2.756992874 | 0.016578952 | 0.204711903 | -3.115843792 |
| PPAN | 2.097605181 | 5.526391696 | 2.735492841 | 0.017268604 | 0.207605029 | -3.153069255 |
| LOC90342 | 2.211685252 | 3.534544226 | 2.732585267 | 0.017364004 | 0.208087445 | -3.158098848 |

| | | | | | | |
|---|---|---|---|---|---|---|
| SIPA1L3 | 2.631394448 | 4.575897302 | 2.727580791 | 0.017529416 | 0.209226677 | -3.166753081 |
| CD36 | 2.437122927 | 10.10201223 | 2.713630705 | 0.017998703 | 0.211432511 | -3.190859093 |
| ALDH1L1 | -2.293940053 | 5.985940987 | -2.710902934 | 0.018091895 | 0.211432511 | -3.195569615 |
| RGS4 | 2.025688518 | 5.897206888 | 2.7027536 | 0.018373132 | 0.213062478 | -3.209636333 |
| CES1 | -2.884908914 | 6.401731088 | -2.671763995 | 0.019482188 | 0.217880979 | -3.263041161 |
| ACSL1 | -2.860087801 | 8.208676897 | -2.669731553 | 0.019557169 | 0.217880979 | -3.266538757 |
| LOC388588 | -2.199714611 | 5.943689236 | -2.651337218 | 0.020248716 | 0.219586004 | -3.298164688 |
| FBP1 | -2.347114983 | 5.876506115 | -2.64174347 | 0.02061881 | 0.220476115 | -3.31463869 |
| CLDN8 | -3.199741738 | 5.479318271 | -2.610965398 | 0.021851179 | 0.223293711 | -3.367389682 |
| TK1 | 2.491391802 | 5.631470463 | 2.610841337 | 0.021856289 | 0.223293711 | -3.367601996 |
| OSBPL10 | 2.898569968 | 7.534504552 | 2.600042633 | 0.022305542 | 0.224499974 | -3.386072598 |
| DLX5 | 2.206752548 | 7.088886584 | 2.589495143 | 0.022753008 | 0.22550878 | -3.404094257 |
| ADAP2 | 2.729526151 | 8.247296683 | 2.587682335 | 0.022830788 | 0.225893424 | -3.407189716 |
| DCHS1 | 2.384369645 | 7.037770577 | 2.583717472 | 0.023001805 | 0.226097015 | -3.41395791 |
| IGFBP4 | 2.017028849 | 10.75417834 | 2.583438347 | 0.023013891 | 0.226097015 | -3.414434284 |
| LOC100134144 | 2.287592435 | 7.087457994 | 2.57894496 | 0.02320931 | 0.226847539 | -3.422101123 |
| LAMA2 | 2.385858067 | 7.427551852 | 2.537711543 | 0.025079379 | 0.23022646 | -3.492284777 |
| GTSF1 | 2.022247773 | 4.943573942 | 2.52373436 | 0.025745811 | 0.231675543 | -3.516003136 |
| NFKBIE | 2.115537579 | 4.390693511 | 2.512607866 | 0.026288539 | 0.232902048 | -3.534856937 |
| LOC647520 | -2.763412105 | 3.342438233 | -2.510447522 | 0.026395191 | 0.233117122 | -3.538514802 |
| NBPF14 | 2.714363272 | 5.417558859 | 2.501831114 | 0.026824742 | 0.234766224 | -3.553094728 |
| USHBP1 | 2.245259864 | 5.379819855 | 2.480604988 | 0.027911914 | 0.237774406 | -3.588947477 |
| LOC641700 | 3.23861812 | 5.574794561 | 2.471105121 | 0.028412139 | 0.239291398 | -3.604963357 |
| DZIP1 | 2.342834979 | 4.46830784 | 2.465454437 | 0.028713768 | 0.240567201 | -3.614480848 |
| PTGS2 | -2.896532106 | 5.575382217 | -2.452530303 | 0.029415287 | 0.242695365 | -3.636223389 |
| OLFML2B | 2.555772999 | 9.286630894 | 2.42373638 | 0.031037992 | 0.247447435 | -3.684532418 |

| | | | | | | |
|---|---|---|---|---|---|---|
| CHI3L1 | -2.073073336 | 6.488053088 | -2.414220147 | 0.031592946 | 0.249645911 | -3.700457325 |
| PLAC9 | 2.142691665 | 10.49630756 | 2.400588908 | 0.032404486 | 0.25248662 | -3.723232113 |
| HN1 | 2.137268407 | 5.297556388 | 2.393536608 | 0.032832153 | 0.252695331 | -3.734997895 |
| RNASEH2C | 2.421042352 | 5.442216992 | 2.372332523 | 0.034150818 | 0.257255532 | -3.770302501 |
| LYPD1 | 2.060251577 | 5.916223099 | 2.360082907 | 0.034935541 | 0.258951454 | -3.790648188 |
| PEAR1 | 2.404699262 | 6.782829618 | 2.359129733 | 0.03499732 | 0.258951454 | -3.792229783 |
| C9orf40 | 2.049306806 | 5.128627599 | 2.35276454 | 0.035412554 | 0.25993461 | -3.802785704 |
| TRIP10 | 2.149876887 | 6.401918003 | 2.35251161 | 0.035429151 | 0.25993461 | -3.80320495 |
| HIC1 | 2.102174153 | 5.325940609 | 2.343172582 | 0.036047182 | 0.261259747 | -3.818673609 |
| LOC647251 | 2.195328714 | 4.611390731 | 2.320647587 | 0.037580409 | 0.26497551 | -3.855891043 |
| GPR124 | 2.091376131 | 8.936856918 | 2.312781262 | 0.038130338 | 0.266320872 | -3.86885725 |
| PRRG2 | -2.347565342 | 6.32574305 | -2.270597546 | 0.041212301 | 0.272140806 | -3.938105736 |
| LOC100132112 | 2.305641803 | 5.096583097 | 2.263282875 | 0.041770204 | 0.272989612 | -3.950063571 |
| TNP1 | -2.009611449 | 3.819636435 | -2.242526317 | 0.043392545 | 0.278568419 | -3.98391315 |
| PPM1D | 2.073787648 | 6.630291336 | 2.229161965 | 0.044468451 | 0.280453253 | -4.005641731 |
| COCH | -2.013195264 | 6.36255057 | -2.222490051 | 0.04501496 | 0.281727238 | -4.0164697 |
