# Supplementary material for: Identification of Diagnostic Markers in Infantile Hemangiomas
Source: J Oncol. 2022 Dec 1;2022:9395876. doi: 10.1155/2022/9395876 (PMC9731762; doi:10.1155/2022/9395876)
Supplement: Supplementary Materials — Table S1: DEGs of IHs in the 6-month-old compared to normal samples. Table S2: DEGs of IHs in the 12-month-old compared to normal samples. Table S3: DEGs of IHs in the 24-month-old compared to normal samples. Table S4: common up- and down-regulated genes among the 6-, 12-, and 24-month-old IHs samples. Table S5: GO and KEGG analysis of candidate genes. Table S6: the top 20 significant genes listed by the SVM-RFE algorithm ranked in 127 candidate genes for characteristics. Table S7: GO items relevant to diagnostic genes. Table S8: all functional annotation enrichment analysis results of the identified diagnostic genes. Table S9: all potential compounds are associated with the identified diagnostic genes. Table S10: potential compounds are associated with the major transcription factors. [file 9395876.f1.zip › Supplementary Table S4.pdf]

Table S4. Common up- and down-regulated genes among the 6-, 12-, and 24-month-old IHs samples

Common up-regulated genes	Common down-regulated genes
SERPINE1	ALDH1L1
ICAM2	SLC31A2
ANKRD20A1	ACSL1
ANTXR2	NEBL
GPR176	CLDN8
EDNRA	ALDH1A2
TDO2	CHI3L1
GJA4	FBP1
MGC16121	FLJ41603
C8orf4	COCH
TRIM27	CES1
STARD8	
MTUS1	
EBF1	
ISL1	
RUNX2	
C5orf13	
GPR162	
HECW2	
IDO2	
GTSF1	
MEG3	
CETP	

ENPEP
HS3ST3A1
EPAS1
USHBP1
PDE1A
FUT11
PDGFB
LYPD1
THY1
PCDH17
PLAC8
PLAC9
SERPINH1
KCNMB3
STXBP6
HOXC6
JAG1
IGF2
BST2
LOC644242
H19
HRC
NOX4
DCHS1
ADA
SETD4

ACVRL1
LOC100132091
COL18A1
ROBO4
TINAGL1
FAM69B
LOC652330
PEAR1
LOC643977
PCSK5
ARHGAP4
OSBPL10
SHE
LOC646332
HEY1
TMEM44
HYAL2
C1QTNF5
RSPO3
TK1
CD36
CD34
WARS
C20orf46
STARD3NL
LOC100133999

FAM101B
CEACAM1
CYTSB
EXOC6
DDR2
POPDC2
C12orf35
NDUFA4L2
ADCY4
MGC61598
PAPSS2
TCEAL7
LOC100132439
FAM184A
CHN1
STX3
JAM2
JAM3
PRSS35
FKBP1A
HEYL
FILIP1
CAMK1
SEMA5B
GUCY1B3
OLFML2B

STEAP4
C1orf54
TUSC3
FHOD1
C20orf160
CDH6
RGS4
CDH5
RGS5
FAM162B
RUVBL1
C6orf188
MYLIP
GUCY1A2
CD93
ANKRD47
ATP1B2
PGF
HIC1
PROCR
COL4A2
COL4A1
MFNG
APLNR
IGFALS
SCG2

KHDRBS3
MRI1
SLC38A11
DTX3
NID1
MGC52282
EFNB1
ADAP2
GPR116
EVI1
MORC2
WSCD1
TFPI2
TNFRSF10A
KLHL23
NDC80
RCAN1
LOC652377
GPER
ESAM
Gcom1
COX4I2
DYSF
SLC2A1
KIAA0100
LYL1

TMEM108
PHACTR2
PMEPA1
ADAMTS9
GPSM3
SH2D3C
ARAP3
LOC641700
TMEM2
TMEM8
LOC645993
PECAM1
SCN4B
LOC647251
GCOM1
COLEC11
CRMP1
CYGB
HDAC7
FAM13C1
HRIHFB2122
HLX
C10orf11
SH2B3
KCNJ2
PDLIM7

KCNJ8
ERAP2
TIE1
LOC285016
ARID3A
HN1
APLN
LOC158376
TBX15
MYO1B
CD4
TEK
FCGR2B
PVRL2
BCAR1
