# Supplementary material for: Identification of Diagnostic Markers in Infantile Hemangiomas
Source: J Oncol. 2022 Dec 1;2022:9395876. doi: 10.1155/2022/9395876 (PMC9731762; doi:10.1155/2022/9395876)
Supplement: Supplementary Materials — Table S1: DEGs of IHs in the 6-month-old compared to normal samples. Table S2: DEGs of IHs in the 12-month-old compared to normal samples. Table S3: DEGs of IHs in the 24-month-old compared to normal samples. Table S4: common up- and down-regulated genes among the 6-, 12-, and 24-month-old IHs samples. Table S5: GO and KEGG analysis of candidate genes. Table S6: the top 20 significant genes listed by the SVM-RFE algorithm ranked in 127 candidate genes for characteristics. Table S7: GO items relevant to diagnostic genes. Table S8: all functional annotation enrichment analysis results of the identified diagnostic genes. Table S9: all potential compounds are associated with the identified diagnostic genes. Table S10: potential compounds are associated with the major transcription factors. [file 9395876.f1.zip › Supplementary Table S5.pdf]

Table S5. GO and KEGG analysis of candidate genes

GO	KEGG
blood vessel development	Purine metabolism
vasculogenesis	cGMP-PKG signaling pathway
stress fiber	Notch signaling pathway
regulation of endothelial cell proliferation	Cell adhesion molecules
positive regulation of endothelial cell proliferation	Leukocyte transendothelial migration
blood vessel maturation	Adipocytokine signaling pathway
regulation of systemic arterial blood pressure by circulatory renin-angiotensin	Renin secretion
regulation of heart rate	AGE-RAGE signaling pathway in diabetic complications
sprouting angiogenesis	Renal cell carcinoma
regulation of adaptive immune response	
circulatory system process	
heart process	
vascular process in circulatory system	
endothelium development	
heart valve development	
atrioventricular valve development	
aortic valve morphogenesis	
pulmonary valve morphogenesis	
endocardial cushion development	
cardiac chamber development	
cardiac septum development	

cell-cell junction
adherens junction
blood circulation
regulation of heart contraction
regulation of blood pressure
negative regulation of platelet activation
regulation of fibroblast migration
mesenchymal cell development
long-chain fatty acid import across plasma membrane
fusion of virus membrane with host plasma membrane
heme binding
negative regulation of cell migration
platelet alpha granule
platelet alpha granule membrane
actin filament bundle
adherens junction organization
collagen-activated tyrosine kinase receptor signaling pathway
negative regulation of locomotion
cell-cell contact zone
cell-cell junction organization
endothelial cell differentiation
regulation of angiogenesis
positive regulation of angiogenesis
positive regulation of heart contraction
blood vessel morphogenesis
mesenchymal cell differentiation

regulation of coagulation
regulation of antigen receptor-mediated signaling pathway
negative regulation of cell activation
regulation of release of sequestered calcium ion into cytosol
regulation of focal adhesion assembly
heart trabecula formation
mesenchyme development
artery development
Notch signaling involved in heart development
renal system development
nephron development
glomerulus vasculature development
endothelial cell apoptotic process
membrane repolarization
regulation of endothelial cell apoptotic process
