# Supplementary material for: Identification of Diagnostic Markers in Infantile Hemangiomas
Source: J Oncol. 2022 Dec 1;2022:9395876. doi: 10.1155/2022/9395876 (PMC9731762; doi:10.1155/2022/9395876)
Supplement: Supplementary Materials — Table S1: DEGs of IHs in the 6-month-old compared to normal samples. Table S2: DEGs of IHs in the 12-month-old compared to normal samples. Table S3: DEGs of IHs in the 24-month-old compared to normal samples. Table S4: common up- and down-regulated genes among the 6-, 12-, and 24-month-old IHs samples. Table S5: GO and KEGG analysis of candidate genes. Table S6: the top 20 significant genes listed by the SVM-RFE algorithm ranked in 127 candidate genes for characteristics. Table S7: GO items relevant to diagnostic genes. Table S8: all functional annotation enrichment analysis results of the identified diagnostic genes. Table S9: all potential compounds are associated with the identified diagnostic genes. Table S10: potential compounds are associated with the major transcription factors. [file 9395876.f1.zip › Supplementary Table S6.pdf]

Table S6. The top 20 significant genes listed by the SVM-RFE algorithm ranked in 127 candidate genes for characteristics

FeatureName	FeatureID	AvgRank
FKBP1A	68	3.8
KHDRBS3	120	6.1
WARS	99	7.3
FUT11	51	8.1
TFPI2	62	9.9
CYTSB	56	12.1
GUCY1A2	73	15.5
STEAP4	114	17.1
ISL1	75	17.8
PVRL2	94	18.4
CRMP1	42	19.7
TMEM2	55	21
FAM162B	49	22.7
ENPEP	32	23.4
SH2D3C	20	23.9
FAM13C1	104	24
TDO2	116	24.1
LYPD1	58	24.2
COL4A2	77	24.3
KCNJ8	82	24.8
