# Supplementary material for: Identification of Diagnostic Markers in Infantile Hemangiomas
Source: J Oncol. 2022 Dec 1;2022:9395876. doi: 10.1155/2022/9395876 (PMC9731762; doi:10.1155/2022/9395876)
Supplement: Supplementary Materials — Table S1: DEGs of IHs in the 6-month-old compared to normal samples. Table S2: DEGs of IHs in the 12-month-old compared to normal samples. Table S3: DEGs of IHs in the 24-month-old compared to normal samples. Table S4: common up- and down-regulated genes among the 6-, 12-, and 24-month-old IHs samples. Table S5: GO and KEGG analysis of candidate genes. Table S6: the top 20 significant genes listed by the SVM-RFE algorithm ranked in 127 candidate genes for characteristics. Table S7: GO items relevant to diagnostic genes. Table S8: all functional annotation enrichment analysis results of the identified diagnostic genes. Table S9: all potential compounds are associated with the identified diagnostic genes. Table S10: potential compounds are associated with the major transcription factors. [file 9395876.f1.zip › Supplementary Table S7.pdf]

Table S7. GO items relevant to diagnostic genes

ID	Term	Term PValue	Gene
GO:0001568	blood vessel development	2.30E-17	ISL1,WARS
GO:0001570	vasculogenesis	1.67E-04	WARS
GO:0002027	regulation of heart rate	6.08E-06	ISL1
GO:0003013	circulatory system process	1.48E-11	ISL1
GO:0003015	heart process	1.95E-05	ISL1
GO:0003197	endocardial cushion development	3.00E-07	ISL1
GO:0003205	cardiac chamber development	2.40E-04	ISL1
GO:0003279	cardiac septum development	6.54E-04	ISL1
GO:0005911	cell-cell junction	2.15E-08	TMEM2
GO:0005912	adherens junction	5.14E-05	TMEM2
GO:0008015	blood circulation	1.15E-10	ISL1
GO:0008016	regulation of heart contraction	1.05E-04	ISL1
GO:0014031	mesenchymal cell development	2.19E-05	ISL1
GO:0020037	heme binding	9.11E-04	GUCY1A2,STEAP4
GO:0045765	regulation of angiogenesis	1.34E-07	ISL1,WARS
GO:0045766	positive regulation of angiogenesis	5.81E-06	ISL1
GO:0048514	blood vessel morphogenesis	3.60E-13	ISL1,WARS
GO:0048762	mesenchymal cell differentiation	7.43E-05	ISL1
GO:0060485	mesenchyme development	2.15E-05	ISL1
