# Supplementary material for: Identification of Diagnostic Markers in Infantile Hemangiomas
Source: J Oncol. 2022 Dec 1;2022:9395876. doi: 10.1155/2022/9395876 (PMC9731762; doi:10.1155/2022/9395876)
Supplement: Supplementary Materials — Table S1: DEGs of IHs in the 6-month-old compared to normal samples. Table S2: DEGs of IHs in the 12-month-old compared to normal samples. Table S3: DEGs of IHs in the 24-month-old compared to normal samples. Table S4: common up- and down-regulated genes among the 6-, 12-, and 24-month-old IHs samples. Table S5: GO and KEGG analysis of candidate genes. Table S6: the top 20 significant genes listed by the SVM-RFE algorithm ranked in 127 candidate genes for characteristics. Table S7: GO items relevant to diagnostic genes. Table S8: all functional annotation enrichment analysis results of the identified diagnostic genes. Table S9: all potential compounds are associated with the identified diagnostic genes. Table S10: potential compounds are associated with the major transcription factors. [file 9395876.f1.zip › Supplementary Table S8.pdf]

Table S8. All functional annotation enrichment analysis results of the identified diagnostic genes

GUCY1A2

GO

ONTOLOGY	ID	Description	setSize	enrichmentScore	NES	pvalue	p.adjust	qvalues	rank	leading_edge
BP	GO:0006631	fatty acid metabolic process	217	-0.37496448	-2.208002917	1.00E-10	3.17E-07	2.62E-07	2907	tags=44%, list=23%, signal=34%
BP	GO:0032787	monocarboxylic acid metabolic process	374	-0.310060172	-1.974630361	1.00E-10	3.17E-07	2.62E-07	2342	tags=32%, list=19%, signal=27%
BP	GO:0043903	regulation of symbiotic process	150	0.462920302	2.05614808	5.26E-09	1.11E-05	9.19E-06	3082	tags=46%, list=24%, signal=35%
BP	GO:0016054	organic acid catabolic process	169	-0.36025271	-2.078510387	1.39E-08	1.58E-05	1.31E-05	2640	tags=40%, list=21%, signal=32%
BP	GO:0046395	carboxylic acid catabolic process	169	-0.36025271	-2.078510387	1.39E-08	1.58E-05	1.31E-05	2640	tags=40%, list=21%, signal=32%
BP	GO:0009062	fatty acid catabolic process	70	-0.496905651	-2.492093433	1.52E-08	1.58E-05	1.31E-05	2640	tags=47%, list=21%, signal=37%
BP	GO:0050792	regulation of viral process	142	0.462304472	2.042571031	1.91E-08	1.58E-05	1.31E-05	2684	tags=42%, list=21%, signal=34%
BP	GO:0072329	monocarboxylic acid catabolic process	80	-0.466253434	-2.437539639	2.06E-08	1.58E-05	1.31E-05	2640	tags=46%, list=21%, signal=37%
BP	GO:0016042	lipid catabolic process	165	-0.362758486	-2.080204157	2.25E-08	1.58E-05	1.31E-05	2673	tags=38%, list=21%, signal=30%
BP	GO:0044242	cellular lipid catabolic process	126	-0.391107421	-2.146871133	4.08E-08	2.59E-05	2.14E-05	2673	tags=40%, list=21%, signal=32%
BP	GO:0034440	lipid oxidation	69	-0.491852909	-2.475833872	5.50E-08	3.17E-05	2.62E-05	2640	tags=45%, list=21%, signal=36%
CC	GO:0005925	focal adhesion	335	0.368418076	1.748527026	6.72E-08	3.55E-05	2.94E-05	3110	tags=41%, list=25%, signal=31%
BP	GO:0072330	monocarboxylic acid biosynthetic process	130	-0.376481172	-2.080372297	7.93E-08	3.87E-05	3.20E-05	2998	tags=45%, list=24%, signal=35%
CC	GO:0030055	cell-substrate junction	337	0.364955175	1.733112298	1.26E-07	5.73E-05	4.73E-05	3110	tags=40%, list=25%, signal=31%
BP	GO:0009615	response to virus	212	0.398971644	1.835126903	2.91E-07	0.000123037	0.000102	3059	tags=41%, list=24%, signal=31%
BP	GO:1903900	regulation of viral life cycle	99	0.488513091	2.059928517	4.01E-07	0.000159049	0.000132	3082	tags=51%, list=24%, signal=38%
BP	GO:0010817	regulation of hormone levels	233	-0.292200628	-1.773522572	5.17E-07	0.000183504	0.000152	1994	tags=28%, list=16%, signal=24%
BP	GO:0045069	regulation of viral genome replication	67	0.537262001	2.143639837	5.21E-07	0.000183504	0.000152	3082	tags=55%, list=24%, signal=42%
BP	GO:0019395	fatty acid oxidation	66	-0.475518462	-2.376916055	6.39E-07	0.000213127	0.000176	2640	tags=44%, list=21%, signal=35%
BP	GO:0051607	defense response to virus	162	0.422393147	1.8903532	7.74E-07	0.000245177	0.000203	3059	tags=43%, list=24%, signal=33%
MF	GO:0016614	oxidoreductase activity, acting on CH-OH group of donors	80	-0.427997681	-2.237541296	8.89E-07	0.000260442	0.000215	1982	tags=35%, list=16%, signal=30%
BP	GO:0051301	cell division	330	0.358743021	1.701631497	9.04E-07	0.000260442	0.000215	3857	tags=42%, list=31%, signal=30%
BP	GO:0030036	actin cytoskeleton organization	422	0.335284955	1.613501052	1.38E-06	0.000380272	0.000314	3343	tags=40%, list=27%, signal=30%
BP	GO:0006720	isoprenoid metabolic process	70	-0.439820686	-2.205799511	1.80E-06	0.000461413	0.000381	1618	tags=36%, list=13%, signal=31%
BP	GO:1901568	fatty acid derivative metabolic process	90	-0.402746526	-2.134159925	1.82E-06	0.000461413	0.000381	2049	tags=49%, list=16%, signal=41%
BP	GO:0019079	viral genome replication	83	0.489663599	2.017831985	2.18E-06	0.000531949	0.00044	3082	tags=48%, list=24%, signal=37%
BP	GO:0048013	ephrin receptor signaling pathway	64	0.520321887	2.052709281	2.78E-06	0.000652026	0.000539	2642	tags=47%, list=21%, signal=37%
BP	GO:0016101	diterpenoid metabolic process	50	-0.494120614	-2.29334833	3.37E-06	0.000713982	0.00059	1618	tags=62%, list=13%, signal=54%
BP	GO:0006721	terpenoid metabolic process	56	-0.469498032	-2.248883872	3.38E-06	0.000713982	0.00059	1618	tags=59%, list=13%, signal=52%
MF	GO:0016616	oxidoreductase activity, acting on the CH-OH group of donors, NAD or NADP as acceptor	74	-0.424949389	-2.164425755	3.39E-06	0.000713982	0.00059	1982	tags=35%, list=16%, signal=30%
BP	GO:0042572	retinol metabolic process	19	-0.703532549	-2.527182035	3.49E-06	0.000713982	0.00059	3279	tags=79%, list=26%, signal=59%
BP	GO:1901570	fatty acid derivative biosynthetic process	59	-0.459055527	-2.232851167	3.98E-06	0.000788732	0.000652	2907	tags=54%, list=23%, signal=42%
BP	GO:1901615	organic hydroxy compound metabolic process	285	-0.25831968	-1.570857268	4.12E-06	0.000790945	0.000654	1777	tags=25%, list=14%, signal=22%

BP	GO:0048525	negative regulation of viral process	64	0.516446473	2.03742048	4.39E-06	0.000818253	0.000677	2684	tags=48%, list=21%, signal=38%
BP	GO:0006635	fatty acid beta-oxidation	49	-0.500879983	-2.333951851	4.64E-06	0.000840535	0.000695	2640	tags=59%, list=21%, signal=47%
BP	GO:0000375	RNA splicing, via transesterification reactions	250	0.366557007	1.709053725	4.93E-06	0.000868514	0.000718	4413	tags=47%, list=35%, signal=31%
BP	GO:0006636	unsaturated fatty acid biosynthetic process	35	-0.553146454	-2.345031099	5.86E-06	0.000900461	0.000744	2326	tags=63%, list=18%, signal=51%
BP	GO:0060337	type I interferon signaling pathway	69	0.504583535	2.032455781	5.88E-06	0.000900461	0.000744	3059	tags=51%, list=24%, signal=39%
BP	GO:0071357	cellular response to type I interferon	69	0.504583535	2.032455781	5.88E-06	0.000900461	0.000744	3059	tags=51%, list=24%, signal=39%
BP	GO:0006820	anion transport	289	-0.259638646	-1.584231108	5.89E-06	0.000900461	0.000744	2273	tags=30%, list=18%, signal=25%
BP	GO:0015711	organic anion transport	236	-0.281032326	-1.704955405	5.93E-06	0.000900461	0.000744	1419	tags=25%, list=11%, signal=22%
MF	GO:0016627	oxidoreductase activity, acting on the CH-CH group of donors	47	-0.49737649	-2.291532118	5.97E-06	0.000900461	0.000744	2165	tags=49%, list=17%, signal=41%
BP	GO:1903901	negative regulation of viral life cycle	54	0.53581529	2.062664837	6.96E-06	0.00102339	0.000846	3082	tags=56%, list=24%, signal=42%
BP	GO:0044772	mitotic cell cycle phase transition	379	0.334426258	1.599357338	7.10E-06	0.00102339	0.000846	2969	tags=34%, list=24%, signal=27%
BP	GO:0034340	response to type I interferon	72	0.49994866	2.024651886	7.70E-06	0.001084981	0.000897	3059	tags=50%, list=24%, signal=38%
BP	GO:0044770	cell cycle phase transition	399	0.332414487	1.592257641	8.29E-06	0.00114291	0.000945	2969	tags=34%, list=24%, signal=27%
BP	GO:0016053	organic acid biosynthetic process	196	-0.291804781	-1.70911255	8.92E-06	0.00120372	0.000995	2998	tags=39%, list=24%, signal=30%
BP	GO:0045071	negative regulation of viral genome replication	39	0.587828217	2.129981644	1.04E-05	0.001346966	0.001114	3082	tags=62%, list=24%, signal=47%
BP	GO:0046394	carboxylic acid biosynthetic process	195	-0.291335086	-1.718662603	1.05E-05	0.001346966	0.001114	2564	tags=34%, list=20%, signal=28%
MF	GO:0016491	oxidoreductase activity	428	-0.22622541	-1.421545091	1.06E-05	0.001346966	0.001114	2056	tags=25%, list=16%, signal=21%
BP	GO:0000904	cell morphogenesis involved in differentiation	425	0.323791286	1.557580216	1.25E-05	0.001511423	0.00125	3295	tags=38%, list=26%, signal=29%
BP	GO:0000377	RNA splicing, via transesterification reactions with bulged adenosine as nucleophile	247	0.364300687	1.700548961	1.26E-05	0.001511423	0.00125	4413	tags=47%, list=35%, signal=31%
BP	GO:0000398	mRNA splicing, via spliceosome	247	0.364300687	1.700548961	1.26E-05	0.001511423	0.00125	4413	tags=47%, list=35%, signal=31%
BP	GO:0030029	actin filament-based process	462	0.319514004	1.542186022	1.48E-05	0.001740139	0.001439	3465	tags=39%, list=27%, signal=30%
BP	GO:0033559	unsaturated fatty acid metabolic process	60	-0.444574243	-2.180859853	1.52E-05	0.001747133	0.001445	2907	tags=52%, list=23%, signal=40%
BP	GO:0120254	olefinic compound metabolic process	54	-0.448687249	-2.128071218	1.56E-05	0.001765318	0.00146	1982	tags=39%, list=16%, signal=33%
BP	GO:0002478	antigen processing and presentation of exogenous peptide antigen	136	0.411304848	1.804525716	1.67E-05	0.001855684	0.001534	3601	tags=47%, list=29%, signal=34%
BP	GO:0019884	antigen processing and presentation of exogenous antigen	138	0.414440879	1.824398481	1.81E-05	0.001975847	0.001634	3601	tags=47%, list=29%, signal=34%
CC	GO:0005788	endoplasmic reticulum lumen	164	0.38500001	1.725374342	2.32E-05	0.002497376	0.002065	3440	tags=45%, list=27%, signal=33%
BP	GO:0006633	fatty acid biosynthetic process	91	-0.377320788	-2.004650305	2.96E-05	0.003129013	0.002587	2326	tags=44%, list=18%, signal=36%
BP	GO:0051640	organelle localization	392	0.322141192	1.542017512	3.12E-05	0.003198137	0.002644	4389	tags=45%, list=35%, signal=30%
BP	GO:0006457	protein folding	154	0.393381891	1.752362258	3.13E-05	0.003198137	0.002644	3005	tags=39%, list=24%, signal=30%
CC	GO:0042613	MHC class II protein complex	10	-0.809901905	-2.408016003	3.28E-05	0.003257245	0.002693	1923	tags=80%, list=15%, signal=68%
BP	GO:0048002	antigen processing and presentation of peptide antigen	143	0.403941643	1.784531889	3.29E-05	0.003257245	0.002693	3601	tags=46%, list=29%, signal=33%
BP	GO:0032990	cell part morphogenesis	392	0.321281302	1.537901409	3.58E-05	0.003478984	0.002876	2600	tags=32%, list=21%, signal=26%
BP	GO:0006066	alcohol metabolic process	212	-0.274732252	-1.592736949	3.62E-05	0.003478984	0.002876	1777	tags=26%, list=14%, signal=23%
BP	GO:0044282	small molecule catabolic process	259	-0.255202372	-1.55377101	3.83E-05	0.003624025	0.002996	2093	tags=32%, list=17%, signal=28%
BP	GO:0030968	endoplasmic reticulum unfolded protein response	95	0.444911471	1.868617323	4.20E-05	0.003916057	0.003238	2702	tags=40%, list=21%, signal=32%
BP	GO:0034308	primary alcohol metabolic process	49	-0.464369721	-2.163824881	4.81E-05	0.004417346	0.003652	1443	tags=33%, list=11%, signal=29%
BP	GO:0016126	sterol biosynthetic process	55	-0.431905394	-2.053696273	4.92E-05	0.004417346	0.003652	2930	tags=47%, list=23%, signal=36%
BP	GO:0001676	long-chain fatty acid metabolic process	58	-0.431538272	-2.084810143	5.00E-05	0.004417346	0.003652	2053	tags=41%, list=16%, signal=35%
BP	GO:0019882	antigen processing and presentation	165	0.384224097	1.724782666	5.02E-05	0.004417346	0.003652	3601	tags=42%, list=29%, signal=31%

BP	GO:0001568	blood vessel development	412	0.319151784	1.534858998	5.55E-05	0.004771383	0.003945	2648	tags=31%, list=21%, signal=26%
BP	GO:0034754	cellular hormone metabolic process	57	-0.432644999	-2.077742244	5.65E-05	0.004771383	0.003945	1756	tags=37%, list=14%, signal=32%
BP	GO:1901617	organic hydroxy compound biosynthetic process	136	-0.314568657	-1.74087983	5.71E-05	0.004771383	0.003945	2964	tags=42%, list=24%, signal=32%
CC	GO:0030027	lamellipodium	143	0.397259184	1.755010146	5.87E-05	0.004771383	0.003945	3295	tags=43%, list=26%, signal=32%
BP	GO:1903311	regulation of mRNA metabolic process	229	0.352250772	1.632559299	5.89E-05	0.004771383	0.003945	3554	tags=38%, list=28%, signal=28%
BP	GO:0035966	response to topologically incorrect protein	152	0.38468412	1.711697293	5.93E-05	0.004771383	0.003945	3311	tags=39%, list=26%, signal=29%
BP	GO:0035967	cellular response to topologically incorrect protein	126	0.409092875	1.779064244	5.99E-05	0.004771383	0.003945	3311	tags=41%, list=26%, signal=31%
CC	GO:0098687	chromosomal region	192	0.369933671	1.686903294	6.02E-05	0.004771383	0.003945	4001	tags=42%, list=32%, signal=29%
CC	GO:0098794	postsynapse	311	0.328435169	1.553975344	6.44E-05	0.005040653	0.004168	3391	tags=37%, list=27%, signal=28%
BP	GO:0001523	retinoid metabolic process	46	-0.462112206	-2.101224405	6.56E-05	0.005070591	0.004192	1443	tags=59%, list=11%, signal=52%
BP	GO:0043648	dicarboxylic acid metabolic process	55	-0.423944207	-2.015841084	7.02E-05	0.005363265	0.004434	2800	tags=47%, list=22%, signal=37%
BP	GO:0050690	regulation of defense response to virus by virus	22	0.641778494	2.044785014	7.25E-05	0.00540927	0.004472	2600	tags=59%, list=21%, signal=47%
BP	GO:0001525	angiogenesis	309	0.32897788	1.555459361	7.25E-05	0.00540927	0.004472	3779	tags=42%, list=30%, signal=30%
BP	GO:0034620	cellular response to unfolded protein	112	0.411689882	1.763060624	7.55E-05	0.005564414	0.004601	2702	tags=37%, list=21%, signal=29%
BP	GO:0035384	thioester biosynthetic process	37	-0.491762805	-2.128008247	7.93E-05	0.0057142	0.004724	2800	tags=59%, list=22%, signal=46%
BP	GO:0071616	acyl-CoA biosynthetic process	37	-0.491762805	-2.128008247	7.93E-05	0.0057142	0.004724	2800	tags=59%, list=22%, signal=46%
BP	GO:0019058	viral life cycle	224	0.352259391	1.628429493	8.44E-05	0.006008879	0.004968	3950	tags=43%, list=31%, signal=30%
CC	GO:0000139	Golgi membrane	478	0.306778784	1.48292003	9.14E-05	0.006374489	0.00527	3221	tags=33%, list=26%, signal=26%
BP	GO:0048858	cell projection morphogenesis	377	0.322313677	1.541211687	9.15E-05	0.006374489	0.00527	2600	tags=32%, list=21%, signal=26%
BP	GO:0015849	organic acid transport	154	-0.288572047	-1.637998452	9.30E-05	0.00640934	0.005299	2782	tags=37%, list=22%, signal=29%
MF	GO:0048156	tau protein binding	31	0.595284859	2.067118042	9.56E-05	0.006518252	0.005389	4071	tags=68%, list=32%, signal=46%
BP	GO:0099504	synaptic vesicle cycle	92	0.429169338	1.796664335	9.75E-05	0.006578042	0.005439	2642	tags=37%, list=21%, signal=29%
BP	GO:0000280	nuclear division	204	0.356436115	1.634192772	0.000120518	0.00804173	0.006649	2730	tags=30%, list=22%, signal=24%
BP	GO:0008380	RNA splicing	311	0.322901242	1.527791833	0.000124791	0.008240075	0.006813	3312	tags=35%, list=26%, signal=26%
BP	GO:0048514	blood vessel morphogenesis	358	0.318946849	1.520424439	0.000131114	0.008511495	0.007037	3779	tags=41%, list=30%, signal=29%
BP	GO:0044839	cell cycle G2/M phase transition	179	0.361582242	1.634628869	0.000132148	0.008511495	0.007037	3241	tags=38%, list=26%, signal=29%
BP	GO:0043393	regulation of protein binding	136	0.390883978	1.714932836	0.000132991	0.008511495	0.007037	3348	tags=43%, list=27%, signal=32%
BP	GO:0034976	response to endoplasmic reticulum stress	210	0.352332205	1.618819229	0.000134272	0.008511495	0.007037	3374	tags=37%, list=27%, signal=28%
BP	GO:0034504	protein localization to nucleus	185	0.363069461	1.644857793	0.000136997	0.008598289	0.007109	3465	tags=40%, list=27%, signal=29%
BP	GO:0044283	small molecule biosynthetic process	418	-0.213353194	-1.333719663	0.000141159	0.008772586	0.007253	2073	tags=25%, list=16%, signal=21%
BP	GO:0006637	acyl-CoA metabolic process	64	-0.395719014	-1.980328987	0.000144914	0.008832798	0.007303	2800	tags=48%, list=22%, signal=38%
BP	GO:0035383	thioester metabolic process	64	-0.395719014	-1.980328987	0.000144914	0.008832798	0.007303	2800	tags=48%, list=22%, signal=38%
CC	GO:0031252	cell leading edge	262	0.336649336	1.580683316	0.00014888	0.008988116	0.007431	3192	tags=37%, list=25%, signal=28%
BP	GO:0001944	vasculature development	436	0.30517235	1.471354641	0.000159811	0.00955698	0.007902	3783	tags=39%, list=30%, signal=28%
BP	GO:0007088	regulation of mitotic nuclear division	93	0.424449809	1.775754377	0.000162482	0.009625909	0.007959	2551	tags=34%, list=20%, signal=28%
CC	GO:0005684	U2-type spliceosomal complex	70	0.453907913	1.830265687	0.000165572	0.009718152	0.008035	3095	tags=44%, list=25%, signal=34%
BP	GO:0051783	regulation of nuclear division	101	0.420601842	1.775175023	0.000167298	0.009729368	0.008044	2551	tags=34%, list=20%, signal=27%
MF	GO:0019901	protein kinase binding	414	0.312307528	1.501558383	0.000171481	0.009882005	0.00817	3520	tags=39%, list=28%, signal=29%
CC	GO:0005911	cell-cell junction	274	0.334168271	1.571415562	0.000185626	0.010600732	0.008765	2085	tags=31%, list=17%, signal=26%

CC	GO:0000784	nuclear chromosome, telomeric region	65	0.471429126	1.873966415	0.000199686	0.011256769	0.009307	2323	tags=38%, list=18%, signal=32%
CC	GO:0030117	membrane coat	65	0.471134066	1.872793528	0.000202441	0.011256769	0.009307	2754	tags=40%, list=22%, signal=31%
CC	GO:0048475	coated membrane	65	0.471134066	1.872793528	0.000202441	0.011256769	0.009307	2754	tags=40%, list=22%, signal=31%
BP	GO:0050873	brown fat cell differentiation	30	-0.535356045	-2.209123875	0.000206708	0.011278155	0.009325	1883	tags=47%, list=15%, signal=40%
BP	GO:0000086	G2/M transition of mitotic cell cycle	171	0.366440811	1.647050038	0.000207535	0.011278155	0.009325	3241	tags=39%, list=26%, signal=30%
BP	GO:1903046	meiotic cell cycle process	70	0.450641216	1.817093583	0.000208647	0.011278155	0.009325	2090	tags=31%, list=17%, signal=26%
BP	GO:0046949	fatty-acyl-CoA biosynthetic process	22	-0.575870562	-2.15226826	0.000209942	0.011278155	0.009325	2320	tags=68%, list=18%, signal=56%
MF	GO:0019900	kinase binding	469	0.303442465	1.464119833	0.000214345	0.011417911	0.00944	3612	tags=39%, list=29%, signal=29%
MF	GO:0003729	mRNA binding	205	0.355355489	1.629343199	0.000225284	0.011900652	0.009839	3526	tags=37%, list=28%, signal=27%
BP	GO:0048812	neuron projection morphogenesis	362	0.313440052	1.495282594	0.000249276	0.013059198	0.010797	2600	tags=31%, list=21%, signal=26%
BP	GO:0031589	cell-substrate adhesion	218	0.348180561	1.600863202	0.000252616	0.01312569	0.010852	2543	tags=35%, list=20%, signal=29%
BP	GO:0006986	response to unfolded protein	138	0.38516872	1.695540339	0.000260305	0.013415207	0.011092	2702	tags=35%, list=21%, signal=28%
BP	GO:0048667	cell morphogenesis involved in neuron differentiation	331	0.321269956	1.524944585	0.000268462	0.013724026	0.011347	3079	tags=36%, list=24%, signal=28%
BP	GO:0031145	anaphase-promoting complex-dependent catabolic process	62	0.463007471	1.81631969	0.000273184	0.013811753	0.011419	2969	tags=44%, list=24%, signal=33%
CC	GO:0015629	actin cytoskeleton	302	0.322713614	1.522911038	0.000274536	0.013811753	0.011419	3436	tags=39%, list=27%, signal=29%
BP	GO:0006690	icosanoid metabolic process	59	-0.385036375	-1.872821189	0.000282329	0.014091995	0.011651	2049	tags=39%, list=16%, signal=33%
BP	GO:0120039	plasma membrane bounded cell projection morphogenesis	374	0.313722162	1.498333435	0.000286258	0.014176477	0.011721	2600	tags=32%, list=21%, signal=26%
MF	GO:0016757	transferase activity, transferring glycosyl groups	142	0.377980749	1.670008779	0.000291002	0.014299688	0.011823	3425	tags=42%, list=27%, signal=31%
BP	GO:0099003	vesicle-mediated transport in synapse	104	0.408695962	1.73802401	0.00031148	0.01507579	0.012465	2642	tags=36%, list=21%, signal=28%
BP	GO:0048285	organelle fission	229	0.335939303	1.556961338	0.000311552	0.01507579	0.012465	2730	tags=29%, list=22%, signal=23%
BP	GO:0033044	regulation of chromosome organization	223	0.336948516	1.556774654	0.000327186	0.015712366	0.012991	3711	tags=37%, list=29%, signal=27%
MF	GO:0016903	oxidoreductase activity, acting on the aldehyde or oxo group of donors	31	-0.507873652	-2.092263179	0.000335764	0.015917377	0.01316	2897	tags=58%, list=23%, signal=45%
CC	GO:0099572	postsynaptic specialization	179	0.350356776	1.583881156	0.000338701	0.015917377	0.01316	2691	tags=35%, list=21%, signal=28%
BP	GO:0032799	low-density lipoprotein receptor particle metabolic process	16	0.689532427	1.966403107	0.000338988	0.015917377	0.01316	2265	tags=56%, list=18%, signal=46%
BP	GO:0008610	lipid biosynthetic process	409	-0.207981415	-1.339851034	0.000344166	0.016041679	0.013263	2326	tags=27%, list=18%, signal=23%
BP	GO:0022604	regulation of cell morphogenesis	296	0.318904857	1.505564593	0.000348545	0.016127194	0.013334	3079	tags=37%, list=24%, signal=29%
BP	GO:0002429	immune response-activating cell surface receptor signaling pathway	229	0.33479693	1.551666836	0.00035436	0.016160343	0.013361	2914	tags=35%, list=23%, signal=27%
BP	GO:0002757	immune response-activating signal transduction	229	0.33479693	1.551666836	0.00035436	0.016160343	0.013361	2914	tags=35%, list=23%, signal=27%
MF	GO:0004029	aldehyde dehydrogenase (NAD+) activity	12	-0.725634678	-2.253528732	0.000366402	0.016472495	0.013619	2897	tags=75%, list=23%, signal=58%
MF	GO:0004030	aldehyde dehydrogenase [NAD(P)+] activity	12	-0.725634678	-2.253528732	0.000366402	0.016472495	0.013619	2897	tags=75%, list=23%, signal=58%
BP	GO:0061982	meiosis I cell cycle process	39	0.531276192	1.925066716	0.000372553	0.016555381	0.013688	2090	tags=36%, list=17%, signal=30%
BP	GO:0061564	axon development	287	0.32327444	1.522937473	0.000373469	0.016555381	0.013688	2600	tags=33%, list=21%, signal=27%
BP	GO:0002479	antigen processing and presentation of exogenous peptide antigen via MHC class I, TAP-dependent	67	0.451438826	1.801211046	0.000378284	0.016652381	0.013768	4092	tags=54%, list=32%, signal=36%
BP	GO:0010638	positive regulation of organelle organization	405	0.306960364	1.473630837	0.000382442	0.016719295	0.013823	3532	tags=37%, list=28%, signal=28%
BP	GO:0098813	nuclear chromosome segregation	135	0.376587347	1.65142344	0.000393491	0.017084521	0.014125	4406	tags=45%, list=35%, signal=30%
BP	GO:0000070	mitotic sister chromatid segregation	94	0.41067879	1.721164651	0.000398349	0.0171778	0.014203	2730	tags=35%, list=22%, signal=28%
BP	GO:0000387	spliceosomal snRNP assembly	28	0.565730475	1.895554401	0.000407055	0.017434608	0.014415	3021	tags=50%, list=24%, signal=38%
BP	GO:0072331	signal transduction by p53 class mediator	196	0.341808752	1.562957527	0.000420579	0.017892968	0.014794	3497	tags=40%, list=28%, signal=29%

BP	GO:0016125	sterol metabolic process	97	-0.309829724	-1.649174868	0.000429491	0.018150295	0.015007	1747	tags=29%, list=14%, signal=25%
MF	GO:0003779	actin binding	242	0.332054643	1.546626203	0.000434766	0.01825154	0.01509	2760	tags=35%, list=22%, signal=28%
BP	GO:0046942	carboxylic acid transport	151	-0.280816499	-1.579716819	0.000440442	0.018368178	0.015187	2782	tags=36%, list=22%, signal=29%
BP	GO:0051656	establishment of organelle localization	268	0.325861435	1.530511239	0.000443934	0.018392791	0.015207	4426	tags=45%, list=35%, signal=30%
BP	GO:0007059	chromosome segregation	173	0.357094028	1.608925068	0.000456636	0.018796221	0.015541	4499	tags=43%, list=36%, signal=28%
BP	GO:0006898	receptor-mediated endocytosis	145	0.368751853	1.631762004	0.000471104	0.019113529	0.015803	2368	tags=31%, list=19%, signal=25%
BP	GO:1901796	regulation of signal transduction by p53 class mediator	135	0.375189256	1.645292486	0.000473692	0.019113529	0.015803	3113	tags=41%, list=25%, signal=31%
BP	GO:0007409	axonogenesis	262	0.325084402	1.526382014	0.000478295	0.019113529	0.015803	2600	tags=33%, list=21%, signal=27%
BP	GO:0006692	prostanoid metabolic process	34	-0.4741323	-2.004696275	0.000479421	0.019113529	0.015803	2907	tags=59%, list=23%, signal=45%
BP	GO:0006693	prostaglandin metabolic process	34	-0.4741323	-2.004696275	0.000479421	0.019113529	0.015803	2907	tags=59%, list=23%, signal=45%
BP	GO:0048524	positive regulation of viral process	78	0.429573518	1.759056724	0.000482823	0.019128862	0.015816	3520	tags=45%, list=28%, signal=33%
BP	GO:0051383	kinetochore organization	13	0.70489649	1.894247021	0.000494498	0.019433448	0.016067	1442	tags=38%, list=11%, signal=34%
BP	GO:0032989	cellular component morphogenesis	435	0.297224189	1.43236436	0.000497458	0.019433448	0.016067	2600	tags=30%, list=21%, signal=25%
BP	GO:0007127	meiosis I	37	0.526747096	1.889323749	0.000500352	0.019433448	0.016067	2090	tags=35%, list=17%, signal=29%
BP	GO:0032091	negative regulation of protein binding	64	0.450821759	1.77852601	0.000502774	0.019433448	0.016067	3348	tags=52%, list=27%, signal=38%
BP	GO:0000819	sister chromatid segregation	114	0.38726644	1.669075109	0.000524037	0.020132564	0.016646	2730	tags=32%, list=22%, signal=26%
BP	GO:0009914	hormone transport	150	-0.272487731	-1.531101893	0.000551154	0.021046797	0.017401	2303	tags=30%, list=18%, signal=25%
MF	GO:0003682	chromatin binding	360	0.302653492	1.4430933	0.000557771	0.021102166	0.017447	3531	tags=34%, list=28%, signal=26%
BP	GO:0009100	glycoprotein metabolic process	231	0.327579308	1.520669998	0.000559262	0.021102166	0.017447	3436	tags=36%, list=27%, signal=27%
CC	GO:0120114	Sm-like protein family complex	57	0.453815517	1.756784293	0.000590936	0.022132523	0.018299	3021	tags=40%, list=24%, signal=31%
BP	GO:0006694	steroid biosynthetic process	102	-0.309121164	-1.652283314	0.000593552	0.022132523	0.018299	2968	tags=42%, list=24%, signal=32%
BP	GO:0006261	DNA-dependent DNA replication	88	0.416419451	1.734950378	0.000618607	0.022931883	0.01896	3944	tags=51%, list=31%, signal=35%
BP	GO:0015718	monocarboxylic acid transport	78	-0.332486291	-1.710355984	0.00063373	0.023340529	0.019298	2624	tags=40%, list=21%, signal=32%
BP	GO:0006869	lipid transport	181	-0.255931412	-1.484838105	0.000636995	0.023340529	0.019298	2657	tags=35%, list=21%, signal=28%
CC	GO:0071013	catalytic step 2 spliceosome	67	0.442726326	1.766448747	0.000649313	0.023591202	0.019505	3055	tags=42%, list=24%, signal=32%
CC	GO:1904949	ATPase complex	66	0.44836114	1.785704847	0.000652473	0.023591202	0.019505	4108	tags=53%, list=33%, signal=36%
BP	GO:0072189	ureter development	11	-0.72235014	-2.189936994	0.00065857	0.023591202	0.019505	1497	tags=55%, list=12%, signal=48%
BP	GO:0120162	positive regulation of cold-induced thermogenesis	55	-0.384822429	-1.829818286	0.000658723	0.023591202	0.019505	2948	tags=60%, list=23%, signal=46%
CC	GO:0000775	chromosome, centromeric region	112	0.383389015	1.641862249	0.000664427	0.023661803	0.019563	3939	tags=42%, list=31%, signal=29%
CC	GO:0019867	outer membrane	139	-0.278434306	-1.530283662	0.000676209	0.023813811	0.019689	1926	tags=27%, list=15%, signal=23%
CC	GO:0031968	organelle outer membrane	139	-0.278434306	-1.530283662	0.000676209	0.023813811	0.019689	1926	tags=27%, list=15%, signal=23%
BP	GO:0090287	regulation of cellular response to growth factor stimulus	161	0.352371651	1.576546225	0.000685876	0.024020832	0.01986	2770	tags=34%, list=22%, signal=27%
BP	GO:0002474	antigen processing and presentation of peptide antigen via MHC class I	86	0.42051896	1.739627319	0.000705649	0.024577536	0.020321	4092	tags=48%, list=32%, signal=32%
BP	GO:0032956	regulation of actin cytoskeleton organization	222	0.329243013	1.52067222	0.000733634	0.025115823	0.020766	3441	tags=41%, list=27%, signal=30%
BP	GO:0006302	double-strand break repair	131	0.378012511	1.648662688	0.000733646	0.025115823	0.020766	4124	tags=40%, list=33%, signal=28%
CC	GO:0070603	SWI/SNF superfamily-type complex	63	0.456506624	1.79748037	0.000736819	0.025115823	0.020766	4263	tags=56%, list=34%, signal=37%
MF	GO:0017124	SH3 domain binding	81	0.420277448	1.722027388	0.000740579	0.025115823	0.020766	3246	tags=44%, list=26%, signal=33%
BP	GO:0070534	protein K63-linked ubiquitination	43	0.486148701	1.792673827	0.000740915	0.025115823	0.020766	3746	tags=53%, list=30%, signal=38%
BP	GO:0051983	regulation of chromosome segregation	61	0.456277	1.783477262	0.000745047	0.025121548	0.02077	2076	tags=33%, list=16%, signal=28%

BP	GO:0050686	negative regulation of mRNA processing	18	0.627408428	1.85276132	0.000760755	0.025515496	0.021096	1087	tags=39%, list=9%, signal=36%
CC	GO:0098978	glutamatergic synapse	165	0.354866839	1.592997881	0.000769722	0.025680363	0.021232	2676	tags=35%, list=21%, signal=28%
MF	GO:0046875	ephrin receptor binding	22	0.584327947	1.861740523	0.000777705	0.02581085	0.02134	3247	tags=64%, list=26%, signal=47%
CC	GO:0045120	pronucleus	12	0.705571288	1.876241383	0.00078526	0.025892021	0.021407	779	tags=33%, list=6%, signal=31%
BP	GO:0034381	plasma lipoprotein particle clearance	26	0.565208636	1.846370931	0.000789686	0.025892021	0.021407	2265	tags=50%, list=18%, signal=41%
CC	GO:0001533	cornified envelope	11	-0.716702907	-2.172816371	0.000794192	0.025892021	0.021407	1817	tags=64%, list=14%, signal=55%
BP	GO:0071168	protein localization to chromatin	15	0.662172947	1.852872822	0.000800387	0.025892021	0.021407	3691	tags=80%, list=29%, signal=57%
BP	GO:0097006	regulation of plasma lipoprotein particle levels	45	0.493107621	1.831966201	0.000800574	0.025892021	0.021407	3902	tags=58%, list=31%, signal=40%
BP	GO:0034502	protein localization to chromosome	48	0.480826015	1.800033572	0.000811384	0.026108446	0.021586	3900	tags=52%, list=31%, signal=36%
BP	GO:0042445	hormone metabolic process	91	-0.325512582	-1.729400863	0.000817534	0.026173475	0.02164	1982	tags=31%, list=16%, signal=26%
BP	GO:0030111	regulation of Wnt signaling pathway	221	0.32933538	1.521321815	0.000829533	0.026424169	0.021847	4311	tags=45%, list=34%, signal=30%
BP	GO:0046165	alcohol biosynthetic process	97	-0.3007997	-1.601109475	0.000835228	0.026472547	0.021887	1777	tags=29%, list=14%, signal=25%
BP	GO:0036498	IRE1-mediated unfolded protein response	56	0.456319814	1.765870556	0.000843368	0.026545977	0.021948	3311	tags=48%, list=26%, signal=36%
BP	GO:0019941	modification-dependent protein catabolic process	453	0.289228199	1.394470266	0.000849062	0.026545977	0.021948	3529	tags=34%, list=28%, signal=25%
BP	GO:0006897	endocytosis	318	0.310081256	1.46884023	0.000850108	0.026545977	0.021948	2534	tags=29%, list=20%, signal=24%
BP	GO:0140014	mitotic nuclear division	162	0.354888995	1.588249129	0.000861656	0.026683725	0.022062	2730	tags=31%, list=22%, signal=24%
BP	GO:0006890	retrograde vesicle-mediated transport, Golgi to endoplasmic reticulum	68	0.421970289	1.693199889	0.000862938	0.026683725	0.022062	2754	tags=41%, list=22%, signal=32%
BP	GO:0050684	regulation of mRNA processing	92	0.401337823	1.680151142	0.000886382	0.027275603	0.022551	4314	tags=48%, list=34%, signal=32%
BP	GO:0071559	response to transforming growth factor beta	160	0.355984843	1.594849739	0.000898151	0.027489276	0.022728	2788	tags=34%, list=22%, signal=27%
BP	GO:0010564	regulation of cell cycle process	470	0.292243644	1.410571519	0.000901999	0.027489276	0.022728	3868	tags=37%, list=31%, signal=27%
BP	GO:0007265	Ras protein signal transduction	223	0.325825743	1.50538505	0.000926401	0.028097868	0.023231	3376	tags=38%, list=27%, signal=28%
MF	GO:0001091	RNA polymerase II general transcription initiation factor binding	17	0.640886445	1.859862535	0.000943187	0.028470773	0.023539	1822	tags=41%, list=14%, signal=35%
BP	GO:0031532	actin cytoskeleton reorganization	69	0.429330309	1.729336786	0.000953866	0.028656671	0.023693	3044	tags=43%, list=24%, signal=33%
BP	GO:0010876	lipid localization	209	-0.245768076	-1.41874494	0.000970975	0.029033057	0.024004	2657	tags=35%, list=21%, signal=28%
BP	GO:0035904	aorta development	34	0.534286454	1.88570145	0.000991971	0.029037669	0.024008	2912	tags=56%, list=23%, signal=43%
BP	GO:0035909	aorta morphogenesis	18	0.620483224	1.832310925	0.000994829	0.029037669	0.024008	2491	tags=61%, list=20%, signal=49%
BP	GO:0003158	endothelium development	80	0.415165477	1.698343821	0.00099887	0.029037669	0.024008	2939	tags=46%, list=23%, signal=36%
BP	GO:0044070	regulation of anion transport	55	-0.379667063	-1.805304684	0.001001916	0.029037669	0.024008	1764	tags=36%, list=14%, signal=31%
BP	GO:0006112	energy reserve metabolic process	49	-0.405372596	-1.888915813	0.001004792	0.029037669	0.024008	1705	tags=37%, list=14%, signal=32%
MF	GO:0008201	heparin binding	69	-0.350056852	-1.762076824	0.001007901	0.029037669	0.024008	2358	tags=45%, list=19%, signal=37%
BP	GO:0016055	Wnt signaling pathway	310	0.306020944	1.448364983	0.001011109	0.029037669	0.024008	4041	tags=40%, list=32%, signal=28%
BP	GO:0198738	cell-cell signaling by wnt	310	0.306020944	1.448364983	0.001011109	0.029037669	0.024008	4041	tags=40%, list=32%, signal=28%
BP	GO:0035239	tube morphogenesis	491	0.286456379	1.385144983	0.001012356	0.029037669	0.024008	2648	tags=29%, list=21%, signal=24%
BP	GO:0019216	regulation of lipid metabolic process	237	-0.236321424	-1.429718998	0.001027878	0.029350094	0.024267	2200	tags=28%, list=17%, signal=23%
BP	GO:0007015	actin filament organization	259	0.322128679	1.510068848	0.001039604	0.029551784	0.024433	3325	tags=40%, list=26%, signal=30%
BP	GO:1903844	regulation of cellular response to transforming growth factor beta stimulus	79	0.420552621	1.717351423	0.001054105	0.029830218	0.024663	2551	tags=37%, list=20%, signal=29%
MF	GO:0008134	transcription factor binding	425	0.294667586	1.417482252	0.001073491	0.030184344	0.024956	4091	tags=40%, list=32%, signal=28%
CC	GO:0005905	clathrin-coated pit	44	0.493201508	1.830251232	0.001076142	0.030184344	0.024956	3731	tags=55%, list=30%, signal=39%
BP	GO:0060840	artery development	54	0.456115385	1.755853526	0.001094431	0.030562107	0.025269	2912	tags=48%, list=23%, signal=37%

BP	GO:0017015	regulation of transforming growth factor beta receptor signaling pathway	77	0.423055507	1.727214964	0.001116445	0.031040119	0.025664	2551	tags=36%, list=20%, signal=29%
CC	GO:0099513	polymeric cytoskeletal fiber	322	0.303700827	1.438710675	0.001123349	0.031095667	0.02571	3827	tags=41%, list=30%, signal=29%
CC	GO:0030135	coated vesicle	191	0.335765545	1.528314408	0.00114312	0.03150538	0.026048	2889	tags=33%, list=23%, signal=26%
MF	GO:0019887	protein kinase regulator activity	117	0.375371611	1.619597535	0.001158075	0.031779389	0.026275	2643	tags=34%, list=21%, signal=27%
BP	GO:0019369	arachidonic acid metabolic process	25	-0.494830341	-1.949888371	0.001181101	0.032174701	0.026602	1982	tags=44%, list=16%, signal=37%
CC	GO:0005884	actin filament	73	0.42661846	1.728527001	0.001184266	0.032174701	0.026602	3421	tags=47%, list=27%, signal=34%
MF	GO:0031490	chromatin DNA binding	57	0.444476849	1.720632984	0.001187708	0.032174701	0.026602	3417	tags=53%, list=27%, signal=39%
BP	GO:0018212	peptidyl-tyrosine modification	206	0.331179901	1.520446682	0.001204673	0.032387299	0.026778	3512	tags=40%, list=28%, signal=29%
BP	GO:0033209	tumor necrosis factor-mediated signaling pathway	116	0.375757246	1.615998284	0.001205774	0.032387299	0.026778	4943	tags=59%, list=39%, signal=36%
BP	GO:0051493	regulation of cytoskeleton organization	339	0.302039006	1.434113579	0.001235359	0.033041953	0.027319	2939	tags=32%, list=23%, signal=25%
BP	GO:0006270	DNA replication initiation	24	0.570296715	1.846662057	0.001256497	0.033359089	0.027581	3917	tags=71%, list=31%, signal=49%
BP	GO:0034383	low-density lipoprotein particle clearance	20	0.620434852	1.914345718	0.001262543	0.033359089	0.027581	2265	tags=60%, list=18%, signal=49%
BP	GO:0032365	intracellular lipid transport	24	-0.520095012	-2.024844605	0.001264326	0.033359089	0.027581	1931	tags=46%, list=15%, signal=39%
BP	GO:0006397	mRNA processing	341	0.302328774	1.435516601	0.001270479	0.033359089	0.027581	4340	tags=40%, list=34%, signal=27%
BP	GO:1905818	regulation of chromosome separation	40	0.4957599	1.81439638	0.001277946	0.033359089	0.027581	1882	tags=35%, list=15%, signal=30%
BP	GO:0007567	parturition	12	-0.681726934	-2.117168985	0.001279789	0.033359089	0.027581	1520	tags=42%, list=12%, signal=37%
BP	GO:1905114	cell surface receptor signaling pathway involved in cell-cell signaling	348	0.300713978	1.431810445	0.001284054	0.033359089	0.027581	4178	tags=41%, list=33%, signal=28%
BP	GO:0006511	ubiquitin-dependent protein catabolic process	447	0.28675342	1.381921034	0.001296206	0.033537341	0.027729	4041	tags=38%, list=32%, signal=27%
BP	GO:0007179	transforming growth factor beta receptor signaling pathway	126	0.370287233	1.610306159	0.001333642	0.034365679	0.028413	2788	tags=36%, list=22%, signal=28%
MF	GO:0008194	UDP-glycosyltransferase activity	63	0.446436449	1.757829374	0.001367871	0.03510499	0.029025	3252	tags=48%, list=26%, signal=36%
BP	GO:0046883	regulation of hormone secretion	122	-0.289312847	-1.580211351	0.001385732	0.035419981	0.029285	2837	tags=38%, list=23%, signal=30%
BP	GO:0046456	icosanoid biosynthetic process	32	-0.438933978	-1.818493258	0.001397598	0.035579824	0.029417	2907	tags=53%, list=23%, signal=41%
BP	GO:0034330	cell junction organization	354	0.297722851	1.417856128	0.001419601	0.035995412	0.029761	2600	tags=32%, list=21%, signal=26%
BP	GO:0051098	regulation of binding	240	0.32451331	1.509693835	0.001434465	0.036227384	0.029953	3697	tags=40%, list=29%, signal=29%
CC	GO:0005811	lipid droplet	63	-0.353249543	-1.748031453	0.00145139	0.036342875	0.030048	2052	tags=38%, list=16%, signal=32%
BP	GO:0043632	modification-dependent macromolecule catabolic process	461	0.287588941	1.387860293	0.001461099	0.036342875	0.030048	3529	tags=34%, list=28%, signal=25%
BP	GO:0006625	protein targeting to peroxisome	50	-0.378538278	-1.756899235	0.001467704	0.036342875	0.030048	2884	tags=36%, list=23%, signal=28%
BP	GO:0072662	protein localization to peroxisome	50	-0.378538278	-1.756899235	0.001467704	0.036342875	0.030048	2884	tags=36%, list=23%, signal=28%
BP	GO:0072663	establishment of protein localization to peroxisome	50	-0.378538278	-1.756899235	0.001467704	0.036342875	0.030048	2884	tags=36%, list=23%, signal=28%
MF	GO:0003688	DNA replication origin binding	13	0.671689183	1.805010027	0.001481205	0.036534476	0.030207	3917	tags=92%, list=31%, signal=64%
BP	GO:0002768	immune response-regulating cell surface receptor signaling pathway	254	0.321071429	1.500823234	0.001500507	0.036867098	0.030482	2914	tags=33%, list=23%, signal=26%
CC	GO:0005912	adherens junction	95	0.398602595	1.674121171	0.001525035	0.037325074	0.03086	3664	tags=51%, list=29%, signal=36%
MF	GO:0051287	NAD binding	44	-0.414544176	-1.839706502	0.001550116	0.037674988	0.031149	1520	tags=30%, list=12%, signal=26%
BP	GO:0045664	regulation of neuron differentiation	363	0.29273613	1.397233495	0.001551218	0.037674988	0.031149	3017	tags=34%, list=24%, signal=26%
BP	GO:0007411	axon guidance	149	0.355113415	1.575111515	0.001595768	0.038416413	0.031762	3054	tags=38%, list=24%, signal=29%
BP	GO:0097485	neuron projection guidance	149	0.355113415	1.575111515	0.001595768	0.038416413	0.031762	3054	tags=38%, list=24%, signal=29%
BP	GO:0006333	chromatin assembly or disassembly	88	0.39998795	1.666490945	0.001604624	0.038416413	0.031762	4467	tags=50%, list=35%, signal=33%
BP	GO:0120035	regulation of plasma membrane bounded cell projection organization	384	0.297068453	1.422193628	0.001605987	0.038416413	0.031762	3295	tags=35%, list=26%, signal=27%
MF	GO:0042826	histone deacetylase binding	85	0.411283439	1.699532674	0.001625245	0.038730921	0.032023	3675	tags=45%, list=29%, signal=32%

BP	GO:0018108	peptidyl-tyrosine phosphorylation	203	0.327305331	1.500221339	0.001638996	0.038912352	0.032173	3512	tags=39%, list=28%, signal=29%
MF	GO:0019904	protein domain specific binding	444	0.288538628	1.390415442	0.001689569	0.039742885	0.032859	2898	tags=30%, list=23%, signal=24%
BP	GO:0033045	regulation of sister chromatid segregation	51	0.467456749	1.774144727	0.001703993	0.039742885	0.032859	2354	tags=35%, list=19%, signal=29%
CC	GO:0097525	spliceosomal snRNP complex	45	0.478600446	1.778069946	0.001704404	0.039742885	0.032859	3021	tags=47%, list=24%, signal=36%
BP	GO:0006695	cholesterol biosynthetic process	51	-0.384618474	-1.790404557	0.001705326	0.039742885	0.032859	3378	tags=49%, list=27%, signal=36%
BP	GO:1902653	secondary alcohol biosynthetic process	51	-0.384618474	-1.790404557	0.001705326	0.039742885	0.032859	3378	tags=49%, list=27%, signal=36%
BP	GO:0007178	transmembrane receptor protein serine/threonine kinase signaling pathway	192	0.330128637	1.50539172	0.001722019	0.039984904	0.033059	2788	tags=33%, list=22%, signal=26%
BP	GO:0031344	regulation of cell projection organization	388	0.294648172	1.411985898	0.001733934	0.040003862	0.033075	3295	tags=35%, list=26%, signal=27%
CC	GO:0000779	condensed chromosome, centromeric region	64	0.426880808	1.684077141	0.001736005	0.040003862	0.033075	3857	tags=45%, list=31%, signal=32%
BP	GO:0001667	ameboidal-type cell migration	267	0.314633576	1.476826569	0.001741768	0.040003862	0.033075	3578	tags=39%, list=28%, signal=29%
MF	GO:0016620	oxidoreductase activity, acting on the aldehyde or oxo group of donors, NAD or NADP as acceptor	27	-0.495272334	-2.011371214	0.001773962	0.04059619	0.033565	2897	tags=56%, list=23%, signal=43%
BP	GO:0071103	DNA conformation change	171	0.339004667	1.523732163	0.001783535	0.040668437	0.033624	4243	tags=42%, list=34%, signal=28%
CC	GO:0014069	postsynaptic density	171	0.33855058	1.521691169	0.001803242	0.040838453	0.033765	2691	tags=34%, list=21%, signal=27%
BP	GO:0008608	attachment of spindle microtubules to kinetochore	13	0.666459572	1.790956652	0.001807334	0.040838453	0.033765	1569	tags=38%, list=12%, signal=34%
BP	GO:1902622	regulation of neutrophil migration	22	0.563757803	1.796201522	0.001817247	0.040838453	0.033765	2311	tags=41%, list=18%, signal=33%
BP	GO:0040020	regulation of meiotic nuclear division	12	0.685003362	1.821547556	0.001821082	0.040838453	0.033765	962	tags=42%, list=8%, signal=39%
MF	GO:0004497	monooxygenase activity	31	-0.463135371	-1.907956989	0.001823203	0.040838453	0.033765	3042	tags=61%, list=24%, signal=47%
BP	GO:0005977	glycogen metabolic process	44	-0.40949033	-1.817278026	0.001846313	0.040922308	0.033834	1526	tags=36%, list=12%, signal=32%
BP	GO:0006073	cellular glucan metabolic process	44	-0.40949033	-1.817278026	0.001846313	0.040922308	0.033834	1526	tags=36%, list=12%, signal=32%
BP	GO:0044042	glucan metabolic process	44	-0.40949033	-1.817278026	0.001846313	0.040922308	0.033834	1526	tags=36%, list=12%, signal=32%
BP	GO:1902749	regulation of cell cycle G2/M phase transition	142	0.355536819	1.570846165	0.001858732	0.041016275	0.033912	4041	tags=44%, list=32%, signal=30%
BP	GO:0051304	chromosome separation	53	0.448743271	1.72033138	0.001863494	0.041016275	0.033912	1882	tags=30%, list=15%, signal=26%
BP	GO:1903902	positive regulation of viral life cycle	43	0.469754016	1.732218408	0.00187656	0.041160943	0.034032	3082	tags=49%, list=24%, signal=37%
BP	GO:0002764	immune response-regulating signaling pathway	256	0.314519096	1.469451339	0.001905865	0.041546378	0.03435	2914	tags=33%, list=23%, signal=26%
BP	GO:0007160	cell-matrix adhesion	136	0.358246236	1.57174064	0.00190724	0.041546378	0.03435	2543	tags=37%, list=20%, signal=30%
MF	GO:0016298	lipase activity	54	-0.362804819	-1.720740882	0.001930727	0.041751494	0.03452	1545	tags=28%, list=12%, signal=24%
BP	GO:1903510	mucopolysaccharide metabolic process	68	0.405388945	1.626665512	0.001933244	0.041751494	0.03452	2393	tags=40%, list=19%, signal=32%
BP	GO:0071560	cellular response to transforming growth factor beta stimulus	155	0.350218622	1.560758898	0.001942259	0.041751494	0.03452	2788	tags=34%, list=22%, signal=27%
BP	GO:0043687	post-translational protein modification	234	0.31982341	1.486045973	0.001950256	0.041751494	0.03452	4801	tags=51%, list=38%, signal=32%
BP	GO:0010389	regulation of G2/M transition of mitotic cell cycle	135	0.356619197	1.563858442	0.001954025	0.041751494	0.03452	2969	tags=36%, list=24%, signal=28%
BP	GO:0051701	interaction with host	134	0.356852085	1.561698723	0.001956175	0.041751494	0.03452	3287	tags=40%, list=26%, signal=30%
BP	GO:0009101	glycoprotein biosynthetic process	189	0.34073878	1.548329701	0.001989676	0.042324007	0.034993	3279	tags=35%, list=26%, signal=27%
BP	GO:0048193	Golgi vesicle transport	269	0.309034575	1.451391174	0.002004499	0.042393114	0.03505	2765	tags=30%, list=22%, signal=24%
BP	GO:0048024	regulation of mRNA splicing, via spliceosome	66	0.427049964	1.700828022	0.0020063	0.042393114	0.03505	4314	tags=53%, list=34%, signal=35%
BP	GO:0006310	DNA recombination	138	0.356922747	1.57119954	0.00203293	0.04281309	0.035398	3480	tags=35%, list=28%, signal=25%
BP	GO:0010965	regulation of mitotic sister chromatid separation	36	0.502644797	1.802018459	0.002059585	0.043230833	0.035743	1882	tags=36%, list=15%, signal=31%
BP	GO:0030258	lipid modification	149	-0.263397136	-1.4862402	0.002066901	0.0432412	0.035752	2640	tags=33%, list=21%, signal=26%
BP	GO:0002253	activation of immune response	274	0.311035637	1.462635095	0.002076577	0.043300725	0.035801	2914	tags=33%, list=23%, signal=26%

CC	GO:0030120	vesicle coat	44	0.478854572	1.77701032	0.002091214	0.043424486	0.035903	2754	tags=41%, list=22%, signal=32%
BP	GO:0045070	positive regulation of viral genome replication	28	0.530099334	1.776167575	0.002096213	0.043424486	0.035903	3082	tags=54%, list=24%, signal=41%
BP	GO:0070268	cornification	17	-0.585705952	-2.037125823	0.002105365	0.043472016	0.035942	1614	tags=53%, list=13%, signal=46%
BP	GO:0034655	nucleobase-containing compound catabolic process	390	0.288549471	1.38155918	0.002133029	0.043788465	0.036204	3547	tags=35%, list=28%, signal=26%
CC	GO:0005667	transcription regulator complex	256	0.313564692	1.464992313	0.00213666	0.043788465	0.036204	4823	tags=49%, list=38%, signal=31%
CC	GO:0005741	mitochondrial outer membrane	123	-0.280667004	-1.533489197	0.002147155	0.043788465	0.036204	1341	tags=24%, list=11%, signal=21%
BP	GO:0030511	positive regulation of transforming growth factor beta receptor signaling pathway	23	0.566541219	1.821785676	0.00215523	0.043788465	0.036204	2342	tags=48%, list=19%, signal=39%
BP	GO:1903846	positive regulation of cellular response to transforming growth factor beta stimulus	23	0.566541219	1.821785676	0.00215523	0.043788465	0.036204	2342	tags=48%, list=19%, signal=39%
BP	GO:0002831	regulation of response to biotic stimulus	253	0.309462643	1.445490057	0.002170645	0.043960758	0.036347	3425	tags=34%, list=27%, signal=26%
BP	GO:1905039	carboxylic acid transmembrane transport	64	-0.340271782	-1.702849878	0.002196713	0.044347022	0.036666	900	tags=25%, list=7%, signal=23%
MF	GO:0019842	vitamin binding	68	-0.328848741	-1.648478731	0.002212864	0.044531256	0.036818	2673	tags=44%, list=21%, signal=35%
BP	GO:0016358	dendrite development	144	0.348669737	1.544116594	0.002236635	0.044867171	0.037096	2945	tags=38%, list=23%, signal=30%
BP	GO:0008202	steroid metabolic process	163	-0.25149551	-1.44905968	0.00225333	0.044960675	0.037173	1756	tags=37%, list=14%, signal=32%
BP	GO:0006281	DNA repair	331	0.30043766	1.426061708	0.002255481	0.044960675	0.037173	3691	tags=33%, list=29%, signal=24%
BP	GO:0051382	kinetochore assembly	10	0.722813854	1.819964025	0.002286006	0.045426302	0.037558	433	tags=30%, list=3%, signal=29%
MF	GO:0016229	steroid dehydrogenase activity	16	-0.578034678	-1.980039944	0.002329095	0.046137909	0.038147	2492	tags=62%, list=20%, signal=50%
BP	GO:0000209	protein polyubiquitination	248	0.31544908	1.471505751	0.002370108	0.046707349	0.038617	4041	tags=39%, list=32%, signal=27%
BP	GO:0032970	regulation of actin filament-based process	243	0.316703427	1.475689576	0.002372577	0.046707349	0.038617	3441	tags=40%, list=27%, signal=29%
MF	GO:0003725	double-stranded RNA binding	54	0.441659552	1.700204614	0.002384162	0.046766528	0.038666	3030	tags=46%, list=24%, signal=35%
MF	GO:0050681	androgen receptor binding	18	0.598867582	1.768479097	0.002391534	0.046766528	0.038666	2342	tags=50%, list=19%, signal=41%
BP	GO:0030073	insulin secretion	102	-0.289484114	-1.547321333	0.002397716	0.046766528	0.038666	2837	tags=39%, list=23%, signal=31%
BP	GO:0042590	antigen processing and presentation of exogenous peptide antigen via MHC class I	71	0.420994608	1.694891407	0.002411509	0.046891267	0.038769	4092	tags=51%, list=32%, signal=34%
BP	GO:0071826	ribonucleoprotein complex subunit organization	143	0.349361171	1.54340648	0.002451466	0.047522447	0.039291	4340	tags=46%, list=34%, signal=31%
BP	GO:0007346	regulation of mitotic cell cycle	407	0.291555052	1.399646336	0.002467764	0.047536913	0.039303	3447	tags=34%, list=27%, signal=26%
BP	GO:0048025	negative regulation of mRNA splicing, via spliceosome	12	0.675066834	1.795124536	0.002468567	0.047536913	0.039303	1087	tags=50%, list=9%, signal=46%
BP	GO:0000724	double-strand break repair via homologous recombination	70	0.407847596	1.644539432	0.002482208	0.047536913	0.039303	2561	tags=31%, list=20%, signal=25%
BP	GO:0000725	recombinational repair	70	0.407847596	1.644539432	0.002482208	0.047536913	0.039303	2561	tags=31%, list=20%, signal=25%
BP	GO:0090181	regulation of cholesterol metabolic process	43	-0.384707824	-1.713712493	0.002491733	0.047575588	0.039335	1546	tags=42%, list=12%, signal=37%
BP	GO:0032543	mitochondrial translation	111	0.363030918	1.553852222	0.002509115	0.047685718	0.039426	4759	tags=50%, list=38%, signal=31%
BP	GO:0010769	regulation of cell morphogenesis involved in differentiation	186	0.329296515	1.493349038	0.002512546	0.047685718	0.039426	3281	tags=40%, list=26%, signal=30%
CC	GO:0005681	spliceosomal complex	140	0.349577253	1.543041126	0.002521469	0.047712216	0.039448	3095	tags=34%, list=25%, signal=26%
BP	GO:0043254	regulation of protein-containing complex assembly	273	0.305693905	1.437035271	0.002536403	0.047836209	0.039551	4160	tags=44%, list=33%, signal=30%
BP	GO:0044270	cellular nitrogen compound catabolic process	409	0.284983475	1.368759666	0.002543114	0.047836209	0.039551	3547	tags=35%, list=28%, signal=26%
BP	GO:0071622	regulation of granulocyte chemotaxis	26	0.537733653	1.756618215	0.002567846	0.048158516	0.039817	2472	tags=42%, list=20%, signal=34%
BP	GO:0022618	ribonucleoprotein complex assembly	137	0.351025868	1.54299768	0.002610686	0.048611224	0.040192	4340	tags=46%, list=34%, signal=30%
BP	GO:0045787	positive regulation of cell cycle	221	0.314584606	1.45318254	0.002614939	0.048611224	0.040192	3691	tags=38%, list=29%, signal=28%
BP	GO:1905144	response to acetylcholine	13	0.653491964	1.756109191	0.00262266	0.048611224	0.040192	924	tags=31%, list=7%, signal=29%
BP	GO:1905145	cellular response to acetylcholine	13	0.653491964	1.756109191	0.00262266	0.048611224	0.040192	924	tags=31%, list=7%, signal=29%
BP	GO:1902652	secondary alcohol metabolic process	96	-0.306073655	-1.625170182	0.002635947	0.048715062	0.040277	2813	tags=39%, list=22%, signal=30%

CC	GO:0045211	postsynaptic membrane	90	0.385460742	1.611935832	0.002645381	0.048747302	0.040304	3275	tags=38%, list=26%, signal=28%
BP	GO:0006577	amino-acid betaine metabolic process	10	-0.689129066	-2.048931863	0.002679669	0.049236013	0.040708	2056	tags=50%, list=16%, signal=42%
BP	GO:0140013	meiotic nuclear division	58	0.433187954	1.682094513	0.002698095	0.049431289	0.04087	2090	tags=29%, list=17%, signal=25%
BP	GO:0034446	substrate adhesion-dependent cell spreading	72	0.418832081	1.696152484	0.002726394	0.049565377	0.04098	3295	tags=50%, list=26%, signal=37%
MF	GO:0016628	oxidoreductase activity, acting on the CH-CH group of donors, NAD or NADP as acceptor	20	-0.53648713	-1.952056395	0.002728576	0.049565377	0.04098	2165	tags=60%, list=17%, signal=50%
CC	GO:0043197	dendritic spine	84	0.393331462	1.627224694	0.002731409	0.049565377	0.04098	3085	tags=44%, list=24%, signal=33%
CC	GO:0019897	extrinsic component of plasma membrane	82	0.389157692	1.599181095	0.002740959	0.049565377	0.04098	3418	tags=40%, list=27%, signal=30%
MF	GO:0070300	phosphatidic acid binding	15	0.632712449	1.770437324	0.002752279	0.049565377	0.04098	3500	tags=67%, list=28%, signal=48%
BP	GO:0048010	vascular endothelial growth factor receptor signaling pathway	69	0.405431867	1.63307418	0.002752329	0.049565377	0.04098	3267	tags=45%, list=26%, signal=33%
BP	GO:0006935	chemotaxis	322	0.295663056	1.400633642	0.002789026	0.049969894	0.041315	3545	tags=38%, list=28%, signal=28%
BP	GO:0006826	iron ion transport	42	-0.391324763	-1.74181534	0.002791075	0.049969894	0.041315	1491	tags=31%, list=12%, signal=27%
BP	GO:0098693	regulation of synaptic vesicle cycle	57	0.429049187	1.660910312	0.002804305	0.049969894	0.041315	2642	tags=39%, list=21%, signal=31%
BP	GO:0010810	regulation of cell-substrate adhesion	128	0.360107137	1.568050743	0.002806323	0.049969894	0.041315	2491	tags=38%, list=20%, signal=30%

KEGG

ID	Description	setSize	enrichmentScore	NES	pvalue	p.adjust	qvalues	rank	leading_edge
hsa03040	Spliceosome	102	0.465409784	1.978657022	2.51E-06	0.000787245	0.000525	4340	tags=55%, list=34%, signal=36%
hsa00620	Pyruvate metabolism	32	-0.577235001	-2.377598961	8.87E-06	0.000816674	0.000545	1312	tags=41%, list=10%, signal=36%
hsa05135	Yersinia infection	99	0.449479804	1.901272982	9.85E-06	0.000816674	0.000545	2910	tags=46%, list=23%, signal=36%
hsa00280	Valine, leucine and isoleucine degradation	38	-0.530869449	-2.231003198	1.04E-05	0.000816674	0.000545	1293	tags=61%, list=10%, signal=54%
hsa05100	Bacterial invasion of epithelial cells	53	0.523778724	2.023001904	1.71E-05	0.000982581	0.000655	4178	tags=68%, list=33%, signal=46%
hsa00071	Fatty acid degradation	30	-0.567240995	-2.310883394	1.88E-05	0.000982581	0.000655	3557	tags=67%, list=28%, signal=48%
hsa05160	Hepatitis C	106	0.436150639	1.869667313	4.58E-05	0.002052307	0.001369	3439	tags=49%, list=27%, signal=36%
hsa05020	Prion disease	177	0.374821549	1.692490086	6.33E-05	0.002478571	0.001653	3093	tags=40%, list=25%, signal=30%
hsa04810	Regulation of actin cytoskeleton	142	0.391509147	1.732171532	7.42E-05	0.002478571	0.001653	2664	tags=42%, list=21%, signal=34%
hsa04146	Peroxisome	55	-0.422609432	-1.992958706	7.89E-05	0.002478571	0.001653	3557	tags=55%, list=28%, signal=39%
hsa05203	Viral carcinogenesis	127	0.399331419	1.744614409	0.00010202	0.002912212	0.001943	3766	tags=48%, list=30%, signal=34%
hsa01212	Fatty acid metabolism	38	-0.48169609	-2.024349904	0.000140456	0.003675273	0.002452	3371	tags=61%, list=27%, signal=44%
hsa05016	Huntington disease	195	0.359919786	1.640947331	0.000178268	0.004305865	0.002873	4317	tags=45%, list=34%, signal=30%
hsa00640	Propanoate metabolism	28	-0.542094639	-2.139390682	0.000201643	0.004522569	0.003017	3650	tags=64%, list=29%, signal=46%
hsa04141	Protein processing in endoplasmic reticulum	136	0.386110913	1.696499952	0.000245252	0.004759107	0.003175	3792	tags=42%, list=30%, signal=30%
hsa00380	Tryptophan metabolism	24	-0.564486528	-2.165203511	0.000249087	0.004759107	0.003175	1373	tags=50%, list=11%, signal=45%
hsa05162	Measles	87	0.425167994	1.766299565	0.000257659	0.004759107	0.003175	3059	tags=43%, list=24%, signal=32%
hsa05130	Pathogenic Escherichia coli infection	133	0.379147981	1.662392527	0.000365226	0.006174292	0.004119	3439	tags=43%, list=27%, signal=31%
hsa05310	Asthma	14	-0.670834027	-2.159167387	0.000373604	0.006174292	0.004119	1227	tags=71%, list=10%, signal=65%
hsa05165	Human papillomavirus infection	216	0.339341463	1.562729815	0.000464415	0.007291315	0.004864	2671	tags=34%, list=21%, signal=27%
hsa04144	Endocytosis	175	0.35796221	1.616918461	0.000487845	0.007294449	0.004866	4225	tags=50%, list=34%, signal=34%
hsa00650	Butanoate metabolism	12	-0.688323323	-2.099565116	0.000764686	0.010914149	0.007281	3305	tags=75%, list=26%, signal=55%
hsa04670	Leukocyte transendothelial migration	70	0.430524144	1.746924624	0.000830956	0.011344359	0.007568	2768	tags=46%, list=22%, signal=36%

	hsa05323	Rheumatoid arthritis	46	-0.403128542	-1.771823448	0.001077609	0.014098719	0.009405	2166	tags=41%, list=17%, signal=34%
	hsa00410	beta-Alanine metabolism	19	-0.558321583	-1.963942394	0.001287515	0.016171193	0.010788	3873	tags=63%, list=31%, signal=44%
	hsa05150	Staphylococcus aureus infection	33	-0.439352195	-1.813309328	0.001425464	0.017215224	0.011485	1923	tags=48%, list=15%, signal=41%
	hsa04510	Focal adhesion	136	0.36069872	1.584843474	0.001949956	0.022677262	0.015128	2768	tags=39%, list=22%, signal=31%
	hsa00061	Fatty acid biosynthesis	12	-0.647916647	-1.976314245	0.002231865	0.024565302	0.016388	1922	tags=58%, list=15%, signal=49%
	hsa01522	Endocrine resistance	61	0.434601346	1.71172684	0.00226877	0.024565302	0.016388	3379	tags=51%, list=27%, signal=37%
	hsa00510	N-Glycan biosynthesis	40	0.474270385	1.743195602	0.00246425	0.025792483	0.017207	4377	tags=57%, list=35%, signal=38%
	hsa04151	PI3K-Akt signaling pathway	200	0.326157683	1.490428702	0.002625338	0.026073277	0.017394	3299	tags=40%, list=26%, signal=30%
	hsa05167	Kaposi sarcoma-associated herpesvirus infection	128	0.360204246	1.576692426	0.002669025	0.026073277	0.017394	2768	tags=34%, list=22%, signal=27%
	hsa00330	Arginine and proline metabolism	30	-0.449190624	-1.829957923	0.002769179	0.026073277	0.017394	1221	tags=47%, list=10%, signal=42%
	hsa05222	Small cell lung cancer	70	0.408576484	1.657868276	0.002823221	0.026073277	0.017394	3780	tags=53%, list=30%, signal=37%
	hsa05034	Alcoholism	59	0.430225976	1.691728347	0.003036803	0.026563681	0.017721	2555	tags=37%, list=20%, signal=30%
	hsa04071	Sphingolipid signaling pathway	78	0.411677653	1.683275808	0.003045518	0.026563681	0.017721	3199	tags=42%, list=25%, signal=32%
	hsa05220	Chronic myeloid leukemia	56	0.428434602	1.677669635	0.00316989	0.026901229	0.017946	2472	tags=39%, list=20%, signal=32%
	hsa05169	Epstein-Barr virus infection	144	0.349188376	1.54645935	0.003420836	0.028266912	0.018857	3439	tags=39%, list=27%, signal=29%
	hsa04926	Relaxin signaling pathway	75	0.402262946	1.630347836	0.003667517	0.029528216	0.019699	3439	tags=43%, list=27%, signal=31%
	hsa05166	Human T-cell leukemia virus 1 infection	153	0.339337178	1.510956091	0.003852781	0.029831108	0.019901	2768	tags=34%, list=22%, signal=27%
	hsa05206	MicroRNAs in cancer	116	0.361094697	1.565531838	0.003895145	0.029831108	0.019901	2491	tags=34%, list=20%, signal=28%
	hsa00532	Glycosaminoglycan biosynthesis - chondroitin sulfate / dermatan sulfate	17	0.607578644	1.832673248	0.004748774	0.034186275	0.022806	3120	tags=65%, list=25%, signal=49%
	hsa04530	Tight junction	108	0.373721512	1.602498664	0.004795518	0.034186275	0.022806	2939	tags=41%, list=23%, signal=32%
	hsa05132	Salmonella infection	187	0.32653359	1.485703769	0.004884391	0.034186275	0.022806	4096	tags=47%, list=32%, signal=32%
	hsa05163	Human cytomegalovirus infection	146	0.341419556	1.511016	0.004899307	0.034186275	0.022806	2768	tags=34%, list=22%, signal=26%
	hsa04914	Progesterone-mediated oocyte maturation	53	0.423277376	1.634833371	0.005578052	0.036518449	0.024362	3364	tags=43%, list=27%, signal=32%
	hsa05211	Renal cell carcinoma	49	0.445915031	1.701852506	0.005606296	0.036518449	0.024362	2768	tags=43%, list=22%, signal=34%
	hsa01200	Carbon metabolism	84	-0.287782774	-1.473260348	0.005630021	0.036518449	0.024362	2093	tags=32%, list=17%, signal=27%
	hsa03030	DNA replication	33	0.482207813	1.691613478	0.005698739	0.036518449	0.024362	4351	tags=61%, list=35%, signal=40%
	hsa05170	Human immunodeficiency virus 1 infection	128	0.345757773	1.513457067	0.006611926	0.041475896	0.027669	2768	tags=34%, list=22%, signal=26%
	hsa03050	Proteasome	41	0.456501882	1.682562894	0.006736531	0.041475896	0.027669	2825	tags=41%, list=22%, signal=32%
	hsa00534	Glycosaminoglycan biosynthesis - heparan sulfate / heparin	14	0.605642413	1.718059436	0.007091799	0.042823553	0.028568	2393	tags=64%, list=19%, signal=52%
	hsa04210	Apoptosis	96	0.363828088	1.530768496	0.007633361	0.045224062	0.03017	2910	tags=36%, list=23%, signal=28%
	hsa04330	Notch signaling pathway	44	0.439096144	1.639990097	0.008212653	0.047066884	0.031399	2491	tags=36%, list=20%, signal=29%
	hsa04115	p53 signaling pathway	47	0.425295947	1.605049078	0.0082442	0.047066884	0.031399	3178	tags=43%, list=25%, signal=32%
	hsa05012	Parkinson disease	181	0.313416297	1.420755272	0.008891675	0.049856892	0.03326	3352	tags=35%, list=27%, signal=26%
	hsa00140	Steroid hormone biosynthesis	17	-0.529761387	-1.800299473	0.009076066	0.049997975	0.033354	1982	tags=41%, list=16%, signal=35%

ISL1

GO

ONTOLOGY	ID	Description	setSize	enrichmentScore	NES	pvalue	p.adjust	qvalues	rank	leading_edge
BP	GO:0000375	RNA splicing, via transesterification reactions	250	0.440317144	1.815582153	1.00E-10	1.05E-07	8.12E-08	4753	tags=59%, list=38%, signal=37%
BP	GO:0000377	RNA splicing, via transesterification reactions with bulged adenosine as nucleophile	247	0.442857842	1.824110627	1.00E-10	1.05E-07	8.12E-08	4840	tags=60%, list=38%, signal=38%

BP	GO:0000398	mRNA splicing, via spliceosome	247	0.442857842	1.824110627	1.00E-10	1.05E-07	8.12E-08	4840	tags=60%, list=38%, signal=38%
BP	GO:0006397	mRNA processing	341	0.41076296	1.715951435	1.00E-10	1.05E-07	8.12E-08	4753	tags=55%, list=38%, signal=35%
BP	GO:0044770	cell cycle phase transition	399	0.401970331	1.693479464	1.00E-10	1.05E-07	8.12E-08	3130	tags=40%, list=25%, signal=31%
BP	GO:0044772	mitotic cell cycle phase transition	379	0.409898833	1.72469713	1.00E-10	1.05E-07	8.12E-08	3130	tags=41%, list=25%, signal=32%
BP	GO:0000904	cell morphogenesis involved in differentiation	425	0.388175328	1.639230805	1.50E-10	1.35E-07	1.04E-07	4121	tags=48%, list=33%, signal=33%
BP	GO:0006325	chromatin organization	440	0.383285421	1.620797354	4.39E-10	3.46E-07	2.67E-07	4841	tags=53%, list=38%, signal=34%
MF	GO:0008134	transcription factor binding	425	0.383098745	1.617792832	5.66E-10	3.96E-07	3.06E-07	4464	tags=50%, list=35%, signal=33%
BP	GO:0008380	RNA splicing	311	0.406386062	1.692725059	7.80E-10	4.91E-07	3.80E-07	4753	tags=55%, list=38%, signal=35%
BP	GO:0010564	regulation of cell cycle process	470	0.373394476	1.581048359	9.47E-10	5.42E-07	4.19E-07	3657	tags=41%, list=29%, signal=30%
BP	GO:0051301	cell division	330	0.397700642	1.658205602	3.09E-09	1.62E-06	1.25E-06	3940	tags=48%, list=31%, signal=34%
BP	GO:0007264	small GTPase mediated signal transduction	327	0.397069496	1.655123245	3.96E-09	1.92E-06	1.48E-06	3892	tags=47%, list=31%, signal=33%
BP	GO:0006614	SRP-dependent cotranslational protein targeting to membrane	90	-0.380315807	-2.417902265	8.89E-09	4.00E-06	3.09E-06	1493	tags=31%, list=12%, signal=28%
BP	GO:0001568	blood vessel development	412	0.374547251	1.580857212	1.04E-08	4.36487E-06	3.38E-06	3770	tags=42%, list=30%, signal=31%
BP	GO:0001944	vasculature development	436	0.368484823	1.55718492	1.73E-08	6.7942E-06	5.25E-06	3770	tags=42%, list=30%, signal=30%
MF	GO:0019900	kinase binding	469	0.363742221	1.540108427	2.09E-08	7.74074E-06	5.99E-06	4085	tags=46%, list=32%, signal=32%
BP	GO:0048514	blood vessel morphogenesis	358	0.380749801	1.596009108	2.95E-08	1.03233E-05	7.98E-06	3770	tags=43%, list=30%, signal=31%
CC	GO:0005684	U2-type spliceosomal complex	70	0.553928284	2.081471174	3.38E-08	1.09682E-05	8.48E-06	4324	tags=69%, list=34%, signal=45%
BP	GO:0000086	G2/M transition of mitotic cell cycle	171	0.44331155	1.783099648	3.48E-08	1.09682E-05	8.48E-06	3127	tags=44%, list=25%, signal=33%
BP	GO:1903311	regulation of mRNA metabolic process	229	0.410536342	1.684695089	5.52E-08	1.54987E-05	1.2E-05	3971	tags=48%, list=31%, signal=33%
MF	GO:0003682	chromatin binding	360	0.379688703	1.592081301	5.83E-08	1.54987E-05	1.2E-05	4515	tags=49%, list=36%, signal=33%
BP	GO:0007346	regulation of mitotic cell cycle	407	0.371966597	1.569692956	6.03E-08	1.54987E-05	1.2E-05	3657	tags=41%, list=29%, signal=30%
MF	GO:0003712	transcription coregulator activity	328	0.383947052	1.600781111	6.04E-08	1.54987E-05	1.2E-05	4694	tags=52%, list=37%, signal=33%
BP	GO:0044839	cell cycle G2/M phase transition	179	0.433904187	1.750604988	6.15E-08	1.54987E-05	1.2E-05	3127	tags=43%, list=25%, signal=33%
BP	GO:0048667	cell morphogenesis involved in neuron differentiation	331	0.385047904	1.605876393	6.46E-08	1.56355E-05	1.21E-05	4121	tags=48%, list=33%, signal=33%
CC	GO:0005681	spliceosomal complex	140	0.459028061	1.82926115	6.90E-08	1.60839E-05	1.24E-05	4753	tags=63%, list=38%, signal=40%
BP	GO:0001525	angiogenesis	309	0.389697877	1.622858862	7.29E-08	1.63909E-05	1.27E-05	3761	tags=44%, list=30%, signal=32%
CC	GO:0022626	cytosolic ribosome	98	-0.353989904	-2.280951625	8.13E-08	1.76593E-05	1.37E-05	1493	tags=30%, list=12%, signal=26%
BP	GO:0045047	protein targeting to ER	102	-0.344671518	-2.185994042	8.88E-08	1.82676E-05	1.41E-05	2263	tags=37%, list=18%, signal=31%
CC	GO:0005819	spindle	206	0.422927165	1.722350161	8.99E-08	1.82676E-05	1.41E-05	3573	tags=47%, list=28%, signal=34%
BP	GO:0001667	ameboidal-type cell migration	267	0.40031096	1.656067377	1.07E-07	2.09664E-05	1.62E-05	3761	tags=45%, list=30%, signal=32%
BP	GO:0032990	cell part morphogenesis	392	0.36938003	1.555709923	1.28E-07	2.43325E-05	1.88E-05	3254	tags=39%, list=26%, signal=30%
BP	GO:1901990	regulation of mitotic cell cycle phase transition	284	0.392250917	1.626522604	1.36E-07	2.51783E-05	1.95E-05	3657	tags=43%, list=29%, signal=31%
BP	GO:0007265	Ras protein signal transduction	223	0.416997872	1.707941845	1.55E-07	2.79275E-05	2.16E-05	3076	tags=44%, list=24%, signal=34%
BP	GO:0072329	monocarboxylic acid catabolic process	80	-0.378287934	-2.239197871	1.77E-07	3.09463E-05	2.39E-05	1682	tags=35%, list=13%, signal=31%
BP	GO:0006613	cotranslational protein targeting to membrane	94	-0.361459164	-2.373470971	2.20E-07	3.75007E-05	2.9E-05	1493	tags=30%, list=12%, signal=26%
BP	GO:0048858	cell projection morphogenesis	377	0.369740074	1.555588491	2.44E-07	4.03645E-05	3.12E-05	3254	tags=39%, list=26%, signal=30%
BP	GO:0043903	regulation of symbiotic process	150	0.436261892	1.747764255	2.83E-07	4.5622E-05	3.53E-05	4322	tags=57%, list=34%, signal=38%
BP	GO:0048812	neuron projection morphogenesis	362	0.371415749	1.557063192	3.07E-07	4.83978E-05	3.74E-05	3254	tags=40%, list=26%, signal=30%
BP	GO:0007059	chromosome segregation	173	0.423887197	1.706683789	3.76E-07	5.77531E-05	4.47E-05	2885	tags=41%, list=23%, signal=32%

BP	GO:0006260	DNA replication	166	0.430602863	1.730472577	3.93E-07	5.89853E-05	4.56E-05	2729	tags=39%, list=22%, signal=31%
BP	GO:0006281	DNA repair	331	0.374580172	1.56221979	4.30E-07	6.30294E-05	4.87E-05	2905	tags=37%, list=23%, signal=29%
BP	GO:0032989	cellular component morphogenesis	435	0.351501802	1.485443104	5.07E-07	6.8589E-05	5.3E-05	4346	tags=46%, list=34%, signal=31%
BP	GO:0007411	axon guidance	149	0.442718294	1.773983881	5.08E-07	6.8589E-05	5.3E-05	3227	tags=46%, list=26%, signal=34%
BP	GO:0097485	neuron projection guidance	149	0.442718294	1.773983881	5.08E-07	6.8589E-05	5.3E-05	3227	tags=46%, list=26%, signal=34%
BP	GO:0072599	establishment of protein localization to endoplasmic reticulum	105	-0.331189353	-2.105526429	5.12E-07	6.8589E-05	5.3E-05	1923	tags=37%, list=15%, signal=32%
BP	GO:0120039	plasma membrane bounded cell projection morphogenesis	374	0.364626944	1.532509803	6.26E-07	8.20973E-05	6.35E-05	3254	tags=39%, list=26%, signal=30%
BP	GO:0070646	protein modification by small protein removal	205	0.404808056	1.648586167	6.70E-07	8.55033E-05	6.61E-05	4230	tags=50%, list=34%, signal=34%
MF	GO:0140297	DNA-binding transcription factor binding	233	0.393964746	1.617443236	6.79E-07	8.55033E-05	6.61E-05	5230	tags=55%, list=41%, signal=33%
BP	GO:1901987	regulation of cell cycle phase transition	299	0.376977024	1.567233581	7.19E-07	8.88318E-05	6.87E-05	3657	tags=41%, list=29%, signal=30%
BP	GO:0009062	fatty acid catabolic process	70	-0.399988163	-2.322966787	7.50E-07	9.08278E-05	7.02E-05	1659	tags=34%, list=13%, signal=30%
BP	GO:0007409	axonogenesis	262	0.391688734	1.617603115	7.80E-07	9.26533E-05	7.16E-05	3660	tags=44%, list=29%, signal=32%
BP	GO:0010608	posttranscriptional regulation of gene expression	436	0.350980145	1.483211669	8.30E-07	9.53294E-05	7.37E-05	4226	tags=45%, list=34%, signal=31%
CC	GO:0098687	chromosomal region	192	0.413616678	1.678749392	8.33E-07	9.53294E-05	7.37E-05	5433	tags=62%, list=43%, signal=36%
BP	GO:0050684	regulation of mRNA processing	92	0.488272312	1.885732227	9.27E-07	0.000104198	8.06E-05	3847	tags=54%, list=31%, signal=38%
BP	GO:0061564	axon development	287	0.383442225	1.590413774	9.51E-07	0.000105028	8.12E-05	3660	tags=44%, list=29%, signal=32%
CC	GO:0005667	transcription regulator complex	256	0.38989024	1.607546695	1.00E-06	0.000108797	8.41E-05	5057	tags=57%, list=40%, signal=35%
BP	GO:0051169	nuclear transport	247	0.386116274	1.590394775	1.34E-06	0.000143517	0.000111	5420	tags=58%, list=43%, signal=34%
BP	GO:0035239	tube morphogenesis	491	0.345753967	1.467661315	1.38E-06	0.00014455	0.000112	4530	tags=47%, list=36%, signal=31%
BP	GO:0050792	regulation of viral process	142	0.430015364	1.715075271	1.40E-06	0.00014455	0.000112	4126	tags=54%, list=33%, signal=37%
BP	GO:0019058	viral life cycle	224	0.396962879	1.626535435	1.77E-06	0.000180264	0.000139	3913	tags=50%, list=31%, signal=35%
MF	GO:0016817	hydrolase activity, acting on acid anhydrides	498	0.336889277	1.430170282	2.12E-06	0.000208122	0.000161	4741	tags=49%, list=38%, signal=32%
MF	GO:0016818	hydrolase activity, acting on acid anhydrides, in phosphorus-containing anhydrides	498	0.336889277	1.430170282	2.12E-06	0.000208122	0.000161	4741	tags=49%, list=38%, signal=32%
BP	GO:0033044	regulation of chromosome organization	223	0.402451668	1.648363434	2.15E-06	0.000208669	0.000161	4507	tags=51%, list=36%, signal=33%
MF	GO:0140097	catalytic activity, acting on DNA	137	0.430386899	1.715046805	2.23E-06	0.000212509	0.000164	3108	tags=44%, list=25%, signal=33%
MF	GO:0019904	protein domain specific binding	444	0.346084617	1.463594007	2.29E-06	0.000214966	0.000166	4484	tags=46%, list=36%, signal=31%
BP	GO:0010389	regulation of G2/M transition of mitotic cell cycle	135	0.438301116	1.74617596	2.37E-06	0.000218369	0.000169	3651	tags=47%, list=29%, signal=34%
BP	GO:0045930	negative regulation of mitotic cell cycle	217	0.39361745	1.610065203	2.39E-06	0.000218369	0.000169	3020	tags=38%, list=24%, signal=29%
BP	GO:0018205	peptidyl-lysine modification	252	0.387669537	1.597926338	2.46E-06	0.000221666	0.000171	4672	tags=52%, list=37%, signal=33%
BP	GO:1903900	regulation of viral life cycle	99	0.472145128	1.833951005	2.60E-06	0.000230248	0.000178	3913	tags=58%, list=31%, signal=40%
CC	GO:0005815	microtubule organizing center	422	0.350540788	1.480714109	2.80E-06	0.000240489	0.000186	5302	tags=55%, list=42%, signal=33%
BP	GO:0006261	DNA-dependent DNA replication	88	0.482493465	1.855869509	2.88E-06	0.000240489	0.000186	2864	tags=48%, list=23%, signal=37%
BP	GO:0010631	epithelial cell migration	196	0.404769531	1.647088049	2.88E-06	0.000240489	0.000186	3761	tags=45%, list=30%, signal=32%
BP	GO:0090132	epithelium migration	196	0.404769531	1.647088049	2.88E-06	0.000240489	0.000186	3761	tags=45%, list=30%, signal=32%
BP	GO:0006913	nucleocytoplasmic transport	245	0.385680123	1.587330768	2.91E-06	0.000240489	0.000186	5420	tags=58%, list=43%, signal=34%
MF	GO:0017111	nucleoside-triphosphatase activity	464	0.342237308	1.448691554	2.94E-06	0.000240489	0.000186	4741	tags=49%, list=38%, signal=32%
BP	GO:1902749	regulation of cell cycle G2/M phase transition	142	0.422792463	1.68626742	3.06E-06	0.000246722	0.000191	3127	tags=42%, list=25%, signal=32%
MF	GO:0003725	double-stranded RNA binding	54	0.546545627	1.987469658	3.20E-06	0.000255314	0.000197	3230	tags=59%, list=26%, signal=44%
BP	GO:0044843	cell cycle G1/S phase transition	180	0.410255317	1.655106908	3.29E-06	0.000259321	0.000201	4416	tags=51%, list=35%, signal=34%

BP	GO:0051640	organelle localization	392	0.355358341	1.49665508	3.39E-06	0.000263373	0.000204	4148	tags=45%, list=33%, signal=31%
MF	GO:0060090	molecular adaptor activity	185	0.404060844	1.63496206	3.47E-06	0.000266241	0.000206	4829	tags=57%, list=38%, signal=36%
MF	GO:0140098	catalytic activity, acting on RNA	235	0.387838609	1.594100262	3.64E-06	0.000276171	0.000214	4234	tags=49%, list=34%, signal=33%
BP	GO:0045786	negative regulation of cell cycle	391	0.352614665	1.484763997	3.84E-06	0.000283218	0.000219	3604	tags=39%, list=29%, signal=29%
BP	GO:0045446	endothelial cell differentiation	67	0.508198943	1.897878655	3.86E-06	0.000283218	0.000219	2951	tags=54%, list=23%, signal=41%
BP	GO:0048024	regulation of mRNA splicing, via spliceosome	66	0.511048871	1.904716134	3.87E-06	0.000283218	0.000219	3690	tags=59%, list=29%, signal=42%
CC	GO:0005813	centrosome	335	0.363222808	1.515392316	3.98E-06	0.000287989	0.000223	5302	tags=57%, list=42%, signal=34%
BP	GO:0003158	endothelium development	80	0.493880763	1.878721143	4.18E-06	0.00029941	0.000232	2951	tags=51%, list=23%, signal=40%
BP	GO:0090130	tissue migration	198	0.401235314	1.632849769	4.40E-06	0.000311515	0.000241	3761	tags=45%, list=30%, signal=32%
MF	GO:0003729	mRNA binding	205	0.390962053	1.592198136	4.68E-06	0.000327131	0.000253	3890	tags=47%, list=31%, signal=33%
BP	GO:0010948	negative regulation of cell cycle process	227	0.38438733	1.577577665	4.81E-06	0.000332789	0.000257	3651	tags=41%, list=29%, signal=29%
BP	GO:0098813	nuclear chromosome segregation	135	0.432357432	1.722496533	4.89E-06	0.000334912	0.000259	2885	tags=41%, list=23%, signal=32%
BP	GO:1905564	positive regulation of vascular endothelial cell proliferation	11	0.821442812	2.137731426	5.45E-06	0.00036881	0.000285	1741	tags=55%, list=14%, signal=47%
MF	GO:0016614	oxidoreductase activity, acting on CH-OH group of donors	80	-0.34193127	-2.023992053	5.63E-06	0.000376994	0.000292	1996	tags=36%, list=16%, signal=31%
BP	GO:0000082	G1/S transition of mitotic cell cycle	168	0.411637618	1.656167627	5.78957E-06	0.000379857	0.000294	4416	tags=52%, list=35%, signal=34%
BP	GO:0000380	alternative mRNA splicing, via spliceosome	44	0.570937433	2.011880254	5.79105E-06	0.000379857	0.000294	3690	tags=66%, list=29%, signal=47%
BP	GO:0016579	protein deubiquitination	191	0.40028701	1.623852369	6.21181E-06	0.000403255	0.000312	4230	tags=50%, list=34%, signal=34%
CC	GO:0000775	chromosome, centromeric region	112	0.446338948	1.753948094	6.3762E-06	0.000409703	0.000317	5165	tags=64%, list=41%, signal=38%
BP	GO:0006403	RNA localization	174	0.412138479	1.659787511	6.62159E-06	0.000421174	0.000326	4272	tags=52%, list=34%, signal=35%
BP	GO:0051056	regulation of small GTPase mediated signal transduction	202	0.397508216	1.617821344	6.76027E-06	0.000425694	0.000329	3989	tags=49%, list=32%, signal=34%
BP	GO:0016570	histone modification	275	0.371203957	1.536695897	8.13614E-06	0.00050726	0.000392	4841	tags=52%, list=38%, signal=33%
BP	GO:0007507	heart development	311	0.357262733	1.488111031	8.54482E-06	0.000527517	0.000408	3866	tags=42%, list=31%, signal=30%
BP	GO:0002478	antigen processing and presentation of exogenous peptide antigen	136	0.422566493	1.683386699	9.55556E-06	0.000584188	0.000452	3684	tags=46%, list=29%, signal=33%
MF	GO:0016462	pyrophosphatase activity	495	0.33631881	1.427617282	9.79526E-06	0.000593084	0.000459	4741	tags=49%, list=38%, signal=32%
BP	GO:1901991	negative regulation of mitotic cell cycle phase transition	164	0.410600502	1.6502071	9.96405E-06	0.000597559	0.000462	3020	tags=38%, list=24%, signal=30%
BP	GO:0051983	regulation of chromosome segregation	61	0.505795373	1.871489736	1.05384E-05	0.000626043	0.000484	2831	tags=46%, list=22%, signal=36%
CC	GO:0071005	U2-type precatalytic spliceosome	40	0.56965551	1.966983586	1.3048E-05	0.000763386	0.00059	4324	tags=68%, list=34%, signal=44%
CC	GO:0071011	precatalytic spliceosome	41	0.572059756	1.996935839	1.30994E-05	0.000763386	0.00059	4324	tags=68%, list=34%, signal=45%
MF	GO:0016836	hydro-lyase activity	30	-0.513014987	-2.33048931	1.3228E-05	0.000763386	0.00059	1562	tags=47%, list=12%, signal=41%
BP	GO:0019884	antigen processing and presentation of exogenous antigen	138	0.424793651	1.691272424	1.33353E-05	0.000763386	0.00059	3684	tags=46%, list=29%, signal=33%
MF	GO:0004386	helicase activity	118	0.436367505	1.720672822	1.40335E-05	0.000796116	0.000616	4741	tags=60%, list=38%, signal=38%
BP	GO:0045601	regulation of endothelial cell differentiation	24	0.649306554	2.060135979	1.48507E-05	0.000834955	0.000646	1489	tags=50%, list=12%, signal=44%
MF	GO:0016616	oxidoreductase activity, acting on the CH-OH group of donors, NAD or NADP as acceptor	74	-0.343612014	-2.059006193	1.56367E-05	0.000871365	0.000674	1978	tags=36%, list=16%, signal=31%
BP	GO:0042572	retinol metabolic process	19	-0.658766668	-2.684026401	1.64921E-05	0.000904887	0.0007	2016	tags=58%, list=16%, signal=49%
BP	GO:0019941	modification-dependent protein catabolic process	453	0.335034306	1.417606858	1.66155E-05	0.000904887	0.0007	3749	tags=40%, list=30%, signal=29%
BP	GO:0034340	response to type I interferon	72	0.487467715	1.833835346	1.66693E-05	0.000904887	0.0007	4308	tags=62%, list=34%, signal=41%
BP	GO:0031589	cell-substrate adhesion	218	0.383947141	1.569591656	1.7768E-05	0.000956284	0.000739	4203	tags=48%, list=33%, signal=33%
BP	GO:1901988	negative regulation of cell cycle phase transition	171	0.403056542	1.621184872	1.86448E-05	0.000994967	0.000769	3020	tags=37%, list=24%, signal=29%
MF	GO:0003688	DNA replication origin binding	13	0.758238651	2.060482783	1.88313E-05	0.000996477	0.000771	1585	tags=77%, list=13%, signal=67%

BP	GO:0016569	covalent chromatin modification	282	0.366817013	1.520462334	1.97069E-05	0.001034122	0.0008	4841	tags=52%, list=38%, signal=33%
MF	GO:0016887	ATPase activity	254	0.367297905	1.514104739	2.16636E-05	0.001127404	0.000872	4741	tags=52%, list=38%, signal=33%
BP	GO:0048002	antigen processing and presentation of peptide antigen	143	0.409401435	1.634280742	2.31075E-05	0.001192687	0.000922	3684	tags=45%, list=29%, signal=33%
MF	GO:0019901	protein kinase binding	414	0.343083074	1.448383267	2.3876E-05	0.001222333	0.000945	4085	tags=44%, list=32%, signal=31%
BP	GO:0043542	endothelial cell migration	145	0.412791054	1.65103082	2.45079E-05	0.001244567	0.000962	3739	tags=46%, list=30%, signal=33%
BP	GO:0051168	nuclear export	146	0.41229075	1.649310685	2.56283E-05	0.001291049	0.000998	5418	tags=62%, list=43%, signal=36%
BP	GO:0045664	regulation of neuron differentiation	363	0.344965898	1.446557667	2.83723E-05	0.001417938	0.001096	3663	tags=40%, list=29%, signal=29%
MF	GO:0030674	protein-macromolecule adaptor activity	161	0.401990264	1.613181178	2.90211E-05	0.001438946	0.001113	4829	tags=57%, list=38%, signal=36%
CC	GO:0000793	condensed chromosome	115	0.434313714	1.709661524	2.96739E-05	0.001459819	0.001129	4682	tags=57%, list=37%, signal=36%
BP	GO:0006511	ubiquitin-dependent protein catabolic process	447	0.334855856	1.417018122	3.00277E-05	0.00146577	0.001133	3749	tags=40%, list=30%, signal=29%
BP	GO:0000280	nuclear division	204	0.387757807	1.57871957	3.06502E-05	0.001484647	0.001148	5302	tags=58%, list=42%, signal=34%
MF	GO:0004029	aldehyde dehydrogenase (NAD+) activity	12	-0.751258445	-2.481534813	3.12786E-05	0.001492133	0.001154	1478	tags=75%, list=12%, signal=66%
MF	GO:0004030	aldehyde dehydrogenase [NAD(P)+] activity	12	-0.751258445	-2.481534813	3.12786E-05	0.001492133	0.001154	1478	tags=75%, list=12%, signal=66%
CC	GO:0097225	sperm midpiece	11	-0.782778607	-2.563101258	3.24363E-05	0.001535723	0.001188	1282	tags=64%, list=10%, signal=57%
BP	GO:0001935	endothelial cell proliferation	85	0.458659958	1.760406618	3.26973E-05	0.00153653	0.001188	4143	tags=54%, list=33%, signal=37%
BP	GO:0006405	RNA export from nucleus	101	0.447767049	1.74024735	3.32997E-05	0.001553244	0.001201	4094	tags=55%, list=32%, signal=38%
BP	GO:0043632	modification-dependent macromolecule catabolic process	461	0.332341501	1.407210598	3.39564E-05	0.001565448	0.001211	3749	tags=40%, list=30%, signal=29%
MF	GO:0061629	RNA polymerase II-specific DNA-binding transcription factor binding	184	0.391928033	1.583894743	3.40585E-05	0.001565448	0.001211	4350	tags=48%, list=35%, signal=32%
CC	GO:0000776	kinetochore	78	0.465607212	1.765311775	3.51855E-05	0.001605531	0.001242	4272	tags=58%, list=34%, signal=38%
BP	GO:0034502	protein localization to chromosome	48	0.530801801	1.900435556	3.7514E-05	0.001699465	0.001314	2705	tags=46%, list=21%, signal=36%
CC	GO:0005874	microtubule	219	0.370361218	1.514437312	3.85234E-05	0.001732728	0.00134	3141	tags=40%, list=25%, signal=30%
BP	GO:0042330	taxis	324	0.354867361	1.478344038	3.96085E-05	0.001768899	0.001368	3899	tags=41%, list=31%, signal=29%
CC	GO:0016607	nuclear speck	259	0.366503359	1.513211567	4.07321E-05	0.001806269	0.001397	5434	tags=61%, list=43%, signal=35%
BP	GO:0016458	gene silencing	149	0.404655263	1.621464309	4.13767E-05	0.001822022	0.001409	4407	tags=52%, list=35%, signal=34%
MF	GO:0016903	oxidoreductase activity, acting on the aldehyde or oxo group of donors	31	-0.490534062	-2.262460411	4.31516E-05	0.001885458	0.001458	1733	tags=48%, list=14%, signal=42%
CC	GO:0071013	catalytic step 2 spliceosome	67	0.484446401	1.809174334	4.34161E-05	0.001885458	0.001458	4040	tags=58%, list=32%, signal=40%
BP	GO:0001936	regulation of endothelial cell proliferation	76	0.468340342	1.771685683	4.50031E-05	0.001940989	0.001501	3114	tags=46%, list=25%, signal=35%
BP	GO:0006611	protein export from nucleus	136	0.409173767	1.630033826	4.53627E-05	0.001943191	0.001503	5024	tags=57%, list=40%, signal=35%
BP	GO:0001938	positive regulation of endothelial cell proliferation	56	0.503783697	1.844187087	4.61414E-05	0.00196319	0.001518	3114	tags=48%, list=25%, signal=36%
BP	GO:0060337	type I interferon signaling pathway	69	0.485834186	1.82666078	4.71039E-05	0.001977423	0.001529	4308	tags=62%, list=34%, signal=41%
BP	GO:0071357	cellular response to type I interferon	69	0.485834186	1.82666078	4.71039E-05	0.001977423	0.001529	4308	tags=62%, list=34%, signal=41%
BP	GO:0006935	chemotaxis	322	0.353611806	1.473097616	5.14066E-05	0.002143758	0.001658	3899	tags=41%, list=31%, signal=29%
BP	GO:0030036	actin cytoskeleton organization	422	0.337106371	1.423965989	5.41615E-05	0.002243784	0.001735	3850	tags=41%, list=31%, signal=29%
BP	GO:0051960	regulation of nervous system development	491	0.323936133	1.375048666	5.80958E-05	0.002391042	0.001849	3663	tags=38%, list=29%, signal=28%
BP	GO:0033045	regulation of sister chromatid segregation	51	0.513041501	1.850330784	5.94627E-05	0.002431408	0.00188	2831	tags=47%, list=22%, signal=37%
BP	GO:0061061	muscle structure development	347	0.343270215	1.435957711	6.26706E-05	0.002546044	0.001969	4052	tags=41%, list=32%, signal=29%
BP	GO:0050657	nucleic acid transport	142	0.395834625	1.578748653	6.39918E-05	0.002566599	0.001985	4272	tags=50%, list=34%, signal=33%
BP	GO:0050658	RNA transport	142	0.395834625	1.578748653	6.39918E-05	0.002566599	0.001985	4272	tags=50%, list=34%, signal=33%
BP	GO:0030029	actin filament-based process	462	0.326887924	1.38408874	6.59921E-05	0.00259847	0.002009	3850	tags=40%, list=31%, signal=29%

BP	GO:0050767	regulation of neurogenesis	449	0.327224919	1.384610491	6.59921E-05	0.00259847	0.002009	3663	tags=38%, list=29%, signal=28%
BP	GO:0051310	metaphase plate congression	36	0.549693423	1.86708708	6.60243E-05	0.00259847	0.002009	2775	tags=56%, list=22%, signal=43%
BP	GO:0090022	regulation of neutrophil chemotaxis	18	0.666227324	1.980578161	6.64624E-05	0.002599465	0.00201	1256	tags=39%, list=10%, signal=35%
BP	GO:0048863	stem cell differentiation	163	0.395093091	1.586462289	6.8522E-05	0.002663477	0.00206	3660	tags=44%, list=29%, signal=31%
CC	GO:0022625	cytosolic large ribosomal subunit	51	-0.399428355	-2.178125751	6.92578E-05	0.002675559	0.002069	1493	tags=33%, list=12%, signal=30%
BP	GO:0071624	positive regulation of granulocyte chemotaxis	14	0.716222789	1.978435849	7.08382E-05	0.002703444	0.002091	1256	tags=43%, list=10%, signal=39%
BP	GO:0090023	positive regulation of neutrophil chemotaxis	14	0.716222789	1.978435849	7.08382E-05	0.002703444	0.002091	1256	tags=43%, list=10%, signal=39%
MF	GO:0005096	GTPase activator activity	164	0.394421963	1.585185408	7.18477E-05	0.002709131	0.002095	3989	tags=46%, list=32%, signal=32%
BP	GO:0043123	positive regulation of I-kappaB kinase/NF-kappaB signaling	132	0.410759316	1.634159666	7.18477E-05	0.002709131	0.002095	4300	tags=52%, list=34%, signal=34%
BP	GO:0071166	ribonucleoprotein complex localization	96	0.439582365	1.700094213	7.25749E-05	0.002720262	0.002104	4094	tags=54%, list=32%, signal=37%
BP	GO:0045596	negative regulation of cell differentiation	389	0.335187774	1.411693496	7.38884E-05	0.002744601	0.002122	5049	tags=50%, list=40%, signal=31%
BP	GO:0000381	regulation of alternative mRNA splicing, via spliceosome	38	0.547621864	1.881500042	7.40959E-05	0.002744601	0.002122	3690	tags=63%, list=29%, signal=45%
BP	GO:0000819	sister chromatid segregation	114	0.424990723	1.672681321	7.65268E-05	0.002814756	0.002177	2885	tags=42%, list=23%, signal=33%
BP	GO:0001570	vasculogenesis	53	0.51358013	1.859881927	7.68839E-05	0.002814756	0.002177	3120	tags=51%, list=25%, signal=38%
BP	GO:0000075	cell cycle checkpoint	134	0.407721506	1.623277622	7.75826E-05	0.002823917	0.002184	4466	tags=50%, list=35%, signal=33%
BP	GO:0016441	posttranscriptional gene silencing	100	0.433181489	1.684432263	8.1718E-05	0.002926129	0.002263	4096	tags=52%, list=32%, signal=35%
BP	GO:0035194	post-transcriptional gene silencing by RNA	100	0.433181489	1.684432263	8.1718E-05	0.002926129	0.002263	4096	tags=52%, list=32%, signal=35%
BP	GO:0010638	positive regulation of organelle organization	405	0.331548965	1.398404228	8.17848E-05	0.002926129	0.002263	5383	tags=54%, list=43%, signal=32%
BP	GO:0031047	gene silencing by RNA	112	0.419648344	1.649063826	8.35808E-05	0.002973493	0.002299	4407	tags=54%, list=35%, signal=35%
CC	GO:0000932	P-body	60	0.489071006	1.802893263	8.4748E-05	0.002998079	0.002318	4259	tags=62%, list=34%, signal=41%
MF	GO:0042393	histone binding	145	0.401759166	1.606906836	8.65564E-05	0.003044947	0.002355	5314	tags=61%, list=42%, signal=36%
BP	GO:0140014	mitotic nuclear division	162	0.389397864	1.563350832	0.000101367	0.003528961	0.002729	5302	tags=59%, list=42%, signal=35%
MF	GO:0008146	sulfotransferase activity	24	0.615674579	1.953427612	0.000101436	0.003528961	0.002729	1434	tags=46%, list=11%, signal=41%
CC	GO:0036464	cytoplasmic ribonucleoprotein granule	155	0.39273984	1.572599991	0.000103632	0.003585547	0.002773	4402	tags=51%, list=35%, signal=34%
CC	GO:0000777	condensed chromosome kinetochore	56	0.492515899	1.80293937	0.000107903	0.003701614	0.002862	4272	tags=62%, list=34%, signal=42%
MF	GO:0003713	transcription coactivator activity	186	0.380639082	1.540454853	0.000108162	0.003701614	0.002862	4740	tags=51%, list=38%, signal=32%
BP	GO:0052372	modulation by symbiont of entry into host	27	0.598687792	1.935768694	0.000109418	0.003724343	0.00288	3818	tags=70%, list=30%, signal=49%
BP	GO:0016126	sterol biosynthetic process	55	-0.362994334	-1.960568387	0.000113148	0.003813762	0.002949	2612	tags=49%, list=21%, signal=39%
MF	GO:0008270	zinc ion binding	438	0.327299228	1.383825579	0.00011337	0.003813762	0.002949	4649	tags=46%, list=37%, signal=30%
BP	GO:0009615	response to virus	212	0.369618195	1.508386077	0.000114842	0.003813762	0.002949	4322	tags=50%, list=34%, signal=33%
BP	GO:0090068	positive regulation of cell cycle process	166	0.388738351	1.562230803	0.000114958	0.003813762	0.002949	3657	tags=43%, list=29%, signal=31%
BP	GO:0060759	regulation of response to cytokine stimulus	117	0.413457099	1.630797839	0.000115073	0.003813762	0.002949	4649	tags=56%, list=37%, signal=36%
BP	GO:0007017	microtubule-based process	425	0.327608645	1.383462945	0.000116765	0.003813853	0.002949	5540	tags=55%, list=44%, signal=32%
MF	GO:0030695	GTPase regulator activity	187	0.375910139	1.521650121	0.000117234	0.003813853	0.002949	4124	tags=45%, list=33%, signal=31%
BP	GO:0050000	chromosome localization	43	0.526023424	1.849739299	0.000117498	0.003813853	0.002949	2775	tags=51%, list=22%, signal=40%
BP	GO:0051303	establishment of chromosome localization	43	0.526023424	1.849739299	0.000117498	0.003813853	0.002949	2775	tags=51%, list=22%, signal=40%
BP	GO:0048729	tissue morphogenesis	374	0.334854489	1.407377581	0.000119033	0.003835724	0.002966	4161	tags=44%, list=33%, signal=30%
BP	GO:0043547	positive regulation of GTPase activity	236	0.360770364	1.48294637	0.00011939	0.003835724	0.002966	3989	tags=44%, list=32%, signal=31%
BP	GO:0043087	regulation of GTPase activity	287	0.35370877	1.467087512	0.000121421	0.003881164	0.003001	4124	tags=44%, list=33%, signal=30%

MF	GO:0019787	ubiquitin-like protein transferase activity	294	0.345981725	1.435965984	0.000123691	0.003933738	0.003042	5248	tags=55%, list=42%, signal=33%
BP	GO:0051236	establishment of RNA localization	144	0.396144366	1.582001799	0.000126339	0.003997778	0.003091	4094	tags=49%, list=32%, signal=33%
BP	GO:0060968	regulation of gene silencing	71	0.465051431	1.752219864	0.000128139	0.004029313	0.003116	3910	tags=56%, list=31%, signal=39%
MF	GO:0003714	transcription corepressor activity	122	0.416521962	1.647317996	0.000128616	0.004029313	0.003116	5361	tags=62%, list=43%, signal=36%
BP	GO:0006302	double-strand break repair	131	0.39874484	1.586899332	0.000133168	0.004151286	0.00321	5360	tags=59%, list=43%, signal=34%
BP	GO:0071426	ribonucleoprotein complex export from nucleus	95	0.434876229	1.680895042	0.000134381	0.004168466	0.003223	4094	tags=54%, list=32%, signal=37%
BP	GO:0043484	regulation of RNA splicing	95	0.434338782	1.678817685	0.000136678	0.004218937	0.003263	4753	tags=59%, list=38%, signal=37%
CC	GO:0035770	ribonucleoprotein granule	161	0.389530844	1.563181705	0.000137721	0.00423038	0.003271	4402	tags=50%, list=35%, signal=33%
BP	GO:0051129	negative regulation of cellular component organization	450	0.325874575	1.379296452	0.000141705	0.004325631	0.003345	4933	tags=48%, list=39%, signal=30%
MF	GO:0046982	protein heterodimerization activity	137	0.394788092	1.573189278	0.000142273	0.004325631	0.003345	5184	tags=60%, list=41%, signal=36%
CC	GO:0000779	condensed chromosome, centromeric region	64	0.478741466	1.783619947	0.00014345	0.004325631	0.003345	4272	tags=61%, list=34%, signal=40%
BP	GO:0099003	vesicle-mediated transport in synapse	104	0.422778775	1.648224987	0.000143569	0.004325631	0.003345	4057	tags=47%, list=32%, signal=32%
BP	GO:0051147	regulation of muscle cell differentiation	85	0.439102492	1.685342091	0.000145867	0.004373912	0.003382	3953	tags=49%, list=31%, signal=34%
BP	GO:0043487	regulation of RNA stability	142	0.38888729	1.551039871	0.000151379	0.004517688	0.003494	4278	tags=48%, list=34%, signal=32%
BP	GO:1901532	regulation of hematopoietic progenitor cell differentiation	73	0.458783307	1.726128226	0.00015432	0.004583731	0.003545	3610	tags=48%, list=29%, signal=34%
BP	GO:0006270	DNA replication initiation	24	0.60849074	1.930634548	0.000157714	0.004662567	0.003606	2472	tags=58%, list=20%, signal=47%
BP	GO:0033209	tumor necrosis factor-mediated signaling pathway	116	0.412226967	1.624227048	0.000158526	0.004664658	0.003607	4128	tags=49%, list=33%, signal=33%
BP	GO:0071711	basement membrane organization	20	0.642426935	1.964590039	0.000160433	0.004698836	0.003634	2103	tags=65%, list=17%, signal=54%
BP	GO:0060147	regulation of posttranscriptional gene silencing	64	0.477370025	1.778510448	0.00016211	0.004704181	0.003638	3910	tags=58%, list=31%, signal=40%
BP	GO:0060966	regulation of gene silencing by RNA	64	0.477370025	1.778510448	0.00016211	0.004704181	0.003638	3910	tags=58%, list=31%, signal=40%
BP	GO:0002040	sprouting angiogenesis	81	0.461861226	1.761257726	0.000164743	0.004745372	0.00367	4143	tags=59%, list=33%, signal=40%
BP	GO:0071897	DNA biosynthetic process	130	0.406435771	1.616858277	0.000165037	0.004745372	0.00367	3950	tags=46%, list=31%, signal=32%
BP	GO:0140056	organelle localization by membrane tethering	103	0.418326869	1.629877892	0.000171134	0.004898329	0.003788	5265	tags=60%, list=42%, signal=35%
CC	GO:1904949	ATPase complex	66	0.461213198	1.718974974	0.000173416	0.00494118	0.003821	4825	tags=64%, list=38%, signal=39%
BP	GO:0016101	diterpenoid metabolic process	50	-0.373826348	-2.004626801	0.00017809	0.005051494	0.003906	2421	tags=50%, list=19%, signal=41%
BP	GO:0018394	peptidyl-lysine acetylation	113	0.409874221	1.611260078	0.000186084	0.00525457	0.004063	4672	tags=53%, list=37%, signal=34%
BP	GO:0035024	negative regulation of Rho protein signal transduction	15	0.687609497	1.951742559	0.000192592	0.00541408	0.004187	1751	tags=47%, list=14%, signal=40%
BP	GO:0043393	regulation of protein binding	136	0.39470329	1.572387492	0.000196905	0.005503002	0.004255	4577	tags=55%, list=36%, signal=36%
BP	GO:0031145	anaphase-promoting complex-dependent catabolic process	62	0.480653882	1.782811763	0.000197503	0.005503002	0.004255	3008	tags=47%, list=24%, signal=36%
BP	GO:0034330	cell junction organization	354	0.337196159	1.411730129	0.000198584	0.005508744	0.00426	3850	tags=41%, list=31%, signal=29%
BP	GO:0006333	chromatin assembly or disassembly	88	0.434472303	1.671160249	0.0002012	0.005556822	0.004297	4825	tags=58%, list=38%, signal=36%
BP	GO:0045069	regulation of viral genome replication	67	0.46547623	1.738329869	0.000205582	0.00565306	0.004372	3690	tags=57%, list=29%, signal=40%
MF	GO:0060589	nucleoside-triphosphatase regulator activity	217	0.355642584	1.454731615	0.00021674	0.005885507	0.004551	4124	tags=43%, list=33%, signal=30%
BP	GO:0048285	organelle fission	229	0.361644743	1.48406136	0.000216957	0.005885507	0.004551	5308	tags=55%, list=42%, signal=33%
BP	GO:0050852	T cell receptor signaling pathway	136	0.393720827	1.568473637	0.000217393	0.005885507	0.004551	2281	tags=34%, list=18%, signal=28%
BP	GO:0043534	blood vessel endothelial cell migration	82	0.444697558	1.699883278	0.000217958	0.005885507	0.004551	3739	tags=50%, list=30%, signal=35%
BP	GO:0007093	mitotic cell cycle checkpoint	107	0.418253893	1.638123345	0.000218709	0.005885507	0.004551	4466	tags=52%, list=35%, signal=34%
BP	GO:0030155	regulation of cell adhesion	414	0.327745838	1.383634532	0.000223326	0.005984174	0.004628	3853	tags=40%, list=31%, signal=29%
BP	GO:0050907	detection of chemical stimulus involved in sensory perception	18	-0.595907568	-2.36697024	0.000228241	0.006089983	0.004709	1665	tags=83%, list=13%, signal=72%

BP	GO:0034470	ncRNA processing	281	0.345174902	1.430405102	0.000230357	0.006120509	0.004733	5032	tags=52%, list=40%, signal=32%
CC	GO:0005938	cell cortex	167	0.382296524	1.53660732	0.00023539	0.006227933	0.004816	4865	tags=53%, list=39%, signal=33%
MF	GO:0042826	histone deacetylase binding	85	0.432270169	1.659118597	0.000237772	0.006264647	0.004844	3824	tags=54%, list=30%, signal=38%
BP	GO:1903901	negative regulation of viral life cycle	54	0.488983126	1.778148207	0.000239457	0.006282747	0.004858	4084	tags=59%, list=32%, signal=40%
CC	GO:0034399	nuclear periphery	100	0.418960021	1.629131885	0.000242389	0.006333289	0.004898	4272	tags=51%, list=34%, signal=34%
BP	GO:0000226	microtubule cytoskeleton organization	302	0.34291321	1.425025938	0.000243798	0.006343779	0.004906	5949	tags=61%, list=47%, signal=33%
BP	GO:1902624	positive regulation of neutrophil migration	16	0.679176091	1.963223627	0.000246274	0.006381849	0.004935	1406	tags=44%, list=11%, signal=39%
BP	GO:1902850	microtubule cytoskeleton organization involved in mitosis	89	0.441372834	1.699595646	0.000247507	0.006387498	0.004939	3428	tags=49%, list=27%, signal=36%
BP	GO:0050851	antigen receptor-mediated signaling pathway	160	0.382015175	1.532578429	0.000249351	0.00640883	0.004956	3151	tags=38%, list=25%, signal=29%
BP	GO:2001251	negative regulation of chromosome organization	84	0.434012418	1.66315915	0.000261651	0.006697615	0.005179	4501	tags=54%, list=36%, signal=35%
BP	GO:0031344	regulation of cell projection organization	388	0.328352261	1.382617278	0.000266582	0.006796221	0.005256	5264	tags=52%, list=42%, signal=31%
BP	GO:0035195	gene silencing by miRNA	97	0.428707812	1.661250624	0.000269817	0.006848807	0.005296	4096	tags=52%, list=32%, signal=35%
MF	GO:0004984	olfactory receptor activity	17	-0.61129582	-2.407658549	0.000271908	0.006848807	0.005296	1665	tags=53%, list=13%, signal=46%
BP	GO:0050911	detection of chemical stimulus involved in sensory perception of smell	17	-0.61129582	-2.407658549	0.000271908	0.006848807	0.005296	1665	tags=53%, list=13%, signal=46%
BP	GO:0051090	regulation of DNA-binding transcription factor activity	256	0.352877772	1.454941512	0.000275971	0.006916749	0.005349	4350	tags=45%, list=35%, signal=30%
BP	GO:0051607	defense response to virus	162	0.379553952	1.52382959	0.000276802	0.006916749	0.005349	4649	tags=53%, list=37%, signal=34%
CC	GO:0099081	supramolecular polymer	448	0.322103688	1.362681631	0.000280253	0.006962212	0.005384	5344	tags=53%, list=42%, signal=32%
BP	GO:0101023	vascular endothelial cell proliferation	13	0.701631261	1.906654496	0.000281938	0.006962212	0.005384	1741	tags=46%, list=14%, signal=40%
BP	GO:1905562	regulation of vascular endothelial cell proliferation	13	0.701631261	1.906654496	0.000281938	0.006962212	0.005384	1741	tags=46%, list=14%, signal=40%
CC	GO:0022627	cytosolic small ribosomal subunit	43	-0.379573606	-1.924471787	0.000285982	0.007013416	0.005423	1452	tags=30%, list=12%, signal=27%
BP	GO:0006310	DNA recombination	138	0.392385163	1.562241351	0.000286239	0.007013416	0.005423	2895	tags=40%, list=23%, signal=31%
BP	GO:0090287	regulation of cellular response to growth factor stimulus	161	0.380265308	1.52599924	0.000295102	0.007202555	0.00557	4281	tags=46%, list=34%, signal=31%
BP	GO:0019079	viral genome replication	83	0.440200978	1.684848949	0.000303945	0.007389748	0.005714	4084	tags=54%, list=32%, signal=37%
BP	GO:0022406	membrane docking	109	0.412951719	1.619545773	0.000305478	0.007398446	0.005721	5265	tags=61%, list=42%, signal=36%
MF	GO:0016628	oxidoreductase activity, acting on the CH-CH group of donors, NAD or NADP as acceptor	20	-0.54071061	-2.197360634	0.000308577	0.007444858	0.005757	2177	tags=60%, list=17%, signal=50%
MF	GO:0031490	chromatin DNA binding	57	0.477402612	1.746784799	0.000314995	0.007570692	0.005854	3660	tags=54%, list=29%, signal=39%
BP	GO:0051099	positive regulation of binding	123	0.400157865	1.583737874	0.000322553	0.007650376	0.005916	5159	tags=58%, list=41%, signal=34%
BP	GO:0006475	internal protein amino acid acetylation	107	0.412603606	1.615993562	0.000324506	0.007650376	0.005916	3823	tags=47%, list=30%, signal=33%
BP	GO:0018393	internal peptidyl-lysine acetylation	107	0.412603606	1.615993562	0.000324506	0.007650376	0.005916	3823	tags=47%, list=30%, signal=33%
CC	GO:0099512	supramolecular fiber	444	0.321553582	1.359852107	0.000325821	0.007650376	0.005916	5344	tags=53%, list=42%, signal=32%
BP	GO:0032092	positive regulation of protein binding	63	0.468752691	1.741741322	0.000326156	0.007650376	0.005916	5001	tags=65%, list=40%, signal=39%
BP	GO:0006406	mRNA export from nucleus	80	0.449522171	1.709981175	0.000326815	0.007650376	0.005916	4226	tags=55%, list=34%, signal=37%
BP	GO:0071427	mRNA-containing ribonucleoprotein complex export from nucleus	80	0.449522171	1.709981175	0.000326815	0.007650376	0.005916	4226	tags=55%, list=34%, signal=37%
MF	GO:0051287	NAD binding	44	-0.373365819	-1.92098151	0.000329759	0.007675162	0.005935	1904	tags=41%, list=15%, signal=35%
MF	GO:0015078	proton transmembrane transporter activity	60	-0.338383911	-1.913656476	0.000330311	0.007675162	0.005935	1486	tags=28%, list=12%, signal=25%
CC	GO:0097525	spliceosomal snRNP complex	45	0.512310715	1.808538289	0.000332054	0.00768729	0.005945	4178	tags=64%, list=33%, signal=43%
BP	GO:0006352	DNA-templated transcription, initiation	159	0.38256785	1.534676227	0.000336279	0.00775658	0.005998	4840	tags=57%, list=38%, signal=35%
BP	GO:0006635	fatty acid beta-oxidation	49	-0.354669721	-1.944319865	0.000341981	0.00785932	0.006078	2794	tags=45%, list=22%, signal=35%
BP	GO:0030900	forebrain development	196	0.368842114	1.500892214	0.000345083	0.007874956	0.00609	5195	tags=56%, list=41%, signal=34%

BP	GO:0043488	regulation of mRNA stability	137	0.384102731	1.530609235	0.000345429	0.007874956	0.00609	4278	tags=47%, list=34%, signal=32%
BP	GO:0034446	substrate adhesion-dependent cell spreading	72	0.447034217	1.681726037	0.000346413	0.007874956	0.00609	3469	tags=50%, list=28%, signal=36%
BP	GO:0042692	muscle cell differentiation	186	0.369697245	1.496172996	0.000350004	0.007899554	0.006109	3853	tags=42%, list=31%, signal=30%
BP	GO:0046578	regulation of Ras protein signal transduction	122	0.405646827	1.604307523	0.000350004	0.007899554	0.006109	3076	tags=44%, list=24%, signal=34%
BP	GO:0006721	terpenoid metabolic process	56	-0.342244164	-1.856515147	0.000353832	0.007939898	0.00614	2421	tags=48%, list=19%, signal=39%
CC	GO:0070603	SWI/SNF superfamily-type complex	63	0.4668488	1.734667044	0.000354313	0.007939898	0.00614	4825	tags=65%, list=38%, signal=40%
BP	GO:0120031	plasma membrane bounded cell projection assembly	291	0.343253588	1.424459582	0.000357719	0.007987793	0.006177	5886	tags=60%, list=47%, signal=33%
BP	GO:0006720	isoprenoid metabolic process	70	-0.32253247	-1.873135969	0.000369824	0.008216924	0.006354	2421	tags=44%, list=19%, signal=36%
BP	GO:0010596	negative regulation of endothelial cell migration	35	0.549641239	1.858164501	0.00037059	0.008216924	0.006354	4332	tags=63%, list=34%, signal=41%
BP	GO:1902807	negative regulation of cell cycle G1/S phase transition	77	0.43909276	1.661797586	0.000383611	0.008456969	0.00654	3332	tags=45%, list=26%, signal=34%
BP	GO:0016573	histone acetylation	106	0.413072673	1.61506711	0.000384343	0.008456969	0.00654	3823	tags=47%, list=30%, signal=33%
CC	GO:0099513	polymeric cytoskeletal fiber	322	0.339639587	1.414891292	0.000385445	0.008456969	0.00654	5344	tags=55%, list=42%, signal=33%
BP	GO:0006897	endocytosis	318	0.33461779	1.393670835	0.000390007	0.008514862	0.006585	4520	tags=45%, list=36%, signal=30%
BP	GO:0031503	protein-containing complex localization	181	0.371871938	1.5015693	0.000390788	0.008514862	0.006585	4865	tags=53%, list=39%, signal=33%
BP	GO:0006338	chromatin remodeling	115	0.408147486	1.606659037	0.00039695	0.008619291	0.006665	5154	tags=61%, list=41%, signal=36%
MF	GO:0035257	nuclear hormone receptor binding	96	0.421526511	1.630262811	0.000399361	0.008641835	0.006683	3824	tags=47%, list=30%, signal=33%
BP	GO:0031345	negative regulation of cell projection organization	113	0.398857659	1.56795278	0.000410717	0.008857139	0.006849	3528	tags=44%, list=28%, signal=32%
BP	GO:0120035	regulation of plasma membrane bounded cell projection organization	384	0.328403432	1.382415639	0.000416959	0.008961051	0.00693	5264	tags=52%, list=42%, signal=31%
BP	GO:0071824	protein-DNA complex subunit organization	139	0.387442966	1.543172595	0.000419052	0.008975403	0.006941	4825	tags=58%, list=38%, signal=36%
BP	GO:0022604	regulation of cell morphogenesis	296	0.338072076	1.404761245	0.000426072	0.009094838	0.007033	5193	tags=54%, list=41%, signal=33%
CC	GO:0030532	small nuclear ribonucleoprotein complex	49	0.492767297	1.767864043	0.000441819	0.009399098	0.007268	4178	tags=61%, list=33%, signal=41%
CC	GO:0005657	replication fork	47	0.499176798	1.775158037	0.000451321	0.009568918	0.0074	4160	tags=57%, list=33%, signal=39%
MF	GO:0046875	ephrin receptor binding	22	0.59660243	1.862164885	0.00046498	0.009825438	0.007598	2847	tags=59%, list=23%, signal=46%
BP	GO:0034308	primary alcohol metabolic process	49	-0.349483572	-1.915889099	0.000466876	0.009832493	0.007603	1841	tags=43%, list=15%, signal=37%
BP	GO:0031123	RNA 3'-end processing	93	0.418313164	1.616157029	0.000468613	0.009836192	0.007606	5032	tags=55%, list=40%, signal=33%
MF	GO:0003697	single-stranded DNA binding	71	0.447938018	1.68773998	0.000470997	0.009853379	0.00762	4590	tags=56%, list=36%, signal=36%
BP	GO:1901343	negative regulation of vasculature development	75	0.443515559	1.672857295	0.000472921	0.009860877	0.007625	4634	tags=57%, list=37%, signal=36%
BP	GO:0001959	regulation of cytokine-mediated signaling pathway	110	0.406624713	1.595506081	0.000475008	0.009871696	0.007634	4636	tags=55%, list=37%, signal=35%
BP	GO:0051345	positive regulation of hydrolase activity	446	0.317415735	1.343075667	0.000480845	0.009872661	0.007635	4006	tags=39%, list=32%, signal=28%
BP	GO:0040017	positive regulation of locomotion	314	0.331736061	1.381531447	0.000481326	0.009872661	0.007635	4186	tags=43%, list=33%, signal=30%
BP	GO:0043161	proteasome-mediated ubiquitin-dependent protein catabolic process	315	0.332772376	1.385536959	0.000481326	0.009872661	0.007635	3749	tags=39%, list=30%, signal=28%
BP	GO:0051493	regulation of cytoskeleton organization	339	0.33363518	1.393151349	0.000481326	0.009872661	0.007635	3473	tags=37%, list=28%, signal=28%
MF	GO:0017048	Rho GTPase binding	100	0.410094732	1.594659086	0.000496451	0.010127242	0.007831	3844	tags=47%, list=30%, signal=33%
BP	GO:2000736	regulation of stem cell differentiation	86	0.42569038	1.635171521	0.000496954	0.010127242	0.007831	3610	tags=45%, list=29%, signal=33%
BP	GO:0019882	antigen processing and presentation	165	0.373826624	1.502883377	0.000500663	0.010169909	0.007864	3744	tags=42%, list=30%, signal=30%
BP	GO:0030031	cell projection assembly	298	0.338805968	1.407970813	0.000508321	0.010292269	0.007959	5886	tags=60%, list=47%, signal=33%
BP	GO:0070848	response to growth factor	418	0.324745327	1.371719135	0.000526638	0.010626865	0.008218	5191	tags=50%, list=41%, signal=30%
BP	GO:0061013	regulation of mRNA catabolic process	151	0.378487695	1.515381029	0.000528221	0.010626865	0.008218	4278	tags=47%, list=34%, signal=31%
BP	GO:0044089	positive regulation of cellular component biogenesis	313	0.330339193	1.376181279	0.000535797	0.010744944	0.008309	5279	tags=52%, list=42%, signal=31%

BP	GO:0071103	DNA conformation change	171	0.370028918	1.48834027	0.000537946	0.010753805	0.008316	4789	tags=52%, list=38%, signal=33%
BP	GO:0099504	synaptic vesicle cycle	92	0.41801768	1.614405307	0.000542298	0.010783299	0.008339	4057	tags=47%, list=32%, signal=32%
BP	GO:0006606	protein import into nucleus	100	0.408831925	1.589748642	0.000542847	0.010783299	0.008339	3234	tags=42%, list=26%, signal=31%
MF	GO:0070491	repressing transcription factor binding	54	0.474272031	1.724652482	0.000557518	0.01103422	0.008533	4501	tags=54%, list=36%, signal=35%
MF	GO:0016620	oxidoreductase activity, acting on the aldehyde or oxo group of donors, NAD or NADP as acceptor	27	-0.467174489	-2.07574334	0.000558983	0.01103422	0.008533	1733	tags=48%, list=14%, signal=42%
BP	GO:0046847	filopodium assembly	32	0.539122753	1.80356001	0.000571143	0.011201925	0.008662	1646	tags=41%, list=13%, signal=35%
MF	GO:0008047	enzyme activator activity	323	0.335645001	1.398092926	0.000573004	0.011201925	0.008662	4006	tags=41%, list=32%, signal=29%
BP	GO:1901342	regulation of vasculature development	207	0.357657294	1.457166417	0.000574152	0.011201925	0.008662	4634	tags=48%, list=37%, signal=31%
BP	GO:0019395	fatty acid oxidation	66	-0.306204129	-1.756147595	0.000574595	0.011201925	0.008662	1659	tags=30%, list=13%, signal=26%
CC	GO:0005912	adherens junction	95	0.415866225	1.607417074	0.000579964	0.0112717	0.008716	3609	tags=48%, list=29%, signal=35%
BP	GO:0009408	response to heat	109	0.40633089	1.5935797	0.000586276	0.01135932	0.008784	4006	tags=45%, list=32%, signal=31%
BP	GO:0002429	immune response-activating cell surface receptor signaling pathway	229	0.354978988	1.456707473	0.000591932	0.011398758	0.008815	3554	tags=39%, list=28%, signal=28%
BP	GO:0002757	immune response-activating signal transduction	229	0.354978988	1.456707473	0.000591932	0.011398758	0.008815	3554	tags=39%, list=28%, signal=28%
CC	GO:0005911	cell-cell junction	274	0.34388641	1.423176181	0.000601109	0.01151412	0.008904	3495	tags=39%, list=28%, signal=29%
CC	GO:0030496	midbody	118	0.398782409	1.572468265	0.000602919	0.01151412	0.008904	5302	tags=61%, list=42%, signal=36%
BP	GO:0007052	mitotic spindle organization	70	0.449097018	1.687551483	0.000603408	0.01151412	0.008904	3428	tags=50%, list=27%, signal=37%
BP	GO:0071363	cellular response to growth factor stimulus	399	0.322272278	1.357715836	0.000609066	0.011586983	0.00896	5191	tags=49%, list=41%, signal=30%
BP	GO:0060964	regulation of gene silencing by miRNA	62	0.467172737	1.732808329	0.000618442	0.011702273	0.009049	4096	tags=58%, list=32%, signal=39%
BP	GO:0051098	regulation of binding	240	0.347507026	1.429726118	0.000618843	0.011702273	0.009049	4625	tags=48%, list=37%, signal=31%
MF	GO:0017049	GTP-Rho binding	15	0.660796036	1.875633991	0.000621281	0.011713191	0.009058	1980	tags=47%, list=16%, signal=39%
BP	GO:1903313	positive regulation of mRNA metabolic process	55	0.474747491	1.732818474	0.000631788	0.011875731	0.009184	3640	tags=51%, list=29%, signal=36%
MF	GO:1990782	protein tyrosine kinase binding	56	0.466806662	1.708826276	0.000648772	0.012158682	0.009402	3234	tags=50%, list=26%, signal=37%
BP	GO:0035633	maintenance of blood-brain barrier	25	0.568875309	1.816323623	0.000662195	0.012353036	0.009553	1114	tags=44%, list=9%, signal=40%
BP	GO:0001937	negative regulation of endothelial cell proliferation	22	0.590083132	1.841816312	0.000663066	0.012353036	0.009553	4143	tags=68%, list=33%, signal=46%
BP	GO:2000134	negative regulation of G1/S transition of mitotic cell cycle	75	0.437020195	1.648358005	0.000678941	0.012611472	0.009752	3332	tags=45%, list=26%, signal=34%
BP	GO:0003197	endocardial cushion development	25	0.567602439	1.812259562	0.000682727	0.012644512	0.009778	3761	tags=64%, list=30%, signal=45%
BP	GO:0034660	ncRNA metabolic process	322	0.33510753	1.396011375	0.000710522	0.013108554	0.010137	5032	tags=51%, list=40%, signal=31%
BP	GO:0006473	protein acetylation	124	0.387777183	1.537146258	0.000711946	0.013108554	0.010137	4841	tags=53%, list=38%, signal=33%
CC	GO:0072686	mitotic spindle	73	0.437543146	1.646214155	0.000717131	0.013165528	0.010181	3473	tags=45%, list=28%, signal=33%
MF	GO:0019842	vitamin binding	68	-0.303117121	-1.703345326	0.000725454	0.013279599	0.010269	2239	tags=40%, list=18%, signal=33%
BP	GO:0010975	regulation of neuron projection development	288	0.332570791	1.37941759	0.000728858	0.01329138	0.010278	5193	tags=52%, list=41%, signal=32%
MF	GO:0004518	nuclease activity	127	0.382472297	1.518264077	0.000730319	0.01329138	0.010278	3987	tags=46%, list=32%, signal=32%
BP	GO:0034035	purine ribonucleoside bisphosphate metabolic process	14	0.667732078	1.844489035	0.000745972	0.013498239	0.010438	1089	tags=50%, list=9%, signal=46%
BP	GO:0050427	3'-phosphoadenosine 5'-phosphosulfate metabolic process	14	0.667732078	1.844489035	0.000745972	0.013498239	0.010438	1089	tags=50%, list=9%, signal=46%
BP	GO:0038095	Fc-epsilon receptor signaling pathway	92	0.41287448	1.594542009	0.000755505	0.013608031	0.010523	4128	tags=45%, list=33%, signal=30%
BP	GO:0002768	immune response-regulating cell surface receptor signaling pathway	254	0.340774466	1.404767703	0.000756362	0.013608031	0.010523	4308	tags=42%, list=34%, signal=28%
BP	GO:0006323	DNA packaging	88	0.415613885	1.598622972	0.000812767	0.014581187	0.011276	4736	tags=57%, list=38%, signal=36%
BP	GO:0010769	regulation of cell morphogenesis involved in differentiation	186	0.359734763	1.455854608	0.000823006	0.014722928	0.011385	3716	tags=44%, list=29%, signal=31%

BP	GO:0000209	protein polyubiquitination	248	0.346036619	1.42602506	0.000829705	0.014800719	0.011445	5280	tags=54%, list=42%, signal=32%
BP	GO:0006367	transcription initiation from RNA polymerase II promoter	129	0.383162382	1.52180499	0.000841397	0.01496689	0.011574	4716	tags=56%, list=37%, signal=35%
BP	GO:0043009	chordate embryonic development	372	0.321440226	1.35073315	0.000847194	0.015027548	0.011621	4139	tags=42%, list=33%, signal=29%
BP	GO:0097064	ncRNA export from nucleus	30	0.535909497	1.766847783	0.000889659	0.015714587	0.012152	4001	tags=70%, list=32%, signal=48%
BP	GO:0016525	negative regulation of angiogenesis	70	0.4425252	1.6628569	0.0008934	0.0157146	0.012	4941	tags=60%, list=39%, signal=37%
BP	GO:2000181	negative regulation of blood vessel morphogenesis	70	0.442525222	1.662856944	0.000893413	0.015714587	0.012152	4941	tags=60%, list=39%, signal=37%
BP	GO:0010498	proteasomal protein catabolic process	350	0.320228308	1.340107673	9.12E-04	0.016000633	0.012373	3749	tags=39%, list=30%, signal=28%
BP	GO:0010594	regulation of endothelial cell migration	113	0.388588137	1.527582173	9.18E-04	0.016052502	0.012413	3739	tags=43%, list=30%, signal=31%
BP	GO:0002009	morphogenesis of an epithelium	324	0.331334644	1.380308953	9.21E-04	0.016071905	0.012428	3866	tags=42%, list=31%, signal=30%
BP	GO:0051028	mRNA transport	106	0.399346003	1.561397394	9.39E-04	0.01633399	0.012631	4764	tags=53%, list=38%, signal=33%
BP	GO:0009792	embryo development ending in birth or egg hatching	379	0.321693583	1.353563258	9.48E-04	0.016433241	0.012708	4139	tags=42%, list=33%, signal=29%
BP	GO:0045787	positive regulation of cell cycle	221	0.352976582	1.443191487	9.50E-04	0.016433241	0.012708	3657	tags=40%, list=29%, signal=29%
MF	GO:0003724	RNA helicase activity	55	0.465205569	1.697990658	9.64E-04	0.016636208	0.012865	4214	tags=56%, list=33%, signal=38%
BP	GO:0000723	telomere maintenance	106	0.398244286	1.557089807	9.76E-04	0.016749513	0.012952	3954	tags=43%, list=31%, signal=30%
BP	GO:0032200	telomere organization	106	0.398244286	1.557089807	9.76E-04	0.016749513	0.012952	3954	tags=43%, list=31%, signal=30%
CC	GO:0000922	spindle pole	89	0.423708377	1.631575071	9.81E-04	0.016788704	0.012983	3141	tags=45%, list=25%, signal=34%
BP	GO:0034508	centromere complex assembly	20	0.598223782	1.829413462	0.000995836	0.016993973	0.013141	4249	tags=75%, list=34%, signal=50%
CC	GO:0001772	immunological synapse	25	-0.450820804	-2.000129239	0.001010055	0.017190046	0.013293	2743	tags=56%, list=22%, signal=44%
MF	GO:0003727	single-stranded RNA binding	58	0.461856366	1.69657378	0.001022646	0.017357421	0.013423	3884	tags=55%, list=31%, signal=38%
BP	GO:1905114	cell surface receptor signaling pathway involved in cell-cell signaling	348	0.323792849	1.354949422	0.00104111	0.017623299	0.013628	5171	tags=49%, list=41%, signal=30%
BP	GO:0003279	cardiac septum development	65	0.449568379	1.677190289	0.001047831	0.017689528	0.013679	4052	tags=57%, list=32%, signal=39%
BP	GO:0051272	positive regulation of cellular component movement	316	0.324221625	1.350311166	0.001059536	0.017795751	0.013761	4186	tags=43%, list=33%, signal=29%
BP	GO:0000288	nuclear-transcribed mRNA catabolic process, deadenylation-dependent decay	55	0.463442396	1.691555114	0.001059776	0.017795751	0.013761	4213	tags=60%, list=33%, signal=40%
BP	GO:0002479	antigen processing and presentation of exogenous peptide antigen via MHC class I, TAP-dependent	67	0.442185357	1.651349659	0.001065619	0.017846282	0.013801	4554	tags=57%, list=36%, signal=36%
BP	GO:2000278	regulation of DNA biosynthetic process	78	0.420124051	1.592866081	0.001078957	0.017977884	0.013902	3950	tags=46%, list=31%, signal=32%
BP	GO:0010632	regulation of epithelial cell migration	156	0.376009076	1.506199998	0.001081206	0.017977884	0.013902	3761	tags=43%, list=30%, signal=31%
CC	GO:0090575	RNA polymerase II transcription regulator complex	118	0.390687967	1.540550475	0.001082291	0.017977884	0.013902	5047	tags=58%, list=40%, signal=35%
BP	GO:1905314	semi-lunar valve development	21	0.57965891	1.786469324	0.001085788	0.017977884	0.013902	2891	tags=52%, list=23%, signal=40%
BP	GO:0007051	spindle organization	107	0.397563058	1.557086106	0.001087752	0.017977884	0.013902	3428	tags=44%, list=27%, signal=32%
CC	GO:0000151	ubiquitin ligase complex	192	0.356128009	1.445419662	0.001098586	0.018109406	0.014004	5264	tags=57%, list=42%, signal=34%
BP	GO:0048525	negative regulation of viral process	64	0.451450597	1.681943902	0.001107875	0.018214849	0.014086	4126	tags=56%, list=33%, signal=38%
BP	GO:0046822	regulation of nucleocytoplasmic transport	81	0.435658397	1.661336076	0.001131792	0.018553666	0.014348	4810	tags=58%, list=38%, signal=36%
BP	GO:0030111	regulation of Wnt signaling pathway	221	0.351551876	1.437366387	0.001134375	0.018553666	0.014348	5393	tags=56%, list=43%, signal=32%
BP	GO:0030335	positive regulation of cell migration	300	0.333186194	1.385185658	0.001150517	0.018768921	0.014514	4186	tags=43%, list=33%, signal=30%
BP	GO:0002526	acute inflammatory response	42	-0.345106355	-1.75890532	0.001154103	0.018778782	0.014522	1004	tags=26%, list=8%, signal=24%
BP	GO:0048013	ephrin receptor signaling pathway	64	0.449178089	1.673477349	0.001164687	0.018902157	0.014617	3770	tags=50%, list=30%, signal=35%
MF	GO:0017016	Ras GTPase binding	266	0.334550506	1.383130464	0.001170093	0.018941076	0.014647	4074	tags=43%, list=32%, signal=30%
BP	GO:1902622	regulation of neutrophil migration	22	0.57986392	1.809919261	0.001185774	0.019132749	0.014795	1406	tags=36%, list=11%, signal=32%

MF	GO:0016829	lyase activity	104	-0.252650069	-1.606485284	0.001188011	0.019132749	0.014795	1271	tags=26%, list=10%, signal=24%
BP	GO:0060485	mesenchyme development	153	0.366994523	1.46963471	0.001192096	0.019149564	0.014808	4121	tags=47%, list=33%, signal=32%
BP	GO:0051052	regulation of DNA metabolic process	209	0.350750416	1.43065842	0.001208154	0.019211683	0.014856	3954	tags=40%, list=31%, signal=28%
BP	GO:0001701	in utero embryonic development	235	0.34180764	1.404903057	0.001209365	0.019211683	0.014856	4137	tags=43%, list=33%, signal=30%
CC	GO:0000123	histone acetyltransferase complex	55	0.461405901	1.684121946	0.001212532	0.019211683	0.014856	3823	tags=51%, list=30%, signal=36%
CC	GO:0031248	protein acetyltransferase complex	55	0.461405901	1.684121946	0.001212532	0.019211683	0.014856	3823	tags=51%, list=30%, signal=36%
CC	GO:1902493	acetyltransferase complex	55	0.461405901	1.684121946	0.001212532	0.019211683	0.014856	3823	tags=51%, list=30%, signal=36%
MF	GO:0016835	carbon-oxygen lyase activity	41	-0.356562018	-1.801218351	0.001214269	0.019211683	0.014856	1271	tags=34%, list=10%, signal=31%
BP	GO:0006399	tRNA metabolic process	117	0.387306682	1.527652813	0.001230294	0.019416437	0.015015	4213	tags=44%, list=33%, signal=30%
BP	GO:0007004	telomere maintenance via telomerase	51	0.467525853	1.686174467	0.001239332	0.01951019	0.015087	3586	tags=43%, list=28%, signal=31%
BP	GO:0045665	negative regulation of neuron differentiation	131	0.372433873	1.482188618	0.001247541	0.019541711	0.015112	3610	tags=43%, list=29%, signal=31%
BP	GO:1901796	regulation of signal transduction by p53 class mediator	135	0.377415807	1.503611066	0.001247541	0.019541711	0.015112	3435	tags=41%, list=27%, signal=30%
BP	GO:0090305	nucleic acid phosphodiester bond hydrolysis	199	0.354533268	1.443193325	0.001301681	0.020309175	0.015705	5005	tags=52%, list=40%, signal=32%
BP	GO:0007160	cell-matrix adhesion	136	0.377210418	1.502700785	0.001302987	0.020309175	0.015705	5001	tags=55%, list=40%, signal=34%
BP	GO:0043648	dicarboxylic acid metabolic process	55	-0.31990005	-1.727811884	0.001313447	0.020421667	0.015792	2239	tags=42%, list=18%, signal=35%
CC	GO:0035267	NuA4 histone acetyltransferase complex	15	0.641554313	1.821017397	0.001338692	0.020711894	0.016017	2726	tags=60%, list=22%, signal=47%
CC	GO:0043189	H4/H2A histone acetyltransferase complex	15	0.641554313	1.821017397	0.001338692	0.020711894	0.016017	2726	tags=60%, list=22%, signal=47%
BP	GO:0001523	retinoid metabolic process	46	-0.338660448	-1.795049094	0.001342075	0.020713343	0.016018	2421	tags=50%, list=19%, signal=41%
CC	GO:1902554	serine/threonine protein kinase complex	58	0.455264087	1.672357835	0.001347591	0.020729596	0.01603	2766	tags=40%, list=22%, signal=31%
BP	GO:0007088	regulation of mitotic nuclear division	93	0.402082733	1.5534506	0.001352156	0.020729596	0.01603	5448	tags=61%, list=43%, signal=35%
BP	GO:0019221	cytokine-mediated signaling pathway	462	0.306885456	1.299395519	0.001353004	0.020729596	0.01603	4128	tags=40%, list=33%, signal=28%
BP	GO:2000147	positive regulation of cell motility	307	0.324850224	1.351273533	0.001371412	0.020960628	0.016209	4186	tags=43%, list=33%, signal=29%
BP	GO:0065004	protein-DNA complex assembly	109	0.392298271	1.538545496	0.00138246	0.021063966	0.016289	4789	tags=58%, list=38%, signal=36%
BP	GO:0043968	histone H2A acetylation	14	0.649683748	1.794633788	0.001384863	0.021063966	0.016289	2726	tags=64%, list=22%, signal=50%
BP	GO:0033962	P-body assembly	14	0.648381822	1.791037454	0.001407753	0.021360527	0.016518	3987	tags=86%, list=32%, signal=59%
BP	GO:0034440	lipid oxidation	69	-0.295156889	-1.657379441	0.001436474	0.021743933	0.016815	1659	tags=42%, list=13%, signal=37%
BP	GO:0090100	positive regulation of transmembrane receptor protein serine/threonine kinase signaling pathway	57	0.454025589	1.661249805	0.00148293	0.022393316	0.017317	3610	tags=49%, list=29%, signal=35%
MF	GO:0004842	ubiquitin-protein transferase activity	277	0.337557798	1.397459018	0.001501767	0.02257399	0.017456	5248	tags=54%, list=42%, signal=32%
CC	GO:0120114	Sm-like protein family complex	57	0.453866742	1.660668595	0.001502065	0.02257399	0.017456	4178	tags=56%, list=33%, signal=38%
BP	GO:0048524	positive regulation of viral process	78	0.415112568	1.573865451	0.001529295	0.022928498	0.017731	4948	tags=59%, list=39%, signal=36%
CC	GO:0097447	dendritic tree	297	0.324267214	1.34749314	0.001537083	0.022990528	0.017779	4144	tags=43%, list=33%, signal=29%
BP	GO:0017144	drug metabolic process	13	-0.610470108	-2.128110399	0.001548075	0.023100062	0.017863	2548	tags=69%, list=20%, signal=55%
BP	GO:0000070	mitotic sister chromatid segregation	94	0.405126354	1.565966443	0.001555733	0.023159461	0.017909	2885	tags=40%, list=23%, signal=31%
BP	GO:0016358	dendrite development	144	0.36713124	1.466137933	0.001561732	0.023193926	0.017936	4113	tags=45%, list=33%, signal=31%
BP	GO:0006959	humoral immune response	78	-0.273386775	-1.674632891	0.001587157	0.023429887	0.018118	1625	tags=31%, list=13%, signal=27%
BP	GO:0000724	double-strand break repair via homologous recombination	70	0.43227863	1.624353789	0.001588823	0.023429887	0.018118	4369	tags=53%, list=35%, signal=35%
BP	GO:0000725	recombinational repair	70	0.43227863	1.624353789	0.001588823	0.023429887	0.018118	4369	tags=53%, list=35%, signal=35%
BP	GO:0051321	meiotic cell cycle	103	0.389500748	1.517566061	0.001594608	0.023429887	0.018118	4450	tags=49%, list=35%, signal=32%
BP	GO:0031570	DNA integrity checkpoint	101	0.400654409	1.557143999	0.001596224	0.023429887	0.018118	4416	tags=49%, list=35%, signal=32%

MF	GO:0008408	3'-5' exonuclease activity	40	0.486540976	1.679994484	0.00160883	0.023538808	0.018203	4407	tags=62%, list=35%, signal=41%
MF	GO:0002039	p53 binding	49	0.468455012	1.680640693	0.001611121	0.023538808	0.018203	3196	tags=49%, list=25%, signal=37%
BP	GO:0043537	negative regulation of blood vessel endothelial cell migration	23	0.571872935	1.80219319	0.001620956	0.023589144	0.018241	4332	tags=70%, list=34%, signal=46%
MF	GO:0051427	hormone receptor binding	110	0.38917891	1.527052581	0.001622058	0.023589144	0.018241	3824	tags=45%, list=30%, signal=31%
BP	GO:1902036	regulation of hematopoietic stem cell differentiation	63	0.438423895	1.629048811	0.001658771	0.024019399	0.018574	3513	tags=44%, list=28%, signal=32%
BP	GO:0006409	tRNA export from nucleus	27	0.545669453	1.764341712	0.001663087	0.024019399	0.018574	4001	tags=70%, list=32%, signal=48%
BP	GO:0071431	tRNA-containing ribonucleoprotein complex export from nucleus	27	0.545669453	1.764341712	0.001663087	0.024019399	0.018574	4001	tags=70%, list=32%, signal=48%
BP	GO:0009081	branched-chain amino acid metabolic process	22	-0.462166738	-1.956856607	0.001692998	0.024395448	0.018865	475	tags=36%, list=4%, signal=35%
CC	GO:0032154	cleavage furrow	32	0.519884797	1.739202112	0.001706905	0.024539676	0.018977	5193	tags=72%, list=41%, signal=42%
CC	GO:0030427	site of polarized growth	114	0.389750502	1.533982624	0.001711303	0.024546865	0.018982	5328	tags=64%, list=42%, signal=37%
BP	GO:0009226	nucleotide-sugar biosynthetic process	18	-0.511409624	-2.031340806	0.001726409	0.024707273	0.019106	2263	tags=67%, list=18%, signal=55%
MF	GO:0051020	GTPase binding	345	0.318703384	1.332906859	0.001739571	0.024839178	0.019208	2678	tags=32%, list=21%, signal=26%
BP	GO:0045744	negative regulation of G protein-coupled receptor signaling pathway	25	0.546572131	1.745113309	0.001743613	0.02484057	0.019209	1411	tags=44%, list=11%, signal=39%
BP	GO:0071559	response to transforming growth factor beta	160	0.361523324	1.450368687	0.001783514	0.025351661	0.019604	5191	tags=54%, list=41%, signal=32%
MF	GO:0004527	exonuclease activity	57	0.450714634	1.64913524	0.00178908	0.025373505	0.019621	4463	tags=56%, list=35%, signal=36%
BP	GO:0051656	establishment of organelle localization	268	0.330151581	1.365418284	0.001798388	0.025442076	0.019674	4097	tags=43%, list=32%, signal=30%
BP	GO:0071560	cellular response to transforming growth factor beta stimulus	155	0.362178217	1.450225832	0.001801996	0.025442076	0.019674	5191	tags=55%, list=41%, signal=33%
MF	GO:0003678	DNA helicase activity	58	0.450834407	1.65608594	0.001806339	0.02544634	0.019678	2729	tags=45%, list=22%, signal=35%
MF	GO:0008094	DNA-dependent ATPase activity	78	0.413384405	1.567313263	0.001810757	0.025451642	0.019682	2816	tags=41%, list=22%, signal=32%
CC	GO:0030425	dendrite	296	0.325201792	1.351282481	0.001831611	0.02563034	0.01982	4144	tags=43%, list=33%, signal=29%
BP	GO:0097435	supramolecular fiber organization	417	0.310646956	1.311650573	0.001831611	0.02563034	0.01982	4865	tags=47%, list=39%, signal=30%
BP	GO:0016055	Wnt signaling pathway	310	0.32367569	1.34832945	0.001868427	0.02602983	0.020129	5393	tags=52%, list=43%, signal=31%
BP	GO:0198738	cell-cell signaling by wnt	310	0.32367569	1.34832945	0.001868427	0.02602983	0.020129	5393	tags=52%, list=43%, signal=31%
CC	GO:0016363	nuclear matrix	80	0.422699625	1.607948278	0.00188198	0.026160774	0.02023	2924	tags=41%, list=23%, signal=32%
BP	GO:0002764	immune response-regulating signaling pathway	256	0.337043345	1.389654982	0.00190733	0.026454744	0.020457	4308	tags=41%, list=34%, signal=28%
CC	GO:0000307	cyclin-dependent protein kinase holoenzyme complex	26	0.548156707	1.762230035	0.001918579	0.02655229	0.020533	2766	tags=46%, list=22%, signal=36%
BP	GO:0006898	receptor-mediated endocytosis	145	0.367846594	1.471267504	0.00194826	0.026813598	0.020735	4061	tags=43%, list=32%, signal=29%
BP	GO:0072331	signal transduction by p53 class mediator	196	0.350995125	1.428269253	0.00194826	0.026813598	0.020735	4515	tags=48%, list=36%, signal=31%
BP	GO:0044774	mitotic DNA integrity checkpoint	74	0.425381799	1.602242146	0.001950235	0.026813598	0.020735	4416	tags=53%, list=35%, signal=34%
CC	GO:0018995	host cellular component	48	0.467589398	1.67411549	0.001966662	0.026921887	0.020819	3208	tags=54%, list=25%, signal=41%
CC	GO:0043657	host cell	48	0.467589398	1.67411549	0.001966662	0.026921887	0.020819	3208	tags=54%, list=25%, signal=41%
BP	GO:0070925	organelle assembly	488	0.305199811	1.294797068	0.001979419	0.027037745	0.020908	4239	tags=42%, list=34%, signal=29%
BP	GO:1905818	regulation of chromosome separation	40	0.481198854	1.661548482	0.001988549	0.027103667	0.020959	2775	tags=45%, list=22%, signal=35%
CC	GO:0005925	focal adhesion	335	0.319217852	1.331800395	0.002018433	0.027451556	0.021228	3796	tags=39%, list=30%, signal=28%
CC	GO:0032153	cell division site	38	0.483086506	1.659771717	0.002068687	0.028074402	0.02171	5193	tags=68%, list=41%, signal=40%
BP	GO:0038093	Fc receptor signaling pathway	143	0.363768438	1.452119366	0.002096695	0.02838927	0.021953	4209	tags=42%, list=33%, signal=28%
BP	GO:0002244	hematopoietic progenitor cell differentiation	117	0.381289369	1.50391874	0.002100905	0.02838927	0.021953	3660	tags=42%, list=29%, signal=30%
BP	GO:0019886	antigen processing and presentation of exogenous peptide antigen via MHC class II	66	0.422543265	1.574849336	0.002146155	0.028938631	0.022378	3684	tags=45%, list=29%, signal=32%
BP	GO:0050770	regulation of axonogenesis	109	0.387250806	1.51874996	0.002181843	0.029356983	0.022702	5193	tags=60%, list=41%, signal=35%

BP	GO:0003205	cardiac chamber development	90	0.393178541	1.514857523	0.002234894	0.029989813	0.023191	4052	tags=48%, list=32%, signal=33%
BP	GO:0009896	positive regulation of catabolic process	311	0.319709278	1.331689143	0.002238401	0.029989813	0.023191	5159	tags=51%, list=41%, signal=31%
BP	GO:0006700	C21-steroid hormone biosynthetic process	11	0.680091089	1.769876211	0.002262707	0.030251099	0.023393	2114	tags=45%, list=17%, signal=38%
CC	GO:0015629	actin cytoskeleton	302	0.323994855	1.346407951	0.002275399	0.030352792	0.023472	4606	tags=46%, list=37%, signal=30%
BP	GO:0032956	regulation of actin cytoskeleton organization	222	0.335236681	1.372015526	0.002279954	0.030352792	0.023472	3445	tags=38%, list=27%, signal=28%
BP	GO:0002831	regulation of response to biotic stimulus	253	0.33372332	1.375389807	0.002317026	0.030747221	0.023777	4508	tags=45%, list=36%, signal=29%
BP	GO:0007178	transmembrane receptor protein serine/threonine kinase signaling pathway	192	0.349621494	1.419011616	0.002319347	0.030747221	0.023777	5191	tags=54%, list=41%, signal=32%
BP	GO:0090501	RNA phosphodiester bond hydrolysis	107	0.388181602	1.520342916	0.00233573	0.030899355	0.023894	3987	tags=48%, list=32%, signal=33%
CC	GO:0030055	cell-substrate junction	337	0.318593839	1.330486413	0.002349394	0.031014953	0.023984	3796	tags=39%, list=30%, signal=28%
BP	GO:1904837	beta-catenin-TCF complex assembly	24	0.556191723	1.764698926	0.002364409	0.031102957	0.024052	4973	tags=79%, list=39%, signal=48%
BP	GO:0070507	regulation of microtubule cytoskeleton organization	115	0.38435178	1.512988028	0.002365939	0.031102957	0.024052	3523	tags=42%, list=28%, signal=30%
BP	GO:0009063	cellular amino acid catabolic process	74	-0.273515932	-1.638973534	0.002376032	0.031122801	0.024067	1897	tags=34%, list=15%, signal=29%
BP	GO:0007369	gastrulation	95	0.39795467	1.538184859	0.002380307	0.031122801	0.024067	3847	tags=46%, list=31%, signal=32%
MF	GO:0045309	protein phosphorylated amino acid binding	39	0.482562233	1.665801216	0.002382276	0.031122801	0.024067	4069	tags=56%, list=32%, signal=38%
CC	GO:0098978	glutamatergic synapse	165	0.357397785	1.436835036	0.002393565	0.031205544	0.024131	4201	tags=44%, list=33%, signal=29%
CC	GO:0030118	clathrin coat	30	0.515030817	1.698012561	0.002416234	0.031435997	0.024309	2384	tags=47%, list=19%, signal=38%
BP	GO:0046596	regulation of viral entry into host cell	22	0.563280876	1.758158893	0.00242544	0.031490708	0.024352	3818	tags=64%, list=30%, signal=44%
BP	GO:0034605	cellular response to heat	87	0.40111727	1.541921217	0.002460256	0.031877019	0.024651	4006	tags=47%, list=32%, signal=32%
CC	GO:0099524	postsynaptic cytosol	10	0.700196005	1.790073518	0.002533757	0.032761951	0.025335	2137	tags=60%, list=17%, signal=50%
BP	GO:0050779	RNA destabilization	24	0.552644621	1.753444595	0.002573643	0.033209488	0.025681	3513	tags=54%, list=28%, signal=39%
BP	GO:0050768	negative regulation of neurogenesis	165	0.35586177	1.430659843	0.002616218	0.033689823	0.026052	3614	tags=42%, list=29%, signal=31%
BP	GO:0051489	regulation of filopodium assembly	25	0.537420068	1.715892306	0.002633531	0.03384356	0.026171	1646	tags=40%, list=13%, signal=35%
BP	GO:0060996	dendritic spine development	63	0.431423132	1.603036123	0.002648097	0.033926323	0.026235	2805	tags=40%, list=22%, signal=31%
BP	GO:0031023	microtubule organizing center organization	84	0.398493212	1.527047621	0.002650747	0.033926323	0.026235	3020	tags=40%, list=24%, signal=31%
CC	GO:0043596	nuclear replication fork	30	0.513271841	1.692213368	0.002659882	0.033974191	0.026272	4160	tags=60%, list=33%, signal=40%
BP	GO:0009083	branched-chain amino acid catabolic process	20	-0.469181747	-1.906678881	0.002678147	0.034138248	0.026399	475	tags=35%, list=4%, signal=34%
BP	GO:0002253	activation of immune response	274	0.331047611	1.370042725	0.002687742	0.034156597	0.026413	4209	tags=42%, list=33%, signal=29%
CC	GO:0030027	lamellipodium	143	0.360883316	1.440602312	0.002690435	0.034156597	0.026413	4344	tags=46%, list=34%, signal=31%
BP	GO:0032606	type I interferon production	97	0.396696914	1.537207807	0.002714919	0.034363748	0.026574	4900	tags=55%, list=39%, signal=34%
BP	GO:0052126	movement in host environment	104	0.387915639	1.512309245	0.002717667	0.034363748	0.026574	3820	tags=48%, list=30%, signal=34%
CC	GO:0098982	GABA-ergic synapse	21	0.560277858	1.726738239	0.002733155	0.034490338	0.026671	2420	tags=48%, list=19%, signal=39%
BP	GO:0097711	ciliary basal body-plasma membrane docking	53	0.456104121	1.651737989	0.00275262	0.03466649	0.026808	3127	tags=45%, list=25%, signal=34%
BP	GO:0032481	positive regulation of type I interferon production	59	0.449768919	1.659833597	0.002809174	0.035308117	0.027304	4858	tags=59%, list=39%, signal=37%
MF	GO:0003779	actin binding	242	0.335496467	1.380794926	0.002833193	0.035468426	0.027428	4507	tags=48%, list=36%, signal=31%
BP	GO:0043254	regulation of protein-containing complex assembly	273	0.32568781	1.348516903	0.002833193	0.035468426	0.027428	4858	tags=47%, list=39%, signal=30%
BP	GO:0010803	regulation of tumor necrosis factor-mediated signaling pathway	39	0.477824947	1.649448139	0.002862724	0.035767014	0.027659	4340	tags=62%, list=34%, signal=40%
MF	GO:0008017	microtubule binding	130	0.367850258	1.463359716	0.002878865	0.035826502	0.027705	3207	tags=41%, list=25%, signal=31%
BP	GO:0045732	positive regulation of protein catabolic process	153	0.355764522	1.424664016	0.002878865	0.035826502	0.027705	5040	tags=55%, list=40%, signal=33%
BP	GO:0010965	regulation of mitotic sister chromatid separation	36	0.481995087	1.637143111	0.002894011	0.035943958	0.027795	2775	tags=44%, list=22%, signal=35%

MF	GO:0031267	small GTPase binding	274	0.329773426	1.364769503	0.002910173	0.03607354	0.027896	4074	tags=43%, list=32%, signal=30%
CC	GO:0030426	growth cone	110	0.38063678	1.493535142	0.002924812	0.036183768	0.027981	5328	tags=63%, list=42%, signal=37%
BP	GO:0051304	chromosome separation	53	0.45487645	1.647292094	0.002945107	0.036363414	0.02812	2885	tags=45%, list=23%, signal=35%
BP	GO:0032886	regulation of microtubule-based process	131	0.362165127	1.44132172	0.002953157	0.036391445	0.028142	3523	tags=40%, list=28%, signal=29%
BP	GO:0032479	regulation of type I interferon production	95	0.395028539	1.526874702	0.002980058	0.036518639	0.02824	4900	tags=55%, list=39%, signal=34%
BP	GO:0032366	intracellular sterol transport	15	-0.55132489	-2.07036187	0.002980877	0.036518639	0.02824	1863	tags=47%, list=15%, signal=40%
BP	GO:0032367	intracellular cholesterol transport	15	-0.55132489	-2.07036187	0.002980877	0.036518639	0.02824	1863	tags=47%, list=15%, signal=40%
BP	GO:0045765	regulation of angiogenesis	189	0.350867722	1.422342174	0.002990303	0.036562986	0.028274	4634	tags=48%, list=37%, signal=31%
BP	GO:0010833	telomere maintenance via telomere lengthening	57	0.441851876	1.616706992	0.003018964	0.036841896	0.02849	3586	tags=42%, list=28%, signal=30%
BP	GO:0007608	sensory perception of smell	25	-0.415868931	-1.845060389	0.003034021	0.036954029	0.028577	1730	tags=44%, list=14%, signal=38%
BP	GO:1900034	regulation of cellular response to heat	60	0.436677482	1.609751715	0.003086265	0.037517782	0.029013	3221	tags=43%, list=26%, signal=32%
CC	GO:0015030	Cajal body	43	0.470670403	1.655092722	0.003102735	0.037645322	0.029111	3248	tags=49%, list=26%, signal=36%
BP	GO:0040029	regulation of gene expression, epigenetic	104	0.386157775	1.505456119	0.003129996	0.037843469	0.029264	6132	tags=73%, list=49%, signal=38%
BP	GO:0006278	RNA-dependent DNA biosynthetic process	52	0.452197482	1.638469642	0.003131086	0.037843469	0.029264	3586	tags=42%, list=28%, signal=30%
MF	GO:0008106	alcohol dehydrogenase (NADP+) activity	13	-0.581393021	-2.026747119	0.003158941	0.03810699	0.029468	1978	tags=62%, list=16%, signal=52%
BP	GO:0033047	regulation of mitotic sister chromatid segregation	42	0.47599009	1.668228119	0.003192004	0.03843222	0.02972	2775	tags=43%, list=22%, signal=34%
BP	GO:0051225	spindle assembly	67	0.425308406	1.588322364	0.003203143	0.038492734	0.029766	3141	tags=43%, list=25%, signal=33%
MF	GO:0033613	activating transcription factor binding	53	0.452777665	1.639691542	0.003214591	0.038556718	0.029816	4042	tags=51%, list=32%, signal=35%
BP	GO:0043331	response to dsRNA	32	0.505509409	1.691111256	0.003230046	0.038668435	0.029902	3493	tags=53%, list=28%, signal=39%
CC	GO:0030119	AP-type membrane coat adaptor complex	26	0.535240758	1.72070747	0.003278728	0.039167557	0.030288	3532	tags=54%, list=28%, signal=39%
MF	GO:0045296	cadherin binding	233	0.330234684	1.355796075	0.003284178	0.039167557	0.030288	4952	tags=50%, list=39%, signal=31%
MF	GO:0051219	phosphoprotein binding	59	0.447199937	1.650352989	0.003306027	0.03928745	0.030381	4969	tags=61%, list=39%, signal=37%
BP	GO:0044773	mitotic DNA damage checkpoint	70	0.422969139	1.589371939	0.003306709	0.03928745	0.030381	4416	tags=53%, list=35%, signal=35%
BP	GO:0031497	chromatin assembly	67	0.424517008	1.585366873	0.003316863	0.0393063	0.030396	4501	tags=54%, list=36%, signal=35%
BP	GO:0051783	regulation of nuclear division	101	0.389515878	1.51385408	0.00332078	0.0393063	0.030396	5448	tags=58%, list=43%, signal=33%
BP	GO:0051495	positive regulation of cytoskeleton organization	141	0.361049098	1.439610424	0.003327956	0.039317339	0.030404	3423	tags=38%, list=27%, signal=28%
BP	GO:0006081	cellular aldehyde metabolic process	38	-0.353793258	-1.719891991	0.003335607	0.039333926	0.030417	2220	tags=42%, list=18%, signal=35%
BP	GO:0045931	positive regulation of mitotic cell cycle	96	0.393053785	1.520143933	0.003354903	0.039487527	0.030536	4864	tags=54%, list=39%, signal=34%
BP	GO:0050808	synapse organization	199	0.345920085	1.408131766	0.003395505	0.03989085	0.030848	4113	tags=44%, list=33%, signal=30%
BP	GO:0006695	cholesterol biosynthetic process	51	-0.322696257	-1.759697377	0.003457096	0.040463445	0.03129	2612	tags=47%, list=21%, signal=37%
BP	GO:1902653	secondary alcohol biosynthetic process	51	-0.322696257	-1.759697377	0.003457096	0.040463445	0.03129	2612	tags=47%, list=21%, signal=37%
BP	GO:0051701	interaction with host	134	0.359371414	1.430779504	0.003510349	0.040909823	0.031636	3820	tags=44%, list=30%, signal=31%
MF	GO:0001098	basal transcription machinery binding	40	0.47270755	1.632228557	0.003514724	0.040909823	0.031636	4256	tags=55%, list=34%, signal=37%
MF	GO:0001099	basal RNA polymerase II transcription machinery binding	40	0.47270755	1.632228557	0.003514724	0.040909823	0.031636	4256	tags=55%, list=34%, signal=37%
MF	GO:0031435	mitogen-activated protein kinase kinase kinase binding	14	0.620244512	1.713313228	0.003565501	0.041424275	0.032033	3817	tags=71%, list=30%, signal=50%
BP	GO:0001885	endothelial cell development	43	0.465277288	1.636128058	0.003577032	0.041481718	0.032078	2063	tags=44%, list=16%, signal=37%
BP	GO:0060271	cilium assembly	171	0.347816414	1.398996542	0.003584642	0.041493548	0.032087	5287	tags=52%, list=42%, signal=31%
MF	GO:0097718	disordered domain specific binding	27	0.52784092	1.706695781	0.00362969	0.041937902	0.032431	2874	tags=48%, list=23%, signal=37%
BP	GO:0060840	artery development	54	0.44034965	1.601296442	0.003638065	0.041957682	0.032446	3030	tags=48%, list=24%, signal=37%

BP	GO:0051031	tRNA transport	29	0.517500193	1.692913143	0.003671062	0.042253223	0.032674	4001	tags=66%, list=32%, signal=45%
BP	GO:0030902	hindbrain development	78	0.404655251	1.534217389	0.003677111	0.042253223	0.032674	3954	tags=45%, list=31%, signal=31%
BP	GO:0032210	regulation of telomere maintenance via telomerase	39	0.471559783	1.62782084	0.003703512	0.042479084	0.032849	3586	tags=46%, list=28%, signal=33%
BP	GO:0031424	keratinization	24	-0.41469719	-1.810340272	0.003723259	0.042627925	0.032964	3020	tags=71%, list=24%, signal=54%
BP	GO:0031124	mRNA 3'-end processing	63	0.425171529	1.579807081	0.003753043	0.042890944	0.033168	4278	tags=48%, list=34%, signal=32%
BP	GO:0099173	postsynapse organization	89	0.402716586	1.550741922	0.003774875	0.043062301	0.0333	2805	tags=39%, list=22%, signal=31%
BP	GO:0007063	regulation of sister chromatid cohesion	14	0.617629493	1.706089711	0.00379553	0.043158029	0.033374	1603	tags=43%, list=13%, signal=37%
BP	GO:0043507	positive regulation of JUN kinase activity	48	0.452105711	1.618679074	0.003796974	0.043158029	0.033374	3945	tags=50%, list=31%, signal=34%
BP	GO:2001258	negative regulation of cation channel activity	22	0.548073854	1.710693477	0.003859583	0.043790623	0.033863	3309	tags=55%, list=26%, signal=40%
BP	GO:1902600	proton transmembrane transport	77	-0.267312406	-1.61742949	0.003898407	0.044151565	0.034142	1486	tags=32%, list=12%, signal=29%
BP	GO:0042176	regulation of protein catabolic process	271	0.321287899	1.330216532	0.003907931	0.044179965	0.034164	4403	tags=46%, list=35%, signal=31%
BP	GO:0007179	transforming growth factor beta receptor signaling pathway	126	0.367090158	1.45659842	0.003915762	0.044189168	0.034172	5191	tags=56%, list=41%, signal=33%
BP	GO:0006577	amino-acid betaine metabolic process	10	-0.602983426	-1.897289802	0.003938077	0.044319626	0.034272	2755	tags=60%, list=22%, signal=47%
BP	GO:0070831	basement membrane assembly	11	0.661709963	1.722040976	0.003941399	0.044319626	0.034272	3469	tags=82%, list=28%, signal=59%
CC	GO:0000812	Swr1 complex	11	0.659977876	1.717533374	0.003988042	0.044764176	0.034616	2315	tags=55%, list=18%, signal=45%
BP	GO:0034728	nucleosome organization	65	0.429354794	1.601780115	0.004004027	0.044798356	0.034643	4825	tags=58%, list=38%, signal=36%
BP	GO:0007091	metaphase/anaphase transition of mitotic cell cycle	34	0.493846795	1.66277718	0.004009785	0.044798356	0.034643	2775	tags=44%, list=22%, signal=34%
BP	GO:0021872	forebrain generation of neurons	26	0.530732756	1.706215014	0.004021072	0.044798356	0.034643	3610	tags=54%, list=29%, signal=39%
BP	GO:0071622	regulation of granulocyte chemotaxis	26	0.530755444	1.706287951	0.004021072	0.044798356	0.034643	1256	tags=31%, list=10%, signal=28%
BP	GO:0000077	DNA damage checkpoint	97	0.390515961	1.5132565	0.004026659	0.044798356	0.034643	4416	tags=47%, list=35%, signal=31%
BP	GO:0030073	insulin secretion	102	-0.22388116	-1.419911003	0.004040788	0.044876263	0.034703	1427	tags=25%, list=11%, signal=23%
CC	GO:0045239	tricarboxylic acid cycle enzyme complex	12	-0.557957008	-1.843027188	0.004051522	0.044911402	0.03473	1713	tags=58%, list=14%, signal=50%
BP	GO:0045071	negative regulation of viral genome replication	39	0.468134794	1.615997804	0.004063851	0.044911402	0.03473	4084	tags=56%, list=32%, signal=38%
CC	GO:1990752	microtubule end	20	0.564191031	1.725338742	0.004065348	0.044911402	0.03473	3127	tags=50%, list=25%, signal=38%
BP	GO:0001886	endothelial cell morphogenesis	10	0.686667501	1.755487463	0.004096599	0.045156988	0.03492	1956	tags=70%, list=16%, signal=59%
MF	GO:0008168	methyltransferase activity	102	0.378251776	1.473171153	0.004101921	0.045156988	0.03492	4466	tags=50%, list=35%, signal=33%
BP	GO:0140058	neuron projection arborization	11	0.65925851	1.715661288	0.004127972	0.045321227	0.035047	3844	tags=82%, list=30%, signal=57%
BP	GO:0071900	regulation of protein serine/threonine kinase activity	319	0.309194169	1.288178968	0.004131235	0.045321227	0.035047	4097	tags=40%, list=32%, signal=28%
BP	GO:0006953	acute-phase response	14	-0.524187292	-1.925550735	0.004165164	0.045613977	0.035273	1659	tags=50%, list=13%, signal=43%
BP	GO:0060674	placenta blood vessel development	21	0.551861319	1.70079904	0.004173864	0.045629903	0.035286	703	tags=29%, list=6%, signal=27%
BP	GO:0071156	regulation of cell cycle arrest	69	0.417230137	1.568720254	0.004198409	0.045818687	0.035432	3332	tags=45%, list=26%, signal=33%
CC	GO:0090734	site of DNA damage	49	0.451871879	1.621146637	0.004247958	0.046279225	0.035788	5360	tags=67%, list=43%, signal=39%
MF	GO:0050839	cell adhesion molecule binding	337	0.311873679	1.302422212	0.004275828	0.046441859	0.035914	4554	tags=44%, list=36%, signal=29%
MF	GO:0048156	tau protein binding	31	0.50737614	1.685018357	0.004280319	0.046441859	0.035914	3007	tags=48%, list=24%, signal=37%
BP	GO:0072583	clathrin-dependent endocytosis	30	0.502619142	1.65709233	0.004285012	0.046441859	0.035914	3831	tags=53%, list=30%, signal=37%
BP	GO:0007257	activation of JUN kinase activity	25	0.526003872	1.679442303	0.004376182	0.047317404	0.036591	2993	tags=48%, list=24%, signal=37%
BP	GO:2000045	regulation of G1/S transition of mitotic cell cycle	113	0.371803808	1.46160115	0.004380824	0.047317404	0.036591	4973	tags=53%, list=39%, signal=32%
BP	GO:0051170	import into nucleus	114	0.375162468	1.476566944	0.00444676	0.047947347	0.037078	3234	tags=39%, list=26%, signal=29%
BP	GO:0060445	branching involved in salivary gland morphogenesis	12	0.6454709	1.724262809	0.004491197	0.04834371	0.037384	1474	tags=50%, list=12%, signal=44%

BP	GO:0010720	positive regulation of cell development	304	0.312045601	1.297035041	0.004498909	0.048344082	0.037385	5434	tags=52%, list=43%, signal=30%
BP	GO:0048598	embryonic morphogenesis	310	0.315784848	1.315458726	0.00457327	0.049059421	0.037938	3866	tags=39%, list=31%, signal=28%
CC	GO:0010494	cytoplasmic stress granule	53	0.445421876	1.613053248	0.004603923	0.049195846	0.038043	3069	tags=43%, list=24%, signal=33%
BP	GO:0002495	antigen processing and presentation of peptide antigen via MHC class II	67	0.421276015	1.573263322	0.004609425	0.049195846	0.038043	3684	tags=45%, list=29%, signal=32%
BP	GO:0002504	antigen processing and presentation of peptide or polysaccharide antigen via MHC class II	67	0.421276015	1.573263322	0.004609425	0.049195846	0.038043	3684	tags=45%, list=29%, signal=32%
BP	GO:0048844	artery morphogenesis	37	0.472935677	1.618494416	0.004627139	0.049301348	0.038125	2892	tags=51%, list=23%, signal=40%
BP	GO:0042590	antigen processing and presentation of exogenous peptide antigen via MHC class I	71	0.413376078	1.55751757	0.004657012	0.049535821	0.038306	3008	tags=42%, list=24%, signal=32%
BP	GO:1903510	mucopolysaccharide metabolic process	68	0.416426362	1.559194757	0.004666507	0.049553107	0.038319	3467	tags=43%, list=28%, signal=31%
**KEGG**										
	**ID**	**Description**	**setSize**	**enrichmentScore**	**NES**	**pvalue**	**p.adjust**	**qvalues**	**rank**	**leading_edge**
	hsa00280	Valine, leucine and isoleucine degradation	38	-0.590034812	-2.886860391	3.12263E-09	9.74259E-07	6.54E-07	1755	tags=42%, list=14%, signal=36%
	hsa03040	Spliceosome	102	0.507583111	1.994048688	1.89397E-08	2.9546E-06	1.98E-06	4048	tags=61%, list=32%, signal=42%
	hsa05200	Pathways in cancer	316	0.378050843	1.591072718	7.45E-07	7.748E-05	5.2E-05	4186	tags=47%, list=33%, signal=32%
	hsa05166	Human T-cell leukemia virus 1 infection	153	0.428177434	1.730681754	1.50487E-06	0.00011738	7.88E-05	3895	tags=46%, list=31%, signal=32%
	hsa00640	Propanoate metabolism	28	-0.564994367	-2.589870841	2.50573E-06	0.000156358	0.000105	1992	tags=46%, list=16%, signal=39%
	hsa05222	Small cell lung cancer	70	0.513753315	1.952777805	3.22213E-06	0.000158288	0.000106	4319	tags=63%, list=34%, signal=42%
	hsa05135	Yersinia infection	99	0.464477255	1.820094433	3.55133E-06	0.000158288	0.000106	3766	tags=52%, list=30%, signal=36%
	hsa05132	Salmonella infection	187	0.399270209	1.642300392	1.20423E-05	0.00046965	0.000315	4613	tags=53%, list=37%, signal=34%
	hsa01200	Carbon metabolism	84	-0.315950542	-1.871257133	1.51557E-05	0.000521531	0.00035	1826	tags=36%, list=14%, signal=31%
	hsa05169	Epstein-Barr virus infection	144	0.419468021	1.692497985	1.67157E-05	0.000521531	0.00035	4468	tags=53%, list=35%, signal=34%
	hsa00520	Amino sugar and nucleotide sugar metabolism	36	-0.457267951	-2.245070179	2.86283E-05	0.000812002	0.000545	2263	tags=44%, list=18%, signal=37%
	hsa04110	Cell cycle	83	0.457253428	1.762331247	3.54018E-05	0.000920448	0.000618	3020	tags=46%, list=24%, signal=35%
	hsa00330	Arginine and proline metabolism	30	-0.474939719	-2.169254685	4.33326E-05	0.001039982	0.000698	2134	tags=50%, list=17%, signal=42%
	hsa05203	Viral carcinogenesis	127	0.416279517	1.668388694	4.93568E-05	0.001099951	0.000738	4350	tags=52%, list=35%, signal=34%
	hsa05165	Human papillomavirus infection	216	0.372387422	1.53763596	7.50915E-05	0.001561903	0.001049	4350	tags=50%, list=35%, signal=34%
	hsa05100	Bacterial invasion of epithelial cells	53	0.499330513	1.83691752	9.51832E-05	0.001856073	0.001246	3809	tags=60%, list=30%, signal=42%
	hsa00620	Pyruvate metabolism	32	-0.462612996	-2.183493775	0.000102588	0.001882786	0.001264	1992	tags=53%, list=16%, signal=45%
	hsa05220	Chronic myeloid leukemia	56	0.487236532	1.80335952	0.000108689	0.001883942	0.001265	4350	tags=59%, list=35%, signal=39%
	hsa04666	Fc gamma R-mediated phagocytosis	73	0.456151439	1.737686903	0.000130124	0.002136775	0.001435	4209	tags=53%, list=33%, signal=36%
	hsa04072	Phospholipase D signaling pathway	89	0.434949064	1.68616917	0.000184676	0.002880941	0.001934	4061	tags=52%, list=32%, signal=35%
	hsa00650	Butanoate metabolism	12	-0.713846858	-2.498869981	0.000197883	0.002939981	0.001974	1562	tags=75%, list=12%, signal=66%
	hsa05160	Hepatitis C	106	0.419627065	1.654455259	0.000223024	0.003149827	0.002115	4578	tags=58%, list=36%, signal=38%
	hsa00380	Tryptophan metabolism	24	-0.496320089	-2.205819225	0.000242236	0.003149827	0.002115	1713	tags=54%, list=14%, signal=47%
	hsa04115	p53 signaling pathway	47	0.504939729	1.816611556	0.000242294	0.003149827	0.002115	4176	tags=60%, list=33%, signal=40%
	hsa00071	Fatty acid degradation	30	-0.432494926	-1.975390998	0.000264604	0.003302252	0.002217	1562	tags=33%, list=12%, signal=29%
	hsa04144	Endocytosis	175	0.375668249	1.537396542	0.000275971	0.003311656	0.002223	3870	tags=49%, list=31%, signal=35%
	hsa03013	Nucleocytoplasmic transport	83	0.431166925	1.661789497	0.000306125	0.003537449	0.002375	5420	tags=69%, list=43%, signal=39%
	hsa05167	Kaposi sarcoma-associated herpesvirus infection	128	0.391717121	1.569836703	0.000327457	0.003648804	0.00245	4308	tags=50%, list=34%, signal=33%
	hsa05206	MicroRNAs in cancer	116	0.405604883	1.612488338	0.000384731	0.004139179	0.002779	4061	tags=51%, list=32%, signal=35%

hsa05164	Influenza A	107	0.405536158	1.599833568	0.000466357	0.004850108	0.003256	4186	tags=49%, list=33%, signal=33%
hsa04151	PI3K-Akt signaling pathway	200	0.36067199	1.48702709	0.000582172	0.005859278	0.003934	4156	tags=45%, list=33%, signal=31%
hsa00630	Glyoxylate and dicarboxylate metabolism	23	-0.478335355	-2.067952559	0.000680082	0.006483565	0.004353	1806	tags=43%, list=14%, signal=37%
hsa05170	Human immunodeficiency virus 1 infection	128	0.384253643	1.539926235	0.000685762	0.006483565	0.004353	4802	tags=52%, list=38%, signal=33%
hsa04010	MAPK signaling pathway	169	0.370044494	1.507987697	0.000730319	0.006701749	0.004499	3992	tags=45%, list=32%, signal=31%
hsa05163	Human cytomegalovirus infection	146	0.38223887	1.540636391	0.000770151	0.006865343	0.004609	4186	tags=47%, list=33%, signal=32%
hsa05224	Breast cancer	78	0.425090413	1.628736797	0.00081504	0.007063677	0.004742	4176	tags=49%, list=33%, signal=33%
hsa00830	Retinol metabolism	19	-0.510600397	-2.068533322	0.000869328	0.007330552	0.004922	846	tags=37%, list=7%, signal=34%
hsa04914	Progesterone-mediated oocyte maturation	53	0.464060169	1.707166359	0.000897601	0.007369777	0.004948	3221	tags=47%, list=26%, signal=35%
hsa05168	Herpes simplex virus 1 infection	265	0.338321628	1.414497857	0.000929623	0.007436982	0.004993	5374	tags=53%, list=43%, signal=31%
hsa00020	Citrate cycle (TCA cycle)	26	-0.431314291	-1.957242519	0.00124033	0.009667889	0.006491	1826	tags=42%, list=14%, signal=36%
hsa04330	Notch signaling pathway	44	0.487960694	1.731830999	0.00127046	0.009667889	0.006491	4350	tags=61%, list=35%, signal=40%
hsa04650	Natural killer cell mediated cytotoxicity	58	0.446264586	1.658264307	0.0013724	0.010194971	0.006845	4071	tags=53%, list=32%, signal=36%
hsa03030	DNA replication	33	0.506142177	1.714869617	0.001426115	0.010347625	0.006947	3893	tags=61%, list=31%, signal=42%
hsa01522	Endocrine resistance	61	0.449462395	1.674922705	0.001517826	0.010762768	0.007226	4416	tags=56%, list=35%, signal=36%
hsa04621	NOD-like receptor signaling pathway	108	0.393622245	1.553721351	0.001677699	0.01145427	0.00769	4186	tags=48%, list=33%, signal=32%
hsa03050	Proteasome	41	0.483597106	1.700463788	0.001688771	0.01145427	0.00769	3008	tags=46%, list=24%, signal=35%
hsa04510	Focal adhesion	136	0.373836975	1.504194806	0.001731542	0.011494489	0.007717	4156	tags=47%, list=33%, signal=32%
hsa05130	Pathogenic Escherichia coli infection	133	0.366607163	1.472032938	0.001859306	0.012085489	0.008114	4850	tags=54%, list=38%, signal=34%
hsa05131	Shigellosis	167	0.360895174	1.469675689	0.001981398	0.01261625	0.00847	4061	tags=45%, list=32%, signal=31%
hsa00500	Starch and sucrose metabolism	20	-0.457635375	-1.883988554	0.002367906	0.014775731	0.00992	1735	tags=45%, list=14%, signal=39%
hsa05225	Hepatocellular carcinoma	103	0.384388517	1.51155218	0.002470277	0.015112285	0.010146	4346	tags=50%, list=34%, signal=33%
hsa05212	Pancreatic cancer	56	0.435885919	1.613300666	0.002716946	0.016301677	0.010945	4316	tags=54%, list=34%, signal=35%
hsa04015	Rap1 signaling pathway	126	0.367766581	1.472212485	0.002813037	0.016559764	0.011118	3853	tags=41%, list=31%, signal=29%
hsa04071	Sphingolipid signaling pathway	78	0.408027919	1.563361734	0.00287375	0.016603888	0.011148	5245	tags=59%, list=42%, signal=35%
hsa00410	beta-Alanine metabolism	19	-0.470206903	-1.904892071	0.003082835	0.017386357	0.011673	1562	tags=37%, list=12%, signal=32%
hsa04146	Peroxisome	55	-0.311297861	-1.660796606	0.003120628	0.017386357	0.011673	1826	tags=33%, list=14%, signal=28%
hsa00220	Arginine biosynthesis	10	-0.628145128	-2.015023672	0.00343801	0.018818582	0.012635	1822	tags=50%, list=14%, signal=43%
hsa04014	Ras signaling pathway	123	0.376968311	1.504489655	0.003561786	0.019159951	0.012864	4061	tags=45%, list=32%, signal=31%
hsa05162	Measles	87	0.397498514	1.538440197	0.003847556	0.020079563	0.013481	4698	tags=53%, list=37%, signal=33%
hsa04740	Olfactory transduction	32	-0.371063111	-1.751386145	0.003861454	0.020079563	0.013481	1877	tags=44%, list=15%, signal=37%
hsa04919	Thyroid hormone signaling pathway	84	0.394784618	1.522673475	0.004008134	0.020500621	0.013764	5383	tags=56%, list=43%, signal=32%
hsa01230	Biosynthesis of amino acids	48	-0.293661661	-1.565703268	0.004457833	0.022387527	0.015031	1826	tags=38%, list=14%, signal=32%
hsa00053	Ascorbate and aldarate metabolism	11	-0.571149085	-1.923276318	0.00452216	0.022387527	0.015031	1682	tags=55%, list=13%, signal=47%
hsa05323	Rheumatoid arthritis	46	-0.311097239	-1.635108066	0.004592313	0.022387527	0.015031	3195	tags=52%, list=25%, signal=39%
hsa04216	Ferroptosis	31	-0.364663832	-1.685594825	0.004716282	0.022479336	0.015092	2036	tags=42%, list=16%, signal=35%
hsa04810	Regulation of actin cytoskeleton	142	0.356632429	1.43739586	0.004755244	0.022479336	0.015092	3858	tags=43%, list=31%, signal=30%
hsa04210	Apoptosis	96	0.379618531	1.486278657	0.007126851	0.033187724	0.022282	5344	tags=55%, list=42%, signal=32%
hsa00534	Glycosaminoglycan biosynthesis - heparan sulfate / heparin	14	0.61319864	1.730157085	0.007316372	0.033569236	0.022538	1213	tags=43%, list=10%, signal=39%

	hsa05161	Hepatitis B	101	0.367573576	1.443694468	0.009664403	0.043458319	0.029177	4789	tags=50%, list=38%, signal=31%
	hsa03018	RNA degradation	63	0.401380582	1.504055966	0.009750264	0.043458319	0.029177	3987	tags=48%, list=32%, signal=33%
	hsa00010	Glycolysis / Gluconeogenesis	39	-0.313133536	-1.571838849	0.010314984	0.044525051	0.029894	1904	tags=38%, list=15%, signal=33%
	hsa04114	Oocyte meiosis	79	0.386113574	1.481883892	0.010357465	0.044525051	0.029894	3221	tags=42%, list=26%, signal=31%
	hsa04540	Gap junction	53	0.416530197	1.532314963	0.01041772	0.044525051	0.029894	3858	tags=53%, list=31%, signal=37%
	hsa05214	Glioma	49	0.427539073	1.550383798	0.010937175	0.046113495	0.03096	4176	tags=55%, list=33%, signal=37%
**STEAP4**										
**GO**										
**ONTOLOGY**	**ID**	**Description**	**setSize**	**enrichmentScore**	**NES**	**pvalue**	**p.adjust**	**qvalues**	**rank**	**leading_edge**
BP	GO:0000904	cell morphogenesis involved in differentiation	425	0.390589478	1.630084429	1.79E-10	5.31E-07	4.33E-07	4187	tags=50%, list=33%, signal=35%
BP	GO:0001568	blood vessel development	412	0.389952965	1.628330815	3.17E-10	5.31E-07	4.33E-07	3703	tags=43%, list=29%, signal=31%
BP	GO:0006614	SRP-dependent cotranslational protein targeting to membrane	90	-0.417965778	-2.505961635	3.17E-10	5.31E-07	4.33E-07	1704	tags=37%, list=14%, signal=32%
BP	GO:0001944	vasculature development	436	0.386494734	1.615255173	3.37E-10	5.31E-07	4.33E-07	3703	tags=42%, list=29%, signal=31%
BP	GO:0006613	cotranslational protein targeting to membrane	94	-0.394220296	-2.41731201	1.46E-09	1.85E-06	1.51E-06	1704	tags=35%, list=14%, signal=31%
CC	GO:0022626	cytosolic ribosome	98	-0.384068203	-2.403747507	2.94E-09	3.10E-06	2.53E-06	1853	tags=37%, list=15%, signal=32%
BP	GO:0045047	protein targeting to ER	102	-0.382221714	-2.401716117	4.34E-09	3.92E-06	3.19E-06	1704	tags=34%, list=14%, signal=30%
BP	GO:0001525	angiogenesis	309	0.405715908	1.680940307	5.49E-09	4.33E-06	3.53E-06	3121	tags=40%, list=25%, signal=31%
BP	GO:0030036	actin cytoskeleton organization	422	0.377716272	1.576855485	7.43E-09	5.21E-06	4.25E-06	3576	tags=43%, list=28%, signal=32%
BP	GO:0048514	blood vessel morphogenesis	358	0.393639856	1.637666217	9.49E-09	5.99E-06	4.88E-06	4212	tags=47%, list=33%, signal=32%
BP	GO:0051301	cell division	330	0.391481832	1.626656266	1.34E-08	7.67E-06	6.25E-06	5227	tags=59%, list=41%, signal=36%
BP	GO:0072599	establishment of protein localization to endoplasmic reticulum	105	-0.369003304	-2.294290603	1.82E-08	9.54E-06	7.78E-06	1704	tags=34%, list=14%, signal=30%
BP	GO:0030029	actin filament-based process	462	0.3675528	1.53939853	2.14E-08	1.04E-05	8.48E-06	3576	tags=42%, list=28%, signal=31%
BP	GO:0035239	tube morphogenesis	491	0.359696261	1.510476604	3.37E-08	1.52E-05	1.24E-05	4256	tags=45%, list=34%, signal=31%
CC	GO:0005925	focal adhesion	335	0.385584754	1.60190729	3.73E-08	1.56909E-05	1.28E-05	3579	tags=44%, list=28%, signal=33%
BP	GO:0032990	cell part morphogenesis	392	0.376037424	1.568016627	6.45E-08	2.54307E-05	2.07E-05	4187	tags=49%, list=33%, signal=34%
BP	GO:0048858	cell projection morphogenesis	377	0.376760384	1.570047453	7.08E-08	2.62698E-05	2.14E-05	4187	tags=49%, list=33%, signal=34%
CC	GO:0030055	cell-substrate junction	337	0.384155619	1.596401589	9.18E-08	3.21615E-05	2.62E-05	3579	tags=44%, list=28%, signal=32%
BP	GO:0001667	ameboidal-type cell migration	267	0.397839068	1.641311959	1.05E-07	3.49955E-05	2.85E-05	4419	tags=52%, list=35%, signal=34%
BP	GO:0070972	protein localization to endoplasmic reticulum	128	-0.314397482	-2.002938499	1.38E-07	4.36303E-05	3.56E-05	1704	tags=31%, list=14%, signal=27%
BP	GO:0007264	small GTPase mediated signal transduction	327	0.384190128	1.596126682	1.63E-07	4.7448E-05	3.87E-05	4358	tags=52%, list=35%, signal=35%
BP	GO:0007409	axonogenesis	262	0.400646701	1.652125727	1.65E-07	4.7448E-05	3.87E-05	4187	tags=52%, list=33%, signal=35%
BP	GO:0120039	plasma membrane bounded cell projection morphogenesis	374	0.372372723	1.550847779	1.87E-07	5.11665E-05	4.17E-05	4187	tags=49%, list=33%, signal=33%
BP	GO:0031589	cell-substrate adhesion	218	0.419134374	1.72026844	2.06E-07	5.39878E-05	4.4E-05	3554	tags=47%, list=28%, signal=34%
BP	GO:0048812	neuron projection morphogenesis	362	0.373288176	1.552653011	2.14E-07	5.39878E-05	4.4E-05	4187	tags=49%, list=33%, signal=34%
BP	GO:0006897	endocytosis	318	0.381227571	1.580915772	2.94E-07	7.14418E-05	5.83E-05	4779	tags=53%, list=38%, signal=34%
BP	GO:0000184	nuclear-transcribed mRNA catabolic process, nonsense-mediated decay	108	-0.330938208	-2.094755073	3.48E-07	8.12822E-05	6.63E-05	1704	tags=31%, list=14%, signal=27%
BP	GO:0097435	supramolecular fiber organization	417	0.362221482	1.512186146	4.73E-07	0.000106475	8.68E-05	4060	tags=46%, list=32%, signal=32%
BP	GO:0000377	RNA splicing, via transesterification reactions with bulged adenosine as nucleophile	247	0.396337946	1.629277375	5.92E-07	0.000124568	0.000102	4979	tags=55%, list=39%, signal=34%
BP	GO:0000398	mRNA splicing, via spliceosome	247	0.396337946	1.629277375	5.92E-07	0.000124568	0.000102	4979	tags=55%, list=39%, signal=34%

BP	GO:0048667	cell morphogenesis involved in neuron differentiation	331	0.377617015	1.569275855	6.26E-07	0.000127361	0.000104	4187	tags=50%, list=33%, signal=34%
MF	GO:0003779	actin binding	242	0.399941632	1.644670285	7.03E-07	0.000138627	0.000113	3666	tags=45%, list=29%, signal=33%
BP	GO:0003158	endothelium development	80	0.517169255	1.96377434	7.88E-07	0.000150682	0.000123	3496	tags=59%, list=28%, signal=43%
BP	GO:0022604	regulation of cell morphogenesis	296	0.379470655	1.570537805	8.64E-07	0.00016036	0.000131	4256	tags=51%, list=34%, signal=35%
BP	GO:0000375	RNA splicing, via transesterification reactions	250	0.394840525	1.621552327	9.35E-07	0.000168453	0.000137	4979	tags=55%, list=39%, signal=34%
BP	GO:0032956	regulation of actin cytoskeleton organization	222	0.398743655	1.637858721	1.04E-06	0.000181622	0.000148	4256	tags=53%, list=34%, signal=36%
BP	GO:0030198	extracellular matrix organization	207	0.404676124	1.656905443	1.12E-06	0.000186469	0.000152	3543	tags=45%, list=28%, signal=33%
BP	GO:0043062	extracellular structure organization	207	0.404676124	1.656905443	1.12E-06	0.000186469	0.000152	3543	tags=45%, list=28%, signal=33%
CC	GO:0005911	cell-cell junction	274	0.385771674	1.591421756	1.17E-06	0.000189629	0.000155	3531	tags=44%, list=28%, signal=32%
BP	GO:0007160	cell-matrix adhesion	136	0.440006211	1.757986482	1.22E-06	0.00019279	0.000157	3554	tags=50%, list=28%, signal=36%
BP	GO:0061564	axon development	287	0.382718959	1.582365561	1.38E-06	0.000211978	0.000173	4187	tags=50%, list=33%, signal=34%
BP	GO:0072329	monocarboxylic acid catabolic process	80	-0.357178251	-2.11819314	1.41E-06	0.000211998	0.000173	2715	tags=45%, list=22%, signal=36%
CC	GO:0030027	lamellipodium	143	0.435448621	1.746726208	1.56E-06	0.000229287	0.000187	4450	tags=57%, list=35%, signal=37%
BP	GO:0009062	fatty acid catabolic process	70	-0.377508009	-2.09649821	1.60E-06	0.000229843	0.000187	2715	tags=46%, list=22%, signal=36%
BP	GO:0006022	aminoglycan metabolic process	98	0.480286528	1.862705309	1.96E-06	0.000271244	0.000221	2302	tags=42%, list=18%, signal=34%
BP	GO:0032989	cellular component morphogenesis	435	0.35377748	1.478743212	1.98E-06	0.000271244	0.000221	4187	tags=47%, list=33%, signal=32%
BP	GO:0007411	axon guidance	149	0.431714522	1.739068565	2.09E-06	0.000272351	0.000222	4585	tags=59%, list=36%, signal=38%
BP	GO:0097485	neuron projection guidance	149	0.431714522	1.739068565	2.09E-06	0.000272351	0.000222	4585	tags=59%, list=36%, signal=38%
CC	GO:0031252	cell leading edge	262	0.38723493	1.596820311	2.12E-06	0.000272351	0.000222	4316	tags=49%, list=34%, signal=33%
BP	GO:0090130	tissue migration	198	0.402421892	1.643329063	2.33E-06	0.000293823	0.00024	4387	tags=51%, list=35%, signal=34%
CC	GO:0022625	cytosolic large ribosomal subunit	51	-0.434096387	-2.341055713	2.55E-06	0.000315664	0.000257	1704	tags=41%, list=14%, signal=36%
BP	GO:0001935	endothelial cell proliferation	85	0.487272048	1.865151398	3.27E-06	0.000388616	0.000317	3304	tags=49%, list=26%, signal=37%
BP	GO:0010631	epithelial cell migration	196	0.404074135	1.648969883	3.36E-06	0.000388616	0.000317	4387	tags=51%, list=35%, signal=34%
BP	GO:0090132	epithelium migration	196	0.404074135	1.648969883	3.36E-06	0.000388616	0.000317	4387	tags=51%, list=35%, signal=34%
BP	GO:0006397	mRNA processing	341	0.361824451	1.503871892	3.39E-06	0.000388616	0.000317	4980	tags=52%, list=40%, signal=32%
BP	GO:0051493	regulation of cytoskeleton organization	339	0.360041623	1.496212485	3.46E-06	0.000389426	0.000318	4316	tags=47%, list=34%, signal=32%
CC	GO:0015629	actin cytoskeleton	302	0.373239653	1.54566518	3.84E-06	0.000424657	0.000346	4663	tags=52%, list=37%, signal=34%
BP	GO:0071363	cellular response to growth factor stimulus	399	0.352406305	1.470974509	4.53E-06	0.000492529	0.000402	3681	tags=41%, list=29%, signal=30%
BP	GO:0030203	glycosaminoglycan metabolic process	92	0.478812213	1.846061846	4.61E-06	0.000492534	0.000402	2302	tags=41%, list=18%, signal=34%
BP	GO:0043903	regulation of symbiotic process	150	0.423921338	1.709603981	4.75E-06	0.000497899	0.000406	4110	tags=55%, list=33%, signal=37%
BP	GO:0007265	Ras protein signal transduction	223	0.390462004	1.603378591	4.81E-06	0.000497899	0.000406	4994	tags=61%, list=40%, signal=37%
BP	GO:0008380	RNA splicing	311	0.368806163	1.52786871	4.94E-06	0.000502958	0.00041	4980	tags=53%, list=40%, signal=33%
BP	GO:0120035	regulation of plasma membrane bounded cell projection organization	384	0.354746424	1.479661638	5.15E-06	0.000515743	0.000421	4190	tags=46%, list=33%, signal=32%
BP	GO:0040017	positive regulation of locomotion	314	0.366411108	1.518348594	5.36E-06	0.000528128	0.000431	4063	tags=46%, list=32%, signal=32%
BP	GO:0006325	chromatin organization	440	0.346996713	1.451148243	7.16E-06	0.000694519	0.000566	4935	tags=51%, list=39%, signal=32%
BP	GO:0044772	mitotic cell cycle phase transition	379	0.35203636	1.467218984	7.29E-06	0.000697277	0.000569	5304	tags=55%, list=42%, signal=33%
BP	GO:0044770	cell cycle phase transition	399	0.349463491	1.458690948	7.57E-06	0.000703631	0.000574	5200	tags=54%, list=41%, signal=33%
MF	GO:0015078	proton transmembrane transporter activity	60	-0.373454244	-2.07573566	7.58E-06	0.000703631	0.000574	1686	tags=33%, list=13%, signal=29%
BP	GO:0045664	regulation of neuron differentiation	363	0.354020132	1.472594107	7.71E-06	0.000705072	0.000575	3305	tags=39%, list=26%, signal=30%

BP	GO:0031344	regulation of cell projection organization	388	0.352895	1.471475152	8.27E-06	0.000734594	0.000599	4190	tags=46%, list=33%, signal=31%
BP	GO:0034330	cell junction organization	354	0.35651614	1.483202454	8.27E-06	0.000734594	0.000599	3579	tags=42%, list=28%, signal=31%
BP	GO:0071711	basement membrane organization	20	0.697650333	2.109630241	8.82E-06	0.000764025	0.000623	2543	tags=75%, list=20%, signal=60%
BP	GO:1901342	regulation of vasculature development	207	0.390264875	1.597900042	8.84E-06	0.000764025	0.000623	3121	tags=38%, list=25%, signal=29%
MF	GO:0050839	cell adhesion molecule binding	337	0.361616023	1.502735779	8.96E-06	0.00076404	0.000623	4293	tags=46%, list=34%, signal=31%
BP	GO:0006023	aminoglycan biosynthetic process	70	0.49790561	1.874864054	9.58E-06	0.000806222	0.000658	2472	tags=46%, list=20%, signal=37%
CC	GO:0005681	spliceosomal complex	140	0.415434227	1.664133259	9.89E-06	0.00082134	0.00067	4887	tags=57%, list=39%, signal=35%
BP	GO:0051272	positive regulation of cellular component movement	316	0.360001632	1.492708485	1.02E-05	0.000836726	0.000682	4063	tags=45%, list=32%, signal=31%
BP	GO:0045446	endothelial cell differentiation	67	0.510655027	1.903250976	1.05E-05	0.000847552	0.000691	3496	tags=60%, list=28%, signal=43%
BP	GO:1903510	mucopolysaccharide metabolic process	68	0.509986213	1.904516744	1.10E-05	0.000879007	0.000717	2472	tags=44%, list=20%, signal=36%
BP	GO:0006935	chemotaxis	322	0.361086229	1.499986005	1.12E-05	0.000882048	0.000719	4316	tags=46%, list=34%, signal=31%
BP	GO:0070848	response to growth factor	418	0.347332508	1.449926072	1.27E-05	0.000990197	0.000808	3681	tags=41%, list=29%, signal=30%
MF	GO:0008134	transcription factor binding	425	0.340959316	1.422958128	1.33E-05	0.001019142	0.000831	4754	tags=50%, list=38%, signal=32%
BP	GO:0007015	actin filament organization	259	0.371656239	1.531483691	1.34E-05	0.001019142	0.000831	3576	tags=44%, list=28%, signal=32%
CC	GO:0005788	endoplasmic reticulum lumen	164	0.411688027	1.667185658	1.36E-05	0.001021527	0.000833	3522	tags=46%, list=28%, signal=33%
BP	GO:0051607	defense response to virus	162	0.405397126	1.640515464	1.42E-05	0.001050924	0.000857	4743	tags=55%, list=38%, signal=35%
BP	GO:2000147	positive regulation of cell motility	307	0.359101211	1.48704437	1.44E-05	0.001055039	0.00086	4063	tags=45%, list=32%, signal=31%
MF	GO:0051015	actin filament binding	114	0.44386325	1.750787637	1.49E-05	0.001077151	0.000878	3666	tags=47%, list=29%, signal=34%
BP	GO:0034329	cell junction assembly	216	0.385618508	1.583847352	1.58E-05	0.001132292	0.000923	3554	tags=43%, list=28%, signal=31%
BP	GO:0042330	taxis	324	0.36250511	1.505971492	1.61E-05	0.001136684	0.000927	4316	tags=46%, list=34%, signal=31%
CC	GO:0005912	adherens junction	95	0.459883023	1.775953004	1.62E-05	0.001136684	0.000927	3515	tags=54%, list=28%, signal=39%
BP	GO:0050792	regulation of viral process	142	0.415092039	1.664872608	1.65E-05	0.001142865	0.000932	4110	tags=54%, list=33%, signal=36%
BP	GO:0010564	regulation of cell cycle process	470	0.34106841	1.430220931	1.75E-05	0.001196877	0.000976	5227	tags=53%, list=41%, signal=32%
MF	GO:0019904	protein domain specific binding	444	0.338091857	1.413871741	1.77E-05	0.001202951	0.000981	3840	tags=41%, list=30%, signal=29%
MF	GO:0003682	chromatin binding	360	0.346893917	1.443152088	1.86E-05	0.001222858	0.000997	4956	tags=52%, list=39%, signal=32%
BP	GO:0030335	positive regulation of cell migration	300	0.366526731	1.517003553	1.85703E-05	0.001222858	0.000997	4063	tags=45%, list=32%, signal=31%
BP	GO:1902903	regulation of supramolecular fiber organization	230	0.376837564	1.547338943	1.86074E-05	0.001222858	0.000997	4060	tags=48%, list=32%, signal=33%
MF	GO:0019900	kinase binding	469	0.34188011	1.433356496	1.91288E-05	0.001244158	0.001015	4036	tags=44%, list=32%, signal=31%
BP	GO:0009615	response to virus	212	0.386733578	1.586216598	2.05455E-05	0.00132267	0.001079	4743	tags=54%, list=38%, signal=34%
BP	GO:0051056	regulation of small GTPase mediated signal transduction	202	0.390632836	1.59889872	2.08668E-05	0.001329609	0.001084	4358	tags=52%, list=35%, signal=35%
BP	GO:0032970	regulation of actin filament-based process	243	0.379469946	1.560356748	2.13841E-05	0.001329609	0.001084	4256	tags=50%, list=34%, signal=34%
BP	GO:1903900	regulation of viral life cycle	99	0.453369247	1.762587566	2.16014E-05	0.001329609	0.001084	4110	tags=58%, list=33%, signal=39%
BP	GO:0002011	morphogenesis of an epithelial sheet	35	0.590134798	1.998955417	2.16798E-05	0.001329609	0.001084	3447	tags=71%, list=27%, signal=52%
BP	GO:0007178	transmembrane receptor protein serine/threonine kinase signaling pathway	192	0.391536045	1.597179989	2.1707E-05	0.001329609	0.001084	3852	tags=47%, list=31%, signal=33%
BP	GO:0071559	response to transforming growth factor beta	160	0.408069628	1.650233778	2.19871E-05	0.001333815	0.001088	3681	tags=45%, list=29%, signal=32%
BP	GO:0030155	regulation of cell adhesion	414	0.340809741	1.423179976	2.3876E-05	0.001434604	0.00117	3566	tags=39%, list=28%, signal=29%
BP	GO:0031424	keratinization	24	-0.579013783	-2.571031981	2.46823E-05	0.001457185	0.001188	1751	tags=67%, list=14%, signal=58%
CC	GO:0099081	supramolecular polymer	448	0.33606494	1.406041925	2.47137E-05	0.001457185	0.001188	4120	tags=43%, list=33%, signal=30%
BP	GO:1904018	positive regulation of vasculature development	120	0.432481085	1.71171825	2.56567E-05	0.001498777	0.001222	3121	tags=40%, list=25%, signal=30%

CC	GO:0099513	polymeric cytoskeletal fiber	322	0.354528626	1.472745107	2.83439E-05	0.001640568	0.001338	4740	tags=50%, list=38%, signal=32%
BP	GO:0043087	regulation of GTPase activity	287	0.365738735	1.512160202	2.89052E-05	0.001657844	0.001352	4386	tags=47%, list=35%, signal=31%
BP	GO:1903901	negative regulation of viral life cycle	54	0.516768896	1.869875532	2.99496E-05	0.00169147	0.001379	2804	tags=52%, list=22%, signal=40%
BP	GO:0042060	wound healing	278	0.365309105	1.507372304	3.00277E-05	0.00169147	0.001379	4605	tags=52%, list=37%, signal=34%
CC	GO:0022627	cytosolic small ribosomal subunit	43	-0.41959136	-2.164975163	3.06693E-05	0.001712324	0.001396	1426	tags=47%, list=11%, signal=41%
MF	GO:0008146	sulfotransferase activity	24	0.640324922	2.014994633	3.14713E-05	0.001741689	0.00142	2472	tags=62%, list=20%, signal=50%
BP	GO:0010810	regulation of cell-substrate adhesion	128	0.421397862	1.677024462	3.26973E-05	0.001785769	0.001456	3554	tags=48%, list=28%, signal=35%
CC	GO:0099512	supramolecular fiber	444	0.334767125	1.399967992	3.28339E-05	0.001785769	0.001456	4740	tags=48%, list=38%, signal=31%
BP	GO:0007507	heart development	311	0.357853455	1.48249447	3.39564E-05	0.001831035	0.001493	5138	tags=54%, list=41%, signal=33%
BP	GO:0019058	viral life cycle	224	0.37703462	1.548305413	3.56758E-05	0.001907447	0.001556	5152	tags=58%, list=41%, signal=35%
BP	GO:0048013	ephrin receptor signaling pathway	64	0.492536492	1.822209124	4.00032E-05	0.002120844	0.00173	4212	tags=61%, list=33%, signal=41%
BP	GO:0030031	cell projection assembly	298	0.355053662	1.469224927	4.12527E-05	0.00216886	0.001769	4316	tags=46%, list=34%, signal=31%
MF	GO:0060090	molecular adaptor activity	185	0.390457117	1.59139629	4.25026E-05	0.00221289	0.001805	4665	tags=55%, list=37%, signal=35%
CC	GO:0005684	U2-type spliceosomal complex	70	0.479932296	1.80718552	4.27917E-05	0.00221289	0.001805	4979	tags=64%, list=39%, signal=39%
CC	GO:0009986	cell surface	356	0.347815235	1.447031536	4.34977E-05	0.002214115	0.001806	4033	tags=43%, list=32%, signal=30%
BP	GO:0001570	vasculogenesis	53	0.516084862	1.85806675	4.35172E-05	0.002214115	0.001806	1683	tags=40%, list=13%, signal=34%
BP	GO:0051640	organelle localization	392	0.343675109	1.433070886	4.51815E-05	0.002280399	0.00186	4156	tags=43%, list=33%, signal=30%
BP	GO:0001936	regulation of endothelial cell proliferation	76	0.481212649	1.819021756	4.71602E-05	0.002344634	0.001912	3304	tags=49%, list=26%, signal=36%
BP	GO:0034446	substrate adhesion-dependent cell spreading	72	0.478018509	1.805348381	4.74492E-05	0.002344634	0.001912	4151	tags=60%, list=33%, signal=40%
CC	GO:0098687	chromosomal region	192	0.385262932	1.571590288	4.7569E-05	0.002344634	0.001912	5950	tags=64%, list=47%, signal=34%
BP	GO:0071560	cellular response to transforming growth factor beta stimulus	155	0.406274336	1.641878085	5.43788E-05	0.002654025	0.002164	3681	tags=45%, list=29%, signal=32%
BP	GO:0048729	tissue morphogenesis	374	0.341557352	1.422508761	5.47228E-05	0.002654025	0.002164	4974	tags=52%, list=39%, signal=33%
BP	GO:0006898	receptor-mediated endocytosis	145	0.410623349	1.649035552	5.51082E-05	0.002654025	0.002164	3928	tags=48%, list=31%, signal=33%
BP	GO:0120031	plasma membrane bounded cell projection assembly	291	0.354444088	1.465819069	5.92239E-05	0.002830632	0.002309	4316	tags=46%, list=34%, signal=31%
BP	GO:0150115	cell-substrate junction organization	71	0.472054943	1.781659424	6.71447E-05	0.003176022	0.00259	3235	tags=52%, list=26%, signal=39%
BP	GO:0035966	response to topologically incorrect protein	152	0.404172736	1.63020371	6.74571E-05	0.003176022	0.00259	3971	tags=45%, list=31%, signal=31%
MF	GO:0016614	oxidoreductase activity, acting on CH-OH group of donors	80	-0.310107624	-1.839047702	6.84749E-05	0.003200059	0.00261	2658	tags=42%, list=21%, signal=34%
BP	GO:0006913	nucleocytoplasmic transport	245	0.363048744	1.493136983	6.94456E-05	0.003221563	0.002627	5757	tags=60%, list=46%, signal=33%
CC	GO:0005813	centrosome	335	0.343436417	1.426802524	7.16323E-05	0.003298748	0.00269	5765	tags=60%, list=46%, signal=33%
BP	GO:0043542	endothelial cell migration	145	0.40707616	1.63479028	7.44088E-05	0.003401775	0.002774	3323	tags=42%, list=26%, signal=31%
BP	GO:0006986	response to unfolded protein	138	0.40982314	1.637481434	7.59267E-05	0.003446198	0.002811	3971	tags=46%, list=31%, signal=32%
BP	GO:0010638	positive regulation of organelle organization	405	0.333681435	1.393582388	7.72726E-05	0.003482234	0.00284	4050	tags=42%, list=32%, signal=29%
BP	GO:0060759	regulation of response to cytokine stimulus	117	0.422196036	1.667372322	7.84481E-05	0.003510136	0.002863	5011	tags=64%, list=40%, signal=39%
BP	GO:0006029	proteoglycan metabolic process	56	0.503556103	1.825751438	8.49607E-05	0.003766278	0.003072	3153	tags=50%, list=25%, signal=38%
CC	GO:0005884	actin filament	73	0.471181412	1.774782086	8.53666E-05	0.003766278	0.003072	4740	tags=62%, list=38%, signal=39%
MF	GO:0019901	protein kinase binding	414	0.332863156	1.389996002	9.75775E-05	0.004275112	0.003487	3498	tags=39%, list=28%, signal=29%
BP	GO:0034340	response to type I interferon	72	0.469575971	1.773463168	0.000100048	0.004353102	0.00355	3932	tags=58%, list=31%, signal=40%
MF	GO:0005201	extracellular matrix structural constituent	91	0.443356484	1.709365061	0.000105461	0.004497152	0.003668	3450	tags=46%, list=27%, signal=34%
BP	GO:0061437	renal system vasculature development	19	0.663233326	1.982513506	0.000106031	0.004497152	0.003668	1135	tags=47%, list=9%, signal=43%

BP	GO:0061440	kidney vasculature development	19	0.663233326	1.982513506	0.000106031	0.004497152	0.003668	1135	tags=47%, list=9%, signal=43%
BP	GO:0009611	response to wounding	344	0.345257799	1.435136431	0.000106602	0.004497152	0.003668	4605	tags=49%, list=37%, signal=32%
MF	GO:0030674	protein-macromolecule adaptor activity	161	0.393657778	1.593151655	0.000106922	0.004497152	0.003668	4665	tags=55%, list=37%, signal=35%
BP	GO:0050907	detection of chemical stimulus involved in sensory perception	18	-0.601356854	-2.438675055	0.0001097	0.004583416	0.003738	2221	tags=67%, list=18%, signal=55%
MF	GO:0004029	aldehyde dehydrogenase (NAD+) activity	12	-0.721574665	-2.509937854	0.00012356	0.005095049	0.004155	1822	tags=67%, list=14%, signal=57%
MF	GO:0004030	aldehyde dehydrogenase [NAD(P)+] activity	12	-0.721574665	-2.509937854	0.00012356	0.005095049	0.004155	1822	tags=67%, list=14%, signal=57%
BP	GO:0070268	cornification	17	-0.634265828	-2.457175102	0.000129348	0.005299072	0.004322	1751	tags=71%, list=14%, signal=61%
CC	GO:0098978	glutamatergic synapse	165	0.389605995	1.578511486	0.000131023	0.005333069	0.004349	4408	tags=51%, list=35%, signal=34%
BP	GO:0017144	drug metabolic process	13	-0.680756746	-2.399446082	0.000133145	0.005384675	0.004391	994	tags=69%, list=8%, signal=64%
BP	GO:0090287	regulation of cellular response to growth factor stimulus	161	0.390026426	1.578455402	0.000139857	0.005586409	0.004556	3153	tags=40%, list=25%, signal=31%
BP	GO:0006024	glycosaminoglycan biosynthetic process	67	0.475581438	1.772528984	0.000139904	0.005586409	0.004556	2472	tags=43%, list=20%, signal=35%
BP	GO:0006909	phagocytosis	169	0.391109341	1.586776138	0.000144695	0.005741385	0.004682	4159	tags=48%, list=33%, signal=33%
BP	GO:0007179	transforming growth factor beta receptor signaling pathway	126	0.410362067	1.632682444	0.000150309	0.005908275	0.004818	4013	tags=49%, list=32%, signal=34%
MF	GO:0016462	pyrophosphatase activity	495	0.322183685	1.353329632	0.000150774	0.005908275	0.004818	4524	tags=45%, list=36%, signal=30%
CC	GO:0001533	cornified envelope	11	-0.723958523	-2.438849539	0.000154251	0.006007214	0.004899	1751	tags=82%, list=14%, signal=71%
BP	GO:0001959	regulation of cytokine-mediated signaling pathway	110	0.423578911	1.665341309	0.000157511	0.006025116	0.004914	5007	tags=64%, list=40%, signal=39%
MF	GO:0016817	hydrolase activity, acting on acid anhydrides	498	0.321550263	1.350882	0.000157576	0.006025116	0.004914	4524	tags=45%, list=36%, signal=30%
MF	GO:0016818	hydrolase activity, acting on acid anhydrides, in phosphorus-containing anhydrides	498	0.321550263	1.350882	0.000157576	0.006025116	0.004914	4524	tags=45%, list=36%, signal=30%
BP	GO:0048002	antigen processing and presentation of peptide antigen	143	0.392742072	1.575416337	0.000159005	0.006041467	0.004927	5328	tags=62%, list=42%, signal=36%
BP	GO:0001937	negative regulation of endothelial cell proliferation	22	0.635969553	1.958807248	0.00016097	0.006041467	0.004927	3531	tags=68%, list=28%, signal=49%
BP	GO:0008299	isoprenoid biosynthetic process	20	-0.554591985	-2.311282069	0.000163649	0.006041467	0.004927	2015	tags=50%, list=16%, signal=42%
BP	GO:0035967	cellular response to topologically incorrect protein	126	0.408766281	1.626333389	0.000164077	0.006041467	0.004927	3971	tags=45%, list=31%, signal=31%
BP	GO:0002478	antigen processing and presentation of exogenous peptide antigen	136	0.397185364	1.586901462	0.000164243	0.006041467	0.004927	5328	tags=62%, list=42%, signal=36%
BP	GO:0051169	nuclear transport	247	0.361131652	1.484550332	0.000164542	0.006041467	0.004927	5757	tags=60%, list=46%, signal=33%
BP	GO:1903311	regulation of mRNA metabolic process	229	0.364526626	1.496804981	0.000164706	0.006041467	0.004927	4961	tags=53%, list=39%, signal=33%
CC	GO:0032432	actin filament bundle	53	0.495059804	1.782369975	0.000170943	0.006233984	0.005084	3594	tags=57%, list=29%, signal=41%
MF	GO:0004984	olfactory receptor activity	17	-0.625496279	-2.423201463	0.000174489	0.006290582	0.00513	2221	tags=71%, list=18%, signal=58%
BP	GO:0050911	detection of chemical stimulus involved in sensory perception of smell	17	-0.625496279	-2.423201463	0.000174489	0.006290582	0.00513	2221	tags=71%, list=18%, signal=58%
BP	GO:0006635	fatty acid beta-oxidation	49	-0.364090508	-1.901610304	0.000177276	0.006354734	0.005183	2626	tags=43%, list=21%, signal=34%
BP	GO:0006458	'de novo' protein folding	30	0.579108924	1.897868741	0.000183566	0.006543031	0.005336	3016	tags=50%, list=24%, signal=38%
BP	GO:0010975	regulation of neuron projection development	288	0.353330475	1.460865546	0.000184782	0.006549395	0.005341	4190	tags=47%, list=33%, signal=32%
MF	GO:0017111	nucleoside-triphosphatase activity	464	0.324879489	1.361417982	0.00018705	0.006592716	0.005377	5368	tags=53%, list=43%, signal=32%
BP	GO:0019079	viral genome replication	83	0.443925131	1.692386964	0.000194633	0.006798993	0.005545	5433	tags=70%, list=43%, signal=40%
BP	GO:0061077	chaperone-mediated protein folding	44	0.524328641	1.841176918	0.000195057	0.006798993	0.005545	3016	tags=50%, list=24%, signal=38%
BP	GO:0019884	antigen processing and presentation of exogenous antigen	138	0.39744113	1.588008114	0.000200793	0.006960455	0.005677	5328	tags=62%, list=42%, signal=36%
MF	GO:0003725	double-stranded RNA binding	54	0.491119579	1.777066093	0.000202032	0.006965143	0.00568	4634	tags=69%, list=37%, signal=44%
BP	GO:0043547	positive regulation of GTPase activity	236	0.3602267	1.479917393	0.000205393	0.007042516	0.005743	4386	tags=47%, list=35%, signal=31%
CC	GO:0005819	spindle	206	0.365000774	1.495001232	0.00020787	0.007088934	0.005781	5013	tags=54%, list=40%, signal=33%
BP	GO:0002040	sprouting angiogenesis	81	0.450088318	1.713334442	0.000213728	0.007233107	0.005899	3120	tags=47%, list=25%, signal=36%

BP	GO:1902624	positive regulation of neutrophil migration	16	0.675786636	1.945955443	0.000214391	0.007233107	0.005899	1678	tags=50%, list=13%, signal=43%
CC	GO:0097225	sperm midpiece	11	-0.715504938	-2.410371357	0.000216953	0.007271101	0.00593	2302	tags=82%, list=18%, signal=67%
BP	GO:0000209	protein polyubiquitination	248	0.351698948	1.445233665	0.00021901	0.007271101	0.00593	4857	tags=49%, list=39%, signal=31%
CC	GO:0001725	stress fiber	50	0.498744015	1.779735593	0.00022109	0.007271101	0.00593	3594	tags=56%, list=29%, signal=40%
CC	GO:0097517	contractile actin filament bundle	50	0.498744015	1.779735593	0.00022109	0.007271101	0.00593	3594	tags=56%, list=29%, signal=40%
MF	GO:0140098	catalytic activity, acting on RNA	235	0.358702393	1.473231118	0.000221279	0.007271101	0.00593	4781	tags=49%, list=38%, signal=31%
BP	GO:0044089	positive regulation of cellular component biogenesis	313	0.346719221	1.436046141	0.000223326	0.007300315	0.005954	4060	tags=44%, list=32%, signal=30%
BP	GO:0010769	regulation of cell morphogenesis involved in differentiation	186	0.372869877	1.519336133	0.000244776	0.007960263	0.006492	4187	tags=51%, list=33%, signal=35%
CC	GO:0062023	collagen-containing extracellular matrix	209	0.362192396	1.483453473	0.000248852	0.008051313	0.006566	3543	tags=40%, list=28%, signal=29%
BP	GO:0060627	regulation of vesicle-mediated transport	301	0.348702451	1.443568426	0.000252911	0.00814091	0.006639	5266	tags=55%, list=42%, signal=33%
BP	GO:0019221	cytokine-mediated signaling pathway	462	0.32125247	1.345481736	0.000257468	0.00824552	0.006725	4421	tags=44%, list=35%, signal=30%
BP	GO:0045069	regulation of viral genome replication	67	0.467294597	1.741643284	0.00026677	0.00850025	0.006932	5433	tags=78%, list=43%, signal=44%
BP	GO:0051495	positive regulation of cytoskeleton organization	141	0.387167097	1.552273791	0.000282795	0.0089656	0.007312	4116	tags=48%, list=33%, signal=32%
BP	GO:0051345	positive regulation of hydrolase activity	446	0.322075832	1.347105755	0.000284809	0.008984312	0.007327	4429	tags=43%, list=35%, signal=29%
BP	GO:0006720	isoprenoid metabolic process	70	-0.312766459	-1.73695473	0.000291313	0.009143754	0.007457	1181	tags=26%, list=9%, signal=23%
CC	GO:0005815	microtubule organizing center	422	0.324748572	1.355730758	0.000293923	0.009180003	0.007487	5767	tags=56%, list=46%, signal=32%
MF	GO:0008194	UDP-glycosyltransferase activity	63	0.469611453	1.729865563	0.000306839	0.009445233	0.007703	3119	tags=46%, list=25%, signal=35%
BP	GO:0032092	positive regulation of protein binding	63	0.469980019	1.731223219	0.000306839	0.009445233	0.007703	5010	tags=67%, list=40%, signal=40%
CC	GO:0005905	clathrin-coated pit	44	0.516673594	1.814296266	0.00030821	0.009445233	0.007703	4503	tags=68%, list=36%, signal=44%
BP	GO:0010811	positive regulation of cell-substrate adhesion	68	0.469053645	1.751656218	0.000308404	0.009445233	0.007703	2494	tags=44%, list=20%, signal=36%
CC	GO:0005667	transcription regulator complex	256	0.350494844	1.441735305	0.000321264	0.009791575	0.007985	4983	tags=54%, list=40%, signal=33%
BP	GO:0003279	cardiac septum development	65	0.468567966	1.737329794	0.000328908	0.009941868	0.008108	4404	tags=65%, list=35%, signal=42%
BP	GO:0060337	type I interferon signaling pathway	69	0.456933677	1.713421292	0.000330923	0.009941868	0.008108	3932	tags=57%, list=31%, signal=39%
BP	GO:0071357	cellular response to type I interferon	69	0.456933677	1.713421292	0.000330923	0.009941868	0.008108	3932	tags=57%, list=31%, signal=39%
BP	GO:0050767	regulation of neurogenesis	449	0.322417095	1.349128735	0.000334935	0.01001471	0.008167	3309	tags=37%, list=26%, signal=28%
BP	GO:0110053	regulation of actin filament organization	174	0.380244335	1.545146905	0.000341196	0.010153799	0.008281	4247	tags=51%, list=34%, signal=34%
BP	GO:0150116	regulation of cell-substrate junction organization	47	0.500482065	1.777382929	0.000364626	0.010800112	0.008808	3235	tags=57%, list=26%, signal=43%
MF	GO:1990782	protein tyrosine kinase binding	56	0.484024265	1.75493454	0.000367548	0.0108358	0.008837	1880	tags=39%, list=15%, signal=34%
BP	GO:0034620	cellular response to unfolded protein	112	0.416257828	1.637013644	0.000372792	0.010939267	0.008921	3971	tags=46%, list=31%, signal=32%
BP	GO:0007044	cell-substrate junction assembly	68	0.466089856	1.740588101	0.000380124	0.011062658	0.009022	3235	tags=51%, list=26%, signal=38%
BP	GO:0007169	transmembrane receptor protein tyrosine kinase signaling pathway	442	0.321510821	1.34489629	0.000380504	0.011062658	0.009022	4590	tags=45%, list=36%, signal=30%
BP	GO:0045765	regulation of angiogenesis	189	0.372687854	1.520167796	0.000390788	0.011309562	0.009223	3121	tags=37%, list=25%, signal=28%
BP	GO:0030900	forebrain development	196	0.365944053	1.493366365	0.000395359	0.01138959	0.009289	4371	tags=48%, list=35%, signal=32%
MF	GO:0016616	oxidoreductase activity, acting on the CH-OH group of donors, NAD or NADP as acceptor	74	-0.298141496	-1.734531304	0.000406296	0.011651462	0.009502	2658	tags=42%, list=21%, signal=33%
BP	GO:0030968	endoplasmic reticulum unfolded protein response	95	0.422471969	1.631480889	0.000423834	0.012099399	0.009868	3971	tags=48%, list=31%, signal=33%
MF	GO:0008047	enzyme activator activity	323	0.336544175	1.398075418	0.000430629	0.012238016	0.009981	4386	tags=44%, list=35%, signal=29%
CC	GO:0098857	membrane microdomain	177	0.374743265	1.52243984	0.000451563	0.012775396	0.010419	4176	tags=47%, list=33%, signal=32%
MF	GO:0045296	cadherin binding	233	0.356700725	1.465589678	0.00046299	0.013040193	0.010635	4435	tags=47%, list=35%, signal=31%
MF	GO:0046982	protein heterodimerization activity	137	0.392870751	1.570637625	0.000467667	0.01311337	0.010695	4315	tags=53%, list=34%, signal=35%

MF	GO:0008270	zinc ion binding	438	0.320178976	1.338575063	0.000471686	0.013162142	0.010734	4270	tags=43%, list=34%, signal=29%
MF	GO:0030695	GTPase regulator activity	187	0.36434896	1.48578635	0.000473578	0.013162142	0.010734	4386	tags=47%, list=35%, signal=31%
BP	GO:0007346	regulation of mitotic cell cycle	407	0.32377885	1.352302654	0.000480845	0.013305478	0.010851	5417	tags=53%, list=43%, signal=31%
BP	GO:0002479	antigen processing and presentation of exogenous peptide antigen via MHC class I, TAP-dependent	67	0.460476616	1.716232138	0.000487871	0.013440961	0.010962	4719	tags=64%, list=37%, signal=40%
BP	GO:0002009	morphogenesis of an epithelium	324	0.3396595	1.411062933	0.000490003	0.013441005	0.010962	4970	tags=52%, list=39%, signal=32%
BP	GO:0045766	positive regulation of angiogenesis	105	0.408751769	1.599640091	0.000517629	0.014137314	0.01153	3121	tags=38%, list=25%, signal=29%
BP	GO:1902905	positive regulation of supramolecular fiber organization	130	0.393089553	1.563227569	0.000534108	0.014524518	0.011845	4045	tags=48%, list=32%, signal=33%
MF	GO:0140297	DNA-binding transcription factor binding	233	0.354977708	1.458510253	0.0005455	0.014770653	0.012046	5571	tags=58%, list=44%, signal=33%
MF	GO:0016903	oxidoreductase activity, acting on the aldehyde or oxo group of donors	31	-0.426242629	-2.04533327	0.000554427	0.014857691	0.012117	1822	tags=42%, list=14%, signal=36%
MF	GO:0050840	extracellular matrix binding	38	0.521722866	1.790261484	0.00055549	0.014857691	0.012117	1890	tags=45%, list=15%, signal=38%
BP	GO:0032102	negative regulation of response to external stimulus	204	0.357684668	1.463866118	0.00055578	0.014857691	0.012117	3221	tags=37%, list=26%, signal=28%
CC	GO:0005938	cell cortex	167	0.381474655	1.546721618	0.000574152	0.01528408	0.012465	4445	tags=50%, list=35%, signal=33%
BP	GO:0050817	coagulation	178	0.365967252	1.488015953	0.000593715	0.015738427	0.012835	4605	tags=52%, list=37%, signal=34%
BP	GO:0032366	intracellular sterol transport	15	-0.600553614	-2.218095029	0.000624861	0.016423921	0.013394	1370	tags=53%, list=11%, signal=48%
BP	GO:0032367	intracellular cholesterol transport	15	-0.600553614	-2.218095029	0.000624861	0.016423921	0.013394	1370	tags=53%, list=11%, signal=48%
BP	GO:0098609	cell-cell adhesion	420	0.319320782	1.333417806	0.000627384	0.016423921	0.013394	3791	tags=39%, list=30%, signal=28%
BP	GO:0036498	IRE1-mediated unfolded protein response	56	0.474747402	1.721299269	0.000631099	0.016424438	0.013395	3971	tags=57%, list=31%, signal=39%
MF	GO:0019955	cytokine binding	64	0.456446413	1.688688723	0.000633447	0.016424438	0.013395	2623	tags=41%, list=21%, signal=32%
BP	GO:0002429	immune response-activating cell surface receptor signaling pathway	229	0.352291004	1.446563546	0.000637817	0.016424438	0.013395	4668	tags=49%, list=37%, signal=31%
BP	GO:0002757	immune response-activating signal transduction	229	0.352291004	1.446563546	0.000637817	0.016424438	0.013395	4668	tags=49%, list=37%, signal=31%
CC	GO:0030496	midbody	118	0.39591976	1.567962776	0.000644918	0.016492834	0.013451	5227	tags=61%, list=41%, signal=36%
BP	GO:0051960	regulation of nervous system development	491	0.313053445	1.314608896	0.000645701	0.016492834	0.013451	4190	tags=42%, list=33%, signal=29%
BP	GO:0048844	artery morphogenesis	37	0.512111031	1.751516775	0.00065773	0.016675714	0.0136	3201	tags=57%, list=25%, signal=42%
BP	GO:0019882	antigen processing and presentation	165	0.373526367	1.513363931	0.000658148	0.016675714	0.0136	5328	tags=59%, list=42%, signal=34%
BP	GO:0043648	dicarboxylic acid metabolic process	55	-0.319929898	-1.725611917	0.000661221	0.016686583	0.013609	2442	tags=44%, list=19%, signal=35%
BP	GO:0001938	positive regulation of endothelial cell proliferation	56	0.473847494	1.71803646	0.000670236	0.016846693	0.013739	3153	tags=46%, list=25%, signal=35%
BP	GO:0035909	aorta morphogenesis	18	0.640815137	1.893062613	0.000690366	0.017159829	0.013995	3831	tags=89%, list=30%, signal=62%
MF	GO:0004386	helicase activity	118	0.395028965	1.564434957	0.000691314	0.017159829	0.013995	5524	tags=63%, list=44%, signal=36%
BP	GO:0072012	glomerulus vasculature development	17	0.645351903	1.889514335	0.000692238	0.017159829	0.013995	1135	tags=47%, list=9%, signal=43%
CC	GO:0098589	membrane region	184	0.372357192	1.518247908	0.000693574	0.017159829	0.013995	4176	tags=47%, list=33%, signal=32%
BP	GO:0001885	endothelial cell development	43	0.500200416	1.747395939	0.00071641	0.017655583	0.014399	3496	tags=60%, list=28%, signal=44%
BP	GO:0007193	adenylate cyclase-inhibiting G protein-coupled receptor signaling pathway	31	0.549468123	1.813909036	0.000725418	0.017808014	0.014523	1996	tags=45%, list=16%, signal=38%
BP	GO:0030111	regulation of Wnt signaling pathway	221	0.351812143	1.443845668	0.000729588	0.017840965	0.01455	4852	tags=52%, list=38%, signal=33%
CC	GO:0000775	chromosome, centromeric region	112	0.407941331	1.604307429	0.000749265	0.018251402	0.014885	5754	tags=66%, list=46%, signal=36%
BP	GO:1905114	cell surface receptor signaling pathway involved in cell-cell signaling	348	0.326583333	1.358460771	0.000755606	0.018335077	0.014953	4852	tags=49%, list=38%, signal=31%
BP	GO:0051168	nuclear export	146	0.383790693	1.54322348	0.000759399	0.018356518	0.014971	5757	tags=62%, list=46%, signal=34%
BP	GO:2001258	negative regulation of cation channel activity	22	0.608115424	1.873015611	0.000768482	0.018505159	0.015092	2288	tags=55%, list=18%, signal=45%
BP	GO:0003013	circulatory system process	252	0.344278273	1.414387932	0.000793034	0.018937203	0.015444	3153	tags=35%, list=25%, signal=27%

CC	GO:0030427	site of polarized growth	114	0.400559595	1.57997939	0.000793386	0.018937203	0.015444	4552	tags=57%, list=36%, signal=37%
BP	GO:0035313	wound healing, spreading of epidermal cells	11	0.727452931	1.885594564	0.000795429	0.018937203	0.015444	2543	tags=82%, list=20%, signal=65%
BP	GO:0050878	regulation of body fluid levels	268	0.343204317	1.415797881	0.0008014	0.019007642	0.015502	4605	tags=47%, list=37%, signal=30%
BP	GO:0051129	negative regulation of cellular component organization	450	0.321349874	1.344998174	0.000810559	0.019152867	0.01562	4247	tags=44%, list=34%, signal=30%
BP	GO:0051648	vesicle localization	146	0.382947605	1.539833419	0.000814628	0.019177191	0.01564	4116	tags=49%, list=33%, signal=33%
BP	GO:0008284	positive regulation of cell population proliferation	473	0.316038357	1.325002282	0.000819718	0.019225272	0.015679	3708	tags=37%, list=29%, signal=27%
BP	GO:0042590	antigen processing and presentation of exogenous peptide antigen via MHC class I	71	0.43701103	1.649394485	0.000829578	0.019352958	0.015783	4719	tags=61%, list=37%, signal=38%
BP	GO:0007229	integrin-mediated signaling pathway	70	0.441157509	1.661179022	0.000831297	0.019352958	0.015783	3554	tags=50%, list=28%, signal=36%
BP	GO:0050684	regulation of mRNA processing	92	0.420478134	1.621154637	0.000837326	0.019421665	0.015839	5491	tags=63%, list=44%, signal=36%
BP	GO:0042692	muscle cell differentiation	186	0.359680598	1.465593664	0.000850593	0.019430612	0.015847	3350	tags=39%, list=27%, signal=29%
BP	GO:0032271	regulation of protein polymerization	139	0.386169394	1.54613497	0.00085488	0.019430612	0.015847	4316	tags=50%, list=34%, signal=33%
BP	GO:0030166	proteoglycan biosynthetic process	41	0.508235981	1.766351459	0.000856684	0.019430612	0.015847	3153	tags=54%, list=25%, signal=40%
BP	GO:0006611	protein export from nucleus	136	0.381984899	1.526170021	0.000858341	0.019430612	0.015847	5757	tags=62%, list=46%, signal=34%
BP	GO:0052372	modulation by symbiont of entry into host	27	0.568867922	1.836726444	0.000862442	0.019430612	0.015847	2661	tags=56%, list=21%, signal=44%
CC	GO:0005874	microtubule	219	0.347636719	1.426667994	0.000867244	0.019430612	0.015847	4733	tags=49%, list=38%, signal=31%
MF	GO:0003727	single-stranded RNA binding	58	0.466015257	1.704189001	0.000868293	0.019430612	0.015847	3992	tags=59%, list=32%, signal=40%
MF	GO:0050431	transforming growth factor beta binding	14	0.679889632	1.890782816	0.000868609	0.019430612	0.015847	2492	tags=71%, list=20%, signal=57%
CC	GO:0031253	cell projection membrane	170	0.366703017	1.487984745	0.000869856	0.019430612	0.015847	3495	tags=38%, list=28%, signal=28%
BP	GO:0016055	Wnt signaling pathway	310	0.333051027	1.379645561	0.00087467	0.019430612	0.015847	4852	tags=50%, list=38%, signal=32%
BP	GO:0061061	muscle structure development	347	0.328330667	1.365553461	0.00087467	0.019430612	0.015847	3377	tags=37%, list=27%, signal=28%
BP	GO:0198738	cell-cell signaling by wnt	310	0.333051027	1.379645561	0.00087467	0.019430612	0.015847	4852	tags=50%, list=38%, signal=32%
BP	GO:0010632	regulation of epithelial cell migration	156	0.380883759	1.539006826	0.000887375	0.019593863	0.01598	4387	tags=49%, list=35%, signal=33%
BP	GO:0007596	blood coagulation	177	0.369202923	1.499931527	0.000889159	0.019593863	0.01598	4605	tags=53%, list=37%, signal=34%
BP	GO:0016570	histone modification	275	0.337161737	1.390645986	0.000892988	0.019593863	0.01598	4931	tags=52%, list=39%, signal=32%
BP	GO:0030177	positive regulation of Wnt signaling pathway	116	0.399803503	1.580132416	0.000895458	0.019593863	0.01598	4737	tags=54%, list=38%, signal=34%
BP	GO:1902600	proton transmembrane transport	77	-0.293248014	-1.712711517	0.000897547	0.019593863	0.01598	1901	tags=32%, list=15%, signal=28%
MF	GO:0005509	calcium ion binding	285	0.335059578	1.38521295	0.000920464	0.020024853	0.016331	4204	tags=44%, list=33%, signal=30%
BP	GO:0010469	regulation of signaling receptor activity	65	0.45358986	1.68179482	0.000931277	0.02014596	0.01643	1568	tags=32%, list=12%, signal=28%
BP	GO:0007599	hemostasis	182	0.369105305	1.504040076	0.000932417	0.02014596	0.01643	4605	tags=52%, list=37%, signal=33%
BP	GO:0035904	aorta development	34	0.532498995	1.788859643	0.000942268	0.02028931	0.016547	4541	tags=76%, list=36%, signal=49%
CC	GO:0016607	nuclear speck	259	0.337744291	1.391742743	0.000966438	0.020569395	0.016775	5067	tags=52%, list=40%, signal=32%
BP	GO:1901987	regulation of cell cycle phase transition	299	0.330763988	1.369042219	0.000966438	0.020569395	0.016775	5417	tags=54%, list=43%, signal=31%
BP	GO:0002831	regulation of response to biotic stimulus	253	0.344288204	1.41495788	0.000967405	0.020569395	0.016775	5176	tags=51%, list=41%, signal=31%
BP	GO:0002474	antigen processing and presentation of peptide antigen via MHC class I	86	0.417653596	1.597872273	0.000968317	0.020569395	0.016775	4719	tags=58%, list=37%, signal=37%
BP	GO:0018212	peptidyl-tyrosine modification	206	0.349960444	1.433397767	0.000986818	0.020892058	0.017038	3002	tags=34%, list=24%, signal=26%
BP	GO:0060840	artery development	54	0.465912141	1.68585555	0.00099206	0.0209328	0.017072	4974	tags=70%, list=39%, signal=43%
BP	GO:1901990	regulation of mitotic cell cycle phase transition	284	0.33944567	1.403175986	0.001003254	0.02109843	0.017207	5258	tags=53%, list=42%, signal=32%
BP	GO:0035023	regulation of Rho protein signal transduction	53	0.46593542	1.67751309	0.001013866	0.021250766	0.017331	2593	tags=43%, list=21%, signal=35%
BP	GO:0044843	cell cycle G1/S phase transition	180	0.359673307	1.464347296	0.001026791	0.021379609	0.017436	5178	tags=56%, list=41%, signal=33%

CC	GO:0045121	membrane raft	176	0.371264461	1.509236135	0.001026791	0.021379609	0.017436	4176	tags=47%, list=33%, signal=32%
BP	GO:0043487	regulation of RNA stability	142	0.371989216	1.491993576	0.001030931	0.021395206	0.017449	4784	tags=53%, list=38%, signal=33%
BP	GO:0051017	actin filament bundle assembly	109	0.398131433	1.561302448	0.001041429	0.021471818	0.017511	4060	tags=54%, list=32%, signal=37%
BP	GO:0061572	actin filament bundle organization	109	0.398131433	1.561302448	0.001041429	0.021471818	0.017511	4060	tags=54%, list=32%, signal=37%
BP	GO:0070831	basement membrane assembly	11	0.720305351	1.867067678	0.001066459	0.021845099	0.017816	2543	tags=82%, list=20%, signal=65%
BP	GO:1905564	positive regulation of vascular endothelial cell proliferation	11	0.719942687	1.866127634	0.001066459	0.021845099	0.017816	1880	tags=55%, list=15%, signal=46%
BP	GO:0051893	regulation of focal adhesion assembly	44	0.493711702	1.733665717	0.001085683	0.022095407	0.01802	3235	tags=57%, list=26%, signal=42%
BP	GO:0090109	regulation of cell-substrate junction assembly	44	0.493711702	1.733665717	0.001085683	0.022095407	0.01802	3235	tags=57%, list=26%, signal=42%
BP	GO:0006457	protein folding	154	0.370860623	1.498440904	0.001099687	0.022095407	0.01802	4261	tags=45%, list=34%, signal=30%
BP	GO:0045071	negative regulation of viral genome replication	39	0.518938367	1.787642286	0.001099953	0.022095407	0.01802	2804	tags=54%, list=22%, signal=42%
BP	GO:0043393	regulation of protein binding	136	0.379489246	1.516198968	0.001109705	0.022095407	0.01802	4406	tags=52%, list=35%, signal=34%
CC	GO:0000139	Golgi membrane	478	0.314070318	1.317536839	0.001113701	0.022095407	0.01802	5604	tags=54%, list=44%, signal=31%
BP	GO:0016569	covalent chromatin modification	282	0.333342209	1.377718837	0.001113701	0.022095407	0.01802	4931	tags=51%, list=39%, signal=32%
BP	GO:0022411	cellular component disassembly	384	0.31791828	1.326049964	0.001113701	0.022095407	0.01802	4984	tags=47%, list=40%, signal=30%
BP	GO:0045596	negative regulation of cell differentiation	389	0.319252375	1.331330436	0.001113701	0.022095407	0.01802	3309	tags=36%, list=26%, signal=27%
MF	GO:0051020	GTPase binding	345	0.323373617	1.344133409	0.001113701	0.022095407	0.01802	4624	tags=48%, list=37%, signal=31%
BP	GO:0040029	regulation of gene expression, epigenetic	104	0.405108024	1.58248291	0.001117623	0.022103715	0.018027	5394	tags=62%, list=43%, signal=36%
BP	GO:0007369	gastrulation	95	0.409709162	1.582194125	0.001144574	0.022565983	0.018404	3153	tags=42%, list=25%, signal=32%
BP	GO:0030833	regulation of actin filament polymerization	101	0.408486287	1.590498672	0.001154013	0.02264115	0.018465	4408	tags=54%, list=35%, signal=36%
BP	GO:0034440	lipid oxidation	69	-0.285321929	-1.616335571	0.001155564	0.02264115	0.018465	2679	tags=42%, list=21%, signal=33%
BP	GO:0048041	focal adhesion assembly	60	0.463787321	1.701596808	0.00116266	0.022709666	0.018521	3235	tags=52%, list=26%, signal=39%
BP	GO:0085029	extracellular matrix assembly	24	0.570623107	1.795654766	0.001180928	0.022995287	0.018754	3708	tags=67%, list=29%, signal=47%
BP	GO:0018205	peptidyl-lysine modification	252	0.340917984	1.400582959	0.00118852	0.023071912	0.018816	5250	tags=54%, list=42%, signal=32%
BP	GO:0006403	RNA localization	174	0.366429484	1.489009382	0.001193293	0.023093513	0.018834	5508	tags=57%, list=44%, signal=33%
MF	GO:0016757	transferase activity, transferring glycosyl groups	142	0.37056945	1.486299107	0.001198105	0.023115725	0.018852	3751	tags=42%, list=30%, signal=30%
BP	GO:0045601	regulation of endothelial cell differentiation	24	0.570033426	1.793799141	0.001202205	0.023124122	0.018859	3496	tags=62%, list=28%, signal=45%
MF	GO:0017081	chloride channel regulator activity	10	-0.672157983	-2.147584722	0.001214428	0.023288217	0.018993	1634	tags=70%, list=13%, signal=61%
BP	GO:0000280	nuclear division	204	0.349153515	1.428951381	0.001246291	0.023826823	0.019432	5258	tags=53%, list=42%, signal=32%
MF	GO:0016836	hydro-lyase activity	30	-0.423651149	-2.000552734	0.001279022	0.024336159	0.019847	1621	tags=43%, list=13%, signal=38%
BP	GO:0045666	positive regulation of neuron differentiation	203	0.352483824	1.44252831	0.001283218	0.024336159	0.019847	4063	tags=45%, list=32%, signal=31%
MF	GO:0005096	GTPase activator activity	164	0.369442979	1.496108693	0.001284505	0.024336159	0.019847	4386	tags=47%, list=35%, signal=31%
MF	GO:0019787	ubiquitin-like protein transferase activity	294	0.333157786	1.37842628	0.00129778	0.024514051	0.019992	5112	tags=53%, list=41%, signal=32%
BP	GO:0009101	glycoprotein biosynthetic process	189	0.359619948	1.466864719	0.001301681	0.024514345	0.019993	4168	tags=44%, list=33%, signal=30%
CC	GO:0150034	distal axon	159	0.367782316	1.48656666	0.001338608	0.025092186	0.020464	4605	tags=53%, list=37%, signal=34%
BP	GO:0044319	wound healing, spreading of cells	21	0.584778968	1.782784524	0.001344295	0.025092186	0.020464	3304	tags=71%, list=26%, signal=53%
BP	GO:0090505	epiboly involved in wound healing	21	0.584778968	1.782784524	0.001344295	0.025092186	0.020464	3304	tags=71%, list=26%, signal=53%
BP	GO:0043254	regulation of protein-containing complex assembly	273	0.332959909	1.374163034	0.001353004	0.025180236	0.020536	4754	tags=48%, list=38%, signal=31%
MF	GO:0031267	small GTPase binding	274	0.332400986	1.37125195	0.001371412	0.025447752	0.020754	4624	tags=49%, list=37%, signal=32%
BP	GO:0033209	tumor necrosis factor-mediated signaling pathway	116	0.393923802	1.556894233	0.001389456	0.025692097	0.020953	4366	tags=52%, list=35%, signal=34%

CC	GO:0042383	sarcolemma	73	0.434548761	1.636799196	0.001393045	0.025692097	0.020953	2937	tags=40%, list=23%, signal=31%
BP	GO:0044839	cell cycle G2/M phase transition	179	0.3517851	1.431078156	0.001396796	0.025692097	0.020953	5304	tags=55%, list=42%, signal=32%
BP	GO:0010770	positive regulation of cell morphogenesis involved in differentiation	99	0.399493793	1.553133118	0.0014051	0.025769702	0.021016	4541	tags=59%, list=36%, signal=38%
BP	GO:0008037	cell recognition	63	0.441129419	1.624948853	0.00141518	0.025858772	0.021089	3751	tags=48%, list=30%, signal=34%
MF	GO:0016758	transferase activity, transferring hexosyl groups	102	0.406530751	1.58488374	0.001418154	0.025858772	0.021089	3119	tags=40%, list=25%, signal=30%
BP	GO:0051258	protein polymerization	181	0.354526563	1.444251614	0.001432359	0.025973727	0.021183	4421	tags=48%, list=35%, signal=31%
BP	GO:1902622	regulation of neutrophil migration	22	0.592694731	1.825519365	0.001436544	0.025973727	0.021183	2063	tags=45%, list=16%, signal=38%
MF	GO:0017016	Ras GTPase binding	266	0.341704717	1.409331941	0.001445043	0.025973727	0.021183	4624	tags=50%, list=37%, signal=32%
BP	GO:0019941	modification-dependent protein catabolic process	453	0.312526938	1.308354756	0.001445043	0.025973727	0.021183	4238	tags=42%, list=34%, signal=29%
CC	GO:0097447	dendritic tree	297	0.323898909	1.34059786	0.001445043	0.025973727	0.021183	4617	tags=47%, list=37%, signal=31%
BP	GO:0010634	positive regulation of epithelial cell migration	99	0.398983344	1.551148619	0.001461681	0.026198134	0.021366	4348	tags=49%, list=34%, signal=33%
BP	GO:0002768	immune response-regulating cell surface receptor signaling pathway	254	0.336941855	1.386039408	0.001500267	0.026813557	0.021868	4668	tags=46%, list=37%, signal=30%
BP	GO:0101023	vascular endothelial cell proliferation	13	0.669318108	1.828022091	0.001509079	0.026819102	0.021872	2485	tags=54%, list=20%, signal=43%
BP	GO:1905562	regulation of vascular endothelial cell proliferation	13	0.669318108	1.828022091	0.001509079	0.026819102	0.021872	2485	tags=54%, list=20%, signal=43%
BP	GO:0071622	regulation of granulocyte chemotaxis	26	0.56438437	1.804036735	0.001525035	0.026951738	0.02198	1513	tags=38%, list=12%, signal=34%
BP	GO:0042572	retinol metabolic process	19	-0.4840946	-2.0083022	0.0015251	0.0269517	0.022	2715	tags=63%, list=22%, signal=50%
BP	GO:0031346	positive regulation of cell projection organization	218	0.351629447	1.443205516	0.00154016	0.027142099	0.022136	4063	tags=44%, list=32%, signal=31%
BP	GO:0007059	chromosome segregation	173	0.364045758	1.478946634	1.54E-03	0.02714802	0.02214	5439	tags=54%, list=43%, signal=31%
BP	GO:0046578	regulation of Ras protein signal transduction	122	0.382515184	1.516702268	1.56E-03	0.027319472	0.02228	4286	tags=53%, list=34%, signal=36%
MF	GO:0031491	nucleosome binding	42	0.489131429	1.708898949	1.57E-03	0.027477704	0.022409	4846	tags=67%, list=38%, signal=41%
CC	GO:0048471	perinuclear region of cytoplasm	441	0.313506759	1.311119425	1.59E-03	0.027751008	0.022632	4255	tags=42%, list=34%, signal=29%
CC	GO:0070603	SWI/SNF superfamily-type complex	63	0.43986248	1.620281943	1.61E-03	0.02798858	0.022826	4935	tags=63%, list=39%, signal=39%
BP	GO:0050851	antigen receptor-mediated signaling pathway	160	0.364860339	1.47549539	1.62E-03	0.028001505	0.022836	4668	tags=49%, list=37%, signal=31%
BP	GO:0007163	establishment or maintenance of cell polarity	135	0.377910822	1.50901746	1.65E-03	0.028529795	0.023267	4052	tags=47%, list=32%, signal=33%
BP	GO:0016126	sterol biosynthetic process	55	-0.300308724	-1.619780822	1.68E-03	0.028897508	0.023567	2653	tags=45%, list=21%, signal=36%
BP	GO:0070534	protein K63-linked ubiquitination	43	0.485108733	1.69467478	1.68E-03	0.028907118	0.023575	4224	tags=58%, list=34%, signal=39%
MF	GO:0005178	integrin binding	84	0.418123399	1.599096467	1.69E-03	0.028946167	0.023607	4185	tags=50%, list=33%, signal=34%
BP	GO:0030041	actin filament polymerization	112	0.394465008	1.551309207	0.001693	0.028946167	0.023607	3666	tags=46%, list=29%, signal=33%
BP	GO:0010720	positive regulation of cell development	304	0.328829448	1.361879232	0.001702755	0.029034271	0.023679	4184	tags=44%, list=33%, signal=30%
BP	GO:0043632	modification-dependent macromolecule catabolic process	461	0.30878118	1.292973315	0.001721163	0.029269046	0.02387	4238	tags=41%, list=34%, signal=28%
BP	GO:0010522	regulation of calcium ion transport into cytosol	49	0.46832496	1.67236892	0.001771405	0.030042464	0.024501	2481	tags=39%, list=20%, signal=31%
MF	GO:0017048	Rho GTPase binding	100	0.399737777	1.55655992	0.001816306	0.030680441	0.025021	4151	tags=51%, list=33%, signal=34%
BP	GO:0016101	diterpenoid metabolic process	50	-0.322644038	-1.720026547	0.001818749	0.030680441	0.025021	1181	tags=48%, list=9%, signal=44%
BP	GO:0007608	sensory perception of smell	25	-0.448762506	-2.016637851	0.001827963	0.030753648	0.025081	2221	tags=60%, list=18%, signal=50%
CC	GO:0019897	extrinsic component of plasma membrane	82	0.419801872	1.598475747	0.001833059	0.030757367	0.025084	4063	tags=49%, list=32%, signal=33%
MF	GO:0004842	ubiquitin-protein transferase activity	277	0.334513034	1.380114546	0.001850019	0.030916678	0.025214	5112	tags=53%, list=41%, signal=32%
CC	GO:0000784	nuclear chromosome, telomeric region	65	0.440894512	1.634723727	0.001852354	0.030916678	0.025214	4649	tags=57%, list=37%, signal=36%
MF	GO:0051219	phosphoprotein binding	59	0.448508455	1.641190866	0.001916596	0.031904488	0.026019	4212	tags=54%, list=33%, signal=36%
BP	GO:0001952	regulation of cell-matrix adhesion	75	0.432862565	1.636783368	0.001927023	0.031993647	0.026092	3782	tags=53%, list=30%, signal=38%

BP	GO:0000086	G2/M transition of mitotic cell cycle	171	0.357714256	1.451338703	0.001954135	0.032358622	0.02639	5304	tags=56%, list=42%, signal=33%
BP	GO:0051220	cytoplasmic sequestering of protein	18	0.615473948	1.818200996	0.001986962	0.032697329	0.026666	1839	tags=50%, list=15%, signal=43%
BP	GO:0060828	regulation of canonical Wnt signaling pathway	180	0.352318202	1.434402264	0.001989356	0.032697329	0.026666	5010	tags=53%, list=40%, signal=33%
BP	GO:0120032	regulation of plasma membrane bounded cell projection assembly	99	0.394098623	1.532158031	0.001990137	0.032697329	0.026666	4316	tags=48%, list=34%, signal=32%
CC	GO:0030424	axon	318	0.328146267	1.360792473	0.002016416	0.03304304	0.026948	4640	tags=48%, list=37%, signal=31%
BP	GO:0060491	regulation of cell projection assembly	100	0.397116753	1.546353776	0.002061729	0.033601048	0.027403	4316	tags=49%, list=34%, signal=32%
BP	GO:0045732	positive regulation of protein catabolic process	153	0.370212427	1.494914502	0.002063722	0.033601048	0.027403	3493	tags=42%, list=28%, signal=31%
BP	GO:0007156	homophilic cell adhesion via plasma membrane adhesion molecules	46	0.474377322	1.672021526	0.002066446	0.033601048	0.027403	3595	tags=48%, list=29%, signal=34%
BP	GO:0033044	regulation of chromosome organization	223	0.339898841	1.395747909	0.002131668	0.034548013	0.028175	5024	tags=50%, list=40%, signal=30%
CC	GO:0030863	cortical cytoskeleton	60	0.451826485	1.657713503	0.002140438	0.034548013	0.028175	4445	tags=58%, list=35%, signal=38%
BP	GO:0043534	blood vessel endothelial cell migration	82	0.417270511	1.588837109	0.002141112	0.034548013	0.028175	3323	tags=44%, list=26%, signal=33%
BP	GO:2000209	regulation of anoikis	18	0.612478287	1.809351372	0.002208967	0.035551966	0.028994	3782	tags=61%, list=30%, signal=43%
MF	GO:0009975	cyclase activity	10	0.695651815	1.763882589	0.00223461	0.035839486	0.029229	1533	tags=60%, list=12%, signal=53%
BP	GO:0007266	Rho protein signal transduction	88	0.399543107	1.53548479	0.002238193	0.035839486	0.029229	2731	tags=40%, list=22%, signal=31%
BP	GO:0051701	interaction with host	134	0.381454175	1.52232277	0.002267854	0.036222505	0.029541	4983	tags=57%, list=40%, signal=35%
BP	GO:0008064	regulation of actin polymerization or depolymerization	111	0.387001009	1.522424528	0.002307674	0.036672832	0.029908	3666	tags=45%, list=29%, signal=32%
BP	GO:0030832	regulation of actin filament length	111	0.387001009	1.522424528	0.002307674	0.036672832	0.029908	3666	tags=45%, list=29%, signal=32%
BP	GO:0032535	regulation of cellular component size	221	0.340142509	1.395953203	0.002354097	0.03726631	0.030392	4316	tags=46%, list=34%, signal=31%
BP	GO:0006721	terpenoid metabolic process	56	-0.303431014	-1.636065648	0.002356833	0.03726631	0.030392	2020	tags=48%, list=16%, signal=41%
BP	GO:0046847	filopodium assembly	32	0.52364025	1.735293564	0.002382031	0.037489549	0.030574	3832	tags=62%, list=30%, signal=44%
BP	GO:0045785	positive regulation of cell adhesion	244	0.335887982	1.380924205	0.002388778	0.037489549	0.030574	3833	tags=40%, list=30%, signal=28%
MF	GO:0060589	nucleoside-triphosphatase regulator activity	217	0.349373266	1.434156503	0.002388778	0.037489549	0.030574	4386	tags=45%, list=35%, signal=30%
MF	GO:0017069	snRNA binding	34	0.516212681	1.734147935	0.002407301	0.0376865	0.030735	4811	tags=71%, list=38%, signal=44%
BP	GO:0051656	establishment of organelle localization	268	0.33308135	1.374038282	0.002423389	0.037844457	0.030864	4116	tags=43%, list=33%, signal=29%
BP	GO:0072583	clathrin-dependent endocytosis	30	0.522459733	1.712216744	0.002450872	0.038114954	0.031084	3000	tags=53%, list=24%, signal=41%
BP	GO:0043123	positive regulation of I-kappaB kinase/NF-kappaB signaling	132	0.382138148	1.523309828	0.002452793	0.038114954	0.031084	4406	tags=52%, list=35%, signal=34%
MF	GO:0042826	histone deacetylase binding	85	0.406011988	1.554108901	0.002483137	0.03842308	0.031336	5348	tags=66%, list=42%, signal=38%
BP	GO:0072665	protein localization to vacuole	51	0.451618137	1.612070975	0.002494298	0.03842308	0.031336	3060	tags=45%, list=24%, signal=34%
BP	GO:0010608	posttranscriptional regulation of gene expression	436	0.307842425	1.286548111	0.002497384	0.03842308	0.031336	4681	tags=46%, list=37%, signal=30%
BP	GO:0071624	positive regulation of granulocyte chemotaxis	14	0.646538695	1.798033381	0.002512796	0.03842308	0.031336	1501	tags=43%, list=12%, signal=38%
BP	GO:0090023	positive regulation of neutrophil chemotaxis	14	0.646538695	1.798033381	0.002512796	0.03842308	0.031336	1501	tags=43%, list=12%, signal=38%
BP	GO:0048525	negative regulation of viral process	64	0.434035754	1.605777286	0.002519808	0.03842308	0.031336	2810	tags=45%, list=22%, signal=35%
BP	GO:0010742	macrophage derived foam cell differentiation	21	0.567571523	1.730325102	0.00252614	0.03842308	0.031336	2810	tags=52%, list=22%, signal=41%
BP	GO:0090077	foam cell differentiation	21	0.567571523	1.730325102	0.00252614	0.03842308	0.031336	2810	tags=52%, list=22%, signal=41%
BP	GO:0042176	regulation of protein catabolic process	271	0.329194844	1.35795695	0.002534382	0.03842308	0.031336	4247	tags=44%, list=34%, signal=30%
BP	GO:0060322	head development	427	0.30937478	1.291922387	0.002534382	0.03842308	0.031336	4371	tags=42%, list=35%, signal=29%
BP	GO:0090504	epiboly	22	0.579269769	1.784170039	0.002539942	0.03842308	0.031336	3447	tags=73%, list=27%, signal=53%
BP	GO:0050770	regulation of axonogenesis	109	0.387848595	1.520977521	0.002545704	0.03842308	0.031336	4187	tags=53%, list=33%, signal=36%
MF	GO:0031625	ubiquitin protein ligase binding	222	0.335378804	1.377584551	0.002573951	0.038709806	0.03157	4887	tags=49%, list=39%, signal=31%

MF	GO:0008378	galactosyltransferase activity	13	0.649926986	1.775061625	0.002576972	0.038709806	0.03157	2716	tags=69%, list=22%, signal=54%
BP	GO:0046822	regulation of nucleocytoplasmic transport	81	0.414953872	1.579589452	0.002601333	0.038982929	0.031792	4574	tags=56%, list=36%, signal=36%
CC	GO:0030425	dendrite	296	0.324648539	1.343642248	0.002608377	0.038995854	0.031803	4617	tags=47%, list=37%, signal=31%
BP	GO:0010959	regulation of metal ion transport	181	0.347944019	1.41743599	0.002618842	0.039041446	0.03184	2973	tags=33%, list=24%, signal=26%
BP	GO:0052126	movement in host environment	104	0.393384004	1.536685099	0.002623803	0.039041446	0.03184	4110	tags=50%, list=33%, signal=34%
BP	GO:0061045	negative regulation of wound healing	39	0.50155749	1.727768529	0.002656969	0.039441917	0.032167	3187	tags=51%, list=25%, signal=38%
CC	GO:0030426	growth cone	110	0.388097179	1.525841459	0.002682898	0.039672154	0.032354	4552	tags=55%, list=36%, signal=36%
BP	GO:0050673	epithelial cell proliferation	224	0.337582833	1.386295318	0.002685055	0.039672154	0.032354	3531	tags=38%, list=28%, signal=27%
BP	GO:0002764	immune response-regulating signaling pathway	256	0.331952368	1.365462167	0.00271937	0.039846082	0.032496	4668	tags=46%, list=37%, signal=30%
CC	GO:0031012	extracellular matrix	255	0.332626208	1.368154864	0.00271937	0.039846082	0.032496	3543	tags=38%, list=28%, signal=28%
BP	GO:0051099	positive regulation of binding	123	0.377162373	1.494839814	0.00272042	0.039846082	0.032496	3522	tags=44%, list=28%, signal=32%
BP	GO:0008015	blood circulation	239	0.329721543	1.355458414	0.002722089	0.039846082	0.032496	3331	tags=36%, list=26%, signal=27%
BP	GO:0002253	activation of immune response	274	0.325964906	1.344701225	0.002756368	0.04000504	0.032626	4624	tags=46%, list=37%, signal=30%
BP	GO:0006511	ubiquitin-dependent protein catabolic process	447	0.309767494	1.29561383	0.002756368	0.04000504	0.032626	4238	tags=41%, list=34%, signal=28%
BP	GO:0070925	organelle assembly	488	0.30010915	1.259700619	0.002756368	0.04000504	0.032626	5418	tags=51%, list=43%, signal=31%
BP	GO:0015701	bicarbonate transport	19	-0.466795798	-1.936536726	0.002761617	0.04000504	0.032626	607	tags=47%, list=5%, signal=45%
BP	GO:0018108	peptidyl-tyrosine phosphorylation	203	0.344760429	1.410920574	0.002764653	0.04000504	0.032626	3002	tags=33%, list=24%, signal=26%
BP	GO:0042339	keratan sulfate metabolic process	23	0.559785275	1.744223896	0.002804407	0.040487427	0.033019	2034	tags=43%, list=16%, signal=37%
BP	GO:0000082	G1/S transition of mitotic cell cycle	168	0.352641551	1.430576093	0.002916011	0.042002537	0.034255	5178	tags=55%, list=41%, signal=33%
BP	GO:0043488	regulation of mRNA stability	137	0.367985435	1.471149908	0.002936629	0.042203171	0.034419	4784	tags=53%, list=38%, signal=33%
BP	GO:0045787	positive regulation of cell cycle	221	0.336932288	1.382778378	0.002984316	0.042791027	0.034898	5425	tags=56%, list=43%, signal=32%
BP	GO:0009100	glycoprotein metabolic process	231	0.336729505	1.381784132	0.003021388	0.043224349	0.035251	3905	tags=40%, list=31%, signal=28%
BP	GO:0044070	regulation of anion transport	55	-0.286663072	-1.546180009	0.003090058	0.044074069	0.035944	1478	tags=31%, list=12%, signal=27%
BP	GO:0048285	organelle fission	229	0.335762298	1.378694022	0.003095532	0.044074069	0.035944	5258	tags=52%, list=42%, signal=31%
BP	GO:1904062	regulation of cation transmembrane transport	156	0.367728591	1.485851782	0.003101741	0.044074069	0.035944	2481	tags=33%, list=20%, signal=27%
BP	GO:0051090	regulation of DNA-binding transcription factor activity	256	0.330897422	1.361122719	0.003126345	0.044323838	0.036148	3522	tags=37%, list=28%, signal=27%
BP	GO:0006261	DNA-dependent DNA replication	88	0.393472349	1.512154247	0.003232924	0.045732109	0.037297	4441	tags=53%, list=35%, signal=35%
BP	GO:0050678	regulation of epithelial cell proliferation	191	0.344878893	1.40709262	0.003247069	0.045755387	0.037316	3373	tags=37%, list=27%, signal=27%
BP	GO:0050654	chondroitin sulfate proteoglycan metabolic process	28	0.536446389	1.736713338	0.003249075	0.045755387	0.037316	3119	tags=54%, list=25%, signal=40%
BP	GO:2000811	negative regulation of anoikis	15	0.649235453	1.837424195	0.003258561	0.04578678	0.037341	3782	tags=73%, list=30%, signal=51%
MF	GO:0003697	single-stranded DNA binding	71	0.411963591	1.554858866	0.003274566	0.04590942	0.037441	5697	tags=63%, list=45%, signal=35%
BP	GO:0034504	protein localization to nucleus	185	0.347410068	1.415948306	0.003321287	0.046360623	0.037809	5149	tags=55%, list=41%, signal=33%
BP	GO:0050690	regulation of defense response to virus by virus	22	0.57215958	1.762270423	0.003321446	0.046360623	0.037809	1944	tags=45%, list=15%, signal=39%
BP	GO:0051147	regulation of muscle cell differentiation	85	0.402086903	1.53908469	0.003330084	0.046378581	0.037824	4157	tags=51%, list=33%, signal=34%
BP	GO:0006400	tRNA modification	58	0.440945049	1.612508801	0.0033508	0.046564306	0.037975	4632	tags=55%, list=37%, signal=35%
CC	GO:0000793	condensed chromosome	115	0.377890764	1.490185737	0.003361709	0.046613229	0.038015	5994	tags=62%, list=48%, signal=33%
BP	GO:0032273	positive regulation of protein polymerization	82	0.4066267	1.548308768	0.003414177	0.047236935	0.038524	4045	tags=49%, list=32%, signal=33%
CC	GO:0042641	actomyosin	58	0.440531463	1.610996341	0.00342918	0.047340693	0.038608	4663	tags=60%, list=37%, signal=38%
BP	GO:1902749	regulation of cell cycle G2/M phase transition	142	0.355884525	1.42740005	0.003453376	0.04757063	0.038796	5304	tags=56%, list=42%, signal=33%

BP	GO:0051650	establishment of vesicle localization	138	0.365368814	1.459860588	0.003501298	0.048125683	0.039249	4116	tags=47%, list=33%, signal=32%
CC	GO:0031256	leading edge membrane	98	0.392567301	1.522501994	0.003555219	0.048738643	0.039748	3004	tags=37%, list=24%, signal=28%
CC	GO:0036477	somatodendritic compartment	410	0.305703619	1.276686066	0.003570318	0.048738643	0.039748	4116	tags=40%, list=33%, signal=28%
BP	GO:0003197	endocardial cushion development	25	0.565802798	1.787953146	0.003570942	0.048738643	0.039748	3153	tags=60%, list=25%, signal=45%
MF	GO:0000049	tRNA binding	36	0.500505036	1.698732988	0.003576794	0.048738643	0.039748	4657	tags=67%, list=37%, signal=42%
BP	GO:0003203	endocardial cushion morphogenesis	19	0.580645528	1.735644994	0.003610487	0.04907255	0.040021	2072	tags=53%, list=16%, signal=44%
BP	GO:0150117	positive regulation of cell-substrate junction organization	23	0.551790609	1.719313474	0.003617885	0.04907255	0.040021	2965	tags=61%, list=24%, signal=47%
BP	GO:0006399	tRNA metabolic process	117	0.373861816	1.476486728	0.003624633	0.04907255	0.040021	4784	tags=49%, list=38%, signal=31%
BP	GO:0019395	fatty acid oxidation	66	-0.273115992	-1.536178072	0.003643198	0.049218279	0.04014	2679	tags=41%, list=21%, signal=32%
MF	GO:0046875	ephrin receptor binding	22	0.569178014	1.753087099	0.003712199	0.049864785	0.040667	4116	tags=73%, list=33%, signal=49%
BP	GO:0038093	Fc receptor signaling pathway	143	0.358761351	1.439108598	0.00371471	0.049864785	0.040667	4668	tags=50%, list=37%, signal=32%
BP	GO:0003205	cardiac chamber development	90	0.397221572	1.530855411	0.003722601	0.049864785	0.040667	4404	tags=54%, list=35%, signal=36%
BP	GO:0050852	T cell receptor signaling pathway	136	0.365416085	1.45997152	0.003729749	0.049864785	0.040667	4668	tags=50%, list=37%, signal=32%
BP	GO:0031532	actin cytoskeleton reorganization	69	0.417444159	1.565342512	0.003730572	0.049864785	0.040667	4256	tags=55%, list=34%, signal=37%
BP	GO:2001257	regulation of cation channel activity	63	0.423252825	1.559098449	0.003746666	0.049974036	0.040756	2410	tags=35%, list=19%, signal=28%
**KEGG**										
	**ID**	**Description**	**setSize**	**enrichmentScore**	**NES**	**pvalue**	**p.adjust**	**qvalues**	**rank**	**leading_edge**
	hsa05200	Pathways in cancer	316	0.391241393	1.636950294	1.44378E-07	2.28975E-05	1.44E-05	3813	tags=45%, list=30%, signal=32%
	hsa04144	Endocytosis	175	0.433375664	1.763193036	1.46779E-07	2.28975E-05	1.44E-05	3927	tags=57%, list=31%, signal=39%
	hsa00280	Valine, leucine and isoleucine degradation	38	-0.52049183	-2.548747749	8.03695E-07	8.35843E-05	5.25E-05	1931	tags=37%, list=15%, signal=31%
	hsa04510	Focal adhesion	136	0.439737149	1.758647272	1.92428E-06	0.000150094	9.42E-05	4151	tags=54%, list=33%, signal=36%
	hsa04151	PI3K-Akt signaling pathway	200	0.39875769	1.635209389	7.16261E-06	0.000446947	0.00028	4034	tags=48%, list=32%, signal=33%
	hsa00640	Propanoate metabolism	28	-0.554295019	-2.527733131	9.27865E-06	0.000456535	0.000286	2609	tags=50%, list=21%, signal=40%
	hsa05132	Salmonella infection	187	0.409943777	1.676286164	1.02428E-05	0.000456535	0.000286	4665	tags=58%, list=37%, signal=37%
	hsa03040	Spliceosome	102	0.461336	1.80230165	1.52758E-05	0.000595756	0.000374	4538	tags=58%, list=36%, signal=37%
	hsa05100	Bacterial invasion of epithelial cells	53	0.53220012	1.946768497	1.77644E-05	0.000602232	0.000378	4332	tags=72%, list=34%, signal=47%
	hsa04810	Regulation of actin cytoskeleton	142	0.41478026	1.661737809	1.93023E-05	0.000602232	0.000378	4151	tags=51%, list=33%, signal=35%
	hsa05135	Yersinia infection	99	0.452183872	1.760048809	2.6099E-05	0.000740262	0.000465	2973	tags=47%, list=24%, signal=37%
	hsa04072	Phospholipase D signaling pathway	89	0.459588651	1.769641396	3.67035E-05	0.000901211	0.000566	3538	tags=47%, list=28%, signal=34%
	hsa05160	Hepatitis C	106	0.438783905	1.720170107	3.75505E-05	0.000901211	0.000566	3710	tags=51%, list=29%, signal=36%
	hsa05222	Small cell lung cancer	70	0.487292912	1.839371976	4.35091E-05	0.000969632	0.000608	4360	tags=60%, list=35%, signal=39%
	hsa04540	Gap junction	53	0.509275162	1.862909844	8.00637E-05	0.001665326	0.001045	3401	tags=55%, list=27%, signal=40%
	hsa04935	Growth hormone synthesis, secretion and action	75	0.461637445	1.751271915	9.98406E-05	0.001946892	0.001222	2810	tags=39%, list=22%, signal=30%
	hsa00650	Butanoate metabolism	12	-0.730824588	-2.523722065	0.000113857	0.00208962	0.001311	3047	tags=83%, list=24%, signal=63%
	hsa05165	Human papillomavirus infection	216	0.371576147	1.526121261	0.00012596	0.00218331	0.00137	3915	tags=47%, list=31%, signal=33%
	hsa05203	Viral carcinogenesis	127	0.411060707	1.638706839	0.000153809	0.002525712	0.001585	4803	tags=57%, list=38%, signal=36%
	hsa05130	Pathogenic Escherichia coli infection	133	0.405240833	1.617371009	0.000199783	0.003116614	0.001956	4366	tags=56%, list=35%, signal=37%
	hsa05211	Renal cell carcinoma	49	0.498738703	1.798536182	0.000219907	0.003267184	0.00205	3516	tags=53%, list=28%, signal=38%
	hsa05145	Toxoplasmosis	72	0.460209901	1.741077936	0.00025762	0.003653515	0.002293	3887	tags=49%, list=31%, signal=34%

hsa04210	Apoptosis	96	0.426364929	1.658748719	0.000285094	0.003867366	0.002427	4366	tags=52%, list=35%, signal=34%
hsa01522	Endocrine resistance	61	0.475177852	1.761137687	0.000299784	0.003897186	0.002446	2604	tags=43%, list=21%, signal=34%
hsa05214	Glioma	49	0.490802784	1.769917917	0.000324775	0.004053192	0.002544	2604	tags=45%, list=21%, signal=36%
hsa05163	Human cytomegalovirus infection	146	0.391045638	1.567879163	0.000360238	0.00432286	0.002713	3782	tags=46%, list=30%, signal=33%
hsa00330	Arginine and proline metabolism	30	-0.449036571	-2.013970779	0.000458467	0.005297845	0.003325	2280	tags=47%, list=18%, signal=38%
hsa04926	Relaxin signaling pathway	75	0.440842492	1.672383994	0.00047879	0.005308885	0.003331	3782	tags=47%, list=30%, signal=33%
hsa04015	Rap1 signaling pathway	126	0.396645676	1.579602516	0.000493454	0.005308885	0.003331	2973	tags=39%, list=24%, signal=30%
hsa05166	Human T-cell leukemia virus 1 infection	153	0.387818274	1.558875591	0.000510873	0.005313074	0.003334	4574	tags=48%, list=36%, signal=31%
hsa04071	Sphingolipid signaling pathway	78	0.441923439	1.680571241	0.000535674	0.005391301	0.003383	4366	tags=55%, list=35%, signal=36%
hsa05169	Epstein-Barr virus infection	144	0.379962301	1.525037442	0.000640381	0.006054837	0.0038	4749	tags=53%, list=38%, signal=33%
hsa05146	Amoebiasis	53	0.476972255	1.744746997	0.000640415	0.006054837	0.0038	2751	tags=40%, list=22%, signal=31%
hsa05220	Chronic myeloid leukemia	56	0.467916636	1.72209679	0.0006931	0.006156078	0.003863	3684	tags=48%, list=29%, signal=34%
hsa05164	Influenza A	107	0.410560772	1.609746338	0.000743733	0.006156078	0.003863	4574	tags=52%, list=36%, signal=34%
hsa05225	Hepatocellular carcinoma	103	0.408061716	1.593460593	0.000746017	0.006156078	0.003863	3813	tags=47%, list=30%, signal=33%
hsa04670	Leukocyte transendothelial migration	70	0.448734528	1.693826642	0.000748119	0.006156078	0.003863	3585	tags=51%, list=28%, signal=37%
hsa04914	Progesterone-mediated oocyte maturation	53	0.474825639	1.736894754	0.000755977	0.006156078	0.003863	3932	tags=49%, list=31%, signal=34%
hsa04371	Apelin signaling pathway	79	0.438566148	1.672705289	0.00076951	0.006156078	0.003863	3795	tags=51%, list=30%, signal=36%
hsa04666	Fc gamma R-mediated phagocytosis	73	0.437192972	1.653673068	0.000890144	0.006943126	0.004357	4574	tags=60%, list=36%, signal=39%
hsa00620	Pyruvate metabolism	32	-0.422160845	-1.962860771	0.000984009	0.007488067	0.004699	2617	tags=53%, list=21%, signal=42%
hsa05131	Shigellosis	167	0.370456414	1.497870199	0.001692802	0.012575104	0.007891	4478	tags=51%, list=36%, signal=34%
hsa04014	Ras signaling pathway	123	0.377646583	1.497891296	0.001924651	0.013964912	0.008763	4358	tags=48%, list=35%, signal=32%
hsa00531	Glycosaminoglycan degradation	15	0.640380779	1.824687437	0.002023792	0.014015893	0.008795	2150	tags=53%, list=17%, signal=44%
hsa00520	Amino sugar and nucleotide sugar metabolism	36	-0.38062608	-1.831563585	0.002040167	0.014015893	0.008795	1417	tags=28%, list=11%, signal=25%
hsa05414	Dilated cardiomyopathy	46	0.465131355	1.65669774	0.002066446	0.014015893	0.008795	2527	tags=43%, list=20%, signal=35%
hsa00230	Purine metabolism	71	0.432932117	1.63713151	0.002326728	0.015445517	0.009693	1533	tags=32%, list=12%, signal=29%
hsa05168	Herpes simplex virus 1 infection	265	0.327453967	1.361450653	0.002645375	0.017125411	0.010747	4501	tags=42%, list=36%, signal=27%
hsa00071	Fatty acid degradation	30	-0.399851873	-1.793372832	0.002689568	0.017125411	0.010747	2626	tags=43%, list=21%, signal=34%
hsa05161	Hepatitis B	101	0.38975545	1.523319828	0.003070566	0.019160334	0.012024	3798	tags=44%, list=30%, signal=31%
hsa04115	p53 signaling pathway	47	0.46970258	1.679858881	0.003372275	0.020481841	0.012853	4467	tags=60%, list=35%, signal=39%
hsa03013	Nucleocytoplasmic transport	83	0.402214874	1.539275736	0.00341364	0.020481841	0.012853	5757	tags=69%, list=46%, signal=38%
hsa05167	Kaposi sarcoma-associated herpesvirus infection	128	0.370950883	1.478362265	0.003740731	0.022020905	0.013819	4370	tags=51%, list=35%, signal=34%
hsa00380	Tryptophan metabolism	24	-0.435272977	-1.893269463	0.003868797	0.022353052	0.014027	1931	tags=50%, list=15%, signal=42%
hsa04270	Vascular smooth muscle contraction	78	0.407389093	1.549242093	0.004272218	0.023867129	0.014977	3596	tags=46%, list=29%, signal=33%
hsa04512	ECM-receptor interaction	51	0.434819279	1.588098282	0.004283844	0.023867129	0.014977	3181	tags=45%, list=25%, signal=34%
hsa05224	Breast cancer	78	0.40670609	1.546644732	0.004426167	0.024227443	0.015203	3092	tags=38%, list=25%, signal=29%
hsa04934	Cushing syndrome	84	0.40001034	1.532179561	0.004565952	0.024460915	0.01535	3813	tags=45%, list=30%, signal=32%
hsa04530	Tight junction	108	0.379753823	1.489568405	0.004671268	0.024460915	0.01535	4059	tags=49%, list=32%, signal=34%
hsa05206	MicroRNAs in cancer	116	0.373743176	1.477834942	0.004704022	0.024460915	0.01535	3153	tags=41%, list=25%, signal=31%
hsa05142	Chagas disease	59	0.430705677	1.594014144	0.00485624	0.024838473	0.015587	3566	tags=46%, list=28%, signal=33%

	hsa01200	Carbon metabolism	84	-0.239170132	-1.407887156	0.005071004	0.025518602	0.016014	1206	tags=21%, list=10%, signal=20%
	hsa05170	Human immunodeficiency virus 1 infection	128	0.365092096	1.455013055	0.005423746	0.026860458	0.016856	4370	tags=50%, list=35%, signal=33%
	hsa05215	Prostate cancer	67	0.419313084	1.570399694	0.006352402	0.030707416	0.01927	3932	tags=51%, list=31%, signal=35%
	hsa05162	Measles	87	0.397288811	1.529548394	0.006444518	0.030707416	0.01927	4657	tags=52%, list=37%, signal=33%
	hsa05213	Endometrial cancer	40	0.467473447	1.627458932	0.0064958	0.030707416	0.01927	3678	tags=52%, list=29%, signal=37%
	hsa05323	Rheumatoid arthritis	46	-0.309944287	-1.611179051	0.006905069	0.032154947	0.020178	3061	tags=54%, list=24%, signal=41%
	hsa04110	Cell cycle	83	0.390347544	1.493859483	0.007014311	0.03218331	0.020196	4467	tags=49%, list=35%, signal=32%
	hsa05032	Morphine addiction	36	0.483114105	1.653450526	0.00741901	0.033546827	0.021052	4087	tags=56%, list=32%, signal=38%
	hsa04010	MAPK signaling pathway	169	0.345140163	1.397566788	0.007886002	0.035149035	0.022057	2900	tags=36%, list=23%, signal=28%
	hsa04062	Chemokine signaling pathway	103	0.36979488	1.444030513	0.008345615	0.036673689	0.023014	4262	tags=45%, list=34%, signal=30%
	hsa00534	Glycosaminoglycan biosynthesis - heparan sulfate / heparin	14	0.607149828	1.702019952	0.008846149	0.03833331	0.024055	2472	tags=57%, list=20%, signal=46%
	hsa04330	Notch signaling pathway	44	0.445745115	1.572394096	0.009544257	0.040791895	0.025598	5472	tags=70%, list=43%, signal=40%
	hsa05212	Pancreatic cancer	56	0.418722942	1.541046797	0.010062266	0.042424688	0.026623	3498	tags=48%, list=28%, signal=35%
	hsa00533	Glycosaminoglycan biosynthesis - keratan sulfate	11	0.65224092	1.698881849	0.011057172	0.045749478	0.028709	2034	tags=55%, list=16%, signal=46%
	hsa00220	Arginine biosynthesis	10	-0.593939354	-1.897378022	0.011144104	0.045749478	0.028709	2084	tags=60%, list=17%, signal=50%
	hsa04919	Thyroid hormone signaling pathway	84	0.382594292	1.465470001	0.011490479	0.04610355	0.028931	5144	tags=52%, list=41%, signal=31%
	hsa04621	NOD-like receptor signaling pathway	108	0.364261376	1.428799932	0.011525888	0.04610355	0.028931	4501	tags=49%, list=36%, signal=32%
	hsa05218	Melanoma	41	0.445123137	1.556336615	0.012467293	0.049237917	0.030898	2604	tags=39%, list=21%, signal=31%
**TEME2**										
**GO**										
**ONTOLOGY**	**ID**	**Description**	**setSize**	**enrichmentScore**	**NES**	**pvalue**	**p.adjust**	**qvalues**	**rank**	**leading_edge**
BP	GO:0000904	cell morphogenesis involved in differentiation	425	0.401067047	1.665527051	1.00E-10	5.73E-08	4.24E-08	3174	tags=41%, list=25%, signal=32%
BP	GO:0001525	angiogenesis	309	0.447095183	1.837041856	1.00E-10	5.73E-08	4.24E-08	2877	tags=41%, list=23%, signal=32%
BP	GO:0001568	blood vessel development	412	0.422659233	1.754109779	1.00E-10	5.73E-08	4.24E-08	3406	tags=43%, list=27%, signal=32%
BP	GO:0001944	vasculature development	436	0.414380022	1.722810453	1.00E-10	5.73E-08	4.24E-08	3406	tags=42%, list=27%, signal=32%
BP	GO:0003158	endothelium development	80	0.611273466	2.309402918	1.00E-10	5.73E-08	4.24E-08	2386	tags=59%, list=19%, signal=48%
BP	GO:0006614	SRP-dependent cotranslational protein targeting to membrane	90	-0.439584583	-2.603664246	1.00E-10	5.73E-08	4.24E-08	2565	tags=47%, list=20%, signal=37%
BP	GO:0007264	small GTPase mediated signal transduction	327	0.434950049	1.794843619	1.00E-10	5.73E-08	4.24E-08	3488	tags=47%, list=28%, signal=35%
BP	GO:0045047	protein targeting to ER	102	-0.404713905	-2.600378412	1.00E-10	5.73E-08	4.24E-08	2261	tags=44%, list=18%, signal=37%
BP	GO:0045446	endothelial cell differentiation	67	0.633304205	2.361500749	1.00E-10	5.73E-08	4.24E-08	2386	tags=61%, list=19%, signal=50%
BP	GO:0048514	blood vessel morphogenesis	358	0.431389394	1.7856738	1.00E-10	5.73E-08	4.24E-08	3406	tags=44%, list=27%, signal=33%
BP	GO:0051301	cell division	330	0.41989954	1.73265413	1.00E-10	5.73E-08	4.24E-08	3820	tags=49%, list=30%, signal=35%
BP	GO:0010564	regulation of cell cycle process	470	0.387593083	1.613696381	1.54E-10	7.56E-08	5.59E-08	4866	tags=53%, list=39%, signal=34%
BP	GO:0035239	tube morphogenesis	491	0.383501209	1.598908985	1.56E-10	7.56E-08	5.59E-08	4342	tags=48%, list=34%, signal=33%
BP	GO:0006613	cotranslational protein targeting to membrane	94	-0.412221786	-2.574080374	2.38E-10	1.07E-07	7.91E-08	2565	tags=45%, list=20%, signal=36%
BP	GO:0072599	establishment of protein localization to endoplasmic reticulum	105	-0.387882076	-2.459005505	3.29E-10	1.3827E-07	1.02E-07	2261	tags=40%, list=18%, signal=33%
BP	GO:0007265	Ras protein signal transduction	223	0.446534268	1.816259249	4.00E-10	1.57538E-07	1.16E-07	3228	tags=48%, list=26%, signal=36%
BP	GO:0044772	mitotic cell cycle phase transition	379	0.399695515	1.657363209	4.95E-10	1.83503E-07	1.36E-07	4576	tags=52%, list=36%, signal=34%
CC	GO:0022626	cytosolic ribosome	98	-0.394940058	-2.512409882	7.55E-10	2.6447E-07	1.96E-07	2717	tags=46%, list=22%, signal=36%

BP	GO:0001667	ameboidal-type cell migration	267	0.430260971	1.757956584	8.83E-10	2.93036E-07	2.17E-07	2877	tags=41%, list=23%, signal=32%
BP	GO:0051056	regulation of small GTPase mediated signal transduction	202	0.451435586	1.827397453	9.63E-10	3.03767E-07	2.25E-07	3463	tags=49%, list=27%, signal=36%
BP	GO:0044770	cell cycle phase transition	399	0.395487327	1.640587986	1.15E-09	3.46336E-07	2.56E-07	4576	tags=51%, list=36%, signal=34%
BP	GO:0090130	tissue migration	198	0.450821871	1.824638921	3.09E-09	8.8529E-07	6.55E-07	3312	tags=45%, list=26%, signal=34%
BP	GO:0032990	cell part morphogenesis	392	0.386400653	1.602754765	4.14E-09	1.13645E-06	8.4E-07	3174	tags=39%, list=25%, signal=30%
BP	GO:0048667	cell morphogenesis involved in neuron differentiation	331	0.398298708	1.644512302	4.34E-09	1.13975E-06	8.43E-07	3174	tags=41%, list=25%, signal=32%
BP	GO:0010631	epithelial cell migration	196	0.453151734	1.833316116	5.18E-09	1.25721E-06	9.29E-07	2937	tags=43%, list=23%, signal=33%
BP	GO:0090132	epithelium migration	196	0.453151734	1.833316116	5.18E-09	1.25721E-06	9.29E-07	2937	tags=43%, list=23%, signal=33%
BP	GO:0002040	sprouting angiogenesis	81	0.547256316	2.06976971	8.10E-09	1.89113E-06	1.4E-06	2772	tags=53%, list=22%, signal=42%
BP	GO:0071363	cellular response to growth factor stimulus	399	0.383005257	1.588808996	1.80E-08	4.06334E-06	3E-06	3443	tags=40%, list=27%, signal=30%
BP	GO:0030036	actin cytoskeleton organization	422	0.379790312	1.577282051	2.22E-08	4.8264E-06	3.57E-06	4433	tags=49%, list=35%, signal=33%
MF	GO:0019900	kinase binding	469	0.368590594	1.534352708	2.90E-08	6.08695E-06	4.5E-06	4295	tags=48%, list=34%, signal=33%
BP	GO:0031589	cell-substrate adhesion	218	0.431627006	1.754708386	3.16E-08	6.42127E-06	4.75E-06	3090	tags=42%, list=25%, signal=32%
BP	GO:0006397	mRNA processing	341	0.391915311	1.619177935	3.26E-08	6.42624E-06	4.75E-06	5511	tags=62%, list=44%, signal=36%
BP	GO:0043542	endothelial cell migration	145	0.465186551	1.852002659	3.59E-08	6.85712E-06	5.07E-06	2937	tags=44%, list=23%, signal=34%
BP	GO:0048812	neuron projection morphogenesis	362	0.385581815	1.595961516	3.73E-08	6.91915E-06	5.12E-06	3174	tags=40%, list=25%, signal=30%
BP	GO:0000375	RNA splicing, via transesterification reactions	250	0.415448745	1.695865585	5.14E-08	9.00239E-06	6.66E-06	5358	tags=64%, list=43%, signal=38%
MF	GO:0016462	pyrophosphatase activity	495	0.363128298	1.515299112	5.14E-08	9.00239E-06	6.66E-06	4141	tags=45%, list=33%, signal=32%
BP	GO:0048858	cell projection morphogenesis	377	0.386325706	1.602157151	6.03E-08	1.02769E-05	7.6E-06	3174	tags=40%, list=25%, signal=30%
MF	GO:0008134	transcription factor binding	425	0.369054045	1.532585385	6.34E-08	1.05285E-05	7.78E-06	5180	tags=55%, list=41%, signal=33%
BP	GO:0070848	response to growth factor	418	0.375780544	1.559986416	7.08E-08	1.13472E-05	8.39E-06	3443	tags=40%, list=27%, signal=30%
BP	GO:0040017	positive regulation of locomotion	314	0.394568154	1.622808988	7.29E-08	1.13472E-05	8.39E-06	2878	tags=38%, list=23%, signal=30%
MF	GO:0016817	hydrolase activity, acting on acid anhydrides	498	0.361691638	1.509160265	7.81E-08	1.13472E-05	8.39E-06	4141	tags=45%, list=33%, signal=32%
MF	GO:0016818	hydrolase activity, acting on acid anhydrides, in phosphorus-containing anhydrides	498	0.361691638	1.509160265	7.81E-08	1.13472E-05	8.39E-06	4141	tags=45%, list=33%, signal=32%
BP	GO:0000377	RNA splicing, via transesterification reactions with bulged adenosine as nucleophile	247	0.413407742	1.687954066	7.92E-08	1.13472E-05	8.39E-06	5358	tags=64%, list=43%, signal=37%
BP	GO:0000398	mRNA splicing, via spliceosome	247	0.413407742	1.687954066	7.92E-08	1.13472E-05	8.39E-06	5358	tags=64%, list=43%, signal=37%
BP	GO:0030029	actin filament-based process	462	0.369050049	1.535975858	8.44E-08	1.17164E-05	8.66E-06	3279	tags=39%, list=26%, signal=30%
BP	GO:0120039	plasma membrane bounded cell projection morphogenesis	374	0.381466172	1.581100567	8.55E-08	1.17164E-05	8.66E-06	3174	tags=39%, list=25%, signal=30%
BP	GO:0030155	regulation of cell adhesion	414	0.373154612	1.54912245	9.18E-08	1.23113E-05	9.1E-06	2910	tags=36%, list=23%, signal=29%
MF	GO:0017111	nucleoside-triphosphatase activity	464	0.365615008	1.52139826	1.09E-07	1.42605E-05	1.05E-05	4141	tags=46%, list=33%, signal=32%
BP	GO:0045601	regulation of endothelial cell differentiation	24	0.724311237	2.269714565	1.23E-07	1.58388E-05	1.17E-05	1814	tags=58%, list=14%, signal=50%
BP	GO:0007346	regulation of mitotic cell cycle	407	0.373028462	1.547944217	1.59E-07	2.00697E-05	1.48E-05	4576	tags=49%, list=36%, signal=32%
BP	GO:2000147	positive regulation of cell motility	307	0.388537424	1.596668827	1.74E-07	2.15004E-05	1.59E-05	2878	tags=37%, list=23%, signal=29%
BP	GO:0003197	endocardial cushion development	25	0.716870766	2.268147076	1.83E-07	2.2193E-05	1.64E-05	1515	tags=60%, list=12%, signal=53%
BP	GO:0030335	positive regulation of cell migration	300	0.396885275	1.629076678	2.18E-07	2.59578E-05	1.92E-05	2878	tags=38%, list=23%, signal=30%
BP	GO:0007409	axonogenesis	262	0.404387956	1.651393305	2.31E-07	2.69882E-05	2E-05	3174	tags=41%, list=25%, signal=31%
BP	GO:0061564	axon development	287	0.393653967	1.613101129	2.44E-07	2.78457E-05	2.06E-05	3174	tags=40%, list=25%, signal=31%
BP	GO:1901342	regulation of vasculature development	207	0.425525126	1.724832993	2.48E-07	2.78457E-05	2.06E-05	2690	tags=38%, list=21%, signal=30%
CC	GO:0005815	microtubule organizing center	422	0.36973405	1.535518055	2.52E-07	2.78457E-05	2.06E-05	4776	tags=51%, list=38%, signal=33%

BP	GO:0006325	chromatin organization	440	0.364638373	1.515905458	2.56E-07	2.78457E-05	2.06E-05	5195	tags=55%, list=41%, signal=34%
BP	GO:0032989	cellular component morphogenesis	435	0.367473124	1.527750373	2.61E-07	2.78457E-05	2.06E-05	3174	tags=38%, list=25%, signal=29%
BP	GO:0006403	RNA localization	174	0.438329378	1.759301574	2.78E-07	2.92209E-05	2.16E-05	4966	tags=60%, list=39%, signal=37%
BP	GO:0051272	positive regulation of cellular component movement	316	0.387703865	1.594459942	4.09E-07	4.226E-05	3.12E-05	2878	tags=37%, list=23%, signal=29%
BP	GO:0072329	monocarboxylic acid catabolic process	80	-0.372540302	-2.202842732	4.50E-07	4.57265E-05	3.38E-05	2542	tags=44%, list=20%, signal=35%
BP	GO:0001935	endothelial cell proliferation	85	0.510937505	1.939473334	4.84E-07	4.84616E-05	3.58E-05	2598	tags=46%, list=21%, signal=37%
BP	GO:0009062	fatty acid catabolic process	70	-0.39779771	-2.230714508	5.61E-07	5.53235E-05	4.09E-05	2542	tags=44%, list=20%, signal=36%
CC	GO:0005813	centrosome	335	0.376510983	1.554772671	5.75E-07	5.57563E-05	4.12E-05	4767	tags=53%, list=38%, signal=34%
BP	GO:0043087	regulation of GTPase activity	287	0.388283224	1.591093091	6.34E-07	6.06059E-05	4.48E-05	3650	tags=42%, list=29%, signal=30%
BP	GO:0051493	regulation of cytoskeleton organization	339	0.375912136	1.553367356	7.88E-07	7.41257E-05	5.48E-05	4639	tags=50%, list=37%, signal=32%
BP	GO:0046578	regulation of Ras protein signal transduction	122	0.457970268	1.79711163	8.30E-07	7.69627E-05	5.69E-05	3670	tags=53%, list=29%, signal=38%
BP	GO:0007507	heart development	311	0.386754463	1.589863992	8.98E-07	8.11144E-05	6E-05	3929	tags=44%, list=31%, signal=31%
BP	GO:0000184	nuclear-transcribed mRNA catabolic process, nonsense-mediated decay	108	-0.32716332	-2.027456039	9.00E-07	8.11144E-05	6E-05	1671	tags=31%, list=13%, signal=27%
MF	GO:0003725	double-stranded RNA binding	54	0.568040411	2.049726247	9.71E-07	8.50001E-05	6.28E-05	3596	tags=65%, list=29%, signal=47%
BP	GO:0001885	endothelial cell development	43	0.589438785	2.048840784	9.78E-07	8.50001E-05	6.28E-05	2386	tags=58%, list=19%, signal=47%
BP	GO:0006897	endocytosis	318	0.38133555	1.569241659	9.84E-07	8.50001E-05	6.28E-05	3614	tags=42%, list=29%, signal=31%
BP	GO:0006281	DNA repair	331	0.372103802	1.536357683	1.07E-06	9.11427E-05	6.74E-05	4621	tags=50%, list=37%, signal=33%
BP	GO:0001570	vasculogenesis	53	0.565798131	2.033911643	1.14E-06	9.54673E-05	7.06E-05	1515	tags=43%, list=12%, signal=38%
CC	GO:0005911	cell-cell junction	274	0.389862301	1.593852957	1.17E-06	9.72635E-05	7.19E-05	3441	tags=42%, list=27%, signal=31%
BP	GO:0071559	response to transforming growth factor beta	160	0.43286718	1.734646615	1.21E-06	9.9001E-05	7.32E-05	3910	tags=49%, list=31%, signal=34%
MF	GO:0003682	chromatin binding	360	0.369652346	1.530170263	1.26E-06	0.000101687	7.52E-05	5322	tags=56%, list=42%, signal=33%
MF	GO:0019955	cytokine binding	64	0.534570922	1.979240262	1.28E-06	0.000102304	7.56E-05	1941	tags=41%, list=15%, signal=35%
BP	GO:0007369	gastrulation	95	0.482798216	1.85872392	1.49E-06	0.000117563	8.69E-05	2986	tags=45%, list=24%, signal=35%
BP	GO:0097435	supramolecular fiber organization	417	0.360508116	1.496654641	1.65E-06	0.000128065	9.47E-05	3624	tags=41%, list=29%, signal=30%
BP	GO:0051640	organelle localization	392	0.362232998	1.502509531	1.67E-06	0.000128065	9.47E-05	4635	tags=48%, list=37%, signal=31%
CC	GO:0005819	spindle	206	0.413802216	1.67687117	1.69E-06	0.000128065	9.47E-05	4594	tags=55%, list=36%, signal=35%
BP	GO:0007015	actin filament organization	259	0.390705079	1.596704155	1.77E-06	0.000132976	9.83E-05	3279	tags=41%, list=26%, signal=31%
BP	GO:0010632	regulation of epithelial cell migration	156	0.432438795	1.728959364	1.91E-06	0.00014176	0.000105	3397	tags=44%, list=27%, signal=33%
BP	GO:0008380	RNA splicing	311	0.380669368	1.564849484	2.05E-06	0.000150059	0.000111	5253	tags=59%, list=42%, signal=35%
BP	GO:0120035	regulation of plasma membrane bounded cell projection organization	384	0.359864482	1.491561178	2.18E-06	0.000158306	0.000117	4508	tags=47%, list=36%, signal=31%
CC	GO:0005925	focal adhesion	335	0.36881226	1.522981397	2.22E-06	0.000158972	0.000118	3955	tags=45%, list=31%, signal=32%
BP	GO:0001936	regulation of endothelial cell proliferation	76	0.511591092	1.929611302	2.27E-06	0.000160882	0.000119	2311	tags=43%, list=18%, signal=36%
MF	GO:0015078	proton transmembrane transporter activity	60	-0.407202596	-2.293149532	2.31E-06	0.00016201	0.00012	1186	tags=30%, list=9%, signal=27%
CC	GO:0030496	midbody	118	0.457939966	1.794516336	2.44E-06	0.000169385	0.000125	4717	tags=58%, list=37%, signal=37%
MF	GO:0019904	protein domain specific binding	444	0.355350807	1.477529866	2.87E-06	0.000191177	0.000141	5026	tags=51%, list=40%, signal=32%
BP	GO:0045664	regulation of neuron differentiation	363	0.368079386	1.523858823	2.87E-06	0.000191177	0.000141	3808	tags=42%, list=30%, signal=30%
BP	GO:1904018	positive regulation of vasculature development	120	0.45786308	1.794521	2.90E-06	0.000191177	0.000141	1891	tags=33%, list=15%, signal=29%
BP	GO:0022604	regulation of cell morphogenesis	296	0.381395658	1.564058706	2.94071E-06	0.000191177	0.000141	3443	tags=42%, list=27%, signal=31%
BP	GO:0031344	regulation of cell projection organization	388	0.361981268	1.500534433	2.94071E-06	0.000191177	0.000141	4508	tags=47%, list=36%, signal=31%

BP	GO:0043547	positive regulation of GTPase activity	236	0.393254025	1.602847665	2.94071E-06	0.000191177	0.000141	3650	tags=43%, list=29%, signal=31%
BP	GO:0043534	blood vessel endothelial cell migration	82	0.499670126	1.890156265	3.03886E-06	0.000195541	0.000145	2937	tags=48%, list=23%, signal=37%
BP	GO:0045765	regulation of angiogenesis	189	0.413621885	1.670714066	3.18465E-06	0.000202853	0.00015	2690	tags=37%, list=21%, signal=30%
BP	GO:0071560	cellular response to transforming growth factor beta stimulus	155	0.43166592	1.726154387	3.3568E-06	0.000210764	0.000156	3910	tags=49%, list=31%, signal=34%
CC	GO:0005684	U2-type spliceosomal complex	70	0.515488017	1.932309226	3.37571E-06	0.000210764	0.000156	4738	tags=69%, list=38%, signal=43%
CC	GO:0030055	cell-substrate junction	337	0.366985402	1.515605418	3.49102E-06	0.000215827	0.00016	3955	tags=45%, list=31%, signal=31%
BP	GO:0044089	positive regulation of cellular component biogenesis	313	0.378896062	1.557944098	3.62927E-06	0.000222196	0.000164	4230	tags=46%, list=34%, signal=32%
BP	GO:1901990	regulation of mitotic cell cycle phase transition	284	0.38351741	1.569572098	3.90578E-06	0.000236825	0.000175	4902	tags=53%, list=39%, signal=33%
MF	GO:0019901	protein kinase binding	414	0.355444679	1.475601039	4.11316E-06	0.000247025	0.000183	4295	tags=46%, list=34%, signal=31%
MF	GO:0005096	GTPase activator activity	164	0.421258121	1.690934243	4.32919E-06	0.000257546	0.00019	3463	tags=43%, list=27%, signal=32%
MF	GO:0140098	catalytic activity, acting on RNA	235	0.390522497	1.591443732	4.4588E-06	0.000262777	0.000194	5234	tags=57%, list=42%, signal=34%
BP	GO:0060759	regulation of response to cytokine stimulus	117	0.456729636	1.790367531	4.5644E-06	0.000264329	0.000195	5140	tags=67%, list=41%, signal=40%
BP	GO:0007411	axon guidance	149	0.43522428	1.736080231	4.61087E-06	0.000264329	0.000195	2829	tags=42%, list=22%, signal=33%
BP	GO:0097485	neuron projection guidance	149	0.43522428	1.736080231	4.61087E-06	0.000264329	0.000195	2829	tags=42%, list=22%, signal=33%
BP	GO:0007266	Rho protein signal transduction	88	0.48936653	1.866680188	4.7148E-06	0.000267852	0.000198	3421	tags=51%, list=27%, signal=38%
BP	GO:0051345	positive regulation of hydrolase activity	446	0.347441723	1.445233071	4.87356E-06	0.000274399	0.000203	3650	tags=38%, list=29%, signal=28%
MF	GO:0008047	enzyme activator activity	323	0.370727712	1.52857276	5.01182E-06	0.000279686	0.000207	3504	tags=39%, list=28%, signal=29%
CC	GO:0022625	cytosolic large ribosomal subunit	51	-0.437061159	-2.36232617	5.21579E-06	0.000288516	0.000213	1286	tags=35%, list=10%, signal=32%
CC	GO:0022627	cytosolic small ribosomal subunit	43	-0.451441297	-2.436935226	5.90402E-06	0.000323746	0.000239	2831	tags=53%, list=22%, signal=42%
BP	GO:0070646	protein modification by small protein removal	205	0.406901379	1.647921576	5.98558E-06	0.000324406	0.00024	4973	tags=57%, list=39%, signal=35%
MF	GO:0051020	GTPase binding	345	0.360517603	1.489672917	6.18698E-06	0.000324406	0.00024	4313	tags=47%, list=34%, signal=32%
CC	GO:0099081	supramolecular polymer	448	0.346086444	1.43955487	6.18698E-06	0.000324406	0.00024	4833	tags=51%, list=38%, signal=32%
BP	GO:1901987	regulation of cell cycle phase transition	299	0.37610596	1.543498964	6.18698E-06	0.000324406	0.00024	4902	tags=52%, list=39%, signal=33%
MF	GO:0030695	GTPase regulator activity	187	0.407555291	1.645771295	6.19317E-06	0.000324406	0.00024	3468	tags=42%, list=28%, signal=31%
BP	GO:0061028	establishment of endothelial barrier	29	0.629602962	2.050044778	6.22472E-06	0.000324406	0.00024	2386	tags=62%, list=19%, signal=50%
BP	GO:0048729	tissue morphogenesis	374	0.360524794	1.494302769	6.60175E-06	0.000341235	0.000252	4451	tags=49%, list=35%, signal=32%
BP	GO:0042330	taxis	324	0.371098562	1.530628014	6.74E-06	0.000345549	0.000255	2829	tags=34%, list=22%, signal=27%
MF	GO:0004386	helicase activity	118	0.449605962	1.761858111	6.93367E-06	0.000352611	0.000261	4121	tags=56%, list=33%, signal=38%
CC	GO:0031252	cell leading edge	262	0.383844211	1.56749911	7.44076E-06	0.000375371	0.000278	3650	tags=43%, list=29%, signal=31%
BP	GO:0006935	chemotaxis	322	0.369902902	1.524406519	7.57226E-06	0.000376366	0.000278	2829	tags=34%, list=22%, signal=27%
BP	GO:0044843	cell cycle G1/S phase transition	180	0.409878287	1.650050129	7.57983E-06	0.000376366	0.000278	4679	tags=54%, list=37%, signal=35%
MF	GO:0017016	Ras GTPase binding	266	0.375578817	1.534132105	7.98907E-06	0.000393587	0.000291	4313	tags=48%, list=34%, signal=33%
CC	GO:0005667	transcription regulator complex	256	0.378372986	1.54511192	8.12801E-06	0.000397327	0.000294	5184	tags=57%, list=41%, signal=35%
BP	GO:0000226	microtubule cytoskeleton organization	302	0.372811094	1.529829937	8.40588E-06	0.00040775	0.000301	4866	tags=53%, list=39%, signal=33%
BP	GO:0034340	response to type I interferon	72	0.501372358	1.877317371	8.93365E-06	0.000430043	0.000318	4753	tags=69%, list=38%, signal=44%
BP	GO:0051960	regulation of nervous system development	491	0.339794036	1.416683249	9.37844E-06	0.000448034	0.000331	4106	tags=42%, list=33%, signal=30%
BP	GO:0034330	cell junction organization	354	0.361038505	1.494083553	9.51738E-06	0.000451253	0.000334	4062	tags=45%, list=32%, signal=32%
BP	GO:0009615	response to virus	212	0.389772929	1.582105063	9.65632E-06	0.000453322	0.000335	5271	tags=59%, list=42%, signal=35%
CC	GO:0005681	spliceosomal complex	140	0.432053749	1.711411638	9.70479E-06	0.000453322	0.000335	4757	tags=61%, list=38%, signal=38%

CC	GO:0098687	chromosomal region	192	0.405786633	1.640701554	1.00832E-05	0.000467534	0.000346	5309	tags=60%, list=42%, signal=35%
BP	GO:0050851	antigen receptor-mediated signaling pathway	160	0.417991461	1.675034531	1.03717E-05	0.000477402	0.000353	3297	tags=41%, list=26%, signal=31%
BP	GO:0003177	pulmonary valve development	13	0.786520262	2.152827084	1.08079E-05	0.000493876	0.000365	2159	tags=69%, list=17%, signal=57%
BP	GO:0031424	keratinization	24	-0.584455046	-2.522592556	1.1E-05	0.000498526	0.000369	1599	tags=62%, list=13%, signal=55%
BP	GO:0043903	regulation of symbiotic process	150	0.421784837	1.684615822	1.10678E-05	0.000498526	0.000369	4427	tags=56%, list=35%, signal=37%
BP	GO:0051129	negative regulation of cellular component organization	450	0.347512576	1.445668909	1.14625E-05	0.000510148	0.000377	3279	tags=37%, list=26%, signal=28%
BP	GO:0071711	basement membrane organization	20	0.700862428	2.106009093	1.15633E-05	0.000510148	0.000377	2207	tags=75%, list=18%, signal=62%
BP	GO:0032956	regulation of actin cytoskeleton organization	222	0.389674135	1.58434122	1.16014E-05	0.000510148	0.000377	3279	tags=41%, list=26%, signal=31%
BP	GO:0034446	substrate adhesion-dependent cell spreading	72	0.498377647	1.866104103	1.16494E-05	0.000510148	0.000377	3605	tags=54%, list=29%, signal=39%
BP	GO:0019058	viral life cycle	224	0.393860204	1.602262775	1.21572E-05	0.000528712	0.000391	4869	tags=56%, list=39%, signal=35%
BP	GO:0007017	microtubule-based process	425	0.344256505	1.429607655	1.27129E-05	0.000549095	0.000406	4866	tags=50%, list=39%, signal=32%
BP	GO:0001704	formation of primary germ layer	58	0.523467963	1.913717669	1.29124E-05	0.000553914	0.00041	3201	tags=48%, list=25%, signal=36%
MF	GO:0003712	transcription coregulator activity	328	0.363494059	1.500014434	1.34076E-05	0.000571274	0.000422	5074	tags=54%, list=40%, signal=33%
BP	GO:0000082	G1/S transition of mitotic cell cycle	168	0.411963909	1.651453729	1.38521E-05	0.000586252	0.000433	4668	tags=55%, list=37%, signal=35%
BP	GO:0006405	RNA export from nucleus	101	0.457080805	1.76984916	1.42772E-05	0.000600212	0.000444	5202	tags=66%, list=41%, signal=39%
BP	GO:0090287	regulation of cellular response to growth factor stimulus	161	0.410759276	1.646064044	1.46902E-05	0.000609647	0.000451	2910	tags=39%, list=23%, signal=30%
MF	GO:0003779	actin binding	242	0.377953831	1.541493228	1.49401E-05	0.000609647	0.000451	3211	tags=40%, list=25%, signal=31%
BP	GO:0030198	extracellular matrix organization	207	0.397750807	1.61225195	1.49401E-05	0.000609647	0.000451	4832	tags=55%, list=38%, signal=34%
BP	GO:0043062	extracellular structure organization	207	0.397750807	1.61225195	1.49401E-05	0.000609647	0.000451	4832	tags=55%, list=38%, signal=34%
CC	GO:0030027	lamellipodium	143	0.423525595	1.684148425	1.4985E-05	0.000609647	0.000451	4668	tags=57%, list=37%, signal=37%
BP	GO:0001959	regulation of cytokine-mediated signaling pathway	110	0.451542554	1.760917499	1.51366E-05	0.00061187	0.000452	5140	tags=66%, list=41%, signal=40%
BP	GO:0060337	type I interferon signaling pathway	69	0.494324769	1.845428777	1.62653E-05	0.00064917	0.00048	4753	tags=68%, list=38%, signal=43%
BP	GO:0071357	cellular response to type I interferon	69	0.494324769	1.845428777	1.62653E-05	0.00064917	0.00048	4753	tags=68%, list=38%, signal=43%
BP	GO:0016579	protein deubiquitination	191	0.399999582	1.616240343	1.77503E-05	0.000703982	0.00052	4973	tags=57%, list=39%, signal=35%
BP	GO:0018205	peptidyl-lysine modification	252	0.378732504	1.54612823	1.8291E-05	0.000719862	0.000532	4900	tags=56%, list=39%, signal=35%
BP	GO:1903900	regulation of viral life cycle	99	0.457526571	1.769716233	1.8379E-05	0.000719862	0.000532	5183	tags=68%, list=41%, signal=40%
CC	GO:0098589	membrane region	184	0.39768224	1.605131723	1.94274E-05	0.00075623	0.000559	2291	tags=34%, list=18%, signal=28%
CC	GO:0016607	nuclear speck	259	0.374199748	1.529251406	2.02457E-05	0.00078325	0.000579	4821	tags=54%, list=38%, signal=34%
BP	GO:0045596	negative regulation of cell differentiation	389	0.347936092	1.442579601	2.0525E-05	0.000789211	0.000583	4565	tags=47%, list=36%, signal=31%
BP	GO:0006913	nucleocytoplasmic transport	245	0.3777399	1.542053226	2.1642E-05	0.000813149	0.000601	5674	tags=60%, list=45%, signal=34%
BP	GO:0010608	posttranscriptional regulation of gene expression	436	0.342896202	1.425612068	2.1642E-05	0.000813149	0.000601	3989	tags=42%, list=32%, signal=30%
BP	GO:0007179	transforming growth factor beta receptor signaling pathway	126	0.428381503	1.687191362	2.17944E-05	0.000813149	0.000601	3775	tags=48%, list=30%, signal=34%
MF	GO:0016614	oxidoreductase activity, acting on CH-OH group of donors	80	-0.327060585	-1.933919708	2.18883E-05	0.000813149	0.000601	1200	tags=28%, list=10%, signal=25%
BP	GO:0002429	immune response-activating cell surface receptor signaling pathway	229	0.383442881	1.561646178	2.19212E-05	0.000813149	0.000601	3312	tags=39%, list=26%, signal=30%
BP	GO:0002757	immune response-activating signal transduction	229	0.383442881	1.561646178	2.19212E-05	0.000813149	0.000601	3312	tags=39%, list=26%, signal=30%
BP	GO:0006898	receptor-mediated endocytosis	145	0.41701629	1.66022701	2.25699E-05	0.000832315	0.000615	3406	tags=43%, list=27%, signal=32%
CC	GO:0099512	supramolecular fiber	444	0.345317397	1.435811477	2.30382E-05	0.000844645	0.000624	4833	tags=51%, list=38%, signal=32%
BP	GO:0035023	regulation of Rho protein signal transduction	53	0.525602183	1.889416634	2.31969E-05	0.000845549	0.000625	3410	tags=55%, list=27%, signal=40%
BP	GO:0006720	isoprenoid metabolic process	70	-0.350404439	-1.964949139	2.35899E-05	0.000854931	0.000632	1621	tags=40%, list=13%, signal=35%

BP	GO:0044839	cell cycle G2/M phase transition	179	0.403209582	1.623318501	2.38998E-05	0.000861214	0.000637	4084	tags=48%, list=32%, signal=33%
BP	GO:0051168	nuclear export	146	0.416131789	1.656292648	2.45325E-05	0.000878988	0.00065	5752	tags=68%, list=46%, signal=37%
CC	GO:0005912	adherens junction	95	0.458807973	1.766363928	2.49072E-05	0.000885787	0.000655	3406	tags=49%, list=27%, signal=36%
BP	GO:0007178	transmembrane receptor protein serine/threonine kinase signaling pathway	192	0.398948734	1.61305414	2.50179E-05	0.000885787	0.000655	2986	tags=40%, list=24%, signal=31%
BP	GO:0050852	T cell receptor signaling pathway	136	0.426025046	1.685896355	2.51437E-05	0.000885787	0.000655	3297	tags=42%, list=26%, signal=31%
MF	GO:0060589	nucleoside-triphosphatase regulator activity	217	0.383644341	1.559858297	2.58307E-05	0.000904936	0.000669	3468	tags=40%, list=28%, signal=29%
CC	GO:0000775	chromosome, centromeric region	112	0.435163259	1.698303003	2.61441E-05	0.000910855	0.000673	5634	tags=68%, list=45%, signal=38%
BP	GO:0050657	nucleic acid transport	142	0.417890225	1.659327439	2.64685E-05	0.000912079	0.000674	5483	tags=63%, list=43%, signal=36%
BP	GO:0050658	RNA transport	142	0.417890225	1.659327439	2.64685E-05	0.000912079	0.000674	5483	tags=63%, list=43%, signal=36%
BP	GO:0071166	ribonucleoprotein complex localization	96	0.455266518	1.752159449	2.74971E-05	0.000942373	0.000697	5202	tags=66%, list=41%, signal=39%
BP	GO:0050767	regulation of neurogenesis	449	0.344371958	1.432656049	2.83439E-05	0.000966145	0.000714	4512	tags=46%, list=36%, signal=31%
CC	GO:0015629	actin cytoskeleton	302	0.364987388	1.497725366	2.94664E-05	0.000999007	0.000739	4711	tags=51%, list=37%, signal=33%
BP	GO:0000086	G2/M transition of mitotic cell cycle	171	0.408576428	1.637953085	3.00878E-05	0.001014618	0.00075	3170	tags=42%, list=25%, signal=32%
BP	GO:0010594	regulation of endothelial cell migration	113	0.443048815	1.727994432	3.0392E-05	0.001019426	0.000754	3056	tags=42%, list=24%, signal=32%
BP	GO:0051169	nuclear transport	247	0.375793149	1.534372753	3.05889E-05	0.001020602	0.000755	5202	tags=55%, list=41%, signal=33%
MF	GO:0016887	ATPase activity	254	0.373696935	1.525822175	3.11502E-05	0.001028445	0.00076	4121	tags=46%, list=33%, signal=32%
MF	GO:0031267	small GTPase binding	274	0.36890115	1.508158614	3.11502E-05	0.001028445	0.00076	4313	tags=48%, list=34%, signal=32%
BP	GO:0016101	diterpenoid metabolic process	50	-0.407975687	-2.159669661	3.14046E-05	0.001031443	0.000763	2032	tags=48%, list=16%, signal=40%
CC	GO:0099513	polymeric cytoskeletal fiber	322	0.360695152	1.48646047	3.17114E-05	0.001036126	0.000766	4776	tags=52%, list=38%, signal=33%
BP	GO:0010975	regulation of neuron projection development	288	0.365607566	1.498645767	3.28339E-05	0.001066013	0.000788	4545	tags=49%, list=36%, signal=32%
BP	GO:1901343	negative regulation of vasculature development	75	0.483672418	1.822164635	3.29642E-05	0.001066013	0.000788	3549	tags=51%, list=28%, signal=37%
BP	GO:0045766	positive regulation of angiogenesis	105	0.443314509	1.722331001	3.39376E-05	0.001091891	0.000807	1891	tags=32%, list=15%, signal=28%
BP	GO:0010389	regulation of G2/M transition of mitotic cell cycle	135	0.422216791	1.668975991	3.41956E-05	0.001094605	0.000809	3127	tags=42%, list=25%, signal=32%
BP	GO:0030031	cell projection assembly	298	0.364446454	1.495072988	3.50789E-05	0.001117211	0.000826	4677	tags=49%, list=37%, signal=32%
BP	GO:0043632	modification-dependent macromolecule catabolic process	461	0.334137256	1.390504008	3.56402E-05	0.001129382	0.000835	4482	tags=46%, list=36%, signal=31%
BP	GO:1905314	semi-lunar valve development	21	0.673092742	2.04089072	3.64027E-05	0.001147776	0.000849	2546	tags=57%, list=20%, signal=46%
MF	GO:0050839	cell adhesion molecule binding	337	0.352564621	1.456049335	3.73239E-05	0.001167234	0.000863	3090	tags=36%, list=25%, signal=28%
BP	GO:0003203	endocardial cushion morphogenesis	19	0.697423121	2.079745388	3.739E-05	0.001167234	0.000863	1515	tags=58%, list=12%, signal=51%
BP	GO:0051607	defense response to virus	162	0.410234245	1.644623974	3.85234E-05	0.001196693	0.000885	5369	tags=62%, list=43%, signal=36%
BP	GO:0001938	positive regulation of endothelial cell proliferation	56	0.518635243	1.885792961	3.93801E-05	0.001217309	0.0009	2282	tags=43%, list=18%, signal=35%
BP	GO:0120031	plasma membrane bounded cell projection assembly	291	0.367220228	1.505051804	4.4059E-05	0.001355297	0.001002	4677	tags=49%, list=37%, signal=32%
BP	GO:0032092	positive regulation of protein binding	63	0.500945763	1.853232319	4.48416E-05	0.001372676	0.001015	3441	tags=52%, list=27%, signal=38%
CC	GO:0097225	sperm midpiece	11	-0.767510167	-2.588389303	4.55832E-05	0.001385698	0.001024	1764	tags=73%, list=14%, signal=63%
BP	GO:1903311	regulation of mRNA metabolic process	229	0.378026655	1.539587541	4.57427E-05	0.001385698	0.001024	4816	tags=51%, list=38%, signal=32%
BP	GO:0051236	establishment of RNA localization	144	0.415242186	1.651811029	4.59262E-05	0.001385698	0.001024	5483	tags=63%, list=43%, signal=36%
BP	GO:0060627	regulation of vesicle-mediated transport	301	0.362436928	1.487329208	4.6304E-05	0.001390442	0.001028	4483	tags=47%, list=36%, signal=31%
BP	GO:0010769	regulation of cell morphogenesis involved in differentiation	186	0.400180447	1.615672121	4.69121E-05	0.00139821	0.001034	3174	tags=42%, list=25%, signal=32%
BP	GO:0050792	regulation of viral process	142	0.412450079	1.637726115	4.70061E-05	0.00139821	0.001034	5183	tags=63%, list=41%, signal=37%
CC	GO:0098857	membrane microdomain	177	0.395590957	1.590846077	4.75214E-05	0.001406902	0.00104	2291	tags=34%, list=18%, signal=28%

BP	GO:0071426	ribonucleoprotein complex export from nucleus	95	0.45290861	1.743651982	4.83441E-05	0.00142457	0.001053	5202	tags=65%, list=41%, signal=39%
BP	GO:0051017	actin filament bundle assembly	109	0.443882933	1.731629564	5.08937E-05	0.001485813	0.001098	3561	tags=50%, list=28%, signal=36%
BP	GO:0061572	actin filament bundle organization	109	0.443882933	1.731629564	5.08937E-05	0.001485813	0.001098	3561	tags=50%, list=28%, signal=36%
BP	GO:0003279	cardiac septum development	65	0.498169398	1.85503303	5.26168E-05	0.001529039	0.00113	4476	tags=66%, list=36%, signal=43%
BP	GO:1902903	regulation of supramolecular fiber organization	230	0.375828782	1.53083631	5.58453E-05	0.00161111	0.001191	4571	tags=49%, list=36%, signal=32%
BP	GO:0016525	negative regulation of angiogenesis	70	0.478969378	1.79541894	5.62397E-05	0.00161111	0.001191	3549	tags=50%, list=28%, signal=36%
BP	GO:2000181	negative regulation of blood vessel morphogenesis	70	0.478969378	1.79541894	5.62397E-05	0.00161111	0.001191	3549	tags=50%, list=28%, signal=36%
MF	GO:0060090	molecular adaptor activity	185	0.398993317	1.611312104	5.6463E-05	0.00161111	0.001191	3605	tags=45%, list=29%, signal=33%
BP	GO:0019221	cytokine-mediated signaling pathway	462	0.337303432	1.403847338	6.03519E-05	0.001714321	0.001267	4474	tags=45%, list=35%, signal=30%
BP	GO:0007162	negative regulation of cell adhesion	166	0.395251772	1.585297023	6.1603E-05	0.001742012	0.001288	2960	tags=40%, list=23%, signal=31%
BP	GO:0010638	positive regulation of organelle organization	405	0.340768657	1.414458854	6.71202E-05	0.001889553	0.001397	4723	tags=48%, list=37%, signal=31%
BP	GO:0051099	positive regulation of binding	123	0.422256941	1.656105162	6.77975E-05	0.001890973	0.001398	2965	tags=41%, list=24%, signal=32%
MF	GO:0004984	olfactory receptor activity	17	-0.632487142	-2.448317958	6.80702E-05	0.001890973	0.001398	1086	tags=47%, list=9%, signal=43%
BP	GO:0050911	detection of chemical stimulus involved in sensory perception of smell	17	-0.632487142	-2.448317958	6.80702E-05	0.001890973	0.001398	1086	tags=47%, list=9%, signal=43%
BP	GO:0007160	cell-matrix adhesion	136	0.415888695	1.645784073	6.97946E-05	0.001930372	0.001427	3090	tags=40%, list=25%, signal=30%
BP	GO:0050907	detection of chemical stimulus involved in sensory perception	18	-0.632152398	-2.569217645	7.2457E-05	0.001995258	0.001475	1086	tags=44%, list=9%, signal=41%
MF	GO:0140097	catalytic activity, acting on DNA	137	0.412133642	1.631367302	7.53931E-05	0.002061019	0.001524	4265	tags=51%, list=34%, signal=34%
BP	GO:0048663	neuron fate commitment	17	0.677782138	1.970521931	7.54988E-05	0.002061019	0.001524	1562	tags=47%, list=12%, signal=41%
BP	GO:0048598	embryonic morphogenesis	310	0.353557968	1.453422262	7.72726E-05	0.002100349	0.001553	3401	tags=38%, list=27%, signal=28%
BP	GO:0043254	regulation of protein-containing complex assembly	273	0.360761709	1.474552051	7.95287E-05	0.002152394	0.001591	4221	tags=43%, list=33%, signal=29%
BP	GO:0043487	regulation of RNA stability	142	0.407418707	1.617747917	8.31621E-05	0.002241111	0.001657	3101	tags=40%, list=25%, signal=31%
BP	GO:2000278	regulation of DNA biosynthetic process	78	0.467423281	1.764280398	8.4776E-05	0.002274883	0.001682	3598	tags=47%, list=29%, signal=34%
BP	GO:0030900	forebrain development	196	0.385144856	1.558180669	8.64697E-05	0.0023105	0.001708	4450	tags=49%, list=35%, signal=32%
CC	GO:0045121	membrane raft	176	0.39016365	1.56767903	8.98606E-05	0.002390976	0.001768	2291	tags=34%, list=18%, signal=28%
MF	GO:0051427	hormone receptor binding	110	0.431009673	1.68084374	9.08611E-05	0.002407438	0.00178	4157	tags=50%, list=33%, signal=34%
BP	GO:0006721	terpenoid metabolic process	56	-0.368623307	-2.08870106	9.13771E-05	0.002410978	0.001782	2032	tags=45%, list=16%, signal=38%
CC	GO:0005938	cell cortex	167	0.400372319	1.605614001	9.32516E-05	0.002450185	0.001811	4433	tags=53%, list=35%, signal=35%
MF	GO:0017048	Rho GTPase binding	100	0.446026572	1.727169296	9.49626E-05	0.002484789	0.001837	3421	tags=47%, list=27%, signal=35%
MF	GO:0061629	RNA polymerase II-specific DNA-binding transcription factor binding	184	0.386671841	1.560691366	9.65459E-05	0.002515778	0.00186	4157	tags=45%, list=33%, signal=30%
BP	GO:0045786	negative regulation of cell cycle	391	0.341870833	1.417924792	9.75775E-05	0.002532195	0.001872	4576	tags=46%, list=36%, signal=30%
MF	GO:0140297	DNA-binding transcription factor binding	233	0.371465723	1.513824774	9.87055E-05	0.002550971	0.001886	5119	tags=52%, list=41%, signal=31%
BP	GO:0007229	integrin-mediated signaling pathway	70	0.473388039	1.774497263	9.91231E-05	0.002551306	0.001886	2986	tags=44%, list=24%, signal=34%
BP	GO:0090068	positive regulation of cell cycle process	166	0.390833365	1.567575438	0.000101164	0.002593246	0.001917	4852	tags=54%, list=38%, signal=34%
BP	GO:0016570	histone modification	275	0.360804179	1.475345064	0.00010209	0.002595877	0.001919	5147	tags=56%, list=41%, signal=34%
BP	GO:0032970	regulation of actin filament-based process	243	0.369928347	1.509410695	0.00010209	0.002595877	0.001919	3279	tags=39%, list=26%, signal=29%
BP	GO:0010810	regulation of cell-substrate adhesion	128	0.418158112	1.648442191	0.000102912	0.002606281	0.001927	3410	tags=45%, list=27%, signal=33%
MF	GO:0004029	aldehyde dehydrogenase (NAD+) activity	12	-0.728778907	-2.525850134	0.000107103	0.002690801	0.001989	1878	tags=75%, list=15%, signal=64%
MF	GO:0004030	aldehyde dehydrogenase [NAD(P)+] activity	12	-0.728778907	-2.525850134	0.000107103	0.002690801	0.001989	1878	tags=75%, list=15%, signal=64%
BP	GO:0007492	endoderm development	40	0.544428547	1.862509783	0.000107743	0.002696131	0.001993	2986	tags=50%, list=24%, signal=38%

BP	GO:0031503	protein-containing complex localization	181	0.388852291	1.565923904	0.000108967	0.00271535	0.002007	5225	tags=59%, list=41%, signal=35%
BP	GO:1903670	regulation of sprouting angiogenesis	52	0.502090338	1.800165432	0.000109372	0.00271535	0.002007	2937	tags=50%, list=23%, signal=39%
BP	GO:0006511	ubiquitin-dependent protein catabolic process	447	0.334526072	1.391212516	0.000112242	0.00276236	0.002042	4482	tags=46%, list=36%, signal=30%
BP	GO:0110053	regulation of actin filament organization	174	0.396314416	1.590668139	0.000112467	0.00276236	0.002042	3279	tags=41%, list=26%, signal=31%
BP	GO:1902749	regulation of cell cycle G2/M phase transition	142	0.404339772	1.605522308	0.00011258	0.00276236	0.002042	3127	tags=41%, list=25%, signal=31%
BP	GO:0043687	post-translational protein modification	234	0.367973477	1.499327324	0.00011337	0.002770978	0.002049	4474	tags=47%, list=35%, signal=31%
BP	GO:0042572	retinol metabolic process	19	-0.594794164	-2.485394302	0.000118497	0.002885112	0.002133	1811	tags=58%, list=14%, signal=50%
BP	GO:0002764	immune response-regulating signaling pathway	256	0.358885959	1.465535316	0.000119033	0.002887	0.002134	3312	tags=36%, list=26%, signal=27%
BP	GO:0002009	morphogenesis of an epithelium	324	0.350839719	1.447068669	0.0001213	0.002930657	0.002167	3346	tags=39%, list=27%, signal=30%
BP	GO:0045603	positive regulation of endothelial cell differentiation	13	0.742622728	2.032672772	0.000121762	0.002930657	0.002167	2386	tags=62%, list=19%, signal=50%
BP	GO:0002253	activation of immune response	274	0.358815184	1.466924707	0.000128102	0.003071515	0.002271	3312	tags=38%, list=26%, signal=29%
BP	GO:0006310	DNA recombination	138	0.408935513	1.617309163	0.000129004	0.003081429	0.002278	4687	tags=56%, list=37%, signal=35%
BP	GO:2000279	negative regulation of DNA biosynthetic process	24	0.620740364	1.945163035	0.000132391	0.003144371	0.002325	3505	tags=67%, list=28%, signal=48%
BP	GO:0016569	covalent chromatin modification	282	0.357665366	1.463143315	0.000132636	0.003144371	0.002325	5166	tags=56%, list=41%, signal=34%
MF	GO:0051219	phosphoprotein binding	59	0.497187809	1.822342839	0.000134953	0.003187307	0.002356	4054	tags=58%, list=32%, signal=39%
BP	GO:0007169	transmembrane receptor protein tyrosine kinase signaling pathway	442	0.330052131	1.372100562	0.00013717	0.003215602	0.002377	3831	tags=38%, list=30%, signal=28%
BP	GO:0019941	modification-dependent protein catabolic process	453	0.332500356	1.383025172	0.00013717	0.003215602	0.002377	4482	tags=45%, list=36%, signal=30%
BP	GO:0070925	organelle assembly	488	0.323441295	1.348513809	0.000139438	0.003256645	0.002408	4869	tags=48%, list=39%, signal=31%
BP	GO:0060840	artery development	54	0.504247025	1.819533158	0.000140518	0.003269774	0.002417	4450	tags=69%, list=35%, signal=45%
MF	GO:0035257	nuclear hormone receptor binding	96	0.438896048	1.68915531	0.000146907	0.003405875	0.002518	3728	tags=47%, list=30%, signal=33%
BP	GO:0043488	regulation of mRNA stability	137	0.40624441	1.608055687	0.000151531	0.003500194	0.002588	3101	tags=40%, list=25%, signal=31%
BP	GO:0070831	basement membrane assembly	11	0.759097241	1.976592604	0.000155978	0.003589775	0.002654	2847	tags=91%, list=23%, signal=70%
BP	GO:1903510	mucopolysaccharide metabolic process	68	0.475222028	1.772595831	0.000159614	0.003660085	0.002706	3259	tags=50%, list=26%, signal=37%
BP	GO:0000910	cytokinesis	96	0.438364695	1.68711032	0.000160788	0.003661944	0.002707	4688	tags=59%, list=37%, signal=38%
BP	GO:0043123	positive regulation of I-kappaB kinase/NF-kappaB signaling	132	0.405596686	1.601293551	0.000161131	0.003661944	0.002707	5369	tags=60%, list=43%, signal=35%
BP	GO:0006029	proteoglycan metabolic process	56	0.50053055	1.819963071	0.000161437	0.003661944	0.002707	3272	tags=50%, list=26%, signal=37%
BP	GO:0045069	regulation of viral genome replication	67	0.480247599	1.790774569	0.000176105	0.003980351	0.002943	5183	tags=72%, list=41%, signal=42%
BP	GO:0052372	modulation by symbiont of entry into host	27	0.590948813	1.897783535	0.000177387	0.003994998	0.002954	3019	tags=63%, list=24%, signal=48%
BP	GO:0006611	protein export from nucleus	136	0.407750471	1.613578919	0.000179054	0.0040182	0.002971	5272	tags=61%, list=42%, signal=36%
BP	GO:0010720	positive regulation of cell development	304	0.351375114	1.442623798	0.000180248	0.004026097	0.002977	3910	tags=41%, list=31%, signal=29%
MF	GO:0016616	oxidoreductase activity, acting on the CH-OH group of donors, NAD or NADP as acceptor	74	-0.313249814	-1.79655373	0.000180683	0.004026097	0.002977	1200	tags=36%, list=10%, signal=33%
BP	GO:0002042	cell migration involved in sprouting angiogenesis	36	0.548222162	1.842549527	0.000182003	0.004041232	0.002988	2937	tags=56%, list=23%, signal=43%
BP	GO:0030336	negative regulation of cell migration	165	0.383654133	1.539876024	0.000185152	0.004096739	0.003029	3293	tags=40%, list=26%, signal=30%
BP	GO:0031400	negative regulation of protein modification process	390	0.332960592	1.380852795	0.00018705	0.004124249	0.003049	4054	tags=41%, list=32%, signal=29%
BP	GO:0042060	wound healing	278	0.355900728	1.455285385	0.000191584	0.004209511	0.003112	4628	tags=50%, list=37%, signal=32%
BP	GO:1901991	negative regulation of mitotic cell cycle phase transition	164	0.390528529	1.567585358	0.000198783	0.00435252	0.003218	3398	tags=40%, list=27%, signal=29%
BP	GO:0050770	regulation of axonogenesis	109	0.426049074	1.662057986	0.000200996	0.004358771	0.003222	3174	tags=43%, list=25%, signal=33%
BP	GO:0048863	stem cell differentiation	163	0.39047032	1.566312183	0.000201055	0.004358771	0.003222	2898	tags=39%, list=23%, signal=30%
BP	GO:0006261	DNA-dependent DNA replication	88	0.443977479	1.693544433	0.000201142	0.004358771	0.003222	4411	tags=59%, list=35%, signal=39%

BP	GO:0034470	ncRNA processing	281	0.351036804	1.436283113	0.000205188	0.004431209	0.003276	5797	tags=60%, list=46%, signal=33%
BP	GO:1903901	negative regulation of viral life cycle	54	0.497601684	1.795554	0.000206643	0.004447416	0.003288	3225	tags=52%, list=26%, signal=39%
BP	GO:0045087	innate immune response	469	0.325905691	1.356665872	0.000207455	0.004449694	0.00329	5271	tags=52%, list=42%, signal=31%
BP	GO:0002768	immune response-regulating cell surface receptor signaling pathway	254	0.361951984	1.477866991	0.000209722	0.004483076	0.003314	3312	tags=37%, list=26%, signal=28%
BP	GO:0010811	positive regulation of cell-substrate adhesion	68	0.47096562	1.75671927	0.000210876	0.004492504	0.003321	3852	tags=56%, list=31%, signal=39%
BP	GO:0007059	chromosome segregation	173	0.385967193	1.547907076	0.000219229	0.004654742	0.003441	4213	tags=46%, list=33%, signal=31%
BP	GO:0003208	cardiac ventricle morphogenesis	33	0.567942074	1.88544524	0.000220786	0.004672072	0.003454	1515	tags=42%, list=12%, signal=37%
BP	GO:0061013	regulation of mRNA catabolic process	151	0.395584696	1.578484884	0.000224222	0.0047289	0.003496	3107	tags=39%, list=25%, signal=30%
MF	GO:0019838	growth factor binding	75	0.457640606	1.724093613	0.000225795	0.004730933	0.003498	2596	tags=43%, list=21%, signal=34%
BP	GO:0060485	mesenchyme development	153	0.398166756	1.592481544	0.000225818	0.004730933	0.003498	3332	tags=43%, list=26%, signal=32%
BP	GO:0003170	heart valve development	37	0.551911338	1.863772047	0.000232554	0.004855912	0.00359	2877	tags=51%, list=23%, signal=40%
BP	GO:0003013	circulatory system process	252	0.359426207	1.467312679	0.000234684	0.004884218	0.003611	2492	tags=32%, list=20%, signal=26%
BP	GO:0009792	embryo development ending in birth or egg hatching	379	0.334024417	1.385053771	0.000239241	0.004962675	0.003669	3633	tags=39%, list=29%, signal=28%
CC	GO:0071011	precatalytic spliceosome	41	0.531483021	1.833605372	0.000240488	0.004972191	0.003676	4738	tags=66%, list=38%, signal=41%
BP	GO:0070268	cornification	17	-0.598391374	-2.316335383	0.000243325	0.00501441	0.003707	1564	tags=82%, list=12%, signal=72%
MF	GO:0050431	transforming growth factor beta binding	14	0.695141924	1.930279935	0.000244139	0.005014781	0.003707	1941	tags=64%, list=15%, signal=54%
BP	GO:0002011	morphogenesis of an epithelial sheet	35	0.549160338	1.838466876	0.000245256	0.005021374	0.003712	2937	tags=60%, list=23%, signal=46%
BP	GO:0002831	regulation of response to biotic stimulus	253	0.355973789	1.45330175	0.000248355	0.005068363	0.003747	5425	tags=56%, list=43%, signal=33%
CC	GO:0001533	cornified envelope	11	-0.712166854	-2.401746773	0.000251341	0.005105602	0.003775	1599	tags=82%, list=13%, signal=72%
BP	GO:0043009	chordate embryonic development	372	0.334238481	1.384645552	0.000252911	0.005105602	0.003775	3362	tags=37%, list=27%, signal=28%
BP	GO:0000280	nuclear division	204	0.369476378	1.496275083	0.000253164	0.005105602	0.003775	4594	tags=49%, list=36%, signal=31%
CC	GO:0034399	nuclear periphery	100	0.434519053	1.682608201	0.000253418	0.005105602	0.003775	4939	tags=62%, list=39%, signal=38%
BP	GO:0006302	double-strand break repair	131	0.404510887	1.595028379	0.000255206	0.005119302	0.003785	5070	tags=58%, list=40%, signal=35%
CC	GO:0030427	site of polarized growth	114	0.409515478	1.599141364	0.000255722	0.005119302	0.003785	3821	tags=50%, list=30%, signal=35%
BP	GO:0051052	regulation of DNA metabolic process	209	0.366823715	1.48792511	0.000262025	0.005220848	0.00386	4239	tags=44%, list=34%, signal=30%
CC	GO:0001650	fibrillar center	98	0.425234939	1.642281418	0.00026245	0.005220848	0.00386	4687	tags=58%, list=37%, signal=37%
BP	GO:0045071	negative regulation of viral genome replication	39	0.536719349	1.830490319	0.000265601	0.005266927	0.003894	2565	tags=49%, list=20%, signal=39%
BP	GO:0019882	antigen processing and presentation	165	0.381449631	1.531027797	0.000267116	0.005273845	0.003899	5675	tags=61%, list=45%, signal=34%
MF	GO:0046332	SMAD binding	54	0.493133643	1.779431449	0.000268185	0.005273845	0.003899	4597	tags=65%, list=36%, signal=41%
BP	GO:0019884	antigen processing and presentation of exogenous antigen	138	0.400810509	1.585175382	0.000268459	0.005273845	0.003899	5675	tags=63%, list=45%, signal=35%
BP	GO:0045165	cell fate commitment	100	0.433502057	1.678670041	0.000272017	0.005312186	0.003927	1691	tags=32%, list=13%, signal=28%
MF	GO:0016903	oxidoreductase activity, acting on the aldehyde or oxo group of donors	31	-0.442214447	-2.12259357	0.000272096	0.005312186	0.003927	2328	tags=55%, list=18%, signal=45%
BP	GO:0034502	protein localization to chromosome	48	0.502470548	1.778832287	0.000276674	0.0053849	0.003981	2801	tags=46%, list=22%, signal=36%
BP	GO:0019079	viral genome replication	83	0.449448627	1.702582392	0.000281891	0.005469554	0.004044	5183	tags=64%, list=41%, signal=38%
BP	GO:0048762	mesenchymal cell differentiation	129	0.398748311	1.571147565	0.000282795	0.005470264	0.004044	3332	tags=44%, list=26%, signal=33%
BP	GO:0003205	cardiac chamber development	90	0.441097052	1.687955634	0.000286258	0.00551648	0.004078	3929	tags=52%, list=31%, signal=36%
BP	GO:0032835	glomerulus development	35	0.545462635	1.826087787	0.000286934	0.00551648	0.004078	2240	tags=46%, list=18%, signal=38%
BP	GO:0050817	coagulation	178	0.378146075	1.522012241	0.000289656	0.005551879	0.004105	5146	tags=56%, list=41%, signal=34%
BP	GO:0009063	cellular amino acid catabolic process	74	-0.306952142	-1.760435248	0.000291036	0.005561442	0.004112	825	tags=27%, list=7%, signal=25%

BP	GO:0034660	ncRNA metabolic process	322	0.344301663	1.418901278	0.000293923	0.005597344	0.004138	4808	tags=49%, list=38%, signal=31%
BP	GO:0035904	aorta development	34	0.558172667	1.857164293	0.000294691	0.005597344	0.004138	4423	tags=74%, list=35%, signal=48%
BP	GO:0003007	heart morphogenesis	132	0.39931744	1.576503121	0.000300885	0.005697833	0.004212	3475	tags=42%, list=28%, signal=31%
BP	GO:0010634	positive regulation of epithelial cell migration	99	0.42719622	1.652398206	0.000318514	0.006013629	0.004446	2598	tags=37%, list=21%, signal=30%
CC	GO:0098978	glutamatergic synapse	165	0.379553174	1.523415971	0.000321907	0.006059547	0.00448	4805	tags=52%, list=38%, signal=32%
BP	GO:0009611	response to wounding	344	0.340039461	1.405025034	0.000325821	0.006103925	0.004513	4628	tags=47%, list=37%, signal=31%
BP	GO:0009127	purine nucleoside monophosphate biosynthetic process	17	0.646705516	1.880172595	0.000326201	0.006103925	0.004513	1245	tags=47%, list=10%, signal=42%
BP	GO:0071897	DNA biosynthetic process	130	0.404224978	1.593797628	0.000333375	0.006219719	0.004598	4387	tags=49%, list=35%, signal=32%
BP	GO:0001523	retinoid metabolic process	46	-0.365119603	-1.920698822	0.000338754	0.006301417	0.004659	1811	tags=41%, list=14%, signal=36%
BP	GO:0006406	mRNA export from nucleus	80	0.442848014	1.673088321	0.000346767	0.006399316	0.004731	4890	tags=60%, list=39%, signal=37%
BP	GO:0071427	mRNA-containing ribonucleoprotein complex export from nucleus	80	0.442848014	1.673088321	0.000346767	0.006399316	0.004731	4890	tags=60%, list=39%, signal=37%
MF	GO:0016628	oxidoreductase activity, acting on the CH-CH group of donors, NAD or NADP as acceptor	20	-0.540405795	-2.300934947	0.000347897	0.006399316	0.004731	2016	tags=65%, list=16%, signal=55%
BP	GO:1904356	regulation of telomere maintenance via telomere lengthening	43	0.508174002	1.766371078	0.000348076	0.006399316	0.004731	3254	tags=49%, list=26%, signal=36%
MF	GO:0019842	vitamin binding	68	-0.322258722	-1.788219022	0.00035018	0.006419295	0.004746	1890	tags=38%, list=15%, signal=33%
BP	GO:0035987	endodermal cell differentiation	21	0.632981557	1.919269224	0.000353098	0.006454013	0.004772	2986	tags=62%, list=24%, signal=47%
CC	GO:0071005	U2-type precatalytic spliceosome	40	0.52819194	1.806963762	0.00035743	0.006500799	0.004806	4738	tags=65%, list=38%, signal=41%
CC	GO:0005874	microtubule	219	0.366392611	1.489598617	0.000357719	0.006500799	0.004806	4776	tags=53%, list=38%, signal=34%
MF	GO:0003724	RNA helicase activity	55	0.477752964	1.729256993	0.000363116	0.006579904	0.004865	5234	tags=71%, list=42%, signal=42%
BP	GO:0061061	muscle structure development	347	0.341683812	1.412009344	0.000366833	0.006628219	0.0049	2878	tags=33%, list=23%, signal=26%
BP	GO:0003176	aortic valve development	19	0.649792293	1.937708235	0.000372205	0.006700514	0.004954	2546	tags=53%, list=20%, signal=42%
BP	GO:0044319	wound healing, spreading of cells	21	0.629350581	1.908259709	0.000374022	0.006700514	0.004954	2937	tags=71%, list=23%, signal=55%
BP	GO:0090505	epiboly involved in wound healing	21	0.629350581	1.908259709	0.000374022	0.006700514	0.004954	2937	tags=71%, list=23%, signal=55%
BP	GO:0043968	histone H2A acetylation	14	0.685957684	1.904777009	0.000375556	0.006708942	0.00496	3250	tags=71%, list=26%, signal=53%
BP	GO:0051058	negative regulation of small GTPase mediated signal transduction	39	0.529624667	1.806293786	0.000377687	0.006727958	0.004974	3410	tags=54%, list=27%, signal=39%
BP	GO:0060674	placenta blood vessel development	21	0.628435695	1.905485675	0.000379253	0.00673681	0.004981	443	tags=29%, list=4%, signal=28%
BP	GO:0022406	membrane docking	109	0.419470716	1.636395185	0.000390127	0.006907526	0.005107	4841	tags=56%, list=38%, signal=35%
BP	GO:0030166	proteoglycan biosynthetic process	41	0.5237173	1.8068139	0.0003911	0.0069075	0.005	3184	tags=54%, list=25%, signal=40%
BP	GO:0002478	antigen processing and presentation of exogenous peptide antigen	136	0.400877627	1.586381215	0.000396551	0.006985059	0.005164	5675	tags=63%, list=45%, signal=35%
BP	GO:0045787	positive regulation of cell cycle	221	0.362661321	1.474501223	4.03E-04	0.007061861	0.005221	4852	tags=52%, list=38%, signal=32%
BP	GO:0003148	outflow tract septum morphogenesis	13	0.7157027	1.958988511	4.04E-04	0.007061861	0.005221	2746	tags=77%, list=22%, signal=60%
MF	GO:0003697	single-stranded DNA binding	71	0.455074035	1.706840223	4.04E-04	0.007061861	0.005221	5184	tags=66%, list=41%, signal=39%
BP	GO:0048002	antigen processing and presentation of peptide antigen	143	0.392807984	1.561999923	4.09E-04	0.007125968	0.005268	5675	tags=62%, list=45%, signal=35%
BP	GO:0034329	cell junction assembly	216	0.362437314	1.47387583	4.12E-04	0.007164201	0.005297	4035	tags=43%, list=32%, signal=30%
MF	GO:0003713	transcription coactivator activity	186	0.380494767	1.536193965	4.17E-04	0.007230687	0.005346	5031	tags=56%, list=40%, signal=34%
BP	GO:0032204	regulation of telomere maintenance	58	0.477792387	1.74673485	4.28E-04	0.007398413	0.00547	3254	tags=45%, list=26%, signal=33%
BP	GO:0007596	blood coagulation	177	0.377110564	1.516528251	4.31E-04	0.007434383	0.005496	5146	tags=56%, list=41%, signal=34%
BP	GO:0060976	coronary vasculature development	30	0.568496454	1.856520123	4.46E-04	0.007656124	0.00566	3256	tags=63%, list=26%, signal=47%
BP	GO:0140056	organelle localization by membrane tethering	103	0.418632656	1.624690099	4.48E-04	0.00767336	0.005673	4841	tags=55%, list=38%, signal=34%
BP	GO:0035313	wound healing, spreading of epidermal cells	11	0.738594093	1.923205016	0.000451547	0.007716684	0.005705	2531	tags=82%, list=20%, signal=65%

BP	GO:0018212	peptidyl-tyrosine modification	206	0.37058011	1.501720093	0.00045797	0.00777983	0.005752	4071	tags=45%, list=32%, signal=31%
BP	GO:0030038	contractile actin filament bundle assembly	78	0.446271161	1.684442119	0.000458943	0.00777983	0.005752	3561	tags=50%, list=28%, signal=36%
BP	GO:0043149	stress fiber assembly	78	0.446271161	1.684442119	0.000458943	0.00777983	0.005752	3561	tags=50%, list=28%, signal=36%
BP	GO:0045744	negative regulation of G protein-coupled receptor signaling pathway	25	0.585611448	1.852848458	0.000466229	0.007835885	0.005793	3581	tags=68%, list=28%, signal=49%
BP	GO:0090670	RNA localization to Cajal body	18	0.646683903	1.900541894	0.000469955	0.007835885	0.005793	3365	tags=67%, list=27%, signal=49%
BP	GO:0090671	telomerase RNA localization to Cajal body	18	0.646683903	1.900541894	0.000469955	0.007835885	0.005793	3365	tags=67%, list=27%, signal=49%
BP	GO:0090672	telomerase RNA localization	18	0.646683903	1.900541894	0.000469955	0.007835885	0.005793	3365	tags=67%, list=27%, signal=49%
BP	GO:0090685	RNA localization to nucleus	18	0.646683903	1.900541894	0.000469955	0.007835885	0.005793	3365	tags=67%, list=27%, signal=49%
BP	GO:1904872	regulation of telomerase RNA localization to Cajal body	18	0.646683903	1.900541894	0.000469955	0.007835885	0.005793	3365	tags=67%, list=27%, signal=49%
BP	GO:0006635	fatty acid beta-oxidation	49	-0.359488414	-1.874343685	0.000470948	0.007835885	0.005793	2542	tags=43%, list=20%, signal=34%
BP	GO:0000723	telomere maintenance	106	0.41704631	1.623520307	0.000478862	0.007925728	0.00586	4219	tags=47%, list=33%, signal=32%
BP	GO:0032200	telomere organization	106	0.41704631	1.623520307	0.000478862	0.007925728	0.00586	4219	tags=47%, list=33%, signal=32%
BP	GO:0016126	sterol biosynthetic process	55	-0.335543808	-1.906948999	0.000480657	0.007934616	0.005866	1943	tags=36%, list=15%, signal=31%
BP	GO:0051098	regulation of binding	240	0.355509621	1.45056928	0.000490003	0.008067784	0.005965	4639	tags=49%, list=37%, signal=32%
BP	GO:0090504	epiboly	22	0.612664969	1.880984021	0.000491516	0.00807162	0.005967	2937	tags=68%, list=23%, signal=52%
BP	GO:0050778	positive regulation of immune response	371	0.333567258	1.380846469	0.000508829	0.008334223	0.006162	4902	tags=48%, list=39%, signal=30%
MF	GO:0004527	exonuclease activity	57	0.480061139	1.749090802	0.000518321	0.008467696	0.00626	5149	tags=61%, list=41%, signal=36%
BP	GO:0050921	positive regulation of chemotaxis	79	0.437096153	1.652009255	0.000519744	0.008469001	0.006261	2829	tags=38%, list=22%, signal=30%
MF	GO:0031435	mitogen-activated protein kinase kinase kinase binding	14	0.679313698	1.886327893	0.000534548	0.008669634	0.00641	2193	tags=57%, list=17%, signal=47%
MF	GO:0008146	sulfotransferase activity	24	0.593465508	1.859694064	0.000534806	0.008669634	0.00641	3745	tags=67%, list=30%, signal=47%
BP	GO:0006022	aminoglycan metabolic process	98	0.416712385	1.609366833	0.000536828	0.00868009	0.006417	3272	tags=44%, list=26%, signal=33%
BP	GO:0052126	movement in host environment	104	0.414075461	1.609834551	0.0005445	0.00878163	0.006492	3263	tags=44%, list=26%, signal=33%
MF	GO:0042826	histone deacetylase binding	85	0.436844651	1.65822345	0.000548959	0.008830952	0.006529	4900	tags=65%, list=39%, signal=40%
BP	GO:0035633	maintenance of blood-brain barrier	25	0.583157474	1.845084195	0.000552112	0.008859077	0.00655	2386	tags=52%, list=19%, signal=42%
CC	GO:0032154	cleavage furrow	32	0.554273372	1.818256502	0.000556706	0.008910117	0.006587	3370	tags=56%, list=27%, signal=41%
BP	GO:0007599	hemostasis	182	0.373092353	1.503770887	0.000573004	0.009147755	0.006763	5146	tags=55%, list=41%, signal=33%
MF	GO:0008168	methyltransferase activity	102	0.418896054	1.622677448	0.000602793	0.009599019	0.007097	4654	tags=54%, list=37%, signal=34%
CC	GO:0005657	replication fork	47	0.500772787	1.764934467	0.000605073	0.009611057	0.007106	4512	tags=64%, list=36%, signal=41%
BP	GO:0030855	epithelial cell differentiation	310	0.337773454	1.38853456	0.000609066	0.009650184	0.007135	2912	tags=34%, list=23%, signal=27%
BP	GO:0046580	negative regulation of Ras protein signal transduction	34	0.543517592	1.808403605	0.0006122	0.009675521	0.007153	3410	tags=56%, list=27%, signal=41%
BP	GO:0003179	heart valve morphogenesis	30	0.563928156	1.841601585	0.000622336	0.009811123	0.007253	2877	tags=53%, list=23%, signal=41%
BP	GO:0001937	negative regulation of endothelial cell proliferation	22	0.608742774	1.868942226	0.000632461	0.009945883	0.007353	3323	tags=64%, list=26%, signal=47%
BP	GO:0033044	regulation of chromosome organization	223	0.359501568	1.462257424	0.000636543	0.009985169	0.007382	4078	tags=44%, list=32%, signal=31%
BP	GO:0006260	DNA replication	166	0.374610818	1.502509176	0.000665348	0.010411131	0.007697	4411	tags=49%, list=35%, signal=32%
BP	GO:0031145	anaphase-promoting complex-dependent catabolic process	62	0.466302868	1.719163713	0.000667714	0.010422287	0.007705	3093	tags=45%, list=25%, signal=34%
CC	GO:0030118	clathrin coat	30	0.561473361	1.833585041	0.000672931	0.010477786	0.007746	1350	tags=43%, list=11%, signal=39%
BP	GO:1902905	positive regulation of supramolecular fiber organization	130	0.395986888	1.561316093	0.000679285	0.010528472	0.007784	4425	tags=48%, list=35%, signal=32%
BP	GO:0032210	regulation of telomere maintenance via telomerase	39	0.519802918	1.772796548	0.000680747	0.010528472	0.007784	3254	tags=49%, list=26%, signal=36%
MF	GO:0005509	calcium ion binding	285	0.342603696	1.402682089	0.000682336	0.010528472	0.007784	2784	tags=33%, list=22%, signal=26%

MF	GO:0030674	protein-macromolecule adaptor activity	161	0.379191051	1.519558512	0.000683702	0.010528472	0.007784	3764	tags=45%, list=30%, signal=32%
CC	GO:0070160	tight junction	69	0.448923613	1.675935753	0.000684534	0.010528472	0.007784	2757	tags=41%, list=22%, signal=32%
BP	GO:1904357	negative regulation of telomere maintenance via telomere lengthening	15	0.672266575	1.88659001	0.000687055	0.010541533	0.007793	3065	tags=67%, list=24%, signal=51%
CC	GO:0071013	catalytic step 2 spliceosome	67	0.460266197	1.71626678	0.000701972	0.010744259	0.007943	4716	tags=63%, list=37%, signal=39%
BP	GO:0051271	negative regulation of cellular component movement	194	0.366392575	1.480598983	0.000711234	0.010844283	0.008017	3293	tags=39%, list=26%, signal=29%
BP	GO:0038093	Fc receptor signaling pathway	143	0.386792768	1.538080433	0.000711946	0.010844283	0.008017	3626	tags=41%, list=29%, signal=29%
BP	GO:0070198	protein localization to chromosome, telomeric region	22	0.606202366	1.861142747	0.00071609	0.010854967	0.008025	2801	tags=55%, list=22%, signal=43%
BP	GO:2001258	negative regulation of cation channel activity	22	0.60621809	1.861191022	0.00071609	0.010854967	0.008025	2168	tags=55%, list=17%, signal=45%
BP	GO:0001837	epithelial to mesenchymal transition	85	0.433298703	1.644763347	0.000717865	0.010855777	0.008026	3443	tags=49%, list=27%, signal=36%
BP	GO:0043122	regulation of I-kappaB kinase/NF-kappaB signaling	165	0.369895244	1.484651849	0.000720411	0.010868205	0.008035	5369	tags=58%, list=43%, signal=34%
BP	GO:0002064	epithelial cell development	121	0.396980505	1.554718716	0.000727695	0.010951895	0.008097	2452	tags=36%, list=19%, signal=29%
BP	GO:0009168	purine ribonucleoside monophosphate biosynthetic process	15	0.67140392	1.884169132	0.000731026	0.01097583	0.008115	1245	tags=47%, list=10%, signal=42%
BP	GO:0000075	cell cycle checkpoint	134	0.391925447	1.54785363	0.000743229	0.011125047	0.008225	4866	tags=52%, list=39%, signal=32%
BP	GO:0030833	regulation of actin filament polymerization	101	0.413994847	1.603017287	0.000744492	0.011125047	0.008225	3279	tags=43%, list=26%, signal=32%
MF	GO:0045309	protein phosphorylated amino acid binding	39	0.517254139	1.764103893	0.00074931	0.011170569	0.008259	4044	tags=59%, list=32%, signal=40%
MF	GO:0004842	ubiquitin-protein transferase activity	277	0.343138566	1.403116956	0.000755606	0.011174175	0.008261	4912	tags=52%, list=39%, signal=32%
BP	GO:0010948	negative regulation of cell cycle process	227	0.358059757	1.457827343	0.000755606	0.011174175	0.008261	5235	tags=52%, list=42%, signal=31%
BP	GO:0003272	endocardial cushion formation	13	0.699360569	1.914257582	0.00075664	0.011174175	0.008261	1515	tags=62%, list=12%, signal=54%
MF	GO:0003688	DNA replication origin binding	13	0.697628906	1.909517753	0.00075664	0.011174175	0.008261	3794	tags=92%, list=30%, signal=65%
MF	GO:0051015	actin filament binding	114	0.397448455	1.552020125	0.000764002	0.011215368	0.008292	2850	tags=37%, list=23%, signal=29%
BP	GO:0010563	negative regulation of phosphorus metabolic process	334	0.336546541	1.389599551	0.000764765	0.011215368	0.008292	4256	tags=43%, list=34%, signal=29%
BP	GO:0045936	negative regulation of phosphate metabolic process	334	0.336546541	1.389599551	0.000764765	0.011215368	0.008292	4256	tags=43%, list=34%, signal=29%
BP	GO:0045931	positive regulation of mitotic cell cycle	96	0.419106143	1.612990981	0.000780357	0.011417468	0.008441	5309	tags=62%, list=42%, signal=36%
BP	GO:0045862	positive regulation of proteolysis	231	0.351764519	1.432962797	0.000783083	0.01143083	0.008451	3537	tags=38%, list=28%, signal=28%
BP	GO:0040013	negative regulation of locomotion	191	0.367132431	1.483437166	0.000802201	0.011682869	0.008637	3293	tags=38%, list=26%, signal=29%
MF	GO:0016796	exonuclease activity, active with either ribo- or deoxyribonucleic acids and producing 5'-phosphomonoesters	43	0.492753733	1.712771488	0.000817001	0.011870985	0.008776	5081	tags=60%, list=40%, signal=36%
BP	GO:1905114	cell surface receptor signaling pathway involved in cell-cell signaling	348	0.329714123	1.363184462	0.000819718	0.011878229	0.008782	3791	tags=38%, list=30%, signal=27%
BP	GO:0006352	DNA-templated transcription, initiation	159	0.376491094	1.506890223	0.000821359	0.011878229	0.008782	4115	tags=47%, list=33%, signal=32%
BP	GO:0010596	negative regulation of endothelial cell migration	35	0.524299592	1.755238621	0.00082315	0.011878229	0.008782	3293	tags=51%, list=26%, signal=38%
BP	GO:0043161	proteasome-mediated ubiquitin-dependent protein catabolic process	315	0.333705798	1.372813017	0.000828876	0.011925602	0.008817	4482	tags=45%, list=36%, signal=30%
BP	GO:0085029	extracellular matrix assembly	24	0.584123557	1.830419959	0.000830216	0.011925602	0.008817	2847	tags=54%, list=23%, signal=42%
MF	GO:0046982	protein heterodimerization activity	137	0.386318467	1.529181922	0.000833037	0.01193894	0.008827	4812	tags=56%, list=38%, signal=35%
BP	GO:1901988	negative regulation of cell cycle phase transition	171	0.377977629	1.515284735	0.00084889	0.012138549	0.008974	3398	tags=39%, list=27%, signal=29%
BP	GO:0006023	aminoglycan biosynthetic process	70	0.441016976	1.653154182	0.000851853	0.012153365	0.008985	3272	tags=47%, list=26%, signal=35%
BP	GO:0006278	RNA-dependent DNA biosynthetic process	52	0.472179599	1.69292521	0.000859201	0.012230527	0.009042	3365	tags=44%, list=27%, signal=33%
CC	GO:0001726	ruffle	124	0.399019479	1.568310744	0.000873364	0.012404126	0.009171	3044	tags=40%, list=24%, signal=30%
MF	GO:0016836	hydro-lyase activity	30	-0.423919985	-2.015385866	0.000876301	0.012417876	0.009181	850	tags=43%, list=7%, signal=41%
BP	GO:0006909	phagocytosis	169	0.378629844	1.51775691	0.000885598	0.012521487	0.009257	4668	tags=50%, list=37%, signal=32%

BP	GO:0032271	regulation of protein polymerization	139	0.392174404	1.551588916	0.000898373	0.012673685	0.00937	3437	tags=40%, list=27%, signal=30%
MF	GO:0019787	ubiquitin-like protein transferase activity	294	0.34155044	1.399924061	0.000911305	0.012827435	0.009483	4912	tags=52%, list=39%, signal=32%
BP	GO:0007004	telomere maintenance via telomerase	51	0.485514215	1.736650649	0.00093895	0.013187128	0.009749	3365	tags=45%, list=27%, signal=33%
CC	GO:0005923	bicellular tight junction	64	0.452529149	1.675481915	0.0009414	0.013192151	0.009753	2757	tags=41%, list=22%, signal=32%
BP	GO:0150116	regulation of cell-substrate junction organization	47	0.492099461	1.734366009	0.000943908	0.013197966	0.009757	2611	tags=47%, list=21%, signal=37%
BP	GO:0000209	protein polyubiquitination	248	0.344209042	1.405132409	0.000948031	0.013223508	0.009776	4538	tags=46%, list=36%, signal=30%
BP	GO:0140014	mitotic nuclear division	162	0.380564575	1.525678638	0.000949928	0.013223508	0.009776	4779	tags=51%, list=38%, signal=32%
BP	GO:0060317	cardiac epithelial to mesenchymal transition	20	0.614982509	1.847950048	0.00096201	0.013361576	0.009878	2877	tags=60%, list=23%, signal=46%
BP	GO:0000083	regulation of transcription involved in G1/S transition of mitotic cell cycle	14	0.661258962	1.836193248	0.000964184	0.013361576	0.009878	1518	tags=57%, list=12%, signal=50%
BP	GO:1902600	proton transmembrane transport	77	-0.289137461	-1.669445618	0.000966203	0.013361576	0.009878	1193	tags=22%, list=9%, signal=20%
MF	GO:0042393	histone binding	145	0.383551453	1.526996659	0.000970316	0.013389083	0.009899	6406	tags=70%, list=51%, signal=35%
MF	GO:0019825	oxygen binding	13	0.691541046	1.892854342	0.000977553	0.013459502	0.009951	810	tags=38%, list=6%, signal=36%
BP	GO:0060322	head development	427	0.319404652	1.326673722	0.000984846	0.01353037	0.010003	4464	tags=42%, list=35%, signal=28%
BP	GO:1902622	regulation of neutrophil migration	22	0.598059986	1.836144278	0.000987887	0.013542642	0.010012	2570	tags=45%, list=20%, signal=36%
BP	GO:0050730	regulation of peptidyl-tyrosine phosphorylation	138	0.38780437	1.53373708	0.000991782	0.01356654	0.01003	3319	tags=39%, list=26%, signal=29%
BP	GO:0033209	tumor necrosis factor-mediated signaling pathway	116	0.401300034	1.571520365	0.000994784	0.013568143	0.010031	5235	tags=59%, list=42%, signal=35%
BP	GO:2000209	regulation of anoikis	18	0.626076894	1.839979871	0.000997915	0.013568143	0.010031	2611	tags=50%, list=21%, signal=40%
BP	GO:0048525	negative regulation of viral process	64	0.451490129	1.671634958	0.000998354	0.013568143	0.010031	3323	tags=48%, list=26%, signal=36%
BP	GO:1901532	regulation of hematopoietic progenitor cell differentiation	73	0.445414331	1.671158656	0.001014229	0.013754259	0.010169	3093	tags=44%, list=25%, signal=33%
BP	GO:0048844	artery morphogenesis	37	0.528726968	1.785479799	0.001018217	0.013778709	0.010187	4423	tags=73%, list=35%, signal=48%
CC	GO:0000776	kinetochore	78	0.436588044	1.647893377	0.001025799	0.013821983	0.010219	4080	tags=53%, list=32%, signal=36%
BP	GO:0030856	regulation of epithelial cell differentiation	79	0.43034228	1.626482923	0.001025799	0.013821983	0.010219	2898	tags=39%, list=23%, signal=30%
BP	GO:0045785	positive regulation of cell adhesion	244	0.350685111	1.431065476	0.00104007	0.013974531	0.010332	2898	tags=34%, list=23%, signal=26%
BP	GO:0003198	epithelial to mesenchymal transition involved in endocardial cushion formation	10	0.733240222	1.863419274	0.001041552	0.013974531	0.010332	1515	tags=70%, list=12%, signal=62%
MF	GO:0005201	extracellular matrix structural constituent	91	0.425161982	1.62854555	0.001062357	0.014223401	0.010516	3034	tags=41%, list=24%, signal=31%
BP	GO:0051495	positive regulation of cytoskeleton organization	141	0.389470149	1.545201162	0.001081206	0.014445092	0.010679	3437	tags=40%, list=27%, signal=29%
BP	GO:0042176	regulation of protein catabolic process	271	0.344062203	1.405782123	0.001095293	0.014571557	0.010773	5213	tags=55%, list=41%, signal=33%
BP	GO:0048285	organelle fission	229	0.350207333	1.426287904	0.001095293	0.014571557	0.010773	4594	tags=47%, list=36%, signal=30%
BP	GO:0044764	multi-organism cellular process	10	0.730747901	1.857085417	0.001100397	0.014608634	0.0108	1350	tags=60%, list=11%, signal=54%
CC	GO:0032153	cell division site	38	0.509426836	1.729498681	0.00111111	0.014689969	0.01086	3370	tags=53%, list=27%, signal=39%
BP	GO:0048013	ephrin receptor signaling pathway	64	0.450162424	1.666719152	0.001112448	0.014689969	0.01086	4550	tags=59%, list=36%, signal=38%
BP	GO:0045930	negative regulation of mitotic cell cycle	217	0.35335845	1.436718988	0.001113701	0.014689969	0.01086	3398	tags=37%, list=27%, signal=28%
BP	GO:0038179	neurotrophin signaling pathway	24	0.577712706	1.810330802	0.001115841	0.014689969	0.01086	2823	tags=46%, list=22%, signal=36%
BP	GO:0043393	regulation of protein binding	136	0.389086789	1.53972168	0.001120417	0.014719475	0.010882	4520	tags=54%, list=36%, signal=35%
BP	GO:0031345	negative regulation of cell projection organization	113	0.402197829	1.568665992	0.001127214	0.014777987	0.010926	3532	tags=45%, list=28%, signal=33%
BP	GO:0051028	mRNA transport	106	0.408299343	1.589469224	0.001130644	0.014792197	0.010936	4890	tags=54%, list=39%, signal=33%
BP	GO:0060973	cell migration involved in heart development	10	0.730650344	1.85683749	0.001135703	0.014806154	0.010946	1441	tags=60%, list=11%, signal=53%
BP	GO:0007088	regulation of mitotic nuclear division	93	0.420489587	1.61325933	0.001136406	0.014806154	0.010946	4576	tags=53%, list=36%, signal=34%
BP	GO:0032465	regulation of cytokinesis	49	0.481009419	1.713849366	0.001166124	0.015162018	0.011209	4598	tags=63%, list=36%, signal=40%

BP	GO:0045666	positive regulation of neuron differentiation	203	0.366358919	1.484509373	0.001168925	0.015166315	0.011213	3857	tags=42%, list=31%, signal=30%
CC	GO:0031253	cell projection membrane	170	0.370206538	1.484426747	0.001171265	0.015166315	0.011213	3095	tags=35%, list=25%, signal=27%
BP	GO:0060411	cardiac septum morphogenesis	42	0.502555734	1.745470082	0.0011822	0.015276544	0.011294	2877	tags=50%, list=23%, signal=39%
BP	GO:0048011	neurotrophin TRK receptor signaling pathway	19	0.619511699	1.847410216	0.001204988	0.015464749	0.011433	2823	tags=47%, list=22%, signal=37%
BP	GO:0061437	renal system vasculature development	19	0.619917724	1.848621001	0.001204988	0.015464749	0.011433	1285	tags=42%, list=10%, signal=38%
BP	GO:0061440	kidney vasculature development	19	0.619917724	1.848621001	0.001204988	0.015464749	0.011433	1285	tags=42%, list=10%, signal=38%
BP	GO:2000146	negative regulation of cell motility	174	0.37283577	1.496433024	0.001208154	0.015464749	0.011433	3293	tags=39%, list=26%, signal=29%
BP	GO:1903052	positive regulation of proteolysis involved in cellular protein catabolic process	94	0.414311125	1.593936298	0.001209074	0.015464749	0.011433	3910	tags=49%, list=31%, signal=34%
BP	GO:0003229	ventricular cardiac muscle tissue development	25	0.560173233	1.772363082	0.001211479	0.015464749	0.011433	1441	tags=40%, list=11%, signal=35%
BP	GO:0001709	cell fate determination	15	0.66028387	1.852962796	0.001226239	0.015621543	0.011549	1585	tags=47%, list=13%, signal=41%
MF	GO:1990782	protein tyrosine kinase binding	56	0.468348567	1.702947195	0.001234185	0.015642102	0.011564	3762	tags=52%, list=30%, signal=36%
BP	GO:0001708	cell fate specification	27	0.551144673	1.76995581	0.001235936	0.015642102	0.011564	1562	tags=33%, list=12%, signal=29%
BP	GO:0000724	double-strand break repair via homologous recombination	70	0.435321422	1.631804374	0.001239164	0.015642102	0.011564	4614	tags=57%, list=37%, signal=36%
BP	GO:0000725	recombinational repair	70	0.435321422	1.631804374	0.001239164	0.015642102	0.011564	4614	tags=57%, list=37%, signal=36%
BP	GO:1903364	positive regulation of cellular protein catabolic process	110	0.402389405	1.569230935	0.001240255	0.015642102	0.011564	3947	tags=48%, list=31%, signal=33%
BP	GO:0046822	regulation of nucleocytoplasmic transport	81	0.428089666	1.619071352	0.001266597	0.015942442	0.011786	3721	tags=47%, list=30%, signal=33%
BP	GO:0060333	interferon-gamma-mediated signaling pathway	65	0.450306119	1.676804572	0.00129194	0.016229026	0.011998	4726	tags=57%, list=37%, signal=36%
BP	GO:0051704	multi-organism process	457	0.317256174	1.319844793	0.00129778	0.01626998	0.012029	4553	tags=45%, list=36%, signal=30%
BP	GO:0050808	synapse organization	199	0.353779067	1.430928843	0.001300378	0.016270206	0.012029	4394	tags=48%, list=35%, signal=32%
MF	GO:0031434	mitogen-activated protein kinase kinase binding	10	0.725323887	1.843301105	0.001312816	0.016369286	0.012102	3082	tags=70%, list=24%, signal=53%
BP	GO:0043535	regulation of blood vessel endothelial cell migration	66	0.448134917	1.668619558	0.001313489	0.016369286	0.012102	2690	tags=41%, list=21%, signal=32%
CC	GO:0034708	methyltransferase complex	73	0.441138644	1.655116625	0.001317543	0.016387422	0.012115	4948	tags=62%, list=39%, signal=38%
CC	GO:0016363	nuclear matrix	80	0.424562338	1.604004685	0.001325589	0.016455053	0.012165	4939	tags=61%, list=39%, signal=37%
CC	GO:0097447	dendritic tree	297	0.334029833	1.370460973	0.001334596	0.016534304	0.012224	4423	tags=46%, list=35%, signal=31%
BP	GO:0010470	regulation of gastrulation	20	0.607579399	1.825704574	0.00134185	0.016591585	0.012266	2690	tags=55%, list=21%, signal=43%
MF	GO:0046875	ephrin receptor binding	22	0.588458212	1.806665225	0.001355211	0.016697504	0.012345	2823	tags=55%, list=22%, signal=42%
BP	GO:0045088	regulation of innate immune response	194	0.36088296	1.458334528	0.001355712	0.016697504	0.012345	5271	tags=55%, list=42%, signal=33%
MF	GO:0003727	single-stranded RNA binding	58	0.461251198	1.686262827	0.00138582	0.017035048	0.012594	3512	tags=55%, list=28%, signal=40%
BP	GO:0001701	in utero embryonic development	235	0.346306753	1.411257266	0.001389819	0.017050976	0.012606	3179	tags=37%, list=25%, signal=28%
BP	GO:2001257	regulation of cation channel activity	63	0.452332573	1.673389427	0.001413783	0.017311288	0.012798	2168	tags=35%, list=17%, signal=29%
BP	GO:0007043	cell-cell junction assembly	79	0.424462395	1.604259841	0.001421051	0.01736657	0.012839	3263	tags=41%, list=26%, signal=30%
BP	GO:2000811	negative regulation of anoikis	15	0.655460652	1.839427341	0.001425084	0.017382162	0.012851	2611	tags=60%, list=21%, signal=48%
BP	GO:0050878	regulation of body fluid levels	268	0.340788405	1.391704852	0.001445043	0.017557694	0.012981	2445	tags=29%, list=19%, signal=24%
BP	GO:0051051	negative regulation of transport	247	0.342033797	1.396532481	0.001445043	0.017557694	0.012981	2636	tags=32%, list=21%, signal=25%
BP	GO:0031032	actomyosin structure organization	126	0.385574091	1.518593292	0.00145522	0.017647337	0.013047	3561	tags=44%, list=28%, signal=32%
BP	GO:0030203	glycosaminoglycan metabolic process	92	0.424071368	1.624449929	0.001458695	0.017655524	0.013053	3272	tags=45%, list=26%, signal=33%
BP	GO:0008277	regulation of G protein-coupled receptor signaling pathway	65	0.448800378	1.671197647	0.001461681	0.017657774	0.013055	3608	tags=46%, list=29%, signal=33%
BP	GO:0050769	positive regulation of neurogenesis	264	0.340278865	1.389516306	0.001464915	0.017663006	0.013058	4450	tags=44%, list=35%, signal=29%
MF	GO:0033613	activating transcription factor binding	53	0.46173167	1.659817112	0.00149067	0.017939248	0.013263	4828	tags=60%, list=38%, signal=37%

BP	GO:0150115	cell-substrate junction organization	71	0.435281216	1.632603555	0.001500937	0.0180284	0.013329	2611	tags=39%, list=21%, signal=31%
BP	GO:0051701	interaction with host	134	0.383521263	1.514662505	0.001512354	0.018130996	0.013404	4397	tags=49%, list=35%, signal=32%
BP	GO:0050678	regulation of epithelial cell proliferation	191	0.360600378	1.457043719	0.00153862	0.01841089	0.013611	2965	tags=34%, list=24%, signal=26%
CC	GO:0044853	plasma membrane raft	52	0.464087689	1.663912948	0.001572531	0.018781028	0.013885	3293	tags=44%, list=26%, signal=33%
MF	GO:0045296	cadherin binding	233	0.347434631	1.415891481	0.001592307	0.018981262	0.014033	3060	tags=36%, list=24%, signal=27%
BP	GO:1905564	positive regulation of vascular endothelial cell proliferation	11	0.700582424	1.824227469	0.001614945	0.019214797	0.014206	2241	tags=55%, list=18%, signal=45%
BP	GO:0032606	type I interferon production	97	0.400023819	1.543958796	0.001641879	0.019492916	0.014411	5397	tags=61%, list=43%, signal=35%
BP	GO:0043928	exonucleolytic catabolism of deadenylated mRNA	28	0.544856	1.762648456	0.001644502	0.019492916	0.014411	4811	tags=71%, list=38%, signal=44%
BP	GO:0010833	telomere maintenance via telomere lengthening	57	0.4588999	1.671990356	0.001653422	0.019531228	0.01444	3365	tags=44%, list=27%, signal=32%
BP	GO:0001706	endoderm formation	26	0.551921559	1.76491999	0.001653929	0.019531228	0.01444	2986	tags=54%, list=24%, signal=41%
BP	GO:0035909	aorta morphogenesis	18	0.611802893	1.798029953	0.001683921	0.019848238	0.014674	3092	tags=72%, list=25%, signal=55%
BP	GO:0046847	filopodium assembly	32	0.525643643	1.724338602	0.001703552	0.020042162	0.014817	2626	tags=47%, list=21%, signal=37%
BP	GO:0034440	lipid oxidation	69	-0.283815108	-1.604546134	0.001712647	0.020111643	0.014869	2542	tags=41%, list=20%, signal=33%
CC	GO:0035770	ribonucleoprotein granule	161	0.368620903	1.477200027	0.001724609	0.020189996	0.014927	4427	tags=49%, list=35%, signal=32%
CC	GO:0000784	nuclear chromosome, telomeric region	65	0.446547789	1.662809681	0.001725723	0.020189996	0.014927	5397	tags=66%, list=43%, signal=38%
BP	GO:0002693	positive regulation of cellular extravasation	11	0.697830874	1.817062783	0.001731126	0.020215705	0.014946	2254	tags=64%, list=18%, signal=52%
BP	GO:0045859	regulation of protein kinase activity	484	0.309947145	1.291874785	0.001739571	0.020276772	0.014991	4253	tags=40%, list=34%, signal=27%
CC	GO:0005884	actin filament	73	0.434925769	1.631806418	0.001772514	0.02060897	0.015236	5182	tags=60%, list=41%, signal=36%
BP	GO:0043276	anoikis	26	0.550325311	1.75981555	0.001775689	0.02060897	0.015236	2611	tags=42%, list=21%, signal=34%
BP	GO:0045732	positive regulation of protein catabolic process	153	0.375889868	1.503384367	0.001778163	0.02060897	0.015236	4092	tags=48%, list=32%, signal=33%
MF	GO:0001102	RNA polymerase II activating transcription factor binding	32	0.524262049	1.719806375	0.001784193	0.02060897	0.015236	2424	tags=44%, list=19%, signal=35%
BP	GO:0006270	DNA replication initiation	24	0.56648268	1.775140192	0.001791473	0.02060897	0.015236	4411	tags=79%, list=35%, signal=52%
BP	GO:1901889	negative regulation of cell junction assembly	16	0.627647994	1.792047992	0.001792681	0.02060897	0.015236	2611	tags=50%, list=21%, signal=40%
MF	GO:0008270	zinc ion binding	438	0.316868795	1.317577367	0.001794795	0.02060897	0.015236	4502	tags=44%, list=36%, signal=29%
BP	GO:0010498	proteasomal protein catabolic process	350	0.327161525	1.352870256	0.001794795	0.02060897	0.015236	4482	tags=45%, list=36%, signal=30%
BP	GO:2000060	positive regulation of ubiquitin-dependent protein catabolic process	78	0.428133599	1.615982232	0.001797484	0.02060897	0.015236	3910	tags=50%, list=31%, signal=35%
BP	GO:0051972	regulation of telomerase activity	36	0.501309467	1.684878113	0.001814888	0.020770754	0.015356	3598	tags=50%, list=29%, signal=36%
BP	GO:0002431	Fc receptor mediated stimulatory signaling pathway	65	0.445136653	1.657555037	0.001820024	0.020791792	0.015372	4746	tags=57%, list=38%, signal=36%
BP	GO:0018108	peptidyl-tyrosine phosphorylation	203	0.362539283	1.469031968	0.001831611	0.020886324	0.015442	4247	tags=45%, list=34%, signal=31%
BP	GO:0031334	positive regulation of protein-containing complex assembly	151	0.373726427	1.491264755	0.001838959	0.020932269	0.015475	4221	tags=44%, list=33%, signal=29%
CC	GO:0009986	cell surface	356	0.325681915	1.347462339	0.001868427	0.021229367	0.015695	2676	tags=31%, list=21%, signal=25%
MF	GO:0005085	guanyl-nucleotide exchange factor activity	126	0.382750834	1.507473822	0.001881585	0.021340419	0.015777	3591	tags=42%, list=28%, signal=30%
CC	GO:0031143	pseudopodium	11	0.696541854	1.813706338	0.00189378	0.021426328	0.015841	3106	tags=73%, list=25%, signal=55%
BP	GO:0099003	vesicle-mediated transport in synapse	104	0.397143074	1.544005146	0.001898777	0.021426328	0.015841	4723	tags=50%, list=37%, signal=32%
CC	GO:0043296	apical junction complex	74	0.429923848	1.615144554	0.001899353	0.021426328	0.015841	2802	tags=39%, list=22%, signal=31%
BP	GO:2000736	regulation of stem cell differentiation	86	0.414146544	1.573982299	0.001954231	0.022006037	0.016269	3093	tags=41%, list=25%, signal=31%
BP	GO:0033631	cell-cell adhesion mediated by integrin	10	0.708883256	1.801519725	0.001975122	0.022201641	0.016414	1663	tags=60%, list=13%, signal=52%
CC	GO:0000932	P-body	60	0.45524862	1.668822585	0.002014885	0.022585295	0.016698	4902	tags=63%, list=39%, signal=39%
BP	GO:0051962	positive regulation of nervous system development	279	0.332249724	1.358794235	0.002016416	0.022585295	0.016698	4450	tags=44%, list=35%, signal=29%

BP	GO:0090305	nucleic acid phosphodiester bond hydrolysis	199	0.347463208	1.405383111	0.002020453	0.022590386	0.016701	4201	tags=43%, list=33%, signal=29%
MF	GO:0008408	3'-5' exonuclease activity	40	0.493005961	1.686591254	0.0020334	0.022694907	0.016779	5081	tags=65%, list=40%, signal=39%
BP	GO:0010721	negative regulation of cell development	190	0.352731131	1.424324449	0.002055467	0.022900664	0.016931	4508	tags=49%, list=36%, signal=32%
BP	GO:0007156	homophilic cell adhesion via plasma membrane adhesion molecules	46	0.470617696	1.651602953	0.002061892	0.022931727	0.016954	1158	tags=30%, list=9%, signal=28%
BP	GO:0015931	nucleobase-containing compound transport	181	0.3575401	1.439828444	0.002129536	0.023642349	0.017479	4966	tags=51%, list=39%, signal=32%
BP	GO:0050684	regulation of mRNA processing	92	0.418154584	1.601785066	0.002137387	0.023687804	0.017513	5439	tags=63%, list=43%, signal=36%
BP	GO:0042692	muscle cell differentiation	186	0.361202383	1.458303685	0.002166571	0.023969112	0.017721	4421	tags=47%, list=35%, signal=31%
BP	GO:0098813	nuclear chromosome segregation	135	0.380919183	1.505731145	0.002179648	0.024071564	0.017796	4779	tags=50%, list=38%, signal=31%
BP	GO:0006367	transcription initiation from RNA polymerase II promoter	129	0.37780918	1.488643229	0.002184043	0.024077927	0.017801	4115	tags=48%, list=33%, signal=33%
BP	GO:0048714	positive regulation of oligodendrocyte differentiation	11	0.69214399	1.802254861	0.002195852	0.024165865	0.017866	2203	tags=73%, list=17%, signal=60%
BP	GO:0007420	brain development	403	0.316658529	1.314129754	0.002201404	0.024184758	0.01788	4247	tags=40%, list=34%, signal=27%
BP	GO:0044782	cilium organization	175	0.358656675	1.440182216	0.002205811	0.024191031	0.017885	4841	tags=49%, list=38%, signal=31%
BP	GO:0003209	cardiac atrium morphogenesis	12	0.678635879	1.821272061	0.002218846	0.02429174	0.017959	2877	tags=67%, list=23%, signal=52%
BP	GO:0016055	Wnt signaling pathway	310	0.326407055	1.341809048	0.002238401	0.024421034	0.018055	3790	tags=38%, list=30%, signal=28%
BP	GO:0198738	cell-cell signaling by wnt	310	0.326407055	1.341809048	0.002238401	0.024421034	0.018055	3790	tags=38%, list=30%, signal=28%
BP	GO:0032211	negative regulation of telomere maintenance via telomerase	13	0.664660158	1.819277211	0.002253368	0.024541859	0.018144	3065	tags=69%, list=24%, signal=52%
BP	GO:0048713	regulation of oligodendrocyte differentiation	23	0.561367303	1.745907276	0.002260283	0.024574732	0.018168	2407	tags=52%, list=19%, signal=42%
BP	GO:0051258	protein polymerization	181	0.356071484	1.433914271	0.002277674	0.024721192	0.018277	4668	tags=48%, list=37%, signal=31%
BP	GO:0050765	negative regulation of phagocytosis	12	0.67676511	1.816251433	0.002331699	0.025220741	0.018646	838	tags=50%, list=7%, signal=47%
BP	GO:0060445	branching involved in salivary gland morphogenesis	12	0.676449894	1.81540548	0.002331699	0.025220741	0.018646	715	tags=50%, list=6%, signal=47%
BP	GO:0030100	regulation of endocytosis	120	0.389514407	1.526639323	0.00233573	0.025221086	0.018646	3406	tags=42%, list=27%, signal=31%
BP	GO:0072012	glomerulus vasculature development	17	0.595704708	1.731897502	0.002347015	0.025299615	0.018704	2100	tags=47%, list=17%, signal=39%
MF	GO:0003729	mRNA binding	205	0.355384242	1.439280846	0.002351743	0.025307325	0.01871	4811	tags=52%, list=38%, signal=33%
BP	GO:0071825	protein-lipid complex subunit organization	24	0.559030981	1.751789417	0.002366151	0.025418993	0.018793	2311	tags=42%, list=18%, signal=34%
BP	GO:0051054	positive regulation of DNA metabolic process	119	0.386536674	1.512923027	0.002373101	0.025450298	0.018816	4157	tags=48%, list=33%, signal=32%
BP	GO:1904063	negative regulation of cation transmembrane transport	44	0.475596325	1.657279056	0.002381126	0.025493006	0.018847	2168	tags=41%, list=17%, signal=34%
BP	GO:0030111	regulation of Wnt signaling pathway	221	0.344526128	1.400767511	0.002386391	0.025506074	0.018857	5235	tags=52%, list=42%, signal=31%
BP	GO:0090092	regulation of transmembrane receptor protein serine/threonine kinase signaling pathway	125	0.380203149	1.495814428	0.002408042	0.025693931	0.018996	2910	tags=38%, list=23%, signal=29%
BP	GO:0043086	negative regulation of catalytic activity	436	0.314864635	1.309069104	0.002423389	0.025814004	0.019085	4975	tags=48%, list=39%, signal=30%
BP	GO:0010743	regulation of macrophage derived foam cell differentiation	19	0.601954646	1.795054339	0.002464741	0.026210211	0.019378	2295	tags=47%, list=18%, signal=39%
BP	GO:0101023	vascular endothelial cell proliferation	13	0.661669171	1.811090421	0.002476466	0.026246382	0.019404	2241	tags=54%, list=18%, signal=44%
BP	GO:1905562	regulation of vascular endothelial cell proliferation	13	0.661669171	1.811090421	0.002476466	0.026246382	0.019404	2241	tags=54%, list=18%, signal=44%
BP	GO:0001569	branching involved in blood vessel morphogenesis	20	0.590095192	1.77316659	0.002484599	0.026277367	0.019427	2877	tags=60%, list=23%, signal=46%
BP	GO:0000819	sister chromatid segregation	114	0.381572214	1.49002405	0.002487724	0.026277367	0.019427	5911	tags=63%, list=47%, signal=34%
BP	GO:0051893	regulation of focal adhesion assembly	44	0.474709828	1.654189939	0.002499196	0.026310397	0.019452	2611	tags=45%, list=21%, signal=36%
BP	GO:0090109	regulation of cell-substrate junction assembly	44	0.474709828	1.654189939	0.002499196	0.026310397	0.019452	2611	tags=45%, list=21%, signal=36%
BP	GO:0097064	ncRNA export from nucleus	30	0.535178891	1.747716058	0.002513989	0.026345105	0.019477	4966	tags=83%, list=39%, signal=51%
BP	GO:0034035	purine ribonucleoside bisphosphate metabolic process	14	0.632732538	1.75698067	0.002515026	0.026345105	0.019477	1209	tags=50%, list=10%, signal=45%
BP	GO:0050427	3'-phosphoadenosine 5'-phosphosulfate metabolic process	14	0.632732538	1.75698067	0.002515026	0.026345105	0.019477	1209	tags=50%, list=10%, signal=45%

BP	GO:0048024	regulation of mRNA splicing, via spliceosome	66	0.436314015	1.624604715	0.002525997	0.026416146	0.01953	4266	tags=56%, list=34%, signal=37%
CC	GO:0030425	dendrite	296	0.334251038	1.370724166	0.002534382	0.026459952	0.019562	4423	tags=46%, list=35%, signal=31%
BP	GO:0007193	adenylate cyclase-inhibiting G protein-coupled receptor signaling pathway	31	0.518645242	1.696749831	0.002549239	0.026571072	0.019644	2850	tags=48%, list=23%, signal=38%
CC	GO:0030426	growth cone	110	0.393505594	1.534586012	0.002567729	0.026719634	0.019754	3821	tags=48%, list=30%, signal=34%
BP	GO:1902624	positive regulation of neutrophil migration	16	0.620036855	1.770316821	0.002598591	0.026996233	0.019959	2570	tags=50%, list=20%, signal=40%
BP	GO:0007063	regulation of sister chromatid cohesion	14	0.628527804	1.745304906	0.002604051	0.027008464	0.019968	2566	tags=50%, list=20%, signal=40%
MF	GO:0016757	transferase activity, transferring glycosyl groups	142	0.367475054	1.459142628	0.002616218	0.027090097	0.020028	3724	tags=42%, list=30%, signal=30%
BP	GO:0032479	regulation of type I interferon production	95	0.40061989	1.542345742	0.002621131	0.02709648	0.020033	5397	tags=61%, list=43%, signal=35%
BP	GO:0110020	regulation of actomyosin structure organization	70	0.423192591	1.586339394	0.00264266	0.027274326	0.020164	3561	tags=47%, list=28%, signal=34%
MF	GO:0000049	tRNA binding	36	0.493392424	1.658269296	0.002651073	0.027316449	0.020195	4439	tags=58%, list=35%, signal=38%
MF	GO:0070412	R-SMAD binding	16	0.618683091	1.766451581	0.002685936	0.027597944	0.020404	3732	tags=69%, list=30%, signal=48%
BP	GO:0060271	cilium assembly	171	0.362407504	1.45286524	0.002687742	0.027597944	0.020404	4841	tags=49%, list=38%, signal=31%
BP	GO:0010586	miRNA metabolic process	17	0.591452743	1.719535727	0.002691522	0.027597944	0.020404	1958	tags=47%, list=16%, signal=40%
BP	GO:1902807	negative regulation of cell cycle G1/S phase transition	77	0.417317557	1.574618971	0.002704821	0.02768929	0.020471	4866	tags=58%, list=39%, signal=36%
BP	GO:0000288	nuclear-transcribed mRNA catabolic process, deadenylation-dependent decay	55	0.446212455	1.615094134	0.002716946	0.02773101	0.020502	4852	tags=62%, list=38%, signal=38%
BP	GO:0001817	regulation of cytokine production	394	0.314425639	1.304158927	0.00271937	0.02773101	0.020502	5311	tags=49%, list=42%, signal=30%
CC	GO:0036464	cytoplasmic ribonucleoprotein granule	155	0.370239507	1.480521206	0.002722089	0.02773101	0.020502	4427	tags=50%, list=35%, signal=33%
BP	GO:0008015	blood circulation	239	0.340946083	1.39018677	0.002756368	0.028034926	0.020727	2492	tags=31%, list=20%, signal=25%
MF	GO:0008757	S-adenosylmethionine-dependent methyltransferase activity	81	0.414810533	1.568848546	0.002771985	0.028148369	0.02081	4654	tags=56%, list=37%, signal=35%
BP	GO:0072577	endothelial cell apoptotic process	34	0.509875711	1.696469605	0.002794735	0.028333758	0.020948	2440	tags=44%, list=19%, signal=36%
CC	GO:0043596	nuclear replication fork	30	0.532954298	1.740451277	0.002800128	0.02834287	0.020954	4411	tags=70%, list=35%, signal=46%
MF	GO:0048156	tau protein binding	31	0.515001666	1.684829856	0.002834747	0.028647304	0.021179	4597	tags=65%, list=36%, signal=41%
MF	GO:0005525	GTP binding	214	0.341431835	1.386793348	0.002867361	0.028930521	0.021389	4295	tags=44%, list=34%, signal=29%
CC	GO:0000777	condensed chromosome kinetochore	56	0.449454229	1.6342461	0.002903591	0.029160466	0.021559	5230	tags=66%, list=41%, signal=39%
BP	GO:0036498	IRE1-mediated unfolded protein response	56	0.449374136	1.633954874	0.002903591	0.029160466	0.021559	4375	tags=57%, list=35%, signal=37%
BP	GO:0098609	cell-cell adhesion	420	0.320508727	1.330843459	0.002904358	0.029160466	0.021559	3050	tags=34%, list=24%, signal=26%
BP	GO:1904874	positive regulation of telomerase RNA localization to Cajal body	15	0.633105135	1.776690778	0.002908648	0.029160466	0.021559	3365	tags=67%, list=27%, signal=49%
BP	GO:0090263	positive regulation of canonical Wnt signaling pathway	102	0.398227214	1.542612571	0.00296357	0.029636608	0.021911	3256	tags=39%, list=26%, signal=29%
MF	GO:0001972	retinoic acid binding	12	0.670639377	1.799811651	0.002965541	0.029636608	0.021911	1585	tags=50%, list=13%, signal=44%
BP	GO:0007608	sensory perception of smell	25	-0.426976136	-1.894256685	0.00298614	0.029795252	0.022028	1437	tags=40%, list=11%, signal=36%
BP	GO:0030177	positive regulation of Wnt signaling pathway	116	0.386918045	1.515199442	0.003008408	0.029948165	0.022141	3256	tags=39%, list=26%, signal=29%
BP	GO:0050900	leukocyte migration	228	0.344697345	1.403568136	0.003015351	0.029948165	0.022141	2829	tags=32%, list=22%, signal=25%
BP	GO:0071801	regulation of podosome assembly	10	0.693845036	1.76330236	0.003018926	0.029948165	0.022141	1652	tags=50%, list=13%, signal=43%
CC	GO:0005901	caveola	40	0.481377637	1.646810334	0.003020462	0.029948165	0.022141	3293	tags=50%, list=26%, signal=37%
MF	GO:0016620	oxidoreductase activity, acting on the aldehyde or oxo group of donors, NAD or NADP as acceptor	27	-0.405923919	-1.83421018	0.003033398	0.030029214	0.022201	2210	tags=52%, list=18%, signal=43%
BP	GO:2000045	regulation of G1/S transition of mitotic cell cycle	113	0.387175009	1.510073465	0.003051938	0.030122243	0.02227	4866	tags=55%, list=39%, signal=34%
CC	GO:0098793	presynapse	235	0.339003864	1.381496786	0.003052349	0.030122243	0.02227	4394	tags=46%, list=35%, signal=30%

BP	GO:0002479	antigen processing and presentation of exogenous peptide antigen via MHC class I, TAP-dependent	67	0.432365611	1.61222949	0.003064062	0.030190583	0.02232	4452	tags=55%, list=35%, signal=36%
MF	GO:0019001	guanyl nucleotide binding	217	0.345057263	1.402967218	0.003089347	0.030344893	0.022434	4295	tags=44%, list=34%, signal=30%
MF	GO:0032561	guanyl ribonucleotide binding	217	0.345057263	1.402967218	0.003089347	0.030344893	0.022434	4295	tags=44%, list=34%, signal=30%
CC	GO:0015030	Cajal body	43	0.470027521	1.633777043	0.003102735	0.030381749	0.022462	5358	tags=70%, list=43%, signal=40%
MF	GO:0050840	extracellular matrix binding	38	0.486883052	1.652962774	0.003102735	0.030381749	0.022462	2231	tags=42%, list=18%, signal=35%
CC	GO:0000151	ubiquitin ligase complex	192	0.351275793	1.420299962	0.003129471	0.030596036	0.02262	4399	tags=48%, list=35%, signal=32%
BP	GO:0002043	blood vessel endothelial cell proliferation involved in sprouting angiogenesis	14	0.621498864	1.725786844	0.003138204	0.030633919	0.022648	2937	tags=57%, list=23%, signal=44%
BP	GO:0009451	RNA modification	100	0.399831817	1.548287214	0.00315233	0.030724259	0.022715	5793	tags=67%, list=46%, signal=37%
BP	GO:0031146	SCF-dependent proteasomal ubiquitin-dependent protein catabolic process	70	0.420742749	1.57715615	0.003174986	0.030849952	0.022808	3145	tags=41%, list=25%, signal=31%
BP	GO:0048259	regulation of receptor-mediated endocytosis	54	0.456214225	1.646210821	0.003179398	0.030849952	0.022808	3406	tags=46%, list=27%, signal=34%
BP	GO:0002433	immune response-regulating cell surface receptor signaling pathway involved in phagocytosis	63	0.436623476	1.61527414	0.003184795	0.030849952	0.022808	4746	tags=56%, list=38%, signal=35%
BP	GO:0038096	Fc-gamma receptor signaling pathway involved in phagocytosis	63	0.436623476	1.61527414	0.003184795	0.030849952	0.022808	4746	tags=56%, list=38%, signal=35%
CC	GO:0000793	condensed chromosome	115	0.395297989	1.545986136	0.003195263	0.030903878	0.022848	5230	tags=58%, list=41%, signal=34%
BP	GO:0051090	regulation of DNA-binding transcription factor activity	256	0.330115166	1.348047818	0.00320034	0.030905581	0.022849	4886	tags=47%, list=39%, signal=30%
BP	GO:0050768	negative regulation of neurogenesis	165	0.351024136	1.408908714	0.003206747	0.030920103	0.02286	4508	tags=50%, list=36%, signal=32%
BP	GO:0032535	regulation of cellular component size	221	0.3404369	1.384141608	0.003237338	0.031151016	0.02303	3182	tags=35%, list=25%, signal=27%
CC	GO:0098552	side of membrane	264	0.330810082	1.350850876	0.003240575	0.031151016	0.02303	2822	tags=31%, list=22%, signal=24%
MF	GO:0003924	GTPase activity	184	0.351829828	1.42006145	0.00327761	0.031378416	0.023198	4464	tags=47%, list=35%, signal=31%
BP	GO:0003171	atrioventricular valve development	19	0.594408247	1.772550657	0.003279159	0.031378416	0.023198	2877	tags=63%, list=23%, signal=49%
BP	GO:0030947	regulation of vascular endothelial growth factor receptor signaling pathway	19	0.594212077	1.771965669	0.003279159	0.031378416	0.023198	3179	tags=63%, list=25%, signal=47%
CC	GO:0061695	transferase complex, transferring phosphorus-containing groups	172	0.352740615	1.413137716	0.003287472	0.031410303	0.023222	4201	tags=42%, list=33%, signal=29%
MF	GO:0017124	SH3 domain binding	81	0.412817098	1.561309208	0.003299975	0.031482058	0.023275	5335	tags=69%, list=42%, signal=40%
BP	GO:0071103	DNA conformation change	171	0.360540568	1.445380827	0.003317963	0.031605849	0.023367	3584	tags=40%, list=28%, signal=29%
BP	GO:2000134	negative regulation of G1/S transition of mitotic cell cycle	75	0.421218811	1.586879864	0.0033445	0.031709586	0.023443	4866	tags=59%, list=39%, signal=36%
BP	GO:0006409	tRNA export from nucleus	27	0.530727435	1.704387527	0.003346305	0.031709586	0.023443	4905	tags=81%, list=39%, signal=50%
BP	GO:0071431	tRNA-containing ribonucleoprotein complex export from nucleus	27	0.530727435	1.704387527	0.003346305	0.031709586	0.023443	4905	tags=81%, list=39%, signal=50%
BP	GO:0120254	olefinic compound metabolic process	54	-0.313077514	-1.758961354	0.003348967	0.031709586	0.023443	2129	tags=37%, list=17%, signal=31%
BP	GO:0006024	glycosaminoglycan biosynthetic process	67	0.431502893	1.609012537	0.003368561	0.031847295	0.023545	3639	tags=49%, list=29%, signal=35%
BP	GO:1902850	microtubule cytoskeleton organization involved in mitosis	89	0.409145463	1.565341001	0.003375402	0.031864198	0.023558	4864	tags=58%, list=39%, signal=36%
MF	GO:0008094	DNA-dependent ATPase activity	78	0.417887916	1.577310092	0.003385745	0.031901415	0.023585	3856	tags=49%, list=31%, signal=34%
BP	GO:0032481	positive regulation of type I interferon production	59	0.445721836	1.633704572	0.003389462	0.031901415	0.023585	5397	tags=66%, list=43%, signal=38%
BP	GO:0046579	positive regulation of Ras protein signal transduction	37	0.497534348	1.680144159	0.003397538	0.031901937	0.023586	2823	tags=54%, list=22%, signal=42%
BP	GO:0050680	negative regulation of epithelial cell proliferation	74	0.421800842	1.584627919	0.003399635	0.031901937	0.023586	3398	tags=42%, list=27%, signal=31%
BP	GO:0035051	cardiocyte differentiation	69	0.428690137	1.600399505	0.00341364	0.031977899	0.023642	3065	tags=43%, list=24%, signal=33%
MF	GO:0016922	nuclear receptor binding	64	0.434607826	1.609128501	0.003420686	0.031977899	0.023642	3728	tags=45%, list=30%, signal=32%
BP	GO:0043112	receptor metabolic process	111	0.38820256	1.514821401	0.003422944	0.031977899	0.023642	3406	tags=42%, list=27%, signal=31%
BP	GO:1903672	positive regulation of sprouting angiogenesis	28	0.529170392	1.711904382	0.003428421	0.031981695	0.023644	2406	tags=46%, list=19%, signal=38%
BP	GO:0019395	fatty acid oxidation	66	-0.281031788	-1.540543199	0.003450308	0.032138321	0.02376	2542	tags=41%, list=20%, signal=33%

MF	GO:0005547	phosphatidylinositol-3,4,5-trisphosphate binding	27	0.529435727	1.700239314	0.003510538	0.032651114	0.024139	1821	tags=33%, list=14%, signal=29%
BP	GO:0007249	I-kappaB kinase/NF-kappaB signaling	189	0.35186307	1.421255989	0.003573888	0.033191366	0.024539	5369	tags=56%, list=43%, signal=33%
BP	GO:0044409	entry into host	89	0.407298521	1.558274828	0.003601683	0.033394437	0.024689	3263	tags=44%, list=26%, signal=33%
BP	GO:1902806	regulation of cell cycle G1/S phase transition	123	0.379666175	1.489062823	0.003606345	0.033394437	0.024689	4866	tags=54%, list=39%, signal=33%
CC	GO:0017053	transcription repressor complex	56	0.443234914	1.611632248	0.003634287	0.033603833	0.024844	4450	tags=55%, list=35%, signal=36%
BP	GO:0009896	positive regulation of catabolic process	311	0.325357858	1.337475822	0.003644313	0.033647202	0.024876	4493	tags=45%, list=36%, signal=29%
CC	GO:0090575	RNA polymerase II transcription regulator complex	118	0.38137031	1.494464998	0.003673673	0.033868689	0.02504	5247	tags=58%, list=42%, signal=34%
BP	GO:0032886	regulation of microtubule-based process	131	0.372284067	1.467954688	0.00371471	0.034131876	0.025234	4839	tags=50%, list=38%, signal=31%
BP	GO:0051656	establishment of organelle localization	268	0.331010602	1.351774458	0.003718309	0.034131876	0.025234	3269	tags=35%, list=26%, signal=27%
BP	GO:0070507	regulation of microtubule cytoskeleton organization	115	0.392790812	1.53618072	0.003718458	0.034131876	0.025234	4839	tags=52%, list=38%, signal=32%
BP	GO:0000281	mitotic cytokinesis	48	0.452708077	1.602664571	0.003730858	0.034195914	0.025281	3370	tags=50%, list=27%, signal=37%
BP	GO:2000058	regulation of ubiquitin-dependent protein catabolic process	116	0.383465234	1.501677979	0.00375583	0.034374835	0.025414	4055	tags=47%, list=32%, signal=32%
CC	GO:0005788	endoplasmic reticulum lumen	164	0.363310932	1.458333657	0.003762825	0.034388946	0.025424	2910	tags=37%, list=23%, signal=29%
CC	GO:0035097	histone methyltransferase complex	52	0.445496746	1.597258066	0.003781071	0.034505692	0.02551	4948	tags=62%, list=39%, signal=38%
BP	GO:1903053	regulation of extracellular matrix organization	25	0.531849111	1.682746825	0.00382628	0.034848309	0.025764	2324	tags=52%, list=18%, signal=42%
BP	GO:0031331	positive regulation of cellular catabolic process	277	0.330427498	1.351140534	0.003829667	0.034848309	0.025764	4538	tags=45%, list=36%, signal=29%
BP	GO:0002687	positive regulation of leukocyte migration	76	0.414800209	1.564536959	0.003843622	0.034910339	0.02581	2829	tags=39%, list=22%, signal=31%
MF	GO:0005178	integrin binding	84	0.407603189	1.545057628	0.003847556	0.034910339	0.02581	3090	tags=40%, list=25%, signal=31%
MF	GO:0140030	modification-dependent protein binding	106	0.391383103	1.523615963	0.003887927	0.035225962	0.026043	5251	tags=60%, list=42%, signal=36%
BP	GO:0010524	positive regulation of calcium ion transport into cytosol	26	0.532177881	1.701784184	0.003895213	0.035241337	0.026054	2775	tags=46%, list=22%, signal=36%
BP	GO:0050731	positive regulation of peptidyl-tyrosine phosphorylation	93	0.402120867	1.542785508	0.003907743	0.035291741	0.026092	3319	tags=40%, list=26%, signal=30%
BP	GO:0034754	cellular hormone metabolic process	57	-0.301663414	-1.671348734	0.003911977	0.035291741	0.026092	2077	tags=42%, list=16%, signal=35%
BP	GO:0030857	negative regulation of epithelial cell differentiation	26	0.530153082	1.695309339	0.003936	0.035434768	0.026197	2714	tags=50%, list=22%, signal=39%
BP	GO:0034341	response to interferon-gamma	123	0.377594935	1.480939353	0.003943422	0.035434768	0.026197	3323	tags=38%, list=26%, signal=28%
BP	GO:0006458	'de novo' protein folding	30	0.526120003	1.718132745	0.003944689	0.035434768	0.026197	3063	tags=47%, list=24%, signal=35%
BP	GO:0032232	negative regulation of actin filament bundle assembly	23	0.548960351	1.707320441	0.003960642	0.035527464	0.026266	3410	tags=65%, list=27%, signal=48%
BP	GO:0030041	actin filament polymerization	112	0.382820788	1.494027084	0.004026659	0.036068337	0.026666	3279	tags=40%, list=26%, signal=30%
MF	GO:0001882	nucleoside binding	221	0.338009479	1.374272248	0.004052747	0.036157999	0.026732	4295	tags=43%, list=34%, signal=29%
CC	GO:0022624	proteasome accessory complex	24	0.546588888	1.712800653	0.004053286	0.036157999	0.026732	3093	tags=50%, list=25%, signal=38%
MF	GO:0031490	chromatin DNA binding	57	0.439800558	1.602402378	0.00405387	0.036157999	0.026732	4420	tags=56%, list=35%, signal=37%
BP	GO:0007183	SMAD protein complex assembly	10	0.681929156	1.73301995	0.004064834	0.03620458	0.026767	2848	tags=70%, list=23%, signal=54%
BP	GO:0060041	retina development in camera-type eye	72	0.416391935	1.559120286	0.004092873	0.036391904	0.026905	2256	tags=36%, list=18%, signal=30%
BP	GO:1903055	positive regulation of extracellular matrix organization	12	0.657319325	1.764064294	0.004097408	0.036391904	0.026905	2207	tags=67%, list=18%, signal=55%
BP	GO:0007093	mitotic cell cycle checkpoint	107	0.387793159	1.510477893	0.004110233	0.036454472	0.026951	4866	tags=53%, list=39%, signal=33%
BP	GO:0007269	neurotransmitter secretion	75	0.417841196	1.574155195	0.004139592	0.036611874	0.027068	3084	tags=36%, list=24%, signal=27%
BP	GO:0099643	signal release from synapse	75	0.417841196	1.574155195	0.004139592	0.036611874	0.027068	3084	tags=36%, list=24%, signal=27%
MF	GO:0003714	transcription corepressor activity	122	0.37689181	1.478953334	0.004168753	0.036752392	0.027172	5140	tags=53%, list=41%, signal=32%
BP	GO:0008064	regulation of actin polymerization or depolymerization	111	0.385577748	1.504579012	0.004172964	0.036752392	0.027172	3279	tags=40%, list=26%, signal=30%
BP	GO:0030832	regulation of actin filament length	111	0.385577748	1.504579012	0.004172964	0.036752392	0.027172	3279	tags=40%, list=26%, signal=30%

CC	GO:0000812	Swr1 complex	11	0.666850813	1.736394646	0.00417988	0.036761954	0.027179	2171	tags=55%, list=17%, signal=45%
MF	GO:0003678	DNA helicase activity	58	0.438129313	1.601732805	0.004208299	0.036907605	0.027286	3856	tags=53%, list=31%, signal=37%
CC	GO:0150034	distal axon	159	0.357237691	1.429829264	0.004209879	0.036907605	0.027286	5249	tags=58%, list=42%, signal=34%
BP	GO:0071772	response to BMP	77	0.408522049	1.541431835	0.004219851	0.036907605	0.027286	2877	tags=42%, list=23%, signal=32%
BP	GO:0071773	cellular response to BMP stimulus	77	0.408522049	1.541431835	0.004219851	0.036907605	0.027286	2877	tags=42%, list=23%, signal=32%
BP	GO:2001212	regulation of vasculogenesis	11	0.66612984	1.734517324	0.004273284	0.037323169	0.027593	1490	tags=55%, list=12%, signal=48%
BP	GO:0010770	positive regulation of cell morphogenesis involved in differentiation	99	0.392824152	1.519446787	0.004287125	0.037392265	0.027645	3124	tags=40%, list=25%, signal=31%
BP	GO:0051783	regulation of nuclear division	101	0.392086556	1.518186836	0.004295883	0.037416902	0.027663	4576	tags=50%, list=36%, signal=32%
BP	GO:0010742	macrophage derived foam cell differentiation	21	0.57542461	1.744750275	0.004310851	0.037443836	0.027683	2295	tags=48%, list=18%, signal=39%
BP	GO:0090077	foam cell differentiation	21	0.57542461	1.744750275	0.004310851	0.037443836	0.027683	2295	tags=48%, list=18%, signal=39%
BP	GO:0010803	regulation of tumor necrosis factor-mediated signaling pathway	39	0.483681329	1.649603262	0.004334188	0.037594759	0.027794	5119	tags=69%, list=41%, signal=41%
BP	GO:0031346	positive regulation of cell projection organization	218	0.345387856	1.404117348	0.004350189	0.037661423	0.027844	4445	tags=45%, list=35%, signal=30%
BP	GO:0032933	SREBP signaling pathway	12	-0.579327841	-2.007872746	0.00435979	0.037661423	0.027844	1734	tags=67%, list=14%, signal=58%
BP	GO:0071501	cellular response to sterol depletion	12	-0.579327841	-2.007872746	0.00435979	0.037661423	0.027844	1734	tags=67%, list=14%, signal=58%
CC	GO:0030119	AP-type membrane coat adaptor complex	26	0.52818811	1.689025803	0.004385865	0.037834835	0.027972	2667	tags=46%, list=21%, signal=36%
BP	GO:0043648	dicarboxylic acid metabolic process	55	-0.296972427	-1.687741687	0.004400687	0.037910839	0.028028	2397	tags=40%, list=19%, signal=33%
BP	GO:0034308	primary alcohol metabolic process	49	-0.312154434	-1.627548116	0.004415723	0.037988472	0.028085	2032	tags=43%, list=16%, signal=36%
BP	GO:0050673	epithelial cell proliferation	224	0.342518131	1.393398075	0.004424549	0.038012541	0.028103	3024	tags=33%, list=24%, signal=26%
MF	GO:0017069	snRNA binding	34	0.49729777	1.654620003	0.004485597	0.038482523	0.028451	4946	tags=71%, list=39%, signal=43%
BP	GO:0040029	regulation of gene expression, epigenetic	104	0.385892655	1.500265986	0.00449642	0.038482523	0.028451	5175	tags=61%, list=41%, signal=36%
MF	GO:0097718	disordered domain specific binding	27	0.523593541	1.681477614	0.004497561	0.038482523	0.028451	4597	tags=63%, list=36%, signal=40%
BP	GO:0061311	cell surface receptor signaling pathway involved in heart development	15	0.621065336	1.742903338	0.004507268	0.038513326	0.028473	1285	tags=40%, list=10%, signal=36%
MF	GO:0008194	UDP-glycosyltransferase activity	63	0.43064512	1.593157407	0.00456125	0.038921841	0.028775	3560	tags=48%, list=28%, signal=34%
BP	GO:0061298	retina vasculature development in camera-type eye	15	0.620398839	1.74103294	0.004596518	0.039117547	0.02892	1403	tags=53%, list=11%, signal=47%
BP	GO:0006081	cellular aldehyde metabolic process	38	-0.345618257	-1.740614097	0.004596591	0.039117547	0.02892	1603	tags=34%, list=13%, signal=30%
BP	GO:1901800	positive regulation of proteasomal protein catabolic process	84	0.406263474	1.539979313	0.004609425	0.039173901	0.028962	3910	tags=48%, list=31%, signal=33%
BP	GO:0031047	gene silencing by RNA	112	0.379983096	1.482952478	0.004628759	0.039285272	0.029044	4052	tags=47%, list=32%, signal=32%
BP	GO:0051031	tRNA transport	29	0.504218618	1.641781895	0.004642019	0.039338268	0.029083	4905	tags=79%, list=39%, signal=49%
CC	GO:0000779	condensed chromosome, centromeric region	64	0.429897067	1.591686992	0.00464748	0.039338268	0.029083	5230	tags=64%, list=41%, signal=38%
BP	GO:0007041	lysosomal transport	78	0.412718768	1.557799241	0.00467604	0.039379301	0.029114	3252	tags=40%, list=26%, signal=30%
BP	GO:0001894	tissue homeostasis	134	0.367521558	1.451473952	0.004685073	0.039379301	0.029114	2626	tags=30%, list=21%, signal=24%
CC	GO:0000123	histone acetyltransferase complex	55	0.437117787	1.582175407	0.004686169	0.039379301	0.029114	4828	tags=60%, list=38%, signal=37%
CC	GO:0031248	protein acetyltransferase complex	55	0.437117787	1.582175407	0.004686169	0.039379301	0.029114	4828	tags=60%, list=38%, signal=37%
CC	GO:1902493	acetyltransferase complex	55	0.437117787	1.582175407	0.004686169	0.039379301	0.029114	4828	tags=60%, list=38%, signal=37%
MF	GO:0008022	protein C-terminus binding	125	0.372886573	1.467029187	0.004689796	0.039379301	0.029114	5225	tags=58%, list=41%, signal=34%
CC	GO:0090734	site of DNA damage	49	0.456462291	1.626387295	0.004710678	0.039475484	0.029185	5453	tags=73%, list=43%, signal=42%
BP	GO:0009124	nucleoside monophosphate biosynthetic process	31	0.50320043	1.646222071	0.004713771	0.039475484	0.029185	2614	tags=52%, list=21%, signal=41%
BP	GO:0006399	tRNA metabolic process	117	0.382722927	1.500263281	0.004760033	0.039767654	0.029401	6115	tags=63%, list=49%, signal=33%
BP	GO:1901550	regulation of endothelial cell development	10	0.678432255	1.724133104	0.004777955	0.039767654	0.029401	2386	tags=60%, list=19%, signal=49%

BP	GO:1903140	regulation of establishment of endothelial barrier	10	0.678432255	1.724133104	0.004777955	0.039767654	0.029401	2386	tags=60%, list=19%, signal=49%
BP	GO:0000380	alternative mRNA splicing, via spliceosome	44	0.461351992	1.60764277	0.004785769	0.039767654	0.029401	4444	tags=61%, list=35%, signal=40%
BP	GO:0070936	protein K48-linked ubiquitination	45	0.463270544	1.619265694	0.004785769	0.039767654	0.029401	4414	tags=60%, list=35%, signal=39%
MF	GO:0043138	3'-5' DNA helicase activity	14	0.60817464	1.68878795	0.004786497	0.039767654	0.029401	3820	tags=79%, list=30%, signal=55%
BP	GO:0050000	chromosome localization	43	0.456656357	1.587299974	0.004806199	0.039826399	0.029444	3869	tags=51%, list=31%, signal=36%
BP	GO:0051303	establishment of chromosome localization	43	0.456656357	1.587299974	0.004806199	0.039826399	0.029444	3869	tags=51%, list=31%, signal=36%
BP	GO:0090100	positive regulation of transmembrane receptor protein serine/threonine kinase signaling pathway	57	0.435012325	1.584956571	0.004826014	0.039938119	0.029527	2910	tags=42%, list=23%, signal=33%
MF	GO:0016627	oxidoreductase activity, acting on the CH-CH group of donors	47	-0.319077863	-1.725000488	0.004851792	0.040055381	0.029613	1140	tags=38%, list=9%, signal=35%
BP	GO:0007044	cell-substrate junction assembly	68	0.420504422	1.568497126	0.004852888	0.040055381	0.029613	2611	tags=38%, list=21%, signal=30%
BP	GO:0002335	mature B cell differentiation	19	0.580148768	1.730028283	0.004867673	0.040072512	0.029626	3288	tags=68%, list=26%, signal=51%
BP	GO:0050849	negative regulation of calcium-mediated signaling	19	0.582396415	1.736730863	0.004867673	0.040072512	0.029626	3608	tags=63%, list=29%, signal=45%
MF	GO:0004518	nuclease activity	127	0.368348709	1.451420764	0.004905013	0.040302369	0.029796	5229	tags=52%, list=41%, signal=31%
BP	GO:0009112	nucleobase metabolic process	20	0.568965404	1.709674062	0.004908376	0.040302369	0.029796	1297	tags=45%, list=10%, signal=40%
BP	GO:0031532	actin cytoskeleton reorganization	69	0.421607903	1.573959885	0.004944539	0.040498217	0.029941	3650	tags=49%, list=29%, signal=35%
BP	GO:0046649	lymphocyte activation	378	0.311626356	1.292227892	0.004945072	0.040498217	0.029941	5019	tags=46%, list=40%, signal=28%
BP	GO:0032231	regulation of actin filament bundle assembly	73	0.417489556	1.566387151	0.005016031	0.041026063	0.030331	3561	tags=48%, list=28%, signal=35%
CC	GO:0036477	somatodendritic compartment	410	0.30628409	1.27087515	0.005093794	0.041568352	0.030732	4445	tags=43%, list=35%, signal=29%
CC	GO:0070603	SWI/SNF superfamily-type complex	63	0.427095957	1.580027396	0.005097855	0.041568352	0.030732	5977	tags=75%, list=47%, signal=39%
BP	GO:0016458	gene silencing	149	0.367365543	1.465396317	0.005109106	0.041568352	0.030732	4052	tags=46%, list=32%, signal=31%
BP	GO:0032273	positive regulation of protein polymerization	82	0.405809925	1.535101124	0.005126992	0.041568352	0.030732	4221	tags=46%, list=33%, signal=31%
MF	GO:0000175	3'-5'-exoribonuclease activity	29	0.502330248	1.635633192	0.005134965	0.041568352	0.030732	5081	tags=66%, list=40%, signal=39%
BP	GO:0048332	mesoderm morphogenesis	30	0.521489068	1.703009653	0.005134965	0.041568352	0.030732	3201	tags=50%, list=25%, signal=37%
BP	GO:0010971	positive regulation of G2/M transition of mitotic cell cycle	16	0.601653706	1.717829622	0.005135069	0.041568352	0.030732	2312	tags=62%, list=18%, signal=51%
BP	GO:1902751	positive regulation of cell cycle G2/M phase transition	16	0.601653706	1.717829622	0.005135069	0.041568352	0.030732	2312	tags=62%, list=18%, signal=51%
BP	GO:0050920	regulation of chemotaxis	121	0.371949675	1.456688966	0.005155599	0.041681036	0.030815	2829	tags=33%, list=22%, signal=26%
BP	GO:0017144	drug metabolic process	13	-0.590286466	-2.087785883	0.005196981	0.041961792	0.031023	1616	tags=62%, list=13%, signal=54%
CC	GO:1904949	ATPase complex	66	0.422924639	1.574749697	0.005229628	0.042168297	0.031176	4386	tags=55%, list=35%, signal=36%
BP	GO:0007051	spindle organization	107	0.384565797	1.497907122	0.005241461	0.042168297	0.031176	4864	tags=55%, list=39%, signal=34%
BP	GO:0003181	atrioventricular valve morphogenesis	18	0.582875764	1.7130159	0.005242617	0.042168297	0.031176	2877	tags=61%, list=23%, signal=47%
BP	GO:0009083	branched-chain amino acid catabolic process	20	-0.450980021	-1.92017869	0.00525399	0.042185716	0.031188	1651	tags=35%, list=13%, signal=30%
BP	GO:0006862	nucleotide transport	24	-0.397697065	-1.716518084	0.005258163	0.042185716	0.031188	1209	tags=33%, list=10%, signal=30%
BP	GO:0099504	synaptic vesicle cycle	92	0.403287353	1.544834574	0.005284293	0.042341486	0.031304	3595	tags=41%, list=29%, signal=30%
MF	GO:0005172	vascular endothelial growth factor receptor binding	10	0.675298018	1.716167916	0.005303254	0.042396391	0.031344	1286	tags=50%, list=10%, signal=45%
MF	GO:0016741	transferase activity, transferring one-carbon groups	110	0.381915249	1.489386193	0.005311495	0.042396391	0.031344	4654	tags=52%, list=37%, signal=33%
BP	GO:0090503	RNA phosphodiester bond hydrolysis, exonucleolytic	35	0.48488543	1.623288756	0.0053226	0.042396391	0.031344	5081	tags=63%, list=40%, signal=38%
BP	GO:0060547	negative regulation of necrotic cell death	12	0.649662751	1.743516155	0.005323397	0.042396391	0.031344	1964	tags=58%, list=16%, signal=49%
BP	GO:0051895	negative regulation of focal adhesion assembly	13	0.629011404	1.721701082	0.005336113	0.042396391	0.031344	2492	tags=54%, list=20%, signal=43%
BP	GO:0150118	negative regulation of cell-substrate junction organization	13	0.629011404	1.721701082	0.005336113	0.042396391	0.031344	2492	tags=54%, list=20%, signal=43%
BP	GO:0016358	dendrite development	144	0.367900344	1.463487733	0.005338207	0.042396391	0.031344	4545	tags=49%, list=36%, signal=32%

MF	GO:0005088	Ras guanyl-nucleotide exchange factor activity	68	0.418421818	1.560728935	0.005387841	0.042736764	0.031596	3502	tags=47%, list=28%, signal=34%
BP	GO:0008299	isoprenoid biosynthetic process	20	-0.448418832	-1.909273685	0.005395034	0.042740057	0.031598	1407	tags=60%, list=11%, signal=53%
BP	GO:0050654	chondroitin sulfate proteoglycan metabolic process	28	0.516069667	1.669522592	0.005405302	0.042767673	0.031619	3518	tags=61%, list=28%, signal=44%
BP	GO:0035455	response to interferon-alpha	18	0.582420277	1.711677268	0.005415923	0.042774632	0.031624	3129	tags=67%, list=25%, signal=50%
CC	GO:0031256	leading edge membrane	98	0.383705164	1.481891075	0.005419748	0.042774632	0.031624	3081	tags=36%, list=24%, signal=27%
BP	GO:0033045	regulation of sister chromatid segregation	51	0.444667792	1.59054583	0.005434162	0.042798578	0.031642	4381	tags=51%, list=35%, signal=33%
BP	GO:0046718	viral entry into host cell	79	0.400923451	1.515294165	0.005436356	0.042798578	0.031642	3117	tags=42%, list=25%, signal=32%
MF	GO:0001883	purine nucleoside binding	217	0.340334095	1.383763303	0.005465597	0.042975131	0.031772	4295	tags=44%, list=34%, signal=29%
BP	GO:0007517	muscle organ development	199	0.336039779	1.359178812	0.005476539	0.043007542	0.031796	1857	tags=27%, list=15%, signal=23%
BP	GO:0001510	RNA methylation	40	0.466404092	1.595585295	0.005515269	0.04325782	0.031981	5793	tags=78%, list=46%, signal=42%
BP	GO:0006826	iron ion transport	42	-0.323932677	-1.709156588	0.005527914	0.043303137	0.032015	1078	tags=24%, list=9%, signal=22%
BP	GO:1902532	negative regulation of intracellular signal transduction	314	0.320324357	1.317453627	0.005539958	0.04334364	0.032044	4072	tags=40%, list=32%, signal=28%
BP	GO:2000249	regulation of actin cytoskeleton reorganization	27	0.517755752	1.662730036	0.005570346	0.043527391	0.03218	1242	tags=33%, list=10%, signal=30%
BP	GO:1903902	positive regulation of viral life cycle	43	0.45355449	1.576518142	0.005600595	0.043709594	0.032315	5179	tags=67%, list=41%, signal=40%
BP	GO:0000291	nuclear-transcribed mRNA catabolic process, exonucleolytic	30	0.519244187	1.69567862	0.005627911	0.043868489	0.032433	4811	tags=70%, list=38%, signal=43%
BP	GO:0003281	ventricular septum development	42	0.468713988	1.627931366	0.005643895	0.043938764	0.032484	4476	tags=60%, list=36%, signal=39%
BP	GO:0009084	glutamine family amino acid biosynthetic process	10	-0.627408597	-2.040078145	0.005660202	0.044011384	0.032538	2231	tags=70%, list=18%, signal=58%
BP	GO:0015701	bicarbonate transport	19	-0.467919412	-1.955238149	0.005680186	0.044112377	0.032613	871	tags=42%, list=7%, signal=39%
BP	GO:0071407	cellular response to organic cyclic compound	321	0.321107309	1.322963236	0.005688679	0.044124	0.032621	4504	tags=44%, list=36%, signal=29%
MF	GO:0016896	exoribonuclease activity, producing 5'-phosphomonoesters	31	0.497670264	1.628130111	0.005697492	0.044138062	0.032632	5081	tags=65%, list=40%, signal=39%
BP	GO:0097711	ciliary basal body-plasma membrane docking	53	0.441291673	1.586340116	0.005710921	0.044187816	0.032669	4841	tags=60%, list=38%, signal=37%
MF	GO:0032550	purine ribonucleoside binding	215	0.340466312	1.38356379	0.00576304	0.044536435	0.032926	4295	tags=44%, list=34%, signal=29%
CC	GO:0099522	region of cytosol	14	0.600417325	1.667247329	0.00577061	0.044540352	0.032929	2750	tags=57%, list=22%, signal=45%
BP	GO:0008284	positive regulation of cell population proliferation	473	0.303621125	1.264810432	0.005837401	0.045000794	0.03327	4561	tags=42%, list=36%, signal=28%
BP	GO:0035567	non-canonical Wnt signaling pathway	106	0.383964382	1.49473561	0.005850713	0.045048348	0.033305	3773	tags=43%, list=30%, signal=31%
BP	GO:0051983	regulation of chromosome segregation	61	0.429791651	1.583181681	0.005858403	0.04505255	0.033308	4381	tags=49%, list=35%, signal=32%
MF	GO:0030551	cyclic nucleotide binding	14	0.598508314	1.66194636	0.00594954	0.045697687	0.033785	2275	tags=50%, list=18%, signal=41%
BP	GO:0045687	positive regulation of glial cell differentiation	23	0.539470264	1.677805341	0.005959902	0.045721584	0.033803	2203	tags=52%, list=17%, signal=43%
CC	GO:0072686	mitotic spindle	73	0.415386921	1.558498233	0.006011562	0.046061859	0.034054	4839	tags=58%, list=38%, signal=36%
CC	GO:0048471	perinuclear region of cytoplasm	441	0.304252831	1.264992943	0.006060483	0.046380349	0.03429	3271	tags=33%, list=26%, signal=25%
CC	GO:0000922	spindle pole	89	0.396862129	1.518346453	0.006101944	0.046641038	0.034482	4839	tags=55%, list=38%, signal=34%
BP	GO:0022613	ribonucleoprotein complex biogenesis	323	0.321280519	1.324693662	0.006134844	0.046825893	0.034619	5739	tags=57%, list=46%, signal=32%
BP	GO:0051961	negative regulation of nervous system development	178	0.343962268	1.384424742	0.006140979	0.046825893	0.034619	3791	tags=42%, list=30%, signal=30%
BP	GO:0051057	positive regulation of small GTPase mediated signal transduction	39	0.475817425	1.622783285	0.006163251	0.046938964	0.034703	2823	tags=51%, list=22%, signal=40%
BP	GO:0045665	negative regulation of neuron differentiation	131	0.365799075	1.442383689	0.006190503	0.047001977	0.034749	4508	tags=50%, list=36%, signal=33%
CC	GO:0098794	postsynapse	311	0.319780197	1.314547265	0.006209205	0.047001977	0.034749	4575	tags=43%, list=36%, signal=28%
BP	GO:1903362	regulation of cellular protein catabolic process	186	0.34879168	1.408197223	0.006215414	0.047001977	0.034749	5213	tags=54%, list=41%, signal=32%
BP	GO:0006400	tRNA modification	58	0.429552791	1.570378368	0.00621588	0.047001977	0.034749	6115	tags=74%, list=49%, signal=38%
MF	GO:1990841	promoter-specific chromatin binding	41	0.466340141	1.608863791	0.006222831	0.047001977	0.034749	4113	tags=59%, list=33%, signal=40%

BP	GO:0003159	morphogenesis of an endothelium	13	0.623696087	1.707152241	0.006232919	0.047001977	0.034749	3717	tags=62%, list=29%, signal=43%
BP	GO:0061154	endothelial tube morphogenesis	13	0.623696087	1.707152241	0.006232919	0.047001977	0.034749	3717	tags=62%, list=29%, signal=43%
BP	GO:0016339	calcium-dependent cell-cell adhesion via plasma membrane cell adhesion molecules	12	0.644163971	1.728758943	0.006233359	0.047001977	0.034749	1584	tags=50%, list=13%, signal=44%
BP	GO:0006577	amino-acid betaine metabolic process	10	-0.620654999	-2.018118185	0.006238607	0.047001977	0.034749	2629	tags=60%, list=21%, signal=48%
MF	GO:0015631	tubulin binding	196	0.342072234	1.383921746	0.006296145	0.047345757	0.035003	4898	tags=52%, list=39%, signal=32%
BP	GO:0006123	mitochondrial electron transport, cytochrome c to oxygen	15	-0.503282265	-1.90987212	0.006306761	0.047345757	0.035003	768	tags=33%, list=6%, signal=31%
BP	GO:0019646	aerobic electron transport chain	15	-0.503282265	-1.90987212	0.006306761	0.047345757	0.035003	768	tags=33%, list=6%, signal=31%
BP	GO:0007186	G protein-coupled receptor signaling pathway	337	0.315627243	1.30350242	0.006357927	0.047598758	0.03519	3639	tags=36%, list=29%, signal=26%
BP	GO:0097006	regulation of plasma lipoprotein particle levels	45	0.457831632	1.600255109	0.006367809	0.047598758	0.03519	2667	tags=38%, list=21%, signal=30%
BP	GO:0051310	metaphase plate congression	36	0.477146604	1.60366784	0.006369939	0.047598758	0.03519	3869	tags=56%, list=31%, signal=39%
BP	GO:0010976	positive regulation of neuron projection development	164	0.354189985	1.421722084	0.006370655	0.047598758	0.03519	4221	tags=44%, list=33%, signal=30%
BP	GO:0032412	regulation of ion transmembrane transporter activity	112	0.375540484	1.465614383	0.00643507	0.04802314	0.035504	3851	tags=42%, list=31%, signal=29%
BP	GO:0032205	negative regulation of telomere maintenance	23	0.536677354	1.669119116	0.006460026	0.048088585	0.035552	3127	tags=52%, list=25%, signal=39%
BP	GO:0003206	cardiac chamber morphogenesis	67	0.420623781	1.568445885	0.006464388	0.048088585	0.035552	2877	tags=40%, list=23%, signal=31%
MF	GO:0030170	pyridoxal phosphate binding	25	-0.405340697	-1.798272224	0.006474343	0.048088585	0.035552	1408	tags=40%, list=11%, signal=36%
MF	GO:0070279	vitamin B6 binding	25	-0.405340697	-1.798272224	0.006474343	0.048088585	0.035552	1408	tags=40%, list=11%, signal=36%
BP	GO:0061844	antimicrobial humoral immune response mediated by antimicrobial peptide	14	-0.49575968	-1.793502557	0.006557733	0.048650666	0.035968	1573	tags=43%, list=12%, signal=38%
BP	GO:2001251	negative regulation of chromosome organization	84	0.400108103	1.51664681	0.006590297	0.048783477	0.036066	3347	tags=43%, list=27%, signal=32%
BP	GO:0003151	outflow tract morphogenesis	46	0.450370155	1.580545495	0.006591107	0.048783477	0.036066	2386	tags=41%, list=19%, signal=34%
BP	GO:0051492	regulation of stress fiber assembly	66	0.416125161	1.549432006	0.006680159	0.049384622	0.036511	3561	tags=47%, list=28%, signal=34%
BP	GO:0071827	plasma lipoprotein particle organization	23	0.53596075	1.666890409	0.006710088	0.049547793	0.036631	2311	tags=39%, list=18%, signal=32%
BP	GO:0038094	Fc-gamma receptor signaling pathway	64	0.422217402	1.563253155	0.006721522	0.049574175	0.036651	4746	tags=55%, list=38%, signal=34%
MF	GO:0017081	chloride channel regulator activity	10	-0.612835816	-1.992693376	0.006734383	0.049611005	0.036678	2206	tags=60%, list=17%, signal=50%
BP	GO:0010324	membrane invagination	43	0.448931087	1.56044757	0.006792193	0.049978492	0.03695	1805	tags=35%, list=14%, signal=30%

KEGG

ID	Description	setSize	enrichmentScore	NES	pvalue	p.adjust	qvalues	rank	leading_edge
hsa00280	Valine, leucine and isoleucine degradation	38	-0.559924171	-2.742646027	7.07602E-08	2.20772E-05	1.51E-05	2350	tags=47%, list=19%, signal=39%
hsa05200	Pathways in cancer	316	0.388390652	1.596891488	4.98089E-07	7.77019E-05	5.32E-05	4605	tags=51%, list=37%, signal=33%
hsa04151	PI3K-Akt signaling pathway	200	0.408049996	1.64494436	2.94366E-06	0.000242789	0.000166	2847	tags=39%, list=23%, signal=31%
hsa05132	Salmonella infection	187	0.415378935	1.671804524	3.11268E-06	0.000242789	0.000166	3447	tags=45%, list=27%, signal=34%
hsa04144	Endocytosis	175	0.411030251	1.645475361	6.18698E-06	0.000335276	0.00023	3251	tags=46%, list=26%, signal=35%
hsa03040	Spliceosome	102	0.470118652	1.808835328	6.44762E-06	0.000335276	0.00023	5439	tags=72%, list=43%, signal=41%
hsa05169	Epstein-Barr virus infection	144	0.43433708	1.714415407	7.89747E-06	0.000352002	0.000241	5015	tags=58%, list=40%, signal=36%
hsa05222	Small cell lung cancer	70	0.506255533	1.885655423	1.66216E-05	0.000648243	0.000444	2902	tags=49%, list=23%, signal=38%
hsa05100	Bacterial invasion of epithelial cells	53	0.525274389	1.898713179	2.72251E-05	0.0009378	0.000642	3605	tags=62%, list=29%, signal=45%
hsa05165	Human papillomavirus infection	216	0.3870021	1.562424068	3.00577E-05	0.0009378	0.000642	4256	tags=50%, list=34%, signal=33%
hsa04510	Focal adhesion	136	0.416770871	1.642129658	5.21248E-05	0.001397796	0.000957	4412	tags=51%, list=35%, signal=34%
hsa05166	Human T-cell leukemia virus 1 infection	153	0.406584871	1.608633883	5.37614E-05	0.001397796	0.000957	4255	tags=46%, list=34%, signal=31%
hsa00640	Propanoate metabolism	28	-0.516651194	-2.356002021	6.77717E-05	0.001626521	0.001114	2900	tags=61%, list=23%, signal=47%

hsa05203	Viral carcinogenesis	127	0.419674965	1.645541658	9.9201E-05	0.002196726	0.001505	5249	tags=61%, list=42%, signal=36%
hsa05135	Yersinia infection	99	0.443430879	1.705228339	0.000105612	0.002196726	0.001505	3650	tags=48%, list=29%, signal=35%
hsa00330	Arginine and proline metabolism	30	-0.47601508	-2.163545275	0.00011613	0.002264544	0.001551	1878	tags=50%, list=15%, signal=43%
hsa04810	Regulation of actin cytoskeleton	142	0.394150159	1.55614409	0.000217393	0.003776999	0.002587	4433	tags=51%, list=35%, signal=33%
hsa04512	ECM-receptor interaction	51	0.499942667	1.803243879	0.000217904	0.003776999	0.002587	3090	tags=49%, list=25%, signal=37%
hsa05164	Influenza A	107	0.424620484	1.641775917	0.000303021	0.004912872	0.003365	3805	tags=44%, list=30%, signal=31%
hsa00650	Butanoate metabolism	12	-0.706163782	-2.464959662	0.000314928	0.004912872	0.003365	2965	tags=83%, list=24%, signal=64%
hsa05130	Pathogenic Escherichia coli infection	133	0.401063021	1.576410165	0.000345429	0.005132086	0.003515	4886	tags=58%, list=39%, signal=36%
hsa05168	Herpes simplex virus 1 infection	265	0.348403074	1.423643226	0.00037139	0.005266982	0.003607	5142	tags=49%, list=41%, signal=30%
hsa05160	Hepatitis C	106	0.418938652	1.618936814	0.000446886	0.006062105	0.004152	5225	tags=64%, list=41%, signal=38%
hsa03013	Nucleocytoplasmic transport	83	0.428723664	1.618297737	0.000662241	0.008609131	0.005896	5239	tags=66%, list=42%, signal=39%
hsa04110	Cell cycle	83	0.425393678	1.605728083	0.000840714	0.01023975	0.007013	4947	tags=61%, list=39%, signal=38%
hsa00520	Amino sugar and nucleotide sugar metabolism	36	-0.38525762	-1.886378288	0.000853313	0.01023975	0.007013	2509	tags=44%, list=20%, signal=36%
hsa04371	Apelin signaling pathway	79	0.436833419	1.64656885	0.000949543	0.010496758	0.007189	4657	tags=62%, list=37%, signal=39%
hsa05220	Chronic myeloid leukemia	56	0.461646186	1.67703145	0.000956786	0.010496758	0.007189	5557	tags=68%, list=44%, signal=38%
hsa01200	Carbon metabolism	84	-0.264930487	-1.546782555	0.000997416	0.010496758	0.007189	2137	tags=37%, list=17%, signal=31%
hsa04014	Ras signaling pathway	123	0.391693439	1.531048023	0.001009304	0.010496758	0.007189	3488	tags=41%, list=28%, signal=30%
hsa05414	Dilated cardiomyopathy	46	0.488999253	1.726449701	0.001072924	0.010798464	0.007396	2393	tags=41%, list=19%, signal=34%
hsa04666	Fc gamma R-mediated phagocytosis	73	0.434122845	1.621698199	0.001434236	0.013983805	0.009577	3978	tags=53%, list=32%, signal=37%
hsa05146	Amoebiasis	53	0.461850306	1.66945368	0.001852253	0.017512213	0.011994	2847	tags=38%, list=23%, signal=29%
hsa05206	MicroRNAs in cancer	116	0.386710629	1.504873947	0.002113638	0.018871301	0.012925	4433	tags=53%, list=35%, signal=34%
hsa05145	Toxoplasmosis	72	0.433511114	1.619030059	0.002154996	0.018871301	0.012925	2847	tags=38%, list=23%, signal=29%
hsa04015	Rap1 signaling pathway	126	0.383232415	1.501664816	0.002177458	0.018871301	0.012925	2829	tags=36%, list=22%, signal=28%
hsa04330	Notch signaling pathway	44	0.480335544	1.676449864	0.002419697	0.019925877	0.013647	3822	tags=57%, list=30%, signal=40%
hsa00071	Fatty acid degradation	30	-0.402497283	-1.829398125	0.002531644	0.019925877	0.013647	2900	tags=53%, list=23%, signal=41%
hsa00220	Arginine biosynthesis	10	-0.66998585	-2.14328923	0.002534446	0.019925877	0.013647	1280	tags=40%, list=10%, signal=36%
hsa05167	Kaposi sarcoma-associated herpesvirus infection	128	0.375591266	1.472888157	0.002584288	0.019925877	0.013647	5015	tags=55%, list=40%, signal=33%
hsa04072	Phospholipase D signaling pathway	89	0.410900573	1.564219662	0.002618465	0.019925877	0.013647	4258	tags=52%, list=34%, signal=34%
hsa00630	Glyoxylate and dicarboxylate metabolism	23	-0.429668813	-1.85392807	0.002950486	0.021917892	0.015011	2350	tags=43%, list=19%, signal=35%
hsa03050	Proteasome	41	0.484155031	1.675967985	0.003089589	0.022358694	0.015313	3093	tags=46%, list=25%, signal=35%
hsa00230	Purine metabolism	71	0.426781435	1.592440544	0.003153149	0.022358694	0.015313	2649	tags=41%, list=21%, signal=32%
hsa00830	Retinol metabolism	19	-0.467372306	-1.891227589	0.003815444	0.026453746	0.018118	1407	tags=42%, list=11%, signal=37%
hsa04670	Leukocyte transendothelial migration	70	0.421481809	1.569897822	0.004195243	0.02823509	0.019338	3288	tags=44%, list=26%, signal=33%
hsa04115	p53 signaling pathway	47	0.460043908	1.633039658	0.004297458	0.02823509	0.019338	4148	tags=57%, list=33%, signal=39%
hsa05163	Human cytomegalovirus infection	146	0.365514972	1.44253846	0.004372027	0.02823509	0.019338	4507	tags=49%, list=36%, signal=32%
hsa05162	Measles	87	0.399802599	1.518803698	0.004434357	0.02823509	0.019338	4148	tags=46%, list=33%, signal=31%
hsa00534	Glycosaminoglycan biosynthesis - heparan sulfate / heparin	14	0.636381599	1.773359886	0.005206939	0.0324913	0.022253	3272	tags=64%, list=26%, signal=48%
hsa05161	Hepatitis B	101	0.381570128	1.468649525	0.005629947	0.0331895	0.022731	5237	tags=55%, list=42%, signal=33%
hsa00380	Tryptophan metabolism	24	-0.40813938	-1.809178036	0.005633787	0.0331895	0.022731	2350	tags=54%, list=19%, signal=44%

	hsa05170	Human immunodeficiency virus 1 infection	128	0.364140341	1.427983142	0.005791971	0.0331895	0.022731	5706	tags=63%, list=45%, signal=35%
	hsa04210	Apoptosis	96	0.387245604	1.4855895	0.005832966	0.0331895	0.022731	5015	tags=53%, list=40%, signal=32%
	hsa04530	Tight junction	108	0.388781217	1.5041966	0.005850713	0.0331895	0.022731	3274	tags=42%, list=26%, signal=31%
	hsa05131	Shigellosis	167	0.358819562	1.428912656	0.006140979	0.034214026	0.023433	3618	tags=41%, list=29%, signal=30%
	hsa01230	Biosynthesis of amino acids	48	-0.298286205	-1.561921544	0.006468333	0.035405614	0.024249	1801	tags=38%, list=14%, signal=32%
	hsa04071	Sphingolipid signaling pathway	78	0.400812556	1.506665353	0.006673315	0.03589783	0.024586	5482	tags=64%, list=43%, signal=36%
	hsa04146	Peroxisome	55	-0.27868739	-1.472762084	0.007176024	0.037477306	0.025668	2137	tags=33%, list=17%, signal=27%
	hsa04270	Vascular smooth muscle contraction	78	0.399376845	1.501268477	0.007207174	0.037477306	0.025668	2393	tags=36%, list=19%, signal=29%
	hsa05211	Renal cell carcinoma	49	0.438476753	1.562817911	0.007554344	0.03863861	0.026463	3887	tags=51%, list=31%, signal=35%
	hsa05215	Prostate cancer	67	0.413701358	1.53129125	0.00845816	0.042563643	0.029151	3887	tags=49%, list=31%, signal=34%
	hsa04540	Gap junction	53	0.433804489	1.56807626	0.00864924	0.042834332	0.029337	4605	tags=62%, list=37%, signal=40%
	hsa01522	Endocrine resistance	61	0.417263137	1.528459121	0.00881426	0.042969515	0.029429	2829	tags=39%, list=22%, signal=31%
	hsa05416	Viral myocarditis	39	0.461265522	1.582579652	0.009217966	0.044246237	0.030304	5443	tags=59%, list=43%, signal=34%
	hsa03018	RNA degradation	63	0.414786425	1.525425154	0.009719858	0.045948418	0.031469	4811	tags=56%, list=38%, signal=35%
	hsa05212	Pancreatic cancer	56	0.418690343	1.520984889	0.01048653	0.048832794	0.033445	3005	tags=41%, list=24%, signal=31%

WARS

GO

**ONTOLOGY**	**ID**	**Description**	**setSize**	**enrichmentScore**	**NES**	**pvalue**	**p.adjust**	**qvalues**	**rank**	**leading_edge**
BP	GO:0001525	angiogenesis	309	0.428238438	1.824881262	1.00E-10	1.58E-07	1.21E-07	3378	tags=44%, list=27%, signal=33%
BP	GO:0001568	blood vessel development	412	0.413140316	1.778308317	1.00E-10	1.58E-07	1.21E-07	3457	tags=43%, list=27%, signal=32%
BP	GO:0001944	vasculature development	436	0.402161333	1.732754446	1.00E-10	1.58E-07	1.21E-07	3457	tags=42%, list=27%, signal=32%
BP	GO:0048514	blood vessel morphogenesis	358	0.414777775	1.776648922	1.00E-10	1.58E-07	1.21E-07	3398	tags=43%, list=27%, signal=32%
BP	GO:0003158	endothelium development	80	0.570603097	2.195486796	9.81E-10	1.24E-06	9.49E-07	3060	tags=61%, list=24%, signal=47%
BP	GO:0030036	actin cytoskeleton organization	422	0.385937977	1.66142915	1.54E-09	1.62E-06	1.24E-06	2790	tags=37%, list=22%, signal=30%
BP	GO:0000904	cell morphogenesis involved in differentiation	425	0.385159661	1.658301242	2.99E-09	2.61E-06	2.00E-06	3422	tags=43%, list=27%, signal=33%
BP	GO:0030029	actin filament-based process	462	0.374966511	1.618809989	3.31E-09	2.61E-06	2.00E-06	2790	tags=36%, list=22%, signal=29%
BP	GO:0000377	RNA splicing, via transesterification reactions with bulged adenosine as nucleophile	247	0.421566118	1.77823183	5.64E-09	3.56E-06	2.73E-06	5336	tags=62%, list=42%, signal=36%
BP	GO:0000398	mRNA splicing, via spliceosome	247	0.421566118	1.77823183	5.64E-09	3.56E-06	2.73E-06	5336	tags=62%, list=42%, signal=36%
BP	GO:0001667	ameboidal-type cell migration	267	0.41518785	1.757516459	6.79E-09	3.90E-06	2.99E-06	3371	tags=45%, list=27%, signal=33%
BP	GO:0000375	RNA splicing, via transesterification reactions	250	0.421109464	1.777189455	7.84E-09	4.12E-06	3.16E-06	5336	tags=62%, list=42%, signal=36%
BP	GO:0051301	cell division	330	0.397560472	1.697613406	8.85E-09	4.23E-06	3.24E-06	4281	tags=50%, list=34%, signal=34%
BP	GO:0006897	endocytosis	318	0.401926584	1.713844395	9.37E-09	4.23E-06	3.24E-06	4096	tags=49%, list=32%, signal=34%
BP	GO:0007264	small GTPase mediated signal transduction	327	0.3982013	1.699471595	1.14E-08	4.81716E-06	3.69E-06	3335	tags=44%, list=26%, signal=33%
BP	GO:0031589	cell-substrate adhesion	218	0.427718401	1.797172392	2.86E-08	1.12684E-05	8.64E-06	3467	tags=47%, list=28%, signal=35%
BP	GO:0009062	fatty acid catabolic process	70	-0.443855162	-2.436809384	5.75E-08	2.04191E-05	1.56E-05	2248	tags=53%, list=18%, signal=44%
BP	GO:0035239	tube morphogenesis	491	0.363347218	1.572235765	5.82E-08	2.04191E-05	1.56E-05	4458	tags=49%, list=35%, signal=33%
BP	GO:0007265	Ras protein signal transduction	223	0.422328536	1.777392674	6.48E-08	2.15217E-05	1.65E-05	3861	tags=52%, list=31%, signal=37%
BP	GO:0072329	monocarboxylic acid catabolic process	80	-0.408566711	-2.374651779	6.99E-08	2.20693E-05	1.69E-05	1971	tags=38%, list=16%, signal=32%
CC	GO:0030055	cell-substrate junction	337	0.387078948	1.655135064	8.24E-08	2.47798E-05	1.9E-05	2792	tags=39%, list=22%, signal=31%

BP	GO:0090130	tissue migration	198	0.428239657	1.792549174	1.07E-07	3.06757E-05	2.35E-05	3371	tags=45%, list=27%, signal=34%
BP	GO:0010631	epithelial cell migration	196	0.428548667	1.79288716	1.30E-07	3.36624E-05	2.58E-05	3371	tags=45%, list=27%, signal=34%
BP	GO:0090132	epithelium migration	196	0.428548667	1.79288716	1.30E-07	3.36624E-05	2.58E-05	3371	tags=45%, list=27%, signal=34%
BP	GO:0045446	endothelial cell differentiation	67	0.558702564	2.112710354	1.33E-07	3.36624E-05	2.58E-05	3009	tags=61%, list=24%, signal=47%
CC	GO:0005925	focal adhesion	335	0.38873355	1.661508924	1.68E-07	4.07391E-05	3.12E-05	2792	tags=39%, list=22%, signal=31%
BP	GO:0032990	cell part morphogenesis	392	0.371707859	1.59798595	1.87E-07	4.36277E-05	3.34E-05	2800	tags=37%, list=22%, signal=30%
BP	GO:0042330	taxis	324	0.381440908	1.626848927	2.06E-07	4.63941E-05	3.56E-05	2594	tags=34%, list=21%, signal=28%
BP	GO:0007409	axonogenesis	262	0.399740823	1.690671893	2.40E-07	5.21723E-05	4E-05	2800	tags=40%, list=22%, signal=32%
MF	GO:0008134	transcription factor binding	425	0.362213701	1.559507632	3.11E-07	6.55419E-05	5.02E-05	4695	tags=51%, list=37%, signal=33%
BP	GO:0048858	cell projection morphogenesis	377	0.369343835	1.585005248	3.33E-07	6.77424E-05	5.19E-05	2800	tags=37%, list=22%, signal=30%
BP	GO:0043542	endothelial cell migration	145	0.450736824	1.846651291	3.49E-07	6.88904E-05	5.28E-05	3371	tags=48%, list=27%, signal=35%
BP	GO:0061564	axon development	287	0.38775001	1.645701463	4.31E-07	8.24465E-05	6.32E-05	2800	tags=39%, list=22%, signal=31%
BP	GO:0048667	cell morphogenesis involved in neuron differentiation	331	0.375842279	1.605510728	4.64E-07	8.61872E-05	6.61E-05	2800	tags=38%, list=22%, signal=30%
BP	GO:0006397	mRNA processing	341	0.372796391	1.594433348	5.41E-07	9.7648E-05	7.48E-05	5275	tags=57%, list=42%, signal=34%
BP	GO:0008380	RNA splicing	311	0.384448868	1.637678346	5.67E-07	9.94206E-05	7.62E-05	5304	tags=59%, list=42%, signal=35%
BP	GO:0044772	mitotic cell cycle phase transition	379	0.363832688	1.561869737	6.00E-07	0.00010245	7.85E-05	4447	tags=48%, list=35%, signal=32%
BP	GO:0007411	axon guidance	149	0.443164201	1.819154432	6.58E-07	0.000106532	8.16E-05	2594	tags=42%, list=21%, signal=33%
BP	GO:0097485	neuron projection guidance	149	0.443164201	1.819154432	6.58E-07	0.000106532	8.16E-05	2594	tags=42%, list=21%, signal=33%
BP	GO:0097435	supramolecular fiber organization	417	0.358009916	1.541654503	7.11E-07	0.00011224	8.6E-05	3143	tags=37%, list=25%, signal=29%
BP	GO:0001935	endothelial cell proliferation	85	0.504729785	1.960680522	7.36E-07	0.000113402	8.69E-05	3216	tags=49%, list=26%, signal=37%
BP	GO:0007015	actin filament organization	259	0.393002662	1.66130137	8.14E-07	0.00012238	9.38E-05	2777	tags=38%, list=22%, signal=30%
BP	GO:0006935	chemotaxis	322	0.380245348	1.621339675	8.57E-07	0.000125792	9.64E-05	2594	tags=34%, list=21%, signal=28%
MF	GO:0019900	kinase binding	469	0.35055013	1.514292471	8.98E-07	0.000128944	9.88E-05	4439	tags=49%, list=35%, signal=33%
BP	GO:0001570	vasculogenesis	53	0.578518395	2.132272759	9.53E-07	0.000133694	0.000102	2760	tags=55%, list=22%, signal=43%
BP	GO:0048812	neuron projection morphogenesis	362	0.366528642	1.570287658	1.09E-06	0.000149329	0.000114	2800	tags=37%, list=22%, signal=30%
MF	GO:0003779	actin binding	242	0.397765691	1.677445172	1.13E-06	0.000151208	0.000116	3848	tags=48%, list=31%, signal=34%
BP	GO:0032989	cellular component morphogenesis	435	0.356161686	1.534542033	1.24E-06	0.000163226	0.000125	2800	tags=36%, list=22%, signal=29%
BP	GO:0120039	plasma membrane bounded cell projection morphogenesis	374	0.363077976	1.557265156	1.46E-06	0.000188753	0.000145	2800	tags=37%, list=22%, signal=29%
CC	GO:0005681	spliceosomal complex	140	0.441911412	1.806583417	1.69E-06	0.000213071	0.000163	5564	tags=68%, list=44%, signal=38%
BP	GO:0032956	regulation of actin cytoskeleton organization	222	0.400171604	1.683394373	1.92E-06	0.000237553	0.000182	2777	tags=39%, list=22%, signal=31%
CC	GO:0030027	lamellipodium	143	0.437314397	1.789525308	2.00E-06	0.000243089	0.000186	3861	tags=51%, list=31%, signal=36%
BP	GO:0044770	cell cycle phase transition	399	0.359304242	1.546326682	2.08E-06	0.000247937	0.00019	4447	tags=47%, list=35%, signal=31%
CC	GO:0015629	actin cytoskeleton	302	0.37451609	1.595340749	2.19E-06	0.000255668	0.000196	4283	tags=49%, list=34%, signal=33%
CC	GO:0005788	endoplasmic reticulum lumen	164	0.423779174	1.74856601	2.76E-06	0.000316802	0.000243	3448	tags=46%, list=27%, signal=34%
BP	GO:0051056	regulation of small GTPase mediated signal transduction	202	0.405294839	1.698599777	3.12E-06	0.000344934	0.000264	3335	tags=44%, list=26%, signal=33%
BP	GO:0002040	sprouting angiogenesis	81	0.502072717	1.933892472	3.13E-06	0.000344934	0.000264	3344	tags=56%, list=27%, signal=41%
BP	GO:0031424	keratinization	24	-0.627957558	-2.547448905	3.17E-06	0.000344934	0.000264	1136	tags=58%, list=9%, signal=53%
BP	GO:0071363	cellular response to growth factor stimulus	399	0.356550974	1.534477525	3.28E-06	0.000351569	0.000269	3533	tags=40%, list=28%, signal=29%
MF	GO:0016614	oxidoreductase activity, acting on CH-OH group of donors	80	-0.365908362	-2.126714976	3.83E-06	0.000403547	0.000309	2512	tags=42%, list=20%, signal=34%

BP	GO:0045664	regulation of neuron differentiation	363	0.359332568	1.539399455	4.53E-06	0.00046922	0.00036	3474	tags=40%, list=28%, signal=30%
BP	GO:0006720	isoprenoid metabolic process	70	-0.390840246	-2.145752172	4.77E-06	0.000479448	0.000367	2129	tags=41%, list=17%, signal=35%
BP	GO:0043903	regulation of symbiotic process	150	0.425653932	1.747938103	4.78E-06	0.000479448	0.000367	5275	tags=67%, list=42%, signal=40%
BP	GO:0007507	heart development	311	0.372192311	1.585467766	4.95E-06	0.000488192	0.000374	3460	tags=40%, list=27%, signal=30%
BP	GO:0001936	regulation of endothelial cell proliferation	76	0.507665002	1.943519434	5.09E-06	0.000494953	0.000379	3216	tags=49%, list=26%, signal=36%
BP	GO:0006325	chromatin organization	440	0.343465563	1.480549881	5.63E-06	0.000539068	0.000413	4769	tags=49%, list=38%, signal=32%
BP	GO:0030155	regulation of cell adhesion	414	0.349586923	1.504642058	6.05E-06	0.000570115	0.000437	3371	tags=39%, list=27%, signal=30%
BP	GO:0070848	response to growth factor	418	0.350568852	1.508882949	6.74E-06	0.000625928	0.00048	3533	tags=39%, list=28%, signal=29%
BP	GO:0120035	regulation of plasma membrane bounded cell projection organization	384	0.349672529	1.50268863	7.02E-06	0.000642164	0.000492	3310	tags=38%, list=26%, signal=29%
BP	GO:0006614	SRP-dependent cotranslational protein targeting to membrane	90	-0.344741182	-2.020254039	7.15E-06	0.000644999	0.000494	2492	tags=38%, list=20%, signal=31%
BP	GO:0006898	receptor-mediated endocytosis	145	0.429033718	1.757734507	7.52E-06	0.000668493	0.000512	4076	tags=51%, list=32%, signal=35%
CC	GO:0005911	cell-cell junction	274	0.373665649	1.58362795	7.73E-06	0.000677356	0.000519	2759	tags=37%, list=22%, signal=29%
BP	GO:0032970	regulation of actin filament-based process	243	0.385836722	1.627540774	7.87E-06	0.000677356	0.000519	2777	tags=38%, list=22%, signal=30%
BP	GO:0045601	regulation of endothelial cell differentiation	24	0.661387814	2.103686736	7.94E-06	0.000677356	0.000519	2676	tags=62%, list=21%, signal=49%
BP	GO:1901342	regulation of vasculature development	207	0.397277571	1.666651129	8.30E-06	0.000691802	0.00053	3496	tags=43%, list=28%, signal=31%
BP	GO:0016101	diterpenoid metabolic process	50	-0.435868318	-2.301114256	8.47E-06	0.000691802	0.00053	2069	tags=48%, list=16%, signal=40%
BP	GO:0010564	regulation of cell cycle process	470	0.341116123	1.473945683	8.54E-06	0.000691802	0.00053	4641	tags=47%, list=37%, signal=31%
BP	GO:0051640	organelle localization	392	0.351808451	1.512437648	8.54E-06	0.000691802	0.00053	4281	tags=44%, list=34%, signal=30%
BP	GO:0071559	response to transforming growth factor beta	160	0.418507494	1.725725778	9.58E-06	0.000766146	0.000587	3424	tags=44%, list=27%, signal=33%
BP	GO:0043534	blood vessel endothelial cell migration	82	0.475867189	1.839706771	1.03E-05	0.000809609	0.000621	3371	tags=50%, list=27%, signal=37%
BP	GO:0043087	regulation of GTPase activity	287	0.367682773	1.560531433	1.07E-05	0.000830318	0.000636	2747	tags=34%, list=22%, signal=28%
CC	GO:0031252	cell leading edge	262	0.375213738	1.586936545	1.11E-05	0.000851504	0.000653	4357	tags=48%, list=35%, signal=32%
MF	GO:0140297	DNA-binding transcription factor binding	233	0.379354379	1.597271278	1.16E-05	0.00088623	0.000679	5057	tags=54%, list=40%, signal=33%
BP	GO:0034446	substrate adhesion-dependent cell spreading	72	0.49302285	1.883861829	1.33E-05	0.000999199	0.000766	3422	tags=54%, list=27%, signal=40%
BP	GO:0072330	monocarboxylic acid biosynthetic process	130	-0.287916499	-1.668796716	1.41E-05	0.0010502	0.000805	2245	tags=38%, list=18%, signal=31%
CC	GO:0001533	cornified envelope	11	-0.806515781	-2.490187166	1.51E-05	0.001092158	0.000837	1136	tags=82%, list=9%, signal=75%
BP	GO:0007160	cell-matrix adhesion	136	0.427404646	1.743284901	1.51E-05	0.001092158	0.000837	3335	tags=46%, list=26%, signal=34%
CC	GO:0005815	microtubule organizing center	422	0.341550914	1.470346737	1.52E-05	0.001092158	0.000837	4455	tags=45%, list=35%, signal=30%
BP	GO:0001938	positive regulation of endothelial cell proliferation	56	0.528966133	1.967110094	1.55E-05	0.00109835	0.000842	3060	tags=48%, list=24%, signal=37%
CC	GO:0005684	U2-type spliceosomal complex	70	0.498770695	1.902453647	1.75E-05	0.001228445	0.000942	5489	tags=74%, list=44%, signal=42%
BP	GO:0034329	cell junction assembly	216	0.385111832	1.61718666	1.81E-05	0.001254952	0.000962	3290	tags=41%, list=26%, signal=31%
MF	GO:0016616	oxidoreductase activity, acting on the CH-OH group of donors, NAD or NADP as acceptor	74	-0.352778505	-1.980211917	1.89E-05	0.001295576	0.000993	2808	tags=43%, list=22%, signal=34%
BP	GO:0034330	cell junction organization	354	0.35285142	1.510191393	2.00E-05	0.001357145	0.00104	3614	tags=43%, list=29%, signal=31%
BP	GO:0040017	positive regulation of locomotion	314	0.358528951	1.528311159	2.11E-05	0.001417823	0.001087	3291	tags=39%, list=26%, signal=29%
CC	GO:0005819	spindle	206	0.388589426	1.630551885	2.14484E-05	0.001420294	0.001089	4138	tags=48%, list=33%, signal=32%
BP	GO:0003197	endocardial cushion development	25	0.654246731	2.090477354	2.15912E-05	0.001420294	0.001089	2346	tags=60%, list=19%, signal=49%
BP	GO:0007229	integrin-mediated signaling pathway	70	0.496534406	1.893923806	2.19211E-05	0.001427131	0.001094	3335	tags=51%, list=26%, signal=38%
BP	GO:0022604	regulation of cell morphogenesis	296	0.359721731	1.52951671	2.25022E-05	0.001437665	0.001102	3674	tags=44%, list=29%, signal=32%
BP	GO:0010810	regulation of cell-substrate adhesion	128	0.429427701	1.742746743	2.25382E-05	0.001437665	0.001102	3167	tags=46%, list=25%, signal=35%

BP	GO:0043547	positive regulation of GTPase activity	236	0.371013978	1.563425438	2.28274E-05	0.001441549	0.001105	2747	tags=36%, list=22%, signal=28%
BP	GO:0070268	cornification	17	-0.682546021	-2.57218661	2.3123E-05	0.00144576	0.001108	1136	tags=65%, list=9%, signal=59%
BP	GO:0030335	positive regulation of cell migration	300	0.361889801	1.540378199	2.36203E-05	0.001462375	0.001121	3291	tags=39%, list=26%, signal=29%
CC	GO:0005912	adherens junction	95	0.459403084	1.808567773	2.38977E-05	0.001465183	0.001123	3319	tags=52%, list=26%, signal=38%
BP	GO:0032092	positive regulation of protein binding	63	0.511077714	1.919374067	2.42876E-05	0.001474769	0.00113	4488	tags=67%, list=36%, signal=43%
BP	GO:0071560	cellular response to transforming growth factor beta stimulus	155	0.417368567	1.717059444	2.49883E-05	0.001502868	0.001152	3424	tags=45%, list=27%, signal=33%
BP	GO:0031344	regulation of cell projection organization	388	0.345687379	1.485682212	2.58307E-05	0.001530408	0.001173	3310	tags=38%, list=26%, signal=29%
BP	GO:0071711	basement membrane organization	20	0.681674174	2.06649494	2.611E-05	0.001530408	0.001173	2256	tags=70%, list=18%, signal=58%
MF	GO:0019901	protein kinase binding	414	0.340402096	1.465110037	2.63892E-05	0.001530408	0.001173	4372	tags=47%, list=35%, signal=31%
MF	GO:0003682	chromatin binding	360	0.34934278	1.496290542	2.64156E-05	0.001530408	0.001173	4265	tags=45%, list=34%, signal=30%
BP	GO:0150115	cell-substrate junction organization	71	0.475955406	1.816388822	2.7894E-05	0.001601367	0.001227	3274	tags=52%, list=26%, signal=39%
BP	GO:0006281	DNA repair	331	0.352889529	1.507461923	2.83439E-05	0.001606165	0.001231	5432	tags=54%, list=43%, signal=31%
BP	GO:0007178	transmembrane receptor protein serine/threonine kinase signaling pathway	192	0.387063283	1.619982584	2.84862E-05	0.001606165	0.001231	3533	tags=44%, list=28%, signal=32%
BP	GO:0051493	regulation of cytoskeleton organization	339	0.352257015	1.506391012	2.89341E-05	0.00161698	0.001239	4574	tags=49%, list=36%, signal=32%
BP	GO:0045047	protein targeting to ER	102	-0.304592769	-1.809769844	3.15069E-05	0.001728085	0.001324	2492	tags=35%, list=20%, signal=29%
BP	GO:0006613	cotranslational protein targeting to membrane	94	-0.325189059	-1.92109137	3.16426E-05	0.001728085	0.001324	2492	tags=36%, list=20%, signal=29%
CC	GO:0005813	centrosome	335	0.359695259	1.537394655	3.17431E-05	0.001728085	0.001324	4488	tags=48%, list=36%, signal=32%
BP	GO:0120031	plasma membrane bounded cell projection assembly	291	0.361103127	1.534820386	3.23049E-05	0.001743639	0.001336	3845	tags=42%, list=30%, signal=30%
BP	GO:0060627	regulation of vesicle-mediated transport	301	0.359391395	1.53049847	3.34286E-05	0.001780357	0.001365	4076	tags=45%, list=32%, signal=31%
BP	GO:0050792	regulation of viral process	142	0.416226597	1.702623343	3.37662E-05	0.001780357	0.001365	5275	tags=66%, list=42%, signal=39%
BP	GO:0030031	cell projection assembly	298	0.359447921	1.529764781	3.39904E-05	0.001780357	0.001365	3845	tags=42%, list=30%, signal=30%
BP	GO:0034340	response to type I interferon	72	0.480239222	1.83501503	3.41129E-05	0.001780357	0.001365	4663	tags=67%, list=37%, signal=42%
BP	GO:1903900	regulation of viral life cycle	99	0.447425253	1.773294863	3.60144E-05	0.00185355	0.001421	5088	tags=69%, list=40%, signal=41%
BP	GO:0006721	terpenoid metabolic process	56	-0.403342995	-2.170432576	3.61024E-05	0.00185355	0.001421	2129	tags=46%, list=17%, signal=39%
CC	GO:0099081	supramolecular polymer	448	0.333774804	1.439615708	3.73239E-05	0.001900812	0.001457	4111	tags=42%, list=33%, signal=30%
BP	GO:0090287	regulation of cellular response to growth factor stimulus	161	0.409888021	1.690020899	3.81904E-05	0.001929379	0.001479	3391	tags=42%, list=27%, signal=31%
BP	GO:1902903	regulation of supramolecular fiber organization	230	0.373561697	1.571875918	4.19817E-05	0.002104083	0.001613	3143	tags=39%, list=25%, signal=30%
CC	GO:0098687	chromosomal region	192	0.383703583	1.605921175	4.25879E-05	0.00211766	0.001623	5637	tags=60%, list=45%, signal=34%
MF	GO:0019904	protein domain specific binding	444	0.332912476	1.435985827	4.79877E-05	0.00236752	0.001815	4518	tags=46%, list=36%, signal=31%
BP	GO:0019884	antigen processing and presentation of exogenous antigen	138	0.410027553	1.67600199	5.06885E-05	0.002466253	0.00189	4737	tags=58%, list=38%, signal=37%
BP	GO:0006635	fatty acid beta-oxidation	49	-0.413360103	-2.171248771	5.07701E-05	0.002466253	0.00189	3088	tags=53%, list=24%, signal=40%
BP	GO:1903901	negative regulation of viral life cycle	54	0.515623496	1.905111963	5.25947E-05	0.002535387	0.001943	2428	tags=48%, list=19%, signal=39%
MF	GO:0050839	cell adhesion molecule binding	337	0.351885338	1.50464851	5.30921E-05	0.002539972	0.001947	3335	tags=38%, list=26%, signal=29%
MF	GO:0015078	proton transmembrane transporter activity	60	-0.363297657	-1.972178299	5.48262E-05	0.002586665	0.001982	2163	tags=38%, list=17%, signal=32%
BP	GO:0009615	response to virus	212	0.377417351	1.584824792	5.48873E-05	0.002586665	0.001982	4073	tags=48%, list=32%, signal=33%
CC	GO:0099512	supramolecular fiber	444	0.332201828	1.432920517	5.69678E-05	0.002664827	0.002042	4111	tags=42%, list=33%, signal=30%
MF	GO:0140098	catalytic activity, acting on RNA	235	0.366234253	1.544192287	6.04727E-05	0.002807981	0.002152	5392	tags=56%, list=43%, signal=33%
BP	GO:0051272	positive regulation of cellular component movement	316	0.350971495	1.497004442	6.15414E-05	0.002836746	0.002174	3291	tags=38%, list=26%, signal=29%
MF	GO:0060090	molecular adaptor activity	185	0.387169708	1.615025521	6.31124E-05	0.002888078	0.002214	3845	tags=47%, list=30%, signal=33%

BP	GO:0001885	endothelial cell development	43	0.542107952	1.945749568	6.64601E-05	0.003019394	0.002314	2958	tags=60%, list=23%, signal=46%
MF	GO:0008146	sulfotransferase activity	24	0.620377191	1.973243596	6.7088E-05	0.003026146	0.002319	945	tags=46%, list=7%, signal=42%
BP	GO:0007179	transforming growth factor beta receptor signaling pathway	126	0.419842188	1.699910306	7.17224E-05	0.00320875	0.002459	4231	tags=52%, list=34%, signal=35%
BP	GO:0110053	regulation of actin filament organization	174	0.395193136	1.639223	7.32728E-05	0.00320875	0.002459	2777	tags=39%, list=22%, signal=31%
BP	GO:0051129	negative regulation of cellular component organization	450	0.33445962	1.442427241	7.38884E-05	0.00320875	0.002459	3985	tags=42%, list=32%, signal=30%
BP	GO:0043254	regulation of protein-containing complex assembly	273	0.358658276	1.519959248	7.40364E-05	0.00320875	0.002459	2883	tags=34%, list=23%, signal=27%
BP	GO:0030198	extracellular matrix organization	207	0.382690578	1.60545606	7.41849E-05	0.00320875	0.002459	3422	tags=42%, list=27%, signal=31%
BP	GO:0043062	extracellular structure organization	207	0.382690578	1.60545606	7.41849E-05	0.00320875	0.002459	3422	tags=42%, list=27%, signal=31%
CC	GO:0022626	cytosolic ribosome	98	-0.310944919	-1.815818718	7.58712E-05	0.003259363	0.002498	2465	tags=36%, list=20%, signal=29%
BP	GO:1903510	mucopolysaccharide metabolic process	68	0.480479796	1.821538893	7.71538E-05	0.00329145	0.002523	2695	tags=43%, list=21%, signal=34%
BP	GO:1903311	regulation of mRNA metabolic process	229	0.371716924	1.56402761	7.76605E-05	0.00329145	0.002523	5239	tags=55%, list=42%, signal=33%
BP	GO:0071897	DNA biosynthetic process	130	0.413660314	1.680208982	8.06567E-05	0.003376533	0.002588	4176	tags=45%, list=33%, signal=31%
BP	GO:2000147	positive regulation of cell motility	307	0.35364116	1.507284292	8.07374E-05	0.003376533	0.002588	3291	tags=38%, list=26%, signal=29%
CC	GO:0005938	cell cortex	167	0.393647479	1.626889006	8.13065E-05	0.003377965	0.002589	4342	tags=51%, list=34%, signal=34%
CC	GO:0005667	transcription regulator complex	256	0.359538449	1.517927915	8.19485E-05	0.003382384	0.002592	4346	tags=48%, list=34%, signal=32%
BP	GO:0045765	regulation of angiogenesis	189	0.386849878	1.614732231	8.80407E-05	0.003610239	0.002767	3496	tags=42%, list=28%, signal=31%
BP	GO:0030041	actin filament polymerization	112	0.421962263	1.69466328	8.92074E-05	0.003634481	0.002786	2777	tags=41%, list=22%, signal=32%
MF	GO:0051015	actin filament binding	114	0.429335767	1.723692471	9.0543E-05	0.00364849	0.002796	2777	tags=41%, list=22%, signal=32%
BP	GO:0072599	establishment of protein localization to endoplasmic reticulum	105	-0.295833281	-1.772994403	9.07067E-05	0.00364849	0.002796	2492	tags=35%, list=20%, signal=29%
BP	GO:1902905	positive regulation of supramolecular fiber organization	130	0.412193924	1.674252789	9.43853E-05	0.003772426	0.002891	3109	tags=42%, list=25%, signal=32%
BP	GO:0050767	regulation of neurogenesis	449	0.329662305	1.422014132	9.53214E-05	0.003785877	0.002902	3474	tags=38%, list=28%, signal=28%
BP	GO:0032271	regulation of protein polymerization	139	0.402632081	1.644792837	9.86617E-05	0.003891959	0.002983	2883	tags=39%, list=23%, signal=30%
BP	GO:0030833	regulation of actin filament polymerization	101	0.439489383	1.743136571	9.92249E-05	0.003891959	0.002983	2777	tags=43%, list=22%, signal=33%
BP	GO:0043648	dicarboxylic acid metabolic process	55	-0.394970824	-2.086623414	0.000100336	0.003911252	0.002998	2185	tags=44%, list=17%, signal=36%
MF	GO:0016628	oxidoreductase activity, acting on the CH-CH group of donors, NAD or NADP as acceptor	20	-0.591610403	-2.317598971	0.000101578	0.003935005	0.003016	1349	tags=60%, list=11%, signal=54%
BP	GO:0044089	positive regulation of cellular component biogenesis	313	0.345750684	1.474221599	0.000102192	0.003935005	0.003016	2883	tags=34%, list=23%, signal=27%
BP	GO:1900026	positive regulation of substrate adhesion-dependent cell spreading	24	0.613503414	1.951380065	0.000102956	0.003936197	0.003017	2790	tags=62%, list=22%, signal=49%
BP	GO:0010811	positive regulation of cell-substrate adhesion	68	0.477291611	1.809452216	0.000103469	0.003936197	0.003017	3167	tags=51%, list=25%, signal=39%
BP	GO:0048002	antigen processing and presentation of peptide antigen	143	0.40272967	1.648001852	0.000109037	0.004123168	0.00316	4737	tags=57%, list=38%, signal=36%
MF	GO:1990782	protein tyrosine kinase binding	56	0.501207315	1.863881083	0.000113526	0.004267366	0.003271	2828	tags=46%, list=22%, signal=36%
BP	GO:0008299	isoprenoid biosynthetic process	20	-0.589528845	-2.309444589	0.000115326	0.004309353	0.003303	1013	tags=35%, list=8%, signal=32%
BP	GO:1901568	fatty acid derivative metabolic process	90	-0.313169922	-1.835239979	0.00011667	0.00432076	0.003312	2220	tags=40%, list=18%, signal=33%
BP	GO:1901990	regulation of mitotic cell cycle phase transition	284	0.352280875	1.495635714	0.000116999	0.00432076	0.003312	4641	tags=48%, list=37%, signal=31%
BP	GO:0051607	defense response to virus	162	0.397923189	1.641373754	0.000117706	0.004321595	0.003312	4746	tags=56%, list=38%, signal=35%
CC	GO:0009986	cell surface	356	0.340976595	1.459869489	0.000119152	0.004349381	0.003333	3214	tags=37%, list=25%, signal=29%
BP	GO:0007346	regulation of mitotic cell cycle	407	0.333143346	1.43418172	0.0001213	0.004377193	0.003355	4671	tags=46%, list=37%, signal=30%
MF	GO:0008270	zinc ion binding	438	0.329728635	1.421049998	0.0001213	0.004377193	0.003355	4044	tags=42%, list=32%, signal=29%
BP	GO:0060337	type I interferon signaling pathway	69	0.473660719	1.802780536	0.000123293	0.00439886	0.003371	4641	tags=65%, list=37%, signal=41%
BP	GO:0071357	cellular response to type I interferon	69	0.473660719	1.802780536	0.000123293	0.00439886	0.003371	4641	tags=65%, list=37%, signal=41%

BP	GO:0042572	retinol metabolic process	19	-0.600357255	-2.343477564	0.000124153	0.004400611	0.003373	2654	tags=68%, list=21%, signal=54%
BP	GO:0060445	branching involved in salivary gland morphogenesis	12	0.760709627	2.03731298	0.000124736	0.004400611	0.003373	2133	tags=67%, list=17%, signal=55%
BP	GO:0002429	immune response-activating cell surface receptor signaling pathway	229	0.365605235	1.538312204	0.000133302	0.004650835	0.003565	3530	tags=40%, list=28%, signal=29%
BP	GO:0002757	immune response-activating signal transduction	229	0.365605235	1.538312204	0.000133302	0.004650835	0.003565	3530	tags=40%, list=28%, signal=29%
BP	GO:0019941	modification-dependent protein catabolic process	453	0.326346304	1.408227092	0.00013717	0.004759514	0.003648	4072	tags=41%, list=32%, signal=29%
BP	GO:0019058	viral life cycle	224	0.368068431	1.54866813	0.000137998	0.004762049	0.00365	5278	tags=60%, list=42%, signal=35%
BP	GO:0000086	G2/M transition of mitotic cell cycle	171	0.383923895	1.590153279	0.000142703	0.004890863	0.003748	4186	tags=47%, list=33%, signal=32%
BP	GO:0002478	antigen processing and presentation of exogenous peptide antigen	136	0.407052293	1.660272347	0.000143279	0.004890863	0.003748	4737	tags=58%, list=38%, signal=37%
BP	GO:0006403	RNA localization	174	0.389849149	1.617056655	0.000149552	0.005077547	0.003892	5218	tags=57%, list=41%, signal=34%
BP	GO:0019221	cytokine-mediated signaling pathway	462	0.325348678	1.404599279	0.00016211	0.005474464	0.004196	3553	tags=37%, list=28%, signal=28%
MF	GO:0019842	vitamin binding	68	-0.336801005	-1.86814641	0.000164257	0.005503483	0.004218	1803	tags=38%, list=14%, signal=33%
BP	GO:0008064	regulation of actin polymerization or depolymerization	111	0.426699117	1.710686989	0.000165584	0.005503483	0.004218	2777	tags=41%, list=22%, signal=32%
BP	GO:0030832	regulation of actin filament length	111	0.426699117	1.710686989	0.000165584	0.005503483	0.004218	2777	tags=41%, list=22%, signal=32%
BP	GO:0007044	cell-substrate junction assembly	68	0.470008341	1.781840733	0.000176707	0.005842424	0.004478	3274	tags=51%, list=26%, signal=38%
BP	GO:0003013	circulatory system process	252	0.355298063	1.500097134	0.000180428	0.005934396	0.004548	2513	tags=32%, list=20%, signal=26%
BP	GO:1901570	fatty acid derivative biosynthetic process	59	-0.356161029	-1.942704909	0.000181632	0.005943041	0.004555	2220	tags=44%, list=18%, signal=36%
BP	GO:0036498	IRE1-mediated unfolded protein response	56	0.494771774	1.839948705	0.000184247	0.005990091	0.004591	2999	tags=52%, list=24%, signal=40%
CC	GO:0099513	polymeric cytoskeletal fiber	322	0.346671071	1.478181299	0.000184967	0.005990091	0.004591	4933	tags=51%, list=39%, signal=32%
BP	GO:0045069	regulation of viral genome replication	67	0.475133192	1.796696272	0.000186719	0.006015967	0.004611	5088	tags=73%, list=40%, signal=44%
BP	GO:0006022	aminoglycan metabolic process	98	0.43419488	1.718192458	0.000192038	0.006155927	0.004718	2695	tags=40%, list=21%, signal=32%
BP	GO:0034440	lipid oxidation	69	-0.338132886	-1.883716975	0.000194452	0.006201855	0.004753	3088	tags=51%, list=24%, signal=39%
BP	GO:0003177	pulmonary valve development	13	0.737785926	2.012752683	0.00019767	0.006272789	0.004808	2346	tags=69%, list=19%, signal=56%
BP	GO:0010975	regulation of neuron projection development	288	0.349502215	1.484115344	0.000201055	0.00630678	0.004834	3420	tags=40%, list=27%, signal=30%
BP	GO:0061437	renal system vasculature development	19	0.659808006	1.970613281	0.00020214	0.00630678	0.004834	1069	tags=47%, list=8%, signal=43%
BP	GO:0061440	kidney vasculature development	19	0.659808006	1.970613281	0.00020214	0.00630678	0.004834	1069	tags=47%, list=8%, signal=43%
BP	GO:0043393	regulation of protein binding	136	0.403074474	1.644047745	0.000202883	0.00630678	0.004834	4488	tags=56%, list=36%, signal=36%
MF	GO:0003729	mRNA binding	205	0.368783127	1.546190718	0.000203734	0.00630678	0.004834	5284	tags=58%, list=42%, signal=34%
BP	GO:0019882	antigen processing and presentation	165	0.388253269	1.602392287	0.000209548	0.006455107	0.004947	4737	tags=55%, list=38%, signal=35%
BP	GO:0030203	glycosaminoglycan metabolic process	92	0.441067652	1.725752885	0.000210928	0.006466057	0.004956	2695	tags=40%, list=21%, signal=32%
BP	GO:0002011	morphogenesis of an epithelial sheet	35	0.553750283	1.900600068	0.000215236	0.006566273	0.005033	4205	tags=74%, list=33%, signal=50%
MF	GO:0003712	transcription coregulator activity	328	0.345146782	1.472981429	0.00021901	0.006649265	0.005096	4731	tags=49%, list=38%, signal=31%
BP	GO:0051972	regulation of telomerase activity	36	0.5444199	1.875732941	0.000223379	0.006749477	0.005173	2994	tags=47%, list=24%, signal=36%
BP	GO:0018212	peptidyl-tyrosine modification	206	0.368109502	1.544616511	0.000228774	0.006879567	0.005273	2502	tags=33%, list=20%, signal=27%
BP	GO:0048729	tissue morphogenesis	374	0.33163465	1.422402676	0.000230357	0.006882473	0.005275	4458	tags=47%, list=35%, signal=32%
BP	GO:0033044	regulation of chromosome organization	223	0.364542934	1.534198817	0.000231051	0.006882473	0.005275	4769	tags=49%, list=38%, signal=31%
BP	GO:0018205	peptidyl-lysine modification	252	0.352680558	1.489045815	0.00023948	0.007100081	0.005442	4760	tags=51%, list=38%, signal=32%
BP	GO:0015849	organic acid transport	154	-0.248503635	-1.534377784	0.000242566	0.007144356	0.005476	1021	tags=19%, list=8%, signal=18%
MF	GO:0017111	nucleoside-triphosphatase activity	464	0.323774659	1.39837124	0.000243798	0.007144356	0.005476	4929	tags=49%, list=39%, signal=31%
BP	GO:0010594	regulation of endothelial cell migration	113	0.420827857	1.690304096	0.000244368	0.007144356	0.005476	3371	tags=44%, list=27%, signal=33%

BP	GO:2000278	regulation of DNA biosynthetic process	78	0.446123704	1.712117181	0.000247544	0.007194309	0.005514	3525	tags=42%, list=28%, signal=31%
BP	GO:0010638	positive regulation of organelle organization	405	0.32720567	1.409182415	0.000248355	0.007194309	0.005514	4671	tags=47%, list=37%, signal=30%
BP	GO:0010769	regulation of cell morphogenesis involved in differentiation	186	0.37751658	1.575609936	0.000250104	0.007211891	0.005527	3405	tags=45%, list=27%, signal=33%
BP	GO:0050907	detection of chemical stimulus involved in sensory perception	18	-0.586072124	-2.215334772	0.000253112	0.00726547	0.005568	2316	tags=50%, list=18%, signal=41%
BP	GO:0048013	ephrin receptor signaling pathway	64	0.470927426	1.773958056	0.000255037	0.007287609	0.005585	3444	tags=52%, list=27%, signal=38%
BP	GO:0006029	proteoglycan metabolic process	56	0.489616104	1.820775888	0.00026241	0.007462553	0.00572	2998	tags=46%, list=24%, signal=36%
BP	GO:0022406	membrane docking	109	0.424030678	1.698159065	0.000263523	0.007462553	0.00572	4138	tags=50%, list=33%, signal=34%
BP	GO:0010632	regulation of epithelial cell migration	156	0.39683013	1.633612086	0.000273599	0.007713286	0.005912	3832	tags=46%, list=30%, signal=33%
BP	GO:0019079	viral genome replication	83	0.449015488	1.737852381	0.000284199	0.007976516	0.006113	5088	tags=65%, list=40%, signal=39%
BP	GO:0008037	cell recognition	63	0.47314882	1.776930495	0.00028935	0.008081424	0.006194	2345	tags=41%, list=19%, signal=34%
BP	GO:0006023	aminoglycan biosynthetic process	70	0.461434163	1.76004147	0.000290496	0.008081424	0.006194	2602	tags=41%, list=21%, signal=33%
BP	GO:0009611	response to wounding	344	0.33606644	1.43791789	0.000294217	0.008149037	0.006246	3811	tags=41%, list=30%, signal=29%
BP	GO:0002768	immune response-regulating cell surface receptor signaling pathway	254	0.35037726	1.478708539	0.000299377	0.00825575	0.006327	3530	tags=37%, list=28%, signal=27%
CC	GO:0070603	SWI/SNF superfamily-type complex	63	0.470536031	1.767118054	0.000318527	0.008745646	0.006703	3088	tags=48%, list=24%, signal=36%
BP	GO:0043632	modification-dependent macromolecule catabolic process	461	0.322351144	1.391778712	0.000321264	0.008782613	0.006731	4234	tags=42%, list=34%, signal=29%
BP	GO:1904018	positive regulation of vasculature development	120	0.416569949	1.679936144	0.000323164	0.008796474	0.006742	3496	tags=42%, list=28%, signal=31%
MF	GO:0016903	oxidoreductase activity, acting on the aldehyde or oxo group of donors	31	-0.464301536	-2.128465307	0.000329573	0.008924885	0.00684	2215	tags=52%, list=18%, signal=43%
BP	GO:0010720	positive regulation of cell development	304	0.341637688	1.455769702	0.000330708	0.008924885	0.00684	3124	tags=36%, list=25%, signal=28%
CC	GO:0005905	clathrin-coated pit	44	0.516902829	1.854810497	0.000334443	0.008933454	0.006847	4076	tags=64%, list=32%, signal=43%
BP	GO:0120254	olefinic compound metabolic process	54	-0.350342965	-1.895229935	0.000335078	0.008933454	0.006847	2220	tags=39%, list=18%, signal=32%
BP	GO:1901987	regulation of cell cycle phase transition	299	0.340647951	1.450371882	0.00033527	0.008933454	0.006847	4641	tags=46%, list=37%, signal=30%
BP	GO:0006511	ubiquitin-dependent protein catabolic process	447	0.323085772	1.393467497	0.000339492	0.009007941	0.006904	4072	tags=41%, list=32%, signal=29%
BP	GO:0007043	cell-cell junction assembly	79	0.450245769	1.730106503	0.000344037	0.009090346	0.006967	2509	tags=38%, list=20%, signal=31%
BP	GO:0044242	cellular lipid catabolic process	126	-0.262488107	-1.55755125	0.000355598	0.009356668	0.007171	2321	tags=33%, list=18%, signal=27%
BP	GO:0000209	protein polyubiquitination	248	0.353303967	1.490506438	0.000363365	0.00952137	0.007297	4686	tags=47%, list=37%, signal=30%
MF	GO:0005096	GTPase activator activity	164	0.380444096	1.569760044	0.000374759	0.009690217	0.007427	2835	tags=36%, list=22%, signal=28%
BP	GO:0140056	organelle localization by membrane tethering	103	0.41848112	1.665637473	0.000375844	0.009690217	0.007427	4138	tags=49%, list=33%, signal=33%
MF	GO:0016817	hydrolase activity, acting on acid anhydrides	498	0.317100268	1.373056278	0.000375947	0.009690217	0.007427	4929	tags=49%, list=39%, signal=31%
MF	GO:0016818	hydrolase activity, acting on acid anhydrides, in phosphorus-containing anhydrides	498	0.317100268	1.373056278	0.000375947	0.009690217	0.007427	4929	tags=49%, list=39%, signal=31%
BP	GO:0030111	regulation of Wnt signaling pathway	221	0.361845459	1.52094902	0.000377834	0.009699274	0.007434	4769	tags=51%, list=38%, signal=32%
MF	GO:0016462	pyrophosphatase activity	495	0.318390096	1.378396871	0.000380504	0.009704198	0.007438	4929	tags=49%, list=39%, signal=31%
BP	GO:0060973	cell migration involved in heart development	10	0.770931961	1.946615872	0.000382164	0.009704198	0.007438	1331	tags=60%, list=11%, signal=54%
BP	GO:0019395	fatty acid oxidation	66	-0.332373172	-1.829728368	0.000382636	0.009704198	0.007438	2248	tags=50%, list=18%, signal=41%
BP	GO:0010959	regulation of metal ion transport	181	0.372192216	1.548966701	0.000388163	0.009804987	0.007515	2586	tags=32%, list=21%, signal=26%
MF	GO:0004984	olfactory receptor activity	17	-0.600255053	-2.262071659	0.0003941	0.009875958	0.007569	2316	tags=53%, list=18%, signal=43%
BP	GO:0050911	detection of chemical stimulus involved in sensory perception of smell	17	-0.600255053	-2.262071659	0.0003941	0.009875958	0.007569	2316	tags=53%, list=18%, signal=43%
BP	GO:0035966	response to topologically incorrect protein	152	0.378914214	1.556508038	0.000398152	0.009928986	0.00761	4017	tags=43%, list=32%, signal=30%
BP	GO:0043123	positive regulation of I-kappaB kinase/NF-kappaB signaling	132	0.393785881	1.600949075	0.000399361	0.009928986	0.00761	4552	tags=55%, list=36%, signal=35%
CC	GO:0098589	membrane region	184	0.36476939	1.520057472	0.000402349	0.009948294	0.007625	2677	tags=35%, list=21%, signal=28%

BP	GO:0051960	regulation of nervous system development	491	0.315910186	1.366971505	0.000403288	0.009948294	0.007625	3474	tags=36%, list=28%, signal=27%
BP	GO:0072012	glomerulus vasculature development	17	0.645527755	1.879324418	0.000430491	0.010525449	0.008067	1069	tags=47%, list=8%, signal=43%
BP	GO:0010498	proteasomal protein catabolic process	350	0.329778704	1.411470379	0.00043106	0.010525449	0.008067	4686	tags=47%, list=37%, signal=31%
MF	GO:0004029	aldehyde dehydrogenase (NAD+) activity	12	-0.722624685	-2.333027238	0.000433352	0.010525449	0.008067	1743	tags=75%, list=14%, signal=65%
MF	GO:0004030	aldehyde dehydrogenase [NAD(P)+] activity	12	-0.722624685	-2.333027238	0.000433352	0.010525449	0.008067	1743	tags=75%, list=14%, signal=65%
BP	GO:0002764	immune response-regulating signaling pathway	256	0.345114702	1.457032598	0.000440623	0.010661058	0.008171	3530	tags=37%, list=28%, signal=27%
BP	GO:0016126	sterol biosynthetic process	55	-0.366128357	-1.934249203	0.000445448	0.010725742	0.008221	3103	tags=51%, list=25%, signal=39%
BP	GO:0002479	antigen processing and presentation of exogenous peptide antigen via MHC class I, TAP-dependent	67	0.463609109	1.753118435	0.000447916	0.010725742	0.008221	4595	tags=63%, list=36%, signal=40%
BP	GO:0003279	cardiac septum development	65	0.466600822	1.760603645	0.000448392	0.010725742	0.008221	2564	tags=46%, list=20%, signal=37%
BP	GO:0010634	positive regulation of epithelial cell migration	99	0.419225891	1.661531431	0.000451469	0.010745465	0.008236	3832	tags=46%, list=30%, signal=33%
BP	GO:0032412	regulation of ion transmembrane transporter activity	112	0.403735214	1.621460737	0.000453358	0.010745465	0.008236	2110	tags=32%, list=17%, signal=27%
BP	GO:0002253	activation of immune response	274	0.345096891	1.462551035	0.000454321	0.010745465	0.008236	3530	tags=38%, list=28%, signal=28%
BP	GO:0046942	carboxylic acid transport	151	-0.244305773	-1.466326865	0.000456075	0.010746699	0.008237	1021	tags=19%, list=8%, signal=18%
CC	GO:1904949	ATPase complex	66	0.458478655	1.732408561	0.000462934	0.010867772	0.008329	3088	tags=45%, list=24%, signal=35%
BP	GO:0007059	chromosome segregation	173	0.37198415	1.540028635	0.000466724	0.010916148	0.008366	4776	tags=47%, list=38%, signal=30%
BP	GO:0061061	muscle structure development	347	0.332009162	1.420868644	0.000472158	0.011002494	0.008433	3943	tags=40%, list=31%, signal=29%
BP	GO:0034035	purine ribonucleoside bisphosphate metabolic process	14	0.677018035	1.862248131	0.00048	0.011064048	0.00848	945	tags=50%, list=7%, signal=46%
BP	GO:0050427	3'-phosphoadenosine 5'-phosphosulfate metabolic process	14	0.677018035	1.862248131	0.00048	0.011064048	0.00848	945	tags=50%, list=7%, signal=46%
BP	GO:0043161	proteasome-mediated ubiquitin-dependent protein catabolic process	315	0.334681887	1.426988928	0.000481326	0.011064048	0.00848	4686	tags=47%, list=37%, signal=30%
BP	GO:0042176	regulation of protein catabolic process	271	0.343183055	1.453620475	0.000481807	0.011064048	0.00848	4780	tags=51%, list=38%, signal=33%
BP	GO:0045596	negative regulation of cell differentiation	389	0.326080639	1.401671597	0.000490003	0.01121149	0.008593	3783	tags=40%, list=30%, signal=29%
CC	GO:0071013	catalytic step 2 spliceosome	67	0.46180242	1.746286517	0.000501229	0.011426935	0.008758	5489	tags=67%, list=44%, signal=38%
BP	GO:0045071	negative regulation of viral genome replication	39	0.522581988	1.825573357	0.000503104	0.011428428	0.008759	3365	tags=56%, list=27%, signal=41%
BP	GO:0018108	peptidyl-tyrosine phosphorylation	203	0.361742745	1.515247691	0.000510873	0.01156082	0.008861	2502	tags=32%, list=20%, signal=26%
MF	GO:0019955	cytokine binding	64	0.459606039	1.731311005	0.000512594	0.01156082	0.008861	2402	tags=39%, list=19%, signal=32%
BP	GO:0006913	nucleocytoplasmic transport	245	0.349001829	1.471945841	0.000528221	0.011831635	0.009068	5481	tags=54%, list=43%, signal=31%
BP	GO:0034502	protein localization to chromosome	48	0.498088423	1.811719739	0.000528349	0.011831635	0.009068	2690	tags=42%, list=21%, signal=33%
MF	GO:0016836	hydro-lyase activity	30	-0.456975509	-2.119228677	0.000540908	0.012070086	0.009251	1448	tags=40%, list=11%, signal=35%
CC	GO:0097225	sperm midpiece	11	-0.722917893	-2.232071464	0.000543346	0.012081796	0.00926	1501	tags=73%, list=12%, signal=64%
BP	GO:0043487	regulation of RNA stability	142	0.38451761	1.572914039	0.000560271	0.012414426	0.009515	3535	tags=41%, list=28%, signal=30%
BP	GO:0030968	endoplasmic reticulum unfolded protein response	95	0.420882316	1.656920074	0.000565285	0.012432394	0.009529	3188	tags=42%, list=25%, signal=32%
CC	GO:0005874	microtubule	219	0.358894647	1.50815227	0.000565533	0.012432394	0.009529	4251	tags=46%, list=34%, signal=31%
BP	GO:0044839	cell cycle G2/M phase transition	179	0.376088717	1.564123789	0.00056724	0.012432394	0.009529	4186	tags=46%, list=33%, signal=31%
CC	GO:0098978	glutamatergic synapse	165	0.375490918	1.549719731	0.000568957	0.012432394	0.009529	4238	tags=48%, list=34%, signal=32%
MF	GO:0017081	chloride channel regulator activity	10	-0.721772789	-2.190129868	0.000571819	0.012434778	0.00953	1689	tags=60%, list=13%, signal=52%
MF	GO:0008047	enzyme activator activity	323	0.336227057	1.433873725	0.000573004	0.012434778	0.00953	4319	tags=44%, list=34%, signal=30%
MF	GO:0005178	integrin binding	84	0.437370809	1.696888509	0.000580804	0.012560053	0.009626	3109	tags=44%, list=25%, signal=33%
BP	GO:0042060	wound healing	278	0.347814503	1.475025184	0.000582755	0.012560053	0.009626	3811	tags=43%, list=30%, signal=31%

BP	GO:0050684	regulation of mRNA processing	92	0.427828477	1.67395234	0.0005855	0.012576293	0.009639	5239	tags=62%, list=42%, signal=36%
BP	GO:0051495	positive regulation of cytoskeleton organization	141	0.392723443	1.605662826	0.000597917	0.012799474	0.00981	2883	tags=38%, list=23%, signal=29%
BP	GO:0060828	regulation of canonical Wnt signaling pathway	180	0.365942234	1.521979867	0.00060535	0.01287134	0.009865	4488	tags=48%, list=36%, signal=31%
MF	GO:0061629	RNA polymerase II-specific DNA-binding transcription factor binding	184	0.359855696	1.499581256	0.00060535	0.01287134	0.009865	5057	tags=53%, list=40%, signal=32%
BP	GO:0043687	post-translational protein modification	234	0.350470277	1.477785387	0.000610286	0.012883972	0.009875	4672	tags=47%, list=37%, signal=30%
BP	GO:0034754	cellular hormone metabolic process	57	-0.347926795	-1.875514216	0.000611171	0.012883972	0.009875	2069	tags=42%, list=16%, signal=35%
BP	GO:0001523	retinoid metabolic process	46	-0.386265525	-2.007851212	0.000612147	0.012883972	0.009875	2069	tags=43%, list=16%, signal=36%
BP	GO:1901343	negative regulation of vasculature development	75	0.449988113	1.722566888	0.000614105	0.012883972	0.009875	3344	tags=48%, list=27%, signal=35%
BP	GO:0051099	positive regulation of binding	123	0.400194095	1.615068169	0.000620219	0.012969154	0.00994	3351	tags=44%, list=27%, signal=33%
MF	GO:0060589	nucleoside-triphosphatase regulator activity	217	0.356239642	1.496551697	0.000629901	0.013128135	0.010062	3355	tags=36%, list=27%, signal=27%
BP	GO:0017144	drug metabolic process	13	-0.655834824	-2.198525049	0.000634873	0.01318823	0.010108	1095	tags=69%, list=9%, signal=63%
BP	GO:0007163	establishment or maintenance of cell polarity	135	0.393206284	1.602292949	0.000643615	0.013326003	0.010213	4273	tags=51%, list=34%, signal=34%
MF	GO:0046875	ephrin receptor binding	22	0.615922459	1.902748095	0.000658022	0.013579773	0.010408	2484	tags=55%, list=20%, signal=44%
BP	GO:0050852	T cell receptor signaling pathway	136	0.385222645	1.571234252	0.000671397	0.013765815	0.010551	3099	tags=38%, list=25%, signal=29%
BP	GO:1902749	regulation of cell cycle G2/M phase transition	142	0.382426683	1.564360858	0.000671397	0.013765815	0.010551	2755	tags=35%, list=22%, signal=28%
MF	GO:0030695	GTPase regulator activity	187	0.367436914	1.533259867	0.000677237	0.013840619	0.010608	3486	tags=40%, list=28%, signal=29%
BP	GO:0033209	tumor necrosis factor-mediated signaling pathway	116	0.398310418	1.598554199	0.000679635	0.013844819	0.010611	3803	tags=46%, list=30%, signal=32%
BP	GO:0048024	regulation of mRNA splicing, via spliceosome	66	0.451466456	1.705912247	0.000696615	0.014069228	0.010783	5239	tags=68%, list=42%, signal=40%
CC	GO:0098857	membrane microdomain	177	0.368834827	1.532594649	0.000697066	0.014069228	0.010783	2677	tags=36%, list=21%, signal=29%
BP	GO:0045744	negative regulation of G protein-coupled receptor signaling pathway	25	0.5881556	1.879300123	0.000697335	0.014069228	0.010783	1944	tags=52%, list=15%, signal=44%
BP	GO:1901617	organic hydroxy compound biosynthetic process	136	-0.250282206	-1.455631583	0.00071278	0.014335057	0.010987	2389	tags=35%, list=19%, signal=29%
BP	GO:0010389	regulation of G2/M transition of mitotic cell cycle	135	0.391722897	1.596248231	0.00072696	0.014573814	0.01117	2755	tags=36%, list=22%, signal=29%
BP	GO:0051169	nuclear transport	247	0.346705945	1.462459909	0.000730319	0.01459482	0.011186	5481	tags=54%, list=43%, signal=31%
BP	GO:0032835	glomerulus development	35	0.529283236	1.81662346	0.000745996	0.014861097	0.01139	2112	tags=46%, list=17%, signal=38%
BP	GO:0046578	regulation of Ras protein signal transduction	122	0.400175806	1.61543114	0.000750789	0.014892552	0.011414	3861	tags=51%, list=31%, signal=36%
BP	GO:0016042	lipid catabolic process	165	-0.234185454	-1.41684349	0.000752292	0.014892552	0.011414	2531	tags=34%, list=20%, signal=27%
BP	GO:0050878	regulation of body fluid levels	268	0.340660579	1.441442842	0.000757119	0.01494127	0.011451	2739	tags=32%, list=22%, signal=25%
BP	GO:0050851	antigen receptor-mediated signaling pathway	160	0.37680452	1.553762557	0.000770151	0.01514618	0.011608	3914	tags=43%, list=31%, signal=30%
BP	GO:0016055	Wnt signaling pathway	310	0.332854512	1.418386587	0.000774698	0.01514618	0.011608	4802	tags=48%, list=38%, signal=31%
BP	GO:0198738	cell-cell signaling by wnt	310	0.332854512	1.418386587	0.000774698	0.01514618	0.011608	4802	tags=48%, list=38%, signal=31%
BP	GO:0034620	cellular response to unfolded protein	112	0.396578826	1.592719617	0.000780357	0.015189679	0.011642	4017	tags=46%, list=32%, signal=31%
BP	GO:0016458	gene silencing	149	0.375527326	1.541510345	0.000781733	0.015189679	0.011642	5188	tags=56%, list=41%, signal=34%
BP	GO:0032535	regulation of cellular component size	221	0.354500223	1.490074707	0.000787014	0.015245372	0.011684	2777	tags=34%, list=22%, signal=27%
BP	GO:0006986	response to unfolded protein	138	0.381969082	1.561312006	0.000790187	0.015260036	0.011696	4017	tags=44%, list=32%, signal=30%
MF	GO:0017048	Rho GTPase binding	100	0.408179572	1.617779943	0.000796195	0.015329181	0.011749	2363	tags=36%, list=19%, signal=29%
MF	GO:0051287	NAD binding	44	-0.367303324	-1.872262215	0.000802323	0.01540021	0.011803	2185	tags=39%, list=17%, signal=32%
BP	GO:0003203	endocardial cushion morphogenesis	19	0.630087034	1.881847245	0.000805301	0.015410531	0.011811	2307	tags=58%, list=18%, signal=47%
MF	GO:0003725	double-stranded RNA binding	54	0.475868981	1.758228039	0.000808651	0.015427898	0.011824	4538	tags=65%, list=36%, signal=42%
BP	GO:0006862	nucleotide transport	24	-0.484818243	-1.966772575	0.000813754	0.015460574	0.011849	799	tags=29%, list=6%, signal=27%

BP	GO:0048525	negative regulation of viral process	64	0.452280782	1.70371716	0.000815261	0.015460574	0.011849	2428	tags=42%, list=19%, signal=34%
MF	GO:0031491	nucleosome binding	42	0.508097583	1.803945114	0.00082369	0.015473677	0.011859	3620	tags=55%, list=29%, signal=39%
BP	GO:0031334	positive regulation of protein-containing complex assembly	151	0.379746528	1.559022854	0.000827989	0.015473677	0.011859	3030	tags=37%, list=24%, signal=29%
BP	GO:0007169	transmembrane receptor protein tyrosine kinase signaling pathway	442	0.316176075	1.363763593	0.000828876	0.015473677	0.011859	4570	tags=45%, list=36%, signal=29%
BP	GO:1905114	cell surface receptor signaling pathway involved in cell-cell signaling	348	0.32813284	1.404590321	0.000829705	0.015473677	0.011859	4802	tags=47%, list=38%, signal=30%
BP	GO:0070831	basement membrane assembly	11	0.709941306	1.856234457	0.000831827	0.015473677	0.011859	3614	tags=91%, list=29%, signal=65%
MF	GO:0019825	oxygen binding	13	0.699357514	1.907916189	0.000833996	0.015473677	0.011859	888	tags=38%, list=7%, signal=36%
MF	GO:0003714	transcription corepressor activity	122	0.39892595	1.610385718	0.000834728	0.015473677	0.011859	4731	tags=54%, list=38%, signal=34%
CC	GO:0031253	cell projection membrane	170	0.370392704	1.533355135	0.000835554	0.015473677	0.011859	2927	tags=35%, list=23%, signal=27%
BP	GO:0032273	positive regulation of protein polymerization	82	0.423046962	1.635503308	0.000848713	0.015671406	0.012011	3030	tags=41%, list=24%, signal=32%
BP	GO:0035967	cellular response to topologically incorrect protein	126	0.391601299	1.585565013	0.000852513	0.015679777	0.012017	4017	tags=44%, list=32%, signal=31%
BP	GO:0000380	alternative mRNA splicing, via spliceosome	44	0.500801863	1.797035149	0.000854132	0.015679777	0.012017	5218	tags=73%, list=41%, signal=43%
BP	GO:1904837	beta-catenin-TCF complex assembly	24	0.578053907	1.838625252	0.00085748	0.015695615	0.01203	4621	tags=79%, list=37%, signal=50%
BP	GO:0006909	phagocytosis	169	0.369001993	1.52720466	0.000863252	0.015755592	0.012076	3783	tags=42%, list=30%, signal=30%
CC	GO:0030425	dendrite	296	0.332972242	1.415779375	0.000884713	0.016100752	0.01234	4820	tags=51%, list=38%, signal=32%
CC	GO:0016607	nuclear speck	259	0.34153483	1.443736483	0.000903049	0.016387218	0.01256	5457	tags=58%, list=43%, signal=34%
BP	GO:0003205	cardiac chamber development	90	0.419470888	1.639480046	0.000922537	0.016692893	0.012794	2564	tags=40%, list=20%, signal=32%
BP	GO:0061028	establishment of endothelial barrier	29	0.553926059	1.827072292	0.000931709	0.016810692	0.012884	2747	tags=59%, list=22%, signal=46%
BP	GO:0045766	positive regulation of angiogenesis	105	0.406571903	1.620265481	0.000934648	0.016815677	0.012888	1625	tags=29%, list=13%, signal=25%
BP	GO:0051168	nuclear export	146	0.378229025	1.550427616	0.000939003	0.016846041	0.012911	5481	tags=60%, list=43%, signal=34%
BP	GO:0042590	antigen processing and presentation of exogenous peptide antigen via MHC class I	71	0.429929192	1.640738961	0.000943908	0.016886056	0.012942	4595	tags=59%, list=36%, signal=38%
BP	GO:0016570	histone modification	275	0.337773223	1.431735187	0.000949928	0.016945758	0.012988	4769	tags=51%, list=38%, signal=32%
BP	GO:0051983	regulation of chromosome segregation	61	0.457050392	1.707540441	0.000959099	0.017042179	0.013062	3838	tags=44%, list=30%, signal=31%
BP	GO:0016569	covalent chromatin modification	282	0.335915309	1.426249442	0.000968373	0.017042179	0.013062	4769	tags=50%, list=38%, signal=32%
MF	GO:0003727	single-stranded RNA binding	58	0.4648779	1.7307824	0.0009689	0.0170422	0.013	3783	tags=57%, list=30%, signal=40%
BP	GO:0015865	purine nucleotide transport	23	-0.503275199	-2.058853236	0.000971728	0.017042179	0.013062	799	tags=30%, list=6%, signal=29%
BP	GO:0015868	purine ribonucleotide transport	23	-0.503275199	-2.058853236	9.72E-04	0.017042179	0.013062	799	tags=30%, list=6%, signal=29%
BP	GO:0051503	adenine nucleotide transport	23	-0.503275199	-2.058853236	9.72E-04	0.017042179	0.013062	799	tags=30%, list=6%, signal=29%
BP	GO:0051258	protein polymerization	181	0.362465456	1.50848647	9.74E-04	0.017042179	0.013062	3077	tags=37%, list=24%, signal=28%
MF	GO:0004386	helicase activity	118	0.393833927	1.583617622	9.87E-04	0.017129531	0.013129	5195	tags=59%, list=41%, signal=35%
BP	GO:0070646	protein modification by small protein removal	205	0.354610681	1.486770145	9.89E-04	0.017129531	0.013129	5347	tags=56%, list=42%, signal=33%
BP	GO:0040029	regulation of gene expression, epigenetic	104	0.402030268	1.600324051	9.91E-04	0.017129531	0.013129	5276	tags=62%, list=42%, signal=37%
BP	GO:0052126	movement in host environment	104	0.402074371	1.600499606	9.91E-04	0.017129531	0.013129	2496	tags=38%, list=20%, signal=31%
MF	GO:0030674	protein-macromolecule adaptor activity	161	0.378938732	1.562413009	9.93E-04	0.017129531	0.013129	3845	tags=47%, list=30%, signal=33%
MF	GO:0016627	oxidoreductase activity, acting on the CH-CH group of donors	47	-0.368692712	-1.913650217	1.02E-03	0.017634924	0.013516	1349	tags=32%, list=11%, signal=29%
BP	GO:0070972	protein localization to endoplasmic reticulum	128	-0.251481634	-1.473648623	1.04E-03	0.017838323	0.013672	2495	tags=33%, list=20%, signal=27%
BP	GO:1904062	regulation of cation transmembrane transport	156	0.381809477	1.571777263	0.001049506	0.017961053	0.013766	2110	tags=31%, list=17%, signal=26%
BP	GO:1902622	regulation of neutrophil migration	22	0.605003494	1.869016514	0.001078264	0.01840334	0.014105	2387	tags=45%, list=19%, signal=37%
MF	GO:0045309	protein phosphorylated amino acid binding	39	0.507502638	1.772895575	0.001094349	0.018627535	0.014277	2484	tags=44%, list=20%, signal=35%

BP	GO:0016525	negative regulation of angiogenesis	70	0.443317165	1.690938074	0.001115301	0.018845168	0.014443	3344	tags=47%, list=27%, signal=35%
BP	GO:2000181	negative regulation of blood vessel morphogenesis	70	0.443317165	1.690938074	0.001115301	0.018845168	0.014443	3344	tags=47%, list=27%, signal=35%
BP	GO:0051098	regulation of binding	240	0.339638111	1.43288982	0.001117049	0.018845168	0.014443	4340	tags=46%, list=34%, signal=31%
BP	GO:1905564	positive regulation of vascular endothelial cell proliferation	11	0.700252028	1.830900573	0.001119072	0.018845168	0.014443	2402	tags=55%, list=19%, signal=44%
BP	GO:0050678	regulation of epithelial cell proliferation	191	0.354923842	1.484356817	0.001137792	0.01910946	0.014646	3060	tags=35%, list=24%, signal=27%
BP	GO:0000184	nuclear-transcribed mRNA catabolic process, nonsense-mediated decay	108	-0.264205975	-1.582453944	0.001153748	0.01932068	0.014808	2003	tags=28%, list=16%, signal=24%
MF	GO:0035198	miRNA binding	14	0.658163349	1.810385253	0.001156487	0.01932068	0.014808	2190	tags=57%, list=17%, signal=47%
BP	GO:2001257	regulation of cation channel activity	63	0.451864609	1.696996735	0.001168858	0.019475819	0.014927	2354	tags=37%, list=19%, signal=30%
BP	GO:0031145	anaphase-promoting complex-dependent catabolic process	62	0.458591918	1.716017351	0.001173863	0.019476509	0.014927	3633	tags=47%, list=29%, signal=33%
MF	GO:0050840	extracellular matrix binding	38	0.522224259	1.821324764	0.001175067	0.019476509	0.014927	1573	tags=42%, list=12%, signal=37%
BP	GO:0048041	focal adhesion assembly	60	0.45564078	1.703530536	0.00118977	0.019523719	0.014964	3274	tags=50%, list=26%, signal=37%
BP	GO:0007188	adenylate cyclase-modulating G protein-coupled receptor signaling pathway	78	0.426741059	1.637731175	0.001194142	0.019523719	0.014964	2747	tags=36%, list=22%, signal=28%
MF	GO:0000295	adenine nucleotide transmembrane transporter activity	22	-0.503840294	-2.041111541	0.00120036	0.019523719	0.014964	799	tags=32%, list=6%, signal=30%
MF	GO:0005346	purine ribonucleotide transmembrane transporter activity	22	-0.503840294	-2.041111541	0.00120036	0.019523719	0.014964	799	tags=32%, list=6%, signal=30%
MF	GO:0015215	nucleotide transmembrane transporter activity	22	-0.503840294	-2.041111541	0.00120036	0.019523719	0.014964	799	tags=32%, list=6%, signal=30%
MF	GO:0015216	purine nucleotide transmembrane transporter activity	22	-0.503840294	-2.041111541	0.00120036	0.019523719	0.014964	799	tags=32%, list=6%, signal=30%
BP	GO:0010833	telomere maintenance via telomere lengthening	57	0.455208426	1.692110339	0.001201309	0.019523719	0.014964	3824	tags=44%, list=30%, signal=31%
BP	GO:0006024	glycosaminoglycan biosynthetic process	67	0.44953197	1.699886323	0.001203016	0.019523719	0.014964	2602	tags=40%, list=21%, signal=32%
BP	GO:0051345	positive regulation of hydrolase activity	446	0.31536052	1.360006167	0.00120574	0.019523719	0.014964	4273	tags=42%, list=34%, signal=29%
CC	GO:0030496	midbody	118	0.390647781	1.570806037	0.001212775	0.019587406	0.015012	4098	tags=47%, list=33%, signal=32%
BP	GO:0034308	primary alcohol metabolic process	49	-0.348418054	-1.830128898	0.001221243	0.019673853	0.015079	2032	tags=43%, list=16%, signal=36%
BP	GO:0030166	proteoglycan biosynthetic process	41	0.496628609	1.756618152	0.001240061	0.019926168	0.015272	2998	tags=49%, list=24%, signal=37%
BP	GO:0060759	regulation of response to cytokine stimulus	117	0.393777226	1.581355869	0.00127308	0.020404815	0.015639	4697	tags=57%, list=37%, signal=36%
MF	GO:0031625	ubiquitin protein ligase binding	222	0.347745737	1.462855463	0.001285794	0.020545782	0.015747	4016	tags=42%, list=32%, signal=29%
CC	GO:0045121	membrane raft	176	0.363465839	1.509353512	0.001288382	0.020545782	0.015747	2677	tags=36%, list=21%, signal=29%
CC	GO:0098794	postsynapse	311	0.331179975	1.410763091	0.001299078	0.02066417	0.015838	3221	tags=35%, list=26%, signal=27%
BP	GO:0030100	regulation of endocytosis	120	0.396890925	1.600574911	0.001305455	0.020713437	0.015875	4076	tags=50%, list=32%, signal=34%
BP	GO:0001937	negative regulation of endothelial cell proliferation	22	0.598067715	1.847590049	0.001322543	0.020884549	0.016007	1884	tags=50%, list=15%, signal=43%
BP	GO:1901522	positive regulation of transcription from RNA polymerase II promoter involved in cellular response to chemical stimulus	17	0.615012906	1.790486563	0.00132442	0.020884549	0.016007	2245	tags=53%, list=18%, signal=44%
BP	GO:0060840	artery development	54	0.464954159	1.717900245	0.001326161	0.020884549	0.016007	2564	tags=46%, list=20%, signal=37%
BP	GO:0015867	ATP transport	20	-0.510288517	-1.999025265	0.00133356	0.020948842	0.016056	1506	tags=30%, list=12%, signal=26%
BP	GO:0006611	protein export from nucleus	136	0.375422881	1.531263277	0.001349425	0.021145449	0.016206	5481	tags=59%, list=43%, signal=34%
BP	GO:0030900	forebrain development	196	0.355655564	1.487929711	0.001359796	0.021255224	0.016291	3507	tags=39%, list=28%, signal=29%
BP	GO:0008154	actin polymerization or depolymerization	127	0.390166266	1.582046934	0.001374981	0.021435882	0.016429	2777	tags=38%, list=22%, signal=30%
CC	GO:0019897	extrinsic component of plasma membrane	82	0.415692085	1.607069285	0.001378142	0.021435882	0.016429	2615	tags=35%, list=21%, signal=28%
CC	GO:0097447	dendritic tree	297	0.33259676	1.414795924	0.001391209	0.021585963	0.016544	4820	tags=51%, list=38%, signal=32%
BP	GO:0042692	muscle cell differentiation	186	0.359250506	1.499374322	0.001399607	0.021663034	0.016603	3799	tags=41%, list=30%, signal=29%
CC	GO:0022627	cytosolic small ribosomal subunit	43	-0.353330437	-1.784500864	0.001403217	0.021665803	0.016605	1823	tags=30%, list=14%, signal=26%

BP	GO:0008015	blood circulation	239	0.337666773	1.424768334	0.001411047	0.021733561	0.016657	2513	tags=31%, list=20%, signal=25%
MF	GO:0051020	GTPase binding	345	0.319443532	1.366849685	0.001446488	0.022225241	0.017034	3486	tags=37%, list=28%, signal=28%
BP	GO:0030838	positive regulation of actin filament polymerization	57	0.45194468	1.679978274	0.001459436	0.022369754	0.017145	3030	tags=44%, list=24%, signal=33%
BP	GO:0006633	fatty acid biosynthetic process	91	-0.2752512	-1.626656137	0.001465272	0.02240483	0.017172	2245	tags=36%, list=18%, signal=30%
BP	GO:0032409	regulation of transporter activity	125	0.384797563	1.555756738	0.001470008	0.022422956	0.017186	2110	tags=30%, list=17%, signal=26%
BP	GO:1900024	regulation of substrate adhesion-dependent cell spreading	35	0.512734497	1.759824329	0.00147868	0.02249505	0.017241	3109	tags=57%, list=25%, signal=43%
BP	GO:0010608	posttranscriptional regulation of gene expression	436	0.311246582	1.341038667	0.001481859	0.02249505	0.017241	4142	tags=41%, list=33%, signal=28%
MF	GO:0033613	activating transcription factor binding	53	0.471137483	1.736493823	0.001502386	0.02274378	0.017431	3213	tags=47%, list=25%, signal=35%
BP	GO:0043968	histone H2A acetylation	14	0.650178757	1.788422334	0.001505447	0.02274378	0.017431	3620	tags=71%, list=29%, signal=51%
BP	GO:0016579	protein deubiquitination	191	0.350051722	1.463980713	0.001526299	0.023003766	0.017631	3754	tags=41%, list=30%, signal=29%
MF	GO:0017016	Ras GTPase binding	266	0.333318052	1.411646156	0.00153862	0.023134253	0.017731	4131	tags=44%, list=33%, signal=30%
MF	GO:0003697	single-stranded DNA binding	71	0.423892175	1.617699894	0.0015469	0.023203507	0.017784	3958	tags=46%, list=31%, signal=32%
BP	GO:0007221	positive regulation of transcription of Notch receptor target	14	0.649970281	1.787848887	0.001553621	0.023249086	0.017819	2329	tags=57%, list=18%, signal=47%
CC	GO:0016363	nuclear matrix	80	0.418598527	1.61062487	0.001561412	0.023299089	0.017857	3848	tags=49%, list=31%, signal=34%
MF	GO:0005201	extracellular matrix structural constituent	91	0.403782791	1.578900718	0.001564341	0.023299089	0.017857	3422	tags=42%, list=27%, signal=31%
BP	GO:0042445	hormone metabolic process	91	-0.272937798	-1.612984591	0.001572812	0.023330438	0.017881	2069	tags=34%, list=16%, signal=29%
BP	GO:0008277	regulation of G protein-coupled receptor signaling pathway	65	0.446480957	1.684686277	0.001576345	0.023330438	0.017881	2104	tags=34%, list=17%, signal=28%
BP	GO:0045785	positive regulation of cell adhesion	244	0.339404606	1.431139966	0.001580214	0.023330438	0.017881	3109	tags=36%, list=25%, signal=28%
BP	GO:0040013	negative regulation of locomotion	191	0.349778804	1.462839319	0.0015818	0.023330438	0.017881	4259	tags=47%, list=34%, signal=31%
BP	GO:0045732	positive regulation of protein catabolic process	153	0.372035342	1.529757741	0.001588178	0.023330438	0.017881	3489	tags=43%, list=28%, signal=32%
BP	GO:0007193	adenylate cyclase-inhibiting G protein-coupled receptor signaling pathway	31	0.554790552	1.859166223	0.001589953	0.023330438	0.017881	2377	tags=48%, list=19%, signal=39%
BP	GO:0022411	cellular component disassembly	384	0.317364787	1.363848792	0.001592307	0.023330438	0.017881	4628	tags=45%, list=37%, signal=29%
CC	GO:0035267	NuA4 histone acetyltransferase complex	15	0.655254089	1.845510871	0.001620315	0.023589674	0.01808	3408	tags=67%, list=27%, signal=49%
CC	GO:0043189	H4/H2A histone acetyltransferase complex	15	0.655254089	1.845510871	0.001620315	0.023589674	0.01808	3408	tags=67%, list=27%, signal=49%
BP	GO:0007266	Rho protein signal transduction	88	0.41454037	1.618446525	0.001621923	0.023589674	0.01808	3848	tags=52%, list=31%, signal=37%
BP	GO:0101023	vascular endothelial cell proliferation	13	0.680867634	1.857473974	0.001628677	0.023589674	0.01808	2402	tags=54%, list=19%, signal=44%
BP	GO:1905562	regulation of vascular endothelial cell proliferation	13	0.680867634	1.857473974	0.001628677	0.023589674	0.01808	2402	tags=54%, list=19%, signal=44%
MF	GO:0016849	phosphorus-oxygen lyase activity	11	0.685910963	1.793403982	0.001647813	0.023812222	0.01825	1686	tags=55%, list=13%, signal=47%
BP	GO:0045603	positive regulation of endothelial cell differentiation	13	0.679309902	1.853224327	0.001700005	0.024470356	0.018755	2676	tags=62%, list=21%, signal=49%
BP	GO:0051090	regulation of DNA-binding transcription factor activity	256	0.333105439	1.406330937	0.001706164	0.024470356	0.018755	4346	tags=45%, list=34%, signal=30%
BP	GO:0010770	positive regulation of cell morphogenesis involved in differentiation	99	0.398535034	1.579526692	0.001710364	0.024470356	0.018755	2800	tags=41%, list=22%, signal=32%
BP	GO:0044409	entry into host	89	0.414228724	1.620344379	0.001712542	0.024470356	0.018755	2496	tags=39%, list=20%, signal=32%
BP	GO:1902624	positive regulation of neutrophil migration	16	0.647205439	1.842285277	0.001712731	0.024470356	0.018755	2387	tags=50%, list=19%, signal=41%
BP	GO:0090092	regulation of transmembrane receptor protein serine/threonine kinase signaling pathway	125	0.38390848	1.552162129	0.001732172	0.024692254	0.018925	3391	tags=42%, list=27%, signal=31%
BP	GO:1905314	semi-lunar valve development	21	0.603087233	1.847957406	0.001737302	0.024709595	0.018938	2346	tags=52%, list=19%, signal=43%
BP	GO:0046847	filopodium assembly	32	0.530899536	1.786731465	0.001796434	0.02549322	0.019539	2692	tags=50%, list=21%, signal=39%
BP	GO:0050657	nucleic acid transport	142	0.369289103	1.510620059	0.001814737	0.025617871	0.019634	5218	tags=56%, list=41%, signal=33%
BP	GO:0050658	RNA transport	142	0.369289103	1.510620059	0.001814737	0.025617871	0.019634	5218	tags=56%, list=41%, signal=33%
BP	GO:0050921	positive regulation of chemotaxis	79	0.426214689	1.637765095	0.001817388	0.025617871	0.019634	2387	tags=35%, list=19%, signal=29%

CC	GO:0000775	chromosome, centromeric region	112	0.385482492	1.548155087	0.001831386	0.025757688	0.019741	6116	tags=70%, list=49%, signal=36%
MF	GO:0008194	UDP-glycosyltransferase activity	63	0.444734368	1.670218811	0.001836763	0.025775912	0.019755	3649	tags=49%, list=29%, signal=35%
BP	GO:0006637	acyl-CoA metabolic process	64	-0.306484232	-1.680233793	0.001855273	0.025906679	0.019856	2122	tags=39%, list=17%, signal=33%
BP	GO:0035383	thioester metabolic process	64	-0.306484232	-1.680233793	0.001855273	0.025906679	0.019856	2122	tags=39%, list=17%, signal=33%
CC	GO:0030863	cortical cytoskeleton	60	0.447195049	1.671953993	0.001858389	0.025906679	0.019856	2883	tags=45%, list=23%, signal=35%
CC	GO:0034399	nuclear periphery	100	0.397829139	1.576757013	0.001865381	0.025946878	0.019886	3943	tags=47%, list=31%, signal=33%
MF	GO:0016620	oxidoreductase activity, acting on the aldehyde or oxo group of donors, NAD or NADP as acceptor	27	-0.442201224	-1.913230423	0.001879215	0.026081854	0.01999	2014	tags=48%, list=16%, signal=41%
MF	GO:0005347	ATP transmembrane transporter activity	19	-0.511018429	-1.994745987	0.001904664	0.026377094	0.020216	1506	tags=32%, list=12%, signal=28%
BP	GO:0006906	vesicle fusion	72	0.426611177	1.630099927	0.001916596	0.026476408	0.020292	4082	tags=56%, list=32%, signal=38%
MF	GO:0008168	methyltransferase activity	102	0.394746488	1.568105267	0.001920221	0.026476408	0.020292	4559	tags=53%, list=36%, signal=34%
MF	GO:0051219	phosphoprotein binding	59	0.449375954	1.674696877	0.001941194	0.02670727	0.020469	4478	tags=56%, list=36%, signal=36%
BP	GO:0044843	cell cycle G1/S phase transition	180	0.352964033	1.468002601	0.001997377	0.027361036	0.02097	4309	tags=47%, list=34%, signal=32%
BP	GO:0050730	regulation of peptidyl-tyrosine phosphorylation	138	0.372586558	1.522960613	0.001997377	0.027361036	0.02097	2484	tags=32%, list=20%, signal=26%
BP	GO:0150116	regulation of cell-substrate junction organization	47	0.487309814	1.769969077	0.002035021	0.027623352	0.021171	3274	tags=55%, list=26%, signal=41%
BP	GO:0030336	negative regulation of cell migration	165	0.361631256	1.49251837	0.002036764	0.027623352	0.021171	4259	tags=48%, list=34%, signal=32%
BP	GO:0009063	cellular amino acid catabolic process	74	-0.285250189	-1.601162813	0.002038815	0.027623352	0.021171	1643	tags=27%, list=13%, signal=24%
BP	GO:0043488	regulation of mRNA stability	137	0.374155644	1.526802671	0.002038821	0.027623352	0.021171	3535	tags=40%, list=28%, signal=29%
BP	GO:0035384	thioester biosynthetic process	37	-0.390499683	-1.90161288	0.002042772	0.027623352	0.021171	2122	tags=49%, list=17%, signal=41%
BP	GO:0071616	acyl-CoA biosynthetic process	37	-0.390499683	-1.90161288	0.002042772	0.027623352	0.021171	2122	tags=49%, list=17%, signal=41%
BP	GO:0022898	regulation of transmembrane transporter activity	119	0.38411457	1.545568426	0.002061729	0.027797576	0.021305	2110	tags=30%, list=17%, signal=25%
CC	GO:0022625	cytosolic large ribosomal subunit	51	-0.337997859	-1.793854084	0.00206446	0.027797576	0.021305	2722	tags=43%, list=22%, signal=34%
BP	GO:0120032	regulation of plasma membrane bounded cell projection assembly	99	0.39411711	1.562016989	0.002108171	0.028219077	0.021628	2733	tags=35%, list=22%, signal=28%
BP	GO:1903670	regulation of sprouting angiogenesis	52	0.470815575	1.725272455	0.002111144	0.028219077	0.021628	3344	tags=54%, list=27%, signal=40%
BP	GO:0006405	RNA export from nucleus	101	0.398276404	1.579674485	0.002112505	0.028219077	0.021628	5436	tags=60%, list=43%, signal=35%
MF	GO:0140097	catalytic activity, acting on DNA	137	0.373677506	1.524851552	0.002113638	0.028219077	0.021628	4657	tags=49%, list=37%, signal=31%
BP	GO:0035313	wound healing, spreading of epidermal cells	11	0.677450095	1.771281934	0.002152974	0.028683615	0.021984	3614	tags=82%, list=29%, signal=58%
MF	GO:0019787	ubiquitin-like protein transferase activity	294	0.327003459	1.390178633	0.002166571	0.028803985	0.022076	4686	tags=48%, list=37%, signal=31%
BP	GO:0050920	regulation of chemotaxis	121	0.382699978	1.542526136	0.002208533	0.029279773	0.022441	2387	tags=32%, list=19%, signal=26%
BP	GO:2001258	negative regulation of cation channel activity	22	0.583065164	1.801243183	0.002211631	0.029279773	0.022441	2354	tags=55%, list=19%, signal=44%
BP	GO:0003170	heart valve development	37	0.505873088	1.75250973	0.00223382	0.029511659	0.022619	2346	tags=46%, list=19%, signal=38%
BP	GO:0030049	muscle filament sliding	11	0.675644537	1.766561067	0.002249196	0.029590982	0.022679	1601	tags=55%, list=13%, signal=48%
BP	GO:0033275	actin-myosin filament sliding	11	0.675644537	1.766561067	0.002249196	0.029590982	0.022679	1601	tags=55%, list=13%, signal=48%
BP	GO:2000279	negative regulation of DNA biosynthetic process	24	0.556193003	1.769091927	0.002287487	0.029892424	0.02291	3746	tags=62%, list=30%, signal=44%
BP	GO:0000723	telomere maintenance	106	0.393452087	1.570945553	0.002291042	0.029892424	0.02291	5591	tags=54%, list=44%, signal=30%
BP	GO:0032200	telomere organization	106	0.393452087	1.570945553	0.002291042	0.029892424	0.02291	5591	tags=54%, list=44%, signal=30%
BP	GO:0050770	regulation of axonogenesis	109	0.396683067	1.588637285	0.002291042	0.029892424	0.02291	2800	tags=40%, list=22%, signal=32%
CC	GO:0000793	condensed chromosome	115	0.38856495	1.560481771	0.002324157	0.030261964	0.023194	4220	tags=45%, list=33%, signal=30%
BP	GO:0060070	canonical Wnt signaling pathway	201	0.33708315	1.412426766	0.002393565	0.031033618	0.023785	4488	tags=46%, list=36%, signal=30%

CC	GO:0030427	site of polarized growth	114	0.389900112	1.565366639	0.002402189	0.031033618	0.023785	2849	tags=41%, list=23%, signal=32%
BP	GO:0032366	intracellular sterol transport	15	-0.565390052	-2.029940256	0.002405079	0.031033618	0.023785	1581	tags=53%, list=13%, signal=47%
BP	GO:0032367	intracellular cholesterol transport	15	-0.565390052	-2.029940256	0.002405079	0.031033618	0.023785	1581	tags=53%, list=13%, signal=47%
BP	GO:0006457	protein folding	154	0.36570428	1.504224839	0.002410472	0.031033618	0.023785	4251	tags=44%, list=34%, signal=30%
MF	GO:0016757	transferase activity, transferring glycosyl groups	142	0.365657459	1.495764395	0.002412907	0.031033618	0.023785	4218	tags=47%, list=33%, signal=32%
BP	GO:0010743	regulation of macrophage derived foam cell differentiation	19	0.600869129	1.794583688	0.00243415	0.031203195	0.023915	1973	tags=47%, list=16%, signal=40%
BP	GO:0006458	'de novo' protein folding	30	0.529937415	1.752433479	0.002435974	0.031203195	0.023915	2493	tags=43%, list=20%, signal=35%
BP	GO:0045786	negative regulation of cell cycle	391	0.313686853	1.348693321	0.002460386	0.031415515	0.024078	4814	tags=47%, list=38%, signal=30%
MF	GO:0044389	ubiquitin-like protein ligase binding	233	0.336831939	1.41823058	0.002470258	0.031415515	0.024078	4077	tags=42%, list=32%, signal=29%
MF	GO:0045296	cadherin binding	233	0.33677156	1.417976357	0.002470258	0.031415515	0.024078	4089	tags=44%, list=32%, signal=30%
BP	GO:0052372	modulation by symbiont of entry into host	27	0.541891773	1.76371144	0.002476205	0.031415515	0.024078	2366	tags=52%, list=19%, signal=42%
BP	GO:0007004	telomere maintenance via telomerase	51	0.471134684	1.725417074	0.002478427	0.031415515	0.024078	3824	tags=45%, list=30%, signal=32%
MF	GO:0009975	cyclase activity	10	0.717975991	1.812901178	0.002482398	0.031415515	0.024078	1686	tags=60%, list=13%, signal=52%
CC	GO:0071011	precatalytic spliceosome	41	0.483137322	1.708898306	0.002492369	0.031478618	0.024126	5336	tags=66%, list=42%, signal=38%
CC	GO:0000151	ubiquitin ligase complex	192	0.344034345	1.43989283	0.002584288	0.032574407	0.024966	4234	tags=45%, list=34%, signal=31%
BP	GO:0006270	DNA replication initiation	24	0.55236207	1.756906817	0.002592476	0.032612528	0.024995	5125	tags=83%, list=41%, signal=50%
BP	GO:0034470	ncRNA processing	281	0.322564301	1.36873817	0.002613599	0.032812877	0.025149	5392	tags=53%, list=43%, signal=31%
BP	GO:0001959	regulation of cytokine-mediated signaling pathway	110	0.389210842	1.560916907	0.002623803	0.032875626	0.025197	4697	tags=56%, list=37%, signal=36%
BP	GO:0051220	cytoplasmic sequestering of protein	18	0.614021826	1.818749979	0.002629057	0.032876226	0.025197	2078	tags=50%, list=16%, signal=42%
BP	GO:0044764	multi-organism cellular process	10	0.713744031	1.802215409	0.00265603	0.033147882	0.025406	595	tags=50%, list=5%, signal=48%
CC	GO:0001772	immunological synapse	25	-0.439527233	-1.847546131	0.002680611	0.033212891	0.025455	2706	tags=48%, list=21%, signal=38%
CC	GO:0000123	histone acetyltransferase complex	55	0.451657509	1.678865818	0.002682276	0.033212891	0.025455	4346	tags=55%, list=34%, signal=36%
CC	GO:0031248	protein acetyltransferase complex	55	0.451657509	1.678865818	0.002682276	0.033212891	0.025455	4346	tags=55%, list=34%, signal=36%
CC	GO:1902493	acetyltransferase complex	55	0.451657509	1.678865818	0.002682276	0.033212891	0.025455	4346	tags=55%, list=34%, signal=36%
BP	GO:0007186	G protein-coupled receptor signaling pathway	337	0.318727857	1.362868362	0.002722089	0.033639909	0.025783	2377	tags=27%, list=19%, signal=23%
BP	GO:0051893	regulation of focal adhesion assembly	44	0.479432894	1.720356544	0.002756209	0.03386471	0.025955	3274	tags=55%, list=26%, signal=41%
BP	GO:0090109	regulation of cell-substrate junction assembly	44	0.479432894	1.720356544	0.002756209	0.03386471	0.025955	3274	tags=55%, list=26%, signal=41%
BP	GO:0098609	cell-cell adhesion	420	0.308919415	1.329391613	0.002756368	0.03386471	0.025955	2692	tags=31%, list=21%, signal=26%
BP	GO:0050690	regulation of defense response to virus by virus	22	0.575400283	1.77756433	0.002836843	0.034730944	0.026619	1469	tags=41%, list=12%, signal=36%
BP	GO:0007156	homophilic cell adhesion via plasma membrane adhesion molecules	46	0.468493024	1.694033353	0.002837873	0.034730944	0.026619	1114	tags=30%, list=9%, signal=28%
MF	GO:0031490	chromatin DNA binding	57	0.44104096	1.639446738	0.00285347	0.034854278	0.026713	3746	tags=51%, list=30%, signal=36%
BP	GO:0035023	regulation of Rho protein signal transduction	53	0.457163068	1.684987656	0.002859633	0.034862126	0.026719	1973	tags=40%, list=16%, signal=34%
BP	GO:0002474	antigen processing and presentation of peptide antigen via MHC class I	86	0.408004083	1.589987518	0.002871097	0.034934443	0.026775	4595	tags=56%, list=36%, signal=36%
BP	GO:0051271	negative regulation of cellular component movement	194	0.346636071	1.45020505	0.002881755	0.034996697	0.026822	4259	tags=47%, list=34%, signal=32%
MF	GO:0005509	calcium ion binding	285	0.323402683	1.372380273	0.002910173	0.035273976	0.027035	4076	tags=42%, list=32%, signal=29%
BP	GO:0006959	humoral immune response	78	-0.274503287	-1.562404099	0.002933276	0.035450782	0.027171	1501	tags=31%, list=12%, signal=27%
BP	GO:1905039	carboxylic acid transmembrane transport	64	-0.297287674	-1.629815645	0.002935987	0.035450782	0.027171	1021	tags=23%, list=8%, signal=22%
BP	GO:0006865	amino acid transport	69	-0.293707803	-1.63622764	0.002963012	0.035640798	0.027316	1021	tags=23%, list=8%, signal=21%
BP	GO:1902600	proton transmembrane transport	77	-0.277864912	-1.590087333	0.002963012	0.035640798	0.027316	2686	tags=40%, list=21%, signal=32%

CC	GO:0032154	cleavage furrow	32	0.520523967	1.751812698	0.002977178	0.035743119	0.027395	4239	tags=62%, list=34%, signal=42%
CC	GO:0042383	sarcolemma	73	0.417733132	1.597690932	0.002985451	0.035774431	0.027419	1697	tags=32%, list=13%, signal=27%
BP	GO:0000280	nuclear division	204	0.342287624	1.435305576	0.003064595	0.036653256	0.028092	4694	tags=45%, list=37%, signal=29%
CC	GO:0032153	cell division site	38	0.49995911	1.743672173	0.003072336	0.036676368	0.02811	4239	tags=61%, list=34%, signal=40%
BP	GO:0038093	Fc receptor signaling pathway	143	0.368246086	1.506892281	0.003089385	0.036734702	0.028155	2777	tags=34%, list=22%, signal=27%
BP	GO:0046822	regulation of nucleocytoplasmic transport	81	0.412783516	1.589966766	0.003096001	0.036734702	0.028155	4754	tags=56%, list=38%, signal=35%
BP	GO:0060491	regulation of cell projection assembly	100	0.390476962	1.547617376	0.003102124	0.036734702	0.028155	2733	tags=35%, list=22%, signal=28%
BP	GO:0048010	vascular endothelial growth factor receptor signaling pathway	69	0.426923995	1.624897815	0.003105799	0.036734702	0.028155	2253	tags=39%, list=18%, signal=32%
MF	GO:0070491	repressing transcription factor binding	54	0.447796548	1.654506761	0.003106307	0.036734702	0.028155	5057	tags=59%, list=40%, signal=36%
BP	GO:0051017	actin filament bundle assembly	109	0.392032398	1.570012276	0.003124132	0.036807642	0.02821	4077	tags=51%, list=32%, signal=35%
BP	GO:0061572	actin filament bundle organization	109	0.392032398	1.570012276	0.003124132	0.036807642	0.02821	4077	tags=51%, list=32%, signal=35%
MF	GO:0042826	histone deacetylase binding	85	0.40541078	1.574864498	0.003160593	0.037167873	0.028487	5558	tags=71%, list=44%, signal=40%
MF	GO:0004842	ubiquitin-protein transferase activity	277	0.327061237	1.386971176	0.003169675	0.03720539	0.028515	4686	tags=48%, list=37%, signal=31%
CC	GO:0005884	actin filament	73	0.41586704	1.590553748	0.003180576	0.037264078	0.02856	5634	tags=64%, list=45%, signal=36%
BP	GO:0006260	DNA replication	166	0.354214127	1.463524329	0.003188827	0.037291565	0.028581	5192	tags=53%, list=41%, signal=32%
CC	GO:0098797	plasma membrane protein complex	251	0.330587822	1.395197299	0.00320996	0.037443427	0.028698	4478	tags=46%, list=36%, signal=30%
CC	GO:0000812	Swr1 complex	11	0.664527931	1.737495245	0.003215205	0.037443427	0.028698	3016	tags=64%, list=24%, signal=48%
BP	GO:0048524	positive regulation of viral process	78	0.410104376	1.573883524	0.003219601	0.037443427	0.028698	5275	tags=67%, list=42%, signal=39%
BP	GO:0000226	microtubule cytoskeleton organization	302	0.322476475	1.37366558	0.003240575	0.037618073	0.028832	4303	tags=42%, list=34%, signal=28%
BP	GO:0003148	outflow tract septum morphogenesis	13	0.660183277	1.80104501	0.00327344	0.037929861	0.029071	2346	tags=69%, list=19%, signal=56%
CC	GO:0000781	chromosome, telomeric region	88	0.402352926	1.570864362	0.003298443	0.038149579	0.029239	5591	tags=59%, list=44%, signal=33%
MF	GO:0042393	histone binding	145	0.36692502	1.50327758	0.003310717	0.038221532	0.029294	5089	tags=53%, list=40%, signal=32%
BP	GO:0035633	maintenance of blood-brain barrier	25	0.550799887	1.75993954	0.003384703	0.038908599	0.029821	1291	tags=44%, list=10%, signal=40%
BP	GO:0051236	establishment of RNA localization	144	0.370130145	1.516539548	0.003385535	0.038908599	0.029821	5218	tags=56%, list=41%, signal=33%
BP	GO:0002009	morphogenesis of an epithelium	324	0.325429013	1.387957687	0.003388714	0.038908599	0.029821	4458	tags=47%, list=35%, signal=31%
BP	GO:0033559	unsaturated fatty acid metabolic process	60	-0.295828796	-1.605920439	0.003404298	0.039016588	0.029903	2220	tags=37%, list=18%, signal=30%
BP	GO:0006310	DNA recombination	138	0.365293018	1.493148012	0.003453376	0.039507371	0.03028	5591	tags=59%, list=44%, signal=33%
MF	GO:0030170	pyridoxal phosphate binding	25	-0.428676422	-1.801934911	0.003482679	0.039678613	0.030411	1607	tags=44%, list=13%, signal=38%
MF	GO:0070279	vitamin B6 binding	25	-0.428676422	-1.801934911	0.003482679	0.039678613	0.030411	1607	tags=44%, list=13%, signal=38%
BP	GO:0000082	G1/S transition of mitotic cell cycle	168	0.35196727	1.455531448	0.003487194	0.039678613	0.030411	4309	tags=47%, list=34%, signal=31%
BP	GO:0009147	pyrimidine nucleoside triphosphate metabolic process	15	0.630012761	1.774419145	0.003494338	0.039688389	0.030418	4069	tags=80%, list=32%, signal=54%
CC	GO:0098793	presynapse	235	0.326552725	1.376878855	0.003540394	0.040002219	0.030659	4774	tags=51%, list=38%, signal=32%
BP	GO:0030177	positive regulation of Wnt signaling pathway	116	0.373257445	1.498008158	0.003540679	0.040002219	0.030659	3530	tags=40%, list=28%, signal=29%
BP	GO:0006244	pyrimidine nucleotide catabolic process	11	0.658806106	1.722534787	0.003553641	0.040002219	0.030659	1887	tags=55%, list=15%, signal=46%
BP	GO:0009223	pyrimidine deoxyribonucleotide catabolic process	11	0.658806106	1.722534787	0.003553641	0.040002219	0.030659	1887	tags=55%, list=15%, signal=46%
BP	GO:0030207	chondroitin sulfate catabolic process	11	0.658435403	1.721565535	0.003553641	0.040002219	0.030659	2695	tags=55%, list=21%, signal=43%
BP	GO:0006352	DNA-templated transcription, initiation	159	0.357153781	1.472408704	0.003599082	0.040328274	0.030909	4676	tags=51%, list=37%, signal=32%
BP	GO:0007596	blood coagulation	177	0.348948819	1.449963653	0.003599082	0.040328274	0.030909	4054	tags=44%, list=32%, signal=30%
MF	GO:0003688	DNA replication origin binding	13	0.658653706	1.796872191	0.003607947	0.040328274	0.030909	2999	tags=77%, list=24%, signal=59%

BP	GO:0002831	regulation of response to biotic stimulus	253	0.33029451	1.394085454	0.003614537	0.040328274	0.030909	3801	tags=39%, list=30%, signal=28%
BP	GO:0051656	establishment of organelle localization	268	0.325766224	1.378420107	0.003614537	0.040328274	0.030909	4712	tags=46%, list=37%, signal=29%
BP	GO:0099024	plasma membrane invagination	35	0.489666706	1.680650294	0.003637009	0.040507429	0.031046	4181	tags=54%, list=33%, signal=36%
BP	GO:0071732	cellular response to nitric oxide	11	0.656173854	1.715652419	0.003650338	0.040527335	0.031061	3654	tags=73%, list=29%, signal=52%
BP	GO:0061097	regulation of protein tyrosine kinase activity	57	0.437176027	1.62507993	0.003651632	0.040527335	0.031061	2484	tags=35%, list=20%, signal=28%
CC	GO:0030118	clathrin coat	30	0.52313079	1.729924863	0.00368627	0.040768464	0.031246	2272	tags=50%, list=18%, signal=41%
BP	GO:0060976	coronary vasculature development	30	0.523058343	1.729685289	0.00368627	0.040768464	0.031246	3398	tags=60%, list=27%, signal=44%
CC	GO:0044309	neuron spine	85	0.40294261	1.565276611	0.003703511	0.040816183	0.031283	3011	tags=42%, list=24%, signal=32%
BP	GO:0051147	regulation of muscle cell differentiation	85	0.402947194	1.565294418	0.003703511	0.040816183	0.031283	3408	tags=44%, list=27%, signal=32%
CC	GO:0000776	kinetochore	78	0.406079693	1.558437743	0.003726926	0.041002684	0.031426	6010	tags=72%, list=48%, signal=38%
BP	GO:0003015	heart process	121	0.376093855	1.515899227	0.003756363	0.041207691	0.031583	2387	tags=32%, list=19%, signal=26%
CC	GO:0000784	nuclear chromosome, telomeric region	65	0.432571044	1.632200633	0.003758611	0.041207691	0.031583	5430	tags=65%, list=43%, signal=37%
BP	GO:0051701	interaction with host	134	0.368935269	1.501213723	0.003767234	0.041230645	0.0316	4082	tags=47%, list=32%, signal=32%
BP	GO:0007187	G protein-coupled receptor signaling pathway, coupled to cyclic nucleotide second messenger	82	0.397919827	1.53836158	0.003815855	0.041668331	0.031936	2747	tags=34%, list=22%, signal=27%
BP	GO:0048844	artery morphogenesis	37	0.493079104	1.708187188	0.003820422	0.041668331	0.031936	2468	tags=49%, list=20%, signal=39%
BP	GO:0044057	regulation of system process	253	0.329535086	1.390880126	0.003837334	0.041780624	0.032022	2727	tags=32%, list=22%, signal=26%
CC	GO:0030119	AP-type membrane coat adaptor complex	26	0.539446883	1.746451591	0.003920093	0.042608238	0.032656	2272	tags=46%, list=18%, signal=38%
MF	GO:0017124	SH3 domain binding	81	0.407702425	1.57039533	0.003952746	0.042889336	0.032872	4719	tags=63%, list=37%, signal=40%
BP	GO:0071825	protein-lipid complex subunit organization	24	0.544060244	1.73050107	0.003986722	0.043014712	0.032968	1973	tags=42%, list=16%, signal=35%
BP	GO:0006261	DNA-dependent DNA replication	88	0.39886042	1.557228936	0.003993225	0.043014712	0.032968	5192	tags=62%, list=41%, signal=37%
BP	GO:0003012	muscle system process	213	0.338527497	1.4212564	0.003994348	0.043014712	0.032968	2768	tags=33%, list=22%, signal=26%
BP	GO:0043535	regulation of blood vessel endothelial cell migration	66	0.422578975	1.596757944	0.003994752	0.043014712	0.032968	3371	tags=44%, list=27%, signal=32%
BP	GO:0045666	positive regulation of neuron differentiation	203	0.33748235	1.41362711	0.003998359	0.043014712	0.032968	3474	tags=38%, list=28%, signal=28%
CC	GO:0014704	intercalated disc	28	0.52390791	1.7203688	0.004041361	0.04340339	0.033266	1697	tags=39%, list=13%, signal=34%
BP	GO:0050673	epithelial cell proliferation	224	0.335557772	1.411877747	0.004077186	0.043504122	0.033343	3216	tags=35%, list=26%, signal=27%
BP	GO:0099003	vesicle-mediated transport in synapse	104	0.381632328	1.51912789	0.004080292	0.043504122	0.033343	5168	tags=55%, list=41%, signal=33%
BP	GO:0006278	RNA-dependent DNA biosynthetic process	52	0.45924955	1.682889526	0.0040815	0.043504122	0.033343	3824	tags=44%, list=30%, signal=31%
BP	GO:0050679	positive regulation of epithelial cell proliferation	107	0.376803664	1.502966865	0.004088671	0.043504122	0.033343	3060	tags=36%, list=24%, signal=27%
BP	GO:0007599	hemostasis	182	0.348227274	1.449231936	0.004089516	0.043504122	0.033343	4054	tags=43%, list=32%, signal=30%
CC	GO:0043197	dendritic spine	84	0.405252015	1.572275685	0.004092074	0.043504122	0.033343	3011	tags=43%, list=24%, signal=33%
BP	GO:0032481	positive regulation of type I interferon production	59	0.433135952	1.61417499	0.004179605	0.044360011	0.033999	4516	tags=53%, list=36%, signal=34%
BP	GO:0010596	negative regulation of endothelial cell migration	35	0.485229315	1.665420132	0.004218925	0.044702197	0.034261	4447	tags=60%, list=35%, signal=39%
BP	GO:0003333	amino acid transmembrane transport	39	-0.359130195	-1.820250857	0.004234417	0.044718628	0.034274	1021	tags=31%, list=8%, signal=28%
BP	GO:0016339	calcium-dependent cell-cell adhesion via plasma membrane cell adhesion molecules	12	0.665892534	1.783376278	0.004234638	0.044718628	0.034274	1794	tags=50%, list=14%, signal=43%
CC	GO:0071005	U2-type precatalytic spliceosome	40	0.480763599	1.688594519	0.004283236	0.045156321	0.034609	5336	tags=65%, list=42%, signal=38%
BP	GO:0090174	organelle membrane fusion	76	0.417069378	1.596687655	0.004297458	0.045169449	0.034619	4082	tags=54%, list=32%, signal=37%
BP	GO:0050817	coagulation	178	0.345782901	1.436702263	0.00430594	0.045169449	0.034619	4054	tags=44%, list=32%, signal=30%
BP	GO:0060271	cilium assembly	171	0.348807011	1.444704586	0.00430594	0.045169449	0.034619	4138	tags=41%, list=33%, signal=28%
BP	GO:0097006	regulation of plasma lipoprotein particle levels	45	0.474311853	1.706698153	0.004313389	0.045172561	0.034622	1973	tags=38%, list=16%, signal=32%

BP	GO:0000381	regulation of alternative mRNA splicing, via spliceosome	38	0.493415844	1.720851684	0.004326745	0.045237411	0.034671	5218	tags=71%, list=41%, signal=42%
BP	GO:0010742	macrophage derived foam cell differentiation	21	0.57719482	1.76861884	0.004347796	0.045273747	0.034699	2037	tags=48%, list=16%, signal=40%
BP	GO:0090077	foam cell differentiation	21	0.57719482	1.76861884	0.004347796	0.045273747	0.034699	2037	tags=48%, list=16%, signal=40%
BP	GO:0006577	amino-acid betaine metabolic process	10	-0.640245438	-1.942745249	0.004354745	0.045273747	0.034699	3086	tags=70%, list=24%, signal=53%
MF	GO:0031267	small GTPase binding	274	0.324123709	1.37366484	0.004358898	0.045273747	0.034699	4131	tags=43%, list=33%, signal=30%
MF	GO:0061980	regulatory RNA binding	20	0.564374415	1.710900775	0.004408941	0.04571833	0.03504	2190	tags=50%, list=17%, signal=41%
BP	GO:0060546	negative regulation of necroptotic process	11	0.650015229	1.699549889	0.004423909	0.045723378	0.035044	2677	tags=64%, list=21%, signal=50%
BP	GO:0062099	negative regulation of programmed necrotic cell death	11	0.650015229	1.699549889	0.004423909	0.045723378	0.035044	2677	tags=64%, list=21%, signal=50%
BP	GO:0035456	response to interferon-beta	23	0.546548005	1.709628333	0.004450474	0.045828363	0.035124	2312	tags=48%, list=18%, signal=39%
BP	GO:2000146	negative regulation of cell motility	174	0.35242913	1.461842029	0.004455708	0.045828363	0.035124	4259	tags=47%, list=34%, signal=32%
BP	GO:0048714	positive regulation of oligodendrocyte differentiation	11	0.64887802	1.696576507	0.004472257	0.045828363	0.035124	3009	tags=73%, list=24%, signal=55%
BP	GO:2001212	regulation of vasculogenesis	11	0.648145465	1.694661145	0.004472257	0.045828363	0.035124	2760	tags=64%, list=22%, signal=50%
MF	GO:0070064	proline-rich region binding	14	0.621336645	1.709087417	0.004478103	0.045828363	0.035124	1522	tags=43%, list=12%, signal=38%
BP	GO:0042339	keratan sulfate metabolic process	23	0.545234139	1.705518496	0.004495202	0.045828363	0.035124	3163	tags=48%, list=25%, signal=36%
BP	GO:0008284	positive regulation of cell population proliferation	473	0.299001917	1.291491687	0.004498909	0.045828363	0.035124	3883	tags=38%, list=31%, signal=27%
CC	GO:0048471	perinuclear region of cytoplasm	441	0.301354734	1.299663932	0.004498909	0.045828363	0.035124	3479	tags=35%, list=28%, signal=26%
MF	GO:0019894	kinesin binding	20	0.56236796	1.70481821	0.00449938	0.045828363	0.035124	3144	tags=60%, list=25%, signal=45%
BP	GO:0050654	chondroitin sulfate proteoglycan metabolic process	28	0.520918238	1.710551543	0.004512622	0.045889226	0.035171	3752	tags=57%, list=30%, signal=40%
BP	GO:0009083	branched-chain amino acid catabolic process	20	-0.473910062	-1.856514807	0.004533908	0.04603156	0.03528	2885	tags=55%, list=23%, signal=42%
BP	GO:1904356	regulation of telomere maintenance via telomere lengthening	43	0.471863905	1.693627599	0.004544813	0.046060923	0.035302	3746	tags=47%, list=30%, signal=33%
BP	GO:1904063	negative regulation of cation transmembrane transport	44	0.469833554	1.685911082	0.004554834	0.046060923	0.035302	1484	tags=36%, list=12%, signal=32%
BP	GO:0009148	pyrimidine nucleoside triphosphate biosynthetic process	12	0.663739086	1.77760897	0.004569578	0.046060923	0.035302	4069	tags=83%, list=32%, signal=56%
BP	GO:0060547	negative regulation of necrotic cell death	12	0.664393245	1.77936092	0.004569578	0.046060923	0.035302	2677	tags=67%, list=21%, signal=53%
BP	GO:0009792	embryo development ending in birth or egg hatching	379	0.307887937	1.321708762	0.00457327	0.046060923	0.035302	4382	tags=44%, list=35%, signal=30%
BP	GO:0050900	leukocyte migration	228	0.331162145	1.393680343	0.004591618	0.046171765	0.035387	2402	tags=29%, list=19%, signal=24%
BP	GO:0030216	keratinocyte differentiation	65	-0.303388906	-1.6600827	0.004598898	0.046171765	0.035387	1613	tags=34%, list=13%, signal=30%
BP	GO:0060411	cardiac septum morphogenesis	42	0.477555175	1.695507621	0.004610753	0.046178302	0.035392	2346	tags=45%, list=19%, signal=37%
BP	GO:0032365	intracellular lipid transport	24	-0.434065618	-1.760883309	0.004614174	0.046178302	0.035392	1581	tags=38%, list=13%, signal=33%
BP	GO:0001701	in utero embryonic development	235	0.3237983	1.36526508	0.004656935	0.046532506	0.035664	4360	tags=46%, list=35%, signal=30%
CC	GO:0036379	myofilament	11	0.646096236	1.689303168	0.00466565	0.04654594	0.035674	2489	tags=64%, list=20%, signal=51%
BP	GO:1901991	negative regulation of mitotic cell cycle phase transition	164	0.351468352	1.450202492	0.004689796	0.046713034	0.035802	4814	tags=49%, list=38%, signal=31%
BP	GO:0090100	positive regulation of transmembrane receptor protein serine/threonine kinase signaling pathway	57	0.433857368	1.612743739	0.004772601	0.04746295	0.036377	3074	tags=46%, list=24%, signal=35%
BP	GO:0034314	Arp2/3 complex-mediated actin nucleation	27	0.526298792	1.712960498	0.004803761	0.047697723	0.036557	3030	tags=59%, list=24%, signal=45%
BP	GO:0019932	second-messenger-mediated signaling	191	0.339456425	1.419669231	0.004820429	0.047718965	0.036573	2747	tags=32%, list=22%, signal=26%
BP	GO:0007369	gastrulation	95	0.390518735	1.537385408	0.004821013	0.047718965	0.036573	4265	tags=51%, list=34%, signal=34%
MF	GO:0046982	protein heterodimerization activity	137	0.361173787	1.473828104	0.004849644	0.047927234	0.036733	5300	tags=61%, list=42%, signal=36%
BP	GO:0070925	organelle assembly	488	0.299136	1.294010897	0.004870712	0.048060227	0.036835	4470	tags=42%, list=35%, signal=28%
CC	GO:0062023	collagen-containing extracellular matrix	209	0.337133204	1.413307627	0.004895163	0.04822614	0.036962	3422	tags=37%, list=27%, signal=28%
CC	GO:0031256	leading edge membrane	98	0.390337659	1.544641016	0.004924254	0.048437172	0.037124	2128	tags=31%, list=17%, signal=26%

CC	GO:0030864	cortical actin cytoskeleton	47	0.468518631	1.701717194	0.0049488	0.048602914	0.037251	2727	tags=47%, list=22%, signal=37%
BP	GO:2000811	negative regulation of anoikis	15	0.620016728	1.746265506	0.005016599	0.049192274	0.037702	1606	tags=53%, list=13%, signal=47%
BP	GO:0031032	actomyosin structure organization	126	0.36880794	1.493276367	0.005030506	0.04925216	0.037748	2564	tags=36%, list=20%, signal=29%
BP	GO:0034199	activation of protein kinase A activity	12	0.660402238	1.768672308	0.005049006	0.049356772	0.037828	1686	tags=50%, list=13%, signal=43%
BP	GO:0070198	protein localization to chromosome, telomeric region	22	0.558002233	1.723817132	0.005073184	0.049516468	0.037951	2690	tags=45%, list=21%, signal=36%
BP	GO:0006302	double-strand break repair	131	0.369242433	1.500601979	0.005090631	0.049610082	0.038023	5432	tags=55%, list=43%, signal=32%
BP	GO:0061298	retina vasculature development in camera-type eye	15	0.618398519	1.741707848	0.005112151	0.049743038	0.038125	895	tags=47%, list=7%, signal=43%
BP	GO:0003198	epithelial to mesenchymal transition involved in endocardial cushion formation	10	0.692588188	1.748796558	0.005138735	0.04981039	0.038176	2307	tags=70%, list=18%, signal=57%
BP	GO:0060485	mesenchyme development	153	0.354847753	1.459084759	0.005140008	0.04981039	0.038176	4265	tags=49%, list=34%, signal=33%
BP	GO:0051924	regulation of calcium ion transport	117	0.372433496	1.49564235	0.005142735	0.04981039	0.038176	2548	tags=31%, list=20%, signal=25%
BP	GO:0072583	clathrin-dependent endocytosis	30	0.516289221	1.707300691	0.005155964	0.049862045	0.038216	4040	tags=63%, list=32%, signal=43%
BP	GO:0010948	negative regulation of cell cycle process	227	0.331632307	1.395931481	0.005188889	0.049980813	0.038307	4641	tags=45%, list=37%, signal=29%
BP	GO:0010324	membrane invagination	43	0.468931207	1.683101475	0.005199904	0.049980813	0.038307	4181	tags=51%, list=33%, signal=34%
BP	GO:0050000	chromosome localization	43	0.468568568	1.681799878	0.005199904	0.049980813	0.038307	3908	tags=51%, list=31%, signal=35%
BP	GO:0051303	establishment of chromosome localization	43	0.468568568	1.681799878	0.005199904	0.049980813	0.038307	3908	tags=51%, list=31%, signal=35%

KEGG

ID	Description	setSize	enrichmentScore	NES	pvalue	p.adjust	qvalues	rank	leading_edge
hsa00280	Valine, leucine and isoleucine degradation	38	-0.600073502	-2.860203076	1.16982E-08	3.67322E-06	2.4E-06	3088	tags=68%, list=24%, signal=52%
hsa05200	Pathways in cancer	316	0.389029385	1.653566937	1.10655E-07	1.73729E-05	1.14E-05	3766	tags=44%, list=30%, signal=31%
hsa05132	Salmonella infection	187	0.414618844	1.719772194	3.79024E-06	0.000301276	0.000197	4286	tags=54%, list=34%, signal=36%
hsa00640	Propanoate metabolism	28	-0.592574674	-2.583326873	3.83791E-06	0.000301276	0.000197	3338	tags=68%, list=26%, signal=50%
hsa04151	PI3K-Akt signaling pathway	200	0.402199447	1.670265217	6.09746E-06	0.000317376	0.000207	3507	tags=44%, list=28%, signal=33%
hsa03040	Spliceosome	102	0.473522867	1.867423848	6.99145E-06	0.000317376	0.000207	5336	tags=69%, list=42%, signal=40%
hsa05135	Yersinia infection	99	0.468150929	1.840240414	7.41471E-06	0.000317376	0.000207	3521	tags=53%, list=28%, signal=38%
hsa04510	Focal adhesion	136	0.431946839	1.751478862	8.08601E-06	0.000317376	0.000207	3460	tags=46%, list=27%, signal=34%
hsa00330	Arginine and proline metabolism	30	-0.533059929	-2.331005338	1.49111E-05	0.000520232	0.00034	1743	tags=50%, list=14%, signal=43%
hsa04144	Endocytosis	175	0.403082369	1.662448893	2.03678E-05	0.00063955	0.000418	2942	tags=45%, list=23%, signal=35%
hsa05100	Bacterial invasion of epithelial cells	53	0.536520518	1.971129401	2.2742E-05	0.000649181	0.000424	2777	tags=57%, list=22%, signal=44%
hsa05222	Small cell lung cancer	70	0.49813663	1.895081741	2.86051E-05	0.000748501	0.000489	3521	tags=53%, list=28%, signal=38%
hsa04810	Regulation of actin cytoskeleton	142	0.406861345	1.651606506	6.92163E-05	0.001671839	0.001093	2777	tags=40%, list=22%, signal=32%
hsa05203	Viral carcinogenesis	127	0.420091175	1.691063699	9.2472E-05	0.002074015	0.001356	4647	tags=56%, list=37%, signal=36%
hsa04670	Leukocyte transendothelial migration	70	0.477052302	1.814869763	0.000143201	0.002887697	0.001888	2617	tags=46%, list=21%, signal=36%
hsa05160	Hepatitis C	106	0.432651893	1.711396833	0.000147144	0.002887697	0.001888	4516	tags=59%, list=36%, signal=38%
hsa01200	Carbon metabolism	84	-0.300759113	-1.67833301	0.000189785	0.00343877	0.002248	1659	tags=35%, list=13%, signal=30%
hsa00650	Butanoate metabolism	12	-0.733984241	-2.44364964	0.000198078	0.00343877	0.002248	3053	tags=83%, list=24%, signal=63%
hsa05165	Human papillomavirus infection	216	0.372018007	1.551453103	0.000208078	0.00343877	0.002248	4516	tags=53%, list=36%, signal=34%
hsa05130	Pathogenic Escherichia coli infection	133	0.403167368	1.628444366	0.000238254	0.003740593	0.002445	2777	tags=41%, list=22%, signal=33%
hsa04666	Fc gamma R-mediated phagocytosis	73	0.45810198	1.752357057	0.000299469	0.00447777	0.002927	3628	tags=53%, list=29%, signal=38%
hsa05166	Human T-cell leukemia virus 1 infection	153	0.388741675	1.579878	0.000372414	0.005266423	0.003443	3654	tags=41%, list=29%, signal=30%

hsa00071	Fatty acid degradation	30	-0.459487297	-2.00928129	0.000385757	0.005266423	0.003443	3233	tags=70%, list=26%, signal=52%
hsa04926	Relaxin signaling pathway	75	0.442227434	1.69173116	0.000443677	0.005630035	0.00368	3460	tags=43%, list=27%, signal=31%
hsa04015	Rap1 signaling pathway	126	0.400102786	1.6097195	0.000448251	0.005630035	0.00368	3460	tags=43%, list=27%, signal=31%
hsa05220	Chronic myeloid leukemia	56	0.459889075	1.698537017	0.000791025	0.009473679	0.006193	3460	tags=46%, list=27%, signal=34%
hsa04512	ECM-receptor interaction	51	0.477447823	1.749134821	0.000832098	0.009473679	0.006193	2736	tags=45%, list=22%, signal=35%
hsa05131	Shigellosis	167	0.378542451	1.552238936	0.000844787	0.009473679	0.006193	4223	tags=50%, list=33%, signal=33%
hsa05164	Influenza A	107	0.411750203	1.628738848	0.000890144	0.009483675	0.0062	4516	tags=52%, list=36%, signal=34%
hsa00520	Amino sugar and nucleotide sugar metabolism	36	-0.409989704	-1.91199578	0.000906084	0.009483675	0.0062	1938	tags=33%, list=15%, signal=28%
hsa01212	Fatty acid metabolism	38	-0.389997544	-1.858892575	0.001056213	0.0102001	0.006668	3088	tags=63%, list=24%, signal=48%
hsa04072	Phospholipase D signaling pathway	89	0.418170611	1.624214064	0.001070873	0.0102001	0.006668	3628	tags=47%, list=29%, signal=34%
hsa05414	Dilated cardiomyopathy	46	0.494705612	1.765930417	0.001102719	0.0102001	0.006668	2696	tags=46%, list=21%, signal=36%
hsa05206	MicroRNAs in cancer	116	0.390653154	1.56139078	0.001104469	0.0102001	0.006668	2963	tags=41%, list=24%, signal=32%
hsa05170	Human immunodeficiency virus 1 infection	128	0.388291589	1.563291959	0.001247833	0.011194848	0.007318	4832	tags=57%, list=38%, signal=36%
hsa05169	Epstein-Barr virus infection	144	0.384885577	1.566181405	0.001293587	0.011282955	0.007376	4595	tags=51%, list=36%, signal=33%
hsa05163	Human cytomegalovirus infection	146	0.380771372	1.546494908	0.001426757	0.011919869	0.007792	3531	tags=42%, list=28%, signal=31%
hsa05167	Kaposi sarcoma-associated herpesvirus infection	128	0.386600225	1.556482398	0.001473002	0.011919869	0.007792	4779	tags=55%, list=38%, signal=34%
hsa00630	Glyoxylate and dicarboxylate metabolism	23	-0.479130653	-1.991413997	0.001480493	0.011919869	0.007792	2248	tags=48%, list=18%, signal=39%
hsa00620	Pyruvate metabolism	32	-0.443207168	-1.983535929	0.001521245	0.011941772	0.007806	2248	tags=50%, list=18%, signal=41%
hsa05162	Measles	87	0.41047052	1.591218705	0.001728699	0.013239301	0.008655	3099	tags=39%, list=25%, signal=30%
hsa04371	Apelin signaling pathway	79	0.429206219	1.648401923	0.001771041	0.013240641	0.008655	4015	tags=53%, list=32%, signal=36%
hsa05146	Amoebiasis	53	0.465216118	1.709163279	0.001816844	0.013267186	0.008673	3422	tags=42%, list=27%, signal=30%
hsa04146	Peroxisome	55	-0.329270356	-1.703996618	0.001880927	0.013422976	0.008775	3013	tags=44%, list=24%, signal=33%
hsa04014	Ras signaling pathway	123	0.380193134	1.524347069	0.001946255	0.013580532	0.008878	3460	tags=41%, list=27%, signal=30%
hsa04914	Progesterone-mediated oocyte maturation	53	0.461557119	1.695720438	0.002254851	0.015255914	0.009973	3654	tags=49%, list=29%, signal=35%
hsa04210	Apoptosis	96	0.398497492	1.56237461	0.002283529	0.015255914	0.009973	4647	tags=52%, list=37%, signal=33%
hsa01522	Endocrine resistance	61	0.447629412	1.675426249	0.002391291	0.015643028	0.010226	3460	tags=48%, list=27%, signal=35%
hsa00220	Arginine biosynthesis	10	-0.680066728	-2.125580653	0.002514969	0.016116333	0.010535	2185	tags=50%, list=17%, signal=41%
hsa04935	Growth hormone synthesis, secretion and action	75	0.411763241	1.575191073	0.002979173	0.018709205	0.01223	3748	tags=43%, list=30%, signal=30%
hsa04540	Gap junction	53	0.453920787	1.667665223	0.00305301	0.018796964	0.012288	3460	tags=53%, list=27%, signal=38%
hsa04270	Vascular smooth muscle contraction	78	0.410285365	1.572657144	0.003453751	0.020855344	0.013633	2679	tags=40%, list=21%, signal=31%
hsa05323	Rheumatoid arthritis	46	-0.331737961	-1.635071445	0.003611175	0.020912476	0.013671	3141	tags=54%, list=25%, signal=41%
hsa00830	Retinol metabolism	19	-0.495817963	-1.889096669	0.003653594	0.020912476	0.013671	1293	tags=42%, list=10%, signal=38%
hsa01230	Biosynthesis of amino acids	48	-0.325977867	-1.628670714	0.003663013	0.020912476	0.013671	1497	tags=33%, list=12%, signal=29%
hsa00380	Tryptophan metabolism	24	-0.452066646	-1.900595768	0.004450755	0.024956017	0.016314	2248	tags=58%, list=18%, signal=48%
hsa04115	p53 signaling pathway	47	0.459003281	1.643789863	0.005097821	0.028082732	0.018358	4223	tags=55%, list=33%, signal=37%
hsa04071	Sphingolipid signaling pathway	78	0.402795394	1.543947474	0.00529447	0.028663168	0.018737	4426	tags=51%, list=35%, signal=33%
hsa00410	beta-Alanine metabolism	19	-0.478106852	-1.821616257	0.005610104	0.029857161	0.019518	3086	tags=58%, list=24%, signal=44%
hsa00531	Glycosaminoglycan degradation	15	0.620410722	1.771611117	0.005979013	0.03129017	0.020455	3041	tags=60%, list=24%, signal=46%
hsa05225	Hepatocellular carcinoma	103	0.379642483	1.496815678	0.006293095	0.032393965	0.021176	4730	tags=53%, list=38%, signal=34%

hsa05214	Glioma	49	0.436962672	1.579742776	0.007016852	0.035536959	0.023231	2548	tags=41%, list=20%, signal=33%
hsa05168	Herpes simplex virus 1 infection	265	0.317367139	1.340199156	0.007175897	0.035765581	0.02338	4702	tags=45%, list=37%, signal=29%
hsa03050	Proteasome	41	0.467987597	1.635926854	0.008508164	0.041036137	0.026826	2656	tags=41%, list=21%, signal=33%
hsa05032	Morphine addiction	36	0.477748925	1.636587317	0.008605068	0.041036137	0.026826	1845	tags=36%, list=15%, signal=31%
hsa00534	Glycosaminoglycan biosynthesis - heparan sulfate / heparin	14	0.612463637	1.705611553	0.00862543	0.041036137	0.026826	2534	tags=57%, list=20%, signal=46%
hsa04330	Notch signaling pathway	44	0.448242991	1.589136591	0.008953089	0.041959254	0.027429	3645	tags=52%, list=29%, signal=37%
hsa04530	Tight junction	108	0.376224859	1.491610681	0.010597862	0.048937189	0.03199	2777	tags=38%, list=22%, signal=30%
hsa05145	Toxoplasmosis	72	0.401825368	1.534242449	0.010787508	0.04909098	0.032091	3126	tags=39%, list=25%, signal=29%
