# Supplementary material for: Identification of Diagnostic Markers in Infantile Hemangiomas
Source: J Oncol. 2022 Dec 1;2022:9395876. doi: 10.1155/2022/9395876 (PMC9731762; doi:10.1155/2022/9395876)
Supplement: Supplementary Materials — Table S1: DEGs of IHs in the 6-month-old compared to normal samples. Table S2: DEGs of IHs in the 12-month-old compared to normal samples. Table S3: DEGs of IHs in the 24-month-old compared to normal samples. Table S4: common up- and down-regulated genes among the 6-, 12-, and 24-month-old IHs samples. Table S5: GO and KEGG analysis of candidate genes. Table S6: the top 20 significant genes listed by the SVM-RFE algorithm ranked in 127 candidate genes for characteristics. Table S7: GO items relevant to diagnostic genes. Table S8: all functional annotation enrichment analysis results of the identified diagnostic genes. Table S9: all potential compounds are associated with the identified diagnostic genes. Table S10: potential compounds are associated with the major transcription factors. [file 9395876.f1.zip › Supplementary Table S9.pdf]

Table S9. All potential compounds associated with the identified diagnostic genes

GeneSymbol	ChemicalName	InteractionActions
TMEM2	abrine	decreases^expression
TMEM2	Benzo(a)pyrene	increases^expression|decreases^methylation
GUCY1A2	Aflatoxin B1	decreases^expression
GUCY1A2	Amiodarone	increases^expression
GUCY1A2	Arbutin	decreases^expression
GUCY1A2	aristolochic acid I	decreases^expression
GUCY1A2	Benzo(a)pyrene	increases^expression|increases^methylation
GUCY1A2	butyraldehyde	decreases^expression
GUCY1A2	Doxorubicin	affects^expression
GUCY1A2	entinostat	decreases^expression
GUCY1A2	Fonofos	increases^methylation
GUCY1A2	kojic acid	decreases^expression
GUCY1A2	Lead	affects^expression
GUCY1A2	Methapyrilene	increases^methylation
GUCY1A2	methyleugenol	decreases^expression
GUCY1A2	Nickel	decreases^expression
GUCY1A2	Parathion	increases^methylation
GUCY1A2	Particulate Matter	decreases^expression
GUCY1A2	p-Chloromercuribenzoic Acid	decreases^expression
GUCY1A2	Phenylmercuric Acetate	increases^expression
GUCY1A2	Plant Extracts	decreases^expression
GUCY1A2	Silicon Dioxide	increases^expression
GUCY1A2	Temozolomide	increases^expression

GUCY1A2	terbufos	increases^methylation
GUCY1A2	trichostatin A	decreases^expression
GUCY1A2	Triclosan	decreases^expression
ISL1	1-Methyl-4-phenylpyridinium	decreases^expression
ISL1	4-(5-benzo(1,3)dioxol-5-yl-4-pyridin-2-yl-1H-imidazol-2-yl)benzamide	increases^expression
ISL1	4-chloro-N-((4-(1,1-dimethylethyl)phenyl)methyl)-3-ethyl-1-methyl-1H-pyrazole-5-carboxamide	decreases^expression
ISL1	Aflatoxin B1	decreases^methylation
ISL1	Antimycin A	decreases^expression
ISL1	Arachidonic Acid	decreases^expression
ISL1	Arsenic	affects^methylation
ISL1	Benzo(a)pyrene	increases^expression|decreases^methylation
ISL1	beta-methylcholine	affects^expression
ISL1	butyraldehyde	increases^expression
ISL1	Carbamazepine	affects^expression
ISL1	Cyclosporine	decreases^expression
ISL1	Diethylhexyl Phthalate	increases^expression
ISL1	dorsomorphin	increases^expression
ISL1	mercuric bromide	increases^expression
ISL1	Panobinostat	increases^expression
ISL1	Phenylmercuric Acetate	increases^expression
ISL1	Pioglitazone	increases^expression
ISL1	Ribavirin	decreases^expression
ISL1	Silicon Dioxide	decreases^expression
ISL1	sodium arsenite	affects^methylation

ISL1	Sunitinib	decreases^expression
ISL1	Tetrachlorodibenzodioxin	increases^expression
ISL1	thifluzamide	decreases^expression
ISL1	Tobacco Smoke Pollution	decreases^expression
ISL1	triacsin C	decreases^expression
ISL1	trichostatin A	increases^expression
ISL1	Triclosan	decreases^expression
ISL1	Valproic Acid	increases^expression
ISL1	Vorinostat	increases^expression
WARS	aristolochic acid I	increases^expression
WARS	Benzo(a)pyrene	affects^methylation
WARS	bisphenol A	affects^expression
WARS	erucylphospho-N,N,N-trimethylpropylammonium	increases^expression
WARS	Gallic Acid	increases^expression
STEAP4	7,8-Dihydro-7,8-dihydroxybenzo(a)pyrene 9,10-oxide	decreases^expression
STEAP4	Aflatoxin B1	decreases^methylation
STEAP4	Air Pollutants, Occupational	decreases^expression
STEAP4	Antirheumatic Agents	decreases^expression
STEAP4	Arsenic	decreases^expression
STEAP4	Asbestos, Crocidolite	increases^expression
STEAP4	Benzo(a)pyrene	increases^expression|affects^methylation
STEAP4	Cadmium Chloride	increases^expression
STEAP4	Calcitriol	increases^expression
STEAP4	Dihydrotestosterone	increases^expression
STEAP4	Estradiol	increases^expression
STEAP4	incobotulinumtoxinA	increases^expression

STEAP4	licochalcone B	decreases^expression
STEAP4	Metribolone	increases^expression
STEAP4	N,N,N',N'-tetrakis(2-pyridylmethyl)ethylenediamine	increases^expression
STEAP4	Particulate Matter	decreases^expression
STEAP4	pentabromodiphenyl ether	increases^expression
STEAP4	Progesterone	increases^expression
STEAP4	Silicon Dioxide	increases^expression
STEAP4	Smoke	decreases^expression
STEAP4	sodium arsenate	decreases^expression
STEAP4	sulforaphane	decreases^expression
STEAP4	Testosterone	increases^expression
STEAP4	Tetrachlorodibenzodioxin	increases^expression
STEAP4	Tobacco Smoke Pollution	affects^expression
STEAP4	Tretinoin	increases^expression
STEAP4	Troglitazone	decreases^expression
STEAP4	Zinc Sulfate	decreases^expression
